# The global, regional, and national burden of cancer, 1990–2023, with forecasts to 2050: a systematic analysis for the Global Burden of Disease Study 2023

**DOI:** 10.1016/S0140-6736(25)01635-6

**Published:** 2025-09-24

**Authors:** GBD 2023 Cancer Collaborators

**Keywords:** cancer, neoplasms, global burden of disease, DALYs, mortality, incidence

## Abstract

**Background:**

Cancer is a leading cause of death globally. Accurate cancer burden information is crucial for policy planning, but many countries lack up-to-date cancer surveillance data. To inform global cancer-control efforts, we used the Global Burden of Diseases, Injuries, and Risk Factors Study (GBD) 2023 framework to generate and analyse estimates of cancer burden for 47 cancer types or groupings by age, sex, and 204 countries and territories from 1990 to 2023, cancer burden attributable to selected risk factors from 1990 to 2023, and forecasted cancer burden through 2050.

**Methods:**

Cancer estimation in GBD 2023 used data from population-based cancer registration systems, vital registration systems, and verbal autopsies. Cancer mortality was estimated using ensemble models, with incidence informed by mortality estimates and mortality-to-incidence ratios (MIRs). Prevalence estimates were generated from modelled survival estimates, then multiplied by disability weights to estimate years lived with disability (YLDs). Years of life lost (YLLs) were estimated by multiplying age-specific cancer deaths by the GBD standard life expectancy at the age of death. Disability-adjusted life-years (DALYs) were calculated as the sum of YLLs and YLDs. We used the GBD 2023 comparative risk assessment framework to estimate cancer burden attributable to 44 behavioural, environmental and occupational, and metabolic risk factors. To forecast cancer burden from 2024 through 2050, we used the GBD 2023 forecasting framework, which included forecasts of relevant risk factor exposures and used Socio-demographic Index (SDI) as a covariate for forecasting the proportion of each cancer not affected by these risk factors. Progress towards the United Nations’ Sustainable Development Goal (SDG) target 3.4 aim to reduce non-communicable disease mortality by one-third between 2015 and 2030 was estimated for cancer.

**Findings:**

In 2023, excluding non-melanoma skin cancers, there were 18.5 million (95% uncertainty interval 16.4–20.7) incident cases of cancer and 10.4 million (9.65–10.9) deaths, contributing to 271 million (255–285) DALYs globally. Of these, 57.9% (56.1–59.8) of incident cases and 65.8% (64.3–67.6) of cancer deaths occurred in low-income to upper-middle-income countries based on World Bank income group classifications. Cancer was the second leading cause of deaths globally in 2023 after cardiovascular diseases. There were 4.33 million (3.85–4.78) risk-attributable cancer deaths globally in 2023, comprising 41.7% (37.8–45.4) of all cancer deaths. Risk-attributable cancer deaths increased by 72.3% (57.1–86.8) from 1990 to 2023, while overall global cancer deaths increased by 74.3% (62.2–86.2) over the same period. The reference forecasts (the most likely future) estimate that in 2050 there will be 30.5 million (22.9–38.9) cases and 18.6 million (15.6–21.5) deaths from cancer globally, 60.7% (41.9–80.6) and 74.5% (50.1–104.2) increases from 2024, respectively. These forecasted increases in deaths are greater in low- and middle-income countries (90.6% [61.0–127.0]) compared to high-income countries (42.8% [28.3–58.6]). Most of these increases are likely due to demographic changes, as age-standardised death rates are forecast to change by −5.6% (−12.8 to 4.6) between 2024 and 2050 globally. Between 2015 and 2030, the probability of dying due to cancer between the ages of 30 and 70 years was projected to decrease by 6.5% (3.2–10.3).

**Interpretation:**

Cancer is a major contributor to global disease burden, with increasing numbers of cases and deaths forecasted through 2050 and a disproportionate growth in burden in countries with limited resources. The decline in age-standardised mortality rates from cancer is encouraging but insufficient to meet the SDG target set for 2030. Effectively and sustainably addressing cancer burden globally will require comprehensive national and international efforts that consider health systems and context in the development and implementation of cancer-control strategies across the continuum of prevention, diagnosis, and treatment.

**Funding:**

Gates Foundation, St. Jude Children’s Research Hospital, St. Baldrick’s Foundation.

## Introduction

Cancer is a major cause of death globally, impacting individuals and communities in every country.^1,2^ The burden of cancer continues to grow globally,^1^ increasing pressure on health systems across the world. In recognition of this expanding need, multiple *Lancet* commissions have focused on cancer and called for high-quality and equitable care.^3-9^ The World Health Organization (WHO) has established initiatives dedicated to improving global outcomes for specific cancer categories, including breast, cervical, and childhood cancers.^10-12^ As part of the overarching goal of improving health and well-being, the United Nations (UN) Sustainable Development Goals (SDGs) established reducing premature mortality due to cancer and other non-communicable diseases (NCDs) as a target.^13^ Crucial work is being done around the world to address the burden of cancer; however, most countries need to accelerate efforts to meet SDG target 3.4, which aims to “reduce by one-third premature mortality from NCDs”.^13,14^ The COVID-19 pandemic created additional disruptions to achieving target reductions in cancer mortality, with many patients across the world experiencing challenges in accessing screening programmes and necessary care for their cancer diagnoses.^15-17^ There is growing evidence that the COVID-19 pandemic impacted the number of cancer cases diagnosed in some countries, for select cancer types, and particularly in the early pandemic period.^18^ Yet to be fully elucidated is the duration and generalisability of these diagnosis declines and the potential impact of the pandemic on cancer mortality. Any impact on cancer mortality may require time to become apparent and may further challenge the international ability to meet SDG target 3.4.

Cancers, referring to malignant and invasive neoplasms, include a broad spectrum of cancer types, with changes in epidemiology over the life course. There are rapidly evolving diagnostic and treatment opportunities for a subset of cancer types, but major disparities remain across the world in the cancer-control continuum, from risk factor exposures and mitigation to access to early diagnosis, high-quality treatment, and survivorship care.^19-21^ Accurate cancer burden information is crucial to inform effective cancer-control policy, but cancer surveillance data are limited or unavailable in many countries. Furthermore, every country has multiple competing health priorities, underscoring the importance of contextualising the local burden of cancer compared to other diseases impacting a country’s population. Two organisations routinely produce estimates of global cancer burden to address the gaps in cancer data that exist: the Global Burden of Diseases, Injuries, and Risk Factors Study (GBD) from the Institute for Health Metrics and Evaluation and its collaborators and the GLOBOCAN study from the International Agency for Research on Cancer.^1,22^ Both studies use existing data sources to generate statistical estimates of cancer burden where data do not exist but differ in methodological choices and categories of estimates presented, with the GBD study presenting cancer burden estimates across expanded measures (including incidence, prevalence, mortality, years of life lost [YLLs], years lived with disability [YLDs], and disability-adjusted life-years [DALYs]) and across time. Several cancer types important in children, adolescents, and young adults were not historically estimated by either study, and a comprehensive synthesis of global cancer burden across prior decades, with projections of future burden, has not previously been published. The GBD 2023 framework provides global, regional, national, and select subnational burden estimates from 1990 to 2023 for 375 diseases and injuries, including 55 cancer types and groupings (47 of which are reported in this paper), nine of which were newly estimated in GBD 2021.^23-26^ Risk-attributable cancer burden was estimated for behavioural, environmental and occupational, and metabolic risk factors from 1990 to 2023, and projections of cancer burden were estimated through 2050.^23,27^ This manuscript was produced as part of the GBD Collaborator Network and in accordance with the GBD Protocol.^28^

## Methods

### Overview

GBD offers a framework for quantifying the global burden of disease across diseases, injuries, and risk factors using comprehensive and comparable measures of burden. GBD estimates are provided by location, age, sex, and across time, and are updated regularly to provide estimates reflecting newly available data and methodological improvements. Findings from GBD 2023—the current GBD round—supersede those from GBD 2021 and all previous GBD rounds. Our analysis does not reflect the potential impact of the COVID-19 pandemic on cancer burden as these data were too limited to be applied comprehensively across locations and measures at the time of the analysis. Details on data sources and code for this GBD 2023 analysis are available online (https://ghdx.healthdata.org/gbd-2023). This study complies with the Guidelines for Accurate and Transparent Health Estimates Reporting (GATHER) (appendix 1 pp4-5).^29^ The Global Burden of Diseases, Injuries, and Risk Factors Study used de-identified data, and the waiver of informed consent was reviewed and approved by the University of Washington Institutional Review Board (study number 9060).

### Presentation of results

Cancers are defined as C00-C97 in the International Classification of Diseases (ICD), 10^th^ revision, chapter II. Non-melanoma skin cancer estimates are included in one summary result reporting total cancer incidence and deaths globally, and are otherwise excluded from cancer aggregates for consistency across results. Details on specific ICD-10 codes included in the modelling of each GBD cancer cause are defined in appendix 1 pp12-21. GBD 2023 also provides estimates for an additional eight neoplasm types and groupings which are not included in this report but are available online in the GBD Results Tool and GBD Compare visualisation; these include other neoplasms such as myelodysplastic, myeloproliferative, and other hemopoietic neoplasms; and other benign and in situ neoplasms. Detailed results for non melanoma skin cancers are also available in the online GBD tools. Results in this analysis are presented globally and nationally and for four groups of countries based on World Bank income group FY25 classifications (low, lower-middle, upper-middle, and high) for the year 2023.^30^ As not all GBD locations are included in the World Bank classifications, these estimates may not sum to the global estimate. All point estimates are accompanied by 95% uncertainty intervals (UIs). Rates are reported per 100 000 population, and the GBD 2023 world standard population was used for presentation of age-standardised rates (appendix 1 p82).

### Cancer fatal and non-fatal burden estimation, 1990 through 2023

**Figure 1.**
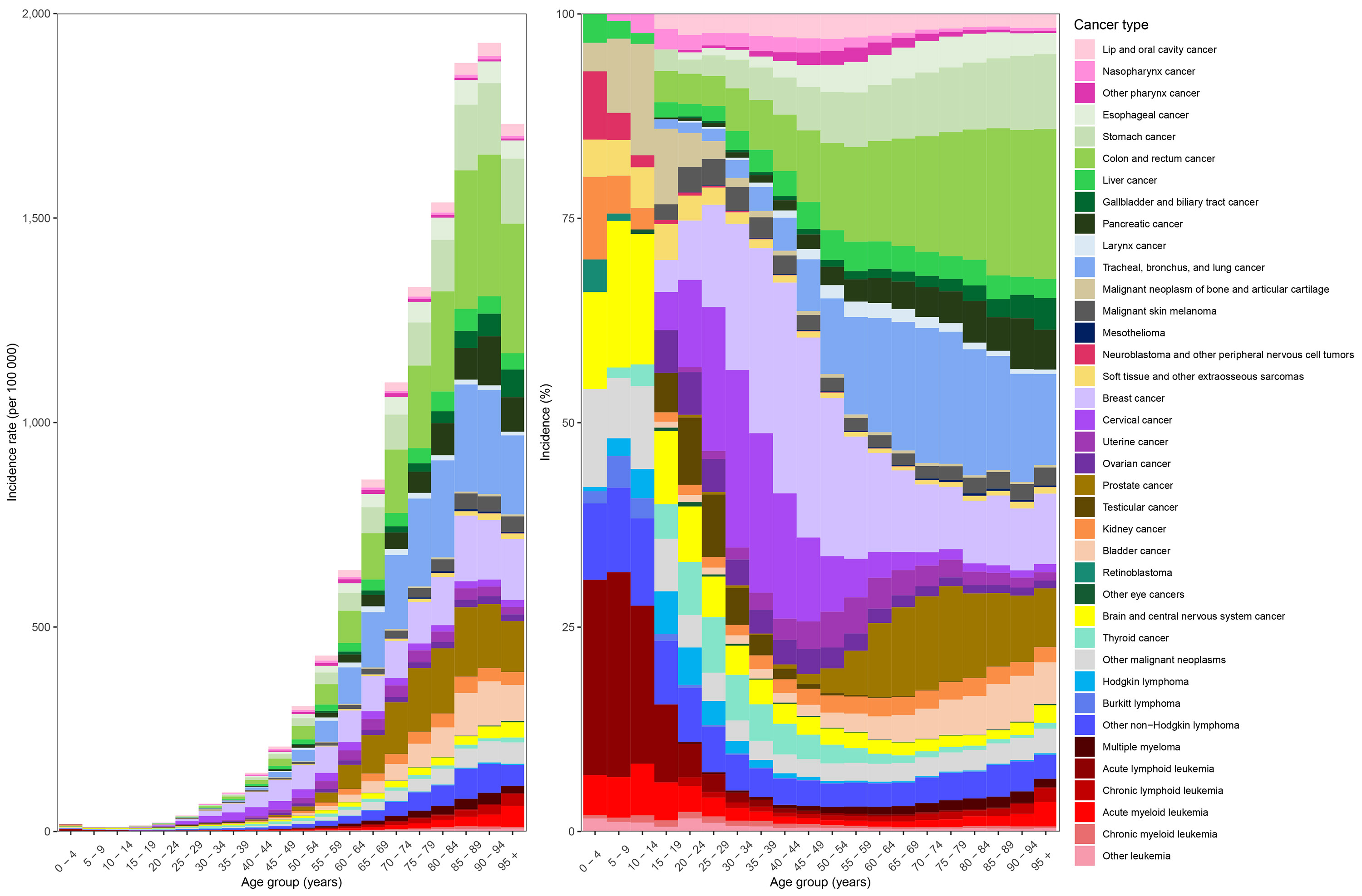

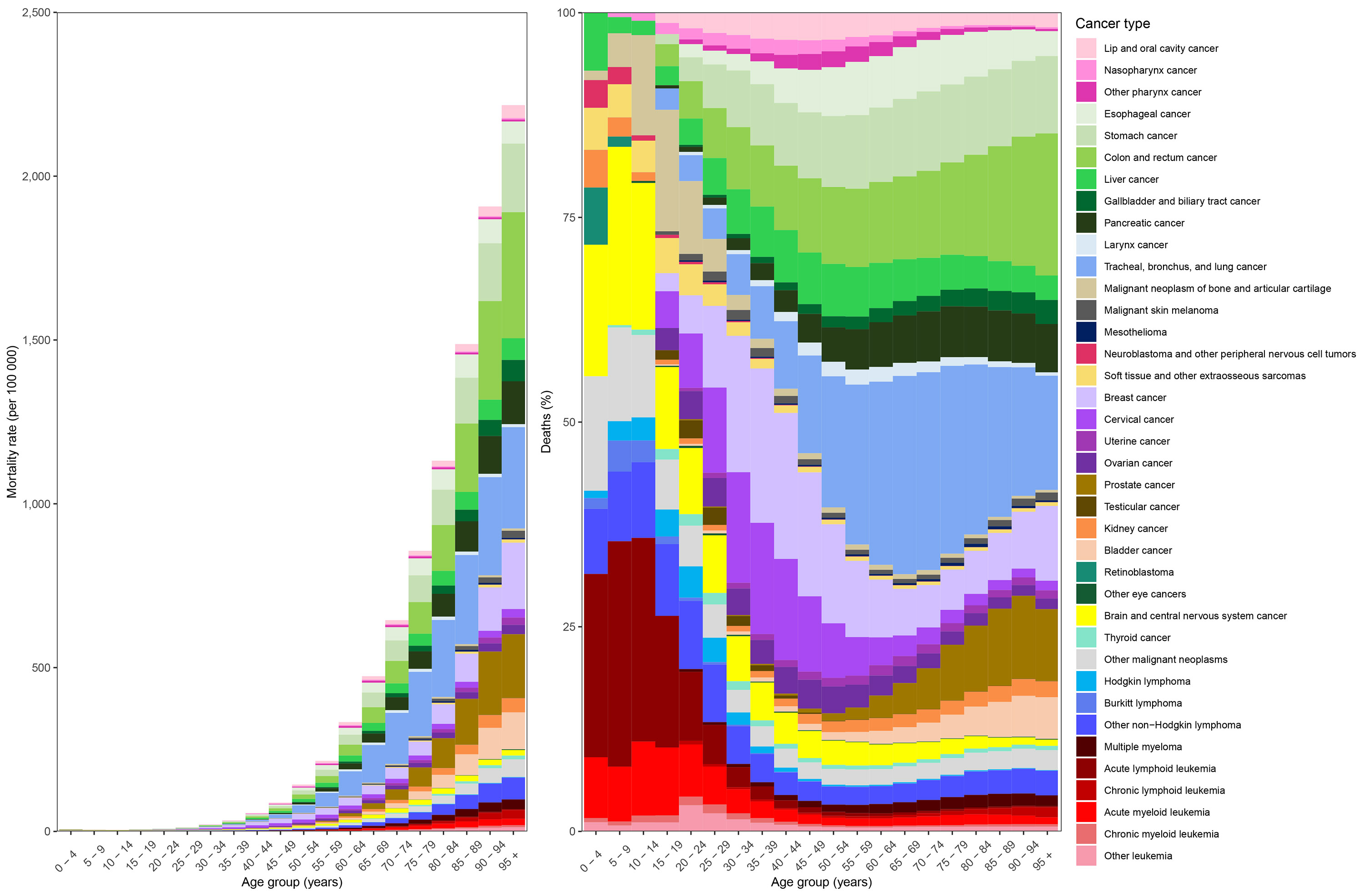
Global age-specific cancer (A) incidence and (B) mortality in 2023 for all sexes combined Results are presented by five-year age group. Left panels present age-specific incidence and mortality rates. Right panels present age-specific new case and death proportions. Several causes are not presented in order to not duplicate burden: eye cancer (Level 3); non-Hodgkin lymphoma (Level 3); leukaemia (Level 3); liver cancer due to hepatitis B, liver cancer due to hepatitis C, liver cancer due to alcohol use, liver cancer due to NASH, liver cancer due to other causes, and hepatoblastoma (Level 4).

**Figure 2.**
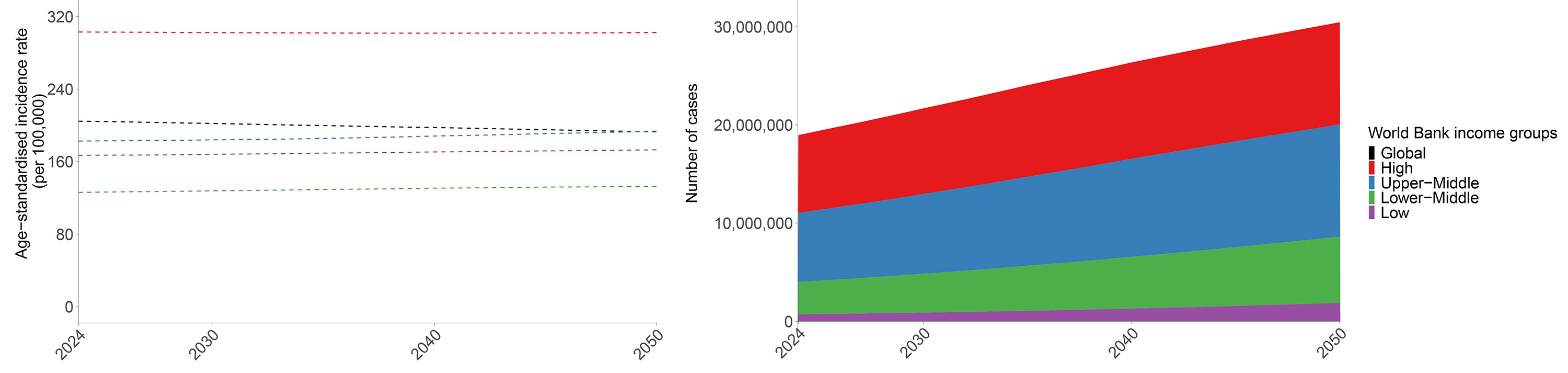

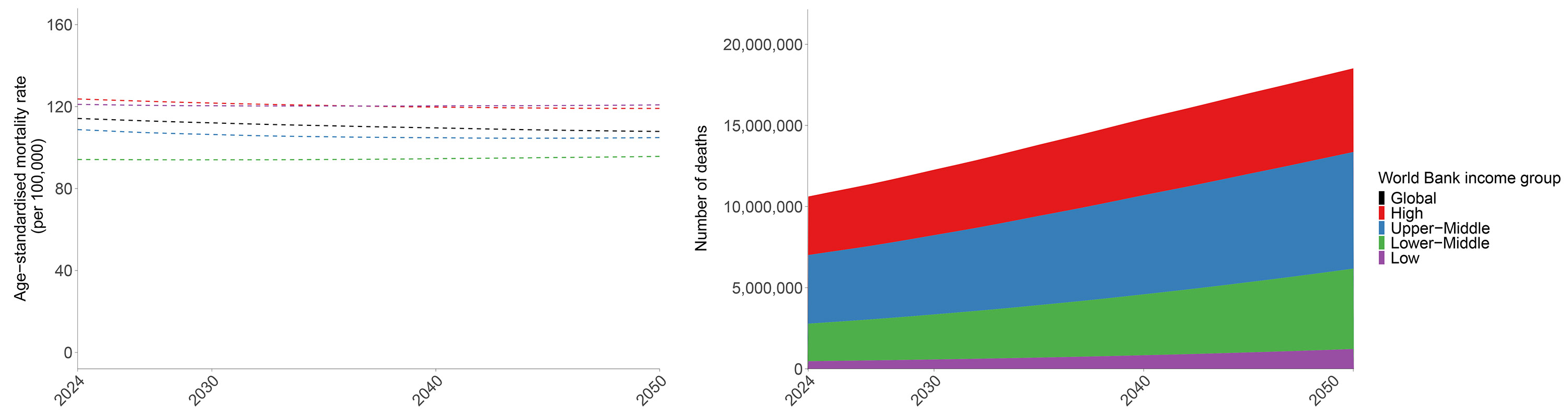
Cancer (A) incidence and (B) mortality in counts and age-standardised rates between 2024 and 2050 globally and by World Bank income group for all ages and sexes combined

GBD 2023 estimates of the fatal and non-fatal burden of cancer were modelled using 4499 site-years from population-based cancer registries (144 new for GBD 2023 compared to GBD 2021), 19 485 site-years from vital registration systems (2097 new), and 421 site-years from verbal autopsy reports (99 new) (appendix 1 table 4, see appendix 2 figures 1-3 for maps of the number of site-years of available cancer data for each data source type). Cancer mortality estimates were made using Cause of Death Ensemble models (CODEm),^31^ based on vital registration and verbal autopsy mortality data, as well as cancer registry incidence data that were converted to mortality estimates using mortality-to-incidence ratios (MIRs). New for GBD 2023, cancer registry data were crosswalked to vital registration data to account for systematic differences, and excluded from CODEm models where directly overlapping (appendix 1 p59). CODEm is a GBD modelling tool that uses all available data to build an ensemble of different statistical models and covariate combinations, to produce cause-specific mortality estimates for each age group, sex, location, and year, with model performance evaluated using out-of-sample predictive validity tests (appendix 1 pp59-74).^24^ Transforming cancer registry incidence data to mortality estimates increased data availability for mortality modelling, especially in locations where mortality data were scarce. MIRs were estimated for each cancer type using either a spatiotemporal Gaussian process regression (ST-GPR) model that used matching incidence and mortality data from population-based cancer registries or,^23,24^ if these data sources were too sparse for a particular cancer type, a negative binomial model (appendix 1 pp56-58). The cancer-specific mortality estimates produced with CODEm, together with estimates for all other causes in GBD, were then scaled to all-cause mortality estimates—which were modelled independently—to produce final estimates of cancer mortality.^32^ YLLs were estimated as the product of the number of cancer-specific deaths for each age group and the standard life expectancy at that age. For full details on estimating the fatal cancer burden for GBD 2023, see appendix 1 p59 and the GBD 2023 Causes of Death Collaborators publication.^24^

Non-fatal cancer-specific estimates were produced by first dividing final cancer mortality estimates by their corresponding MIRs to produce incidence estimates. Ten-year cancer prevalence estimates were then produced using the incidence estimates from the previous step, expected background mortality, and survival estimates modelled from MIRs (appendix 1 p77). Next, these prevalence estimates were split into phases of cancer care to estimate YLDs. Cancer care phases differed depending on whether cohorts were estimated to survive ten years after cancer diagnosis. For surviving cohorts, the phases were: (1) diagnosis and treatment and (2) remission. For the non-surviving cohorts, the phases were: (1) diagnosis and treatment, (2) remission, (3) metastatic or disseminated, and (4) terminal. For five cancers, an additional phase beyond ten years represented ongoing disability from surgical procedures related to cancer treatment. YLD estimates were calculated as the product of the prevalence of each cancer phase and a phase-specific disability weight (appendix 1 pp78-81). Disability weights quantify the magnitude of health loss associated with a particular health outcome, and range from zero, equating to full health, to one, equating to death (appendix 1 p81). Finally, DALYs were calculated as the sum of YLDs and YLLs. For full details on estimating the non-fatal cancer burden for GBD 2023, see appendix 1 p76 and the GBD 2023 Disease and Injury and Risk Factor Collaborators publication.^23^

### Sustainable Development Goal progress

Progress towards achievement of UN SDG target 3.4, which aims to “reduce by one-third premature mortality from NCDs” was estimated specific to cancer.^13^ The probability of dying due to cancer between the ages of 30 and 70 years old was estimated by transforming total cancer (without non-melanoma skin cancer) mortality rates for each age group between 30 and 70 years into probabilities, then summing the probabilities across age groups (appendix 1 pp82-83).

### Cancer risk-attributable burden, 1990 through 2023

GBD 2023 also estimated risk-attributable burden for 44 risk factors associated with 35 cancer types (appendix 2 table 1).^23^ These risk factors are potentially modifiable and categorised into three main categories: occupational and environmental risk factors, metabolic risk factors, and behavioural risk factors, with more detailed categories presented in hierarchical levels. Risk factors and associated cancer pairs were identified for possible inclusion in the analysis based on (1) importance of the risk factor to disease burden or policy, (2) sufficient data and methods to estimate exposure across locations, and (3) evidence that the effects of exposure on the outcome can be generalised across populations. Next, data for risk–outcome associations were identified and extracted through systematic reviews in accordance with the Preferred Reporting Items for Systematic Reviews and Meta-Analyses (PRISMA) framework.^33^ Relative risks for the majority of risk factor–cancer pairs were estimated by synthesising all available relative risk data and estimates of exposure levels, distribution of exposure, and the theoretical minimum risk exposure level (TMREL) using an ensemble spline modelling tool called meta-regression—Bayesian, regularised, trimmed (MR-BRT).^23,34^ MR-BRT captures the shape of relative risk functions (including if the function is non-linear), incorporates different exposure ranges, tests and adjusts for systematic biases, and trims outliers. Select risk factor–cancer pairs were estimated using alternative methods, such as the unsafe sex–cervical cancer pairing, in which a direct population attributable fraction (PAF) strategy was used; more details on methods for estimating each risk factor–cancer pair can be found in the GBD 2023 Disease and Injury and Risk Factors Collaborators publication.^23^

The GBD 2023 comparative risk assessment framework was then used to estimate the proportion of cancer burden attributable to each risk factor, for each relevant cancer type.^23^ PAF is an estimate of the proportional reduction in the risk of cancer that would occur if exposure to a particular risk factor were reduced to the TMREL.

### Forecasting cancer burden, 2024 through 2050

The Institute for Health Metrics and Evaluation forecasting framework was used to forecast cause-specific cancer burden from 2024 through 2050, with reference scenario results presented. In brief, forecasted independent drivers of health, such as age-specific fertility rates by location, age-specific educational attainment by location, and risk factor exposure (measured as summary exposure value) by location, age, and sex are used to forecast cause-specific fatal disease outcomes (deaths and YLLs) by age, sex, and location. A three-component model was used to forecast cancer cause-specific mortality separately for males and females. The model included: 1) risk-deleted (underlying) mortality modelled as a function of Socio-demographic Index (SDI) and time, 2) a risk factor scalar that captures the cause-specific impact of all risk factors that are deemed causal by the GBD comparative risk assessment for the cancer cause in question, and 3) an ARIMA model (random walk with attenuated drift) to capture unexplained residual mortality. The all-cause forecasts constrained the cause-specific forecasts at successively deeper levels of the GBD cause hierarchy using cascading mortality models and thus ensured a robust estimate of cause-specific mortality. For cancers without risk factors associated in GBD 2023, the second component of the model was not performed. The future incidence of cancers was forecasted by dividing forecasted mortality estimates by forecasted MIRs computed using a mixed-effects regression model with SDI as a covariate. Prevalence was computed from incidence using a forecasted prevalence-to-incidence ratio, and YLDs from forecasted prevalence using average disability weights for each cause from the GBD. More details on the forecasting framework are described in the GBD 2021 forecasting capstone publication and appendices.^27^

To ensure forecasted values align with GBD 2023 past estimates, the GBD 2021 forecasts were shifted to the GBD 2023 estimate for the year 2023, maintaining the relative change in values over time. The mean-level ratio of the year 2023 value estimated in GBD 2023 to the year 2023 value forecasted in GBD 2021 was applied to each of the GBD 2021 forecasted years between 2024 and 2050 at the mean level. A similar multiplier was computed for both the upper and lower boundaries (95% UIs) as well, by computing the ratio of the distance between the mean and boundary for the year 2023 in GBD 2023 estimates and the year 2023 in GBD 2021 forecasted estimates. This scalar was applied to the distance between the mean and boundaries for each forecasted year between 2024 and 2050.

### Uncertainty estimation

At each modelling step described above, parameter uncertainty was incorporated by randomly drawing 250 samples from parameter distributions and propagating this uncertainty forward through each subsequent step of the analysis. Final estimates calculated the 2·5^th^ and 97·5^th^ percentile values of the posterior distribution as the 95% UI.

### Role of the funding source

The funder of the study had no role in study design, data collection, data analysis, data interpretation, or the writing of the report.

## Results

### Cancer burden in 2023

There were 18.5 million (95% UI 16.4–20.7) new cancer cases (25.7 million [23.4–28.1] when including non-melanoma skin cancers]) and 10.4 million (9.65–10.9) cancer deaths (10.4 million [9.71–11.0] when including non-melanoma skin cancers]) estimated to occur globally in 2023 (table 1; see GBD Results Tool. Premature mortality comprised the majority of the 271 million (255–285) global cancer DALYs in 2023 (table 1), with 97.0% (95.9–97.8; 263 million [247–277]) of DALYs due to YLLs and 3.0% (2.2–4.1; 8.27 million [5.95–11.4]) due to YLDs (appendix 2 table 11). Globally in 2023, there were 9.56 million (8.51–10.7) new cancer cases in males and 8.99 million (7.85–10.2) in females; 5.77 million (5.37–6.12) cancer deaths in males and 4.61 million (4.17–5.00) cancer deaths in females (appendix 2 tables 2-3). The male-to-female ratio for cancer cases was 1.07 (1.00–1.14) and for cancer deaths 1.25 (1.14–1.36) (appendix 2 table 4).

The cancer types contributing the greatest global burden in terms of incident cases for males and females combined in 2023 were breast cancer (2.30 million [95% UI 2.03–2.61]), tracheal, bronchus, and lung cancer (2.30 million [2.08–2.53]), colon and rectum cancer (2.29 million [2.01–2.55]), prostate cancer (1.41 million [1.19–1.63]), and stomach cancer (1.26 million [1.07–1.52]). The greatest contributors to global cancer deaths for males and females combined in 2023 were tracheal, bronchus, and lung cancer (2.04 million [1.84–2.21] deaths), colon and rectum cancer (1.11 million [1.00–1.20]), stomach cancer (935 000 [797 000–1 060 000]), breast cancer (778 000 [683 000–870 000]), and oesophageal cancer (577 000 [510 000–649 000]) (table 2).

The distribution of cancer across World Bank income groups in 2023 differed for age-standardised incidence and mortality rates. In 2023, the age-standardised cancer incidence rate was highest in the high-income group (303.2 [95% UI 262.9–344.1]) and lowest in the lower-middle-income group (125.7 [112.5–139.7]). Similarly, the highest age-standardised death rate was in the high-income group (124.1 [114.5–129.2]), while the lowest was the lower-middle-income group (94.2 [86.4–103.0]) (table 1).

The cancer type with the highest burden in any given country in 2023 differed by sex. In males, prostate cancer was the most common incident cancer in 112 countries and territories, followed by tracheal, bronchus, and lung cancer (53 countries) and colon and rectum cancer (17 countries) (appendix 2 figure 4). In females, breast cancer was the most common incident cancer in 164 countries and territories, followed by cervical cancer (37 countries) (appendix 2 figure 5). For deaths, tracheal, bronchus, and lung cancer was the leading cause of cancer death in males in 111 countries and territories, followed by prostate cancer (57 countries) and stomach cancer (14 countries) (appendix 2 figure 6). In females, breast cancer was the most common cause of cancer death in 134 countries and territories, followed by tracheal, bronchus, and lung cancer (29 countries) and cervical cancer (27 countries) (appendix 2 figure 7).

Globally in 2023, cancer incidence rates increased with age until the 95 years and over age group, while mortality rates continually increased with age (figure 1). Childhood cancers occurring in the 0–14-year age range comprised 1.4% (95% UI 1.2–1.8) of all cancer cases and 1.0% (0.9–1.1) of all cancer deaths globally in 2023, but 3.2% (2.9–3.6) of all cancer DALYs (appendix 2 table 5). The most common cancer types occurring and causing death in children (0–14 years old) were acute lymphoid leukaemia and brain and central nervous system (CNS) cancers, while carcinomas tended to be more common in the adolescent and young adult (AYA; 15–39 years old) cancer age range. Breast cancer and cervical cancer contributed the greatest share to both incidence and mortality, even for males and females combined, highlighting a disproportionate burden of AYA cancers on females (figure 1). AYA cancers contributed to 7.7% (7.0–8.5) of all cancer cases, 4.6% (4.3–5.1) of all cancer deaths, and 10.6% (10.0–11.4) of all cancer DALYs globally in 2023, and in total 9.1% (8.2–10.1) of cancers globally in 2023 occurred in individuals less than age 40 years (appendix 2 table 5).

Breast cancer was also the largest contributor to cancer incidence in adults 40–64 years of age globally in 2023, followed by colon and rectum cancer and tracheal, bronchus, and lung cancer. For mortality in this age range, tracheal, bronchus, and lung cancer was the largest contributor, followed by breast, colon and rectum, and stomach (figure 1). Cancers in this age group contributed to 38.2% (95% UI 37.1–39.9) of all cancer cases, 32.7% (31.2–34.5) of all cancer deaths, and 45.2% (43.9–46.9) of all cancer DALYs globally in 2023. In contrast, cancers in older adults 65 years and older contributed to 52.7% (50.5–54.3) of all cancer cases, 61.7% (59.5–63.4) of all cancer deaths, and 40.9% (39.1–42.5) of all cancer DALYs globally in 2023, with tracheal, bronchus, and lung cancer and colon and rectum cancer the largest contributors to both incidence and mortality (appendix 2 table 5, figure 1).

### Risk-attributable cancer burden in 1990–2023

Overall, 41.7% (95% UI 37.8–45.4) of cancer deaths globally in 2023 were attributable to potentially modifiable risk factors modelled in GBD 2023 (appendix 2 table 6). Behavioural risk factors were the greatest contributor to risk-attributable cancer deaths globally in 2023, greater than environmental and occupational or metabolic risk categories. Tobacco was the leading Level 2 risk factor for cancer-attributable deaths overall, at 21.4% (18.8–24.3) (appendix 2 table 7). Risk factors contributing to cancer burden differed widely by cancer type, with tobacco contributing to 16 cancers, high alcohol use to ten cancers, dietary risk to six cancers, and unsafe sex solely contributing to one cancer (table 3). Risk-attributable cancer deaths also varied by World Bank income group, greatest in the upper-middle-income group (45.5% [41.0–50.1]) and lowest in the low-income group (31.3% [26.6–35.7]) (appendix 2 table 6). Tobacco was the leading Level 2 risk factor for risk-attributable cancer deaths in each World Bank income group except for the low-income group, in which the leading risk factor was unsafe sex (appendix 2 table 7). A greater proportion of global cancer deaths in 2023 were attributable to estimated risk factors for males (46.0% [41.9–50.3]) than females (36.3% [31.7–40.8]) (appendix 2 table 6). For males globally in 2023, the leading three risk factors contributing to cancer deaths were tobacco, dietary risk factors, and high alcohol use, and for females they were tobacco, unsafe sex, and dietary risk factors (appendix 2 figure 8).

The percentage of cancer deaths which were estimated to be risk-attributable remained similar over time, changing from 42.2% (95% UI 37.7–47.6) in 1990 to 42.0% (37.6–47.0) in 2000, and 42.3% (38.2–46.3) in 2010 (see GBD Results Tool). This change over time was equivalent to a −1.1% (−6.6 to 4.2) change in attributable fractions from 1990 to 2023. The leading three risk factors for both sexes combined in 1990 were tobacco, dietary risks, and air pollution, while in 2023 they were tobacco, dietary risks, and high fasting plasma glucose (see GBD Results Tool).

### Trends in cancer burden, 1990–2023

Between 1990 and 2023, for males and females combined, new cancer cases increased by 105.1% (95% UI 79.5–137.4), and cancer deaths increased by 74.3% (62.2–86.2). In contrast, age-standardised mortality rates due to cancer were estimated to have decreased between 1990 and 2023, by 23.9% (19.1–29.1), while age-standardised incidence rates changed by −7.1% (−18.4 to 7.4) (table 1). Global age-standardised cancer mortality rates decreased similarly in males and females between 1990 and 2023 (appendix 2 table 2-3). Among World Bank income groups, the largest decline in age-standardised mortality rates occurred in the upper-middle-income group, a 33.5% (25.7−41.4) decline, followed by the high-income group (27.3% [25.5–30.1] decline), while increases in age-standardised mortality rates occurred in the lower-middle-income group (16.6% [3.9–32.8]) and were suggestive in the low-income group (14.2% [−0.3 to 31.1]) (table 1). Cancer was the second leading Level 2 cause of death and age-standardised mortality rates globally in 2023, and was consistently ranked second from 1990 through 2023 with the exception of 2021, when respiratory infections and tuberculosis (which include COVID-19) were ranked second, following cardiovascular disease (see GBD Results Tool).^24^

### Cancer burden in the future through 2050

The global number of cancer cases and deaths was forecasted to rise between 2024 and 2050 by 60.7% (95% UI 41.9–80.6) and 74.5% (50.1–104.2), respectively, from 19.0 million (16.8–21.2) to 30.5 million (22.9–38.9) and from 10.6 million (9.92–11.2) to 18.6 million (15.6–21.5) (figure 2; appendix 2 tables 8-9). DALYs were forecasted to increase by 46.2% (29.8–67.2), from 276 million (260–290) to 404 million (356–455). Age-standardised cancer incidence and mortality rates were forecasted to change between 2024 and 2050 by −5.7% (−12.4 to 1.6, from 204.6 [180.9–228.8] to 192.9 [157.0–232.5]) and −5.6% (−12.8 to 4.6, from 114.2 [106.3–120.6] to 107.9 [96.7–119.4]), suggesting that most of the increases in cases and deaths are due to demographic changes (figure 2; appendix 2 tables 8-9). These forecasts were also used to estimate progress of the cancer component of UN SDG target 3.4, which aims by 2030 to reduce premature mortality by one-third from non-communicable diseases between ages 30 and 70. Between 2015 and 2030, the probability of death from cancer between ages 30 and 70 is projected to decrease from 7.0% (6.7–7.3) in 2015 to 6.5% (6.0–7.1) in 2030. The projected decrease of 6.5% (3.2–10.3) in the probability of death from cancer suggests that currently projected cancer reductions are unlikely by themselves to meet the SDG target.

The three leading cancer types for deaths globally in 2024 (tracheal, bronchus, and lung cancer; colon and rectum cancer; and stomach cancer), are each projected to have an increasing number of deaths globally through 2050, increasing by 62.5% (95% UI 26.2–104.0), 93.5% (42.2–154.7), and 22.5% (12.3–33.1), to 3.39 million (2.57–4.29), 2.21 million (1.54–3.07), and 1.16 million (0.959–1.30), respectively (appendix 2 table 10). Global age-standardised mortality rates are projected to decrease for stomach cancer (36.0% [32.6–39.4] decrease), while age-standardised mortality rates for tracheal, bronchus, and lung cancer and colon and rectum cancer are projected to change by −15.2% (−28.9 to 1.1) and −2.0% (−21.7 to 21.5), respectively. Continuing declines in age-standardised mortality rates for lung and stomach cancer are forecasted for each of the four World Bank income groups, though for lung only the high-income group shows a significant decline. For colon and rectum cancers projections there is a trend toward a modest decline in the high-income group and increases in the other income groups, but with wide UIs. For breast, cervical, and childhood cancers, the three cancer types that are part of WHO initiatives to improve global cancer outcomes,^10-12^ forecasts suggest mixed progress by 2050 that varies by World Bank income group. For cervical cancer and childhood cancers, forecasts project modest declines in the age-standardised mortality rates globally, with the largest decreases in the high-income group (25.0% [19.2-30.4] decrease and 18.0% [9.8-24.6] decrease, respectively). For breast cancer, forecasts suggest a stable age-standardised death rate globally, and a decline in higher World Bank income groups while lower World Bank income groups experience an increase, although both with wide UIs (appendix 2 table 10).

## Discussion

Cancer is an increasing global health problem, with rising numbers of cases and deaths impacting health systems worldwide. Cancer is one of four target disease groups in the WHO Global Action Plan for the Prevention and Control of NCDs,^35^ as well as a cornerstone of UN Sustainable Development Goal 3.4.^13^ However, progress towards reducing mortality from cancer and other NCDs is not occurring rapidly enough to achieve established targets globally, and the COVID-19 pandemic has disrupted cancer care and shifted international focus and funding.^15-17,36-38^

We found that cancer deaths increased over the past three decades and are forecasted to continue to increase through 2050. Between 1990 and 2023, the global age-standardised cancer mortality rate decreased by 23.9% (95% UI 19.1–29.1) while the age-standardised incidence rate changed by −7.1% (−18.4 to 7.4), indicating modest improvement in case-fatality rates over this three-decade period. However, progress in the probability of cancer death in ages 30–70 appears to be an insufficient contribution to the SDG target of a reduction in NCD deaths by one-third between 2015 and 2030. Cancer has consistently ranked as the second-leading cause of death globally across the past three decades, including in 2023, with the exception of 2021, when deaths from COVID-19 pushed the respiratory infections and tuberculosis group to rank second.^24^ While age-standardised mortality rates of cancer between 1990 and 2023 decreased globally, the decrease was limited to the World Bank high-income and upper-middle-income groups. Simultaneously, the low- and lower-middle-income groups had the largest increases in age-standardised mortality rates and the largest percentage increases in cancer deaths over this period. This highlights a developing cancer challenge in settings with limited resources and an obligation of the global health community to prioritise equitable cancer-control efforts.

Of the 10.4 million cancer deaths estimated in 2023, 41.7% (95% UI 37.8–45.4) of these were attributable to risk factors included in the GBD 2023 analysis. Reducing exposure to potentially modifiable risk factors could help reduce future cancer burden. However, these GBD estimates do not currently account for several infectious diseases known to be causally associated with cancers and which are more common in low-income countries, resulting in likely underestimates of risk-attributable cancer burden. Although some previous work has captured cancer burden attributable to infections such as with Epstein-Barr virus,^39^ future work is needed to incorporate this and other infectious agents into the full GBD risk factor hierarchy. There was also a substantial portion of cancer deaths estimated to not be avoidable based on currently captured risk factors in the GBD study. This suggests that a combination of interventions addressing exposure to established cancer risk factors, accurate and timely cancer diagnosis, and quality cancer treatment will be needed to simultaneously support individuals developing cancer, while working towards reducing future cancer burden across all settings.

In spite of the substantial and growing contribution of NCDs to the global disease burden, they have received relatively little funding in global health in comparison to communicable diseases.^37,40,41^ Cancers represent a large share of the NCD burden. This discrepancy between burden and funding highlights the need for more monetary support dedicated towards reducing NCDs, including cancer, in the global health arena, as many countries are faced with the double burden of communicable and non-communicable diseases. Insufficiently funding efforts to reduce a major contributor to global disease burden will perpetuate the current disparities, which are evident when comparing cancer outcomes between countries around the world.^42-44^ While the ongoing SDG work is an important motivator and target, much stronger efforts to reduce cancer burden are needed, which will require higher than current levels of funding and prioritisation to be successful.^36,41^

Another necessary component for reducing the burden of cancer is the development of well-functioning health systems that are equipped to address the entire cancer continuum, from prevention, screening, and treatment to palliative care or survivorship. Such broad requirements can make effective cancer care seem complex and challenging, particularly in lower-resource settings. However, there are established strategies to developing and implementing national cancer-control plans.^45,46^ Ideally, such plans are informed by all stakeholders and use established monitoring and evaluation strategies. Essential packages for cost-effective interventions addressing cancer control have been proposed as part of the Disease Control Priorities recommendations for low- and middle-income countries, and WHO has produced summary recommendations of “best buys” for cost-effective cancer services.^47,48^ Cancer often impacts caregivers and families beyond the individual diagnosed with cancer, and cancer treatment has the potential to be financially and psychosocially devastating in countries across the resource spectrum.^14,49-53^ Universal health coverage that includes cancer screening where relevant, diagnosis, treatment, and health care for survivors is crucial to ensuring financial protection for families, access to early identification and care, and equity in outcomes.^54,55^

In addition, there are potential synergies of cancer prevention and care with NCDs more broadly; an investment in certain aspects of capacity to prevent, detect, and treat cancer overlaps with other areas of intervention to address growing NCD burden. Beyond aspects of health system strengthening and financial protections for NCD care, addressing social, environmental, and commercial determinants of health and risk factors for cancers offers opportunities for prevention of a subset of cancers.^56-58^ Several common cancer types globally are associated with risk factors that have opportunities for mitigation. For example, smoking is associated with lung cancer; this risk factor could potentially be reduced through taxation policies.^59,60^ Another example is cervical cancer, which is linked to high-risk types of human papillomavirus (HPV). Vaccination initiatives have the potential to reduce cervical cancer burden,^61,62^ particularly when combined with screening efforts to identify cancers in early stages, where it is feasible and ethical to do so.^63,64^ Tobacco use, high alcohol intake, an elevated BMI, low physical activity, an unhealthy diet, and air pollution are shared risk factors across multiple cancer types as well as other NCDs, with substantial potential for reducing mortality due to NCDs if effectively addressed.^65^ Changes in climate across the world may further alter exposure to risk factors for cancer and other NCDs in the coming years, in addition to disrupting access to treatment, with potential benefits of addressing these environmental impacts across NCDs. Underscoring the importance of decreased exposure to cancer risk factors as one facet of a comprehensive approach to addressing cancer burden should not be conflated with ascribing fault to individual patients who develop cancer types associated with these risk factors. Many behaviours and environmental exposures are influenced by economic factors, the built environment, social policies and norms, and other social determinants of health. Thus, reducing the burden of cancer across countries and worldwide will require both individual action and effective population-level approaches to reduce risk exposures.^66^

Our estimates of total global cancer deaths and incident cases for GBD 2023 are most comparable to the 2022 estimates by GLOBOCAN.^22^ GBD 2023 estimated 18.5 million cancer cases and 10.4 million cancer deaths in 2023, and GLOBOCAN estimated 18.7 million cancer cases and 9.7 million cancer deaths in 2022.^22,67^ There are several differences in the cancer-specific estimates produced by GBD 2023 and GLOBOCAN 2022. Both GBD and GLOBOCAN found that lung cancer and colon and rectum cancer were the first- and second-leading causes of cancer mortality, respectively, but with variation in ranking of following cancer causes of death. While GBD estimated breast cancer as the leading cause of new cancer cases in 2023, followed by lung and colorectal cancer, GLOBOCAN estimated that lung cancer was the leading cause of new cancer cases in 2022, with breast cancer second and colorectal cancer third. These differences in estimates likely stem from differences in data sources, data processing, and modelling strategies. For example, GBD 2023 redistributed non-specific cause of death codes to more specific underlying causes of death, including cancers,^68^ to account for all causes of death in a population. These differences underscore the importance of supporting high-quality cancer surveillance efforts internationally, as estimates are only as reliable as the data informing them. The Global Initiative for Cancer Registry Development is one important example of coordinated international efforts to establish and improve cancer registration endeavours.^69^ The effective and sufficient allocation of resources to support cancer data surveillance will be important for accurate cancer burden information and policy implementation. There are additional differences between GBD 2023 and GLOBOCAN 2022, with both presenting burden estimates for some cancer types not estimated by the other, and GBD 2023 producing cancer burden estimates that include DALYs and generating global estimates across time since 1990. GBD 2023 also provides the ability to compare cancer burden to the burden of other diseases in the same framework of estimation.

GLOBOCAN has provided estimates of cancer incidence attributable to individual cancer risk factors in individual years of estimation, such as alcohol in 2020^70^ and obesity in 2012,^71^ but has not produced a comprehensive summary of risk-attributable cancer burden in the same year that allows for comparison to the GBD results presented here. GLOBOCAN 2022 also forecasted cancer incidence and mortality through 2050,^72^ assuming constant rates and informed by demographic projections. In contrast, the GBD 2023 forecasting framework includes time and independent drivers of disease to forecast cancer mortality, then uses forecasted mortality-to-incidence ratios and disability weights to forecast cancer incidence and DALYs. A key feature of the GBD forecasting framework is that the cause-specific mortality forecasts are directly informed by future risk factor exposures that are deemed causally linked to each cancer according to the GBD comparative risk assessment. GBD forecasts also provide the opportunity to evaluate alternative scenarios that can inform policy decisions, such as eliminating exposure to behavioural and metabolic risk factors by 2050. This, as well as forecasts of additional cancer burden measures such as YLLs, YLDs, and DALYs through 2050 are available online in the GBD forecasting viz tool: https://vizhub.healthdata.org/gbd-foresight/. The GLOBOCAN forecasts estimate that there will be 32.6 million cancer cases and 18.3 million deaths in 2050,^72^ while the GBD forecasts estimate that there will be 30.5 million cancer cases and 18.6 million deaths in 2050, both excluding non-melanoma skin cancers. Apart from GBD’s explicit modelling of risk factors of future cancer burden, the differences between these estimates could also be due to the differences in population projections by the United Nations Population Division, used by GLOBOCAN, and those made for GBD, the latter of which do not incorporate an unlikely assumption of reversal of fertility declines.^32^ In both sets of forecasts, it is clear that global cancer estimation groups predict substantial coming cancer burden if interventions are not intensified and underscore the importance of addressing cancer across the international spectrum of resource availability.

The cancer types newly incorporated in GBD 2021 were particularly relevant to cancer cases and deaths in the childhood and adolescent age groups, and several new cancer types added are index cancers selected for monitoring of progress towards the WHO Global Initiative for Childhood Cancer goals,^10^ which aim to achieve 60% survival and reduce suffering for all children with cancer globally. Trends in global cancer burden among younger age groups will be explored in a separate manuscript. A cancer diagnosis often does not impact only the individual with cancer. Caregivers contribute time and emotional and physical labour that is often uncompensated financially.^73^ More broadly, a cancer diagnosis can lead to negative impacts beyond just the health ramifications, such as disruptions to family finances, employment, or housing security due to health-care costs;^49-53^ stigma,^74^ which may impede other family members seeking medical care; or the death of a single parent, which can contribute to children being orphaned.^75^ Ultimately, these family- and community-level challenges underscore that as cancer burden continues to grow, there will be a growing need for support systems that expand beyond an individual patient with cancer and consider the family and community.

There were limitations to our analysis, many of which are areas for potential future improvements in the GBD study, depending on data availability. Apart from limited vital registration data on cancer deaths in the early COVID-19 pandemic, GBD 2023 cancer estimates and forecasts did not incorporate the impact of the COVID-19 pandemic or recent conflicts on cancer burden, as these data were not available in a geographically and temporally robust and diverse way at the time of our analysis. We hope to be able to understand the impact of COVID-19 on cancer in future analyses as these data become more available over time. Data availability and quality in general can be a challenge, with sparse data from some countries and years. While verbal autopsy studies can help contribute additional information in data-sparse locations, these studies may vary in their ability to accurately capture mortality. For this reason, verbal autopsy data were used for a limited number of cancer types and locations, and the impact of these data is expected to be minimal for most cancer estimates. Uncertainty estimates are provided with all GBD cancer results, but estimates should be interpreted with consideration of data sparsity, and producing statistical estimates of cancer burden does not supplant the need for continued and expanded support of cancer registries and vital registration systems. Furthermore, forecasts of future cancer burden cannot account for potential discoveries and implementation changes in cancer diagnosis and treatment that are currently unknown, and should be interpreted with caution. GBD estimates do not incorporate cancer burden attributable to several infectious diseases, such as *Helicobacter pylori* or *Schistosoma haematobium*, which weigh more heavily on lower-income countries, and therefore likely underestimate risk-attributable cancer burden in those settings. Lifelong burden from surviving cancer is not incorporated into GBD beyond the impact of permanent surgical procedures such as mastectomy, which likely contributes to an underestimation of cancer morbidity, most notably in childhood cancer survivors.

### Conclusion

The global burden of cancer is large and increasing—and expected to continue increasing through 2050—with disproportionate growth occurring in lower-resource settings. The decline in age-standardised mortality rates from cancer is encouraging but insufficient to meet the SDG target set for a reduction in NCD mortality by one-third between 2015 and 2030. The findings from this analysis demonstrate that it is imperative that greater efforts are made to prevent, diagnose, and treat cancer across world regions and economically diverse settings. Despite the clear need for action, there remains a gap in funding and prioritisation of cancer-control policies in global health. Policy makers, governments, and agencies can use these GBD estimates and forecasts in the development and implementation of evidence-based strategies across the cancer continuum locally, nationally, regionally, and globally.

## Data sharing

To download the data used in these analyses, please visit the Global Health Data Exchange (https://ghdx.healthdata.org/gbd-2023).

## Supplementary Material

Methods appendix

Results appendix

Authorship appendix

## Figures and Tables

**Table 1.** Cancer incidence, death, and DALY counts and age-standardised rates in 2023 and percentage change in counts and age-standardised rates between 1990 and 2023 by World Bank income group for all ages and sexes combined

	Global	World Bank income group			
	-	High	Upper middle	Lower middle	Low
Cases 2023 in thousands (UI)	18,500 (16,400 to 20,700)	7,780 (6,700 to 8,760)	6,860 (6,070 to 7,760)	3,190 (2,850 to 3,580)	698 (613 to 797)
Cases, percentage change 1990 to 2023 (UI)	105.1 (79.5 to 137.4)	67.7 (42.9 to 96.2)	117.3 (88.0 to 159.0)	219.5 (170.8 to 277.2)	194.4 (142.8 to 252.9)
Age-standardised incidence rate 2023, per 100 000 (UI)	205.1 (180.9 to 229.1)	303.2 (262.9 to 344.1)	182.7 (160.5 to 207.8)	125.7 (112.5 to 139.7)	166.9 (147.4 to 188.5)
Age-standardised incidence rate, percentage change 1990 to 2023 (UI)	−.1 (−18.4 to 7.4)	−3.4 (−17.9 to 14.8)	−8.8 (−20.9 to 8.9)	28.6 (10.0 to 51.2)	23.6 (3.1 to 43.8)
					
Deaths 2023 in thousands (UI)	10,400 (9,650 to 10,900)	3,530 (3,210 to 3,700)	4,130 (3,800 to 4,440)	2,240 (2,060 to 2,460)	455 (406 to 505)
Deaths, percentage change 1990 to 2023 (UI)	74.3 (62.2 to 86.2)	37.7 (30.2 to 42.3)	68.7 (48.7 to 87.9)	195.6 (163.7 to 234.7)	171.0 (136.8 to 209.7)
Age-standardised mortality rate 2023, per 100 000 (UI)	114.6 (106.5 to 121.0)	124.1 (114.5 to 129.2)	109.3 (100.7 to 117.4)	94.2 ( 86.4 to 103.0)	121.3 (106.1 to 134.7)
Age-standardised mortality rate, percentage change 1990 to 2023 (UI)	−23.9 (−29.1 to −19.1)	−27.3 (−30.1 to −25.5)	−33.5 (−41.4 to −25.7)	16.6 (3.9 to 32.8)	14.2 (−0.3 to 31.1)
					
DALYs 2023 in millions (UI)	271,000 (255,000 to 285,000)	74,000 (69,200 to 77,000)	108,000 (100,000 to 116,000)	71,900 (66,100 to 78,200)	17,400 (15,800 to 19,400)
DALYs, percentage change 1990 to 2023 (UI)	53.4 (42.6 to 64.5)	14.7 (10.2 to 17.8)	39.2 (24.2 to 54.0)	159.2 (129.8 to 193.3)	147.8 (115.9 to 183.3)
Age-standardised DALY rate 2023, per 100 000 (UI)	3,023.1 (2,840.3 to 3,177.3)	2,969.7 (2,806.5 to 3,071.0)	2,890.4 (2,691.4 to 3,107.3)	2,727.6 (2,510.9 to 2,965.9)	3,791.6 (3,385.4 to 4,200.8)
Age-standardised DALY rate, percentage change 1990 to 2023 (UI)	−26.1 (−31.1 to −20.9)	−33.0 (−35.0 to −31.5)	−37.4 (−44.1 to −30.4)	12.7 (0.3 to 27.0)	9.8 (−4.7 to 25.4)

DALY=disability-adjusted life-years. UI=95% uncertainty interval.

**Table 2.** Global cancer incidence, death, and DALY counts and age-standardised rates in 2023 and percentage change in counts and age-standardised rates between 1990 and 2023 for all ages and sexes combined

Cancer type	Incident cases, 2023, in thousands (UI)	Incident cases, percentage change 1990 to 2023 (UI)	Age- standardised incidence rate, 2023 (UI)	Age- standardised incidence rate, percentage change 1990 to 2023 (UI)	Deaths, 2023, in thousands (UI)	Deaths, percentage change 1990 to 2023 (UI)	Age- standardised death rate, 2023 (UI)	Age- standardised death rate, percentage change 1990 to 2023 (UI)	DALYs, 2023, in thousands (UI)	DALYs, percentage change 1990 to 2023 (UI)	Age-standardised DALY rate, 2023 (UI)	Age- standardised DALY rate, percentage change 1990 to 2023 (UI)
**Total Cancers excluding non-elanoma skin cancer**	**18,500 (16,400 to 20,700)**	**105.1 (79.5 to 137.4)**	**205.1 (180.9 to 229.1)**	**−7.1 (−18.4 to 7.4)**	**10,400 (9,650 to 10,900)**	**74.3 (62.2 to 86.2)**	**114.6 (106.5 to 121.0)**	**−23.9 (−29.1 to −19.1)**	**271,000 (255,000 to 285,000)**	**53.4 (42.6 to 64.5)**	**3,023.1 (2,840.3 to 3,177.3)**	**−26.1 (−31.1 to −20.9)**
Lip and oral cavity cancer	422 (361 to 498)	145.5 (98.9 to 209.2)	4.6 (4.0 to 5.5)	11.2 (−9.8 to 39.7)	229 (197 to 267)	133.9 (89.0 to 185.4)	2.5 (2.2 to 2.9)	3.0 (−16.3 to 25.6)	6,540 (5,600 to 7,680)	120.1 (76.4 to 170.9)	72.2 (61.7 to 84.7)	3.8 (−16.5 to 27.6)
Nasopharynx cancer	167 (130 to 218)	86.3 (34.9 to 167.9)	1.9 (1.5 to 2.4)	−7.5 (−33.4 to 32.9)	76 (63.5 to 87.9)	20.9 (−7.7 to 52.5)	0.8 (0.7 to 1.0)	−42.3 (−56.0 to −27.7)	2,600 (2,150 to 3,050)	12.8 (−15.2 to 42.7)	29.2 (24.0 to 34.2)	−42.2 (−56.6 to −26.9)
Other pharynx cancer	191 (159 to 227)	189.2 (136.8 to 263.1)	2.1 (1.7 to 2.5)	31.0 (7.1 to 64.7)	117 (93.8 to 144)	166.2 (112.5 to 242.0)	1.3 (1.0 to 1.6)	18.9 (−4.9 to 52.3)	3,420 (2,680 to 4,240)	151.6 (99.5 to 226.7)	37.3 (29.2 to 46.4)	17.2 (−7.2 to 51.6)
Oesophageal cancer	605 (539 to 668)	53.1 (30.9 to 82.6)	6.6 (5.9 to 7.3)	−32.8 (−42.4 to −20.4)	577 (510 to 649)	51.2 (28.1 to 79.2)	6.3 (5.6 to 7.1)	−34.6 (−44.2 to −22.4)	14,100 (12,600 to 15,800)	37.0 (16.6 to 65.2)	152.9 (136.4 to 171.7)	−38.3 (−47.4 to −25.8)
Stomach cancer	1,260 (1,070 to 1,520)	20.0 (2.0 to 41.5)	13.8 (11.7 to 16.6)	−47.5 (−55.3 to −38.4)	935 (797 to 1,060)	2.4 (−12.6 to 20.3)	10.3 (8.7 to 11.7)	−56.0 (−62.4 to −48.6)	22,500 (19,300 to 26,000)	−8.8 (−22.5 to 7.3)	246.7 (211.9 to 285.5)	−58.4 (−64.5 to −51.3)
Colon and rectum cancer	2,290 (2,010 to 2,550)	142.7 (113.0 to 177.4)	25.1 (22.0 to 28.0)	2.1 (−10.3 to 16.2)	1,110 (1,000 to 1,200)	90.2 (74.4 to 106.0)	12.2 (11.1 to 13.3)	−22.0 (−27.8 to −15.9)	26,200 (24,000 to 28,500)	76.3 (60.4 to 93.4)	287.4 (263.3 to 313.0)	−21.2 (−28.1 to −14.0)
Liver cancer	570 (500 to 639)	109.9 (78.4 to 144.6)	6.3 (5.5 to 7.0)	−3.4 (−17.5 to 12.3)	508 (451 to 571)	104.4 (75.4 to 138.9)	5.6 (4.9 to 6.3)	−7.6 (−20.7 to 7.6)	13,900 (12,200 to 16,100)	76.5 (48.3 to 112.0)	154.8 (135.0 to 179.6)	−13.9 (−27.7 to 3.4)
*Liver cancer due to hepatitis B*	216 (188 to 249)	75.3 (44.5 to 108.8)	2.4 (2.1 to 2.7)	−16.3 (−30.7 to −0.1)	189 (162 to 221)	69.6 (41.3 to 102.9)	2.1 (1.8 to 2.4)	−20.2 (−33.4 to −4.2)	6,020 (5,140 to 7,140)	54.2 (27.2 to 88.6)	66.9 (57.1 to 79.5)	−23.3 (−36.4 to −6.2)
*Liver cancer due to hepatitis C*	169 (141 to 202)	136.8 (101.9 to 175.1)	1.9 (1.6 to 2.2)	1.6 (−13.1 to 16.9)	155 (131 to 188)	130.7 (97.8 to 168.5)	1.7 (1.4 to 2.1)	−2.8 (−16.6 to 12.5)	3,390 (2,810 to 4,160)	101.2 (69.6 to 136.7)	37.0 (30.8 to 45.4)	−10.9 (−24.6 to 4.2)
*Liver cancer due to alcohol use*	107 (85.9 to 133)	159.2 (118.2 to 202.1)	1.2 (0.9 to 1.4)	14.1 (−4.0 to 32.7)	95.2 (76.4 to 118)	150.2 (112.7 to 192.9)	1.0 (0.8 to 1.3)	8.9 (−7.1 to 26.8)	2,440 (1,930 to 3,080)	132.1 (95.1 to 175.5)	26.5 (20.9 to 33.4)	5.5 (−11.3 to 25.2)
*Liver cancer due to NASH*	44.9 (34.4 to 56)	183.8 (138.2 to 238.2)	0.5 (0.4 to 0.6)	26.5 (6.2 to 50.5)	41.7 (31.2 to 52.7)	179.2 (137.1 to 233.4)	0.5 (0.3 to 0.6)	21.9 (3.7 to 45.6)	1,060 (810 to 1,350)	147.9 (107.0 to 201.9)	11.6 (8.9 to 14.9)	16.5 (−2.1 to 42.1)
*Hepatoblastoma*	5.78 (4.33 to 7.93)	−2.2 (−31.9 to 47.7)	0.1 (0.1 to 0.1)	−8.8 (−36.7 to 38.2)	3.53 (2.28 to 5.22)	−21.5 (−50.2 to 27.6)	0.1 (0.0 to 0.1)	−25.7 (−53.0 to 21.1)	310 (200 to 460)	−21.2 (−50.0 to 27.7)	4.8 (3.1 to 7.1)	−25.3 (−52.7 to 21.5)
*Liver cancer due to other causes*	27.3 (21 to 33.8)	95.9 (61.7 to 132.2)	0.3 (0.2 to 0.4)	−6.5 (−21.9 to 11.3)	23.6 (18.3 to 29.5)	89.6 (62.2 to 123.2)	0.3 (0.2 to 0.3)	−11.5 (−23.4 to 4.5)	711 (554 to 886)	65.3 (38.8 to 101.8)	8.0 (6.2 to 9.9)	−16.3 (−29.6 to 1.9)
Gallbladder and biliary tract cancer	227 (197 to 263)	103.4 (82.3 to 128.3)	2.5 (2.2 to 2.9)	−15.4 (−24.1 to −5.2)	185 (161 to 218)	82.8 (63.1 to 103.2)	2.0 (1.8 to 2.4)	−25.2 (−33.2 to −17.1)	4,040 (3,480 to 4,830)	68.7 (49.4 to 89.9)	44.0 (38.0 to 52.7)	−26.5 (−34.6 to −17.5)
Pancreatic cancer	574 (518 to 609)	152.8 (132.9 to 172.5)	6.3 (5.7 to 6.7)	6.3 (−1.4 to 14.6)	552 (504 to 587)	150.5 (133.0 to 169.6)	6.0 (5.5 to 6.4)	3.6 (−3.0 to 10.9)	12,300 (11,500 to 13,000)	125.8 (108.5 to 145.9)	133.3 (124.4 to 141.8)	−0.6 (−7.9 to 7.9)
Larynx cancer	234 (197 to 281)	82.8 (44.8 to 121.9)	2.5 (2.1 to 3.0)	−19.2 (−35.7 to −2.1)	132 (113 to 159)	63.1 (33.6 to 94.9)	1.4 (1.2 to 1.7)	−29.1 (−41.8 to −15.2)	3,610 (3,000 to 4,400)	54.6 (25.2 to 86.8)	39.1 (32.5 to 47.7)	−29.8 (−43.2 to −15.3)
Tracheal, bronchus, and lung cancer	2,300 (2,080 to 2,530)	96.0 (77.8 to 117.1)	24.9 (22.5 to 27.4)	−15.0 (−22.8 to −5.9)	2,040 (1,840 to 2,210)	85.8 (68.6 to 100.6)	22.2 (19.9 to 24.1)	−20.3 (−27.7 to −14.1)	46,700 (42,200 to 50,900)	62.0 (47.6 to 75.1)	504.9 (455.7 to 550.0)	−27.5 (−34.0 to −21.6)
Malignant skin melanoma	322 (253 to 397)	150.1 (86.8 to 236.8)	3.6 (2.8 to 4.4)	17.5 (−11.9 to 57.1)	66.2 (59.6 to 75.2)	104.5 (88.0 to 118.6)	0.7 (0.7 to 0.8)	−10.3 (−17.6 to −3.9)	1,810 (1,590 to 2,120)	74.6 (56.1 to 89.1)	20.3 (17.7 to 23.8)	−15.4 (−24.3 to −8.0)
Soft tissue and other extraosseous sarcomas	137 (107 to 171)	111.6 (51.3 to 185.1)	1.6 (1.2 to 2.0)	11.0 (−19.5 to 47.3)	60.8 (49 to 75.7)	89.1 (33.7 to 142.8)	0.7 (0.6 to 0.9)	−4.5 (−31.2 to 21.9)	2,210 (1,720 to 2,880)	57.8 (5.0 to 110.2)	26.6 (20.4 to 34.9)	−5.1 (−35.6 to 26.2)
Malignant neoplasm of bone and articular cartilage	109 (81.4 to 157)	86.4 (23.5 to 196.2)	1.3 (1.0 to 1.9)	8.7 (−27.9 to 72.9)	76.3 (59.9 to 96.3)	88.1 (41.7 to 147.7)	0.9 (0.7 to 1.1)	−0.8 (−24.1 to 29.2)	2,990 (2,260 to 3,890)	58.3 (13.1 to 114.4)	35.9 (26.8 to 47.0)	−1.3 (−29.0 to 33.2)
Breast cancer	2,300 (2,030 to 2,610)	146.0 (111.0 to 187.8)	25.5 (22.4 to 29.0)	12.8 (−3.3 to 32.0)	778 (683 to 870)	111.1 (85.4 to 139.2)	8.7 (7.6 to 9.7)	−7.8 (−18.7 to 4.5)	24,500 (21,500 to 27,800)	107.7 (77.7 to 139.9)	274.4 (239.7 to 311.4)	−1.0 (−14.8 to 14.5)
Cervical cancer	867 (676 to 1,140)	86.2 (40.3 to 152.6)	9.9 (7.7 to 13.0)	−4.4 (−27.7 to 29.0)	368 (290 to 476)	70.9 (32.3 to 133.0)	4.1 (3.2 to 5.3)	−19.6 (−37.7 to 8.8)	13,200 (10,200 to 17,300)	70.1 (29.1 to 136.0)	149.7 (114.8 to 197.1)	−13.3 (−34.2 to 19.8)
Uterine cancer	519 (440 to 617)	157.8 (107.2 to 223.6)	5.6 (4.8 to 6.7)	14.6 (−7.4 to 43.4)	108 (93 to 126)	85.4 (56.5 to 117.4)	1.2 (1.0 to 1.4)	−21.9 (−33.9 to −9.1)	2,900 (2,450 to 3,370)	76.8 (44.6 to 113.0)	31.6 (26.7 to 36.8)	−20.0 (−34.4 to −4.1)
Ovarian cancer	331 (284 to 394)	109.2 (74.6 to 146.0)	3.7 (3.1 to 4.4)	−2.1 (−17.8 to 15.1)	221 (190 to 254)	108.1 (77.4 to 141.0)	2.4 (2.1 to 2.8)	−9.3 (−22.4 to 4.7)	6,480 (5,450 to 7,650)	104.9 (67.5 to 142.9)	72.0 (60.4 to 85.0)	−2.9 (−20.5 to 15.1)
Prostate cancer	1,410 (1,190 to 1,630)	168.7 (117.1 to 239.2)	15.2 (12.9 to 17.6)	8.8 (−11.7 to 36.6)	469 (413 to 526)	118.2 (95.9 to 148.3)	5.2 (4.6 to 5.9)	−17.2 (−25.6 to −6.1)	8,850 (7,790 to 9,960)	108.1 (84.9 to 137.8)	96.7 (84.9 to 108.7)	−16.2 (−25.4 to −4.5)
Testicular cancer	97.6 (73.4 to 128)	122.5 (50.9 to 225.8)	1.2 (0.9 to 1.5)	40.4 (−4.6 to 104.9)	11.8 (9.56 to 14.5)	54.1 (11.5 to 94.6)	0.1 (0.1 to 0.2)	−12.1 (−36.1 to 10.9)	584 (469 to 730)	48.0 (5.8 to 91.1)	7.1 (5.7 to 8.9)	−6.3 (−32.9 to 20.7)
Kidney cancer	397 (346 to 460)	132.8 (97.7 to 173.9)	4.4 (3.8 to 5.1)	8.3 (−7.4 to 26.7)	165 (147 to 180)	106.1 (90.5 to 122.2)	1.8 (1.6 to 2.0)	−11.0 (−17.9 to −4.5)	4,050 (3,590 to 4,500)	73.7 (58.2 to 88.6)	45.4 (40.1 to 50.3)	−16.4 (−23.8 to −8.7)
Bladder cancer	569 (499 to 643)	105.5 (75.5 to 143.7)	6.2 (5.5 to 7.1)	−13.9 (−26.1 to 1.4)	234 (209 to 258)	91.5 (74.1 to 112.0)	2.6 (2.3 to 2.9)	−24.0 (−30.3 to −16.2)	4,630 (4,210 to 5,140)	68.6 (53.7 to 89.9)	50.7 (46.2 to 56.3)	−28.2 (−34.3 to −19.1)
Brain and central nervous system cancer	377 (315 to 466)	86.4 (50.8 to 131.6)	4.3 (3.6 to 5.4)	0.9 (−18.1 to 25.2)	263 (230 to 311)	82.9 (61.9 to 100.1)	3.0 (2.6 to 3.5)	−6.4 (−16.8 to 1.9)	9,110 (7,920 to 10,900)	41.7 (23.5 to 57.9)	107.4 (93.1 to 128.5)	−16.1 (−26.7 to −7.4)
Eye cancer	20.9 (15.4 to 29.4)	34.4 (−8.4 to 102.3)	0.3 (0.2 to 0.4)	−19.8 (−42.0 to 15.5)	10.1 (7.22 to 14.2)	8.3 (−46.1 to 88.5)	0.1 (0.1 to 0.2)	−34.5 (−65.2 to 13.2)	489 (294 to 810)	−12.7 (−65.5 to 102.8)	6.7 (3.7 to 11.7)	−33.8 (−73.9 to 53.9)
*Retinoblastoma*	5.4 (3.02 to 9.58)	−2.9 (−65.7 to 184.0)	0.1 (0.0 to 0.1)	−9.6 (−68.1 to 164.2)	3.25 (1.28 to 7.1)	−31.6 (−80.6 to 155.9)	0.0 (0.0 to 0.1)	−36.3 (−82.0 to 138.5)	285 (113 to 622)	−31.2 (−80.5 to 156.8)	4.4 (1.7 to 9.5)	−35.9 (−81.8 to 139.6)
*Other eye cancers*	15.5 (11.5 to 22.6)	55.2 (3.2 to 144.6)	0.2 (0.1 to 0.3)	−23.8 (−49.0 to 19.4)	6.81 (5.39 to 8.81)	50.1 (21.5 to 79.6)	0.1 (0.1 to 0.1)	−33.3 (−45.4 to −21.0)	204 (156 to 279)	40.1 (9.9 to 68.0)	2.3 (1.8 to 3.2)	−29.5 (−45.0 to −15.9)
Neuroblastoma and other peripheral nervous cell tumors	20.9 (13.9 to 30.1)	67.9 (0.2 to 202.3)	0.3 (0.2 to 0.4)	33.5 (−21.5 to 137.4)	5.99 (5.01 to 7.6)	68.8 (33.0 to 111.1)	0.1 (0.1 to 0.1)	13.0 (−10.9 to 41.8)	332 (266 to 441)	35.2 (2.8 to 75.1)	4.5 (3.6 to 6.1)	6.2 (−19.6 to 36.6)
Thyroid cancer	297 (220 to 373)	191.4 (104.9 to 327.0)	3.4 (2.5 to 4.2)	46.5 (3.4 to 114.0)	52.5 (44.8 to 61.1)	120.6 (84.5 to 170.1)	0.6 (0.5 to 0.7)	−3.8 (−19.6 to 18.2)	1,560 (1,310 to 1,880)	112.1 (73.1 to 167.9)	17.6 (14.8 to 21.3)	4.4 (−14.9 to 31.5)
Mesothelioma	29.2 (25.7 to 32.2)	99.3 (71.9 to 126.8)	0.3 (0.3 to 0.4)	−14.2 (−26.0 to −2.6)	27.9 (24.6 to 30.8)	105.4 (79.6 to 132.9)	0.3 (0.3 to 0.3)	−13.0 (−23.6 to −1.6)	623 (550 to 691)	80.9 (56.5 to 109.6)	6.9 (6.0 to 7.6)	−18.3 (−29.5 to −5.3)
Hodgkin lymphoma	68.7 (55.8 to 84.3)	15.0 (−7.4 to 36.3)	0.8 (0.7 to 1.0)	−32.8 (−45.7 to −20.3)	27.1 (20.8 to 34.9)	−9.9 (−30.7 to 9.0)	0.3 (0.2 to 0.4)	−51.1 (−62.0 to −41.3)	1,180 (866 to 1,560)	−16.0 (−38.5 to 4.1)	14.3 (10.4 to 19.0)	−47.4 (−61.9 to −34.9)
Non-Hodgkin lymphoma	632 (468 to 849)	142.9 (68.8 to 256.1)	7.1 (5.3 to 9.6)	17.1 (−18.6 to 70.8)	282 (246 to 319)	90.9 (61.0 to 126.3)	3.2 (2.8 to 3.6)	−11.8 (−25.4 to 4.4)	8,290 (7,080 to 9,590)	58.3 (29.6 to 93.0)	96.2 (81.3 to 111.9)	−14.3 (−29.7 to 3.8)
*Burkitt lymphoma*	15.9 (9.67 to 26)	99.9 (6.0 to 280.5)	0.2 (0.1 to 0.3)	34.1 (−30.1 to 152.5)	6.68 (5.04 to 9.56)	57.6 (11.4 to 138.2)	0.1 (0.1 to 0.1)	2.0 (−28.5 to 53.4)	369 (260 to 566)	33.3 (−13.2 to 112.2)	4.8 (3.4 to 7.5)	−0.6 (−35.1 to 59.3)
*Other non-Hodgkin lymphoma*	616 (457 to 825)	144.3 (70.2 to 256.3)	6.9 (5.1 to 9.3)	16.7 (−18.7 to 69.9)	276 (240 to 312)	91.9 (62.2 to 127.8)	3.1 (2.7 to 3.5)	−12.2 (−25.4 to 4.2)	7,920 (6,800 to 9,160)	59.7 (31.2 to 94.8)	91.4 (77.8 to 106.4)	−14.9 (−30.0 to 3.5)
Multiple myeloma	176 (142 to 222)	188.2 (122.3 to 288.7)	1.9 (1.6 to 2.4)	21.1 (−6.4 to 62.8)	125 (112 to 138)	156.9 (130.5 to 186.7)	1.4 (1.2 to 1.5)	5.0 (−5.7 to 16.9)	2,820 (2,520 to 3,130)	141.3 (114.2 to 174.6)	30.7 (27.5 to 34.1)	5.2 (−6.6 to 19.5)
Leukemia	573 (447 to 736)	42.0 (6.0 to 100.7)	6.7 (5.2 to 8.7)	−22.7 (−42.7 to 8.6)	341 (307 to 384)	26.5 (10.5 to 44.7)	3.9 (3.5 to 4.4)	−33.5 (−41.4 to −24.4)	12,100 (10,700 to 14,000)	−7.5 (−20.9 to 9.3)	147.9 (130.3 to 171.4)	−40.8 (−49.3 to −30.5)
*Acute lymphoid leukemia*	128 (91.7 to 181)	−3.2 (−30.3 to 48.9)	1.7 (1.2 to 2.4)	−29.6 (−49.6 to 9.2)	76.9 (53.7 to 98.4)	−15.5 (−32.0 to 6.6)	1.0 (0.7 to 1.2)	−42.7 (−53.7 to −27.7)	4,350 (3,120 to 5,470)	−29.5 (−43.6 to −10.7)	57.1 (41.3 to 71.9)	−46.4 (−56.9 to −32.2)
*Chronic lymphoid leukemia*	128 (101 to 157)	88.8 (44.1 to 149.7)	1.4 (1.1 to 1.7)	−19.1 (−37.8 to 6.8)	44.9 (39.9 to 51.9)	45.2 (27.2 to 65.4)	0.5 (0.4 to 0.6)	−41.6 (−48.3 to −33.9)	985 (863 to 1,190)	23.5 (4.6 to 47.9)	10.9 (9.5 to 13.1)	−44.4 (−52.5 to −34.0)
*Acute myeloid leukemia*	183 (135 to 258)	90.5 (36.9 to 178.9)	2.1 (1.5 to 3.0)	0.4 (−29.4 to 48.4)	131 (113 to 152)	71.8 (45.4 to 103.6)	1.5 (1.3 to 1.7)	−12.0 (−24.1 to 1.7)	4,190 (3,420 to 5,190)	20.3 (−2.0 to 50.1)	49.8 (40.2 to 62.3)	−26.3 (−40.0 to −9.3)
*Chronic myeloid leukemia*	56.8 (37.7 to 80.2)	−10.6 (−43.4 to 47.7)	0.6 (0.4 to 0.9)	−56.0 (−71.9 to −27.1)	26.4 (21.1 to 32.8)	−26.7 (−43.8 to −14.1)	0.3 (0.2 to 0.4)	−65.7 (−73.1 to −55.3)	797 (600 to 1,070)	−39.8 (−55.9 to −15.6)	9.3 (6.9 to 12.5)	−67.0 (−75.7 to −54.3)
*Other leukemia*	76.8 (61.7 to 96.6)	75.4 (35.2 to 128.7)	0.9 (0.7 to 1.1)	−12.0 (−31.0 to 14.3)	61.7 (49.2 to 77.3)	75.3 (37.4 to 133.1)	0.7 (0.6 to 0.9)	−16.8 (−33.7 to 9.1)	1,810 (1,400 to 2,380)	35.1 (1.5 to 89.1)	20.8 (16.1 to 27.4)	−25.8 (−44.3 to 2.5)
Other malignant neoplasms	454 (388 to 530)	78.3 (43.7 to 122.2)	5.1 (4.4 to 6.0)	−12.0 (−28.7 to 9.3)	226 (197 to 252)	46.9 (21.4 to 72.3)	2.6 (2.2 to 2.9)	−30.6 (−42.2 to −19.3)	6,690 (5,730 to 7,680)	14.3 (−8.1 to 38.9)	78.5 (66.9 to 90.5)	−35.7 (−48.2 to −22.3)

Italicised cancer types are Level 4 causes. DALY=disability-adjusted life-years. UI=95% uncertainty interval. NASH=non-alcoholic steatohepatitis. NA=not applicable.

**Table 3.** Global proportion of DALYs attributable to risk factors by cancer cause for all ages and sexes combined

Cancer type	Total risk attributable DALYs, % (UI)	Tobacco attributable DALYs, % (UI)	Dietary risk attributable DALYs, % (UI)	High alcohol use attributable DALYs, % (UI)	Unsafe sex attributable DALYs, % (UI)	Other risk attributable DALYs, % (UI)
**Total cancers excluding non-melanoma skin cancer**	**40.3 (36.4 to 43.9)**	**19.9 (17.5 to 22.5)**	**6.6 (2.2 to 11.0)**	**3.6 (2.0 to 5.4)**	**4.9 (3.8 to 6.2)**	**14.7 (12.3 to 17.6)**
Lip and oral cavity cancer	39.4 (32.2 to 47.0)	35.4 (28.2 to 43.3)	NA	6.8 (1.9 to 11.8)	NA	NA
Nasopharynx cancer	21.9 (17.2 to 27.2)	21.0 (16.4 to 26.2)	NA	NA	NA	1.0 (0.7 to 1.4)
Other pharynx cancer	51.9 (44.7 to 59.1)	43.2 (35.9 to 51.3)	NA	16.5 (13.9 to 18.9)	NA	NA
Oesophageal cancer	56.9 (42.0 to 70.0)	38.1 (32.0 to 44.6)	14.3 (−2.7 to 29.5)	19.6 (−0.8 to 43.3)	NA	NA
Stomach cancer	18.9 (10.3 to 48.7)	11.2 (9.1 to 13.8)	7.2 (0.0 to 40.8)	1.7 (−0.7 to 3.5)	NA	NA
Colon and rectum cancer	55.5 (41.3 to 67.2)	4.8 (3.0 to 6.9)	37.1 (18.2 to 53.4)	5.2 (2.2 to 8.9)	NA	23.8 (18.5 to 29.2)
Liver cancer	46.4 (40.4 to 53.1)	10.8 (3.6 to 18.8)	NA	19.8 (15.8 to 25.9)	NA	24.8 (18.1 to 31.2)
Gallbladder and biliary tract cancer	11.9 (7.7 to 15.7)	NA	NA	NA	NA	11.9 (7.7 to 15.7)
Pancreatic cancer	38.6 (33.7 to 44.6)	15.6 (13.8 to 17.4)	NA	2.1 (0.4 to 2.9)	NA	26.1 (19.4 to 33.5)
Larynx cancer	72.9 (67.1 to 78.0)	68.3 (62.9 to 73.6)	NA	12.5 (3.0 to 23.0)	NA	5.7 (3.4 to 8.7)
Tracheal, bronchus, and lung cancer	77.0 (73.7 to 80.4)	62.9 (59.0 to 66.7)	4.7 (2.3 to 7.4)	NA	NA	39.5 (29.9 to 49.1)
Breast cancer Cervical cancer	28.1 (16.5 to 38.8) 100.0 (100.0 to 100.0)	7.5 (5.2 to 9.9) 5.9 (3.2 to 9.3)	10.8 (0.0 to 23.4) NA	2.2 (1.1 to 3.5) NA	NA 100.0 (100.0 to 100.0)	11.7 (7.3 to 16.1) NA
Uterine cancer	33.7 (24.2 to 42.3)	NA	NA	NA	NA	33.7 (24.2 to 42.3)
Ovarian cancer Prostate cancer	9.9 (4.4 to 15.6) 1.6 (−5.6 to 6.8)	NA 3.6 (1.6 to 5.9)	NA −3.7 (−10.2 to 2.0)	NA 1.6 (0.1 to 3.6)	NA NA	10.1 (4.5 to 15.8) NA
Kidney cancer	27.1 (17.0 to 36.7)	9.4 (5.9 to 13.4)	NA	NA	NA	19.7 (8.6 to 29.6)
Bladder cancer	34.6 (29.7 to 39.8)	27.3 (23.1 to 31.2)	NA	NA	NA	10.2 (6.1 to 15.1)
Thyroid cancer	11.4 (7.9 to 15.8)	NA	NA	NA	NA	11.4 (7.9 to 15.8)
Mesothelioma	84.9 (81.5 to 88.2)	NA	NA	NA	NA	84.9 (81.5 to 88.2)
Non-Hodgkin	4.3 (1.4 to 7.1)	NA	NA	NA	NA	4.3 (1.4 to 7.1)
Multiple myeloma	7.6 (−3.4 to 17.4)	NA	NA	NA	NA	7.6 (−3.4 to 17.4)
Leukaemia	13.3 (9.5 to 17.8)	6.5 (2.3 to 11.0)	NA	NA	NA	7.6 (5.5 to 10.0)

The leading four Level 2 risk factors contributing to cancer DALYs for both sexes combined in 2023 are separately reported. The remainder of risk factors are presented in the “other risk-attributable DALYs” column. Causes without any risk-attributable DALYs include malignant skin melanoma; soft tissue and other extraosseous sarcomas; malignant neoplasm of bone and articular cartilage; testicular cancer; brain and central nervous system cancer; eye cancer; neuroblastoma and other peripheral nervous cell tumours; Hodgkin lymphoma; other malignant neoplasms; and other neoplasms. Details on risk-attributable burden estimation are available in the methods section of this paper and in other publications.^23^ The total column value may be smaller than the sum of the risk factor columns because it accounts for overlapping risk exposures when applicable (including when the ‘other’ column contains multiple risk factors, such as for ovarian cancer). Negative values in this table can occur when risk factors are protective.

DALYs=disability-adjusted life-years. UI=95% uncertainty interval. NA=not applicable because GBD 2023 does not estimate these risk factor–cancer combinations.

## Research in Context

### Evidence before this study

The Global Burden of Diseases, Injuries, and Risk Factors Study (GBD) has produced estimates of cancer burden since its inception in 1993. Over time, additional cancer types have been separately estimated, with additional years of estimates produced for cancer incidence, mortality, years of life lost (YLLs), years lived with disability (YLDs), and disability-adjusted life-years (DALYs) across global, regional, national, and select subnational geographies. For relevant cancer types, the burden attributable to potentially modifiable risk factors has also been estimated across time and geography. Another source of cancer estimates is the Global Cancer Observatory (GLOBOCAN) study from the International Agency for Research on Cancer, which generates estimates for cancer incidence and mortality in single years, with the most recent iteration providing estimates for 36 cancers for the year 2022. Cancer burden attributable to individual risk factors or groups of risk factors (eg, obesity, alcohol, infections) are also produced for single years, although not in each GLOBOCAN round. In the current GLOBOCAN round, forecasts of cancer incidence and mortality are provided through 2050. However, no other research enterprise besides GBD produces, on a regular basis, global estimates of cancer DALYs—a comprehensive measure that accounts for both the mortality and morbidity of cancers—or generates estimates for other disease groups (eg, cardiovascular diseases, respiratory infections and tuberculosis, etc.) with consistent methodology, which allows for comparison of cancer burden to other diseases as part of policy and resource priority setting. For GBD 2023, we estimated cancer burden for 55 cancer types or groupings (47 of which are reported in this paper) from 1990 to 2023; generated estimates of risk-attributable cancer burden across all relevant cancer types from 1990 to 2023; and forecasted cancer deaths, YLLs, and DALYs through 2050.

### Added value of this study

Our GBD 2023 study updates prior GBD round estimates of cancer burden and includes additional data and improvements in methods. We separately report for the first time comprehensive burden estimates for nine new cancer types: soft tissue and other extraosseous sarcomas, malignant neoplasms of the bone and articular cartilage, Burkitt lymphoma, other non-Hodgkin lymphoma, eye cancers (including separate estimates for retinoblastoma and other eye cancers), neuroblastoma and other malignant peripheral nervous cell tumours, and hepatoblastoma. Also, for the first time, we present forecasts of the trajectory of cancer burden through 2050. These estimates can inform ongoing and future cancer-control efforts to address the growing challenge of cancer globally.

### Implications of all the available evidence

Cancer is a major contributor to global disease burden, with inequitable burden weighing on low-income and lower-middle-income countries as their populations age. A substantial proportion of global cancer burden is attributable to known risk factors, which have the opportunity for mitigation through prevention efforts. Cancer burden continues to grow globally, and increasing cancer cases and deaths are expected in the coming decades. Addressing the continued and inequitable growth of cancer globally will require comprehensive and sustainable national and international efforts that consider the unique aspects of health systems and context in the development and implementation of cancer-control strategies.
