# Supplementary material for: The global, regional, and national burden of cancer, 1990–2023, with forecasts to 2050: a systematic analysis for the Global Burden of Disease Study 2023
Source: Lancet. Author manuscript; Available in PMC 2025 Dec 9. (PMC12687902; doi:10.1016/S0140-6736(25)01635-6)
Supplement: Methods appendix [file NIHMS2113905-supplement-Methods_appendix.pdf]

Appendix 1: supplementary methods to "The global, regional, and national burden of cancer from 1990 to 2023 and projections to 2050: a systematic analysis for the Global Burden of Disease Study 2023"

Please note that portions of this supplement detailing cancer incidence, mortality, years of life lost, years lived with disability, and disability-adjusted life-years estimation methods in the Global Burden of Diseases, Injuries, and Risk Factors Study 2023 were copied from the supplementary content to the following GBD 2019 and 2023 publications:

> GBD 2019 Cancer Collaboration. Cancer incidence, mortality, years of life lost, years lived with disability, and disability-adjusted life years for 29 cancer groups from 2010 to 2019: a systematic analysis for the Global Burden of Disease Study 2019. *JAMA Oncol.* 2022; 8:420–44. doi:10.1001/jamaoncol.2021.6987[1]
>
> GBD 2023 Causes of Death Collaborators. Global burden of 292 causes of death in 204 countries and territories and 660 subnational locations, 1990–2023: a systematic analysis for the Global Burden of Disease Study 2023. Lancet (in review).[2] *(Note to editors and reviewers that complete files under review at The Lancet for the GBD 2023 Cause of Death capstone can currently be accessed here: https://cloud.ihme.washington.edu/s/QaxAAkTLGfAxgZb [Password: GBD2023_Capstone]).*
>
> GBD 2023 Disease and Injury and Risk Factor Collaborators. Non-fatal burden of 375 diseases and injuries, risk-attributable burden of 88 risk factors, and healthy life expectancy in 204 countries and territories, including 660 subnational locations, 1990–2023: a systematic analysis for the Global Burden of Disease Study 2023. Lancet (in review).[3] *(Note to editors and reviewers that complete files under review at The Lancet for the GBD 2023 Disease and Injury and Risk Factors capstone can currently be accessed here: https://cloud.ihme.washington.edu/s/B9xQWLwiGiHjGH2 [Password: GBD2023_Capstone]).*

This appendix does not contain details of estimating risk-attributable burden, forecasts through 2050, or demographics for GBD 2023. The comprehensive methodology underlying these analyses can be found in the relevant GBD 2021 and GBD 2023 publications:

> GBD 2021 Risk Factors Collaborators. Global burden and strength of evidence for 88 risk factors in 204 countries and 811 subnational locations, 1990–2021: a systematic analysis for the Global Burden of Disease Study 2021. *Lancet* 2024; published online May 18. https://doi.org/10.1016/S0140-6736(24)00933-4.[4]
>
> GBD 2021 Forecasting Collaborators. Burden of disease scenarios for 204 countries and territories, 2022–2050: a forecasting analysis for the Global Burden of Disease Study 2021. *Lancet 2024;* published online May 18. https://doi.org/10.1016/S0140-6736(24)00685-8.[5]
>
> GBD 2023 Demographics Collaborators. Global age-sex-specific all-cause mortality and life expectancy estimates for 204 countries and territories and 660 subnational locations, 1950–2023: a demographic analysis for the Global Burden of Disease Study 2023. Lancet (in review).[6] *(Note to editors and reviewers that complete files under review at The Lancet for the GBD 2023 Demographics capstone can currently be accessed here: https://cloud.ihme.washington.edu/s/gKJqDyB6BNagEna [Password: GBD2023_Capstone]).*

Table of Contents

Figures and tables ... 3
Statement of GATHER compliance ... 4
The Global Burden of Disease study ... 6
GBD cancer estimation process ... 7
Additional method summaries for all GBD malignant neoplasms except non-melanoma skin cancer ... 9
Cancers in GBD cause hierarchy ... 9
Data sources ... 22
Cancer registry data sources ... 24
Mortality-to-incidence ratio data sources ... 50
Cancer mortality data in the cause of death database other than cancer registry data ... 50
Bias of categories of input data ... 50
Data analysis ... 51
Cancer registry data processing ... 51
CODEm ... 59
Liver cancer aetiology split models ... 74
CoDCorrect ... 76
Calculating years of life lost ... 76
Incidence estimation ... 76
Prevalence estimation ... 77
Disability estimation ... 78
Years lived with disability estimation ... 81
Reporting standards ... 81
Socio-demographic Index (SDI) ... 82
Forecasting ... 82
Probability of death due to cancer ... 82
Interpretation of results ... 84
Changes in GBD 2023 methods and results from GBD 2021 ... 84
Comparison to GLOBOCAN ... 84
Tables reporting GBD estimates globally and by various subgroups ... 84
Limitations ... 85
Additional method summaries for non-melanoma skin cancer (squamous and basal cell carcinoma) ... 86
Case definition ... 86
Input data ... 86
Modelling strategy ... 87
Interpretation of results ... 88
Limitations ... 88
Additional method summary for other neoplasms ... 89
Method summary for myelodysplastic, myeloproliferative, and other haemopoietic neoplasms ... 89
Case definition ... 89
Input data ... 89
Modelling strategy ... 90
Interpretation of results ... 90
Limitations ... 90
Method summary for benign and in situ neoplasms (intestinal; cervical and uterine; and other benign and in situ neoplasms) ... 91
Case definition ... 91
Input data ... 91
Modelling strategy ... 94
Interpretation of results ... 94
Limitations ... 94
Additional methodology tables ... 95
References ... 103

Figures and tables

Figures

Appendix Figure 1: Flowchart of GBD cancer mortality and YLL estimation ... 7
Appendix Figure 2: Flowchart of GBD cancer incidence, prevalence, and YLD estimation ... 8
Appendix Figure 3: Flowchart of GBD non-melanoma skin cancer nonfatal estimation ... 86
Appendix Figure 4: Flowchart of GBD myelodysplastic, myeloproliferative, and other haemopoietic neoplasms nonfatal estimation ... 89
Appendix Figure 5: Flowchart of GBD benign and in situ neoplasms nonfatal estimation ... 91

Tables

Appendix Table 1: GBD cancer cause characteristics in GBD 2023 ... 10
Appendix Table 2: List of ICD codes mapped to GBD cause list for cancer incidence data ... 12
Appendix Table 3: List of ICD codes mapped to GBD cause list for cancer mortality data ... 16
Appendix Table 4: Number of site-years for cancer mortality data by source type, for GBD 2023 compared with GBD 2021 ... 22
Appendix Table 5: Cancer registry sources for cancer incidence, mortality, and mortality-to-incidence ratio data by country, year, and registry ... 24
Appendix Table 6: Undefined cancer code categories (ICD-10) and respective target codes for cancer registry incidence data ... 54
Appendix Table 7: MIR modelling approach for causes newly estimated in GBD 2021 ... 58
Appendix Table 8: Covariates provided to CODEm for each GBD cause, by level and direction, in GBD 2021 and GBD 2023 ... 60
Appendix Table 9: Results for CODEm model performance testing ... 71
Appendix Table 10: Duration of four prevalence phases by cancer ... 78
Appendix Table 11: Disability weights ... 81
Appendix Table 12: ICD codes mapped to non-melanoma skin cancers in clinical informatics data ... 87
Appendix Table 13: ICD codes mapped to myelodysplastic, myeloproliferative, and other haemopoietic neoplasms in clinical informatics data ... 90
Appendix Table 14: ICD codes mapped to benign and in situ neoplasms in clinical informatics data ... 93
Appendix Table 15: World Bank income group by country for GBD 2023 ... 95
Appendix Table 16: GBD location hierarchy: 7 GBD super regions, 21 GBD regions, and 204 countries and territories ... 99

Statement of GATHER compliance

This study complies with the Guidelines for Accurate and Transparent Health Estimates Reporting (GATHER) recommendations. Below is the GATHER checklist.[7]

Item #	Checklist item	Reported on page #
**Objectives and funding**		
1	Define the indicator(s), populations (including age, sex, and geographic entities), and time period(s) for which estimates were made.	Appendix 1 p9
2	List the funding sources for the work.	See main manuscript
**Data Inputs**		
For all data inputs from multiple sources that are synthesised as part of the study:		
3	Describe how the data were identified and how the data were accessed.	Appendix 1 p24
4	Specify the inclusion and exclusion criteria. Identify all ad-hoc exclusions.	Appendix 1 p24
5	Provide information on all included data sources and their main characteristics. For each data source used, report reference information or contact name/institution, population represented, data collection method, year(s) of data collection, sex and age range, diagnostic criteria or measurement method, and sample size, as relevant.	Appendix pp22-51, 86, 89, 91, and https://ghdx.healthdata.org/gbd-2023
6	Identify and describe any categories of input data that have potentially important biases (eg, based on characteristics listed in item 5).	Appendix 1 p50
For data inputs that contribute to the analysis but were not synthesised as part of the study:		
7	Describe and give sources for any other data inputs.	Appendix 1 p24 and https://ghdx.healthdata.org/gbd-2023
For all data inputs:		
8	Provide all data inputs in a file format from which data can be efficiently extracted (eg, a spreadsheet rather than a PDF), including all relevant meta-data listed in item 5. For any data inputs that cannot be shared because of ethical or legal reasons, such as third-party ownership, provide a contact name or the name of the institution that retains the right to the data.	https://ghdx.healthdata.org/gbd-2023
**Data analysis**		
9	Provide a conceptual overview of the data analysis method. A diagram may be helpful.	Appendix 1 Figures 1-5, pp7, 8, 86, 89, 91
10	Provide a detailed description of all steps of the analysis, including mathematical formulae. This description should cover, as relevant, data cleaning, data pre-processing, data adjustments and weighting of data sources, and mathematical or statistical model(s).	Appendix 1 pp51-83, 87, 90, 94
11	Describe how candidate models were evaluated and how the final model(s) were selected.	Found in *Section X: Causes of death modelling methods* of the Supplementary Appendix 1 to "GBD 2023 Causes of Death Collaborators. Global

		burden of 292 causes of death in 204 countries and territories and 660 subnational locations, 1990–2023: a systematic analysis for the Global Burden of Disease Study 2023. Lancet (in review)".[2] *(To the editors and reviewers: note that section detail will be finalized once the GBD 2023 Causes of Death Collaborators publication is final.)* Details of covariate selection for cancer models can be found in: Appendix 1 Table 8, p60.
**12**	Provide the results of an evaluation of model performance, if done, as well as the results of any relevant sensitivity analysis.	Appendix 1 Table 9, p71
**13**	Describe methods for calculating uncertainty of the estimates. State which sources of uncertainty were, and were not, accounted for in the uncertainty analysis.	Appendix 1 pp71, 76, 84, 89, 91
**14**	State how analytic or statistical source code used to generate estimates can be accessed.	https://ghdx.healthdata.org/gbd-2023
**Results and Discussion**		
**15**	Provide published estimates in a file format from which data can be efficiently extracted.	GBD 2023 estimates are available online in the GBD Results Tool and GBD Compare visualisation
**16**	Report a quantitative measure of the uncertainty of the estimates (eg, uncertainty intervals).	See main manuscript, "Results"
**17**	Interpret results in light of existing evidence. If updating a previous set of estimates, describe the reasons for changes in estimates.	See main manuscript, "Discussion"
**18**	Discuss limitations of the estimates. Include a discussion of any modelling assumptions or data limitations that affect interpretation of the estimates.	Appendix 1 pp84, 88, 90, 94

The Global Burden of Disease study

The Global Burden of Diseases, Injuries, and Risk Factors Study (GBD) was created in an effort to establish comprehensive and comparable health metrics. A key principle in the GBD approach to estimation of disease burden is that an individual can have only one cause of death (herein referred to as a "cause"), while recognising that this may underestimate disease burden due to intermediate causes of death. In addition to reporting estimates of mortality and years of life lost for over 300 diseases and injuries, GBD also quantifies non-fatal components of disease including years lived with disability and disability-adjusted life-years, a metric that represents a combination of both the fatal and non-fatal components of disease. The GBD approach uses all relevant data sources, rather than a single type of data. Finally, as there is continual methodological refinement with each GBD iteration, the results in each successive iteration supersede the results of prior GBD studies for the entire newly estimated time series. The protocol for GBD can be found online at http://www.healthdata.org/sites/default/files/files/Projects/GBD/GBD_Protocol.pdf.

GBD cancer estimation process

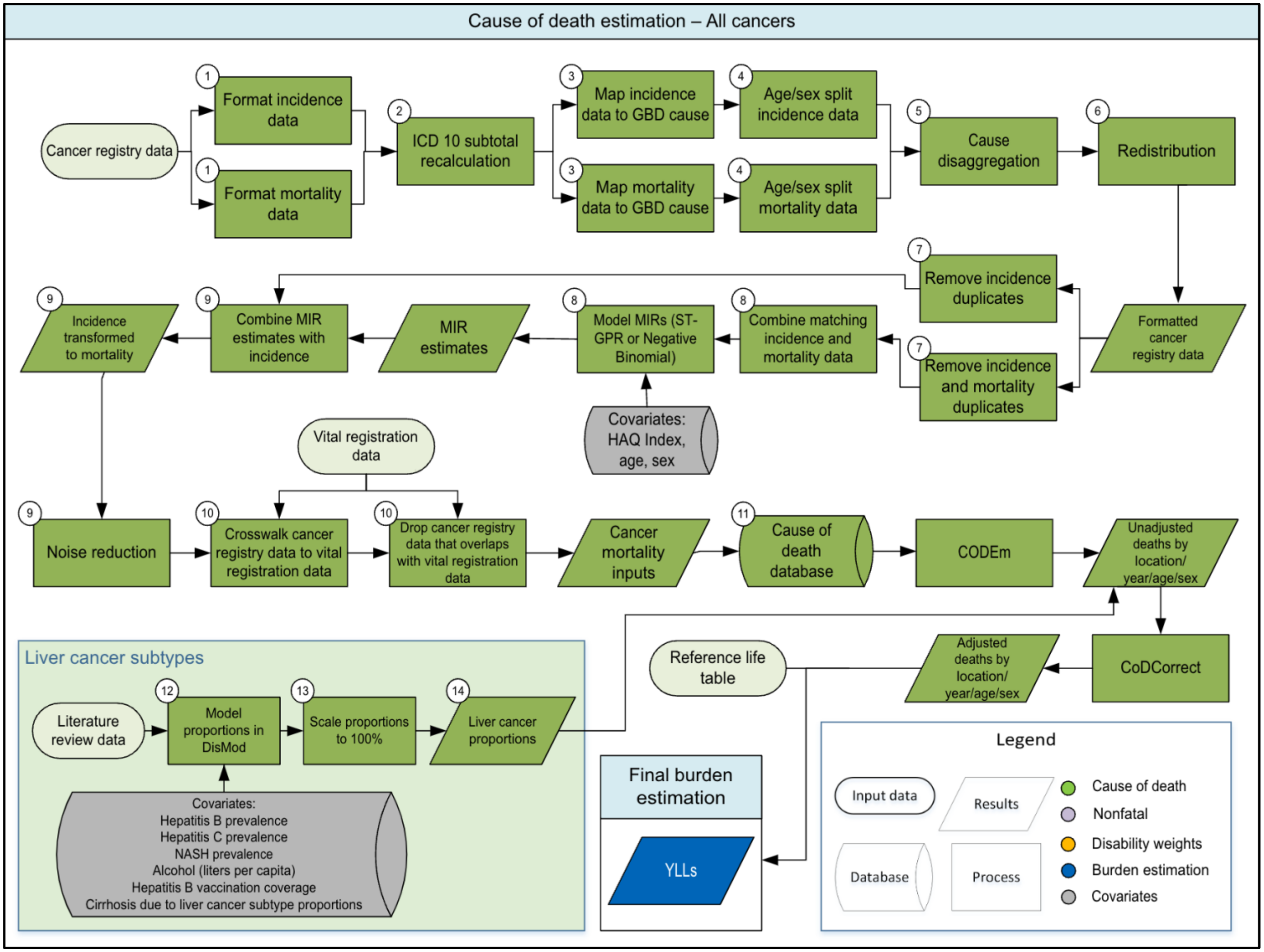

Appendix Figure 1: Flowchart of GBD cancer mortality and YLL estimation. Abbreviations: CODEm, cause of death ensemble model; DisMod-MR, disease model - Bayesian meta-regression; GBD, Global Burden of Disease study; HAQ Index, Healthcare Access and Quality Index; ICD, International Classification of Diseases; MIR, mortality-to-incidence ratio; NASH, nonalcoholic steatohepatitis; ST-GPR, spatiotemporal Gaussian process regression; YLLs, years of life lost.

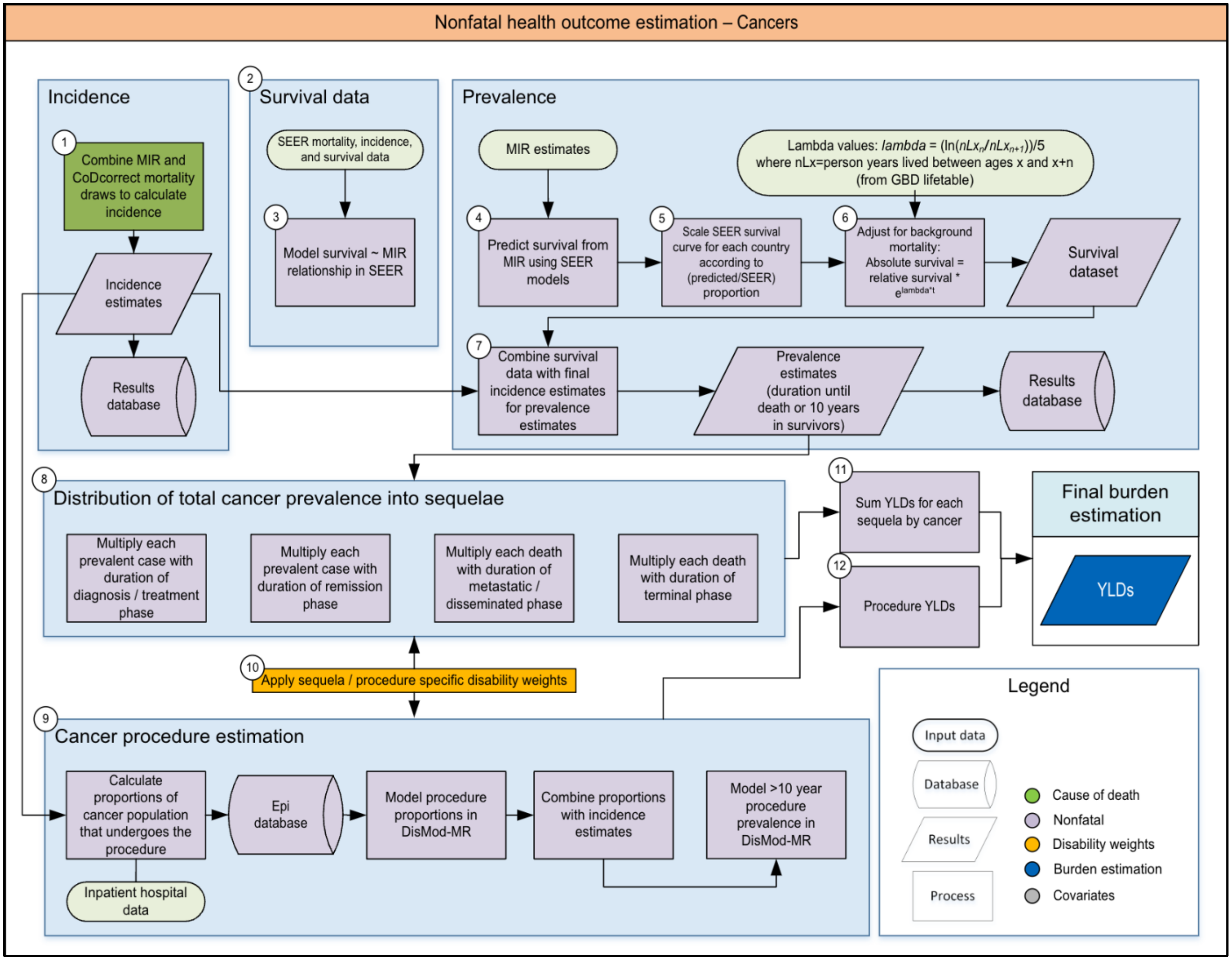

Appendix Figure 2: Flowchart of GBD cancer incidence, prevalence, and YLD estimation. Abbreviations: DisMod-MR, disease model – Bayesian meta-regression; GBD, Global Burden of Disease study; MIR, mortality-to-incidence ratio; SEER, Surveillance, Epidemiology and End Results Program; YLDs, years lived with disability.

Additional method summaries for all GBD malignant neoplasms except non-melanoma skin cancer

Cancers in GBD cause hierarchy

The Global Burden of Diseases, Injuries, and Risk Factors Study (GBD) 2023 cause list is organised in a hierarchy. Levels 1 and 2 represent general groupings of causes, while Levels 3 and 4 represent increasingly specific causes. The general Level 1 group "non-communicable diseases" includes the broad Level 2 group "neoplasms", which includes all malignant and benign neoplasms. Level 3 includes 34 cancer groups, and Level 4 includes 55 groups since the Level 3 parent causes "eye cancer", "leukaemia", "liver cancer", "non-melanoma skin cancer", "other neoplasms", and "non-Hodgkin lymphoma" are further subdivided into 21 additional Level 4 subtypes. Appendix 1 Table 1 lists all GBD cancer causes by cause level, sexes estimated, measures estimated, and start and end age of estimation. Benign neoplasms are accounted for in the Level 3 GBD cause "other neoplasms" (ICD-9 codes 210–239, ICD-10 codes D00–D49), which is further subdivided into the Level 4 causes "myelodysplastic, myeloproliferative, and other haemopoietic neoplasms", "benign and in situ cervical and uterine neoplasms", "benign and in situ intestinal neoplasms", and "other benign and in situ neoplasms".

In this publication, estimates are presented for 47 GBD cancer groups, which include all GBD 2023 level 3 causes except non-melanoma skin cancer and "other neoplasms", and their respective underlying level 4 causes: squamous cell carcinoma (within the level 3 cause non-melanoma skin cancer); basal cell carcinoma (within the level 3 cause non-melanoma skin cancer); benign and in situ cervical and uterine neoplasms (within the level 3 cause other neoplasms); benign and in situ intestinal neoplasms (within the level 3 cause other neoplasms); other benign and in situ neoplasms (within the level 3 cause other neoplasms); and myelodysplastic, myeloproliferative, and other hematopoietic neoplasms (within the level 3 cause other neoplasms). Estimates are presented as totals combined across age groups, sexes, and locations for the time period from 1990 to 2023, with forecasts provided through 2050. More specific results are available via the GBD 2023 Results Tool, which provides estimates for every 5-year GBD age group (0–4; 5–9; etc. until 95+ years, plus more granular reporting in ages under 5 years), for every year from 1990 to 2023, for both sexes (males and females), and in 204 countries and territories. Additional forecast estimates for each year from 2024 to 2050 are available online in two places: general GBD 2021 forecast estimates can be accessed via the online Foresight Results Tool (https://vizhub.healthdata.org/gbd-foresight/#), while cancer-specific forecast estimates that have been adjusted to align with GBD 2023 are available from the Global Health Data Exchange *(To the editors and reviewers: note that cancer-specific forecast estimates have been submitted as csv files for review. A publicly accessbile GHDx link will be provided prior to publication).* All *International Classification of Disease* (ICD)[8] codes pertaining to cancer from ICD-9 (codes 140–209) and ICD-10 (codes C00–C97) are included in the estimates for "cancer" in this publication, except for Kaposi sarcoma (ICD-9: 176; ICD-10: C46). Kaposi sarcoma deaths are proportionally redistributed to either HIV/AIDS or to the GBD cause "soft tissue and other extraosseous sarcomas", with the redistribution proportion varying by GBD region (ranging from 7.7% to HIV/AIDS in the South Asia region to 93.0% HIV/AIDS in the Southern Sub-Saharan Africa region).[9] This region-based redistribution of Kaposi sarcoma was an update from GBD 2021 where the proportion of Kaposi sarcoma redistributed was based on age globally. The region-based redistribution for GBD 2023 was age agnostic and led to fewer C46 deaths being redistributed to HIV/AIDS globally than in previous GBD rounds.[10] All ICD-9 and ICD-10 codes pertaining to neoplasms (ICD-9: 140–239, ICD-10: C00–D49) are included in the estimates for "neoplasms" online, except for codes that are mapped to other causes in the GBD hierarchy, such as fibroids, which are estimated under the GBD cause "uterine fibroids". Because childhood cancers are often best defined by morphology rather than topography and some datasets are available using *International Classification of Childhood Cancer 3rd Edition* groupings (ICCC-3 diagnostic groups I–XII and related subgroups), ICCC-3 codes were also mapped to GBD causes.[11] Appendix 1 Table 2 and Appendix 1 Table 3 list all ICD-9, ICD-10, and ICCC-3 codes and their respective mapping to GBD causes.[11,12]

Of note, in this current iteration of GBD results reporting (GBD 2023), GBD Compare and the GBD Results Tools report incidence, prevalence, mortality, years lived with disability (YLDs), years of life lost (YLLs), and disability-adjusted life-years (DALYs) estimates for "neoplasms", which is an aggregate of

both level 3 malignant neoplasms and level 3 “other neoplasms”, which include benign neoplasms. For convenience to those primarily interested in estimates for malignant neoplasms (cancers), the GBD 2023 Results Tool also includes two custom Level 2 cancer groupings, “Total Cancers”, which provides estimates for the subset of the total neoplasms that exclude benign and in situ neoplasms but include non-melanoma skin cancers, and “Total Cancers excluding Non-melanoma skin cancer”, which provides estimates for the subset of total neoplasms that exclude both benign and in situ neoplasms and non-melanoma skin cancers. GBD Cancer Compare also presents visualizations for total cancer estimates (malignant neoplasms with and without non-melanoma skin cancers).

Appendix Table 1. GBD cancer cause characteristics in GBD 2023

GBD cause	Level	Sex estimated	Age start	Age end	Additional note
Neoplasms	2	Female, male	0-6 days	95+ years	
Lip and oral cavity cancer	3	Female, male	15-19 years	95+ years	
Nasopharynx cancer	3	Female, male	5-9 years	95+ years	
Other pharynx cancer	3	Female, male	20-24 years	95+ years	
Oesophageal cancer	3	Female, male	20-24 years	95+ years	
Stomach cancer	3	Female, male	15-19 years	95+ years	
Colon and rectum cancer	3	Female, male	15-19 years	95+ years	Includes additional estimation of individuals undergoing stoma, and prevalence beyond 10 years.
Liver cancer	3	Female, male	0-6 days	95+ years	
Liver cancer due to hepatitis B	*4*	*Female, male*	*10-14 years*	*95+ years*	*Estimates are proportionally split from the level 3 parent model.*
Liver cancer due to hepatitis C	*4*	*Female, male*	*10-14 years*	*95+ years*	*Estimates are proportionally split from the level 3 parent model.*
Liver cancer due to alcohol use	*4*	*Female, male*	*15-19 years*	*95+ years*	*Estimates are proportionally split from the level 3 parent model.*
Liver cancer due to NASH	*4*	*Female, male*	*15-19 years*	*95+ years*	*Estimates are proportionally split from the level 3 parent model.*
Hepatoblastoma	*4*	*Female, male*	*0-6 days*	*5-9 years*	*Estimates are the age <10 results from the level 3 parent model.*
Liver cancer due to other causes	*4*	*Female, male*	*10-14 years*	*95+ years*	*Estimates are proportionally split from the level 3 parent model.*
Gallbladder and biliary tract cancer	3	Female, male	20-24 years	95+ years	
Pancreatic cancer	3	Female, male	15-19 years	95+ years	
Larynx cancer	3	Female, male	20-24 years	95+ years	Includes additional estimation of individuals undergoing laryngectomy, and prevalence beyond 10 years.
Tracheal, bronchus, and lung cancer	3	Female, male	15-19 years	95+ years	
Malignant skin melanoma	3	Female, male	15-19 years	95+ years	
Non-melanoma skin cancer	3	Female, male	20-24 years	95+ years	Estimates are the aggregate of the underlying level 4 models.
Non-melanoma skin cancer (squamous-cell carcinoma)	*4*	*Female, male*	*20-24 years*	*95+ years*	*Non-fatal estimates are modeled via DisMod-MR and not from MIRs.*
Non-melanoma skin cancer (basal-cell carcinoma)	*4*	*Female, male*	*20-24 years*	*95+ years*	*No mortality estimated. Non-fatal estimates are modeled via DisMod-MR and not from MIRs.*
Soft tissue and other extraosseous sarcomas	3	Female, male	0-6 days	95+ years	
Malignant neoplasm of bone and articular cartilage	3	Female, male	12-23 months	95+ years	
Breast cancer	3	Female, male	15-19 years	95+ years	Includes additional estimation of individuals undergoing mastectomy, and prevalence beyond 10 years.
Cervical cancer	3	Female	15-19 years	95+ years	
Uterine cancer	3	Female	20-24 years	95+ years	
Ovarian cancer	3	Female	15-19 years	95+ years	
Prostate cancer	3	Male	20-24 years	95+ years	Includes additional estimation of individuals undergoing prostatectomy, and prevalence beyond 10 years.
Testicular cancer	3	Male	15-19 years	95+ years	
Kidney cancer	3	Female, male	0-6 days	95+ years	
Bladder cancer	3	Female, male	15-19 years	95+ years	Includes additional estimation of individuals undergoing cystectomy, and prevalence beyond 10 years.
Brain and central nervous system cancer	3	Female, male	0-6 days	95+ years	
Eye cancer	3	Female, male	0-6 days	95+ years	Estimates are the aggregate of the underlying level 4 models.

Retinoblastoma	*4*	*Female, male*	*0-6 days*	*5-9 years*	
Other eye cancers	*4*	*Female, male*	*10-14 years*	*95+ years*	
Neuroblastoma and other peripheral nervous cell tumors	3	Female, male	0-6 days	95+ years	
Thyroid cancer	3	Female, male	5-9 years	95+ years	
Mesothelioma	3	Female, male	20-24 years	95+ years	
Hodgkin lymphoma	3	Female, male	2-4 years	95+ years	
Non-Hodgkin lymphoma	3	Female, male	12-23 months	95+ years	
Burkitt lymphoma	*4*	*Female, male*	*12-23 months*	*95+ years*	
Other non-Hodgkin lymphoma	*4*	*Female, male*	*12-23 months*	*95+ years*	
Multiple myeloma	3	Female, male	20-24 years	95+ years	
Leukemia	3	Female, male	0-6 days	95+ years	
Acute lymphoid leukemia	*4*	*Female, male*	*0-6 days*	*95+ years*	
Chronic lymphoid leukemia	*4*	*Female, male*	*20-24 years*	*95+ years*	
Acute myeloid leukemia	*4*	*Female, male*	*0-6 days*	*95+ years*	
Chronic myeloid leukemia	*4*	*Female, male*	*0-6 days*	*95+ years*	
Other leukemia	*4*	*Female, male*	*0-6 days*	*95+ years*	
Other malignant neoplasms	3	Female, male	0-6 days	95+ years	
Other neoplasms	3	Female, male	0-6 days	95+ years	
Myelodysplastic, myeloproliferative, and other hematopoietic neoplasms	*4*	*Female, male*	*0-6 days*	*95+ years*	*Non-fatal estimates are modeled via DisMod-MR and not from MIRs.*
Benign and in situ intestinal neoplasms	*4*	*Female, male*	*6-11 months*	*95+ years*	*No mortality estimated. Non-fatal estimates are modeled via DisMod-MR and not from MIRs.*
Benign and in situ cervical and uterine neoplasms	*4*	*Female*	*6-11 months*	*95+ years*	*No mortality estimated. Non-fatal estimates are modeled via DisMod-MR and not from MIRs.*
Other benign and in situ neoplasms	*4*	*Female, male*	*1-5 months*	*95+ years*	*Non-fatal estimates are modeled via DisMod-MR and not from MIRs.*

Abbreviations: DisMod-MR, disease model - Bayesian meta-regression; GBD, Global Burden of Diseases, Injuries, and Risk Factors Study 2023; MIR, mortality-to-incidence ratio. Causes are listed in GBD hierarchy order. Level 4 causes are italicized and listed under their Level 3 parent cause. Comments in the “Notes” column are explained further in the methods below.

Appendix Table 2: List of ICD codes mapped to GBD cause list for cancer incidence data

GBD cause	ICCC-3	ICD-10	ICD-9
Lip and oral cavity cancer	XIf1	C0, C00, C00.0, C00.1, C00.2, C00.3, C00.4, C00.5, C00.6, C00.8, C00.9, C01, C01.9, C02, C02.0, C02.1, C02.2, C02.3, C02.4, C02.8, C02.9, C03, C03.0, C03.1, C03.9, C04, C04.0, C04.1, C04.8, C04.9, C05, C05.0, C05.1, C05.2, C05.8, C05.9, C06, C06.0, C06.1, C06.2, C06.8, C06.80, C06.89, C06.9, C07, C07.0, C07.9, C08, C08.0, C08.1, C08.8, C08.9	140, 140.0, 140.1, 140.2, 140.3, 140.4, 140.5, 140.6, 140.7, 140.8, 140.9, 141, 141.0, 141.1, 141.2, 141.3, 141.4, 141.5, 141.6, 141.8, 141.9, 142, 142.0, 142.1, 142.2, 142.3, 142.8, 142.9, 143, 143.0, 143.1, 143.8, 143.9, 144, 144.0, 144.1, 144.4, 144.8, 144.9, 145, 145.0, 145.1, 145.2, 145.3, 145.4, 145.5, 145.6, 145.8, 145.9
Nasopharynx cancer	XIc	C11, C11.0, C11.1, C11.2, C11.3, C11.8, C11.9	147, 147.0, 147.1, 147.2, 147.3, 147.8, 147.9
Other pharynx cancer	NA	C09, C09.0, C09.1, C09.8, C09.9, C1, C10, C10.0, C10.1, C10.2, C10.3, C10.4, C10.8, C10.9, C12, C12.0, C12.9, C13, C13.0, C13.1, C13.2, C13.8, C13.9	146, 146.0, 146.1, 146.2, 146.3, 146.4, 146.5, 146.6, 146.7, 146.8, 146.9, 148, 148.0, 148.1, 148.2, 148.3, 148.4, 148.5, 148.8, 148.9
Oesophageal cancer	NA	C15, C15.0, C15.1, C15.2, C15.3, C15.4, C15.5, C15.8, C15.9	150, 150.0, 150.1, 150.2, 150.3, 150.4, 150.5, 150.6, 150.7, 150.8, 150.9
Stomach cancer	NA	C16, C16.0, C16.1, C16.2, C16.3, C16.4, C16.5, C16.6, C16.7, C16.8, C16.9	151, 151.0, 151.1, 151.2, 151.3, 151.4, 151.5, 151.6, 151.8, 151.9, 209.23
Colon and rectum cancer	XIf2, XIf3	C18, C18.0, C18.1, C18.2, C18.3, C18.4, C18.5, C18.6, C18.7, C18.8, C18.9, C19, C19.0, C19.9, C2, C20, C20.0, C20.8, C20.9, C21, C21.0, C21.1, C21.2, C21.8, C21.9	153, 153.0, 153.1, 153.2, 153.3, 153.4, 153.5, 153.6, 153.7, 153.8, 153.9, 154, 154.0, 154.1, 154.2, 154.3, 154.4, 154.8, 154.9, 209.1, 209.10, 209.11, 209.12, 209.13, 209.14, 209.15, 209.16, 209.17, 569.0, 569.43, 569.44, 569.84, 569.85
Liver cancer	VIIa, VIIb, VIIc	C22, C22.0, C22.1, C22.2, C22.3, C22.4, C22.5, C22.7, C22.8	155, 155.0, 155.1, 155.3, 155.5, 155.9
*Hepatoblastoma**	*See footnote*	*See footnote*	*See footnote*
Gallbladder and biliary tract cancer	NA	C23, C23.0, C23.9, C24, C24.0, C24.1, C24.4, C24.8, C24.9	156, 156.0, 156.1, 156.2, 156.3, 156.8, 156.9
Pancreatic cancer	XIIa2	C25, C25.0, C25.1, C25.2, C25.3, C25.4, C25.7, C25.8, C25.9	157, 157.0, 157.1, 157.2, 157.3, 157.4, 157.5, 157.7, 157.8, 157.9
Larynx cancer	NA	C32, C32.0, C32.1, C32.2, C32.3, C32.8, C32.9	161, 161.0, 161.1, 161.2, 161.3, 161.8, 161.9
Tracheal, bronchus, and lung cancer	XIIa3, XIf4	C33, C33.0, C33.2, C33.9, C34, C34.0, C34.00, C34.01, C34.02, C34.1, C34.10, C34.11, C34.12, C34.2, C34.3, C34.30, C34.31, C34.32, C34.4, C34.7, C34.8, C34.80, C34.81, C34.82, C34.9, C34.90, C34.91, C34.92	162, 162.0, 162.1, 162.2, 162.3, 162.4, 162.5, 162.8, 162.9, 209.21
Malignant neoplasm of bone and articular cartilage	VIII, VIIIa, VIIIb, VIIIc, VIIIc1, VIIIc2, VIIId, VIIId1, VIIId2, VIIId3, VIIId4, VIIIe	C40, C40.0, C40.00, C40.01, C40.02, C40.1, C40.10, C40.11, C40.12, C40.2, C40.20, C40.21, C40.22, C40.3, C40.30, C40.31, C40.32, C40.8, C40.80, C40.81, C40.82, C40.9, C40.90, C40.91, C40.92, C41, C41.0, C41.1, C41.2, C41.3, C41.4, C41.5, C41.6, C41.7, C41.8, C41.9	170, 170.0, 170.1, 170.2, 170.3, 170.4, 170.5, 170.6, 170.7, 170.8, 170.9
Malignant skin melanoma	XId	C43, C43.0, C43.1, C43.10, C43.11, C43.12, C43.2, C43.20, C43.21, C43.22, C43.3, C43.30, C43.31, C43.39, C43.4, C43.5, C43.51, C43.52, C43.59, C43.6, C43.60, C43.61, C43.62, C43.7, C43.70, C43.71, C43.72, C43.8, C43.9	172, 172.0, 172.1, 172.2, 172.3, 172.4, 172.5, 172.6, 172.7, 172.8, 172.9
Non-melanoma skin cancer	XIe	C44, C44.0, C44.00, C44.01, C44.02 C44.09, C44.1, C44.10, C44.101, C44.102, C44.109, C44.11, C44.111, C44.112, C44.119, C44.112, C44.119, C44.12, C44.121, C44.122, C44.129, C44.19, C44.191, C44.192, C44.199, C44.2, C44.20, C44.201, C44.202, C44.209, C44.21, C44.211, C44.212, C44.222, C44.229, C44.29, C44.291, C44.292, C44.299, C44.3, C44.30, C44.300, C44.301, C44.309, C44.31, C44.310, C44.311, C44.319, C44.32, C44.321, C44.329, C44.39, C44.390, C44.391, C44.399, C44.4, C44.40, C44.41, C44.42, C44.49, C44.5, C44.50, C44.500, C44.501, C44.509, C44.51, C44.510, C44.511, C44.519, C44.52, C44.520, C44.51, C44.529, C44.59, C44.590, C44.591, C44.599, C44.6, C44.60, C44.601, C44.602, C44.609, C44.61, C44.611, C44.612, C44.619, C44.62, C44.621, C44.622, C44.629, C44.69, C44.691, C44.692, C44.699, C44.7, C44.70, C44.701, C44.702, C44.709, C44.71, C44.711, C44.712, C44.719, C44.72, C44.721, C44.722, C44.729, C44.79, C44.791, C44.792, C44.799, C44.8, C44.80, C44.81, C44.82, C44.89, C44.9, C44.90, C44.91, C44.92, C44.99	173, 173.0, 173.00, 173.01, 173.02, 173.09, 173.1, 173.10, 173.11, 173.12, 173.19, 173.2, 173.20, 173.21, 173.22, 173.29, 173.3, 173.30, 173.31, 173.32, 173.39, 173.4, 173.40, 173.41, 173.42, 173.49, 173.5, 173.50, 173.51, 173.52, 173.59, 173.6, 173.60, 173.61, 173.62, 173.69, 173.7, 173.70, 173.71, 173.72, 173.79, 173.8, 173.80, 173.81, 173.82, 173.89, 173.9, 173.90, 173.91, 173.92, 173.99
Mesothelioma	XIIa5	C45, C45.0, C45.1, C45.2, C45.3, C45.4, C45.5, C45.6, C45.7, C45.8, C45.9	NA

GBD cause	ICCC-3	ICD-10	ICD-9
Neuroblastoma and other peripheral nervous cell tumours	IVa, IVb	C47, C47.0, C47.1, C47.10, C47.11, C47.12, C47.2, C47.20, C47.21, C47.22, C47.3, C47.4, C47.5, C47.6, C47.8, C47.9, C74.90	NA
Soft tissue and other extraosseous sarcomas	IX, IXa, IXb, IXb1, IXb2, IXb3, IXd, IXd1, IXd10, IXd11, IXd2, IXd3, IXd4, IXd5, IXd6, IXd7, IXd8, IXd9, IXe	C49, C49.0, C49.1, C49.10, C49.11, C49.12, C49.2, C49.20, C49.21, C49.22, C49.3, C49.4, C49.5, C49.6, C49.8, C49.9	171, 171.0, 171.2, 171.3, 171.4, 171.5, 171.6, 171.7, 171.8, 171.9
Breast cancer	XIf6	C50, C50.0, C50.01, C50.011, C50.012, C50.019, C50.02, C50.021, C50.022, C50.029, C50.1, C50.11, C50.111, C50.112, C50.119, C50.12, C50.121, C50.122, C50.129, C50.2, C50.21, C50.211, C50.212, C50.219, C50.22, C50.221, C50.222, C50.229, C50.3, C50.31, C50.311, C50.312, C50.319, C50.32, C50.321, C50.322, C50.329, C50.4, C50.41, C50.411, C50.412, C50.419, C50.42, C50.421, C50.422, C50.429, C50.5, C50.51, C50.511, C50.512, C50.519, C50.52, C50.521, C50.522, C50.529, C50.6, C50.61, C50.611, C50.612, C50.619, C50.62, C50.621, C50.622, C50.629, C50.7, C50.8, C50.81, C50.811, C50.812, C50.819, C50.82, C50.821, C50.822, C50.829, C50.9, C50.91, C50.911, C50.912, C50.919, C50.92, C50.921, C50.922, C50.929	174, 174.0, 174.1, 174.2, 174.3, 174.4, 174.5, 174.6, 174.8, 174.9, 175, 175.0, 175.3, 175.9
Cervical cancer	XIf7	C53, C53.0, C53.1, C53.3, C53.4, C53.8, C53.9	180, 180.0, 180.1, 180.2, 180.3, 180.4, 180.5, 180.6, 180.8, 180.9
Uterine cancer	NA	C54, C54.0, C54.1, C54.2, C54.3, C54.4, C54.8, C54.9	182, 182.0, 182.1, 182.8, 182.9
Ovarian cancer	Xc, Xd, Xe	C56, C56.0, C56.1, C56.2, C56.4, C56.9	183, 183.0
Prostate cancer	NA	C61, C61.0, C61.9	185, 185.0, 185.9
Testicular cancer	Xc, Xd, Xe	C62, C62.0, C62.00, C62.01, C62.02, C62.1, C62.10, C62.11, C62.12, C62.9, C62.90, C62.91, C62.92	186, 186.0, 186.9
Kidney cancer	VI, VIa, VIa1, VIa2, VIa3, VIa4, VIb, VIc	C64, C64.0, C64.1, C64.2, C64.4, C64.5, C64.6, C64.8, C64.9, C65, C65.0, C65.1, C65.2, C65.9	189.0, 189.1, 189.5, 189.6, 209.24
Bladder cancer	XIf8	C67, C67.0, C67.1, C67.2, C67.3, C67.4, C67.5, C67.6, C67.7, C67.8, C67.9	188, 188.0, 188.1, 188.2, 188.3, 188.4, 188.5, 188.6, 188.7, 188.8, 188.9
Eye cancer	V, XIf9	C69.0, C69.00, C69.01, C69.02, C69.1, C69.10, C69.11, C69.12, C69.2, C69.20, C69.21, C69.22, C69.3, C69.30, C69.31, C69.32, C69.4, C69.40, C69.41, C69.42, C69.5, C69.50, C69.51, C69.52, C69.6, C69.60, C69.61, C69.62, C69.7, C69.8, C69.80, C69.81, C69.82	190, 190.0, 190.1, 190.2, 190.3, 190.4, 190.5, 190.6, 190.7, 190.8
Retinoblastoma	*V*	*C69.2, C69.20, C69.21, C69.22*	*190.5*
Other eye cancers	*XIf9*	*C69.0, C69.00, C69.01, C69.02, C69.1, C69.10, C69.11, C69.12, C69.3, C69.30, C69.31, C69.32, C69.4, C69.40, C69.41, C69.42, C69.5, C69.50, C69.51, C69.52, C69.6, C69.60, C69.61, C69.62, C69.7, C69.8, C69.80, C69.81, C69.82*	*190, 190.0, 190.1, 190.2, 190.3, 190.4, 190.6, 190.7, 190.8*
Brain and central nervous system cancer	III, IIIa, IIIa1, IIIa2, IIIb, IIIc, IIIc1, IIIc2, IIIc3, IIIc4, IIId, IIId1, IIId2, IIId3, IIIe, IIIe1, IIIe2, IIIe3, IIIe4, IIIe5, IIIf, Xa, Xa1, Xa2, Xa3, Xa4, Xa5, Xa6	C70, C70.0, C70.1, C70.5, C70.6, C70.9, C71, C71.0, C71.1, C71.2, C71.3, C71.4, C71.5, C71.6, C71.7, C71.8, C71.9, C72, C72.0, C72.1, C72.2, C72.20, C72.21, C72.22, C72.3, C72.30, C72.31, C72.32, C72.4, C72.40, C72.41, C72.42, C72.5, C72.50, C72.59, C72.8, C72.9, C75.1, C75.2, C75.3	191, 191.0, 191.1, 191.2, 191.3, 191.4, 191.5, 191.6, 191.7, 191.8, 191.9, 192, 192.0, 192.1, 192.2, 192.3, 192.4, 192.8, 192.9
Thyroid cancer	XIb	C73, C73.0, C73.1, C73.2, C73.3, C73.4, C73.5, C73.8, C73.9	193, 193.0, 193.2, 193.9
Other malignant neoplasms	XIIa1, XIIa4, XIIa6, XIIb, Xia, Xif10, Xif11, Xif5, Xb, Xb1, Xb2, Xb3, Xb4, Xb5, Xb6	C17, C17.0, C17.1, C17.2, C17.3, C17.8, C17.9, C3, C30, C30.0, C30.1, C30.2, C30.3, C30.5, C30.8, C30.9, C31, C31.0, C31.1, C31.2, C31.3, C31.8, C31.9, C37, C37.0, C37.1, C37.2, C37.3, C38, C38.0, C38.1, C38.2, C38.3, C38.4, C38.8, C4, C48, C48.0, C48.1, C48.2, C48.8, C48.9, C4A, C5, C51, C51.0, C51.1, C51.2, C51.8, C51.9, C52, C52.0, C52.9, C57, C57.0, C57.00, C57.01, C57.02, C57.1, C57.10, C57.11, C57.12, C57.2, C57.20, C57.21, C57.22, C57.3, C57.4, C57.7, C57.8, C60, C60.0, C60.1, C60.2, C60.8, C60.9, C63, C63.0, C63.00,	152, 152.0, 152.1, 152.2, 152.3, 152.4, 152.6, 152.8, 152.9, 158, 158.0, 158.3, 158.4, 158.5, 158.6, 158.8, 158.9, 160, 160.0, 160.1, 160.2, 160.3, 160.4, 160.5, 160.6, 160.8, 160.9, 163, 163.0, 163.1, 163.3, 163.5, 163.8, 163.9, 164, 164.0, 164.1, 164.2, 164.3, 164.8, 164.9, 183.2, 183.3, 183.4, 183.5, 183.8, 184.0, 184.1, 184.2, 184.3, 184.4, 184.8, 187.1, 187.2,

GBD cause	ICCC-3	ICD-10	ICD-9
		C63.01, C63.02, C63.1, C63.10, C63.11, C63.12, C63.2, C63.7, C63.8, C66, C66.0, C66.1, C66.2, C66.9, C68.0, C68.1, C68.8, C7, C75, C75.0, C75.4, C75.5, C75.6, C75.8	187.3, 187.4, 187.5, 187.6, 187.7, 187.8, 189.2, 189.3, 189.4, 189.8, 194.1, 194.5, 194.6, 194.8, 209.0, 209.00, 209.01, 209.02, 209.03, 209.22, 209.25, 209.26, 209.27, 209.31, 209.32, 209.33, 209.34, 209.35, 209.36
Hodgkin lymphoma	Iia	C81, C81.0, C81.00, C81.01, C81.02, C81.03, C81.04, C81.05, C81.06, C81.07, C81.08, C81.09, C81.1, C81.10, C81.11, C81.12, C81.13, C81.14, C81.15, C81.16, C81.17, C81.18, C81.19, C81.2, C81.20, C81.21, C81.22, C81.23, C81.24, C81.25, C81.26, C81.27, C81.28, C81.29, C81.3, C81.30, C81.31, C81.32, C81.33, C81.34, C81.35, C81.36, C81.37, C81.38, C81.39, C81.4, C81.40, C81.41, C81.42, C81.43, C81.44, C81.45, C81.46, C81.47, C81.48, C81.49, C81.5, C81.6, C81.7, C81.70, C81.71, C81.72, C81.73, C81.74, C81.75, C81.76, C81.77, C81.78, C81.79, C81.8, C81.9, C81.90, C81.91, C81.92, C81.93, C81.94, C81.95, C81.96, C81.97, C81.98, C81.99	201, 201.0, 201.00, 201.01, 201.02, 201.03, 201.04, 201.05, 201.06, 201.07, 201.08, 201.1, 201.10, 201.11, 201.12, 201.13, 201.14, 201.15, 201.16, 201.17, 201.18, 201.2, 201.20, 201.21, 201.22, 201.23, 201.24, 201.25, 201.26, 201.27, 201.28, 201.4, 201.40, 201.41, 201.42, 201.43, 201.44, 201.45, 201.46, 201.47, 201.48, 201.5, 201.50, 201.51, 201.52, 201.53, 201.54, 201.55, 201.56, 201.57, 201.58, 201.6, 201.60, 201.61, 201.62, 201.63, 201.64, 201.65, 201.66, 201.67, 201.68, 201.7, 201.70, 201.71, 201.72, 201.73, 201.74, 201.75, 201.76, 201.77, 201.78, 201.9, 201.90, 201.91, 201.92, 201.93, 201.94, 201.95, 201.96, 201.97, 201.98
Non-Hodgkin lymphoma	Iib, Iib1, Iib2, Iib3, Iib4, Iic, Iid, Iie	C82, C82.0, C82.00, C82.01, C82.02, C82.03, C82.04, C82.05, C82.06, C82.07, C82.08, C82.09, C82.1, C82.10, C82.11, C82.12, C82.13, C82.14, C82.15, C82.16, C82.17, C82.18, C82.19, C82.2, C82.20, C82.21, C82.22, C82.23, C82.24, C82.25, C82.26, C82.27, C82.28, C82.29, C82.3, C82.30, C82.31, C82.32, C82.33, C82.34, C82.35, C82.36, C82.37, C82.38, C82.39, C82.4, C82.40, C82.41, C82.42, C82.43, C82.44, C82.45, C82.46, C82.47, C82.48, C82.49, C82.5, C82.50, C82.51, C82.52, C82.53, C82.54, C82.55, C82.56, C82.57, C82.58, C82.59, C82.6, C82.60, C82.61, C82.62, C82.63, C82.64, C82.65, C82.66, C82.67, C82.68, C82.69, C82.7, C82.8, C82.80, C82.81, C82.82, C82.83, C82.84, C82.85, C82.86, C82.87, C82.88, C82.89, C82.9, C82.90, C82.91, C82.92, C82.93, C82.94, C82.95, C82.96, C82.97, C82.98, C82.99, C83.0, C83.00, C83.01, C83.02, C83.03, C83.04, C83.05, C83.06, C83.07, C83.08, C83.09, C83.1, C83.10, C83.11, C83.12, C83.13, C83.14, C83.15, C83.16, C83.17, C83.18, C83.19, C83.2, C83.3, C83.30, C83.31, C83.32, C83.33, C83.34, C83.35, C83.36, C83.37, C83.38, C83.39, C83.4, C83.5, C83.50, C83.51, C83.52, C83.53, C83.54, C83.55, C83.56, C83.57, C83.58, C83.59, C83.6, C83.7, C83.70, C83.71, C83.72, C83.73, C83.74, C83.75, C83.76, C83.77, C83.78, C83.79, C83.8, C83.80, C83.81, C83.82, C83.83, C83.84, C83.85, C83.86, C83.87, C83.88, C83.89, C84, C84.0, C84.00, C84.01, C84.02, C84.03, C84.04, C84.05, C84.06, C84.07, C84.08, C84.09, C84.1, C84.10, C84.11, C84.12, C84.13, C84.14, C84.15, C84.16, C84.17, C84.18, C84.19, C84.2, C84.3, C84.4, C84.40, C84.41, C84.42, C84.43, C84.44, C84.45, C84.46, C84.47, C84.48, C84.49, C84.5, C84.6, C84.60, C84.61, C84.62, C84.63, C84.64, C84.65, C84.66, C84.67, C84.68, C84.69, C84.7, C84.70, C84.71, C84.72, C84.73, C84.74, C84.75, C84.76, C84.77, C84.78, C84.79, C84.8, C84.9, C84.90, C84.91, C84.92, C84.93, C84.94, C84.95, C84.96, C84.97, C84.98, C84.99, C85, C85.0, C85.1, C85.10, C85.11, C85.12, C85.13, C85.14, C85.15, C85.16, C85.17, C85.18, C85.19, C85.2, C85.20, C85.21, C85.22, C85.23, C85.24, C85.25, C85.26, C85.27, C85.28, C85.29, C85.3, C85.4, C85.5, C85.6, C85.7, C85.8, C85.80, C85.81, C85.82, C85.83, C85.84, C85.85, C85.86, C85.87, C85.88, C85.89, C86, C86.0, C86.1, C86.2, C86.3, C86.4, C86.5, C86.6, C96, C96.0, C96.1, C96.2, C96.3, C96.4, C96.5, C96.6, C96.7, C96.8, C96.9	200, 200.0, 200.00, 200.01, 200.02, 200.03, 200.04, 200.05, 200.06, 200.07, 200.08, 200.1, 200.10, 200.11, 200.12, 200.13, 200.14, 200.15, 200.16, 200.17, 200.18, 200.2, 200.20, 200.21, 200.22, 200.23, 200.24, 200.25, 200.26, 200.27, 200.28, 200.3, 200.30, 200.31, 200.32, 200.33, 200.34, 200.35, 200.36, 200.37, 200.38, 200.4, 200.40, 200.41, 200.42, 200.43, 200.44, 200.45, 200.46, 200.47, 200.48, 200.5, 200.50, 200.51, 200.52, 200.53, 200.54, 200.55, 200.56, 200.57, 200.58, 200.6, 200.60, 200.61, 200.62, 200.63, 200.64, 200.65, 200.66, 200.67, 200.68, 200.7, 200.70, 200.71, 200.72, 200.73, 200.74, 200.75, 200.76, 200.77, 200.78, 200.8, 200.80, 200.81, 200.82, 200.83, 200.84, 200.85, 200.86, 200.87, 200.88, 200.9, 202, 202.0, 202.00, 202.01, 202.02, 202.03, 202.04, 202.05, 202.06, 202.07, 202.08, 202.1, 202.10, 202.11, 202.12, 202.13, 202.14, 202.15, 202.16, 202.17, 202.18, 202.2, 202.20, 202.21, 202.22, 202.23, 202.24, 202.25, 202.26, 202.27, 202.28, 202.3, 202.30, 202.31, 202.32, 202.33, 202.34, 202.35, 202.36, 202.37, 202.38, 202.4, 202.40, 202.41, 202.42, 202.43, 202.44, 202.45, 202.46, 202.47, 202.48, 202.5, 202.50, 202.51, 202.52, 202.53, 202.54, 202.55, 202.56, 202.57, 202.58, 202.6, 202.60, 202.61, 202.62, 202.63, 202.64, 202.65, 202.66, 202.67, 202.68, 202.7, 202.70, 202.71, 202.72, 202.73, 202.74, 202.75, 202.76, 202.77, 202.78, 202.8, 202.80, 202.81, 202.82, 202.83, 202.84, 202.85, 202.86, 202.87, 202.88, 202.9, 202.90, 202.91, 202.92, 202.93, 202.94, 202.95, 202.96, 202.97, 202.98
Burkitt lymphoma	*Iic*	*C83.7, C83.70, C83.71, C83.72, C83.73, C83.74, C83.75, C83.76, C83.77, C83.78, C83.79*	*200.2, 200.20, 200.21, 200.22, 200.23, 200.24, 200.25, 200.26, 200.27, 200.28*
Other non-Hodgkin lymphoma	*Iib, Iib1, Iib2, Iib3, Iib4, Iid, Iie*	*C82, C82.0, C82.00, C82.01, C82.02, C82.03, C82.04, C82.05, C82.06, C82.07, C82.08, C82.09, C82.1, C82.10, C82.11, C82.12, C82.13, C82.14, C82.15, C82.16, C82.17, C82.18, C82.19, C82.2, C82.20, C82.21, C82.22, C82.23, C82.24, C82.25, C82.26, C82.27, C82.28, C82.29, C82.3, C82.30, C82.31, C82.32, C82.33, C82.34, C82.35, C82.36, C82.37, C82.38, C82.39, C82.4, C82.40, C82.41, C82.42, C82.43, C82.44, C82.45, C82.46, C82.47, C82.48, C82.49, C82.5, C82.50, C82.51, C82.52, C82.53, C82.54, C82.55, C82.56, C82.57, C82.58,*	*200, 200.0, 200.00, 200.01, 200.02, 200.03, 200.04, 200.05, 200.06, 200.07, 200.08, 200.1, 200.10, 200.11, 200.12, 200.13, 200.14, 200.15, 200.16, 200.17, 200.18, 200.3, 200.30, 200.31, 200.32, 200.33, 200.34, 200.35, 200.36, 200.37, 200.38, 200.4, 200.40, 200.41, 200.42, 200.43, 200.44, 200.45, 200.46, 200.47, 200.48,*

GBD cause	ICCC-3	ICD-10	ICD-9
		C82.59, C82.6, C82.60, C82.61, C82.62, C82.63, C82.64, C82.65, C82.66, C82.67, C82.68, C82.69, C82.7, C82.8, C82.80, C82.81, C82.82, C82.83, C82.84, C82.85, C82.86, C82.87, C82.88, C82.89, C82.9, C82.90, C82.91, C82.92, C82.93, C82.94, C82.95, C82.96, C82.97, C82.98, C82.99, C83.0, C83.00, C83.01, C83.02, C83.03, C83.04, C83.05, C83.06, C83.07, C83.08, C83.09, C83.1, C83.10, C83.11, C83.12, C83.13, C83.14, C83.15, C83.16, C83.17, C83.18, C83.19, C83.2, C83.3, C83.30, C83.31, C83.32, C83.33, C83.34, C83.35, C83.36, C83.37, C83.38, C83.39, C83.4, C83.5, C83.50, C83.51, C83.52, C83.53, C83.54, C83.55, C83.56, C83.57, C83.58, C83.59, C83.6, C83.8, C83.80, C83.81, C83.82, C83.83, C83.84, C83.85, C83.86, C83.87, C83.88, C83.89, C84, C84.0, C84.00, C84.01, C84.02, C84.03, C84.04, C84.05, C84.06, C84.07, C84.08, C84.09, C84.1, C84.10, C84.11, C84.12, C84.13, C84.14, C84.15, C84.16, C84.17, C84.18, C84.19, C84.2, C84.3, C84.4, C84.40, C84.41, C84.42, C84.43, C84.44, C84.45, C84.46, C84.47, C84.48, C84.49, C84.5, C84.6, C84.60, C84.61, C84.62, C84.63, C84.64, C84.65, C84.66, C84.67, C84.68, C84.69, C84.7, C84.70, C84.71, C84.72, C84.73, C84.74, C84.75, C84.76, C84.77, C84.78, C84.79, C84.8, C84.9, C84.90, C84.91, C84.92, C84.93, C84.94, C84.95, C84.96, C84.97, C84.98, C84.99, C85, C85.0, C85.1, C85.10, C85.11, C85.12, C85.13, C85.14, C85.15, C85.16, C85.17, C85.18, C85.19, C85.2, C85.20, C85.21, C85.22, C85.23, C85.24, C85.25, C85.26, C85.27, C85.28, C85.29, C85.3, C85.4, C85.5, C85.6, C85.7, C85.8, C85.80, C85.81, C85.82, C85.83, C85.84, C85.85, C85.86, C85.87, C85.88, C85.89, C86, C86.0, C86.1, C86.2, C86.3, C86.4, C86.5, C86.6, C96, C96.0, C96.1, C96.2, C96.3, C96.4, C96.5, C96.6, C96.7, C96.8, C96.9	*200.5, 200.50, 200.51, 200.52, 200.53, 200.54, 200.55, 200.56, 200.57, 200.58, 200.6, 200.60, 200.61, 200.62, 200.63, 200.64, 200.65, 200.66, 200.67, 200.68, 200.7, 200.70, 200.71, 200.72, 200.73, 200.74, 200.75, 200.76, 200.77, 200.78, 200.8, 200.80, 200.81, 200.82, 200.83, 200.84, 200.85, 200.86, 200.87, 200.88, 200.9, 202, 202.0, 202.00, 202.01, 202.02, 202.03, 202.04, 202.05, 202.06, 202.07, 202.08, 202.1, 202.10, 202.11, 202.12, 202.13, 202.14, 202.15, 202.16, 202.17, 202.18, 202.2, 202.20, 202.21, 202.22, 202.23, 202.24, 202.25, 202.26, 202.27, 202.28, 202.3, 202.30, 202.31, 202.32, 202.33, 202.34, 202.35, 202.36, 202.37, 202.38, 202.4, 202.40, 202.41, 202.42, 202.43, 202.44, 202.45, 202.46, 202.47, 202.48, 202.5, 202.50, 202.51, 202.52, 202.53, 202.54, 202.55, 202.56, 202.57, 202.58, 202.6, 202.60, 202.61, 202.62, 202.63, 202.64, 202.65, 202.66, 202.67, 202.68, 202.7, 202.70, 202.71, 202.72, 202.73, 202.74, 202.75, 202.76, 202.77, 202.78, 202.8, 202.80, 202.81, 202.82, 202.83, 202.84, 202.85, 202.86, 202.87, 202.88, 202.9, 202.90, 202.91, 202.92, 202.93, 202.94, 202.95, 202.96, 202.97, 202.98*
Multiple myeloma	NA	C88, C88.0, C88.1, C88.2, C88.20, C88.3, C88.4, C88.40, C88.7, C88.70, C88.71, C88.8, C88.9, C89, C90, C90.0, C90.00, C90.01, C90.02, C90.1, C90.10, C90.11, C90.12, C90.2, C90.20, C90.21, C90.22, C90.3, C90.30, C90.31, C90.32, C90.4, C90.5, C90.6, C90.7, C90.8, C90.9	203, 203.0, 203.00, 203.01, 203.02, 203.1, 203.10, 203.11, 203.12, 203.8, 203.80, 203.81, 203.82, 203.9
Leukaemia	Ia, Ia1, Ia2, Ia3, Ia4, Ib, Ic, Ie	C91, C92, C93, C94, C95, C95.0, C95.00, C95.01, C95.02, C95.1, C95.10, C95.11, C95.12, C95.2, C95.4, C95.6, C95.7, C95.9, C95.90, C95.91, C95.92	204.0, 204.00, 204.01, 204.02, 204.2, 204.20, 204.21, 204.22, 205.0, 205.00, 205.01, 205.02, 205.1, 205.10, 205.11, 205.12, 205.2, 205.20, 205.21, 205.22, 205.3, 205.30, 205.31, 205.32, 205.92, 206.0, 206.00, 206.01, 206.02, 206.1, 206.10, 206.11, 206.12, 207.0, 207.00, 207.01, 207.02, 207.1, 207.10, 207.11, 207.12, 207.2, 207.21, 207.22, 207.8, 207.80, 207.81, 207.82, 207.9
*Acute lymphoid leukaemia**	*Ia, Ia1, Ia2, Ia3, Ia4*	*C91.0, C91.00, C91.01, C91.02, C91.2, C91.3, C91.30, C91.31, C91.32, C91.6, C91.60, C91.61, C91.62, custom mapping (see footnote)*	*204.0, 204.00, 204.01, 204.02, 204.2, 204.20, 204.21, 204.22, custom mapping (see footnote)*
*Chronic lymphoid leukaemia**	*NA*	*Custom mapping (see footnote)*	*Custom mapping (see footnote)*
Acute myeloid leukaemia	*Ib*	*C92.0, C92.00, C92.01, C92.02, C92.3, C92.30, C92.31, C92.32, C92.4, C92.40, C92.41, C92.42, C92.5, C92.50, C92.51, C92.52, C92.6, C92.60, C92.61, C92.62, C93.0, C93.00, C93.01, C93.02, C94.0, C94.00, C94.01, C94.02, C94.2, C94.20, C94.21, C94.22, C94.4, C94.40, C94.41, C94.42, C94.5*	*205.0, 205.00, 205.01, 205.02, 205.2, 205.20, 205.21, 205.22, 205.3, 205.30, 205.31, 205.32, 206.0, 206.00, 206.01, 206.02, 207.0, 207.00, 207.01, 207.02, 207.2, 207.20, 207.21, 207.22, 207.8, 207.80, 207.81, 207.82*
Chronic myeloid leukaemia	*Ic*	*C92.1, C92.10, C92.11, C92.12, C92.2, C92.20, C92.21, C92.22*	*205.1, 205.10, 205.11, 205.12*
Other leukaemia	*Ie*	*C93.1, C93.10, C93.11, C93.12, C93.3, C93.30, C93.31, C93.32, C93.8, C94.1, C94.3, C94.30, C94.31, C94.32, C94.7, C94.8, C94.80, C94.81, C94.82*	*205.92, 206.1, 206.10, 206.11, 206.12, 207, 207.1, 207.10, 207.11, 207.12, 207.9*

Abbreviations: ICCC-3, International Classification of Childhood Cancer, Third Edition; ICD-9, International Classification of Diseases, Ninth Revision; ICD-10, International Classification of Diseases, Tenth Revision; NA, not applicable (i.e., no relevant codes mapped for that coding system). Italicised rows indicate Level 4 GBD causes within the Level 3 cause directly above (not italicized). For example, "*Burkitt lymphoma*" and "*Other non-Hodgkin lymphoma*" are Level 4 causes within the Level 3 cause "Non-Hodgkin lymphoma". *Hepatoblastoma codes (ICCC-3: VIIa; ICD-10: C22.2) are included within the "liver cancer" parent cause, and liver cancer parent codes are used for hepatoblastoma estimation for GBD 2023. Chronic lymphoid leukaemia is only modelled for ages 20 years and above in GBD 2023; thus, relevant ICD codes (ICD-9: 204.1, 204.10, 204.11, and 204.12; ICD-10: C91.1, C91.10, C91.11, and C91.12) under 20 years are redistributed to "Acute lymphoid leukaemia" (see section "6.

Redistribution" in this appendix for more information), while these ICD codes over 20 years old are mapped directly to "Chronic lymphoid leukaemia".

Appendix Table 3: List of ICD codes mapped to GBD cause list for cancer mortality data cause list for cancer mortality data

GBD cause	ICCC-3	ICD-10	ICD-9
Lip and oral cavity cancer	Xif1	C0, C00, C00.0, C00.1, C00.2, C00.3, C00.4, C00.5, C00.6, C00.8, C00.9, C01, C01.9, C02, C02.0, C02.1, C02.2, C02.3, C02.4, C02.8, C02.9, C03, C03.0, C03.1, C03.9, C04, C04.0, C04.1, C04.8, C04.9, C05, C05.0, C05.1, C05.2, C05.8, C05.9, C06, C06.0, C06.1, C06.2, C06.8, C06.80, C06.89, C06.9, C07, C07.0, C07.9, C08, C08.0, C08.1, C08.8, C08.9, D00.00, D00.01, D00.02, D00.03, D00.04, D00.05, D00.06, D00.07, D010.0, D010.1, D010.2, D010.3, D010.30, D010.39, D010.4, D010.5, D11, D011.7, D11.9, D37.01, D37.02, D37.03, D37.030, D37.031, D37.032, D37.039, D37.04, D37.09	140, 140.0, 140.1, 140.2, 140.3, 140.4, 140.5, 140.6, 140.7, 140.8, 140.9, 141, 141.0, 141.1, 141.2, 141.3, 141.4, 141.5, 141.6, 141.8, 141.9, 142, 142.0, 142.1, 142.2, 142.3, 142.8, 142.9, 143, 143.0, 143.1, 143.8, 143.9, 144, 144.0, 144.1, 144.4, 144.8, 144.9, 145, 145.0, 145.1, 145.2, 145.3, 145.4, 145.5, 145.6, 145.8, 145.9, 210.0, 210.1, 210.2, 210.3, 210.4, 210.5, 210.6, 235.0
Nasopharynx cancer	Xic	C11, C11.0, C11.1, C11.2, C11.3, C11.8, C11.9, D00.08, D10.6, D37.05	147, 147.0, 147.1, 147.2, 147.3, 147.8, 147.9, 210.7, 210.8, 210.9
Other pharynx cancer	NA	C09, C09.0, C09.1, C09.8, C09.9, C1, C10, C10.0, C10.1, C10.2, C10.3, C10.4, C10.8, C10.9, C12, C12.0, C12.9, C13, C13.0, C13.1, C13.2, C13.8, C13.9, D10.7	146, 146.0, 146.1, 146.2, 146.3, 146.4, 146.5, 146.6, 146.7, 146.8, 146.9, 148, 148.0, 148.1, 148.2, 148.3, 148.4, 148.5, 148.8, 148.9
Oesophageal cancer	NA	C15, C15.0, C15.1, C15.2, C15.3, C15.4, C15.5, C15.8, C15.9, D00.1, D13.0	150, 150.0, 150.1, 150.2, 150.3, 150.4, 150.5, 150.6, 150.7, 150.8, 150.9, 211.0, 230.1
Stomach cancer	NA	C16, C16.0, C16.1, C16.2, C16.3, C16.4, C16.5, C16.6, C16.7, C16.8, C16.9, D00.2, D13.1, D37.1	151, 151.0, 151.1, 151.2, 151.3, 151.4, 151.5, 151.6, 151.8, 151.9, 209.23, 209.63, 211.1, 230.2
Colon and rectum cancer	Xif2, Xif3	C18, C18.0, C18.1, C18.2, C18.3, C18.4, C18.5, C18.6, C18.7, C18.8, C18.9, C19, C19.0, C19.9, C2, C20, C20.0, C20.8, C20.9, C21, C21.0, C21.1, C21.2, C21.8, C21.9, D01.0, D01.1, D01.2, D01.3, D12, D12.0, D12.1, D12.2, D12.3, D12.4, D12.5, D12.6, D12.7, D12.8, D12.9, D37.3, D37.4, D37.5	153, 153.0, 153.1, 153.2, 153.3, 153.4, 153.5, 153.6, 153.7, 153.8, 153.9, 154, 154.0, 154.1, 154.2, 154.3, 154.4, 154.8, 154.9, 209.1, 209.10, 209.11, 209.12, 209.13, 209.14, 209.15, 209.16, 209.17, 209.5, 209.50, 209.51, 209.52, 209.53, 209.54, 209.55, 209.56, 209.57, 211.3, 211.4, 230.4, 230.5, 230.6, 569.0, 569.43, 569.44, 569.84, 569.85
Liver cancer	VIIa, VIIb, VIIc	C22, C22.0, C22.1, C22.2, C22.3, C22.4, C22.5, C22.7, C22.8, D13.4	155, 155.0, 155.1, 155.3, 155.5, 155.9, 211.5
*Hepatoblastoma**	*See footnote*	*See footnote*	*See footnote*
Gallbladder and biliary tract cancer	NA	C23, C23.0, C23.9, C24, C24.0, C24.1, C24.4, C24.8, C24.9, D13.5	156, 156.0, 156.1, 156.2, 156.3, 156.8, 156.9, 209.65, 209.66, 209.67
Pancreatic cancer	XIIa2	C25, C25.0, C25.1, C25.2, C25.3, C25.4, C25.7, C25.8, C25.9, D13.6, D13.7	157, 157.0, 157.1, 157.2, 157.3, 157.4, 157.5, 157.7, 157.8, 157.9, 211.6, 211.7
Larynx cancer	NA	C32, C32.0, C32.1, C32.2, C32.3, C32.8, C32.9, D02.0, D14.1, D38.0	161, 161.0, 161.1, 161.2, 161.3, 161.8, 161.9, 212.1, 231.0, 235.6
Tracheal, bronchus, and lung cancer	XIIa3, Xif4	C33, C33.0, C33.2, C33.9, C34, C34.0, C34.00, C34.01, C34.02, C34.1, C34.10, C34.11, C34.12, C34.2, C34.3, C34.30, C34.31, C34.32, C34.4, C34.7, C34.8, C34.80, C34.81, C34.82, C34.9, C34.90, C34.91, C34.92, D02.1, D02.2, D02.20, D02.21, D02.22, D02.3, D14.2, D14.3, D14.30, D14.31, D14.32, D38.1	162, 162.0, 162.1, 162.2, 162.3, 162.4, 162.5, 162.8, 162.9, 209.21, 209.61, 212.2, 212.3, 231.1, 231.2, 235.7
Malignant neoplasm of bone and articular cartilage	VIII, VIIIa, VIIIb, VIIIc, VIIIc1, VIIIc2, VIIId, VIIId1, VIIId2, VIIId3, VIIId4, VIIIe	C40, C40.0, C40.00, C40.01, C40.02, C40.1, C40.10, C40.11, C40.12, C40.2, C40.20, C40.21, C40.22, C40.3, C40.30, C40.31, C40.32, C40.8, C40.80, C40.81, C40.82, C40.9, C40.90, C40.91, C40.92, C41, C41.0, C41.1, C41.2, C41.3, C41.4, C41.5, C41.6, C41.7, C41.8, C41.9	170, 170.0, 170.1, 170.2, 170.3, 170.4, 170.5, 170.6, 170.7, 170.8, 170.9
Malignant skin melanoma	Xid	C43, C43.0, C43.1, C43.10, C43.11, C43.12, C43.2, C43.20, C43.21, C43.22, C43.3, C43.30, C43.31, C43.39, C43.4, C43.5, C43.51, C43.52, C43.59, C43.6, C43.60, C43.61, C43.62, C43.7, C43.70, C43.71, C43.72, C43.8, C43.9, D03, D030.0, D03.1, D03.10, D03.11, D03.12, D03.2, D03.20, D03.21, D03.22, D03.3, D03.30, D03.39, D03.4, D03.5, D03.51, D03.52,	172, 172.0, 172.1, 172.2, 172.3, 172.4, 172.5, 172.6, 172.7, 172.8, 172.9

GBD cause	ICCC-3	ICD-10	ICD-9
		D03.59, D03.6, D03.60, D03.61, D03.62, D03.7, D03.70, D03.71, D03.72, D03.8, D03.9, D22, D22.0, D22.1, D22.10, D22.11, D22.12, D22.2, D22.20, D22.21, D22.22, D22.3, D22.30, D22.39, D22.4, D22.5, D22.6, D22.60, D22.61, D22.62, D22.7, D22.70, D22.71, D22.72, D22.9, D23, D23.0, D23.1, D23.10, D23.11, D23.12, D23.2, D23.20, D23.21, D23.22, D23.3, D23.30, D23.39, D23.4, D23.5, D23.6, D23.60, D23.61, D23.62, D23.7, D23.70, D23.71, D23.72, D23.9, D48.5	
Non-melanoma skin cancer	Xie	C44, C44.0, C44.00, C44.01, C44.02 C44.09, C44.1, C44.10, C44.101, C44.102, C44.109, C44.11, C44.111, C44.112, C44.119, C44.112, C44.119, C44.12, C44.121, C44.122, C44.129, C44.19, C44.191, C44.192, C44.199, C44.2, C44.20, C44.201, C44.202, C44.209, C44.21, C44.211, C44.212, C44.222, C44.229, C44.29, C44.291, C44.292, C44.299, C44.3, C44.30, C44.300, C44.301, C44.309, C44.31, C44.310, C44.311, C44.319, C44.32, C44.321, C44.329, C44.39, C44.390, C44.391, C44.399, C44.4, C44.40, C44.41, C44.42, C44.49, C44.5, C44.50, C44.500, C44.501, C44.509, C44.51, C44.510, C44.511, C44.519, C44.52, C44.520, C44.51, C44.529, C44.59, C44.590, C44.591, C44.599, C44.6, C44.60, C44.601, C44.602, C44.609, C44.61, C44.611, C44.612, C44.619, C44.62, C44.621, C44.622, C44.629, C44.69, C44.691, C44.692, C44.699, C44.7, C44.70, C44.701, C44.702, C44.709, C44.71, C44.711, C44.712, C44.719, C44.72, C44.721, C44.722, C44.729, C44.79, C44.791, C44.792, C44.799, C44.8, C44.80, C44.81, C44.82, C44.89, C44.9, C44.90, C44.91, C44.92, C44.99, D04, D04.0, D04.1, D04.10, D04.11, D04.12, D04.2, D04.20, D04.21, D04.22, D04.3, D04.30, D04.39, D04.4, D04.5, D04.6, D04.60, D04.61, D04.62, D04.7, D04.70, D04.71, D04.72, D04.8, D04.9, D49.2	173, 173.0, 173.00, 173.01, 173.02, 173.09, 173.1, 173.10, 173.11, 173.12, 173.19, 173.2, 173.20, 173.21, 173.22, 173.29, 173.3, 173.30, 173.31, 173.32, 173.39, 173.4, 173.40, 173.41, 173.42, 173.49, 173.5, 173.50, 173.51, 173.52, 173.59, 173.6, 173.60, 173.61, 173.62, 173.69, 173.7, 173.70, 173.71, 173.72, 173.79, 173.8, 173.80, 173.81, 173.82, 173.89, 173.9, 173.90, 173.91, 173.92, 173.99, 222.4, 232, 232.0, 232.1, 232.2, 232.3, 232.4, 232.5, 232.6, 232.7, 232.8, 232.9, 238.2
Non-melanoma skin cancer (squamous-cell carcinoma)	*NA*	*C44, C44.0, C44.00, C44.01, C44.02 C44.09, C44.1, C44.10, C44.101, C44.102, C44.109, C44.11, C44.111, C44.112, C44.119, C44.112, C44.119, C44.12, C44.121, C44.122, C44.129, C44.19, C44.191, C44.192, C44.199, C44.2, C44.20, C44.201, C44.202, C44.209, C44.21, C44.211, C44.212, C44.222, C44.229, C44.29, C44.291, C44.292, C44.299, C44.3, C44.30, C44.300, C44.301, C44.309, C44.31, C44.310, C44.311, C44.319, C44.32, C44.321, C44.329, C44.39, C44.390, C44.391, C44.399, C44.4, C44.40, C44.41, C44.42, C44.49, C44.5, C44.50, C44.500, C44.501, C44.509, C44.51, C44.510, C44.511, C44.519, C44.52, C44.520, C44.51, C44.529, C44.59, C44.590, C44.591, C44.599, C44.6, C44.60, C44.601, C44.602, C44.609, C44.61, C44.611, C44.612, C44.619, C44.62, C44.621, C44.622, C44.629, C44.69, C44.691, C44.692, C44.699, C44.7, C44.70, C44.701, C44.702, C44.709, C44.71, C44.711, C44.712, C44.719, C44.72, C44.721, C44.722, C44.729, C44.79, C44.791, C44.792, C44.799, C44.8, C44.80, C44.81, C44.82, C44.89, C44.9, C44.90, C44.91, C44.92, C44.99, D04, D04.0, D04.1, D04.10, D04.11, D04.12, D04.2, D04.20, D04.21, D04.22, D04.3, D04.30, D04.39, D04.4, D04.5, D04.6, D04.60, D04.61, D04.62, D04.7, D04.70, D04.71, D04.72, D04.8, D04.9, D49.2*	*173, 173.0, 173.00, 173.01, 173.02, 173.09, 173.1, 173.10, 173.11, 173.12, 173.19, 173.2, 173.20, 173.21, 173.22, 173.29, 173.3, 173.30, 173.31, 173.32, 173.39, 173.4, 173.40, 173.41, 173.42, 173.49, 173.5, 173.50, 173.51, 173.52, 173.59, 173.6, 173.60, 173.61, 173.62, 173.69, 173.7, 173.70, 173.71, 173.72, 173.79, 173.8, 173.80, 173.81, 173.82, 173.89, 173.9, 173.90, 173.91, 173.92, 173.99, 222.4, 232, 232.0, 232.1, 232.2, 232.3, 232.4, 232.5, 232.6, 232.7, 232.8, 232.9, 238.2*
Mesothelioma	XIIa5	C45, C45.0, C45.1, C45.2, C45.3, C45.4, C45.5, C45.6, C45.7, C45.8, C45.9	NA
Neuroblastoma and other peripheral nervous cell tumours	Iva, Ivb	C47, C47.0, C47.1, C47.10, C47.11, C47.12, C47.2, C47.20, C47.21, C47.22, C47.3, C47.4, C47.5, C47.6, C47.8, C47.9, C74.90	NA
Soft tissue and other extraosseous sarcomas	IX, Ixa, Ixb, Ixb1, Ixb2, Ixb3, Ixd, Ixd1, Ixd10, Ixd11, Ixd2, Ixd3, Ixd4, Ixd5, Ixd6, Ixd7, Ixd8, Ixd9, Ixe	C49, C49.0, C49.1, C49.10, C49.11, C49.12, C49.2, C49.20, C49.21, C49.22, C49.3, C49.4, C49.5, C49.6, C49.8, C49.9	171, 171.0, 171.2, 171.3, 171.4, 171.5, 171.6, 171.7, 171.8, 171.9
Breast cancer	Xif6	C50, C50.0, C50.01, C50.011, C50.012, C50.019, C50.02, C50.021, C50.022, C50.029, C50.1, C50.11, C50.111, C50.112, C50.119, C50.12, C50.121, C50.122, C50.129, C50.2, C50.21, C50.211, C50.212, C50.219, C50.22, C50.221, C50.222, C50.229, C50.3, C50.31, C50.311, C50.312, C50.319, C50.32, C50.321, C50.322, C50.329, C50.4, C50.41, C50.411, C50.412, C50.419, C50.42, C50.421, C50.422, C50.429, C50.5, C50.51, C50.511, C50.512, C50.519, C50.52, C50.521, C50.522, C50.529, C50.6, C50.61, C50.611, C50.612, C50.619, C50.62, C50.621, C50.622, C50.629, C50.7, C50.8, C50.81, C50.811, C50.812, C50.819, C50.82,	174, 174.0, 174.1, 174.2, 174.3, 174.4, 174.5, 174.6, 174.8, 174.9, 175, 175.0, 175.3, 175.9, 217.0, 217.8, 233.0, 238.3, 239.3, 610, 610.0, 610.1, 610.2, 610.3, 610.4, 610.8, 610.9

GBD cause	ICCC-3	ICD-10	ICD-9
		C50.821, C50.822, C50.829, C50.9, C50.91, C50.911, C50.912, C50.919, C50.92, C50.921, C50.922, C50.929, D05, C50, D05.00, D05.01, D05.02, D05.1, D05.10, D05.11, D05.12, D05.7, D05.8, D05.80, D05.81, D05.82, D05.9, D05.90, D05.91, D05.92, D24, D24.0, D24.1, D24.2, D24.9, D48.6, D48.60, D48.61, D48.62, D49.3	
Cervical cancer	Xif7	C53, C53.0, C53.1, C53.3, C53.4, C53.8, C53.9, D06, D06.0, D06.1, D06.7, D06.9, D26.0	180, 180.0, 180.1, 180.2, 180.3, 180.4, 180.5, 180.6, 180.8, 180.9, 219.0, 233.1, 622.1, 622.10, 622.11, 622.12, 622.2, 622.7
Uterine cancer	NA	C54, C54.0, C54.1, C54.2, C54.3, C54.4, C54.8, C54.9, D07.0, D07.1, D07.2, D26.1, D26.7, D26.9	182, 182.0, 182.1, 182.8, 182.9, 233.2
Ovarian cancer	Xc, Xd, Xe	C56, C56.0, C56.1, C56.2, C56.4, C56.9, D27, D27.0, D27.1, D27.9, D39.1, D39.10, D39.11, D39.12	183, 183.0, 220, 220.0, 220.9, 236.2
Prostate cancer	NA	C61, C61.0, C61.9, D07.5, D29.1, D40.0	185, 185.0, 185.9, 222.2, 236.5
Testicular cancer	Xc, Xd, Xe	C62, C62.0, C62.00, C62.01, C62.02, C62.1, C62.10, C62.11, C62.12, C62.9, C62.90, C62.91, C62.92, D29.2, D29.20, D29.21, D29.22, D29.3, D29.30, D29.31, D29.32, D29.4, D29.7, D29.8, D40.1, D40.10, D40.11, D40.12, D40.7, D40.8	186, 186.0, 186.9, 222.0, 222.3, 236.4
Kidney cancer	VI, Via, Via1, Via2, Via3, Via4, Vib, Vic	C64, C64.0, C64.1, C64.2, C64.4, C64.5, C64.6, C64.8, C64.9, C65, C65.0, C65.1, C65.2, C65.9, D30.0, D30.00, D30.01, D30.02, D30.1, D30.10, D30.11, D30.12, D41.0, D41.00, D41.01, D41.02, D41.1, D41.10, D41.11, D41.12	189.0, 189.1, 189.5, 189.6, 209.24, 209.64, 223.0, 223.1, 236.91
Bladder cancer	Xif8	C67, C67.0, C67.1, C67.2, C67.3, C67.4, C67.5, C67.6, C67.7, C67.8, C67.9, D09.0, D30.3, D41.4, D41.7, D41.8, D49.4	188, 188.0, 188.1, 188.2, 188.3, 188.4, 188.5, 188.6, 188.7, 188.8, 188.9, 233.3, 233.7, 236.7, 239.4
Eye cancer	V, Xif9	C69.0, C69.00, C69.01, C69.02, C69.1, C69.10, C69.11, C69.12, C69.2, C69.20, C69.21, C69.22, C69.3, C69.30, C69.31, C69.32, C69.4, C69.40, C69.41, C69.42, C69.5, C69.50, C69.51, C69.52, C69.6, C69.60, C69.61, C69.62, C69.7, C69.8, C69.80, C69.81, C69.82	190, 190.0, 190.1, 190.2, 190.3, 190.4, 190.5, 190.6, 190.7, 190.8
Retinoblastoma	*V*	*C69.2, C69.20, C69.21, C69.22*	*190.5*
Other eye cancers	*Xif9*	*C69.0, C69.00, C69.01, C69.02, C69.1, C69.10, C69.11, C69.12, C69.3, C69.30, C69.31, C69.32, C69.4, C69.40, C69.41, C69.42, C69.5, C69.50, C69.51, C69.52, C69.6, C69.60, C69.61, C69.62, C69.7, C69.8, C69.80, C69.81, C69.82*	*190, 190.0, 190.1, 190.2, 190.3, 190.4, 190.6, 190.7, 190.8*
Brain and central nervous system cancer	III, IIIa, IIIa1, IIIa2, IIIb, IIIc, IIIc1, IIIc2, IIIc3, IIIc4, IIId, IIId1, IIId2, IIId3, IIIe, IIIe1, IIIe2, IIIe3, IIIe4, IIIe5, IIIf, Xa, Xa1, Xa2, Xa3, Xa4, Xa5, Xa6	C70, C70.0, C70.1, C70.5, C70.6, C70.9, C71, C71.0, C71.1, C71.2, C71.3, C71.4, C71.5, C71.6, C71.7, C71.8, C71.9, C72, C72.0, C72.1, C72.2, C72.20, C72.21, C72.22, C72.3, C72.30, C72.31, C72.32, C72.4, C72.40, C72.41, C72.42, C72.5, C72.50, C72.59, C72.8, C72.9, C75.1, C75.2, C75.3	191, 191.0, 191.1, 191.2, 191.3, 191.4, 191.5, 191.6, 191.7, 191.8, 191.9, 192, 192.0, 192.1, 192.2, 192.3, 192.4, 192.8, 192.9, 194.3, 194.4
Thyroid cancer	XIb	C73, C73.0, C73.1, C73.2, C73.3, C73.4, C73.5, C73.8, C73.9, D09.3, D09.8, D34, D34.0, D34.9, D44.0	193, 193.0, 193.2, 193.9, 226, 226.0, 226.9
Other malignant neoplasms	XIIa1, XIIa4, XIIa6, XIIb, XIa, XIf10, XIf11, XIf5, Xb, Xb1, Xb2, Xb3, Xb4, Xb5, Xb6	C17, C17.0, C17.1, C17.2, C17.3, C17.8, C17.9, C3, C30, C30.0, C30.1, C30.2, C30.3, C30.5, C30.8, C30.9, C31, C31.0, C31.1, C31.2, C31.3, C31.8, C31.9, C37, C37.0, C37.1, C37.2, C37.3, C38, C38.0, C38.1, C38.2, C38.3, C38.4, C38.8, C4, C48, C48.0, C48.1, C48.2, C48.8, C48.9, C4A, C5, C51, C51.0, C51.1, C51.2, C51.8, C51.9, C52, C52.0, C52.9, C57, C57.0, C57.00, C57.01, C57.02, C57.1, C57.10, C57.11, C57.12, C57.2, C57.20, C57.21, C57.22, C57.3, C57.4, C57.7, C57.8, C60, C60.0, C60.1, C60.2, C60.8, C60.9, C63, C63.0, C63.00, C63.01, C63.02, C63.1, C63.10, C63.11, C63.12, C63.2, C63.7, C63.8, C66, C66.0, C66.1, C66.2, C66.9, C68.0, C68.1, C68.8, C7, C75, C75.0, C75.4, C75.5, C75.6, C75.8, D07.4, D09.2, D09.20, D09.21, D09.22, D13.2, D13.3, D13.30, D13.39, D14.0, D15, D15.0, D15.1, D15.2, D15.7, D15.9, D16, D16.0, D16.00, D16.01, D16.02, D16.1, D16.10, D16.11, D16.12, D16.2, D16.20, D16.21, D16.22, D16.3, D16.30,D16.31, D16.32, D16.4, D16.5, D16.6, D16.7, D16.8, D16.9, D28.0, D28.1, D28.7, D29.0, D30.2, D30.20, D30.21, D30.22, D30.4, D30.7,	152, 152.0, 152.1, 152.2, 152.3, 152.4, 152.6, 152.8, 152.9, 158, 158.0, 158.3, 158.4, 158.5, 158.6, 158.8, 158.9, 160, 160.0, 160.1, 160.2, 160.3, 160.4, 160.5, 160.6, 160.8, 160.9, 163, 163.0, 163.1, 163.3, 163.5, 163.8, 163.9, 164, 164.0, 164.1, 164.2, 164.3, 164.8, 164.9, 183.2, 183.3, 183.4, 183.5, 183.8, 184.0, 184.1, 184.2, 184.3, 184.4, 184.8, 187.1, 187.2, 187.3, 187.4, 187.5, 187.6, 187.7, 187.8, 189.2, 189.3, 189.4, 189.8,<br>194.1, 194.5, 194.6, 194.8, 209.0, 209.00, 209.01, 209.02, 209.03, 209.22, 209.25, 209.26, 209.27, 209.31, 209.32, 209.33, 209.34, 209.35, 209.36, 209.4. 209.40, 209.41, 209.42, 209.43, 211.2, 211.8, 212.0,

GBD cause	ICCC-3	ICD-10	ICD-9
		D30.8, D31, D31.0, D31.00, D31.01, D31.02, D31.1, D31.10, D31.11, D31.12, D31.2, D31.20, D31.21, D31.22, D31.3, D31.30, D31.31, D31.32, D31.4, D31.40, D31.41, D31.42, D31.5, D31.50, D31.51, D31.52, D31.6, D31.60, D31.61, D31.62, D31.9, D31.90, D31.91, D31.92, D35, D35.0, D35.00, D35.01, D35.02, D35.1, D35.2, D35.5, D35.6, D35.7, D35.8, D35.9, D36, D36.1, D36.10, D36.11, D36.12, D36.13, D36.14, D36.15, D36.16, D36.17, D36.7, D37.2, D38.2, D38.3, D38.4, D38.5, D39.2, D39.8, D41.2, D41.20, D41.21, D41.22, D41.3, D44.1, D44.10, D44.11, D44.12, D44.2, D44.3, D44.4, D44.5, D44.6, D44.7, D44.8, D48.0, D48.1, D48.2, D48.3, D48.4, D49.81	212.4, 212.5, 212.6, 212.7, 212.8, 213, 213.0, 213.1, 213.2, 213.3, 213.4, 213.5, 213.6, 213.7, 213.8, 213.9, 221.0, 221.2, 221.8, 222.1, 222.8, 223.2, 223.8, 223.81, 223.89, 224, 224.0, 224.1, 224.2, 224.3, 224.4, 224.5, 224.6, 224.7, 224.8, 224.9, 227, 227.0, 227.1, 227.3, 227.4, 227.5, 227.6, 227.8, 227.9, 228, 228.0, 228.00, 228.01, 228.02, 228.03, 228.04, 228.09, 228.1, 228.9, 229.0, 229.8, 230.7, 230.8, 233.31, 233.32, 233.4,<br>233.5, 234.0, 234.5, 234.8, 235.4, 235.8, 236.1, 236.99, 238.0, 238.1, 239.2
Hodgkin lymphoma	IIa	C81, C81.0, C81.00, C81.01, C81.02, C81.03, C81.04, C81.05, C81.06, C81.07, C81.08, C81.09, C81.1, C81.10, C81.11, C81.12, C81.13, C81.14, C81.15, C81.16, C81.17, C81.18, C81.19, C81.2, C81.20, C81.21, C81.22, C81.23, C81.24, C81.25, C81.26, C81.27, C81.28, C81.29, C81.3, C81.30, C81.31, C81.32, C81.33, C81.34, C81.35, C81.36, C81.37, C81.38, C81.39, C81.4, C81.40, C81.41, C81.42, C81.43, C81.44, C81.45, C81.46, C81.47, C81.48, C81.49, C81.5, C81.6, C81.7, C81.70, C81.71, C81.72, C81.73, C81.74, C81.75, C81.76, C81.77, C81.78, C81.79, C81.8, C81.9, C81.90, C81.91, C81.92, C81.93, C81.94, C81.95, C81.96, C81.97, C81.98, C81.99	201, 201.0, 201.00, 201.01, 201.02, 201.03, 201.04, 201.05, 201.06, 201.07, 201.08, 201.1, 201.10, 201.11, 201.12, 201.13, 201.14, 201.15, 201.16, 201.17, 201.18, 201.2, 201.20, 201.21, 201.22, 201.23, 201.24,<br>201.25, 201.26, 201.27, 201.28, 201.4, 201.40, 201.41, 201.42, 201.43, 201.44, 201.45, 201.46, 201.47, 201.48, 201.5, 201.50, 201.51, 201.52, 201.53, 201.54, 201.55, 201.56, 201.57, 201.58, 201.6, 201.60, 201.61,<br>201.62, 201.63, 201.64, 201.65, 201.66, 201.67, 201.68, 201.7, 201.70, 201.71, 201.72, 201.73, 201.74, 201.75, 201.76, 201.77, 201.78, 201.9, 201.90, 201.91, 201.92, 201.93, 201.94, 201.95, 201.96, 201.97, 201.98
Non-Hodgkin lymphoma	IIb, IIb1, IIb2, IIb3, IIb4, IIc, IId, IIe	C82, C82.0, C82.00, C82.01, C82.02, C82.03, C82.04, C82.05, C82.06, C82.07, C82.08, C82.09, C82.1, C82.10, C82.11, C82.12, C82.13, C82.14, C82.15, C82.16, C82.17, C82.18, C82.19, C82.2, C82.20, C82.21, C82.22, C82.23, C82.24, C82.25, C82.26, C82.27, C82.28, C82.29, C82.3, C82.30, C82.31, C82.32, C82.33, C82.34, C82.35, C82.36, C82.37, C82.38, C82.39, C82.4, C82.40, C82.41, C82.42, C82.43, C82.44, C82.45, C82.46, C82.47, C82.48, C82.49, C82.5, C82.50, C82.51, C82.52, C82.53, C82.54, C82.55, C82.56, C82.57, C82.58, C82.59, C82.6, C82.60, C82.61, C82.62, C82.63, C82.64, C82.65, C82.66, C82.67, C82.68, C82.69, C82.7, C82.8, C82.80, C82.81, C82.82, C82.83, C82.84, C82.85, C82.86, C82.87, C82.88, C82.89, C82.9, C82.90, C82.91, C82.92, C82.93, C82.94, C82.95, C82.96, C82.97, C82.98, C82.99, C83.0, C83.00, C83.01, C83.02, C83.03, C83.04, C83.05, C83.06, C83.07, C83.08, C83.09, C83.1, C83.10, C83.11, C83.12, C83.13, C83.14, C83.15, C83.16, C83.17, C83.18, C83.19, C83.2, C83.3, C83.30, C83.31, C83.32, C83.33, C83.34, C83.35, C83.36, C83.37, C83.38, C83.39, C83.4, C83.5, C83.50, C83.51, C83.52, C83.53, C83.54, C83.55, C83.56, C83.57, C83.58, C83.59, C83.6, C83.7, C83.70, C83.71, C83.72, C83.73, C83.74, C83.75, C83.76, C83.77, C83.78, C83.79, C83.8, C83.80, C83.81, C83.82, C83.83, C83.84, C83.85, C83.86, C83.87, C83.88, C83.89, C84, C84.0, C84.00, C84.01, C84.02, C84.03, C84.04, C84.05, C84.06, C84.07, C84.08, C84.09, C84.1, C84.10, C84.11, C84.12, C84.13, C84.14, C84.15, C84.16, C84.17, C84.18, C84.19, C84.2, C84.3, C84.4, C84.40, C84.41, C84.42, C84.43, C84.44, C84.45, C84.46, C84.47, C84.48, C84.49, C84.5, C84.6, C84.60, C84.61, C84.62, C84.63, C84.64, C84.65, C84.66, C84.67, C84.68, C84.69, C84.7, C84.70, C84.71, C84.72, C84.73, C84.74, C84.75, C84.76, C84.77, C84.78, C84.79, C84.8, C84.9, C84.90, C84.91, C84.92, C84.93, C84.94, C84.95, C84.96, C84.97, C84.98, C84.99, C85, C85.0, C85.1, C85.10, C85.11, C85.12, C85.13, C85.14, C85.15, C85.16, C85.17, C85.18, C85.19, C85.2, C85.20, C85.21, C85.22, C85.23, C85.24, C85.25, C85.26, C85.27, C85.28, C85.29, C85.3, C85.4, C85.5, C85.6, C85.7, C85.8, C85.80, C85.81, C85.82, C85.83, C85.84, C85.85, C85.86, C85.87, C85.88, C85.89, C86, C86.0, C86.1, C86.2, C86.3, C86.4, C86.5, C86.6, C96, C96.0, C96.1, C96.2, C96.3, C96.4, C96.5, C96.6, C96.7, C96.8, C96.9	200, 200.0, 200.00, 200.01, 200.02, 200.03, 200.04, 200.05, 200.06, 200.07, 200.08, 200.1, 200.10, 200.11, 200.12, 200.13, 200.14, 200.15, 200.16, 200.17, 200.18, 200.2, 200.20, 200.21, 200.22, 200.23, 200.24, 200.25, 200.26, 200.27, 200.28, 200.3, 200.30, 200.31, 200.32, 200.33, 200.34, 200.35, 200.36, 200.37, 200.38, 200.4, 200.40, 200.41, 200.42, 200.43, 200.44, 200.45, 200.46, 200.47, 200.48, 200.5, 200.50, 200.51, 200.52, 200.53, 200.54, 200.55, 200.56, 200.57, 200.58, 200.6, 200.60, 200.61, 200.62, 200.63, 200.64, 200.65, 200.66, 200.67, 200.68, 200.7, 200.70, 200.71, 200.72, 200.73, 200.74, 200.75, 200.76, 200.77, 200.78, 200.8, 200.80, 200.81, 200.82, 200.83, 200.84, 200.85, 200.86, 200.87, 200.88, 200.9, 202, 202.0, 202.00, 202.01, 202.02, 202.03, 202.04, 202.05, 202.06, 202.07, 202.08, 202.1, 202.10, 202.11, 202.12, 202.13, 202.14, 202.15, 202.16, 202.17, 202.18, 202.2, 202.20, 202.21, 202.22, 202.23, 202.24, 202.25, 202.26, 202.27, 202.28, 202.3, 202.30, 202.31, 202.32, 202.33, 202.34, 202.35, 202.36, 202.37, 202.38, 202.4, 202.40, 202.41, 202.42, 202.43, 202.44, 202.45, 202.46, 202.47, 202.48, 202.5, 202.50, 202.51, 202.52, 202.53, 202.54, 202.55, 202.56, 202.57, 202.58, 202.6, 202.60, 202.61, 202.62, 202.63, 202.64, 202.65, 202.66, 202.67, 202.68, 202.7, 202.70, 202.71, 202.72, 202.73, 202.74, 202.75, 202.76, 202.77, 202.78, 202.8, 202.80, 202.81, 202.82, 202.83, 202.84, 202.85, 202.86, 202.87, 202.88
Burkitt lymphoma	*IIc*	*C83.7, C83.70, C83.71, C83.72, C83.73, C83.74, C83.75, C83.76, C83.77, C83.78, C83.79*	*200.2, 200.20, 200.21, 200.22, 200.23, 200.24, 200.25, 200.26, 200.27, 200.28*

GBD cause	ICCC-3	ICD-10	ICD-9
Other non-Hodgkin lymphoma	*IIb, IIb1, IIb2, IIb3, IIb4, IId, IIe*	*C82, C82.0, C82.00, C82.01, C82.02, C82.03, C82.04, C82.05, C82.06, C82.07, C82.08, C82.09, C82.1, C82.10, C82.11, C82.12, C82.13, C82.14, C82.15, C82.16, C82.17, C82.18, C82.19, C82.2, C82.20, C82.21, C82.22, C82.23, C82.24, C82.25, C82.26, C82.27, C82.28, C82.29, C82.3, C82.30, C82.31, C82.32, C82.33, C82.34, C82.35, C82.36, C82.37, C82.38, C82.39, C82.4, C82.40, C82.41, C82.42, C82.43, C82.44, C82.45, C82.46, C82.47, C82.48, C82.49, C82.5, C82.50, C82.51, C82.52, C82.53, C82.54, C82.55, C82.56, C82.57, C82.58, C82.59, C82.6, C82.60, C82.61, C82.62, C82.63, C82.64, C82.65, C82.66, C82.67, C82.68, C82.69, C82.7, C82.8, C82.80, C82.81, C82.82, C82.83, C82.84, C82.85, C82.86, C82.87, C82.88, C82.89, C82.9, C82.90, C82.91, C82.92, C82.93, C82.94, C82.95, C82.96, C82.97, C82.98, C82.99, C83.0, C83.00, C83.01, C83.02, C83.03, C83.04, C83.05, C83.06, C83.07, C83.08, C83.09, C83.1, C83.10, C83.11, C83.12, C83.13, C83.14, C83.15, C83.16, C83.17, C83.18, C83.19, C83.2, C83.3, C83.30, C83.31, C83.32, C83.33, C83.34, C83.35, C83.36, C83.37, C83.38, C83.39, C83.4, C83.5, C83.50, C83.51, C83.52, C83.53, C83.54, C83.55, C83.56, C83.57, C83.58, C83.59, C83.6, C83.8, C83.80, C83.81, C83.82, C83.83, C83.84, C83.85, C83.86, C83.87, C83.88, C83.89, C84, C84.0, C84.00, C84.01, C84.02, C84.03, C84.04, C84.05, C84.06, C84.07, C84.08, C84.09, C84.1, C84.10, C84.11, C84.12, C84.13, C84.14, C84.15, C84.16, C84.17, C84.18, C84.19, C84.2, C84.3, C84.4, C84.40, C84.41, C84.42, C84.43, C84.44, C84.45, C84.46, C84.47, C84.48, C84.49, C84.5, C84.6, C84.60, C84.61, C84.62, C84.63, C84.64, C84.65, C84.66, C84.67, C84.68, C84.69, C84.7, C84.70, C84.71, C84.72, C84.73, C84.74, C84.75, C84.76, C84.77, C84.78, C84.79, C84.8, C84.9, C84.90, C84.91, C84.92, C84.93, C84.94, C84.95, C84.96, C84.97, C84.98, C84.99, C85, C85.0, C85.1, C85.10, C85.11, C85.12, C85.13, C85.14, C85.15, C85.16, C85.17, C85.18, C85.19, C85.2, C85.20, C85.21, C85.22, C85.23, C85.24, C85.25, C85.26, C85.27, C85.28, C85.29, C85.3, C85.4, C85.5, C85.6, C85.7, C85.8, C85.80, C85.81, C85.82, C85.83, C85.84, C85.85, C85.86, C85.87, C85.88, C85.89, C86, C86.0, C86.1, C86.2, C86.3, C86.4, C86.5, C86.6, C96, C96.0, C96.1, C96.2, C96.3, C96.4, C96.5, C96.6, C96.7, C96.8, C96.9*	*200, 200.0, 200.00, 200.01, 200.02, 200.03, 200.04, 200.05, 200.06, 200.07, 200.08, 200.1, 200.10, 200.11, 200.12, 200.13, 200.14, 200.15, 200.16, 200.17, 200.18, 200.3, 200.30, 200.31, 200.32, 200.33, 200.34, 200.35, 200.36, 200.37, 200.38, 200.4, 200.40, 200.41, 200.42, 200.43, 200.44, 200.45, 200.46, 200.47, 200.48, 200.5, 200.50, 200.51, 200.52, 200.53, 200.54, 200.55, 200.56, 200.57, 200.58, 200.6, 200.60, 200.61, 200.62, 200.63, 200.64, 200.65, 200.66, 200.67, 200.68, 200.7, 200.70, 200.71, 200.72, 200.73, 200.74, 200.75, 200.76, 200.77, 200.78, 200.8, 200.80, 200.81, 200.82, 200.83, 200.84, 200.85, 200.86, 200.87, 200.88, 200.9, 202, 202.0, 202.00, 202.01, 202.02, 202.03, 202.04, 202.05, 202.06, 202.07, 202.08, 202.1, 202.10, 202.11, 202.12, 202.13, 202.14, 202.15, 202.16, 202.17, 202.18, 202.2, 202.20, 202.21, 202.22, 202.23, 202.24, 202.25, 202.26, 202.27, 202.28, 202.3, 202.30, 202.31, 202.32, 202.33, 202.34, 202.35, 202.36, 202.37, 202.38, 202.4, 202.40, 202.41, 202.42, 202.43, 202.44, 202.45, 202.46, 202.47, 202.48, 202.5, 202.50, 202.51, 202.52, 202.53, 202.54, 202.55, 202.56, 202.57, 202.58, 202.6, 202.60, 202.61, 202.62, 202.63, 202.64, 202.65, 202.66, 202.67, 202.68, 202.7, 202.70, 202.71, 202.72, 202.73, 202.74, 202.75, 202.76, 202.77, 202.78, 202.8, 202.80, 202.81, 202.82, 202.83, 202.84, 202.85, 202.86, 202.87, 202.88*
Multiple myeloma	NA	C88, C88.0, C88.1, C88.2, C88.20, C88.3, C88.4, C88.40, C88.7, C88.70, C88.71, C88.8, C88.9, C89, C90, C90.0, C90.00, C90.01, C90.02, C90.1, C90.10, C90.11, C90.12, C90.2, C90.20, C90.21, C90.22, C90.3, C90.30, C90.31, C90.32, C90.4, C90.5, C90.6, C90.7, C90.8, C90.9	203, 203.0, 203.00, 203.01, 203.02, 203.1, 203.10, 203.11, 203.12, 203.8, 203.80, 203.81, 203.82, 203.9
Leukaemia	Ia, Ia1, Ia2, Ia3, Ia4, Ib, Ic, Ie	C91, C92, C93, C94, C95, C95.0, C95.00, C95.01, C95.02, C95.1, C95.10, C95.11, C95.12, C95.2, C95.4, C95.6, C95.7, C95.9, C95.90, C95.91, C95.92	204.0, 204.00, 204.01, 204.02, 204.2, 204.20, 204.21, 204.22, 205.0, 205.00, 205.01, 205.02, 205.1, 205.10, 205.11, 205.12, 205.2, 205.20, 205.21, 205.22, 205.3, 205.30, 205.31, 205.32, 205.92, 206.0, 206.00, 206.01, 206.02, 206.1, 206.10, 206.11, 206.12, 207.0, 207.00, 207.01, 207.02, 207.1, 207.10, 207.11, 207.12, 207.2, 207.21, 207.22, 207.8, 207.80, 207.81, 207.82, 207.9
*Acute lymphoid leukaemia**	*Ia, Ia1, Ia2, Ia3, Ia4*	*C91.0, C91.00, C91.01, C91.02, C91.2, C91.3, C91.30, C91.31, C91.32, C91.6, C91.60, C91.61, C91.62, custom mapping (see footnote)*	*204.0, 204.00, 204.01, 204.02, 204.2, 204.20, 204.21, 204.22, custom mapping (see footnote)*
*Chronic lymphoid leukaemia**	*NA*	*Custom mapping (see footnote)*	*Custom mapping (see footnote)*
Acute myeloid leukaemia	*Ib*	*C92.0, C92.00, C92.01, C92.02, C92.3, C92.30, C92.31, C92.32, C92.4, C92.40, C92.41, C92.42, C92.5, C92.50, C92.51, C92.52, C92.6, C92.60, C92.61, C92.62, C93.0, C93.00, C93.01, C93.02, C94.0, C94.00, C94.01, C94.02, C94.2, C94.20, C94.21, C94.22, C94.4, C94.40, C94.41, C94.42, C94.5*	*205.0, 205.00, 205.01, 205.02, 205.2, 205.20, 205.21, 205.22, 205.3, 205.30, 205.31, 205.32, 206.0, 206.00, 206.01, 206.02, 207.0, 207.00, 207.01, 207.02, 207.2, 207.20, 207.21, 207.22, 207.8, 207.80, 207.81, 207.82*
Chronic myeloid leukaemia	*Ic*	*C92.1, C92.10, C92.11, C92.12, C92.2, C92.20, C92.21, C92.22*	*205.1, 205.10, 205.11, 205.12*
Other leukaemia	*Ie*	*C93.1, C93.10, C93.11, C93.12, C93.3, C93.30, C93.31, C93.32, C93.8, C94.1, C94.3, C94.30, C94.31, C94.32, C94.7, C94.8, C94.80, C94.81, C94.82*	*205.92, 206.1, 206.10, 206.11, 206.12, 207, 207.1, 207.10, 207.11, 207.12, 207.9*

GBD cause	ICCC-3	ICD-10	ICD-9
Other neoplasms	Id	D32, D32.0, D32.1, D32.9, D33, D33.0, D33.1, D33.2, D33.3, D33.4, D33.7, D33.9, D35.2, D35.3, D35.4, D42, D42.0, D42.1, D42.9, D43, D43.0, D43.1, D43.2, D43.3, D43.4, D43.7, D43.8, D43.9, D45, D45.0, D45.9, D46, D46.0, D46.1, D46.2, D46.20, D46.21, D46.22, D46.3, D46.4, D46.5, D46.7, D46.9, D47, D47.0, D47.1, D47.2, D47.3, D47.4, D47.5, D47.7, D47.9, D49.6	225, 225.0, 225.1, 225.2, 225.3, 225.4, 225.8, 225.9, 237, 237.0, 237.1, 237.2, 237.3, 237.5, 237.6, 237.7, 237.70, 237.71, 237.72, 237.73, 237.79, 237.9, 238.4, 238.5, 238.6, 238.7, 238.71, 238.72, 238.73, 238.74, 238.75, 238.76, 238.77, 238.79, 238.8, 238.9, 239.6
Myelodysplastic, myeloproliferative, and other haemopoietic neoplasms	*Id*	*D45, D45.0, D45.9, D46, D46.0, D46.1, D46.2, D46.20, D46.21, D46.22, D46.3, D46.4, D46.5, D46.7, D46.9, D47, D47.0, D47.1, D47.2, D47.3, D47.4, D47.5, D47.7, D47.9*	*238.4, 238.5, 238.6, 238.7, 238.71, 238.72, 238.73, 238.74, 238.75, 238.76, 238.77, 238.79, 238.8, 238.9*
Other benign and in situ neoplasms	*NA*	*D32, D32.0, D32.1, D32.9, D33, D33.0, D33.1, D33.2, D33.3, D33.4, D33.7, D33.9, D35.2, D35.3, D35.4, D42, D42.0, D42.1, D42.9, D43, D43.0, D43.1, D43.2, D43.3, D43.4, D43.7, D43.8, D43.9, D49.6*	*225, 225.0, 225.1, 225.2, 225.3, 225.4, 225.8, 225.9, 237, 237.0, 237.1, 237.5, 237.6, 237.7, 237.70, 237.71, 237.72, 237.73, 237.79, 237.9, 239.6*

Abbreviations: ICCC-3, International Classification of Childhood Cancer, Third Edition; ICD-9, International Classification of Diseases, Ninth Revision; ICD-10, International Classification of Diseases, Tenth Revision; NA, not applicable (i.e., no relevant codes mapped for that coding system). Level 4 causes are italicized and listed under their Level 3 parent cause. For example, "*Burkitt lymphoma*" and "*Other non-Hodgkin lymphoma*" are Level 4 causes within the Level 3 cause "Non-Hodgkin lymphoma". *Hepatoblastoma codes (ICCC-3: VIIa; ICD-10: C22.2) are included within the "liver cancer" parent cause, and liver cancer parent codes are used for hepatoblastoma estimation for GBD 2023. Chronic lymphoid leukaemia is only modelled for ages 20 years and above in GBD 2023; thus, relevant ICD codes (ICD-9: 204.1, 204.10, 204.11, and 204.12; ICD-10: C91.1, C91.10, C91.11, and C91.12) under 20 years are redistributed to "Acute lymphoid leukaemia" (see section "6. Redistribution" in this appendix for more information), while these ICD codes over 20 years old are mapped directly to "Chronic lymphoid leukaemia".

Data sources

GBD causes of death (CoD) database contains cancer mortality data originating from multiple sources, including vital registration (VR), verbal autopsy (VA), and population-based cancer registry (CR) data. The cancer registry mortality estimates that are uploaded into the CoD database stem from cancer registry incidence data that have been transformed to mortality estimates through the use of mortality-to-incidence ratios (MIRs).

A summary of GBD 2023 data sources specific to cancer type, location, and year can be found in Appendix 1 Table 4. Across all cancer types, there were a unique total of 19 485 unique site-years from vital registration systems (2097 new for GBD 2023), 4499 site-years from cancer registries (144 new for GBD 2023), and 421 site-years from verbal autopsy reports (99 new for GBD 2023). These counts include locations at the most detailed level of the GBD location hierarchy. Of note, the decrease in VR data from GBD 2021 to GBD 2023 is mainly due to the change in the GBD location hierarchy for the United Kingdom, where the most-detailed location was reduced from the 150 level 6 Upper Tier Local Authorities (UTLAs) in GBD 2021 to the 9 level 5 regions in GBD 2023. The decrease in CR data from GBD 2021 to GBD 2023 is mainly due to a change in data preparation, which now excludes CR data from mortality modeling when there is VR data available for the same granularity (by cause, age, sex, year, and location; see Cancer registry data processing, step 10 below).

Appendix Table 4: Number of site-years for cancer mortality data by source type, for GBD 2023 compared with GBD 2021

GBD cause	VR GBD 2021	VR GBD 2023	VR % change GBD 2021 to GBD 2023	VA GBD 2021	VA GBD 2023	VA % change GBD 2021 to GBD 2023	CR GBD 2021	CR GBD 2023	CR % change GBD 2021 to GBD 2023	Total GBD 2021	Total GBD 2023	Total % change GBD 2021 to GBD 2023
Lip and oral cavity cancer	22524	18236	-19.0	176	182	3.4	4690	2349	-49.9	27390	20767	-24.2
Nasopharynx cancer	22515	18481	-17.9	NA	NA	NA	5172	1447	-72.0	27687	19928	-28.0
Other pharynx cancer	22519	18685	-17.0	NA	NA	NA	5154	1830	-64.5	27673	20515	-25.9
Esophageal cancer	22807	18974	-16.8	166	235	41.6	5263	2237	-57.5	28236	21446	-24.0
Stomach cancer	22813	18978	-16.8	NA	NA	NA	5324	2884	-45.8	28137	21862	-22.3
Colon and rectum cancer	22815	18855	-17.4	216	265	22.7	5343	3047	-43.0	28374	22167	-21.9
Liver cancer	21767	17606	-19.1	NA	NA	NA	5275	2631	-50.1	27042	20237	-25.2
Hepatoblastoma	*21934*	*18116*	*-17.4*	*NA*	*NA*	*NA*	*954*	*787*	*-17.5*	*22888*	*18903*	*-17.4*
Liver cancer due to alcohol use	*20036*	*15746*	*-21.4*	*NA*	*NA*	*NA*	*NA*	*NA*	*NA*	*20036*	*15746*	*-21.4*
Liver cancer due to hepatitis B	*20036*	*15746*	*-21.4*	*NA*	*NA*	*NA*	*NA*	*NA*	*NA*	*20036*	*15746*	*-21.4*
Liver cancer due to hepatitis C	*20036*	*15746*	*-21.4*	*NA*	*NA*	*NA*	*NA*	*NA*	*NA*	*20036*	*15746*	*-21.4*
Liver cancer due to NASH	*20036*	*15746*	*-21.4*	*NA*	*NA*	*NA*	*NA*	*NA*	*NA*	*20036*	*15746*	*-21.4*
Liver cancer due to other causes	*20036*	*15746*	*-21.4*	*NA*	*NA*	*NA*	*NA*	*NA*	*NA*	*20036*	*15746*	*-21.4*
Gallbladder and biliary tract cancer	22225	18332	-17.5	NA	NA	NA	5193	1819	-65.0	27418	20151	-26.5
Pancreatic cancer	22183	18223	-17.9	NA	NA	NA	5166	2414	-53.3	27349	20637	-24.5
Larynx cancer	22807	18849	-17.4	NA	NA	NA	5274	2324	-55.9	28081	21173	-24.6
Tracheal, bronchus, and lung cancer	23316	19482	-16.4	185	262	41.6	5323	3301	-38.0	28824	23045	-20.0
Malignant neoplasm of bone and articular cartilage	21320	17335	-18.7	NA	NA	NA	5286	2012	-61.9	26606	19347	-27.3
Malignant skin melanoma	22509	18551	-17.6	NA	NA	NA	5175	1637	-68.4	27684	20188	-27.1
Non-melanoma skin cancer	22119	17835	-19.4	NA	NA	NA	NA	NA	NA	22119	17835	-19.4
Non-melanoma skin cancer (squamous-cell carcinoma)	*22132*	*17848*	*-19.4*	*NA*	*NA*	*NA*	*NA*	*NA*	*NA*	*22132*	*17848*	*-19.4*
Mesothelioma	13199	11747	-11.0	NA	NA	NA	2490	1404	-43.6	15689	13151	-16.2
Neuroblastoma and other peripheral nervous cell tumors	20343	16177	-20.5	NA	NA	NA	4467	1731	-61.2	24810	17908	-27.8
Soft tissue and other extraosseous sarcomas	19424	15460	-20.4	NA	NA	NA	4706	2062	-56.2	24130	17522	-27.4

Breast cancer	23292	19458	-16.5	295	374	26.8	5348	3533	-33.9	28935	23365	-19.3
Cervical cancer	22778	18942	-16.8	NA	1	NA	5042	2539	-49.6	27820	21482	-22.8
Uterine cancer	22685	18724	-17.5	NA	NA	NA	5031	2514	-50.0	27716	21238	-23.4
Ovarian cancer	22146	18310	-17.3	NA	NA	NA	5028	2273	-54.8	27174	20583	-24.3
Prostate cancer	22715	18881	-16.9	NA	NA	NA	5061	3084	-39.1	27776	21965	-20.9
Testicular cancer	22279	18365	-17.6	NA	NA	NA	4855	1494	-69.2	27134	19859	-26.8
Kidney cancer	20385	18119	-11.1	NA	NA	NA	5113	2604	-49.1	25498	20723	-18.7
Bladder cancer	22492	18657	-17.1	NA	NA	NA	5100	2366	-53.6	27592	21023	-23.8
Eye cancer	20469	16290	-20.4	NA	NA	NA	4194	1567	-62.6	24663	17857	-27.6
Retinoblastoma	*20318*	*16200*	*-20.3*	*NA*	*NA*	*NA*	*4031*	*1498*	*-62.8*	*24349*	*17698*	*-27.3*
Other eye cancers	*20378*	*16213*	*-20.4*	*NA*	*NA*	*NA*	*4063*	*1545*	*-62.0*	*24441*	*17758*	*-27.3*
Brain and central nervous system cancer	21946	18112	-17.5	NA	NA	NA	5249	2250	-57.1	27195	20362	-25.1
Thyroid cancer	22342	18481	-17.3	NA	NA	NA	5250	1894	-63.9	27592	20375	-26.2
Other malignant neoplasms	22154	18200	-17.8	NA	NA	NA	5304	1846	-65.2	27458	20046	-27.0
Hodgkin lymphoma	22124	18109	-18.1	NA	NA	NA	5171	1619	-68.7	27295	19728	-27.7
Non-Hodgkin lymphoma	22518	18676	-17.1	NA	NA	NA	5218	2347	-55.0	27736	21023	-24.2
Burkitt lymphoma	*20375*	*16113*	*-20.9*	*NA*	*NA*	*NA*	*5300*	*2135*	*-59.7*	*25675*	*18248*	*-28.9*
Other non-Hodgkin lymphoma	*20375*	*16210*	*-20.4*	*NA*	*NA*	*NA*	*5316*	*2176*	*-59.1*	*25691*	*18386*	*-28.4*
Multiple myeloma	21848	17888	-18.1	NA	NA	NA	5170	1547	-70.1	27018	19435	-28.1
Leukemia	22981	19023	-17.2	NA	NA	NA	5268	1801	-65.8	28249	20824	-26.3
Acute lymphoid leukemia	*16591*	*14919*	*-10.1*	*NA*	*NA*	*NA*	*3088*	*1293*	*-58.1*	*19679*	*16212*	*-17.6*
Chronic lymphoid leukemia	*16696*	*14291*	*-14.4*	*NA*	*NA*	*NA*	*2970*	*1255*	*-57.7*	*19666*	*15546*	*-20.9*
Acute myeloid leukemia	*17668*	*14951*	*-15.4*	*NA*	*NA*	*NA*	*3065*	*1305*	*-57.4*	*20733*	*16256*	*-21.6*
Chronic myeloid leukemia	*19414*	*15024*	*-22.6*	*NA*	*NA*	*NA*	*2972*	*1257*	*-57.7*	*22386*	*16281*	*-27.3*
Other leukemia	*17074*	*14922*	*-12.6*	*NA*	*NA*	*NA*	*2521*	*1128*	*-55.3*	*19595*	*16050*	*-18.1*
Other neoplasms	20505	16202	-21.0	NA	NA	NA	NA	NA	NA	20505	16202	-21.0
Myelodysplastic, myeloproliferative, and other hematopoietic neoplasms	*13889*	*12553*	*-9.6*	*NA*	*NA*	*NA*	*NA*	*NA*	*NA*	*13889*	*12553*	*-9.6*
Other benign and in situ neoplasms	*19209*	*4323*	*-77.5*	*NA*	*NA*	*NA*	*NA*	*NA*	*NA*	*19209*	*4323*	*-77.5*
All cause total*	1089097	882656	-19.0	1038	1319	27.1	194983	84872	-56.5	1285118	968847	-24.6
All cause total (unique years and locations)**	23319	19485	-16.4	335	421	25.7	5567	4499	-19.2	29221	24405	-16.5

Abbreviations: CR, cancer registry; GBD, Global Burden of Diseases, Injuries, and Risk Factors Study 2023; NA, not applicable; VA, verbal autopsy; VR, vital registry. GBD causes are ordered by ICD code. Total columns refer to all data sources combined based on the respective iteration of the GBD study. Level 4 causes are italicized and listed under their Level 3 parent causes. For example, "*Burkitt lymphoma*" and "*Other non-Hodgkin lymphoma*" are Level 4 causes under the Level 3 parent cause, "Non-Hodgkin lymphoma". VA sources are not available for some cancer causes due to inherent limitations of these heterogeneous survey instruments to capture deaths due to those cancers. For more detail, see the GBD 2023 publication, "Global burden of 292 causes of death in 204 countries and territories and 660 subnational locations, 1990–2023: a systematic analysis for the Global Burden of Disease Study 2023. Lancet (in review)".[2] *"All cause total" is the sum of all causes. **"All cause total (unique years and locations)" is the number of unique location and year combinations for each source type.

Cancer registry data sources

Cancer incidence and mortality data were sought from individual cancer registries, such as the Surveillance, Epidemiology, and End Results (SEER) Program;[13] provided by collaborators; or downloaded from aggregated databases of cancer registry data such as "Cancer Incidence In Five Continents" (CI5),[14–24] EUREG,[25] or NORDCAN.[26] Population-based cancer registries were included that satisfied the following criteria: the registry must have included all cancers (i.e., were not specialty registries for a subset of cancer types), reported data for all age groups (with the exception of paediatric cancer registries), and reported data for both sexes. Hospital-based and pathology-based cancer registries were excluded. Redundant cancer registry data were excluded from either the final incidence data input or the MIR model input if a more detailed source (e.g., one which provides more detailed age or diagnostic groups) was available for the same population. Preference was given to cancer registries with national coverage over those with subnational coverage, except those from countries where the GBD study provides subnational estimates. Data were excluded if the coverage population was unknown, except in high Socio-demographic Index (SDI) quintile locations with full geographic coverage where the GBD estimated population could be substituted.

A list of the cancer registries included in our analysis and the years covered can be found in Appendix 1 Table 5. We used data from GBD 2021 and added registry data from Benin, China, Congo, Côte d'Ivoire, Eswatini, Ghana, Kenya, Lithuania, Mali, Mozambique, Niger, Nigeria, North Macedonia, Spain, Türkiye, Uganda, United Republic of Tanzania, and Zambia. Additional metadata for each source are available in the online GBD citation tool, https://ghdx.healthdata.org/gbd-2023.

Appendix Table 5: Cancer registry sources for cancer incidence and mortality-to-incidence ratio data by country, year, and registry

Location	Registry	Years of incidence data available	Years used for incidence	Years of mortality data available	Years used for mortality	Years available for MIRs	Years used for MIRs
Algeria	5 Registries Combined (Algiers, Annaba, Batna, Setif, Tlemcen)	1996-2014	19	0	0	0	0
Algeria	Algiers	1993-1997, 2004-2016	18	0	0	0	0
Algeria	Batna	2000-2006, 2008-2012	12	0	0	0	0
Algeria	Mascara	2000-2010	11	0	0	0	0
Algeria	Oran	2005-2006	2	0	0	0	0
Algeria	Setif	1986-2011	26	0	0	0	0
Argentina	6 Registries Combined (Bahia Blanca, Chaco, Cordoba, Entre Rios, Mendoza, Neuquen)	1991-2013	23	0	0	0	0
Argentina	Bahia Blanca	1993-2007	15	0	0	0	0
Argentina	Chaco	2008-2012	5	0	0	0	0
Argentina	Concordia	1990-1997	8	0	0	0	0
Argentina	Cordoba	2004-2013	10	0	0	0	0
Argentina	Entre Rios Province	2008-2011	4	0	0	0	0
Argentina	Mendoza	2003-2014	12	0	0	0	0
Argentina	National Registry	2006-2008	3	2006-2008, 2016	4	2006-2008	3
Argentina	Neuquen	2003-2017	15	0	0	0	0
Argentina	Oncopaediatric Registry	2000-2008	9	0	0	0	0
Argentina	El Registro Oncopediátrico Hospitalario Argentino (ROHA)	2000-2016	17	2000-2016	17	2000-2016	17
Argentina	Tierra del Fuego	2003-2012	10	0	0	0	0
Australia	Capital Territory	1983-2008	26	2004-2008	5	0	0
Australia	National Registry	1982-2014	33	1968-2014	35	1982-2014	33
Australia	New South Wales	1983-2012	30	0	0	0	0
Australia	Northern Territory	1998-2012	15	0	0	0	0
Australia	Queensland	1982-2015	34	1982-2015	34	2015	1
Australia	South Australia	1977-2012	33	0	0	0	0

Australia	Tasmania	1978-2012, 2016	34	2001-2006, 2016	7	2016	1
Australia	Victoria	1970-2012, 2017	34	1982-2007, 2017	27	2017	1
Australia	Western Australia	1983-2012	30	0	0	0	0
Austria	Carinthia	2008-2012	5	0	0	0	0
Austria	National Registry	1983-2016	34	1990-2016	23	1996, 2001, 2006, 2010	4
Austria	Tyrol	1988-2012	25	0	0	0	0
Austria	Vorarlberg	1993-2012	20	0	0	0	0
Bahrain	National Registry	1998-2012	15	0	0	0	0
Belarus	Belarus Paediatric	1986-2014	29	0	0	0	0
Belarus	National Registry	1983-2012	30	0	0	0	0
Belgium	Antwerp	1998-2002	5	0	0	0	0
Belgium	Flanders	1998-2001	4	0	0	0	0
Belgium	Flanders except Limburg	1997-1998	2	0	0	0	0
Belgium	Limburg	1997-1998	2	0	0	0	0
Belgium	National Registry	1999-2013	15	0	0	0	0
Benin	Cotonou Cancer Registry	2013-2016	4	0	0	0	0
Bermuda	Bermuda	1975-2018	39	1975-2018	39	1996	1
Bolivia (Plurinational State of)	La Paz	1978-1979	0	0	0	0	0
Botswana	National Registry	1999-2013	15	0	0	0	0
Brazil	Angra Dos Reis	2007-2008	2	0	0	0	0
Brazil	Aracaju	1996-2013	18	0	0	0	0
Brazil	Barretos	2008-2013	6	0	0	0	0
Brazil	Belem	1987-1991, 1996-2012	22	0	0	0	0
Brazil	Belo Horizonte	2000-2011	12	0	0	0	0
Brazil	Brasilia	1998-2001	4	0	0	0	0
Brazil	Campinas	1991-2005	15	0	0	0	0
Brazil	Campo Grande	2000-2005, 2008-2010	9	0	0	0	0
Brazil	Cuiaba	2000-2009	10	0	0	0	0
Brazil	Curitiba	1996, 1998-2012	16	0	0	0	0
Brazil	Distrito Federal	1999-2002	4	0	0	0	0
Brazil	Espirito Santo	1997-2012	16	0	0	0	0
Brazil	Florianopolis	2008-2012	5	0	0	0	0
Brazil	Fortaleza	1978-1982, 1990-2009	23	0	0	0	0
Brazil	Goiania	1987-2012	26	0	0	0	0
Brazil	Jahu	1996-2015	20	0	0	0	0
Brazil	Joao Pessoa	1999-2012	14	0	0	0	0
Brazil	Manaus	1999-2009	11	0	0	0	0
Brazil	Mato Grosso Interior	2001-2005	5	0	0	0	0
Brazil	Natal	1999-2005	7	0	0	0	0
Brazil	Palmas	2000-2012	13	0	0	0	0
Brazil	Pocos de Caldas	2007-2011	5	0	0	0	0
Brazil	Porto Alegre	1979-1982, 1987, 1990-2006	21	0	0	0	0
Brazil	Recife	1968-1971, 1980, 1995-2012	19	0	0	0	0
Brazil	Roraima	2003-2010	8	0	0	0	0
Brazil	Salvador	1996-2005	10	0	0	0	0
Brazil	Santos	2008-2009	2	0	0	0	0
Brazil	Sao Paulo	1969, 1973, 1978, 1997-2013	17	0	0	0	0
Brazil	Teresina	2000-2006	7	0	0	0	0
Brunei Darussalam	Darussalam	2002-2017	16	0	0	0	0
Brunei Darussalam	National Registry	2010-2012	3	0	0	0	0
Bulgaria	National Registry	1980-2013	34	1993-2003, 2006, 2011-2012	3	2006, 2011-2012	3
Cameroon	Yaounde	2004-2006	3	0	0	0	0

Canada	Alberta	1960-1966, 1969-2013	34	0	0	0	0
Canada	British Columbia	1969-2013	34	0	0	0	0
Canada	Manitoba	1958-2013	34	0	0	0	0
Canada	Maritime	1969-1987	8	0	0	0	0
Canada	National Registry	1978-2007	28	0	0	0	0
Canada	New Brunswick	1962-1966, 1978-2013	34	0	0	0	0
Canada	Newfoundland	1969-2002	23	0	0	0	0
Canada	Newfoundland and Labrador	1960-1966, 1969-2012	33	0	0	0	0
Canada	Northwest Territories	1983-2012	30	0	0	0	0
Canada	Northwest Territories and Yukon	1973-1987	8	0	0	0	0
Canada	Nova Scotia	1978-2015	36	0	0	0	0
Canada	Nunavut	2008-2012	5	0	0	0	0
Canada	Ontario	1969-1971, 1978-2012	33	0	0	0	0
Canada	Prince Edward Island	1978-2012	33	0	0	0	0
Canada	Quebec	1963-1966, 1969-2010	31	0	0	0	0
Canada	Saskatchewan	1960-1966, 1968-2014	35	2008-2014	7	2008-2014	7
Canada	Yukon	1983-1997, 2003-2012	25	0	0	0	0
Chile	4 Registries Combined (Antofagasta, Bio Bio, Concepcion, Valdivia)	1998-2012	15	0	0	0	0
Chile	Antofagasta	2003-2010	8	0	0	0	0
Chile	Bio Bio	2003-2012	10	0	0	0	0
Chile	Chile Paediatric	2007-2011	5	0	0	0	0
Chile	Concepcion	2008-2010	3	0	0	0	0
Chile	Los Rios	2003-2007	5	0	0	0	0
Chile	National Registry	1959-1961, 2007-2011	5	2007-2011	5	2009	1
Chile	Valdivia	1998-2012	15	0	0	0	0
China	Anfu County	2014	1	2014	1	2014	1
China	Anguo	2013-2014	2	2013-2014	2	2013-2014	2
China	Anshan	1998-2014	17	1998-2009, 2011-2014	16	1998-2009, 2011-2014	16
China	Aohanqi of Chifeng	2013-2014	2	2013-2014	2	2013-2014	2
China	Arun	2014	1	2014	1	2014	1
China	Baoding	2009, 2011-2014	5	2009, 2011-2014	5	2009, 2011-2014	5
China	Baoxing	2013-2014	2	2013-2014	2	2013-2014	2
China	Baoying	2013-2014	2	2013-2014	2	2013-2014	2
China	Bayannaoer	2011	1	2011	1	2011	1
China	Beijing	1990-2014	25	1990-2009, 2011, 2013-2014	23	1990-2009, 2011, 2013-2014	23
China	Beijing Rural Areas	2011-2014	4	2011-2014	4	2011-2014	4
China	Beilin District, Xi'an	2014	1	2014	1	2014	1
China	Beiliu	2011-2014	4	2011-2014	4	2011-2014	4
China	Bengbu	2011-2012, 2014	3	2011-2012, 2014	3	2011-2012, 2014	3
China	Benxi	2003-2014	12	2003-2009, 2011-2014	11	2003-2009, 2011-2014	11
China	Bijiang District, Tongren	2011	1	2011	1	2011	1
China	Bincheng District, Binzhou	2011-2014	4	2011-2014	4	2011-2014	4
China	Binghai	2011-2014	4	2011-2014	4	2011-2014	4
China	Binyang County	2014	1	2014	1	2014	1
China	Boli	2011, 2014	2	2011, 2014	2	2011, 2014	2
China	Cangwu	2011-2014	4	2011-2014	4	2011-2014	4
China	Cangzhou	2011-2014	4	2011-2014	4	2011-2014	4
China	Ceheng County	2014	1	2014	1	2014	1
China	Changfeng	2011-2014	4	2011-2014	4	2011-2014	4
China	Changjiang	2014	1	2014	1	2014	1

China	Changle	1990-2009, 2011-2014	24	1990-2009, 2011-2014	24	1990-2009, 2011-2014	24
China	Changle	2004, 2006-2007	3	0	0	0	0
China	Changning	2011, 2013-2014	3	2011, 2013-2014	3	2011, 2013-2014	3
China	Changtai	2013-2014	2	2013-2014	2	2013-2014	2
China	Changxing County	2014	1	2014	1	2014	1
China	Changzhou	2011-2014	4	2011-2014	4	2011-2014	4
China	Chaohu	2013-2014	2	2013-2014	2	2013-2014	2
China	Chifeng	2009, 2011-2012, 2014	4	2009, 2011-2012, 2014	4	2009, 2011-2012, 2014	4
China	Chuzhou District, Huai'an	2004, 2006-2007	3	0	0	0	0
China	Ci County	1990-2012, 2014	24	1990-2009, 2014	21	1990-2009, 2014	21
China	Cili	2011-2014	4	2011-2014	4	2011-2014	4
China	Cixi	2011-2014	4	2011-2014	4	2011-2014	4
China	Cixian	2011-2013	3	2011-2013	3	2011-2013	3
China	Daan	2011	1	2011	1	2011	1
China	Dafeng	2003-2009, 2011-2014	11	2003-2009, 2011-2014	11	2003-2009, 2011-2014	11
China	Daishan County	2014	1	2014	1	2014	1
China	Dalian City	1998-2012, 2014	16	1998-2009, 2011-2012, 2014	15	1998-2009, 2011-2012, 2014	15
China	Dancheng	2011-2014	4	2011-2014	4	2011-2014	4
China	Dandong	2008-2009, 2011-2014	6	2008-2009, 2011-2014	6	2008-2009, 2011-2014	6
China	Danyang	2012-2014	3	2012-2014	3	2012-2014	3
China	Daoli District, Harbin City	2005-2009, 2011-2014	9	2005-2009, 2011-2014	9	2005-2009, 2011-2014	9
China	Dawa County	2014	1	2014	1	2014	1
China	Dawukou	2011-2014	4	2011-2014	4	2011-2014	4
China	Dazhu	2011-2012, 2014	3	2011-2012, 2014	3	2011-2012, 2014	3
China	Decheng District, Dezhou	2011, 2014	2	2011, 2014	2	2011, 2014	2
China	Dehui	2009, 2011-2014	5	2009, 2011-2014	5	2009, 2011-2014	5
China	Dingan	2011	1	2011	1	2011	1
China	Dingyuan County	2014	1	2014	1	2014	1
China	Diqi	2014	1	2014	1	2014	1
China	Donggang	2009, 2011-2012, 2014	4	2009, 2011-2012, 2014	4	2009, 2011-2012, 2014	4
China	Dongguan	2014	1	2014	1	2014	1
China	Donghai County	2004, 2009, 2011-2014	6	2009, 2011-2014	5	2009, 2011-2014	5
China	Dongtai	2012-2014	3	2012-2014	3	2012-2014	3
China	Duanzhou District, Zhaoqing	2014	1	2014	1	2014	1
China	Dunhua	2014	1	2014	1	2014	1
China	Dunhuang	2011	1	2011	1	2011	1
China	Enshi	2014	1	2014	1	2014	1
China	Ewenke	2013-2014	2	2013-2014	2	2013-2014	2
China	Faku	2011-2012, 2014	3	2011-2012, 2014	3	2011-2012, 2014	3
China	Fangcheng County	2014	1	2014	1	2014	1
China	Feicheng	1998-2009, 2011-2014	16	1998-2009, 2011-2014	16	1998-2009, 2011-2014	16
China	Feidong	2011-2014	4	2011-2014	4	2011-2014	4
China	Feixi County	2009, 2011-2014	5	2009, 2011-2014	5	2009, 2011-2014	5
China	Fengdu County	2014	1	2014	1	2014	1
China	Fengning	2012-2014	3	2012-2014	3	2012-2014	3
China	Funing	2012-2014	3	2012-2014	3	2012-2014	3
China	Fuquan	2014	1	2014	1	2014	1
China	Furong District, Changsha	2014	1	2014	1	2014	1

China	Fusui County	1990-1997, 2003-2009, 2011-2014	19	1990-1997, 2003-2009, 2011-2014	19	1990-1997, 2003-2009, 2011-2014	19
China	Fuxin	2014	1	2014	1	2014	1
China	Fuyang	2011	1	2011	1	2011	1
China	Fuyuan	2011	1	2011	1	2011	1
China	Gan County	2014	1	2014	1	2014	1
China	Ganyu	2004, 2011-2014	5	2011-2014	4	2011-2014	4
China	Ganzhou District, Zhangye	2011-2014	4	2011-2014	4	2011-2014	4
China	Gaoling County	2014	1	2014	1	2014	1
China	Gaomi	2011, 2013-2014	3	2011, 2013-2014	3	2011, 2013-2014	3
China	Gaotang	2011-2014	4	2011-2014	4	2011-2014	4
China	Gejiu	2004, 2008, 2011-2014	6	2008, 2011-2014	5	2008, 2011-2014	5
China	Genhe	2013-2014	2	2013-2014	2	2013-2014	2
China	Gongan	2011-2014	4	2011-2014	4	2011-2014	4
China	Guandu District, Kunming	2012-2014	3	2012-2014	3	2012-2014	3
China	Guangrao	2011, 2013-2014	3	2011, 2013-2014	3	2011, 2013-2014	3
China	Guangzhou City	2000-2014	15	2000-2009, 2011-2014	14	2000-2009, 2011-2014	14
China	Guangzhou Suburban Areas	2011	1	2011	1	0	0
China	Guannan	2011, 2013-2014	3	2011, 2013-2014	3	2011, 2013-2014	3
China	Guanyun County	2004, 2007-2014	9	2007, 2009, 2011-2014	6	2007, 2009, 2011-2014	6
China	Guilin	2011-2014	4	2011-2014	4	2011-2014	4
China	Guyuan	2011	1	2011	1	2011	1
China	Hai'an County	2009, 2011-2014	5	2009, 2011-2014	5	2009, 2011-2014	5
China	Hailar	2013-2014	2	2013-2014	2	2013-2014	2
China	Hailin	2014	1	2014	1	2014	1
China	Haimen	2003-2014	12	2003-2009, 2011-2014	11	2003-2009, 2011-2014	11
China	Hainan	2011-2014	4	2011-2014	4	2011-2014	4
China	Haining	1998-2009, 2011-2014	16	1998-2009, 2011-2014	16	1998-2009, 2011-2014	16
China	Haixing	2013-2014	2	2013-2014	2	2013-2014	2
China	Hangzhou City	2000-2014	15	2000-2009, 2011-2014	14	2000-2009, 2011-2014	14
China	Hanjiang District, Putian	2011-2014	4	2011-2014	4	2011-2014	4
China	Hanyuan	2013-2014	2	2013-2014	2	2013-2014	2
China	Hebi	2012-2014	3	2012-2014	3	2012-2014	3
China	Hefei	2010-2014	5	2011-2014	4	2011-2014	4
China	Hengdong	2012-2013	2	2012-2013	2	2012-2013	2
China	Hengdong County	2009-2012, 2014	5	2009, 2011, 2014	3	2009, 2011, 2014	3
China	Hengfeng	2013-2014	2	2013-2014	2	2013-2014	2
China	Hepu	2011-2014	4	2011-2014	4	2011-2014	4
China	Hetian	2011	1	2011	1	2011	1
China	Hong Kong Special Administrative Region of China	1974-2018	39	2003-2018	16	2003-2018	16
China	Honghu	2013-2014	2	2013-2014	2	2013-2014	2
China	Hongta District, Yuxi	2011-2014	4	2011-2014	4	2011-2014	4
China	Hongtong	2011, 2014	2	2011, 2014	2	2011, 2014	2
China	Hongze	2011-2014	4	2011-2014	4	2011-2014	4
China	Hotan County	2011	1	2011	1	0	0
China	Huai'an	2012-2014	3	2012-2014	3	2012-2014	3
China	Huai'an District, Huai'an	1998-2009, 2011, 2013-2014	15	1998-2009, 2011, 2013-2014	15	1998-2009, 2011, 2013-2014	15
China	Huaiyin District, Huai'an	2009-2014	6	2009, 2011-2014	5	2009, 2011-2014	5

China	Huangdao District, Qingdao	2011, 2013	2	2011, 2013	2	2011, 2013	2
China	Huangzhong	2013-2014	2	2013-2014	2	2013-2014	2
China	Huian	2011-2013	3	2011-2013	3	2011-2013	3
China	Huichuan District, Zunyi	2011, 2013-2014	3	2011, 2013-2014	3	2011, 2013-2014	3
China	Huinong	2011-2014	4	2011-2014	4	2011-2014	4
China	Huixian	2011-2014	4	2011-2014	4	2011-2014	4
China	Huzhu	2011-2012, 2014	3	2011-2012, 2014	3	2011-2012, 2014	3
China	Jiangdong District, Ningbo	2014	1	2014	1	2014	1
China	Jiange County	2014	1	2014	1	2014	1
China	Jiangjin District, Chongqing	2014	1	2014	1	2014	1
China	Jiangmen	2010-2014	5	2011-2014	4	2011-2014	4
China	Jianhu County	2003-2014	12	2003-2009, 2011-2014	11	2003-2009, 2011-2014	11
China	Jianou	2011, 2013-2014	3	2011, 2013-2014	3	2011, 2013-2014	3
China	Jianping	2011, 2013-2014	3	2011, 2013-2014	3	2011, 2013-2014	3
China	Jiashan County	1990-2014	25	1990-2009, 2011-2014	24	1990-2009, 2011-2014	24
China	Jiaxing	2000-2014	15	2000-2009, 2011-2014	14	2000-2009, 2011-2014	14
China	Jiayu	2012-2014	3	2012-2014	3	2012-2014	3
China	Jilin	2011, 2014	2	2011, 2014	2	2011, 2014	2
China	Jilin City	2013	1	2013	1	2013	1
China	Jinan	2011, 2014	2	2011, 2014	2	2011, 2014	2
China	Jingan	2011, 2013-2014	3	2011, 2013-2014	3	2011, 2013-2014	3
China	Jingshan County	2014	1	2014	1	2014	1
China	Jingtai County	2009, 2011-2014	5	2009, 2011-2014	5	2009, 2011-2014	5
China	Jingxian	2011-2014	4	2011-2014	4	2011-2014	4
China	Jingyang	2011, 2013	2	2011, 2013	2	2011, 2013	2
China	Jinhu County	2007-2009, 2011-2014	7	2007-2009, 2011-2014	7	2007-2009, 2011-2014	7
China	Jintan District	2003-2007, 2009, 2011-2014	10	2003-2007, 2009, 2011-2014	10	2003-2007, 2009, 2011-2014	10
China	Jinzhai	2011	1	2011	1	2011	1
China	Jishan	2013	1	2013	1	2013	1
China	Jiulongpo District, Chongqing	2004, 2007, 2009, 2011-2014	7	2004, 2007, 2009, 2011-2014	7	2004, 2007, 2009, 2011-2014	7
China	Jiyuan	2011-2014	4	2011-2014	4	2011-2014	4
China	Junan	2011-2014	4	2011-2014	4	2011-2014	4
China	Juye	2013-2014	2	2013-2014	2	2013-2014	2
China	Kaifeng	2012-2014	3	2012-2014	3	2012-2014	3
China	Kaifu District, Changsha	2014	1	2014	1	2014	1
China	Kaihua	2011-2014	4	2011-2014	4	2011-2014	4
China	Kailu	2011-2014	4	2011-2014	4	2011-2014	4
China	Kaiyang	2011, 2013-2014	3	2011, 2013-2014	3	2011, 2013-2014	3
China	Kangping	2011-2014	4	2011-2014	4	2011-2014	4
China	Karamay	2014	1	2014	1	2014	1
China	Kelamayi	2013	1	2013	1	2013	1
China	Kunes County	2009	1	2009	1	2009	1
China	Lanping	2011	1	2011	1	2011	1
China	Lanzhou	2011	1	2011	1	2011	1
China	Ledu District, Haidong	2014	1	2014	1	2014	1
China	Leishan	2011	1	2011	1	2011	1
China	Leshan	2011	1	2011	1	2011	1
China	Lhasa	2011-2012	2	2011-2012	2	2011-2012	2
China	Liangshan County	2014	1	2014	1	2014	1
China	Liangzhou District	2008-2009, 2011-2014	6	2008-2009, 2011-2014	6	2008-2009, 2011-2014	6

China	Lianhu District, Xi'an	2011, 2014	2	2011, 2014	2	2011, 2014	2
China	Lianshui	2011-2014	4	2011-2014	4	2011-2014	4
China	Lianyuan	2013-2014	2	2013-2014	2	2013-2014	2
China	Lianyungang	2004, 2007-2014	9	2007-2009, 2011-2014	7	2007-2009, 2011-2014	7
China	Liaoyang	2012-2014	3	2012-2014	3	2012-2014	3
China	Lingbi	2011-2012, 2014	3	2011-2012, 2014	3	2011-2012, 2014	3
China	Lingshui	2014	1	2014	1	2014	1
China	Linhe District, Bayannaoer	2011-2014	4	2011-2014	4	2011-2014	4
China	Linqu	2012-2013	2	2012-2013	2	2012-2013	2
China	Linqu County	1998-2009, 2011, 2014	14	1998-2009, 2011, 2014	14	1998-2009, 2011, 2014	14
China	Lintan	2011-2014	4	2011-2014	4	2011-2014	4
China	Linwu	2013-2014	2	2013-2014	2	2013-2014	2
China	Linxian	2011-2012	2	2011-2012	2	2011-2012	2
China	Linzhou	1990-2014	25	1990-2009, 2011-2014	24	1990-2009, 2011-2014	24
China	Linzi District, Zibo	2013-2014	2	2013-2014	2	2013-2014	2
China	Liuzhou	2009-2014	6	2009, 2011-2014	5	2009, 2011-2014	5
China	Liyang	2011-2014	4	2011-2014	4	2011-2014	4
China	Long County	2014	1	2014	1	2014	1
China	Long'an County	2014	1	2014	1	2014	1
China	Longnan	2011, 2013-2014	3	2011, 2013-2014	3	2011, 2013-2014	3
China	Longquan	2014	1	2014	1	2014	1
China	Longquanyi District, Chengdu	2011, 2014	2	2011, 2014	2	2011, 2014	2
China	Longyang District, Baoshan	2014	1	2014	1	2014	1
China	Luanchuan County	2014	1	2014	1	2014	1
China	Lucheng District, Wenzhou	2014	1	2014	1	2014	1
China	Lujiang	2011, 2013-2014	3	2011, 2013-2014	3	2011, 2013-2014	3
China	Luoshan	2011-2014	4	2011-2014	4	2011-2014	4
China	Luoyang	2011-2014	4	2011-2014	4	2011-2014	4
China	Lushan	2011-2014	4	2011-2014	4	2011-2014	4
China	Lushan County	2013-2014	2	2013-2014	2	2013-2014	2
China	Lusong District, Zhuzhou	2014	1	2014	1	2014	1
China	Luxian	2013-2014	2	2013-2014	2	2013-2014	2
China	Ma'anshan	2003-2014	12	2003-2009, 2011-2014	11	2003-2009, 2011-2014	11
China	Macheng	2011-2014	4	2011-2014	4	2011-2014	4
China	Maiji District, Tianshui	2011	1	2011	1	2011	1
China	Mayang	2011-2014	4	2011-2014	4	2011-2014	4
China	Meihekou	2013-2014	2	2013-2014	2	2013-2014	2
China	Meixian	2011, 2013	2	2011, 2013	2	2011, 2013	2
China	Mengcheng	2012-2014	3	2012-2014	3	2012-2014	3
China	Mengjin County	2014	1	2014	1	2014	1
China	Mingshan	2013-2014	2	2013-2014	2	2013-2014	2
China	Minhe	2011	1	2011	1	2011	1
China	Mudanjiang	2014	1	2014	1	2014	1
China	Naidong	2011, 2013	2	2011, 2013	2	2011, 2013	2
China	Nangang District, Harbin City	1992-2014	23	1992-2009, 2011-2014	22	1992-2009, 2011-2014	22
China	Nanhai District, Foshan	2014	1	2014	1	2014	1
China	Nantong	2011-2014	4	2011-2014	4	2011-2014	4
China	Nanxiong	2012-2014	3	2012-2014	3	2012-2014	3
China	Neiqiu	2013-2014	2	2013-2014	2	2013-2014	2
China	Neixiang	2011-2013	3	2011-2013	3	2011-2013	3
China	Ningyang	2011-2014	4	2011-2014	4	2011-2014	4
China	Nongqishi	2011, 2013	2	2011, 2013	2	2011, 2013	2
China	Nyingchi County	2014	1	2014	1	2014	1
China	Panlong District, Kunming	2014	1	2014	1	2014	1
China	Pengzhou	2011-2014	4	2011-2014	4	2011-2014	4

China	Pingbian	2013-2014	2	2013-2014	2	2013-2014	2
China	Pingding	2012-2014	3	2012-2014	3	2012-2014	3
China	Pingluo	2011, 2013-2014	3	2011, 2013-2014	3	2011, 2013-2014	3
China	Qian'an	2014	1	2014	1	2014	1
China	Qianxi County	2009, 2011-2014	5	2009, 2011-2014	5	2009, 2011-2014	5
China	Qianyang	2013-2014	2	2013-2014	2	2013-2014	2
China	Qidong	1978-2002, 2011-2014	27	2011-2014	4	2011-2014	4
China	Qidong County	1983-1997, 2003-2012	25	0	0	0	0
China	Qidong County	1990-2009, 2011	21	1990-2009, 2011	21	1990-2009, 2011	21
China	Qilin District, Qujing	2014	1	2014	1	2014	1
China	Qingdao	2011, 2014	2	2011, 2014	2	2011, 2014	2
China	Qingdao Urban	2013	1	2013	1	2013	1
China	Qinghe	2011	1	2011	1	2011	1
China	Qingpu District, Huai'an	2011	1	2011	1	2011	1
China	Qingtongxia	2013-2014	2	2013-2014	2	2013-2014	2
China	Qingyang District, Chengdu	2009, 2011-2014	5	2009, 2011-2014	5	2009, 2011-2014	5
China	Qinhuangdao	2011-2014	4	2011-2014	4	2011-2014	4
China	Qionghai	2011-2014	4	2011-2014	4	2011-2014	4
China	Renhe District, Panzhihua	2011, 2014	2	2011, 2014	2	2011, 2014	2
China	Renxian	2013-2014	2	2013-2014	2	2013-2014	2
China	Rudong County	2014	1	2014	1	2014	1
China	Rugao	2012-2014	3	2012-2014	3	2012-2014	3
China	Rushan	2011-2014	4	2011-2014	4	2011-2014	4
China	Ruyang County	2014	1	2014	1	2014	1
China	Sanmenxia	2011-2014	4	2011-2014	4	2011-2014	4
China	Sanya	2011	1	2011	1	2011	1
China	Shan County	2014	1	2014	1	2014	1
China	Shanggao	2011-2014	4	2011-2014	4	2011-2014	4
China	Shanghai	1975, 1978-2014	35	1990-2009, 2011-2014	24	1990-2009, 2011-2014	24
China	Shangyu	2009, 2011	2	2009, 2011	2	2009, 2011	2
China	Shangyu District, Shaoxing	2012-2014	3	2012-2014	3	2012-2014	3
China	Shangzhi	2009, 2011, 2013-2014	4	2009, 2011, 2013-2014	4	2009, 2011, 2013-2014	4
China	Shangzhou District, Shangluo	2011, 2013-2014	3	2011, 2013-2014	3	2011, 2013-2014	3
China	Shaodong	2013	1	2013	1	2013	1
China	Shapingba District, Chongqing	2011-2014	4	2011-2014	4	2011-2014	4
China	Shenqiu	2011-2014	4	2011-2014	4	2011-2014	4
China	Shenyang	2011-2013	3	2011-2013	3	2011-2013	3
China	Shenyang City	2003-2012, 2014	11	2003-2009, 2011, 2014	9	2003-2009, 2011, 2014	9
China	Shenze	2013	1	2013	1	2013	1
China	Shenzhen City	2004, 2011-2014	5	2011-2014	4	2011-2014	4
China	Shexian County	2003-2014	12	2003-2009, 2011-2014	11	2003-2009, 2011-2014	11
China	Sheyang County	2008-2014	7	2008-2009, 2011-2014	6	2008-2009, 2011-2014	6
China	Shifeng District, Zhuzhou	2011-2014	4	2011-2014	4	2011-2014	4
China	Shihezi	2011, 2013-2014	3	2011, 2013-2014	3	2011, 2013-2014	3
China	Shijiazhuang	2013-2014	2	2013-2014	2	2013-2014	2
China	Shimian	2013-2014	2	2013-2014	2	2013-2014	2
China	Shizhong District, Leshan	2012-2014	3	2012-2014	3	2012-2014	3
China	Shouxian	2011-2014	4	2011-2014	4	2011-2014	4
China	Shouyang	2011-2014	4	2011-2014	4	2011-2014	4
China	Shuangqiao District, Chengde	2014	1	2014	1	2014	1

China	Shunde District, Foshan	2014	1	2014	1	2014	1
China	Sihui	1998-2009, 2011-2014	16	1998-2009, 2011-2014	16	1998-2009, 2011-2014	16
China	Song County	2014	1	2014	1	2014	1
China	Suzhou	2006-2009, 2011-2014	8	2006-2009, 2011-2014	8	2006-2009, 2011-2014	8
China	Taixing	2004-2005, 2007-2009, 2011-2014	9	2004-2005, 2007-2009, 2011-2014	9	2004-2005, 2007-2009, 2011-2014	9
China	Tengchong	2011-2014	4	2011-2014	4	2011-2014	4
China	Tengzhou	2011-2014	4	2011-2014	4	2011-2014	4
China	Tianchang	2011-2014	4	2011-2014	4	2011-2014	4
China	Tianjin	1981-2004, 2011-2014	28	1990-2004, 2011-2014	19	1990-2004, 2011-2014	19
China	Tianjin Rural Areas	2011-2014	4	2011-2014	4	2011-2014	4
China	Tianquan	2013-2014	2	2013-2014	2	2013-2014	2
China	Tianshan District, Urumqi	2011, 2013-2014	3	2011, 2013-2014	3	2011, 2013-2014	3
China	Tianxin District, Changsha	2014	1	2014	1	2014	1
China	Tinghu District, Yancheng	2013-2014	2	2013-2014	2	2013-2014	2
China	Tong'an District, Xiamen	2011-2014	4	2011-2014	4	2011-2014	4
China	Tongguan	2011, 2014	2	2011, 2014	2	2011, 2014	2
China	Tonghua	2011-2014	4	2011-2014	4	2011-2014	4
China	Tongling	2008-2014	7	2008-2009, 2011, 2013-2014	5	2008-2009, 2011, 2013-2014	5
China	Tongling County	2012-2013	2	2012-2013	2	2012-2013	2
China	Wangdu County	2014	1	2014	1	2014	1
China	Wangyi District, Tongchuan	2014	1	2014	1	2014	1
China	Wanxiu District, Wuzhou	2014	1	2014	1	2014	1
China	Wanzai County	2014	1	2014	1	2014	1
China	Wanzhou District, Chongqing	2012-2013	2	2012-2013	2	2012-2013	2
China	Wanzhouqu District, Chongqing	2011	1	2011	1	2011	1
China	Weiyang District, Xi'an	2014	1	2014	1	2014	1
China	Wenshang	2012-2013	2	2012-2013	2	2012-2013	2
China	Wenshang County	2009, 2011, 2014	3	2009, 2011, 2014	3	2009, 2011, 2014	3
China	Wolong District, Nanyang	2014	1	2014	1	2014	1
China	Wuan	2011-2014	4	2011-2014	4	2011-2014	4
China	Wuchang	2012	1	2012	1	2012	1
China	Wufeng	2011-2014	4	2011-2014	4	2011-2014	4
China	Wuhan	2012-2014	3	2012-2014	3	2012-2014	3
China	Wuhan City	1990-2012	23	1990-2009, 2011	21	1990-2009, 2011	21
China	Wuhu	2011-2014	4	2011-2014	4	2011-2014	4
China	Wuling District, Changde	2013-2014	2	2013-2014	2	2013-2014	2
China	Wuning	2011-2014	4	2011-2014	4	2011-2014	4
China	Wuwei	2004	1	0	0	0	0
China	Wuxi	2006, 2010-2014	6	2006, 2011-2014	5	2006, 2011-2014	5
China	Xiamen City	2009, 2011-2012	3	2009, 2011-2012	3	2009, 2011-2012	3
China	Xiamen Urban	2011, 2013	2	2011, 2013	2	2011, 2013	2
China	Xiang'an District, Xiamen	2011, 2013	2	2011, 2013	2	2011, 2013	2
China	Xiangfang District, Harbin	2011, 2013	2	2011, 2013	2	2011, 2013	2
China	Xianju	2011-2013	3	2011-2013	3	2011-2013	3
China	Xianju County	2009-2012, 2014	5	2009, 2011, 2014	3	2009, 2011, 2014	3
China	Xihai'an District, Qingdao	2014	1	2014	1	2014	1

China	Xilinhaote	2011, 2014	2	2011, 2014	2	2011, 2014	2
China	Xin'an County	2014	1	2014	1	2014	1
China	Xinghualing District, Taiyuan	2011, 2014	2	2011, 2014	2	2011, 2014	2
China	Xingtai	2013-2014	2	2013-2014	2	2013-2014	2
China	Xining	2009, 2011-2014	5	2009, 2011-2014	5	2009, 2011-2014	5
China	Xinji	2013-2014	2	2013-2014	2	2013-2014	2
China	Xinjian	2012-2014	3	2012-2014	3	2012-2014	3
China	Xinluo District, Longyan	2014	1	2014	1	2014	1
China	Xintian County	2014	1	2014	1	2014	1
China	Xinyuan	2011-2013	3	2011-2013	3	2011-2013	3
China	Xinzhou District, Shangrao	2011-2014	4	2011-2014	4	2011-2014	4
China	Xiping County	2009-2014	6	2009, 2011-2014	5	2009, 2011-2014	5
China	Xishan District, Kunming	2014	1	2014	1	2014	1
China	Xuanhua	2013-2014	2	2013-2014	2	2013-2014	2
China	Xuanwei	2011	1	2011	1	2011	1
China	Xuyi County	2009, 2011-2014	5	2009, 2011-2014	5	2009, 2011-2014	5
China	Xuzhou	2011, 2013-2014	3	2011, 2013-2014	3	2011, 2013-2014	3
China	Yakeshi	2011-2014	4	2011-2014	4	2011-2014	4
China	Yancheng	2011-2012	2	2011-2012	2	2011-2012	2
China	Yancheng District, Luohe	2011-2014	4	2011-2014	4	2011-2014	4
China	Yandu District, Yancheng	2013-2014	2	2013-2014	2	2013-2014	2
China	Yangcheng County	2003-2009, 2011-2014	11	2003-2009, 2011-2014	11	2003-2009, 2011-2014	11
China	Yangquan	2009, 2011-2014	5	2009, 2011-2014	5	2009, 2011-2014	5
China	Yangshan	2011	1	2011	1	2011	1
China	Yangzhong	1998-2009, 2011-2014	16	1998-2009, 2011-2014	16	1998-2009, 2011-2014	16
China	Yanji	2009, 2011-2014	5	2009, 2011-2014	5	2009, 2011-2014	5
China	Yanshan	2013-2014	2	2013-2014	2	2013-2014	2
China	Yanshi	2009-2014	6	2009, 2011-2014	5	2009, 2011-2014	5
China	Yanta District, Xi'an	2014	1	2014	1	2014	1
China	Yantai	2011-2014	4	2011-2014	4	2011-2014	4
China	Yanting County	1998-2014	17	1998-2009, 2011-2014	16	1998-2009, 2011-2014	16
China	Yichang	2013-2014	2	2013-2014	2	2013-2014	2
China	Yicheng	2014	1	2014	1	2014	1
China	Yihuang County	2014	1	2014	1	2014	1
China	Yimen County	2014	1	2014	1	2014	1
China	Yinan County	2014	1	2014	1	2014	1
China	Yinchuan	2011-2012	2	2011-2012	2	2011-2012	2
China	Yingdong District, Fuyang	2011-2014	4	2011-2014	4	2011-2014	4
China	Yingjing	2013-2014	2	2013-2014	2	2013-2014	2
China	Yingkou	2014	1	2014	1	2014	1
China	Yingshan	2011	1	2011	1	2011	1
China	Yingzhou District, Fuyang	2014	1	2014	1	2014	1
China	Yishui	2013-2014	2	2013-2014	2	2013-2014	2
China	Yiyang County	2014	1	2014	1	2014	1
China	Yiyuan	2011, 2013	2	2011, 2013	2	2011, 2013	2
China	Yongan	2012-2013	2	2012-2013	2	2012-2013	2
China	Yongding	2011-2014	4	2011-2014	4	2011-2014	4
China	Yongkang	2013-2014	2	2013-2014	2	2013-2014	2
China	Yongqiao District, Suzhou	2011-2014	4	2011-2014	4	2011-2014	4
China	You County	2014	1	2014	1	2014	1

China	Yuanhui District, Luohe	2011-2013	3	2011-2013	3	2011-2013	3
China	Yuanqu	2011	1	2011	1	2011	1
China	Yucheng	2011-2014	4	2011-2014	4	2011-2014	4
China	Yucheng District, Ya'an	2013-2014	2	2013-2014	2	2013-2014	2
China	Yuci District, Jinzhong	2011	1	2011	1	2011	1
China	Yuelu District, Changsha	2014	1	2014	1	2014	1
China	Yueyanglou	2009-2012	4	2011	1	2011	1
China	Yueyanglou District, Yueyang	2012-2014	3	2012-2014	3	2012-2014	3
China	Yugan County	2014	1	2014	1	2014	1
China	Yuhu District, Xiangtan	2014	1	2014	1	2014	1
China	Yuhua District, Changsha	2014	1	2014	1	2014	1
China	Yun County	2014	1	2014	1	2014	1
China	Yunmeng County	2009, 2011-2014	5	2009, 2011-2014	5	2009, 2011-2014	5
China	Yuxian	2013-2014	2	2013-2014	2	2013-2014	2
China	Yuzhong District, Chongqing	2011-2014	4	2011-2014	4	2011-2014	4
China	Yuzhou	2011-2014	4	2011-2014	4	2011-2014	4
China	Zanhuang	2011-2014	4	2011-2014	4	2011-2014	4
China	Zhangbei	2013-2014	2	2013-2014	2	2013-2014	2
China	Zhanggong District	2009	1	2009	1	2009	1
China	Zhanggong District, Ganzhou	2011-2014	4	2011-2014	4	2011-2014	4
China	Zhangqiu	2011-2014	4	2011-2014	4	2011-2014	4
China	Zhaoling District, Luohe	2011-2014	4	2011-2014	4	2011-2014	4
China	Zhaoyuan	2011-2014	4	2011-2014	4	2011-2014	4
China	Zhenning	2014	1	2014	1	2014	1
China	Zhongning	2013	1	2013	1	2013	1
China	Zhongshan	1998-2013	16	1998-2009, 2011-2013	15	1998-2009, 2011-2013	15
China	Zhongshan County	2004, 2006-2007, 2014	4	2014	1	2014	1
China	Zhongwei	2011-2012, 2014	3	2011-2012, 2014	3	2011-2012, 2014	3
China	Zhongxiang	2011-2014	4	2011-2014	4	2011-2014	4
China	Zhuanghe	2009, 2011-2014	5	2009, 2011-2014	5	2009, 2011-2014	5
China	Zhuhai	2010-2012, 2014	4	2011-2012, 2014	3	2011-2012, 2014	3
China	Zhuixian	2012-2014	3	2012-2014	3	2012-2014	3
China	Ziliujing District	2009	1	2009	1	2009	1
China	Ziliujing District, Zigong	2011-2014	4	2011-2014	4	2011-2014	4
China	Zixing	2011-2014	4	2011-2014	4	2011-2014	4
China	Ziyang District, Yiyang	2013-2014	2	2013-2014	2	2013-2014	2
China	Zoucheng	2011-2014	4	2011-2014	4	2011-2014	4
Colombia	4 Registries Combined (Bucaramanga, Cali, Manizales, Pasto)	1992-2013	22	0	0	0	0
Colombia	Bucaramanga	2003-2012	10	0	0	0	0
Colombia	Cali	1962-2012	33	1984-2011	28	0	0
Colombia	Manizales	2003-2012	10	0	0	0	0
Colombia	National Registry	2003-2010, 2015, 2017	10	0	0	0	0
Colombia	Pasto	2003-2012	10	0	0	0	0
Congo	Registre des Cancers de Brazzaville	2009-2016	8	0	0	0	0
Costa Rica	National Registry	1980-2012	33	2012-2013	2	0	0
Croatia	National Registry	1987-1991, 1993-2014	27	0	0	0	0
Cuba	National Registry	1968-1977, 1986-1990, 2000-2012	18	0	0	0	0
Cuba	Villa Clara	1995-1997, 2004-2007	7	0	0	0	0

Cyprus	Cyprus	1998-2002, 2008-2012	10	0	0	0	0
Cyprus	National Registry	1998-2008	11	0	0	0	0
Cyprus	South West	1998-2012	15	0	0	0	0
Czechia	National Registry	1980-2012	33	1998-2007	10	2003-2007	5
Côte d'Ivoire	Abidjan	2012-2015	4	0	0	0	0
Côte d'Ivoire	National Registry	1995-1997	3	0	0	0	0
Denmark	National Registry	1953-2014	35	1970-2014	35	1980-2014	35
Ecuador	5 Registries Combined (Cuenca, Guayaquil, Loja, Manabi, Quito)	1993-2013	21	0	0	0	0
Ecuador	Cuenca	2003-2012	10	0	0	0	0
Ecuador	Guayaquil	2003-2006, 2008-2012	9	0	0	0	0
Ecuador	Loja	2008-2010	3	0	0	0	0
Ecuador	Manabi	2008-2012	5	0	0	0	0
Ecuador	Quito	1985-2012	28	0	0	0	0
Egypt	Aswan	2008-2010	3	0	0	0	0
Egypt	Damietta	2009, 2011-2012	3	0	0	0	0
Egypt	Gharbiah	1999-2010	12	0	0	0	0
Egypt	Minia	2009	1	0	0	0	0
Estonia	National Registry	1968-2017	38	1981-1982, 1985-2016	30	1996, 1998, 2004, 2006, 2008-2009, 2015	7
Eswatini	Eswatini National Cancer Registry	2016-2017	2	0	0	0	0
Ethiopia	Addis Ababa	2011-2016	6	0	0	0	0
Fiji	National Registry	1998-2010	13	1998-2008	11	1998-2008	11
Finland	National Registry	1953-2014	35	1970-2014	35	1980-2014	35
France	13 Registries Combined (Bas-Rhin, Calvados, Doubs, Gironde, Haut-Rhin, Hérault, Isère, Lille, Limousin, Loire-Atlantique and Vendée, Manche, Somme, Tarn)	1993-2012	20	0	0	0	0
France	Bas Rhin	1975-2011	32	0	0	0	0
France	Brittany	1991-1997	7	0	0	0	0
France	Calvados	1978-2012	33	0	0	0	0
France	Doubs	1977-2012	33	0	0	0	0
France	France Paediatric	2000-2012	13	0	0	0	0
France	Francim Combined (Calvados, Côte-d'Or, Doubs, Finistère, Gironde, Hérault, Isère, Loire-Atlantique, Manche, Marne, Ardennes, Orne, Bas-Rhin, Haut-Rhin, Saône-et-Loire, Somme, Tarn, Vendée	2012, 2018	2	2012, 2018	2	0	0
France	Gironde	2008-2012	5	0	0	0	0
France	Haut Rhin	1988-2012	25	0	0	0	0
France	Herault	1986-2012	27	0	0	0	0
France	Isere	1979-2012	33	0	0	0	0
France	Lille	2008-2012	5	0	0	0	0
France	Limousin	2009-2012	4	0	0	0	0
France	Loire Atlantique	1991-2012	15	0	0	0	0
France	Lorraine	1983-1997	15	0	0	0	0
France	Lorraine Childhood Cancer Registry	1983-1992	10	0	0	0	0
France	Manche	1994-2011	18	0	0	0	0
France	Martinique Cancer Registry	1982-2016	35	0	0	0	0
France	National Registry	1983-1992	10	0	0	0	0
France	Provence, Alps, Coted'Azur, and Corsica	1984-1996	13	0	0	0	0
France	Reunion Cancer Registry	2011-2013	3	0	0	0	0
France	Rhone	1987-2004	18	0	0	0	0

France	Somme	1982-2012	30	0	0	0	0
France	Tarn	1982-2012	30	0	0	0	0
France	Territoire de Belfort	2008-2012	5	0	0	0	0
France	Vendee	1998-2012	15	0	0	0	0
French Polynesia	French Polynesia	1998-2002	5	0	0	0	0
Gambia	National Registry	1987-1998, 2002-2014	25	0	0	0	0
Georgia	National Registry	1972, 1975, 1977-2018	39	0	0	0	0
Germany	5 Western Registries Combined (Bavaria, Lower Saxony, Rhine-Pfalz, Saarland, Schleswig-Holstein)	1994-2012	19	0	0	0	0
Germany	6 Eastern Registries Combined (Berlin, Brandenburg, Mecklenburg, Saxony, Saxony-Anhalt, Thüringen)	2001-2007	7	0	0	0	0
Germany	Bavaria	2002-2012	6	2003-2007	2	0	0
Germany	Berlin	1998-2008	2	2003-2008	2	0	0
Germany	Brandenburg	1998-2008, 2014-2015	13	2003-2008, 2014-2015	6	0	0
Germany	Bremen	2000-2012	10	0	0	0	0
Germany	Eastern States (former GDR)	1964-1966, 1968-1989	10	0	0	0	0
Germany	Free State of Saxony	1998-2008, 2014-2015	13	2003-2008, 2014-2015	4	0	0
Germany	Germany Paediatric	1980-2012	33	0	0	0	0
Germany	Hamburg	1969-1979, 1995-2012	15	1998-2010	10	0	0
Germany	Lower Saxony	2003-2015	10	2006-2009, 2011-2015	9	2006-2009, 2011-2015	9
Germany	Mecklenburg	1998-2007, 2014-2015	2	2003-2007, 2014-2015	2	0	0
Germany	Mecklenburg-West Pomerania	1998-2008	11	2007-2008	2	0	0
Germany	Munich	1998-2012	15	0	0	0	0
Germany	National Registry	1991-2014, 2016	25	1999-2016	18	1999-2014, 2016	17
Germany	North Rhine Westphalia	1998-2007	1	1994-2007	10	0	0
Germany	Rhineland Palatinate	2000-2012	5	0	0	0	0
Germany	Saarland	1968-2012	33	0	0	0	0
Germany	Saxony-Anhalt	1998-2008	2	2003-2008	2	0	0
Germany	Schleswig Holstein	1998-2012	10	0	0	0	0
Germany	Thuringen	1998-2008, 2014-2015	4	2003-2008, 2014-2015	4	0	0
Germany	Westphalia	1998-2012	15	0	0	0	0
Ghana	Kumasi Cancer Registry	2014-2016	3	0	0	0	0
Greenland	Greenland	1980-2014	35	1983-2014	32	1983-2014	32
Grenada	St. George's Central Hospital	1996-2000	5	0	0	0	0
Guinea	Conakry	1992-1995, 2001-2010	14	0	0	0	0
Honduras	Francisco Morazan	2002-2012	11	0	0	0	0
Hungary	County Szabolcs-Szatmar	1962-1966, 1969-1971, 1973-1987	8	0	0	0	0
Hungary	County Vas	1962-1966, 1968-1987	8	0	0	0	0
Hungary	Hungarian Study Group	1985-1990	6	0	0	0	0
Hungary	Hungary Paediatric	1977-2014	35	0	0	0	0
Hungary	Miskolc	1962-1966	0	0	0	0	0
Hungary	National Registry	2001-2011	11	0	0	0	0
Hungary	Northeast Hungary	1984-1998	15	0	0	0	0
Iceland	National Registry	1955-2014	35	1970-2014	35	1980-2014	35
India	Aizawl	2005-2008, 2012-2014	2	0	0	0	0
India	Arunachal Pradesh, Rural		3	0	0	0	0

India	Aurangabad	2005-2010, 2012-2014	2	0	0	0	0
India	Barshi Rural	1988-1992, 2003-2014	3	0	0	0	0
India	Imphal	2005-2006, 2009-2010, 2012-2014	3	0	0	0	0
India	Manipur	2006-2010	5	0	0	0	0
India	Manipur Excl Imphal West	2009-2010, 2012-2014	3	0	0	0	0
India	Manipur, Urban		3	0	0	0	0
India	Mansa District	2013-2014	1	0	0	0	0
India	Meghalaya	2010-2014	5	0	0	0	0
India	Mizoram	2003-2012	10	0	0	0	0
India	Nagaland	2010, 2012-2014	4	0	0	0	0
India	Nagaland, Rural		4	0	0	0	0
India	Nagaland, Urban		4	0	0	0	0
India	Papum Pare	2012-2014	3	0	0	0	0
India	Pasighat	2012-2014	3	0	0	0	0
India	Punjab, Rural		1	0	0	0	0
India	Sangrur District	2013-2014	1	0	0	0	0
India	Sikkim	2003-2014	12	0	0	0	0
India	Tamil Nadu	2012-2013	2	0	0	0	0
India	Tripura	2010-2014	5	0	0	0	0
Iran (Islamic Republic of)	Alborz	2000-2010	11	0	0	0	0
Iran (Islamic Republic of)	Ardabil	1985, 1996-1999, 2006-2008	8	0	0	0	0
Iran (Islamic Republic of)	Ardabil Pediatric	2010-2013	4	0	0	0	0
Iran (Islamic Republic of)	Ardebil	2000-2010	11	0	0	0	0
Iran (Islamic Republic of)	Bushehr	2000-2010	11	0	0	0	0
Iran (Islamic Republic of)	Chahar Mahaal and Bakhtiari	2000-2010	11	0	0	0	0
Iran (Islamic Republic of)	East Azarbayejan	2000-2010	11	0	0	0	0
Iran (Islamic Republic of)	Fars	2000-2010	11	0	0	0	0
Iran (Islamic Republic of)	Gilan	2000-2010	11	0	0	0	0
Iran (Islamic Republic of)	Golestan	1996-2000, 2004-2011	13	0	0	0	0
Iran (Islamic Republic of)	Golestan	2000-2011	12	0	0	0	0
Iran (Islamic Republic of)	Hamadan	2000-2010	11	0	0	0	0
Iran (Islamic Republic of)	Hormozgan	2000-2010	11	0	0	0	0
Iran (Islamic Republic of)	Ilam	2000-2010	11	0	0	0	0
Iran (Islamic Republic of)	Isfahan	2000-2010	11	0	0	0	0
Iran (Islamic Republic of)	Kerman	2000-2010	11	0	0	0	0
Iran (Islamic Republic of)	Kermanshah	2000-2010	11	0	0	0	0
Iran (Islamic Republic of)	Khorasan-e-Razavi	2000-2010	11	0	0	0	0
Iran (Islamic Republic of)	Khuzestan	2000-2010	11	0	0	0	0
Iran (Islamic Republic of)	Kohgiluyeh and Boyer-Ahmad	2000-2010	11	0	0	0	0
Iran (Islamic Republic of)	Kurdistan	2000-2010	11	0	0	0	0
Iran (Islamic Republic of)	Lorestan	2000-2010	11	0	0	0	0
Iran (Islamic Republic of)	Markazi	2000-2010	11	0	0	0	0
Iran (Islamic Republic of)	Mazandaran	2000-2010	11	0	0	0	0

Iran (Islamic Republic of)	National Registry	2003-2007	5	2004-2005	2	2004	1
Iran (Islamic Republic of)	North Khorasan	2000-2010	11	0	0	0	0
Iran (Islamic Republic of)	Qazvin	2000-2010	11	0	0	0	0
Iran (Islamic Republic of)	Qom	2000-2010	11	0	0	0	0
Iran (Islamic Republic of)	Semnan	2000-2010	11	0	0	0	0
Iran (Islamic Republic of)	Sistan and Baluchistan	2000-2010	11	0	0	0	0
Iran (Islamic Republic of)	South Khorasan	2000-2010	11	0	0	0	0
Iran (Islamic Republic of)	Tehran	2000-2010	11	0	0	0	0
Iran (Islamic Republic of)	West Azarbayejan	2000-2010	11	0	0	0	0
Iran (Islamic Republic of)	Yazd	2000-2010	11	0	0	0	0
Iran (Islamic Republic of)	Zanjan	2000-2010	11	0	0	0	0
Iraq	National Registry	2007, 2009, 2011, 2014-2016	6	0	0	0	0
Ireland	National Registry	1994-2012	19	0	0	0	0
Ireland	Southern Ireland	1980-1986, 1988-1992	12	0	0	0	0
Israel	National Registry	1960-1963, 1980-2012	33	0	0	0	0
Italy	Alessandria	2015	1	2015	1	2015	1
Italy	Aosta Valley	2008-2012	5	2013	1	0	0
Italy	Asti	2015	1	2015	1	2015	1
Italy	Barletta	2008-2011	4	0	0	0	0
Italy	Bergamo	2008-2012	5	0	0	0	0
Italy	Biella	1995-2002, 2008-2012, 2015	14	2015	1	2015	1
Italy	Brescia	1999-2001	3	0	0	0	0
Italy	Caserta	2008-2010	3	0	0	0	0
Italy	Catania, Messina and Enna	2008-2012	5	0	0	0	0
Italy	Chieri-Carmagnola-Moncalieri-Nichelino	2015	1	2015	1	2015	1
Italy	Cirie-Chivasso-Ivrea	2015	1	2015	1	2015	1
Italy	Collegno-Pinerolo	2015	1	2015	1	2015	1
Italy	Como	2008-2011	4	0	0	0	0
Italy	Cremona	2008-2010	3	0	0	0	0
Italy	Cuneo	2015	1	2015	1	2015	1
Italy	Ferrara	1991-2002, 2008-2011	16	0	0	0	0
Italy	Florence	1985-2002	18	0	0	0	0
Italy	Florence and Prato	1985-2002, 2008-2010	21	0	0	0	0
Italy	Friuli Venezia Giulia	2008-2010	3	2013	1	0	0
Italy	Genoa	1986-1996, 1998-2000	14	0	0	0	0
Italy	Latina	1983-1991, 2008-2012	14	0	0	0	0
Italy	Lecco	2008-2010	3	0	0	0	0
Italy	Liguria	1986-1991	6	2013	1	0	0
Italy	Lombardy Pediatric	1980-1991, 1996-1997	14	0	0	0	0
Italy	Macerata	1991-2000	10	0	0	0	0
Italy	Mantua	2008-2010	3	0	0	0	0
Italy	Marche Paediatric	1998-2007	10	0	0	0	0
Italy	Milan	1999-2002, 2008-2012	9	0	0	0	0
Italy	Modena	1988-2012	25	0	0	0	0
Italy	Monza	2008-2012	5	0	0	0	0
Italy	Naples	1998-2002, 2008-2012	10	0	0	0	0

Italy	National Registry	1986-2013	28	2006-2009, 2013	5	2006-2009	4
Italy	North East Italy	1995-2002	8	0	0	0	0
Italy	Novara	2015	1	2015	1	2015	1
Italy	Nuoro	2008-2012	5	0	0	0	0
Italy	Palermo	2008-2012	5	0	0	0	0
Italy	Parma	1978-2002, 2008-2012	28	0	0	0	0
Italy	Pavia	2008-2010	3	0	0	0	0
Italy	Piacenza	2008-2011	4	0	0	0	0
Italy	Piedmont	2008-2012, 2015	6	2008-2013, 2015	7	2010, 2015	2
Italy	Piedmont Paediatric	1967-2011	32	0	0	0	0
Italy	Ragusa	1981-2002	22	0	0	0	0
Italy	Ragusa and Caltanissetta	2008-2012	5	0	0	0	0
Italy	Reggio Emilia	1998-2002, 2008-2012	10	2013	1	0	0
Italy	Romagna	1986-2002, 2008-2012	22	0	0	0	0
Italy	Salerno	1998-2001	4	0	0	0	0
Italy	Sassari	1993-2002, 2008-2011	14	0	0	0	0
Italy	Sondrio	1998-2012	15	0	0	0	0
Italy	South Tyrol	2008-2010	3	0	0	0	0
Italy	Syracuse	1999-2002, 2008-2012	9	0	0	0	0
Italy	Taranto	2008-2011	4	0	0	0	0
Italy	Torino	1985-2002, 2015	19	2015	1	2015	1
Italy	Trento	2008-2010	3	2013	1	0	0
Italy	Trieste	1984-1985, 1989-1992	6	0	0	0	0
Italy	Turin	2008-2012	5	0	0	0	0
Italy	Tuscany	1985-1997, 2013-2015	16	2013	1	0	0
Italy	Umbria	1994-1996, 1998-2006, 2008-2011	16	2000-2005, 2013	7	2004	1
Italy	Varese	1976-2000, 2008-2012	26	0	0	0	0
Italy	Veneto	1988-1996, 1998-2001, 2008-2010	16	2013	1	0	0
Italy	Verbano-Cusio-Ossola	2015	1	2015	1	2015	1
Italy	Vercelli	2015	1	2015	1	2015	1
Jamaica	Kingston and St Andrew	1958-1977, 1982-2012	31	0	0	0	0
Jamaica	National Registry	1978-1982, 1988-1997	13	0	0	0	0
Japan	Aichi	1998-2012, 2015	16	2006, 2015	2	2006, 2015	2
Japan	Fukui	1998-2012	15	0	0	0	0
Japan	Fukuoka	1974-1975	0	0	0	0	0
Japan	Hiroshima	1978-2012	33	0	0	0	0
Japan	Kanagawa	1980-1989	10	0	0	0	0
Japan	Miyagi	1959-1960, 1962-1964, 1968-1971, 1973-2010	31	0	0	0	0
Japan	Nagasaki	1973-2012	33	0	0	0	0
Japan	National Registry	1975-2010	31	1958-2017	38	1975-2010	31
Japan	Niigata	2003-2012	10	0	0	0	0
Japan	Okayama	1966, 1969	0	0	0	0	0
Japan	Osaka	1963-2012	33	0	0	0	0
Japan	Saga	1984-1986, 1988-1997, 2003-2007	18	0	0	0	0
Japan	Tochigi	2005-2012	8	0	0	0	0
Japan	Yamagata	1983-2013	31	0	0	0	0
Jordan	National Registry	1996-1998, 2000-2013	16	0	0	0	0
Kenya	Eldoret	2000-2011	12	0	0	0	0
Kenya	Eldoret Cancer Registry	2008-2016	9	0	0	0	0
Kenya	Nairobi	2000-2002, 2004-2014	14	0	0	0	0

Kuwait	National Registry	1979-2012	33	0	0	0	0
Kyrgyzstan	National Registry	1986-1987	2	0	0	0	0
La Martinique	La Martinique	1981-1987, 1993-2002	17	0	0	0	0
La Reunion	La Reunion	1988-1994, 2002-2011	17	0	0	0	0
Latvia	National Registry	1983-2007, 2010-2012	27	0	0	0	0
Lebanon	National Registry	1998, 2003-2012, 2015	12	0	0	0	0
Libya	Benghazi	2003-2008	6	0	0	0	0
Lithuania	Alytus	2008-2015	8	2008-2015	8	0	0
Lithuania	Kaunas	2008-2012	5	2008-2012	5	0	0
Lithuania	Klaipeda	2008-2015	8	2008-2015	8	0	0
Lithuania	Marijampole	2008-2012	5	2008-2012	5	0	0
Lithuania	National Registry	1978-2012	33	2006-2011	6	2006-2011	6
Lithuania	Panevezys	2008-2015	8	2008-2015	8	0	0
Lithuania	Siauliai	2008-2015	8	2008-2015	8	0	0
Lithuania	Taurage	2008-2015	8	2008-2015	8	0	0
Lithuania	Telsiai	2008-2015	8	2008-2015	8	0	0
Lithuania	Utena	2008-2015	8	2008-2015	8	0	0
Lithuania	Vilnius	2008-2015	8	2008-2015	8	0	0
Malawi	Blantyre	1991-1998, 2003-2010	16	0	0	0	0
Malaysia	National Registry	2003	1	0	0	0	0
Malaysia	Penang	1998-2002, 2004-2010	12	0	0	0	0
Malaysia	Sarawak	1998-2002	5	0	0	0	0
Mali	Bamako	1987-1996, 2005-2017	23	0	0	0	0
Mali	Mali	1995-2004	10	0	0	0	0
Malta	National Registry	1969-1972, 1989-2015	27	1995-2012, 2014-2015	19	1995, 1997-1998, 2000-2012	16
Mauritius	National Registry	2001-2015	15	0	0	0	0
Mexico	Mexico City Paediatric	1997-2013	17	0	0	0	0
Morocco	2 Registries Combined (Casablanca, Rabat)	2005-2012	8	0	0	0	0
Morocco	Greater Casablanca	2004-2007	4	0	0	0	0
Mozambique	Beira	2009-2010	2	0	0	0	0
Mozambique	Lourenco Marques	1956-1960	0	0	0	0	0
Mozambique	Maputo City Cancer Registry	2015-2017	3	0	0	0	0
Mozambique	Registro de Cancro de Beira	2009-2017	9	0	0	0	0
Namibia	Namibia Pediatric	1983-1992	10	0	0	0	0
Namibia	National Registry	1983-1988, 2000-2015	22	0	0	0	0
Namibia	Windhoek State Hospital	2003-2010	8	0	0	0	0
Nepal	Nepal Hospitals	2010-2013	4	0	0	0	0
Netherland Antilles excluding Aruba	Antilles except Aruba	1968-1982	3	0	0	0	0
Netherlands	Eindhoven	1973-2007	28	0	0	0	0
Netherlands	Maastricht	1986-2002	17	0	0	0	0
Netherlands	National Registry	1989-2018	30	1989-2017	29	1989-2017	29
Netherlands	Three Provinces Combined (Eindhoven, Maastricht, Antilles)	1960-1962	0	0	0	0	0
New Zealand	National Registry	1948-2017	38	1948-2015	36	1970-2015	36
New Zealand	New Zealand Maori population	1970-2016	37	1995-2017	23	1995-2015	21
New Zealand	New Zealand non-Maori population	1980-2016	37	1995-2017	23	1995-2015	21
Niger	National Registry	1992-2009	18	0	0	0	0
Niger	Niamey	2001-2009	9	0	0	0	0
Niger	Registre des Cancers du Niger	2006-2009, 2013-2017	9	0	0	0	0
Nigeria	Abuja Cancer Registry	2013-2016	4	0	0	0	0
Nigeria	Benin Pediatric	2010-2016	7	0	0	0	0

Nigeria	Calabar	2009-2017	9	0	0	0	0
Nigeria	Ekiti Cancer Registry	2013-2017	5	0	0	0	0
Nigeria	Ekiti Pediatric	2014-2015	2	0	0	0	0
Nigeria	Enugu Pediatric	2006-2009	4	0	0	0	0
Nigeria	Ibadan	1960-1969, 1985-1992, 2003-2012, 2015-2017	21	0	0	0	0
Nigeria	Maiduguri Pediatric	2006-2008, 2016	4	0	0	0	0
Nigeria	Midwestern Nigeria	2008-2009	2	2008-2009	2	0	0
Nigeria	Sokoto Pediatric	2013-2017	5	0	0	0	0
North Macedonia	North Macedonia Cancer Registry	2020	1	2020	1	2020	1
Norway	Agder	1953-2015	36	0	0	1980-2015	36
Norway	Innlandet	1953-2015	36	0	0	1980-2015	36
Norway	More og Romsdal	1953-2015	36	0	0	1980-2015	36
Norway	National Registry	1953-2017	38	1970-2014, 2016	36	1980-2014	35
Norway	Nordland	1953-2015	36	0	0	1980-2015	36
Norway	Oslo	1953-2015	36	0	0	1980-2015	36
Norway	Rogaland	1953-2015	36	0	0	0	0
Norway	Troms og Finnmark	1953-2015	36	0	0	1980-2015	36
Norway	Trondelag	1953-2015	36	0	0	1980-2015	36
Norway	Vestfold og Telemark	1953-2015	36	0	0	1980-2015	36
Norway	Vestland	1953-2015	36	0	0	1980-2015	36
Norway	Viken	1953-2015	36	0	0	1980-2015	36
Oman	National Registry	1993-2013, 2015	22	0	0	0	0
Pakistan	Karachi	1995-2002	8	0	0	0	0
Pakistan	Lahore	2008-2012	5	0	0	0	0
Pakistan	SKMCH & RC	2004-2006	3	0	0	0	0
Pakistan	South Karachi	1995-2002	8	0	0	0	0
Palestine	West Bank	1998-2007, 2010-2011	12	0	0	0	0
Panama	National Registry	1988-2011	24	1999-2011	13	1999	1
Paraguay	Asuncion Region	1988-1989	2	0	0	0	0
Peru	Arequipa	2008-2018	11	0	0	0	0
Peru	Lima	1990-1991, 2010-2012	5	2010-2012	3	0	0
Peru	Trujillo	1984-2002	19	1996-2002	7	1999	1
Philippines	Manila	1983-2012	30	0	0	0	0
Philippines	Rizal	1978-2012	33	0	0	0	0
Poland	Cieszyn	1973-1977	0	0	0	0	0
Poland	Cieszyn and Nowy Sacz	1968-1972	0	0	0	0	0
Poland	Cracow	1973-2002	23	0	0	0	0
Poland	Cracow City and District	1965-1966, 1968-1972	0	0	0	0	0
Poland	Four Rural Areas	1965-1966	0	0	0	0	0
Poland	Greater Poland	2008-2012	5	0	0	0	0
Poland	Katowice	1965-1966, 1970-1974	0	0	0	0	0
Poland	Kielce	1988-2002, 2008-2012	20	0	0	0	0
Poland	Lower Silesia	1984-1997, 2001-2014	28	0	0	0	0
Poland	Lublin	2008-2012	5	0	0	0	0
Poland	National Registry	1980-1989, 1999-2013, 2016	26	1999-2012, 2016	15	1999-2012	14
Poland	Nowy Sacz	1973-1981, 1983-1986	6	0	0	0	0
Poland	Opole	1985-1987	3	0	0	0	0
Poland	Podkarpackie	2008-2012	5	0	0	0	0
Poland	Warsaw	1988-2002	15	0	0	0	0
Poland	Warsaw Rural	1968-1977, 1983-1987	5	0	0	0	0
Poland	Warsaw Urban	1965-1966, 1968-1977, 1980-2002	23	0	0	0	0
Poland	Wroclaw City	2001-2014	14	0	0	0	0
Portugal	Azores	1997-2011	9	0	0	0	0
Portugal	National Registry	1989-2012	24	0	0	0	0
Portugal	North Portugal	1991-2012	22	1991-2012	22	1991-2012	22

Portugal	Porto	1998-2002	5	0	0	0	0
Portugal	South Portugal	1998-2007	3	0	0	0	0
Portugal	Vila Nova de Gaia	1983-1987, 1993-1997	10	0	0	0	0
Qatar	National Registry	2002-2014	13	0	0	0	0
Republic of Korea	Busan	1996-2012	17	0	0	0	0
Republic of Korea	Daegu	1997-2012	16	0	0	0	0
Republic of Korea	Daejeon	1998-2014	17	0	0	0	0
Republic of Korea	Gwangju	1998-2012	15	0	0	0	0
Republic of Korea	Incheon	1998-2012	15	0	0	0	0
Republic of Korea	Jejudo	2000-2002, 2004-2012	12	0	0	0	0
Republic of Korea	Kangwha County	1986-1997	12	0	0	0	0
Republic of Korea	National Registry	1999-2014	16	0	0	0	0
Republic of Korea	Seoul	1992-2012	21	0	0	0	0
Republic of Korea	Ulsan	1999-2012	14	0	0	0	0
Romania	Banat Region	1967	0	0	0	0	0
Romania	County Cluj	1974-1987	8	0	0	0	0
Romania	County Timis	1970-1972	0	0	0	0	0
Russian Federation	Altai kray	2007-2016	10	2007-2016	10	2007-2016	10
Russian Federation	Amur oblast	2007-2016	10	2007-2016	10	2007-2016	10
Russian Federation	Arkhangelsk oblast without Nenets autonomous district	1993-2001, 2007-2015	18	2007-2015	9	2007-2015	9
Russian Federation	Astrakhan oblast	2007-2016	10	2007-2016	10	2007-2016	10
Russian Federation	Belgorod oblast	2007-2016	10	2007-2016	10	2007-2016	10
Russian Federation	Bryansk oblast	2007-2016	10	2007-2016	10	2007-2016	10
Russian Federation	Chechen Republic	2007-2016	10	2007-2016	10	2007-2016	10
Russian Federation	Chelyabinsk oblast	2007-2016	10	2007-2016	10	2007-2016	10
Russian Federation	Chukchi autonomous area	2007-2016	10	2007-2016	10	2007-2016	10
Russian Federation	Chuvash Republic	2007-2016	10	2007-2016	10	2007-2016	10
Russian Federation	Irkutsk oblast	2007-2016	10	2007-2016	10	2007-2016	10
Russian Federation	Ivanovo oblast	2007-2016	10	2007-2016	10	2007-2016	10
Russian Federation	Jewish autonomous oblast	2007-2016	10	2007-2016	10	2007-2016	10
Russian Federation	Kabardian-Balkar Republic	2007-2016	10	2007-2016	10	2007-2016	10
Russian Federation	Kaliningrad oblast	2007-2016	10	2007-2016	10	2007-2016	10
Russian Federation	Kaluga oblast	2007-2016	10	2007-2016	10	2007-2016	10
Russian Federation	Kamchatka kray	2007-2016	10	2007-2016	10	2007-2016	10
Russian Federation	Karachaev-Chercassian Republic	2007-2016	10	2007-2016	10	2007-2016	10
Russian Federation	Kemerovo oblast	2007-2016	10	2007-2016	10	2007-2016	10
Russian Federation	Khabarovsk kray	2007-2016	10	2007-2016	10	2007-2016	10
Russian Federation	Khanty-Mansi autonomous area	2011-2016	6	2011-2016	6	2011-2016	6

Russian Federation	Kirov oblast	2007-2016	10	2007-2016	10	2007-2016	10
Russian Federation	Komi Republic	2007-2016	10	2007-2016	10	2007-2016	10
Russian Federation	Kostroma oblast	2007-2016	10	2007-2016	10	2007-2016	10
Russian Federation	Krasnodar kray	2007-2016	10	2007-2016	10	2007-2016	10
Russian Federation	Krasnoyarsk kray	2007-2016	10	2007-2016	10	2007-2016	10
Russian Federation	Kurgan oblast	2007-2016	10	2007-2016	10	2007-2016	10
Russian Federation	Kursk oblast	2007-2016	10	2007-2016	10	2007-2016	10
Russian Federation	Leningrad oblast	2007-2016	10	2007-2016	10	2007-2016	10
Russian Federation	Lipetzk oblast	2007-2016	10	2007-2016	10	2007-2016	10
Russian Federation	Magadan oblast	2007-2016	10	2007-2016	10	2007-2016	10
Russian Federation	Moscow City	2007-2016	10	2007-2016	10	2007-2016	10
Russian Federation	Moscow Region Paediatric	2000-2009	10	0	0	0	0
Russian Federation	Moscow oblast	2007-2016	10	2007-2016	10	2007-2016	10
Russian Federation	Murmansk oblast	2007-2016	10	2007-2016	10	2007-2016	10
Russian Federation	National Registry	2017	1	2017	1	2017	1
Russian Federation	Nenets autonomous district	2016	1	2016	1	2016	1
Russian Federation	Nizhny Novgorod oblast	2007-2016	10	2007-2016	10	2007-2016	10
Russian Federation	Novgorod oblast	2007-2016	10	2007-2016	10	2007-2016	10
Russian Federation	Novosibirsk oblast	2007-2016	10	2007-2016	10	2007-2016	10
Russian Federation	Omsk oblast	2007-2016	10	2007-2016	10	2007-2016	10
Russian Federation	Orenburg oblast	2007-2016	10	2007-2016	10	2007-2016	10
Russian Federation	Oryol oblast	2007-2016	10	2007-2016	10	2007-2016	10
Russian Federation	Penza oblast	2007-2016	10	2007-2016	10	2007-2016	10
Russian Federation	Perm kray	2007-2016	10	2007-2016	10	2007-2016	10
Russian Federation	Primorsky kray	2007-2016	10	2007-2016	10	2007-2016	10
Russian Federation	Pskov oblast	2007-2016	10	2007-2016	10	2007-2016	10
Russian Federation	Republic of Adygeya	2007-2016	10	2007-2016	10	2007-2016	10
Russian Federation	Republic of Altai	2007-2016	10	2007-2016	10	2007-2016	10
Russian Federation	Republic of Bashkortostan	2007-2016	10	2007-2016	10	2007-2016	10
Russian Federation	Republic of Buryatia	2007-2016	10	2007-2016	10	2007-2016	10
Russian Federation	Republic of Dagestan	2007-2016	10	2007-2016	10	2007-2016	10
Russian Federation	Republic of Ingushetia	2007-2016	10	2007-2016	10	2007-2016	10
Russian Federation	Republic of Kalmykia	2007-2016	10	2007-2016	10	2007-2016	10
Russian Federation	Republic of Karelia	2007-2016	10	2007-2016	10	2007-2016	10
Russian Federation	Republic of Khakasia	2007-2016	10	2007-2016	10	2007-2016	10

Russian Federation	Republic of Mariy El	2007-2016	10	2007-2016	10	2007-2016	10
Russian Federation	Republic of Mordovia	2007-2016	10	2007-2016	10	2007-2016	10
Russian Federation	Republic of North Ossetia-Alania	2007-2016	10	2007-2016	10	2007-2016	10
Russian Federation	Republic of Sakha (Yakutia)	2007-2016	10	2007-2016	10	2007-2016	10
Russian Federation	Republic of Tatarstan	2007-2016	10	2007-2016	10	2007-2016	10
Russian Federation	Republic of Tuva	2007-2016	10	2007-2016	10	2007-2016	10
Russian Federation	Rostov oblast	2007-2016	10	2007-2016	10	2007-2016	10
Russian Federation	Ryazan oblast	2007-2016	10	2007-2016	10	2007-2016	10
Russian Federation	Sakhalin oblast	2007-2016	10	2007-2016	10	2007-2016	10
Russian Federation	Samara oblast	1998-2016	19	2007-2016	10	2007-2016	10
Russian Federation	Sankt-Petersburg	2007-2016	10	2007-2016	10	2007-2016	10
Russian Federation	Saratov oblast	2007-2016	10	2007-2016	10	2007-2016	10
Russian Federation	Smolensk oblast	2007-2016	10	2007-2016	10	2007-2016	10
Russian Federation	St Petersburg	1983-1987, 1994-2002	14	0	0	0	0
Russian Federation	Stavropol kray	2007-2016	10	2007-2016	10	2007-2016	10
Russian Federation	Sverdlovsk oblast	2007-2016	10	2007-2016	10	2007-2016	10
Russian Federation	Tambov oblast	2007-2016	10	2007-2016	10	2007-2016	10
Russian Federation	Tomsk oblast	2007-2016	10	2007-2016	10	2007-2016	10
Russian Federation	Tula oblast	2007-2016	10	2007-2016	10	2007-2016	10
Russian Federation	Tver oblast	2007-2016	10	2007-2016	10	2007-2016	10
Russian Federation	Tyumen oblast without autonomous areas	2011-2015	5	2011-2015	5	2011-2015	5
Russian Federation	Udmurt Republic	2007-2016	10	2007-2016	10	2007-2016	10
Russian Federation	Ulyanovsk oblast	2007-2016	10	2007-2016	10	2007-2016	10
Russian Federation	Vladimir oblast	2007-2016	10	2007-2016	10	2007-2016	10
Russian Federation	Volgograd oblast	2007-2016	10	2007-2016	10	2007-2016	10
Russian Federation	Vologda oblast	2007-2016	10	2007-2016	10	2007-2016	10
Russian Federation	Voronezh oblast	2007-2016	10	2007-2016	10	2007-2016	10
Russian Federation	Yamalo-Nenets autonomous area	2011-2016	6	2011-2016	6	2011-2016	6
Russian Federation	Yaroslavl oblast	2007-2016	10	2007-2016	10	2007-2016	10
Russian Federation	Zabaikalsk kray	2007-2016	10	2007-2016	10	2007-2016	10
Rwanda	Butare	1991-1993	3	0	0	0	0
Samoa	National Registry	1980-1988	9	0	0	0	0
Saudi Arabia	National Registry	1994-1996, 2006-2012, 2015	11	0	0	2006-2012	7
Saudi Arabia	Riyadh	1994-2012	19	0	0	0	0
Senegal	Dakar	1969-1974	0	0	0	0	0
Serbia	Central Serbia	2003-2007, 2019-2020	7	2003-2007, 2019-2020	2	2019-2020	2
Serbia	National Registry	1999-2002, 2008-2013	10	2008-2013	6	2008-2013	6
Serbia	Vojvodina	1988-1997	10	0	0	0	0

Seychelles	National Registry	2009-2017	9	0	0	0	0
Singapore	National Registry	1950-1961, 1968-2015	36	0	0	0	0
Singapore	SG-CCSS	1981-2005	25	0	0	0	0
Slovakia	National Registry	1968-2010	31	0	0	0	0
Slovenia	National Registry	1956-2016	37	1985-2017	33	1985-2016	32
South Africa	Eastern Cape	2003-2016	14	0	0	0	0
South Africa	Johannesburg, Bantu	1953-1955	0	0	0	0	0
South Africa	National Registry	2003-2005, 2007, 2010-2014	9	0	0	0	0
South Africa	Eastern Cape Province Cancer Registry by the Programme on Mycotoxin and Experimental Carcinogenesis (PROMEC)	1998-2012	15	0	0	0	0
South Africa	South Africa Paediatric	1988-1991, 1998-2012	19	0	0	0	0
South Africa	South African Children Tumour Registry	1987-2007	21	0	0	0	0
South Africa	South African Children's Cancer Study Group	2008-2012	5	0	0	0	0
Spain	11 Registries Combined (Albacete, Asturias, Balearic Islands, Basque Country, Canary Islands, Cuenca, Girona, Granada, Murcia, Navarra, Tarragona)	1990-2013	24	0	0	0	0
Spain	Albacete	1991-2010	19	0	0	0	0
Spain	Asturias	1982-2000, 2003-2010	27	0	0	0	0
Spain	Basque	1986-1994	9	0	0	0	0
Spain	Basque Country	1986-2012	20	0	0	0	0
Spain	Canary Islands	1993-1995, 1997-2001, 2003-2013	19	0	0	0	0
Spain	Castellon	2008-2012	5	0	0	0	0
Spain	Ciudad Real	2004-2011	8	0	0	0	0
Spain	Cuenca	1993-2011	19	0	0	0	0
Spain	Girona	1980-2012	18	0	0	0	0
Spain	Granada	1985-2013	29	1985-2007, 2011-2013	3	0	0
Spain	La Rioja	1993-2012	10	0	0	0	0
Spain	Mallorca	1988-2000, 2003-2012	23	1993-2000, 2009-2012	12	1994, 1998, 2010	3
Spain	Murcia	1983-2010	27	0	0	0	0
Spain	National Registry	1980-1991	12	0	0	0	0
Spain	Navarra	1973-2010	31	0	0	0	0
Spain	Registro Poblacional de Tumores Infanties de Castilla y Leon	2005, 2010-2013	5	0	0	0	0
Spain	Spanish Registry of Childhood Tumours	1990-1995	6	0	0	0	0
Spain	Tarragona	1980-2012	32	0	0	0	0
Spain	Valencia Paediatric	1983-2010	28	0	0	0	0
Spain	Zaragoza	1968-2000	21	0	0	0	0
Sri Lanka	National Registry	2006-2011, 2014	5	0	0	0	0
Sweden	National Registry	1958-2014	35	1970-2014	35	1980-2014	35
Sweden	Stockholm	2002-2016	15	0	0	0	0
Sweden	Sweden except Stockholm	2002-2016	15	0	0	0	0
Switzerland	7 Registries Combined (Fribourg, Geneva, Neuchatel, Ticino, Valais, Vaud, Zurich)	1990-2013	24	0	0	0	0
Switzerland	Basel	1981-1997, 2003-2007	22	0	0	0	4
Switzerland	Bern	2015	1	0	0	0	1

Switzerland	Fribourg	2008-2012	5	0	0	2008-2009	4
Switzerland	Geneva	1970-2012	33	0	0	1995-2012	19
Switzerland	Graubunden	1989-1997	9	0	0	0	0
Switzerland	Graubunden and Glarus	1989-2012	15	0	0	1998-2012	19
Switzerland	National Registry	1980-2013	13	0	0	0	0
Switzerland	Neuchatel	1974-1976, 1978-1996, 1998-2012	32	0	0	0	0
Switzerland	St Gallen - Appenzell	1980-2012	30	0	0	1995-2012	19
Switzerland	Switzerland Paediatric	1985-2017	33	1986-2015	30	1986-2015	30
Switzerland	Ticino	1996-2012	17	0	0	2000-2012	13
Switzerland	Valais	1989-2012	24	0	0	1995-2012	20
Switzerland	Vaud	1975-1996, 1998-2012	32	0	0	0	0
Switzerland	Zurich	1980-2012	27	0	0	1995-1996, 2003-2012	19
Taiwan*	National Registry	1980-2016	37	1980-2007	28	1980-2007	28
Thailand	4 Registries Combined (Bangkok, Chiang Mai, Khon Kaen, Songkhla)	1983-1993	11	0	0	0	0
Thailand	Bangkok	1995-1997, 2001-2012	15	0	0	0	0
Thailand	Chiang Mai	1983-2012	30	0	0	0	0
Thailand	Chonburi	1998-2012	15	0	0	0	0
Thailand	Khon Kaen	1985-2012	25	0	0	0	0
Thailand	Lampang	1993-2013	21	0	0	0	0
Thailand	Lop Buri	2001-2003	3	0	0	0	0
Thailand	Lopburi Province	2009-2012	4	0	0	0	0
Thailand	Nakhon Phanom	2001-2003	3	0	0	0	0
Thailand	Prachuap Khiri	2001-2003	3	0	0	0	0
Thailand	Rayong	2001-2003	3	0	0	0	0
Thailand	Songkhla	1993-2013	21	0	0	0	0
Thailand	Surat Thani	2001-2003	3	0	0	0	0
Thailand	Thai Cancer Registry	1995-1997	3	0	0	0	0
Thailand	Thai Pediatric Oncology Group	2003-2005	3	0	0	0	0
Thailand	Ubon Ratchathani	2001-2003	3	0	0	0	0
Thailand	Udon Thani	2001-2003	3	0	0	0	0
Trinidad and Tobago	National Registry	1995-2006	12	1995-2006	12	1995-2006	12
Tunisia	Center of Tunisia	1993-2003	11	0	0	0	0
Tunisia	Central Tunisia	1993-2007	15	0	0	0	0
Tunisia	Centre Sousse	1998-2002	5	0	0	0	0
Tunisia	North Tunisia	1994-2009	16	0	0	0	0
Türkiye	8 Registries Combined (Ankara, Antalya, Bursa, Edirne, Erzurum, Izmir, Samsun, Trabzon)	1992-2012	21	0	0	0	0
Türkiye	Ankara	2002-2005	4	0	0	0	0
Türkiye	Antalya	1998-2012	15	0	0	0	0
Türkiye	Bursa	2008-2012	5	0	0	0	0
Türkiye	Edirne	2002-2012	11	0	0	0	0
Türkiye	Eight Provinces	2007	1	0	0	0	0
Türkiye	Erzurum	2002-2003, 2005, 2010-2012	6	0	0	0	0
Türkiye	Eskisehir	2002-2005, 2008-2012	9	0	0	0	0
Türkiye	Izmir	1993-1996, 1998-2012	19	0	0	0	0
Türkiye	Nine Provinces Combined (Ankara, Antalya, Bursa, Edirne, Erzurum, Eskisehir, Izmir, Samsun, Trabzon)	2008-2014	7	0	0	0	0
Türkiye	Samsun	2002-2005, 2008-2012	9	0	0	0	0
Türkiye	Trabzon	2002-2012	11	0	0	0	0
Uganda	Gulu Cancer Registry	2013-2015	3	0	0	0	0

Uganda	Kampala	1954-1960, 1990-2013	24	0	0	0	0
Ukraine	National Registry	2002-2012	11	0	0	0	0
Ukraine	National Registry (without Crimea & Sevastopol)	2000-2012	13	2003, 2005, 2009-2010	2	2009-2010	2
Ukraine	Republic of Crimea	2014-2016	3	2015-2016	2	2015-2016	2
Ukraine	Sevastopol	2014-2016	3	2015-2016	2	2015-2016	2
United Kingdom	Aryshire	1970-1972	0	0	0	0	0
United Kingdom	British National Registry of Childhood Tumours	1981-1990	10	0	0	0	0
United Kingdom	East Anglia	1988-1997	10	0	0	0	0
United Kingdom	East Midlands	1990-2014	25	1981-2012	31	1990-1994, 2001-2009, 2011-2012	16
United Kingdom	East Scotland	1973-1987	8	0	0	0	0
United Kingdom	East of England	1990-2014	25	1981-2012	31	1990-1994, 2001-2009, 2011-2012	16
United Kingdom	England	1971-2016	37	0	0	0	0
United Kingdom	England and Wales	1979-1990	11	0	0	0	0
United Kingdom	Greater London	1990-2014	25	1981-2012	30	1990-1994, 2001-2009, 2011-2012	16
United Kingdom	Hackney & City of London	2015-2017	3	0	0	0	0
United Kingdom	Merseyside and Cheshire	1959-1966, 1968-1972, 1975-2002	15	0	0	0	0
United Kingdom	National Registry	2008-2012	5	0	0	0	0
United Kingdom	North East England	1990-2014	25	1981-2012	31	1990-1994, 2001-2009, 2011-2012	16
United Kingdom	North East Scotland	1973-1987	8	0	0	0	0
United Kingdom	North Scotland	1973-1987	8	0	0	0	0
United Kingdom	North West England	1973-1977, 1979-2014	35	1981-2012	22	1990-1994, 2001-2009, 2011-2012	16
United Kingdom	Northern England and Yorkshire	1998-2002, 2008-2012	10	0	0	0	0
United Kingdom	Northern Ireland	1993-2013	20	0	0	0	0
United Kingdom	Northern Region	1968-1982	3	0	0	0	0
United Kingdom	Oxford	1963-1966, 1968-1972, 1974-1977, 1979-2007	28	0	0	0	0
United Kingdom	Scotland	1963-1966, 1975-2017	38	1974-2017	38	1980-2009, 2011-2017	37
United Kingdom	South East England	1990-2014	25	1981-2012	31	1990-1994, 2001-2009, 2011-2012	16
United Kingdom	South East Scotland	1973-1987	8	0	0	0	0
United Kingdom	South Thames	1960-1997	18	0	0	0	0
United Kingdom	South West England	1960-1970, 1979-2014	33	1981-2012	29	1991-1994, 2001-2009, 2011-2012	15
United Kingdom	St. Helens	2015-2017	3	0	0	0	0
United Kingdom	Thames	1998-2007	5	0	0	0	0
United Kingdom	Trent	1963-1970, 1974-1976, 1979-1987, 1993-2002, 2005-2007	13	0	0	0	0
United Kingdom	Wales	2000-2012	13	1981-2012	32	2005, 2010	2
United Kingdom	West Midlands	1960-1966, 1968-1976, 1979-2014	35	1981-2012	31	1981-1994, 2001-2009, 2011-2012	25
United Kingdom	West Scotland	1975-1992	13	0	0	0	0
United Kingdom	Yorkshire	1983-2002	20	0	0	0	0
United Kingdom	Yorkshire and the Humber	1990-2014	25	1981-2012	32	1990-1994, 2001-2012	17
United Republic of Tanzania	Kilimanjaro Cancer Registry	2013-2017	5	0	0	0	0
United Republic of Tanzania	Mwanza Cancer Registry	2016-2017	2	0	0	0	0
United States of America	Alabama	1998-2012	15	0	0	0	0
United States of America	Alameda County	1983-1987	5	0	0	0	0
United States of America	Alaska	1992-2013	22	1992-2009	18	1992-2008	17

United States of America	Arizona	1998-2012	15	0	0	0	0
United States of America	Arkansas	1998-2012	15	0	0	0	0
United States of America	Atlanta	1973-2016	37	1973-2012	33	1974-2008, 2010	30
United States of America	California	1998-2012	15	0	0	0	0
United States of America	California except San Francisco, San Jose - Monterey & Los Angeles	2000-2016	17	2000-2009	10	2000-2008	9
United States of America	Central California	1988-1992	5	0	0	0	0
United States of America	Colorado	1998-2012	15	0	0	0	0
United States of America	Connecticut	1960-1962, 1973-2016	37	1973-2012	33	1973-2008, 2010	30
United States of America	Delaware	1998-2012	15	0	0	0	0
United States of America	Detroit	1973-2016	37	1973-2012	33	1973-2008, 2010	30
United States of America	District of Columbia	1998-2012	15	0	0	0	0
United States of America	El Paso	1960-1966, 1968-1970	0	0	0	0	0
United States of America	Florida	1995-2012	18	1995-1999	5	1995-1999	5
United States of America	Georgia	1998-2016	19	2010-2016	7	2012-2014	3
United States of America	Greater Delaware Valley Pediatric Tumor Registry	1980-1989	10	0	0	0	0
United States of America	Greater Georgia	1973-2016	37	1973-2012	33	1974, 1977, 1980, 1983, 1986, 1989, 1992, 1995, 1998, 2001, 2004, 2007, 2010	11
United States of America	Hawaii	1960-1964, 1968-2016	37	1973-2012	33	1973-2008, 2010	30
United States of America	Hawaiian	1973-1992	13	0	0	0	0
United States of America	Idaho	1998-2012	15	0	0	0	0
United States of America	Illinois	1998-2012	15	0	0	0	0
United States of America	Indiana	1998-2012	15	0	0	0	0
United States of America	Iowa	1973-2016	37	1973-2012	33	1973-2008, 2010	30
United States of America	Kentucky	1973-2016	37	1973-2012	33	1974, 1977, 1980, 1983, 1986, 1989, 1992, 1995, 1998, 2000-2008, 2010	17
United States of America	Los Angeles	1973-2016	37	1973-2012	33	1974, 1977, 1980, 1983, 1986, 1989, 1992-2008, 2010	22
United States of America	Louisiana	1973-2016	37	1973-2012	33	1974, 1977, 1980, 1983, 1986, 1989, 1992, 1995, 1998, 2000-2004, 2006-2008, 2010	16
United States of America	Maine	1998-2012	15	0	0	0	0
United States of America	Maryland	1998-2012	15	0	0	0	0
United States of America	Massachusetts	1998-2012	15	0	0	0	0
United States of America	Michigan	1998-2012	15	0	0	0	0

United States of America	Minnesota	1998-2012	15	0	0	0	0
United States of America	Mississippi	2002-2012	11	0	0	0	0
United States of America	Missouri	1996-2012	17	0	0	0	0
United States of America	Montana	1998-2012	15	0	0	0	0
United States of America	National Program of Cancer Registries (NPCR)	1998-2002, 2008-2012	10	0	0	0	0
United States of America	National Registry	2008-2012	5	0	0	0	0
United States of America	National Registry	1962-1966, 1968-1977, 1980-2012	33	2008-2012	5	2010	1
United States of America	Nebraska	1998-2012	15	0	0	0	0
United States of America	Nevada	1959-1966, 1998-2012	15	0	0	0	0
United States of America	New Hampshire	1998-2012	15	0	0	0	0
United States of America	New Jersey	1973-2016	37	1973-2012	33	1974, 1977, 1980, 1983, 1986, 1989, 1992, 1995, 1998, 2000-2008, 2010	17
United States of America	New Mexico	1969-2016	37	1973-2012	33	1973-2008, 2010	30
United States of America	New Orleans	1983-2012	30	0	0	0	0
United States of America	New York	1983-1991, 1993-2012	29	0	0	0	0
United States of America	New York except New York City	1959-1961	0	0	0	0	0
United States of America	North Carolina	1998-2012	15	0	0	0	0
United States of America	North Dakota	1998-2012	15	0	0	0	0
United States of America	Ohio	1998-2012	15	0	0	0	0
United States of America	Oklahoma	1998-2012	15	0	0	0	0
United States of America	Oregon	1998-2012	15	2006-2010	5	0	0
United States of America	Pennsylvania	1998-2012	15	0	0	0	0
United States of America	Rhode Island	1998-2012	15	0	0	0	0
United States of America	Rural Georgia	1973-2016	37	1973-2012	33	1974, 1977, 1980, 1983, 1986, 1989, 1992-2008, 2010	22
United States of America	Surveillance, Epidemiology, and End Results Program (SEER)	1973-2019	35	1969-2019	25	1997, 2002, 2007, 2012, 2017	5
United States of America	San Francisco	1973-2012	33	0	0	0	0
United States of America	San Francisco, Oakland, San Mateo, and Surrounding Area	1973-2016	37	1973-2009	30	1973-2008	29
United States of America	San Jose Monterey	1973-2016	37	1973-2012	33	1974, 1977, 1980, 1983, 1986, 1989, 1992-2008, 2010	22
United States of America	Seattle	1973-2016	37	1973-2012	33	1974-2008, 2010	30
United States of America	South Carolina	1998-2012	15	0	0	0	0
United States of America	South Dakota	2001-2012	12	0	0	0	0
United States of America	Tennessee	1999-2012	14	0	0	0	0

United States of America	Texas	1998-2012	15	0	0	0	0
United States of America	Utah	1973-2016	37	1973-2012	33	1973-2008, 2010	30
United States of America	Vermont	1998-2012	15	0	0	0	0
United States of America	Virginia	1998-2012	15	0	0	0	0
United States of America	Washington	1998-2012	15	0	0	0	0
United States of America	West Virginia	1998-2012	15	0	0	0	0
United States of America	Wisconsin	1998-2012	15	0	0	0	0
United States of America	Wyoming	1998-2012	15	0	0	0	0
Uruguay	Montevideo	1990-1995	6	0	0	0	0
Uruguay	National Registry	1988-2014	27	2012-2016	5	0	0
Viet Nam	Hanoi	1991-1997	7	0	0	0	0
Viet Nam	Ho Chi Minh	1995-2013	19	0	0	0	0
Yemen	Aden	1997-2011	15	0	0	0	0
Yemen	Hadhramout	2002-2014	13	0	0	0	0
Zambia	Zambia National Cancer Registry	2011-2015	5	0	0	0	0
Zimbabwe	Bulawayo	1963-1972, 2011-2015	5	0	0	0	0
Zimbabwe	Harare	1990-2015	26	0	0	0	0
Zimbabwe	National Registry	2005-2006, 2011-2012	4	0	0	0	0

Abbreviations: MIR, mortality-to-incidence ratio. Cancer registry data are only used for estimating MIRs (column “Years used for MIRs”) if both incidence and mortality data are available, with the exception of Norway and Saudi Arabia where cancer registry incidence data was matched to vital registration mortality data to produce MIRs. While this manuscript reports estimates modeled from available data between 1980 - 2023, the columns reporting the years of data available can include years earlier than 1980 because earlier years of data were available for collection. Years used for MIRs may be lower than the years of data available for incidence and mortality separately if there are overlapping registries for a single year, if the incidence and mortality data represent year ranges rather than individual years of data, or if the MIR does not meet our inclusion criteria and is dropped from the model. *United Nations convention recognizes Taiwan as a province of China.

Mortality-to-incidence ratio data sources

Most population-based cancer registries only report cancer incidence. However, if a cancer registry also reported cancer mortality, mortality data were also extracted. Cancer registry sources with matching incidence and mortality data were used in the mortality-to-incidence ratio (MIR) estimation, as listed in Appendix 1 Table 5.

Cancer mortality data in the cause of death database other than cancer registry data

As shown in Appendix 1 Table 4, the GBD Cause of Death (CoD) database contains cancer mortality data originating from multiple sources in addition to cancer registries, including vital registration and verbal autopsy data. In countries without vital registration systems, verbal autopsy studies can be a viable data source to inform cause of death. Verbal autopsy data are obtained by trained interviewers who use a standardised questionnaire to ask relatives about the signs, symptoms, and demographic characteristics of recently deceased family members. Cause of death is assigned based on the answers to the questionnaires. A detailed description of the data sources and processing steps for the CoD database can be found in the appendix to the GBD 2023 paper, “Global burden of 292 causes of death in 204 countries and territories and 660 subnational locations, 1990–2023: a systematic analysis for the Global Burden of Disease Study 2023. Lancet (in review)”,[2] as well as in the online GBD citation tool https://ghdx.healthdata.org/gbd-2023 *(To the editors and reviewers: note that this URL is not yet live, but will be made available to the public prior to publication)*.

Bias of categories of input data

Cancer registry data can be biased in multiple ways. A high proportion of ill-defined cancer cases in the cancer registry data requires redistribution of these cases to other cancers, which introduces a potential for bias. Changes

between coding systems can lead to artificial differences in disease estimates. However, we adjust for this bias by mapping the different coding systems to GBD cancer causes (Appendix 1 Tables 1 and 2). Incomplete overlap of different coding systems and data sources can also lead to artificial shifts in the estimates over time, which we lessen through temporal smoothing. Underreporting of cancers that require advanced diagnostic techniques (e.g., leukaemia, brain, pancreatic, and liver cancer) can be an issue in cancer registries from lower-income countries. On the other hand, misclassification of metastatic sites as primary cancer can lead to overestimation of cancer sites that are common sites for metastases (e.g., brain or liver cancer). Since many cancer registries are located in urban areas, the representativeness of the registry for the general non-urban population can also be problematic. Because mortality input data go through a noise reduction procedure to lessen the impact of outliers, national input values and estimates can be shifted closer to regional averages. The accuracy of mortality data reported by a subset of cancer registries usually depends on the quality of the local vital registration system. If the vital registration system is incomplete or of poor quality, the mortality-to-incidence ratio can be biased to lower ratios (more information on mortality-to-incidence ratios can be found in sections *"8a MIR input data"* and *"8b MIR modelling"*, below). Additional potential biases of the input data included for the CoD database can be found in the appendix to the GBD 2023 paper "Global burden of 292 causes of death in 204 countries and territories and 660 subnational locations, 1990–2023: a systematic analysis for the Global Burden of Disease Study 2023. Lancet (in review)".[2]

Data analysis

Flowcharts describing the conceptual overview of the processing of cancer data are available in Appendix Figure 1 and Appendix Figure 2.

Cancer registry data processing

Cancer registry data goes through multiple processing steps before entering the CoD database.

1. Formatting incidence and mortality data. First, the original data are transformed into standardised files, which included standardisation of format, categorisation of cancers, and registry names (#1 in Appendix 1 Figure 1).

2. Subtotal recalculation. Some cancer registries report individual codes as well as aggregated totals. An example of this would be where the registry data report C18, C19, and C20 individually and also report the aggregated group of C18–C20 (colon and rectum cancer). The data processing step, "subtotal recalculation" (#2 in Appendix 1 Figure 1), verifies these totals and subtracts the values of any individual codes from the aggregates so that no cases and deaths are counted more than once between specific codes and aggregated groupings.

3. Mapping data to GBD causes. In the third step (#3 in Appendix 1 Figure 1), cancer registry incidence data and cancer registry mortality data are mapped to GBD causes. A different map is used for incidence and for mortality data because of different assumptions around misclassification of ICD 'D' prefix codes. For incidence data, these D codes are assumed to be correctly assigned to incident cases and are not mapped to a GBD cause because they are not malignant cancers. In contrast, for mortality data these D codes do get mapped to the GBD cause, because it is assumed that a death that was assigned a 'D' code was misclassified and should have been assigned the relevant 'C' code instead. The maps also differ because of the assumption that there are no deaths for certain cancers. One example is basal cell carcinoma of the skin. In the cancer registry incidence data, basal cell carcinoma is mapped to "non-melanoma skin cancer (basal cell carcinoma)". However, if basal cell skin cancer is recorded in the cancer registry mortality data, the deaths are instead mapped to "non-melanoma skin cancer (squamous cell carcinoma)" under the assumption that they were misclassified squamous cell skin cancers. Another example is benign or in situ neoplasms. Because cancer registries do not collect most non-malignant neoplasms in a standardised way, any benign or in situ neoplasms reported in a cancer registry incidence dataset are excluded. The same neoplasms reported in a cancer registry mortality dataset are instead mapped to the respective invasive cancer. For example, incident cases of "ductal carcinoma in situ" in a cancer registry incidence dataset are excluded, while deaths from "ductal carcinoma in situ" in a cancer registry mortality dataset are mapped to "breast cancer". The exception is for codes for benign tumours of the brain, which are mapped to "other benign and in situ neoplasms" rather than brain

cancer, as these non-invasive tumors can cause death through intracranial pressure. Maps of ICD codes to GBD causes for incidence and mortality data can be found in Appendix 1 Table 2 and Appendix 1 Table 3.

4. Age/sex splitting. In the fourth data processing step (#4 in Appendix 1 Figure 1), cancer registry data are standardised to the GBD age groups. For GBD 2023 these age groups were the same as for GBD 2021. For GBD 2021 and GBD 2023, these categories were 0–6 days (early neonatal), 7–27 days (late neonatal), 1–5 months, 6–11 months, 12–23 months, and 2–4 years. These are followed by 19 childhood, adolescent, and adult age categories binned by five years: 5–9 years, 10–14 years, etc., up to the terminal age group of 95 years and older (95+). The modelled starting and ending age groups for each cancer are reported in Appendix 1 Table 1.

Reference global age-specific incidence rates are generated using select comprehensive cancer registry datasets, such as from SEER,[13] NORDCAN,[26] CI5,[14–24] and the International Incidence of Childhood Cancer (IICC).[27] The use of high-quality population-based cancer registry data to generate these rates is the same as for GBD 2021, and a change from GBD 2019 where incidence age rates were based on hospital inpatient data. Reference age-specific mortality rates were generated using age weights from processed vital registration data, using the approach described in Section X of the appendix to the GBD 2023 paper "Global burden of 292 causes of death in 204 countries and territories and 660 subnational locations, 1990–2023: a systematic analysis for the Global Burden of Disease Study 2023. Lancet (in review)" *(To the editors and reviewers: note that section detail will be finalized once the GBD 2023 Causes of Death Collaborators publication is final.)*.[2] For incidence or mortality datasets that require age splitting, global age-specific proportions are then generated by applying the reference age-specific rates to the overall registry population to produce the expected number of cases (or deaths for a mortality dataset) for that registry by age. The expected number of cases (or deaths) for each sex, age, and cancer were normalised to 1, creating final, age-specific proportions. These proportions were then applied to the total number of cases (or deaths) by sex and cancer to get the GBD age group-specific number of cases (or deaths) related to that dataset.

In the rare case that the cancer registry only contains data for both sexes combined, the age-specific cases or deaths are split and reassigned to separate sexes using the same weights that are used for the age splitting process. Starting from the expected number of deaths, global proportions are generated by sex for each age. For example, if for ages 15–19 years old there are 6 expected deaths for males and 4 expected deaths for females from cause of death data, then 60% of the combined-sex deaths for ages 15–19 years would be assigned to males and the remaining 40% would be assigned to females.

5. Cause disaggregation. In the fifth step (#5 in Appendix 1 Figure 1), data for cause entries that are aggregates of GBD causes were redistributed across those GBD causes. Examples of these aggregated causes include some cancer registries reporting ICD-10 codes C00–C14 together as "lip, oral cavity, and pharyngeal cancer". These groups are broken down into sub-causes that can be individually mapped to single GBD causes. In this example, the more specific ICD-10 codes within C00–C14 are disaggregated into "lip and oral cavity cancer" (C00–C08), "nasopharynx cancer" (C11), "cancer of other parts of the pharynx" (C09–C10, C12–C13), and "malignant neoplasm of other and ill-defined sites in the lip, oral cavity, and pharynx" (C14). To redistribute the data, weights were created using the same "rate-applied-to-population" method employed in age/sex splitting (see step four above). For the undefined code (C14 in this example) an "average all cancer" weight was used, calculated on the high quality cancer registry data from SEER,[28] NORDCAN,[26] and CI5[14–24] by dividing the sum of the cases across these registries by the combined population across these registries. Then, proportions were generated by sub-cause for each aggregate cause as in the sex splitting example above (see step four). The total number of cases from the aggregated group (C00–C14) was recalculated for each subgroup and the undefined code (C14). C14 was then redistributed as a "garbage code" in step six. For some exceptions, C44 (non-melanoma skin cancer), C46 (Kaposi sarcoma), and C74 (malignant neoplasm of adrenal gland), fixed proportions were used to redistribute into GBD causes. Non-melanoma skin cancer processing is described under section "Additional method summaries for non-melanoma skin cancer (squamous and basal cell carcinoma)". C46 entries were primarily redistributed to either HIV or to the GBD cause "soft tissue and other extraosseous sarcomas", with the redistribution proportion varying by GBD region (ranging from 7.7% to HIV in the South Asia region to 93.0% HIV in the Southern Sub-Saharan Africa region).[9] This proportion was an update from GBD 2021 where the proportion was based on age (100% to HIV for age <15 years, 95% for age 15–49 years, and 90% for age ≥50 years), and led to fewer C46 deaths being

redistributed to HIV than in previous GBD rounds. This is an additional change from GBD 2019, where the non-HIV redistribution target was “Other malignant neoplasms”. C74 entries were redistributed to “neuroblastoma and other peripheral nervous cell tumours” and “other malignant neoplasms” with percentages varying by age (younger age groups had a greater proportion redistributed to “neuroblastoma and other peripheral nervous cell tumours”).[28]

6. Redistribution. In the sixth step (#6 in in Appendix 1 Figure 1), unspecified ICD codes (“garbage codes”) such as “ill-defined cancer site” (for example, C76 or C80) are redistributed across relevant causes estimated within the GBD hierarchy. Redistribution of cancer registry incidence and mortality data mirrored the process of the redistribution used in the CoD database and used the same redistribution maps as specified in the appendix to the GBD 2023 paper, “Global burden of 292 causes of death in 204 countries and territories and 660 subnational locations, 1990–2023: a systematic analysis for the Global Burden of Disease Study 2023. Lancet (in review)”.[2] Sources and targets of garbage codes can be found in this Appendix 1 Table 6.

Appendix Table 6: Undefined cancer code categories (ICD-10) and respective target codes for cancer registry incidence data

Unspecified site cancer codes	Target codes for redistribution of these unspecified site cancers
C14, C14.0-14.3, C14.8-14.9	C00-13, C00.0-00.6, C00.8-01.0, C01.9-02.4, C02.8-03.1, C03.9-04.1, C04.8-05.2, C05.8-06.2, C06.8-07.0, C07.9-08.1, C08.8-09.1, C09.8-10.4, C10.8-11.3, C11.8-12.0, C12.9-13.2, C13.8-13.9, C43.0, C44.0, D03.0, D04.0, D10.0-10.7, D11, D11.0, D11.7, D11.9, D22.0, D23.0
C26-29, C26.0-26.2, C26.8-26.9, C35-36	C02.0, C03.0, C04.0, C05.0, C07.0, C15-25, C15.0-15.5, C15.8-17.3, C17.8-19.0, C19.9-20.0, C20.8-21.2, C21.8-22.5, C22.7-22.8, C23.0, C23.9-24.1, C24.4, C24.8-25.4, C25.7-25.9, C30-31, C30.0-30.3, C30.5, C30.8-31.3, C31.8-31.9, C37-38, C37.0-37.3, C38.0-38.4, C38.8, C45, C45.0-45.9, C48, C48.0-48.2, C48.8-48.9, C51-52, C51.0-51.2, C51.8-52.0, C52.9, C57, C57.0-57.4, C57.7-57.8, C60, C60.0-60.2, C60.8-60.9, C63, C63.0-63.2, C63.7-63.8, C66, C66.0-66.2, C66.9, C68.0-68.1, C68.8, C75, C75.0, C75.4-75.6, C75.8, D00.1-00.2, D01.0-01.3, D07.4, D09.2, D12, D12.0-13.7, D14.0, D15-16, D15.0-15.2, D15.7, D15.9-16.9, D28.0-28.1, D28.7, D29.0, D30.2, D30.4, D30.7-30.8, D31, D31.0-31.6, D31.9, D35-36, D35.0-35.1, D35.5-35.9, D36.1, D36.7, D37.1-37.5, D38.2-38.5, D39.2, D39.8, D41.2-41.3, D44.1-44.8, D48.0-48.4
C39, C39.0, C39.8-39.9	C03.0, C04.0, C05.0, C07.0, C17, C17.0-17.3, C17.8-17.9, C30-34, C30.0-30.3, C30.5, C30.8-31.3, C31.8-32.3, C32.8-33.0, C33.2, C33.9-34.4, C34.7-34.9, C37-38, C37.0-37.3, C38.0-38.4, C38.8, C45, C45.0-45.9, C48, C48.0-48.2, C48.8-48.9, C51-52, C51.0-51.2, C51.8-52.0, C52.9, C57, C57.0-57.4, C57.7-57.8, C60, C60.0-60.2, C60.8-60.9, C63, C63.0-63.2, C63.7-63.8, C66, C66.0-66.2, C66.9, C68.0-68.1, C68.8, C75, C75.0, C75.4-75.6, C75.8, D02.0-02.3, D07.4, D09.2, D13.2-13.3, D14.0-14.3, D15-16, D15.0-15.2, D15.7, D15.9-16.9, D28.0-28.1, D28.7, D29.0, D30.2, D30.4, D30.7-30.8, D31, D31.0-31.6, D31.9, D35-36, D35.0-35.1, D35.5-35.9, D36.1, D36.7, D37.2, D38.0-38.5, D39.2, D39.8, D41.2-41.3, D44.1-44.8, D48.0-48.4
C55, C55.0-55.1, C55.9	C03.0, C04.0, C05.0, C07.0, C17, C17.0-17.3, C17.8-17.9, C30-31, C30.0-30.3, C30.5, C30.8-31.3, C31.8-31.9, C37-38, C37.0-37.3, C38.0-38.4, C38.8, C48, C48.0-48.2, C48.8-48.9, C51-54, C51.0-51.2, C51.8-52.0, C52.9-53.1, C53.3-53.4, C53.8-54.4, C54.8-54.9, C57, C57.0-57.4, C57.7-57.8, C60, C60.0-60.2, C60.8-60.9, C63, C63.0-63.2, C63.7-63.8, C66, C66.0-66.2, C66.9, C68.0-68.1, C68.8, C75, C75.0, C75.4-75.6, C75.8, D06, D06.0-06.1, D06.7, D06.9-07.2, D07.4, D09.2, D13.2-13.3, D14.0, D15-16, D15.0-15.2, D15.7, D15.9-16.9, D26.0-26.1, D26.7, D26.9, D28.0-28.1, D28.7, D29.0, D30.2, D30.4, D30.7-30.8, D31, D31.0-31.6, D31.9, D35-36, D35.0-35.1, D35.5-35.9, D36.1, D36.7, D37.2, D38.2-38.5, D39.2, D39.8, D41.2-41.3, D44.1-44.8, D48.0-48.4
C57.9, C59	C03.0, C04.0, C05.0, C07.0, C17, C17.0-17.3, C17.8-17.9, C30-31, C30.0-30.3, C30.5, C30.8-31.3, C31.8-31.9, C37-38, C37.0-37.3, C38.0-38.4, C38.8, C48, C48.0-48.2, C48.8-48.9, C51-54, C51.0-51.2, C51.8-52.0, C52.9-53.1, C53.3-53.4, C53.8-54.4, C54.8-54.9, C56-57, C56.0-56.2, C56.4, C56.9-57.4, C57.7-57.8, C60, C60.0-60.2, C60.8-60.9, C63, C63.0-63.2, C63.7-63.8, C66, C66.0-66.2, C66.9, C68.0-68.1, C68.8, C75, C75.0, C75.4-75.6, C75.8, D06, D06.0-06.1, D06.7, D06.9-07.2, D07.4, D09.2, D13.2-13.3, D14.0, D15-16, D15.0-15.2, D15.7, D15.9-16.9, D26.0-26.1, D26.7, D26.9-27.1, D27, D27.9-28.1, D28.7, D29.0, D30.2, D30.4, D30.7-30.8, D31, D31.0-31.6, D31.9, D35-36, D35.0-35.1, D35.5-35.9, D36.1, D36.7, D37.2, D38.2-38.5, D39.1-39.2, D39.8, D41.2-41.3, D44.1-44.8, D48.0-48.4
C63.9	C03.0, C04.0, C05.0, C07.0, C17, C17.0-17.3, C17.8-17.9, C30-31, C30.0-30.3, C30.5, C30.8-31.3, C31.8-31.9, C37-38, C37.0-37.3, C38.0-38.4, C38.8, C48, C48.0-48.2, C48.8-48.9, C51-52, C51.0-51.2, C51.8-52.0, C52.9, C57, C57.0-57.4, C57.7-57.8, C60-63, C60.0-60.2, C60.8-61.0, C61.9-62.1, C62.9-63.2, C63.7-63.8, C66, C66.0-66.2, C66.9, C68.0-68.1, C68.8, C75, C75.0, C75.4-75.6, C75.8, D07.4-07.5, D09.2, D13.2-13.3, D14.0, D15-16, D15.0-15.2, D15.7, D15.9-16.9, D28.0-28.1, D28.7, D29.0-29.4, D29.7-29.8, D30.2, D30.4, D30.7-30.8, D31, D31.0-31.6, D31.9, D35-36, D35.0-35.1, D35.5-35.9, D36.1, D36.7, D37.2, D38.2-38.5, D39.2, D39.8, D40.0-40.1, D40.7-40.8, D41.2-41.3, D44.1-44.8, D48.0-48.4
C68, C68.9	C03.0, C04.0, C05.0, C07.0, C17, C17.0-17.3, C17.8-17.9, C30-31, C30.0-30.3, C30.5, C30.8-31.3, C31.8-31.9, C37-38, C37.0-37.3, C38.0-38.4, C38.8, C48, C48.0-48.2, C48.8-48.9, C51-52, C51.0-51.2, C51.8-52.0, C52.9, C57, C57.0-57.4, C57.7-57.8, C60, C60.0-60.2, C60.8-60.9, C63-67, C63.0-63.2, C63.7-63.8, C64.0-64.2, C64.4-64.6, C64.8-65.2, C65.9-66.2, C66.9-68.1, C68.8, C75, C75.0, C75.4-75.6, C75.8, D07.4, D09.0, D09.2, D13.2-13.3, D14.0, D15-16, D15.0-15.2, D15.7, D15.9-16.9, D28.0-28.1, D28.7, D29.0, D30.0-30.4, D30.7-30.8, D31, D31.0-31.6, D31.9, D35-36, D35.0-35.1, D35.5-35.9, D36.1, D36.7, D37.2, D38.2-38.5, D39.2, D39.8, D41.0-41.4, D41.7-41.8, D44.1-44.8, D48.0-48.4, D49.4
C75.9, C75.91, C75.92	C03.0, C04.0, C05.0, C07.0, C17, C17.0-17.3, C17.8-17.9, C30-31, C30.0-30.3, C30.5, C30.8-31.3, C31.8-31.9, C37-38, C37.0-37.3, C38.0-38.4, C38.8, C48, C48.0-48.2, C48.8-48.9, C51-52, C51.0-51.2, C51.8-52.0, C52.9, C57, C57.0-57.4, C57.7-57.8, C60, C60.0-60.2, C60.8-60.9, C63, C63.0-63.2, C63.7-63.8, C66, C66.0-66.2, C66.9, C68.0-68.1, C68.8, C73, C73.0-73.5, C73.8-73.9, C75, C75.0, C75.4-75.6, C75.8, D07.4, D09.2-09.3, D09.8, D13.2-13.3, D14.0, D15-16, D15.0-15.2, D15.7, D15.9-16.9, D28.0-28.1, D28.7, D29.0, D30.2, D30.4, D30.7-30.8, D31, D31.0-31.6, D31.9, D34-36, D34.0, D34.9-35.1, D35.5-35.9, D36.1, D36.7, D37.2, D38.2-38.5, D39.2, D39.8, D41.2-41.3, D44.0-44.8, D48.0-48.4
C42, C76-80, C76.4, C76.41, C76.42, C76.5, C76.51, C76.52, C76.7-76.9, C77.3-77.4, C77.8-77.9, C79.2-79.3, C79.31, C79.32, C79.4, C79.49, C79.5, C79.51, C79.52, C79.6, C79.61, C79.62, C79.7, C79.71, C79.72, C79.8, C79.81, C79.82, C79.89, C79.9-80.2, C80.9, C87, C97-99, C97.0, C97.9	C00-13, C00.0-00.6, C00.8-01.0, C01.9-02.4, C02.8-03.1, C03.9-04.1, C04.8-05.2, C05.8-06.2, C06.8-07.0, C07.9-08.1, C08.8-09.1, C09.8-10.4, C10.8-11.3, C11.8-12.0, C12.9-13.2, C13.8-13.9, C15-21, C15.0-15.5, C15.8-17.3, C17.8-19.0, C19.9-20.0, C20.8-21.2, C21.8-21.9, C22.2, C23-25, C23.0, C23.9-24.1, C24.4, C24.8-25.4, C25.7-25.9, C30-34, C30.0-30.3, C30.5, C30.8-31.3, C31.8-32.3, C32.8-33.0, C33.2, C33.9-34.4, C34.7-34.9, C37-38, C37.0-37.3, C38.0-38.4, C38.8, C40-41, C40.0-40.3, C40.8-41.9, C43-44, C43.0-44.9, C47-54, C47.0-47.6, C47.8-48.2, C48.8-49.6, C49.8-51.2, C51.8-52.0, C52.9-53.1, C53.3-53.4, C53.8-54.4, C54.8-54.9, C56-57, C56.0-56.2, C56.4, C56.9-57.4, C57.7-57.8, C60-67, C60.0-60.2, C60.8-61.0, C61.9-62.1, C62.9-63.2, C63.7-63.8, C64.0-64.2, C64.4-64.6, C64.8-65.2, C65.9-66.2, C66.9-68.1, C68.8, C69.0-69.8, C70-73, C70.0-70.1, C70.5-70.6, C70.9-72.5, C72.8-73.5, C73.8-73.9, C75, C75.0-75.6, C75.8, C81-82, C81.0-83.8, C84-86, C84.0-85.0, C85.2-85.8, C86.0-86.6, C88, C88.0-88.4, C88.7-88.9, C90-96, C90.0-91.0, C91.2-91.3, C91.6, C92.0-92.6, C93.0-93.1, C93.3, C93.8, C94.0-94.5, C94.7-94.8, C95.0-95.2, C95.4, C95.6-95.7, C95.9-96.9, D00.1-00.2, D01.0-01.3, D02.0-02.3, D03-06, D03.0-05.1, D05.7-06.1, D06.7, D06.9-07.2, D07.4-07.5, D09.0, D09.2-09.3, D09.8, D10.0-10.7, D11-12, D11.0, D11.7, D11.9-13.3, D13.5-13.7, D14.0-14.3, D15-16, D15.0-15.2, D15.7, D15.9-16.9, D22-24, D22.0-22.7, D22.9-23.7, D23.9-24.2, D24.9, D26.0-26.1, D26.7, D26.9-27.1, D27, D27.9-28.1, D28.7, D29.0-29.4, D29.7-29.8, D30.0-30.4, D30.7-30.8, D31, D31.0-31.6, D31.9, D34-36, D34.0, D34.9-35.1, D35.5-35.9, D36.1, D36.7, D37.1-37.5, D38.0-38.5, D39.1-39.2, D39.8, D40.0-40.1, D40.7-40.8, D41.0-41.4, D41.7-41.8, D44.0-44.8, D48.0-48.6, D49.2-49.4
C76.0-76.1, C77.0-77.1, C78.0, C78.01, C78.02, C78.1-78.3, C78.39	C00-13, C00.0-00.6, C00.8-01.0, C01.9-02.4, C02.8-03.1, C03.9-04.1, C04.8-05.2, C05.8-06.2, C06.8-07.0, C07.9-08.1, C08.8-09.1, C09.8-10.4, C10.8-11.3, C11.8-12.0, C12.9-13.2, C13.8-13.9, C15, C15.0-15.5, C15.8-15.9, C17, C17.0-17.3, C17.8-17.9, C30-34, C30.0-30.3, C30.5, C30.8-31.3, C31.8-32.3, C32.8-33.0, C33.2, C33.9-34.4, C34.7-34.9, C37-38, C37.0-37.3, C38.0-38.4, C38.8, C43-45, C43.0-45.9, C48, C48.0-48.2, C48.8-48.9, C50-52, C50.0-51.2, C51.8-52.0, C52.9, C57,

	C57.0-57.4, C57.7-57.8, C60, C60.0-60.2, C60.8-60.9, C63, C63.0-63.2, C63.7-63.8, C66, C66.0-66.2, C66.9, C68.0-68.1, C68.8, C70-73, C70.0-70.1, C70.5-70.6, C70.9-72.5, C72.8-73.5, C73.8-73.9, C75, C75.0-75.6, C75.8, D00.1, D02.0-02.3, D03-05, D03.0-05.1, D05.7-05.9, D07.4, D09.2-09.3, D09.8, D10.0-10.7, D11, D11.0, D11.7, D11.9, D13.0, D13.2-13.3, D14.0-14.3, D15-16, D15.0-15.2, D15.7, D15.9-16.9, D22-24, D22.0-22.7, D22.9-23.7, D23.9-24.2, D24.9, D28.0-28.1, D28.7, D29.0, D30.2, D30.4, D30.7-30.8, D31, D31.0-31.6, D31.9, D34-36, D34.0, D34.9-35.1, D35.5-35.9, D36.1, D36.7, D37.2, D38.0-38.5, D39.2, D39.8, D41.2-41.3, D44.0-44.8, D48.0-48.6, D49.2-49.3
C76.2-76.3, C77.2, C77.5, C78.4-78.8, C78.89, C79.0, C79.01, C79.02, C79.1, C79.11, C79.19	C02.0, C03.0, C04.0, C05.0, C07.0, C15-25, C15.0-15.5, C15.8-17.3, C17.8-19.0, C19.9-20.0, C20.8-21.2, C21.8-22.5, C22.7-22.8, C23.0, C23.9-24.1, C24.4, C24.8-25.4, C25.7-25.9, C30-31, C30.0-30.3, C30.5, C30.8-31.3, C31.8-31.9, C37-38, C37.0-37.3, C38.0-38.4, C38.8, C45, C45.0-45.9, C48, C48.0-48.2, C48.8-48.9, C51-54, C51.0-51.2, C51.8-52.0, C52.9-53.1, C53.3-53.4, C53.8-54.4, C54.8-54.9, C56-57, C56.0-56.2, C56.4, C56.9-57.4, C57.7-57.8, C60-61, C60.0-60.2, C60.8-61.0, C61.9, C63-67, C63.0-63.2, C63.7-63.8, C64.0-64.2, C64.4-64.6, C64.8-65.2, C65.9-66.2, C66.9-68.1, C68.8, C75, C75.0, C75.4-75.6, C75.8, C82, C82.0-83.8, C84-86, C84.0-85.0, C85.2-85.8, C86.0-86.6, C88, C88.0-88.4, C88.7-88.9, C90, C90.0-90.9, C96, C96.0-96.9, D00.1-00.2, D01.0-01.3, D06, D06.0-06.1, D06.7, D06.9-07.2, D07.4-07.5, D09.0, D09.2, D12, D12.0-13.7, D14.0, D15-16, D15.0-15.2, D15.7, D15.9-16.9, D26.0-26.1, D26.7, D26.9-27.1, D27, D27.9-28.1, D28.7, D29.0-29.1, D30.0-30.4, D30.7-30.8, D31, D31.0-31.6, D31.9, D35-36, D35.0-35.1, D35.5-35.9, D36.1, D36.7, D37.1-37.5, D38.2-38.5, D39.1-39.2, D39.8, D40.0, D41.0-41.4, D41.7-41.8, D44.1-44.8, D48.0-48.4, D49.4
C46, C46.0-46.5, C46.51, C46.52, C46.6-46.9	C49, C49.0-49.6, C49.8-49.9
C22.9	C02.0, C15-16, C15.0-15.5, C15.8-16.9, C18-25, C18.0-19.0, C19.9-20.0, C20.8-21.2, C21.8-22.5, C22.7-22.8, C23.0, C23.9-24.1, C24.4, C24.8-25.4, C25.7-25.9, C50, C50.0-50.9, C53-54, C53.0-53.1, C53.3-53.4, C53.8-54.4, C54.8-54.9, C56, C56.0-56.2, C56.4, C56.9, C61-62, C61.0, C61.9-62.1, C62.9, C64-65, C64.0-64.2, C64.4-64.6, C64.8-65.2, C65.9, C67, C67.0-67.9, D00.1-00.2, D01.0-01.3, D05-06, D05.0-05.1, D05.7-06.1, D06.7, D06.9-07.2, D07.5, D09.0, D12, D12.0-13.1, D13.4-13.7, D24, D24.0-24.2, D24.9, D26.0-26.1, D26.7, D26.9-27.1, D27, D27.9, D29.1-29.4, D29.7-29.8, D30.0-30.1, D30.3, D37.1, D37.3-37.5, D39.1, D40.0-40.1, D40.7-40.8, D41.0-41.1, D41.4, D41.7-41.8, D48.6, D49.3-49.4
C69, C69.9, C69.91, C69.92	C69.0-69.8
C74, C74.0	C03.0, C04.0, C05.0, C07.0, C17, C17.0-17.3, C17.8-17.9, C30-31, C30.0-30.3, C30.5, C30.8-31.3, C31.8-31.9, C37-38, C37.0-37.3, C38.0-38.4, C38.8, C47-48, C47.0-47.6, C47.8-48.2, C48.8-48.9, C51-52, C51.0-51.2, C51.8-52.0, C52.9, C57, C57.0-57.4, C57.7-57.8, C60, C60.0-60.2, C60.8-60.9, C63, C63.0-63.2, C63.7-63.8, C66, C66.0-66.2, C66.9, C68.0-68.1, C68.8, C75, C75.0, C75.4-75.6, C75.8, D07.4, D09.2, D13.2-13.3, D14.0, D15-16, D15.0-15.2, D15.7, D15.9-16.9, D28.0-28.1, D28.7, D29.0, D30.2, D30.4, D30.7-30.8, D31, D31.0-31.6, D31.9, D35-36, D35.0-35.1, D35.5-35.9, D36.1, D36.7, D37.2, D38.2-38.5, D39.2, D39.8, D41.2-41.3, D44.1-44.8, D48.0-48.4
C91.1, C91.11, C91.12	C91.0, C91.2-91.3, C91.6
C91.4, C91.41, C91.42, C91.5, C91.51, C91.52, C91.7-91.9, C91.91, C91.92	C91.0, C91.2-91.3, C91.6, C93.1, C93.3, C93.8, C94.1, C94.3, C94.7-94.8
C92.7-92.9, C92.91, C92.92, C93.2, C93.5, C93.7, C93.9, C93.91, C93.92	C92.0, C92.3-92.6, C93.0-93.1, C93.3, C93.8, C94.0-94.5, C94.7-94.8
C74.01, C74.02, C74.1, C74.11, C74.12	C03.0, C04.0, C05.0, C07.0, C17, C17.0-17.3, C17.8-17.9, C30-31, C30.0-30.3, C30.5, C30.8-31.3, C31.8-31.9, C37-38, C37.0-37.3, C38.0-38.4, C38.8, C47-48, C47.0-47.6, C47.8-48.2, C48.8-48.9, C51-52, C51.0-51.2, C51.8-52.0, C52.9, C57, C57.0-57.4, C57.7-57.8, C60, C60.0-60.2, C60.8-60.9, C63, C63.0-63.2, C63.7-63.8, C66, C66.0-66.2, C66.9, C68.0-68.1, C68.8, C75, C75.0, C75.4-75.6, C75.8, D07.4, D09.2, D13.2-13.3, D14.0, D15-16, D15.0-15.2, D15.7, D15.9-16.9, D28.0-28.1, D28.7, D29.0, D30.2, D30.4, D30.7-30.8, D31, D31.0-31.6, D31.9, D35-36, D35.0-35.1, D35.5-35.9, D36.1, D36.7, D37.2, D38.2-38.5, D39.2, D39.8, D41.2-41.3, D44.1-44.8, D48.0-48.4
C74.9, C74.91, C74.92	C03.0, C04.0, C05.0, C07.0, C17, C17.0-17.3, C17.8-17.9, C30-31, C30.0-30.3, C30.5, C30.8-31.3, C31.8-31.9, C37-38, C37.0-37.3, C38.0-38.4, C38.8, C47-48, C47.0-47.6, C47.8-48.2, C48.8-48.9, C51-52, C51.0-51.2, C51.8-52.0, C52.9, C57, C57.0-57.4, C57.7-57.8, C60, C60.0-60.2, C60.8-60.9, C63, C63.0-63.2, C63.7-63.8, C66, C66.0-66.2, C66.9, C68.0-68.1, C68.8, C75, C75.0, C75.4-75.6, C75.8, D07.4, D09.2, D13.2-13.3, D14.0, D15-16, D15.0-15.2, D15.7, D15.9-16.9, D28.0-28.1, D28.7, D29.0, D30.2, D30.4, D30.7-30.8, D31, D31.0-31.6, D31.9, D35-36, D35.0-35.1, D35.5-35.9, D36.1, D36.7, D37.2, D38.2-38.5, D39.2, D39.8, D41.2-41.3, D44.1-44.8, D48.0-48.4
C94.6	C92.0, C92.3-92.6, C93.0-93.1, C93.3, C93.8, C94.0-94.5, C94.7-94.8
C76.4-76.5	C40-41, C40.0-40.3, C40.8-41.9, C49, C49.0-49.6, C49.8-49.9
C83, C83.9, C85.9	C82, C82.0-83.8, C84-86, C84.0-85.0, C85.2-85.8, C86.0-86.6, C96, C96.0-96.9

7. Removal of duplicates. In the seventh step (#7 in Appendix 1 Figure 1), duplicate or redundant data sources were removed from the processed cancer registry datasets. Duplicate sources were present if, for example, a cancer registry was part of the CI5[14–24] database but we also had data from that registry directly. Redundancies occurred and were removed as described in the "Cancer registry data sources" section above, where more detailed data were available, or when national registry data could replace subnationally representative data. From here, two parallel selection processes were run: one to generate input data for the mortality-to-incidence ratio (MIR) models, and one to generate incidence for final mortality estimation. When creating the final incidence input, higher priority was given to registry data from the most standardised source; whereas for the MIR model input, only sources that reported both incidence and mortality were used.

8. Combine matching incidence and mortality data and model MIRs. In the eighth step (#8 in the flowchart) the processed incidence and mortality data from cancer registries were matched by cancer, age, sex, year, and location to generate MIRs. These MIRs were used as input for further modelling, using one of two approaches, depending on the cancer.

8a. MIR input data

New for GBD 2023, we processed incidence and mortality data from paediatric-specific cancer registries independent of the data from all-ages cancer registries and included both in our MIR models, to support more accurate paediatric estimates. For all causes that existed in GBD 2019, data cleaning steps for the input data to MIR estimation in GBD 2023 were similar as for GBD 2021. For each cancer, MIRs from locations in Healthcare Access and Quality (HAQ) Index quintiles 1–4 were dropped if they were below the median of MIRs from locations in HAQ Index quintile 5. We also dropped MIRs from locations in HAQ Index quintiles 1–4 if the MIRs were above an outlier threshold calculated as the third quartile + 1.5 * IQR (interquartile range). We dropped all MIR data that were based on fewer than 15 incident cases to avoid excessive variation in the ratio due to small numbers. An exception to this threshold was made for mesothelioma, acute myeloid leukaemia and, new in GBD 2023, acute lymphoid leukaemia, where instead we dropped MIRs that were based on fewer than ten cases because of lower data availability for these three cancers. For the lower end of the age spectrum where cancers are generally rarer, in GBD 2021 we aggregated incidence and mortality to the youngest five-year age bin where SEER[13] reported at least 50 cases from 1990 to 2015, to avoid unstable MIR predictions in young age groups because of too few cases or deaths. For GBD 2023, instead of using the SEER-based age threshold, we allowed age groups with greater than ten observations and more than one unique location to retain their age-specific data and be modeled independently. Below this new threshold, the MIR estimates were copied down to all younger GBD age groups estimated for that cancer. This resulted in younger age groups being modelled with observed data than in prior GBD rounds.

For the nine new cancer causes estimated for the first time in GBD 2021, additional data processing steps were used to help stabilise the input data and MIR estimates. These additional steps were determined to be necessary due to the much smaller counts of cases and deaths in these causes. First, for retinoblastoma and hepatoblastoma, data were aggregated across sexes and across bins of ten calendar years (for example, 2000–2009). Data were then only excluded if there were less than 0.01 cases, since such small values were a result of redistribution and were leading to implausibly high MIRs. As cancer registry mortality data were limited for retinoblastoma, we supplemented with mortality data from vital registration systems. For this cause, cancer registry incidence data was matched with vital registration mortality data by age, year, sex, and location. These cancer registry-vital registration matched inputs were processed the same as the standard matched cancer registry inputs.

Since MIRs can be above 1, especially in older age groups and for cancers with low cure rates, we used the 95th percentile (by age group) of the cleaned dataset (detailed above) as upper limits for the MIR input data. Any MIR input values over this upper limit cap were Winsorised to the cap value. These “upper cap” values were used to allow MIRs over 1 in some age groups but to constrain the MIRs to a maximum level. The addition of new data for GBD 2023 led to slightly different upper caps compared to GBD 2021 (see upper cap values below). For GBD 2023, we no longer set an upper cap of 1 (regardless of the 95th percentile) for paediatric age groups (under age 20 years), as was done in GBD 2021.

Age group (years)	GBD 2021 upper cap	GBD 2023 upper cap
0–4	1.00	0.678
5–9	1.00	0.750
10–14	1.00	0.793
15–19	1.00	0.961
20–24	1.04	0.954
25–29	0.95	0.798
30–34	0.94	0.752
35–39	0.89	0.812
40–44	0.93	0.859
45–49	0.95	0.866
50–54	0.96	0.893
55–59	0.99	0.925
60–64	1.02	0.973
65–69	1.06	1.02
70–74	1.11	1.06
75–79	1.18	1.16
80–84	1.27	1.28
85–89	1.36	1.41
90–94	1.48	1.61
95+	1.61	1.84

8b. MIR modelling

As in previous GBD cycles, MIRs for most cancers were estimated with a three-step modelling approach using the general GBD spatiotemporal Gaussian process regression (ST-GPR) method. The first step used a linear model for logit-transformed MIR as the outcome, with covariates for sex, categorical age group (number of categories depending on cancer type), and HAQ Index.[29] For the MIRs, this initial linear prior used the following general form:

$$\text{logit}\left(MIR_{c,a,s,t}\right) = \alpha + \beta_1 (HAQIndex)_{c,t} + \beta_2 \mathrm{I}_s + \sum_{a}^{A} \beta_a \mathrm{I}_a + \epsilon$$

MIR = mortality-to-incidence ratio
c = country (or subnational location for subnationally modelled locations), a = age group, t: = time (years); s = sex
HAQIndex = Healthcare Access and Quality Index
I = indicator variable
ϵ = error term

Results from the final linear model were used as input for space-time smoothing and a Gaussian Process Regression. The ST-GPR model has three main hyperparameters that control for smoothing across time, age, and geography.[3] These hyperparameter values were iteratively evaluated in GBD 2019 for balancing model performance with local data availability, and were not changed for GBD 2021 or GBD 2023. The time adjustment parameter lambda (λ) aims to borrow strength from neighbouring time points (i.e., the value in this year is highly correlated with the value in the previous year but less so further back in time) and was set to 0.05. The age adjustment parameter omega (ω) borrows strength from data in neighbouring age groups and was set to 0.5. The space adjustment parameter zeta (ξ) aims to borrow strength across the GBD hierarchy of geographical locations and was set to 0.01. For the remaining parameters in the Gaussian process regression, we set amplitude to 1 (influences fluctuation from the mean function) and set the scale value to 10 (influences the time distance over which points are correlated).

Additional details on ST-GPR can be found in the appendix to the GBD 2023 paper, "Global burden of 292 causes of death in 204 countries and territories and 660 subnational locations, 1990–2023: a systematic analysis for the

Global Burden of Disease Study 2023. Lancet (in review)".[2] These models were used to obtain MIR estimates for all combinations of GBD cause, age group, sex, year, and location. Input data points were outliered manually if they clearly influenced the model in an unrealistic way. For example, a data point was marked as an outlier if it created a single year, single age group spike in model predictions that was inconsistent with the trend suggested by surrounding data points. To run the logit model in ST-GPR, the input data were first divided by the upper caps to get proportional data ranging from 0 to 1. Model predictions from ST-GPR were then rescaled back to MIRs by multiplying the scaled predictions by the upper caps. To constrain the MIRs at the lower end of the distribution, we used the fifth percentile of the cancer and age-specific cleaned MIR input data to Winsorise all model predictions below this lower cap.

As in GBD 2021, for a subset of cancers we used a different approach than ST-GPR to model MIRs because of differences in data availability, the age distribution of cases and deaths, and the reliability of the modelled estimates. Details for each of these causes are available in Appendix 1 Table 7. For retinoblastoma and hepatoblastoma, we modeled MIRs using a negative binomial regression approach for GBD 2023. The negative binomial approach was used for these two cancers because it allows modelling of count data with overdispersion (meaning the mean and variance are allowed to differ in the underlying distribution), which was determined to be needed due to the relatively low number of deaths from these cancer causes. MIRs were estimated for each age, sex, year, and location combination using a negative binomial regression run in R (version 4.4.0) using glm.nb from the MASS package. Models included covariates for HAQ Index and categorical age groups and were offset by the logarithm of cases. As with the ST-GPR models, the modelled MIRs were Winsorised to the upper and lower cap values. For hepatoblastoma, the negative binomial approach is a change from GBD 2021, in which MIRs for hepatoblastoma were estimated as part of the parent liver cancer model and copied to hepatoblastoma (age 0–9). For five additional cancers that had been modelled using negative binomial models in GBD 2021, in GBD 2023 these were able to be modelled using the ST-GPR methods described above due to greater data availability this round. For the "eye cancer" parent cause, the MIRs were copied from the models for the child causes of retinoblastoma (age 0–9 years) and "other eye cancer" (age 10+ years), as was done in GBD 2021.

Appendix Table 7: Modelling details for causes using negative binomial MIR models in GBD 2023

**Cancer cause**	**MIR model type**	**Input data**	**HAQ Index covariate**	**Age groups covariate**
Retinoblastoma	Negative Binomial	CR, VR	Continuous	GBD 5–year age groups
Hepatoblastoma	Negative Binomial	CR	Continuous	GBD 5–year age groups

Abbreviations: CR, cancer registry; GBD, Global Burden of Diseases, Injuries, and Risk Factors Study 2023; MIR, mortality-to-incidence ratio; VR, vital registration.

9. Generate mortality estimates from incidence and MIRs. Final estimated MIRs were matched with the cleaned cancer registry (CR) incidence dataset to generate mortality input data (#9 in Appendix 1 Figure 1):

$$MIR_{estimates} * \ incidence_{CR} = mortality_{CR\ inputs}$$

These mortality estimates were then smoothed by a Bayesian noise reduction algorithm and a non-zero floor algorithm to deal with zero counts, both of which were also applied to the additional CoD data inputs (vital registration and verbal autopsy data), as specified in Section X of the appendix to the GBD 2023 paper "Global burden of 292 causes of death in 204 countries and territories and 660 subnational locations, 1990–2023: a systematic analysis for the Global Burden of Disease Study 2023. Lancet (in review)" *(To the editors and reviewers: note that section detail will be finalized once the GBD 2023 Causes of Death Collaborators publication is final)*.[2]

10. Compositional bias and crosswalk approach. Compositional bias can occur when data from multiple reporting systems are used across time, for example Cancer Registry (CR) and Vital Registration (VR) data. When there is a switch between reporting systems over time a disjoint can occur that is an artifact of underlying differences between the reporting systems (e.g., how cancer data is collected, reported, coded, etc.) rather than actual changes in cancer mortality that occurred over time. In GBD 2023 we newly applied a crosswalk using RegMod, that takes systematically biased data points and estimates their unbiased value based on a gold standard reference set.[30] This crosswalk adjustment was applied to the CR data, leveraging the VR data as the reference standard to provide more appropriate time trends in locations with multiple data sources available.

To correct for compositional bias due to systematic differences between CR and VR data, this crosswalk approach was applied to all causes and locations with CR and VR data that overlapped. The crosswalk method differed depending on the number of years of overlapping CR and VR data for a given location. For those locations with two years or more of overlap, a spline method was used; for those locations with less than two years of overlap, a monotonic method was used. For those locations that only had CR data (i.e., no VR data) or in which the model did not converge, the pre-crosswalked CR value was retained. Pre-crosswalked CR data was also retained in locations where the only VR data available was from 2019 or later, due to concern that effects of the COVID-19 pandemic may have made the VR data less reliable to use for crosswalk purposes.

Compositional bias can also occur when there are multiple data sources for the same point in time. Once crosswalking was complete, CR data that had a direct overlap in the VR time series was dropped. This process substantially decreased the amount of CR data that directly contributed to mortality estimates in GBD 2023, which is reflected in the lower source counts compared to previous rounds. The CR data remaining after this exclusion thus reflects CR contributions to mortality beyond the data from VR systems. (#10 in Appendix 1 Figure 1).

11. Upload CR data to CoD database. These data were uploaded into the CoD database as cancer registry data (#11 in Appendix 1 Figure 1). Cancer-specific mortality modelling then followed the general cause of death ensemble model (CODEm) process using the totality of verbal autopsy, vital registration, and cancer registry data.

CODEm

Mortality estimates for each cancer were generated using the GBD CODEm (#12 in Appendix 1 Figure 1) approach, the methods of which have been described in previous publications.[31] Additional details can be found in Section X of the appendix to the GBD 2023 paper, "Global burden of 292 causes of death in 204 countries and territories and 660 subnational locations, 1990–2023: a systematic analysis for the Global Burden of Disease Study 2023. Lancet (in review)" *(To the editors and reviewers: note that section detail will be finalized once the GBD 2023 Causes of Death Collaborators publication is final)*.[2]

In brief, the CODEm approach is based on several principles: that all types of available data should be used, even if data quality varies; that a diverse set of plausible models with different combinations of covariates should be

evaluated; that both individual models and the overall ensemble models should be tested for their predictive validity; and that the best model or sets of models should be chosen based on the out-of-sample predictive validity.

Covariates are provided for potential use in the ensemble based on a possible predictive relationship between the covariate and the specific cancer mortality, with an expected level and direction of association. Generally, Level 1 covariates have a proven strong relationship with the outcome, such as aetiological or biological roles. Level 2 covariates have a strong relationship but no known direct biological link. Level 3 covariates have a relationship that may be more distal in the causal chain, or are mediated through Level 1 or 2 covariates.[31] The covariates provided to CODEm, as well as their level and direction, differ by cause and sex. The covariates used for GBD 2023, and differences between GBD 2021 and GBD 2023 covariates, can be found in Appendix 1 Table 8.

Appendix Table 8: Covariates provided to CODEm for each GBD cause, by level and direction, in GBD 2021 and 2023

GBD cause	Covariate name	Level GBD 2021	Direction GBD 2021	Level GBD 2023	Direction GBD 2023
Acute lymphoid leukemia	Healthcare access and quality index	2	-1	2	-1
Acute lymphoid leukemia	Mean BMI	2	1	2	1
Acute lymphoid leukemia	Education (years per capita)	3	-1	3	-1
Acute lymphoid leukemia	LDI (I$ per capita)	3	-1	3	-1
Acute lymphoid leukemia	Socio-demographic Index	3	1	3	1
Acute lymphoid leukemia	Log-transformed age-standardized SEV scalar: Leukemia	1	1	NA	NA
Acute lymphoid leukemia	Log-transformed SEV scalar: Leukemia	1	1	NA	NA
Acute myeloid leukemia	Cumulative Cigarettes (10 Years)	2	1	2	1
Acute myeloid leukemia	Cumulative Cigarettes (20 Years)	2	1	2	1
Acute myeloid leukemia	Healthcare access and quality index	2	-1	2	-1
Acute myeloid leukemia	Liters of alcohol consumed per capita	2	1	2	1
Acute myeloid leukemia	Mean BMI	2	1	2	1
Acute myeloid leukemia	Smoking Prevalence	2	1	2	1
Acute myeloid leukemia	Education (years per capita)	3	-1	3	-1
Acute myeloid leukemia	LDI (I$ per capita)	3	1	3	1
Acute myeloid leukemia	Socio-demographic Index	3	1	3	1
Acute myeloid leukemia	Log-transformed age-standardized SEV scalar: Leukemia	1	1	NA	NA
Acute myeloid leukemia	Log-transformed SEV scalar: Leukemia	1	1	NA	NA
Bladder cancer	Cumulative Cigarettes (10 Years) [Data Rich, female]	2	1	1	1
Bladder cancer	Healthcare access and quality index [Data Rich, female]	2	-1	1	-1
Bladder cancer	Schistosomiasis Prevalence Results	1	1	1	1
Bladder cancer	Smoking Prevalence	1	1	1	1
Bladder cancer	Socio-demographic Index [Data Rich, female]	3	1	1	1
Bladder cancer	Age- and sex-specific SEV for Low vegetables	2	1	2	1
Bladder cancer	Cumulative Cigarettes (10 Years) [Data Rich, male]	2	1	2	1
Bladder cancer	Cumulative Cigarettes (10 Years) [Global]	2	1	2	1
Bladder cancer	Diabetes Fasting Plasma Glucose (mmol/L), age-standardized 25+	2	1	2	1
Bladder cancer	Healthcare access and quality index [Data Rich, male]	2	-1	2	-1
Bladder cancer	Healthcare access and quality index [Global]	2	-1	2	-1
Bladder cancer	Liters of alcohol consumed per capita	2	1	2	1
Bladder cancer	Age- and sex-specific SEV for Low fruit	3	1	3	1
Bladder cancer	LDI (I$ per capita)	3	1	3	1
Bladder cancer	Socio-demographic Index [Data Rich, male]	3	1	3	1
Bladder cancer	Socio-demographic Index [Global]	3	1	3	1
Bladder cancer	Log-transformed SEV scalar: Bladder C	1	1	NA	NA
Brain and central nervous system cancer	Cumulative Cigarettes (10 Years)	1	1	1	1
Brain and central nervous system cancer	Liters of alcohol consumed per capita	1	1	1	1
Brain and central nervous system cancer	Smoking Prevalence	1	1	1	1
Brain and central nervous system cancer	Age- and sex-specific SEV for High red meat	2	1	2	1
Brain and central nervous system cancer	Age- and sex-specific SEV for Low fruit	2	1	2	1
Brain and central nervous system cancer	Age- and sex-specific SEV for Low vegetables	2	1	2	1
Brain and central nervous system cancer	Healthcare access and quality index	2	-1	2	-1
Brain and central nervous system cancer	Low-Density Lipoprotein (mmol/L)	2	1	2	1
Brain and central nervous system cancer	Systolic Blood Pressure (mmHg)	2	1	2	1
Brain and central nervous system cancer	Education (years per capita)	3	-1	3	-1

Brain and central nervous system cancer	LDI (I$ per capita)	3	-1	3	-1
Brain and central nervous system cancer	Socio-demographic Index	3	1	3	1
Breast cancer	Liters of alcohol consumed per capita	1	1	1	1
Breast cancer	Mean BMI	1	1	1	1
Breast cancer	Age- and sex-specific SEV for Low fruit	2	1	2	1
Breast cancer	Age- and sex-specific SEV for Low vegetables	2	1	2	1
Breast cancer	Age-Specific Fertility Rate [female]	2	-1	2	-1
Breast cancer	Cumulative Cigarettes (10 Years)	2	1	2	1
Breast cancer	Cumulative Cigarettes (20 Years)	2	1	2	1
Breast cancer	Diabetes Fasting Plasma Glucose (mmol/L), age-standardized 25+	2	1	2	1
Breast cancer	Healthcare access and quality index	2	-1	2	-1
Breast cancer	Smoking Prevalence	2	1	2	1
Breast cancer	Total Fertility Rate [female]	2	-1	2	-1
Breast cancer	LDI (I$ per capita)	3	-1	3	-1
Breast cancer	Socio-demographic Index	3	1	3	1
Breast cancer	Log-transformed SEV scalar: Breast C	1	1	NA	NA
Burkitt lymphoma	Education (years per capita) [Data Rich, Global, Sub-Saharan Africa]	3	-1	3	-1
Burkitt lymphoma	Healthcare access and quality index [Data Rich, Global, Sub-Saharan Africa]	2	-1	2	-1
Burkitt lymphoma	Latitude Under 15 (proportion) [Sub-Saharan Africa]	2	1	2	1
Burkitt lymphoma	LDI (I$ per capita) [Data Rich, Global, Sub-Saharan Africa]	3	-1	3	-1
Burkitt lymphoma	Log-transformed age-standardized SEV scalar: HIV [Data Rich, Global, Sub-Saharan Africa]	2	1	1	1
Burkitt lymphoma	Log-transformed SEV scalar: HIV [Data Rich, Global, Sub-Saharan Africa]	2	1	1	1
Burkitt lymphoma	Malaria incidence adjusted for antimalarial coverage and drug effectiveness [Data Rich, Global, Sub-Saharan Africa]	2	1	1	1
Burkitt lymphoma	Malaria incidence_MAP [Data Rich, Global, Sub-Saharan Africa]	2	1	2	1
Burkitt lymphoma	Maternal care and immunization [Data Rich, Global, Sub-Saharan Africa]	3	-1	3	-1
Burkitt lymphoma	Socio-demographic Index [Data Rich, Global, Sub-Saharan Africa]	3	-1	3	-1
Burkitt lymphoma	Universal health coverage [Data Rich, Global, Sub-Saharan Africa]	2	-1	2	-1
Cervical cancer	Cumulative Cigarettes (5 Years) [female]	1	1	1	1
Cervical cancer	HIV age-standardized prevalence [female]	1	1	1	1
Cervical cancer	Log-transformed age-standardized SEV scalar: HIV [female]	1	1	1	1
Cervical cancer	Log-transformed SEV scalar: HIV [female]	1	1	1	1
Cervical cancer	Age- and sex-specific SEV for Low fruit [female]	2	1	2	1
Cervical cancer	Age- and sex-specific SEV for Low vegetables [female]	2	1	2	1
Cervical cancer	Age-Specific Fertility Rate [female]	2	1	2	1
Cervical cancer	Healthcare access and quality index [female]	2	-1	2	-1
Cervical cancer	Smoking Prevalence [female]	2	1	2	1
Cervical cancer	Total Fertility Rate [female]	2	1	2	1
Cervical cancer	Education (years per capita) [female]	3	-1	3	-1
Cervical cancer	LDI (I$ per capita) [female]	3	-1	3	-1
Cervical cancer	Socio-demographic Index [female]	3	-1	3	-1
Chronic lymphoid leukemia	Cumulative Cigarettes (10 Years)	2	1	2	1
Chronic lymphoid leukemia	Cumulative Cigarettes (15 Years)	2	1	2	1
Chronic lymphoid leukemia	Cumulative Cigarettes (20 Years)	2	1	2	1
Chronic lymphoid leukemia	Cumulative Cigarettes (5 Years)	2	1	2	1
Chronic lymphoid leukemia	Healthcare access and quality index	2	-1	2	-1
Chronic lymphoid leukemia	Liters of alcohol consumed per capita	2	1	2	1
Chronic lymphoid leukemia	Mean BMI	2	1	2	1
Chronic lymphoid leukemia	Smoking Prevalence	2	1	2	1
Chronic lymphoid leukemia	Tobacco (cigarettes per capita)	2	1	2	1
Chronic lymphoid leukemia	Education (years per capita)	3	-1	3	-1
Chronic lymphoid leukemia	LDI (I$ per capita)	3	-1	3	-1
Chronic lymphoid leukemia	Socio-demographic Index	3	1	3	1
Chronic lymphoid leukemia	Log-transformed age-standardized SEV scalar: Leukemia	1	1	NA	NA
Chronic lymphoid leukemia	Log-transformed SEV scalar: Leukemia	1	1	NA	NA

Chronic myeloid leukemia	Cumulative Cigarettes (10 Years)	2	1	2	1
Chronic myeloid leukemia	Cumulative Cigarettes (15 Years)	2	1	2	1
Chronic myeloid leukemia	Cumulative Cigarettes (20 Years)	2	1	2	1
Chronic myeloid leukemia	Cumulative Cigarettes (5 Years)	2	1	2	1
Chronic myeloid leukemia	Healthcare access and quality index	2	-1	2	-1
Chronic myeloid leukemia	Liters of alcohol consumed per capita	2	1	2	1
Chronic myeloid leukemia	Mean BMI	2	1	2	1
Chronic myeloid leukemia	Smoking Prevalence	2	1	2	1
Chronic myeloid leukemia	Tobacco (cigarettes per capita)	2	1	2	1
Chronic myeloid leukemia	Education (years per capita)	3	-1	3	-1
Chronic myeloid leukemia	LDI (I$ per capita)	3	1	3	1
Chronic myeloid leukemia	Socio-demographic Index	3	-1	3	-1
Chronic myeloid leukemia	Log-transformed age-standardized SEV scalar: Leukemia	1	1	NA	NA
Chronic myeloid leukemia	Log-transformed SEV scalar: Leukemia	1	1	NA	NA
Colon and rectum cancer	Age- and sex-specific SEV for High red meat	1	1	1	1
Colon and rectum cancer	Log-transformed SEV scalar: Colorect C	1	1	1	1
Colon and rectum cancer	Mean BMI	1	1	1	1
Colon and rectum cancer	Tobacco (cigarettes per capita)	1	1	1	1
Colon and rectum cancer	Total Physical Activity (MET-min/week), Age-specific	1	-1	1	-1
Colon and rectum cancer	Age- and sex-specific SEV for Low calcium	2	1	2	1
Colon and rectum cancer	Age- and sex-specific SEV for Low fiber	2	1	2	1
Colon and rectum cancer	Age- and sex-specific SEV for Low vegetables	2	1	2	1
Colon and rectum cancer	Cumulative Cigarettes (20 Years) [Global, male]	2	1	2	1
Colon and rectum cancer	Cumulative Cigarettes (5 Years) [Data Rich]	2	1	2	1
Colon and rectum cancer	Cumulative Cigarettes (5 Years) [Global, female]	2	1	2	1
Colon and rectum cancer	Diabetes Fasting Plasma Glucose (mmol/L), age-standardized 25+	2	1	2	1
Colon and rectum cancer	Liters of alcohol consumed per capita	2	1	2	1
Colon and rectum cancer	pufa adjusted(percent)	2	-1	2	-1
Colon and rectum cancer	Age- and sex-specific SEV for Low fruit	3	1	3	1
Colon and rectum cancer	Age- and sex-specific SEV for Low milk	3	1	3	1
Colon and rectum cancer	Age- and sex-specific SEV for Low nuts and seeds	3	1	3	1
Colon and rectum cancer	Education (years per capita)	3	-1	3	-1
Colon and rectum cancer	Healthcare access and quality index	3	-1	3	-1
Colon and rectum cancer	LDI (I$ per capita)	3	1	3	1
Colon and rectum cancer	Socio-demographic Index	3	1	3	1
Esophageal cancer	Liters of alcohol consumed per capita	1	1	1	1
Esophageal cancer	Mean BMI	1	1	1	1
Esophageal cancer	Smoking Prevalence	1	1	1	1
Esophageal cancer	Age- and sex-specific SEV for Low fruit	2	1	2	1
Esophageal cancer	Age- and sex-specific SEV for Low vegetables	2	1	2	1
Esophageal cancer	Healthcare access and quality index	2	-1	2	-1
Esophageal cancer	Indoor Air Pollution (All Cooking Fuels)	2	1	2	1
Esophageal cancer	Tobacco (cigarettes per capita)	2	1	2	1
Esophageal cancer	Education (years per capita)	3	-1	3	-1
Esophageal cancer	Improved Water Source (proportion with access)	3	-1	3	-1
Esophageal cancer	LDI (I$ per capita)	3	1	3	1
Esophageal cancer	Sanitation (proportion with access)	3	-1	3	-1
Esophageal cancer	Socio-demographic Index	3	1	3	1
Esophageal cancer	Log-transformed age-standardized SEV scalar: Esophag C	1	1	NA	NA
Eye cancer	Age-standardized melanoma	NA	NA	2	1
Eye cancer	Education (years per capita) [male]	NA	NA	2	-1
Eye cancer	Healthcare access and quality index	NA	NA	2	-1
Eye cancer	LDI (I$ per capita) [male]	NA	NA	2	-1
Eye cancer	Socio-demographic Index [male]	NA	NA	2	-1
Eye cancer	Universal health coverage	NA	NA	2	-1
Eye cancer	Universal health coverage	NA	NA	2	-1
Eye cancer	Universal health coverage	NA	NA	2	-1
Eye cancer	Universal health coverage	NA	NA	2	-1
Eye cancer	Education (years per capita) [female]	NA	NA	3	-1
Eye cancer	LDI (I$ per capita) [female]	NA	NA	3	-1
Eye cancer	Socio-demographic Index [female]	NA	NA	3	-1
Gallbladder and biliary tract cancer	Log-transformed SEV scalar: Gallblad C	1	1	1	1
Gallbladder and biliary tract cancer	Mean BMI	1	1	1	1
Gallbladder and biliary tract cancer	Age- and sex-specific SEV for Low fruit	2	1	2	1
Gallbladder and biliary tract cancer	Age- and sex-specific SEV for Low vegetables	2	1	2	1

Gallbladder and biliary tract cancer	Cumulative Cigarettes (10 Years)	2	1	2	1
Gallbladder and biliary tract cancer	Cumulative Cigarettes (5 Years)	2	1	2	1
Gallbladder and biliary tract cancer	Diabetes Age-Standardized Prevalence (proportion)	2	1	2	1
Gallbladder and biliary tract cancer	Healthcare access and quality index	2	-1	2	-1
Gallbladder and biliary tract cancer	Liters of alcohol consumed per capita	2	1	2	1
Gallbladder and biliary tract cancer	Smoking Prevalence	2	1	2	1
Gallbladder and biliary tract cancer	Tobacco (cigarettes per capita)	2	1	2	1
Gallbladder and biliary tract cancer	Education (years per capita)	3	-1	3	-1
Gallbladder and biliary tract cancer	LDI (I$ per capita)	3	1	3	1
Gallbladder and biliary tract cancer	Socio-demographic Index	3	-1	3	-1
Hodgkin lymphoma	Healthcare access and quality index	2	-1	2	-1
Hodgkin lymphoma	Education (years per capita)	3	-1	3	-1
Hodgkin lymphoma	LDI (I$ per capita)	3	-1	3	-1
Hodgkin lymphoma	Socio-demographic Index	3	-1	3	-1
Kidney cancer	Cumulative Cigarettes (10 Years)	1	1	1	1
Kidney cancer	Mean BMI	1	1	1	1
Kidney cancer	Tobacco (cigarettes per capita)	1	1	1	1
Kidney cancer	Diabetes Age-Standardized Prevalence (proportion)	2	1	2	1
Kidney cancer	Healthcare access and quality index	2	-1	2	-1
Kidney cancer	Liters of alcohol consumed per capita	2	1	2	1
Kidney cancer	Systolic Blood Pressure (mmHg)	2	1	2	1
Kidney cancer	Education (years per capita)	3	-1	3	-1
Kidney cancer	LDI (I$ per capita)	3	1	3	1
Kidney cancer	Socio-demographic Index	3	1	3	1
Kidney cancer	Log-transformed SEV scalar: Kidney C	1	1	NA	NA
Larynx cancer	Liters of alcohol consumed per capita	1	1	1	1
Larynx cancer	Age- and sex-specific SEV for Low fruit [Data Rich]	2	1	2	1
Larynx cancer	Age- and sex-specific SEV for Low vegetables [Global]	2	1	2	1
Larynx cancer	Asbestos consumption (metric tons per year per capita)	2	1	2	1
Larynx cancer	Cumulative Cigarettes (10 Years)	2	1	2	1
Larynx cancer	Cumulative Cigarettes (20 Years)	2	1	2	1
Larynx cancer	Healthcare access and quality index	2	-1	2	-1
Larynx cancer	Population Density (over 1000 ppl/sqkm, proportion)	2	1	2	1
Larynx cancer	Smoking Prevalence	2	1	2	1
Larynx cancer	Age- and sex-specific SEV for Low fruit [Global]	3	1	3	1
Larynx cancer	Age- and sex-specific SEV for Low vegetables [Data Rich]	3	1	3	1
Larynx cancer	LDI (I$ per capita)	3	1	3	1
Larynx cancer	Socio-demographic Index	3	1	3	1
Larynx cancer	Log-transformed SEV scalar: Larynx C	1	1	NA	NA
Leukemia	Cumulative Cigarettes (10 Years)	2	1	2	1
Leukemia	Cumulative Cigarettes (20 Years)	2	1	2	1
Leukemia	Healthcare access and quality index	2	-1	2	-1
Leukemia	Liters of alcohol consumed per capita	2	1	2	1
Leukemia	Mean BMI	2	1	2	1
Leukemia	Tobacco (cigarettes per capita)	2	1	2	1
Leukemia	Education (years per capita)	3	-1	3	-1
Leukemia	LDI (I$ per capita)	3	1	3	1
Leukemia	Socio-demographic Index	3	-1	3	-1
Leukemia	Log-transformed age-standardized SEV scalar: Leukemia	1	1	NA	NA
Leukemia	Log-transformed SEV scalar: Leukemia	1	1	NA	NA
Lip and oral cavity cancer	Cumulative Cigarettes (10 Years)	1	1	1	1
Lip and oral cavity cancer	Cumulative Cigarettes (15 Years) [Data Rich, female]	NA	NA	1	1
Lip and oral cavity cancer	Cumulative Cigarettes (20 Years)	1	1	1	1
Lip and oral cavity cancer	Cumulative Cigarettes (5 Years) [Data Rich, female]	NA	NA	1	1
Lip and oral cavity cancer	Liters of alcohol consumed per capita	1	1	1	1
Lip and oral cavity cancer	Tobacco (cigarettes per capita)	1	1	1	1
Lip and oral cavity cancer	Age- and sex-specific SEV for High red meat	2	1	2	1
Lip and oral cavity cancer	Age- and sex-specific SEV for Low fruit	2	1	2	1
Lip and oral cavity cancer	Age- and sex-specific SEV for Low vegetables	2	1	2	1
Lip and oral cavity cancer	Healthcare access and quality index	2	-1	2	-1

Lip and oral cavity cancer	Education (years per capita)	3	-1	3	-1
Lip and oral cavity cancer	LDI (I$ per capita)	3	1	3	1
Lip and oral cavity cancer	Socio-demographic Index	3	1	3	1
Lip and oral cavity cancer	Log-transformed SEV scalar: Lip Oral C	1	1	NA	NA
Liver cancer	Alcohol consumption, age standardized, in grams per day [Data Rich, female]	NA	NA	1	1
Liver cancer	Chronic Hepatitis C age standardized [Data Rich, female]	NA	NA	1	1
Liver cancer	Hepatitis B vaccine coverage (proportion), aged through time, COVID-inclusive [Data Rich, female]	NA	NA	1	1
Liver cancer	Hepatitis C Seroprevalence (anti-HCV) age standardized	1	1	1	1
Liver cancer	HIV age-standardized prevalence	1	1	1	1
Liver cancer	Intravenous drug use (proportion by age) [Data Rich, female]	NA	NA	1	1
Liver cancer	Liters of alcohol consumed per capita	1	1	1	1
Liver cancer	Vaccine adjusted HbSAg serporevalence age standardized	1	1	1	1
Liver cancer	Cumulative Cigarettes (20 Years)	2	1	2	1
Liver cancer	Diabetes Fasting Plasma Glucose (mmol/L), age-standardized 25+	2	1	2	1
Liver cancer	Healthcare access and quality index	2	-1	2	-1
Liver cancer	Hepatitis B 3-dose coverage, COVID-free (proportion)	NA	NA	2	-1
Liver cancer	Hepatitis B vaccine coverage, aged through time, COVID-free (proportion)	NA	NA	2	-1
Liver cancer	Intravenous drug use (age-standardized proportion)	2	1	2	1
Liver cancer	Mean BMI	2	1	2	1
Liver cancer	Tobacco (cigarettes per capita)	2	1	2	1
Liver cancer	Age- and sex-specific SEV for High red meat	3	1	3	1
Liver cancer	Education (years per capita)	3	-1	3	-1
Liver cancer	LDI (I$ per capita)	3	-1	3	-1
Liver cancer	Socio-demographic Index	3	-1	3	-1
Liver cancer	Hepatitis B 3-dose coverage (proportion)	2	-1	NA	NA
Liver cancer	Hepatitis B vaccine coverage (proportion), aged through time	2	-1	NA	NA
Liver cancer	Log-transformed SEV scalar: Liver C	1	1	NA	NA
Malignant neoplasm of bone and articular cartilage	Healthcare access and quality index	2	-1	2	-1
Malignant neoplasm of bone and articular cartilage	LDI (I$ per capita)	2	-1	2	-1
Malignant neoplasm of bone and articular cartilage	Socio-demographic Index	2	-1	2	-1
Malignant neoplasm of bone and articular cartilage	Universal health coverage	2	-1	2	-1
Malignant neoplasm of bone and articular cartilage	Age- and sex-specific SEV for Low bone mineral density	3	1	3	1
Malignant neoplasm of bone and articular cartilage	Education (years per capita)	3	-1	3	-1
Malignant neoplasm of bone and articular cartilage	Health worker density	3	-1	3	-1
Malignant neoplasm of bone and articular cartilage	Log-transformed age-standardized SEV scalar: Osteoarth	3	1	3	1
Malignant neoplasm of bone and articular cartilage	Log-transformed SEV scalar: Osteoarth	3	1	3	1
Malignant neoplasm of bone and articular cartilage	Maternal care and immunization	3	-1	3	-1
Malignant neoplasm of bone and articular cartilage	Smoking Prevalence	3	1	3	1
Malignant skin melanoma	Liters of alcohol consumed per capita	1	1	1	1
Malignant skin melanoma	Healthcare access and quality index	2	-1	2	-1
Malignant skin melanoma	Latitude 15 to 30 (proportion)	2	-1	2	-1
Malignant skin melanoma	Latitude 30 to 45 (proportion)	2	-1	2	-1
Malignant skin melanoma	Latitude Over 45 (proportion)	2	-1	2	-1
Malignant skin melanoma	Latitude Under 15 (proportion)	2	-1	2	-1
Malignant skin melanoma	Education (years per capita)	3	-1	3	-1
Malignant skin melanoma	LDI (I$ per capita)	3	-1	3	-1
Malignant skin melanoma	Socio-demographic Index	3	1	3	1

Mesothelioma	Age- and sex-specific SEV for Occupational asbestos	1	1	1	1
Mesothelioma	Age-standardized SEV for Occupational asbestos	1	1	1	1
Mesothelioma	Asbestos consumption (metric tons per year per capita)	1	1	1	1
Mesothelioma	Smoking Prevalence	1	1	1	1
Mesothelioma	Cumulative Cigarettes (5 Years)	2	1	2	1
Mesothelioma	Gold production (binary)	2	1	2	1
Mesothelioma	Healthcare access and quality index	2	-1	2	-1
Mesothelioma	Indoor Air Pollution (All Cooking Fuels)	2	1	2	1
Mesothelioma	Population Density (over 1000 ppl/sqkm, proportion)	2	1	2	1
Mesothelioma	Education (years per capita)	3	-1	3	-1
Mesothelioma	LDI (I$ per capita)	3	-1	3	-1
Mesothelioma	Socio-demographic Index	3	1	3	1
Multiple myeloma	Liters of alcohol consumed per capita	1	1	1	1
Multiple myeloma	Smoking Prevalence	1	1	1	1
Multiple myeloma	Tobacco (cigarettes per capita)	1	1	1	1
Multiple myeloma	Age- and sex-specific SEV for High red meat	2	1	2	1
Multiple myeloma	Age- and sex-specific SEV for Low fruit	2	1	2	1
Multiple myeloma	Age- and sex-specific SEV for Low vegetables	2	1	2	1
Multiple myeloma	Healthcare access and quality index	2	-1	2	-1
Multiple myeloma	Improved Water Source (proportion with access)	2	-1	2	-1
Multiple myeloma	Mean BMI	2	1	2	1
Multiple myeloma	Sanitation (proportion with access)	2	-1	2	-1
Multiple myeloma	Education (years per capita)	3	-1	3	-1
Multiple myeloma	LDI (I$ per capita)	3	1	3	1
Multiple myeloma	Socio-demographic Index	3	1	3	1
Myelodysplastic, myeloproliferative, and other hematopoietic neoplasms	Log-transformed age-standardized SEV scalar: Leukemia [Global, male]	1	1	1	1
Myelodysplastic, myeloproliferative, and other hematopoietic neoplasms	Log-transformed SEV scalar: Leukemia [Global, male]	1	1	1	1
Myelodysplastic, myeloproliferative, and other hematopoietic neoplasms	Cumulative Cigarettes (10 Years)	2	1	2	1
Myelodysplastic, myeloproliferative, and other hematopoietic neoplasms	Cumulative Cigarettes (15 Years)	2	1	2	1
Myelodysplastic, myeloproliferative, and other hematopoietic neoplasms	Cumulative Cigarettes (20 Years)	2	1	2	1
Myelodysplastic, myeloproliferative, and other hematopoietic neoplasms	Cumulative Cigarettes (5 Years)	2	1	2	1
Myelodysplastic, myeloproliferative, and other hematopoietic neoplasms	Healthcare access and quality index	2	-1	2	-1
Myelodysplastic, myeloproliferative, and other hematopoietic neoplasms	Liters of alcohol consumed per capita	2	1	2	1
Myelodysplastic, myeloproliferative, and other hematopoietic neoplasms	Smoking Prevalence	2	1	2	1
Myelodysplastic, myeloproliferative, and other hematopoietic neoplasms	Tobacco (cigarettes per capita)	2	1	2	1
Myelodysplastic, myeloproliferative, and other hematopoietic neoplasms	Education (years per capita)	3	-1	3	-1
Myelodysplastic, myeloproliferative, and other hematopoietic neoplasms	LDI (I$ per capita)	3	1	3	1
Myelodysplastic, myeloproliferative, and other hematopoietic neoplasms	Socio-demographic Index	3	1	3	1
Myelodysplastic, myeloproliferative, and other hematopoietic neoplasms	Log-transformed age-standardized SEV scalar: Leukemia [Data Rich]	1	1	NA	NA
Myelodysplastic, myeloproliferative, and other hematopoietic neoplasms	Log-transformed age-standardized SEV scalar: Leukemia [Global, female]	1	1	NA	NA
Myelodysplastic, myeloproliferative, and other hematopoietic neoplasms	Log-transformed SEV scalar: Leukemia [Data Rich]	1	1	NA	NA
Myelodysplastic, myeloproliferative, and other hematopoietic neoplasms	Log-transformed SEV scalar: Leukemia [Global, female]	1	1	NA	NA
Nasopharynx cancer	Cumulative Cigarettes (10 Years)	1	1	1	1
Nasopharynx cancer	Cumulative Cigarettes (20 Years)	1	1	1	1
Nasopharynx cancer	Liters of alcohol consumed per capita	1	1	1	1
Nasopharynx cancer	Tobacco (cigarettes per capita)	1	1	1	1
Nasopharynx cancer	Age- and sex-specific SEV for Low vegetables	2	1	2	1
Nasopharynx cancer	Healthcare access and quality index	2	-1	2	-1
Nasopharynx cancer	Population Density (over 1000 ppl/sqkm, proportion)	2	1	2	1

Nasopharynx cancer	Age- and sex-specific SEV for Low fruit	3	1	3	1
Nasopharynx cancer	Education (years per capita)	3	-1	3	-1
Nasopharynx cancer	LDI (I$ per capita)	3	-1	3	-1
Nasopharynx cancer	Socio-demographic Index	3	1	3	1
Nasopharynx cancer	Log-transformed SEV scalar: Nasoph C	1	1	NA	NA
Neuroblastoma and other peripheral nervous cell tumors	Education (years per capita)	3	-1	3	-1
Neuroblastoma and other peripheral nervous cell tumors	Health worker density	3	-1	3	-1
Neuroblastoma and other peripheral nervous cell tumors	Healthcare access and quality index	3	-1	3	-1
Neuroblastoma and other peripheral nervous cell tumors	LDI (I$ per capita)	3	1	3	1
Neuroblastoma and other peripheral nervous cell tumors	Maternal care and immunization	3	-1	3	-1
Neuroblastoma and other peripheral nervous cell tumors	Smoking Prevalence	3	1	3	1
Neuroblastoma and other peripheral nervous cell tumors	Socio-demographic Index	3	1	3	1
Neuroblastoma and other peripheral nervous cell tumors	Universal health coverage	3	-1	3	-1
Non-Hodgkin lymphoma	Cumulative Cigarettes (10 Years) [Global, female]	2	1	1	1
Non-Hodgkin lymphoma	Cumulative Cigarettes (15 Years) [Global, female]	2	1	1	1
Non-Hodgkin lymphoma	Cumulative Cigarettes (20 Years) [Global, female]	2	1	1	1
Non-Hodgkin lymphoma	Cumulative Cigarettes (5 Years) [Global, female]	2	1	1	1
Non-Hodgkin lymphoma	Hepatitis C Seroprevalence (anti-HCV) age standardized [Global, female]	NA	NA	1	1
Non-Hodgkin lymphoma	HIV age-standardized prevalence [Global, female]	NA	NA	1	1
Non-Hodgkin lymphoma	HIV Prevalence Unadjusted (proportion) [Global, female]	NA	NA	1	1
Non-Hodgkin lymphoma	Mean BMI [Global, female]	2	1	1	1
Non-Hodgkin lymphoma	Cumulative Cigarettes (10 Years) [Data Rich, female]	2	1	2	1
Non-Hodgkin lymphoma	Cumulative Cigarettes (10 Years) [male]	2	1	2	1
Non-Hodgkin lymphoma	Cumulative Cigarettes (15 Years) [Data Rich, female]	2	1	2	1
Non-Hodgkin lymphoma	Cumulative Cigarettes (15 Years) [male]	2	1	2	1
Non-Hodgkin lymphoma	Cumulative Cigarettes (20 Years) [Data Rich, female]	2	1	2	1
Non-Hodgkin lymphoma	Cumulative Cigarettes (20 Years) [male]	2	1	2	1
Non-Hodgkin lymphoma	Cumulative Cigarettes (5 Years) [Data Rich, female]	2	1	2	1
Non-Hodgkin lymphoma	Cumulative Cigarettes (5 Years) [male]	2	1	2	1
Non-Hodgkin lymphoma	Education (years per capita) [Global, female]	3	-1	2	-1
Non-Hodgkin lymphoma	Healthcare access and quality index	2	-1	2	-1
Non-Hodgkin lymphoma	LDI (I$ per capita) [Global, female]	3	-1	2	-1
Non-Hodgkin lymphoma	Liters of alcohol consumed per capita	2	1	2	1
Non-Hodgkin lymphoma	Mean BMI [Data Rich, female]	2	1	2	1
Non-Hodgkin lymphoma	Mean BMI [male]	2	1	2	1
Non-Hodgkin lymphoma	Socio-demographic Index [Global, female]	3	-1	2	-1
Non-Hodgkin lymphoma	Total Fertility Rate [Global, female]	3	-1	2	-1
Non-Hodgkin lymphoma	Universal health coverage	2	-1	2	-1
Non-Hodgkin lymphoma	Education (years per capita) [Data Rich, female]	3	-1	3	-1
Non-Hodgkin lymphoma	Education (years per capita) [male]	3	-1	3	-1
Non-Hodgkin lymphoma	LDI (I$ per capita) [Data Rich, female]	3	-1	3	-1
Non-Hodgkin lymphoma	LDI (I$ per capita) [male]	3	-1	3	-1
Non-Hodgkin lymphoma	Log-transformed age-standardized SEV scalar: HIV	3	1	3	1
Non-Hodgkin lymphoma	Log-transformed SEV scalar: HIV	3	1	3	1
Non-Hodgkin lymphoma	Socio-demographic Index [Data Rich, female]	3	-1	3	-1
Non-Hodgkin lymphoma	Socio-demographic Index [male]	3	-1	3	-1
Non-Hodgkin lymphoma	Total Fertility Rate [Data Rich, female]	3	-1	3	-1
Non-melanoma skin cancer	Cumulative Cigarettes (10 Years)	1	1	1	1
Non-melanoma skin cancer	Cumulative Cigarettes (15 Years)	1	1	1	1
Non-melanoma skin cancer	Cumulative Cigarettes (5 Years)	1	1	1	1
Non-melanoma skin cancer	Smoking Prevalence	1	1	1	1
Non-melanoma skin cancer	Average latitude	2	-1	2	-1
Non-melanoma skin cancer	Healthcare access and quality index	2	-1	2	-1
Non-melanoma skin cancer	Education (years per capita)	3	-1	3	-1
Non-melanoma skin cancer	LDI (I$ per capita)	3	-1	3	-1

Non-melanoma skin cancer	Socio-demographic Index	3	1	3	1
Non-melanoma skin cancer (squamous-cell carcinoma)	Cumulative Cigarettes (10 Years)	1	1	1	1
Non-melanoma skin cancer (squamous-cell carcinoma)	Cumulative Cigarettes (15 Years)	1	1	1	1
Non-melanoma skin cancer (squamous-cell carcinoma)	Cumulative Cigarettes (5 Years)	1	1	1	1
Non-melanoma skin cancer (squamous-cell carcinoma)	Smoking Prevalence	1	1	1	1
Non-melanoma skin cancer (squamous-cell carcinoma)	Average latitude	2	-1	2	-1
Non-melanoma skin cancer (squamous-cell carcinoma)	Healthcare access and quality index	2	-1	2	-1
Non-melanoma skin cancer (squamous-cell carcinoma)	Education (years per capita)	3	-1	3	-1
Non-melanoma skin cancer (squamous-cell carcinoma)	LDI (I$ per capita)	3	-1	3	-1
Non-melanoma skin cancer (squamous-cell carcinoma)	Socio-demographic Index	3	1	3	1
Other benign and in situ neoplasms	Cumulative Cigarettes (10 Years)	2	1	2	1
Other benign and in situ neoplasms	Healthcare access and quality index	2	-1	2	-1
Other benign and in situ neoplasms	Smoking Prevalence	2	1	2	1
Other benign and in situ neoplasms	Tobacco (cigarettes per capita)	2	1	2	1
Other benign and in situ neoplasms	Education (years per capita)	3	-1	3	-1
Other benign and in situ neoplasms	LDI (I$ per capita)	3	-1	3	-1
Other benign and in situ neoplasms	Liters of alcohol consumed per capita	3	1	3	1
Other benign and in situ neoplasms	Socio-demographic Index [Global, female]	3	-1	3	-1
Other benign and in situ neoplasms	Socio-demographic Index [male]	3	-1	3	-1
Other eye cancers	Age-standardized melanoma	2	1	2	1
Other eye cancers	Healthcare access and quality index	2	-1	2	-1
Other eye cancers	Universal health coverage	2	-1	2	-1
Other eye cancers	Education (years per capita)	3	-1	3	-1
Other eye cancers	LDI (I$ per capita)	3	-1	3	-1
Other eye cancers	Socio-demographic Index	3	-1	3	-1
Other leukemia	Liters of alcohol consumed per capita [Data Rich, female]	1	1	1	1
Other leukemia	Cumulative Cigarettes (10 Years)	2	1	2	1
Other leukemia	Cumulative Cigarettes (20 Years)	2	1	2	1
Other leukemia	Healthcare access and quality index	2	-1	2	-1
Other leukemia	Liters of alcohol consumed per capita [Global, female]	2	1	2	1
Other leukemia	Liters of alcohol consumed per capita [male]	2	1	2	1
Other leukemia	Mean BMI	2	1	2	1
Other leukemia	Tobacco (cigarettes per capita)	2	1	2	1
Other leukemia	Education (years per capita)	3	-1	3	-1
Other leukemia	LDI (I$ per capita)	3	1	3	1
Other leukemia	Socio-demographic Index	3	-1	3	-1
Other leukemia	Log-transformed age-standardized SEV scalar: Leukemia	1	1	NA	NA
Other leukemia	Log-transformed SEV scalar: Leukemia	1	1	NA	NA
Other malignant neoplasms	Smoking Prevalence	1	1	1	1
Other malignant neoplasms	Tobacco (cigarettes per capita)	1	1	1	1
Other malignant neoplasms	Age- and sex-specific SEV for Low fruit	2	1	2	1
Other malignant neoplasms	Age- and sex-specific SEV for Low nuts and seeds	2	1	2	1
Other malignant neoplasms	Age- and sex-specific SEV for Low vegetables	2	1	2	1
Other malignant neoplasms	Healthcare access and quality index	2	-1	2	-1
Other malignant neoplasms	pufa adjusted(percent)	2	-1	2	-1
Other malignant neoplasms	Education (years per capita)	3	-1	3	-1
Other malignant neoplasms	LDI (I$ per capita)	3	1	3	1
Other malignant neoplasms	Socio-demographic Index	3	1	3	1
Other neoplasms	Cumulative Cigarettes (10 Years) [Data Rich]	NA	NA	2	1
Other neoplasms	Liters of alcohol consumed per capita [Data Rich]	NA	NA	2	1
Other neoplasms	Smoking Prevalence [Data Rich]	NA	NA	2	1
Other neoplasms	Tobacco (cigarettes per capita) [Data Rich]	NA	NA	2	1
Other neoplasms	Cumulative Cigarettes (10 Years) [Global]	NA	NA	3	1
Other neoplasms	Education (years per capita)	NA	NA	3	-1
Other neoplasms	Health worker density [Data Rich]	NA	NA	3	-1
Other neoplasms	Healthcare access and quality index	NA	NA	3	-1
Other neoplasms	LDI (I$ per capita)	NA	NA	3	1
Other neoplasms	Liters of alcohol consumed per capita [Global]	NA	NA	3	1

Other neoplasms	Log-transformed age-standardized SEV scalar: Leukemia	NA	NA	3	1
Other neoplasms	Log-transformed SEV scalar: Leukemia	NA	NA	3	1
Other neoplasms	Low-Density Lipoprotein (mmol/L)	NA	NA	3	1
Other neoplasms	Maternal care and immunization	NA	NA	3	-1
Other neoplasms	Mean BMI [Data Rich]	NA	NA	3	1
Other neoplasms	Smoking Prevalence [Global]	NA	NA	3	1
Other neoplasms	Socio-demographic Index	NA	NA	3	-1
Other neoplasms	Tobacco (cigarettes per capita) [Global]	NA	NA	3	1
Other neoplasms	Universal health coverage	NA	NA	3	-1
Other non-Hodgkin lymphoma	Cumulative Cigarettes (10 Years)	2	1	2	1
Other non-Hodgkin lymphoma	Cumulative Cigarettes (15 Years)	2	1	2	1
Other non-Hodgkin lymphoma	Cumulative Cigarettes (20 Years)	2	1	2	1
Other non-Hodgkin lymphoma	Cumulative Cigarettes (5 Years)	2	1	2	1
Other non-Hodgkin lymphoma	Healthcare access and quality index	2	-1	2	-1
Other non-Hodgkin lymphoma	Liters of alcohol consumed per capita	2	1	2	1
Other non-Hodgkin lymphoma	Mean BMI	2	1	2	1
Other non-Hodgkin lymphoma	Universal health coverage	2	-1	2	-1
Other non-Hodgkin lymphoma	Education (years per capita)	3	-1	3	-1
Other non-Hodgkin lymphoma	LDI (I$ per capita)	3	-1	3	-1
Other non-Hodgkin lymphoma	Log-transformed age-standardized SEV scalar: HIV	3	1	3	1
Other non-Hodgkin lymphoma	Log-transformed SEV scalar: HIV	3	1	3	1
Other non-Hodgkin lymphoma	Socio-demographic Index	3	-1	3	-1
Other non-Hodgkin lymphoma	Total Fertility Rate [female]	3	-1	3	-1
Other pharynx cancer	Liters of alcohol consumed per capita	1	1	1	1
Other pharynx cancer	Smoking Prevalence	1	1	1	1
Other pharynx cancer	Age- and sex-specific SEV for Low fruit	2	1	2	1
Other pharynx cancer	Age- and sex-specific SEV for Low vegetables	2	1	2	1
Other pharynx cancer	Cumulative Cigarettes (5 Years)	2	1	2	1
Other pharynx cancer	Healthcare access and quality index	2	-1	2	-1
Other pharynx cancer	Population Density (over 1000 ppl/sqkm, proportion)	2	1	2	1
Other pharynx cancer	Population Density (under 150 ppl/sqkm, proportion)	2	1	2	1
Other pharynx cancer	Education (years per capita)	3	-1	3	-1
Other pharynx cancer	LDI (I$ per capita)	3	1	3	1
Other pharynx cancer	Socio-demographic Index	3	1	3	1
Other pharynx cancer	Log-transformed SEV scalar: Oth Phar C	1	1	NA	NA
Ovarian cancer	Liters of alcohol consumed per capita [female]	1	1	1	1
Ovarian cancer	Asbestos consumption (metric tons per year per capita) [female]	2	1	2	1
Ovarian cancer	Contraception (Modern) Prevalence (proportion) [female]	2	-1	2	-1
Ovarian cancer	Cumulative Cigarettes (10 Years) [female]	2	1	2	1
Ovarian cancer	Cumulative Cigarettes (20 Years) [female]	2	1	2	1
Ovarian cancer	Diabetes Age-Standardized Prevalence (proportion) [female]	2	1	2	1
Ovarian cancer	energy unadjusted(kcal) [female]	2	1	2	1
Ovarian cancer	Healthcare access and quality index [female]	2	-1	2	-1
Ovarian cancer	Mean BMI [female]	2	1	2	1
Ovarian cancer	Smoking Prevalence [female]	2	1	2	1
Ovarian cancer	Total Fertility Rate [female]	2	-1	2	-1
Ovarian cancer	Age- and sex-specific SEV for Low fruit [female]	3	1	3	1
Ovarian cancer	Age- and sex-specific SEV for Low vegetables [female]	3	1	3	1
Ovarian cancer	Education (years per capita) [female]	3	-1	3	-1
Ovarian cancer	LDI (I$ per capita) [female]	3	-1	3	-1
Ovarian cancer	Socio-demographic Index [female]	3	1	3	1
Ovarian cancer	Log-transformed SEV scalar: Ovary C [female]	1	1	NA	NA
Pancreatic cancer	Cumulative Cigarettes (10 Years)	1	1	1	1
Pancreatic cancer	Cumulative Cigarettes (20 Years)	1	1	1	1
Pancreatic cancer	Mean BMI	1	1	1	1
Pancreatic cancer	Tobacco (cigarettes per capita)	1	1	1	1
Pancreatic cancer	Age- and sex-specific SEV for High red meat	2	1	2	1
Pancreatic cancer	Diabetes Age-Standardized Prevalence (proportion)	2	1	2	1
Pancreatic cancer	Diabetes Fasting Plasma Glucose (mmol/L), age-standardized 25+	2	1	2	1
Pancreatic cancer	energy unadjusted(kcal)	2	1	2	1

Pancreatic cancer	Healthcare access and quality index	2	-1	2	-1
Pancreatic cancer	Liters of alcohol consumed per capita	2	1	2	1
Pancreatic cancer	Age- and sex-specific SEV for Low fruit	3	1	3	1
Pancreatic cancer	Age- and sex-specific SEV for Low vegetables	3	1	3	1
Pancreatic cancer	Education (years per capita)	3	-1	3	-1
Pancreatic cancer	LDI (I$ per capita)	3	1	3	1
Pancreatic cancer	Socio-demographic Index	3	1	3	1
Pancreatic cancer	Log-transformed SEV scalar: Pancreas C	1	1	NA	NA
Prostate cancer	Log-transformed SEV scalar: Prostate C [male]	1	1	1	1
Prostate cancer	Healthcare access and quality index [male]	2	-1	2	-1
Prostate cancer	Smoking Prevalence [male]	2	1	2	1
Prostate cancer	Education (years per capita) [male]	3	-1	3	-1
Prostate cancer	LDI (I$ per capita) [male]	3	-1	3	-1
Prostate cancer	Socio-demographic Index [male]	3	1	3	1
Retinoblastoma	Healthcare access and quality index	2	-1	2	-1
Retinoblastoma	Universal health coverage	2	-1	2	-1
Retinoblastoma	Education (years per capita)	3	-1	3	-1
Retinoblastoma	LDI (I$ per capita)	3	-1	3	-1
Retinoblastoma	Maternal care and immunization	3	-1	3	-1
Retinoblastoma	Socio-demographic Index	3	-1	3	-1
Soft tissue and other extraosseous sarcomas	Log-transformed age-standardized SEV scalar: HIV/AIDS+STIs [Data Rich, female]	NA	NA	1	1
Soft tissue and other extraosseous sarcomas	Log-transformed SEV scalar: HIV/AIDS+STIs [Data Rich, female]	NA	NA	1	1
Soft tissue and other extraosseous sarcomas	Healthcare access and quality index	2	-1	2	-1
Soft tissue and other extraosseous sarcomas	LDI (I$ per capita)	2	-1	2	-1
Soft tissue and other extraosseous sarcomas	Socio-demographic Index	2	-1	2	-1
Soft tissue and other extraosseous sarcomas	Universal health coverage	2	-1	2	-1
Soft tissue and other extraosseous sarcomas	Education (years per capita)	3	-1	3	-1
Soft tissue and other extraosseous sarcomas	Liters of alcohol consumed per capita	3	1	3	1
Soft tissue and other extraosseous sarcomas	Log-transformed age-standardized SEV scalar: HIV	3	1	3	1
Soft tissue and other extraosseous sarcomas	Log-transformed SEV scalar: HIV	3	1	3	1
Soft tissue and other extraosseous sarcomas	Maternal care and immunization	3	-1	3	-1
Stomach cancer	Diet high in sodium	1	1	1	1
Stomach cancer	Tobacco (cigarettes per capita)	1	1	1	1
Stomach cancer	Age- and sex-specific SEV for Unsafe sanitation	2	1	2	1
Stomach cancer	Age- and sex-specific SEV for Unsafe water	2	1	2	1
Stomach cancer	Cumulative Cigarettes (20 Years)	2	1	2	1
Stomach cancer	Healthcare access and quality index	2	-1	2	-1
Stomach cancer	Improved Water Source (proportion with access)	2	-1	2	-1
Stomach cancer	Mean BMI	2	1	2	1
Stomach cancer	Sanitation (proportion with access)	2	-1	2	-1
Stomach cancer	Age- and sex-specific SEV for Low fruit	3	1	3	1
Stomach cancer	Age- and sex-specific SEV for Low vegetables	3	1	3	1
Stomach cancer	Education (years per capita)	3	-1	3	-1
Stomach cancer	LDI (I$ per capita)	3	1	3	1
Stomach cancer	Socio-demographic Index	3	-1	3	-1
Stomach cancer	Log-transformed SEV scalar: Stomach C	1	1	NA	NA
Testicular cancer	Age- and sex-specific SEV for Low fruit [male]	2	1	2	1
Testicular cancer	Age- and sex-specific SEV for Low vegetables [male]	2	1	2	1
Testicular cancer	Cumulative Cigarettes (10 Years) [male]	2	1	2	1
Testicular cancer	Cumulative Cigarettes (15 Years) [male]	2	1	2	1
Testicular cancer	Cumulative Cigarettes (20 Years) [male]	2	1	2	1
Testicular cancer	Cumulative Cigarettes (5 Years) [male]	2	1	2	1
Testicular cancer	Healthcare access and quality index [male]	2	-1	2	-1
Testicular cancer	Smoking Prevalence [male]	2	1	2	1
Testicular cancer	Tobacco (cigarettes per capita) [male]	2	1	2	1
Testicular cancer	Education (years per capita) [male]	3	-1	3	-1
Testicular cancer	LDI (I$ per capita) [male]	3	1	3	1
Testicular cancer	Socio-demographic Index [male]	3	1	3	1

Thyroid cancer	Liters of alcohol consumed per capita	1	1	1	1
Thyroid cancer	Age- and sex-specific SEV for High red meat	2	1	2	1
Thyroid cancer	Age- and sex-specific SEV for Low vegetables	2	1	2	1
Thyroid cancer	Healthcare access and quality index	2	-1	2	-1
Thyroid cancer	Mean BMI	2	1	2	1
Thyroid cancer	Tobacco (cigarettes per capita)	2	1	2	1
Thyroid cancer	Age- and sex-specific SEV for Low fruit	3	1	3	1
Thyroid cancer	Cumulative Cigarettes (10 Years) [Global, female]	NA	NA	3	1
Thyroid cancer	Cumulative Cigarettes (15 Years) [Global, female]	NA	NA	3	1
Thyroid cancer	Cumulative Cigarettes (20 Years) [Global, female]	NA	NA	3	1
Thyroid cancer	Cumulative Cigarettes (5 Years) [Global, female]	NA	NA	3	1
Thyroid cancer	Diabetes Fasting Plasma Glucose (mmol/L), age-standardized 25+ [Global, female]	NA	NA	3	1
Thyroid cancer	Education (years per capita)	3	-1	3	-1
Thyroid cancer	Improved Water Source (proportion with access)	3	-1	3	-1
Thyroid cancer	LDI (I$ per capita)	3	1	3	1
Thyroid cancer	Outdoor Air Pollution (PM2.5) [Global, female]	NA	NA	3	1
Thyroid cancer	Sanitation (proportion with access)	3	-1	3	-1
Thyroid cancer	Smoking Prevalence [Global, female]	NA	NA	3	1
Thyroid cancer	Socio-demographic Index	3	1	3	1
Thyroid cancer	Total Physical Activity (MET-min/week), Age-specific [Global, female]	NA	NA	3	-1
Thyroid cancer	Log-transformed SEV scalar: Thyroid C	1	1	NA	NA
Tracheal, bronchus, and lung cancer	Asbestos consumption (metric tons per year per capita)	1	1	1	1
Tracheal, bronchus, and lung cancer	Smoking Prevalence	1	1	1	1
Tracheal, bronchus, and lung cancer	Cumulative Cigarettes (10 Years)	2	1	2	1
Tracheal, bronchus, and lung cancer	Cumulative Cigarettes (20 Years)	2	1	2	1
Tracheal, bronchus, and lung cancer	Diabetes Fasting Plasma Glucose (mmol/L), age-standardized 25+	2	1	2	1
Tracheal, bronchus, and lung cancer	Healthcare access and quality index	2	-1	2	-1
Tracheal, bronchus, and lung cancer	Indoor Air Pollution (All Cooking Fuels)	2	1	2	1
Tracheal, bronchus, and lung cancer	Outdoor Air Pollution (PM2.5)	2	1	2	1
Tracheal, bronchus, and lung cancer	Residential radon	2	1	2	1
Tracheal, bronchus, and lung cancer	Secondhand smoke	2	1	2	1
Tracheal, bronchus, and lung cancer	Education (years per capita)	3	-1	3	-1
Tracheal, bronchus, and lung cancer	LDI (I$ per capita)	3	1	3	1
Tracheal, bronchus, and lung cancer	Socio-demographic Index	3	1	3	1
Tracheal, bronchus, and lung cancer	Log-transformed age-standardized SEV scalar: Lung C	1	1	NA	NA
Tracheal, bronchus, and lung cancer	Log-transformed SEV scalar: Lung C	1	1	NA	NA
Uterine cancer	Mean BMI [female]	1	1	1	1
Uterine cancer	Age- and sex-specific SEV for Low fruit [female]	2	1	2	1
Uterine cancer	Age- and sex-specific SEV for Low vegetables [female]	2	1	2	1
Uterine cancer	Cumulative Cigarettes (10 Years) [female]	2	1	2	1
Uterine cancer	Cumulative Cigarettes (5 Years) [female]	2	1	2	1
Uterine cancer	Diabetes Age-Standardized Prevalence (proportion) [female]	2	1	2	1
Uterine cancer	Healthcare access and quality index [female]	2	-1	2	-1
Uterine cancer	Smoking Prevalence [female]	2	1	2	1
Uterine cancer	Tobacco (cigarettes per capita) [female]	2	1	2	1
Uterine cancer	Total Fertility Rate [female]	2	-1	2	-1
Uterine cancer	Education (years per capita) [female]	3	-1	3	-1
Uterine cancer	LDI (I$ per capita) [female]	3	1	3	1
Uterine cancer	Socio-demographic Index [female]	3	1	3	1
Uterine cancer	Log-transformed SEV scalar: Uterus C [female]	1	1	NA	NA

Abbreviations: BMI, body mass index; GBD, Global Burden of Diseases, Injuries, and Risk Factors 2023 Study; HBsAg, hepatitis B surface antigen; HCV, hepatitis C virus; LDI, lag-distributed income; MET, metabolic equivalent of task; mmHg, millimetres of mercury; mmol/L, millimoles per litre; NA, not applicable because the covariate was not used in that GBD round; PM2.5, particulate matter ≤ 2.5 micrometers; ppl/sqkm, people per square kilometre; PUFA, polyunsaturated fatty acid; SEV, summary exposure value: for definitions and calculations, please see the Supplementary Appendix 1 to "Global burden of 292 causes of death in 204 countries and territories and 660 subnational locations, 1990–2023: a systematic analysis for the Global Burden of Disease Study 2023. Lancet (in review)";[2] covariates with "C" following a cancer site name refer to a cancer site (e.g., uterus C = uterus cancer) and were shortened due to space limitations in covariate names.Causes are listed in alphabetical order. The "Direction" column refers to the direction of the association for that covariate. Cells including "[Data Rich]" indicate that the specified covariate was only included as a potential covariate for submodels in the data rich ensemble

model (i.e., the ensemble model that was restricted to countries determined to have high quality vital registration system data). Cells including "[Global]" indicate that the specified covariate was only included as a potential covariate for submodels in the global ensemble model (i.e., the ensemble model that included all available data). Cells that do not include "[Data Rich]" or "[Global]" indicate that the specified covariate was included as a potential covariate for submodels in both data rich and global ensemble models for the respective GBD cause. For Burkitt lymphoma an additional model limited to "[Sub-Saharan Africa]" was run that additionally utilized the "Latitude Under 15 (proportion)" covariate. Final CODEm models are the hybridized result of data rich and global models. Cells including "NA" indicate that the covariate was not included as an option for CODEm to select in modeling for that cause and GBD round.

To generate an ensemble model, CODEm iteratively generates submodels that hierarchically evaluate all plausible relationships between covariates and the response variable in a forward-selection model building process. Three additive components of data variance are used in CODEm: sampling variance, non-sampling variance, and garbage code redistribution variance. Similar to MIR estimation, CODEm smooths estimates over time, age group, and geography. The weighting hyperparameters for CODEm were adjusted in GBD 2021 to decrease temporal smoothing so that the model estimates would more closely follow short-term data trends, which were maintained for GBD 2023.

Model performance of all submodels are evaluated through out-of-sample predictive validity tests (Appendix 1 Table 9). Ensemble models are constructed from the individual submodels, with the contribution of individual submodels to the ensemble model weighted by the basis of their predictive validity ranking. The final ensemble combines 1000 total draws from these individual component models, from which a mean estimate and a 95% uncertainty interval are calculated. The 95% uncertainty interval represents the 0.025 and 0.975 quantiles of the draws.

Appendix Table 9: Results for CODEm model performance testing

Cause	Sex	RMSE in	RMSE out	Trend in	Trend out	Coverage in	Coverage out
Acute lymphoid leukemia [Data Rich]	Male	0.135481	0.463638	0.105516	0.189389	0.999659	0.999032
Acute lymphoid leukemia [Data Rich]	Female	0.180594	0.479686	0.134838	0.211869	0.999234	0.998594
Acute lymphoid leukemia [Global]	Male	0.719389	0.870941	0.446229	0.440699	0.994479	0.989887
Acute lymphoid leukemia [Global]	Female	0.790922	0.955750	0.482954	0.483399	0.993917	0.988924
Acute myeloid leukemia [Data Rich]	Male	0.169828	0.452943	0.135193	0.236643	0.998800	0.997516
Acute myeloid leukemia [Data Rich]	Female	0.163374	0.490194	0.137626	0.219027	0.999106	0.998357
Acute myeloid leukemia [Global]	Male	0.766198	0.961836	0.495325	0.517377	0.993618	0.988700
Acute myeloid leukemia [Global]	Female	0.773685	0.880577	0.505279	0.496642	0.993722	0.988590
Bladder cancer [Data Rich]	Male	0.109379	0.220478	0.087304	0.150612	0.999963	0.999124
Bladder cancer [Data Rich]	Female	0.110214	0.224430	0.086542	0.154471	0.999996	0.999533
Bladder cancer [Global]	Male	0.190812	0.337974	0.138913	0.144894	0.999718	0.993357
Bladder cancer [Global]	Female	0.204972	0.364546	0.143286	0.144742	0.999563	0.996185
Brain and central nervous system cancer [Data Rich]	Male	0.106245	0.249195	0.082003	0.142122	0.999989	0.999537
Brain and central nervous system cancer [Data Rich]	Female	0.106741	0.242175	0.081527	0.138448	0.999988	0.999702
Brain and central nervous system cancer [Global]	Male	0.231154	0.368562	0.135731	0.144909	0.999528	0.995385
Brain and central nervous system cancer [Global]	Female	0.248084	0.389178	0.135020	0.143105	0.999748	0.995475
Breast cancer [Data Rich]	Male	0.170411	0.413502	0.118370	0.220053	0.999643	0.999032
Breast cancer [Data Rich]	Female	0.103919	0.200364	0.083831	0.145563	0.999900	0.998111
Breast cancer [Global]	Male	0.368098	0.576575	0.225141	0.241821	0.998400	0.992913
Breast cancer [Global]	Female	0.193996	0.311076	0.131387	0.136746	0.999565	0.989680
Burkitt lymphoma [Data Rich]	Male	0.466183	0.834519	0.170345	0.227413	0.999967	0.999704
Burkitt lymphoma [Data Rich]	Female	0.391725	0.665823	0.171428	0.208555	0.999956	0.999699
Burkitt lymphoma [Global]	Male	0.390820	0.674234	0.191013	0.208934	0.999758	0.994031
Burkitt lymphoma [Global]	Female	0.351273	0.585383	0.202228	0.219175	0.999612	0.996616
Burkitt lymphoma [Sub-Saharan Africa]	Male	0.431241	0.858961	0.144099	0.126331	1.000000	0.974164
Burkitt lymphoma [Sub-Saharan Africa]	Female	0.372243	0.817144	0.159804	0.149366	1.000000	0.985562
Cervical cancer [Data Rich]	Female	0.108432	0.207446	0.085186	0.140129	0.999928	0.999720
Cervical cancer [Global]	Female	0.226963	0.361748	0.135640	0.134617	0.999840	0.994699
Chronic lymphoid leukemia [Data Rich]	Male	0.120403	0.337014	0.090184	0.162402	0.999828	0.999031
Chronic lymphoid leukemia [Data Rich]	Female	0.164737	0.400663	0.101168	0.176263	0.999079	0.995816
Chronic lymphoid leukemia [Global]	Male	0.590201	0.827262	0.377039	0.356447	0.993887	0.972932
Chronic lymphoid leukemia [Global]	Female	0.618671	1.106080	0.399243	0.371074	0.991595	0.962327
Chronic myeloid leukemia [Data Rich]	Male	0.215304	0.541348	0.138551	0.151804	0.999558	0.995823
Chronic myeloid leukemia [Data Rich]	Female	0.267416	0.638104	0.178137	0.179673	0.998803	0.992418
Chronic myeloid leukemia [Global]	Male	0.656453	0.931691	0.430871	0.397322	0.992627	0.972491
Chronic myeloid leukemia [Global]	Female	0.668584	1.102660	0.443652	0.454454	0.992983	0.971523

Colon and rectum cancer [Data Rich]	Male	0.102382	0.196563	0.082948	0.142803	0.999952	0.998698
Colon and rectum cancer [Data Rich]	Female	0.101624	0.194717	0.081853	0.138301	0.999936	0.999178
Colon and rectum cancer [Global]	Male	0.181979	0.304793	0.132008	0.137589	0.999618	0.990014
Colon and rectum cancer [Global]	Female	0.183472	0.295703	0.128929	0.130385	0.999705	0.993043
Eye cancer [Data Rich]	Male	0.459360	0.583017	0.399533	0.432774	0.992234	0.987468
Eye cancer [Data Rich]	Female	0.475400	0.563803	0.388674	0.380285	0.995092	0.991359
Eye cancer [Global]	Male	0.775126	0.892215	0.596002	0.575336	0.982967	0.972589
Eye cancer [Global]	Female	0.723331	0.847318	0.558073	0.514045	0.987521	0.976409
Gallbladder and biliary tract cancer [Data Rich]	Male	0.112383	0.255644	0.090092	0.160773	0.999969	0.999665
Gallbladder and biliary tract cancer [Data Rich]	Female	0.107607	0.235082	0.085274	0.142841	0.999943	0.999498
Gallbladder and biliary tract cancer [Global]	Male	0.237787	0.389343	0.152392	0.155421	0.999620	0.992130
Gallbladder and biliary tract cancer [Global]	Female	0.220773	0.398287	0.143021	0.147336	0.999706	0.988298
Hodgkin lymphoma [Data Rich]	Male	0.127331	0.302892	0.094446	0.147418	0.999993	0.999553
Hodgkin lymphoma [Data Rich]	Female	0.127473	0.287692	0.097214	0.162810	0.999982	0.999638
Hodgkin lymphoma [Global]	Male	0.378141	0.564351	0.182844	0.190858	0.999548	0.992683
Hodgkin lymphoma [Global]	Female	0.345588	0.546777	0.192572	0.199598	0.999259	0.992104
Kidney cancer [Data Rich]	Male	0.114482	0.253644	0.083643	0.145175	0.999960	0.999473
Kidney cancer [Data Rich]	Female	0.116366	0.274597	0.088328	0.154888	0.999980	0.999506
Kidney cancer [Global]	Male	0.264911	0.391427	0.141427	0.142303	0.999607	0.995613
Kidney cancer [Global]	Female	0.265878	0.416286	0.163403	0.165861	0.999463	0.995170
Larynx cancer [Data Rich]	Male	0.112131	0.215508	0.086663	0.142168	0.999974	0.999464
Larynx cancer [Data Rich]	Female	0.124742	0.248042	0.094558	0.172496	0.999996	0.999721
Larynx cancer [Global]	Male	0.264148	0.431025	0.141242	0.141539	0.999835	0.986493
Larynx cancer [Global]	Female	0.279739	0.485046	0.166005	0.166224	0.999511	0.993116
Leukemia [Data Rich]	Male	0.106613	0.215902	0.082176	0.136548	0.999964	0.999561
Leukemia [Data Rich]	Female	0.105210	0.210900	0.081333	0.132873	0.999947	0.999622
Leukemia [Global]	Male	0.203844	0.326354	0.137403	0.142295	0.999801	0.995198
Leukemia [Global]	Female	0.200317	0.323824	0.167592	0.174761	0.999778	0.995637
Lip and oral cavity cancer [Data Rich]	Male	0.106154	0.220991	0.084476	0.148703	0.999992	0.999813
Lip and oral cavity cancer [Data Rich]	Female	0.113933	0.227005	0.090067	0.155626	0.999972	0.999788
Lip and oral cavity cancer [Global]	Male	0.191948	0.356422	0.141826	0.145084	0.999685	0.992354
Lip and oral cavity cancer [Global]	Female	0.210086	0.346367	0.146740	0.145013	0.999295	0.996952
Liver cancer [Data Rich]	Male	0.144966	0.319652	0.099647	0.176498	0.999749	0.998785
Liver cancer [Data Rich]	Female	0.141716	0.327648	0.101018	0.191755	0.999728	0.999328
Liver cancer [Global]	Male	0.245112	0.484242	0.175040	0.182245	0.998455	0.983559
Liver cancer [Global]	Female	0.249693	0.475806	0.176764	0.184676	0.999175	0.991433
Malignant neoplasm of bone and articular cartilage [Data Rich]	Male	0.138312	0.308907	0.107055	0.175280	0.999676	0.999534
Malignant neoplasm of bone and articular cartilage [Data Rich]	Female	0.159217	0.311106	0.112063	0.180995	0.999306	0.998923
Malignant neoplasm of bone and articular cartilage [Global]	Male	0.370542	0.542159	0.249696	0.263489	0.996815	0.991717
Malignant neoplasm of bone and articular cartilage [Global]	Female	0.364173	0.531808	0.261305	0.278359	0.996778	0.991905
Malignant skin melanoma [Data Rich]	Male	0.122556	0.267620	0.099651	0.186017	1.000000	0.999687
Malignant skin melanoma [Data Rich]	Female	0.119241	0.243161	0.097586	0.172314	1.000000	0.999533
Malignant skin melanoma [Global]	Male	0.299861	0.463229	0.172150	0.185186	0.999322	0.993398
Malignant skin melanoma [Global]	Female	0.307316	0.450724	0.184846	0.197173	0.998573	0.995326
Mesothelioma [Data Rich]	Male	0.107512	0.245653	0.081855	0.158153	0.999801	0.999638
Mesothelioma [Data Rich]	Female	0.132178	0.333323	0.099184	0.202799	0.999791	0.999321
Mesothelioma [Global]	Male	0.232232	0.476443	0.167614	0.185103	0.999238	0.996228
Mesothelioma [Global]	Female	0.269168	0.486952	0.187308	0.203266	0.998642	0.994483
Multiple myeloma [Data Rich]	Male	0.114058	0.250626	0.088732	0.154023	0.999706	0.999392
Multiple myeloma [Data Rich]	Female	0.112628	0.237025	0.086864	0.147318	0.999751	0.999446
Multiple myeloma [Global]	Male	0.343418	0.469189	0.213918	0.238332	0.995776	0.989778
Multiple myeloma [Global]	Female	0.333348	0.495018	0.226211	0.256963	0.995565	0.991660
Myelodysplastic, myeloproliferative, and other hematopoietic neoplasms [Data Rich]	Male	0.220486	0.755094	0.154055	0.287115	0.999126	0.997587
Myelodysplastic, myeloproliferative, and other hematopoietic neoplasms [Data Rich]	Female	0.253049	0.778061	0.179280	0.269979	0.998065	0.995865
Myelodysplastic, myeloproliferative, and other hematopoietic neoplasms [Global]	Male	0.496742	0.784421	0.229034	0.215236	0.997628	0.984049
Myelodysplastic, myeloproliferative, and other hematopoietic neoplasms [Global]	Female	0.423138	0.756790	0.264428	0.228657	0.995662	0.982140
Nasopharynx cancer [Data Rich]	Male	0.150494	0.245569	0.108633	0.141666	0.999974	0.999866
Nasopharynx cancer [Data Rich]	Female	0.194994	0.283884	0.150123	0.198937	0.999551	0.998684
Nasopharynx cancer [Global]	Male	0.210411	0.456501	0.141814	0.150265	0.999728	0.989936
Nasopharynx cancer [Global]	Female	0.256854	0.470129	0.177206	0.186116	0.998741	0.994403
Neuroblastoma and other peripheral nervous cell tumors [Data Rich]	Male	0.203702	0.492419	0.154872	0.193364	0.999812	0.998918

Neuroblastoma and other peripheral nervous cell tumors [Data Rich]	Female	0.199297	0.456795	0.159890	0.162958	0.999591	0.999101
Neuroblastoma and other peripheral nervous cell tumors [Global]	Male	0.353595	0.614512	0.191045	0.208292	0.999274	0.992110
Neuroblastoma and other peripheral nervous cell tumors [Global]	Female	0.330838	0.578397	0.201260	0.219564	0.998899	0.994188
Non-Hodgkin lymphoma [Data Rich]	Male	0.105394	0.234761	0.082952	0.146667	1.000000	0.999970
Non-Hodgkin lymphoma [Data Rich]	Female	0.104703	0.229364	0.081172	0.145022	0.999993	0.999928
Non-Hodgkin lymphoma [Global]	Male	0.190949	0.346145	0.134610	0.137606	0.999918	0.997036
Non-Hodgkin lymphoma [Global]	Female	0.191784	0.352306	0.132526	0.135686	0.999920	0.997294
Non-melanoma skin cancer (squamous-cell carcinoma) [Data Rich]	Male	0.128296	0.344166	0.102555	0.187950	0.999978	0.999651
Non-melanoma skin cancer (squamous-cell carcinoma) [Data Rich]	Female	0.133631	0.318362	0.105597	0.187114	0.999951	0.999329
Non-melanoma skin cancer (squamous-cell carcinoma) [Global]	Male	0.264009	0.461975	0.180063	0.196769	0.999326	0.992361
Non-melanoma skin cancer (squamous-cell carcinoma) [Global]	Female	0.288753	0.486236	0.194914	0.204409	0.998596	0.991904
Non-melanoma skin cancer [Data Rich]	Male	0.127866	0.330376	0.102425	0.193983	0.999973	0.999629
Non-melanoma skin cancer [Data Rich]	Female	0.133455	0.318463	0.105535	0.200934	0.999942	0.999360
Non-melanoma skin cancer [Global]	Male	0.262794	0.478371	0.178925	0.182617	0.999292	0.990931
Non-melanoma skin cancer [Global]	Female	0.292715	0.501940	0.194731	0.195459	0.998695	0.988794
Oesophageal cancer [Data Rich]	Male	0.104488	0.206529	0.083425	0.146638	0.999983	0.999418
Oesophageal cancer [Data Rich]	Female	0.105570	0.204588	0.084186	0.138567	0.999966	0.999698
Oesophageal cancer [Global]	Male	0.189482	0.411375	0.134752	0.138508	0.999669	0.979144
Oesophageal cancer [Global]	Female	0.209556	0.462769	0.142525	0.143535	0.999681	0.983586
Other benign and in situ neoplasms (internal) [Data Rich]	Male	0.173470	0.930644	0.132924	0.161528	1.000000	0.951625
Other benign and in situ neoplasms (internal) [Data Rich]	Female	0.177001	0.930205	0.136013	0.166695	1.000000	0.961521
Other benign and in situ neoplasms (internal) [Global]	Male	0.405486	1.092240	0.158754	0.165725	0.999936	0.946737
Other benign and in situ neoplasms (internal) [Global]	Female	0.500266	1.102950	0.163746	0.169571	0.999915	0.951758
Other eye cancers [Data Rich]	Male	0.385665	0.486305	0.326024	0.333868	0.998290	0.996412
Other eye cancers [Data Rich]	Female	0.444872	0.482741	0.364439	0.308936	0.996765	0.994444
Other eye cancers [Global]	Male	0.799740	0.880333	0.614344	0.550849	0.982181	0.971497
Other eye cancers [Global]	Female	0.797316	0.857998	0.587898	0.523737	0.984359	0.971545
Other leukemia [Data Rich]	Male	0.256995	0.673536	0.175553	0.194724	0.997837	0.991660
Other leukemia [Data Rich]	Female	0.300667	0.783131	0.168188	0.206487	0.998025	0.986487
Other leukemia [Global]	Male	0.606472	0.978830	0.372567	0.376176	0.993650	0.965175
Other leukemia [Global]	Female	0.751294	1.117750	0.378459	0.343876	0.994916	0.973148
Other malignant neoplasms [Data Rich]	Male	0.107332	0.246646	0.082786	0.128328	0.999963	0.999736
Other malignant neoplasms [Data Rich]	Female	0.110662	0.227933	0.087551	0.134600	0.999901	0.999567
Other malignant neoplasms [Global]	Male	0.231306	0.369000	0.148585	0.149545	0.999738	0.995449
Other malignant neoplasms [Global]	Female	0.203559	0.345900	0.142419	0.144959	0.999627	0.996150
Other neoplasms [Data Rich]	Male	0.166039	0.697391	0.119075	0.208542	0.999716	0.997470
Other neoplasms [Data Rich]	Female	0.168986	0.658211	0.104485	0.196913	0.999842	0.997208
Other neoplasms [Global]	Male	0.308879	0.611955	0.194242	0.179756	0.999312	0.985134
Other neoplasms [Global]	Female	0.268577	0.616986	0.160495	0.172151	0.999509	0.985031
Other non-Hodgkin lymphoma [Data Rich]	Male	0.109020	0.334036	0.084299	0.138756	0.999783	0.999675
Other non-Hodgkin lymphoma [Data Rich]	Female	0.110091	0.330631	0.084041	0.140046	0.999826	0.999794
Other non-Hodgkin lymphoma [Global]	Male	0.190702	0.354253	0.131009	0.134746	0.999810	0.997599
Other non-Hodgkin lymphoma [Global]	Female	0.259966	0.366310	0.130917	0.135611	0.999867	0.998544
Other pharynx cancer [Data Rich]	Male	0.116233	0.261713	0.086639	0.166137	0.999991	0.999631
Other pharynx cancer [Data Rich]	Female	0.142180	0.278281	0.092664	0.167379	0.999987	0.998751
Other pharynx cancer [Global]	Male	0.244116	0.467720	0.165441	0.174073	0.999216	0.989089
Other pharynx cancer [Global]	Female	0.296219	0.461091	0.174507	0.177810	0.998817	0.994397
Ovarian cancer [Data Rich]	Female	0.109948	0.231790	0.089617	0.156975	0.999946	0.999143
Ovarian cancer [Global]	Female	0.207552	0.330520	0.143844	0.154997	0.999641	0.992475
Pancreatic cancer [Data Rich]	Male	0.109028	0.228510	0.086036	0.153908	0.999942	0.998993
Pancreatic cancer [Data Rich]	Female	0.110693	0.225541	0.087227	0.151479	0.999938	0.999180
Pancreatic cancer [Global]	Male	0.184295	0.316478	0.136218	0.142463	0.999558	0.993319
Pancreatic cancer [Global]	Female	0.190464	0.333813	0.140568	0.147986	0.999488	0.994875
Prostate cancer [Data Rich]	Male	0.106149	0.223300	0.083378	0.148713	0.999953	0.998903
Prostate cancer [Global]	Male	0.238860	0.366050	0.139600	0.141806	0.999619	0.989633
Retinoblastoma [Data Rich]	Male	1.026270	1.272100	0.896494	0.928034	0.969049	0.946114
Retinoblastoma [Data Rich]	Female	1.053480	1.351740	0.927582	1.003110	0.965148	0.939515
Retinoblastoma [Global]	Male	1.234630	1.542900	0.957585	0.912189	0.971115	0.939697
Retinoblastoma [Global]	Female	1.232940	1.606220	0.980918	0.978727	0.971146	0.939139

Soft tissue and other extraosseous sarcomas [Data Rich]	Male	0.127657	0.344045	0.095736	0.163252	0.999874	0.999586
Soft tissue and other extraosseous sarcomas [Data Rich]	Female	0.126818	0.332669	0.099169	0.166035	0.999936	0.999405
Soft tissue and other extraosseous sarcomas [Global]	Male	0.281554	0.435444	0.157705	0.155080	0.998658	0.995772
Soft tissue and other extraosseous sarcomas [Global]	Female	0.277257	0.437728	0.167640	0.155394	0.998760	0.996011
Stomach cancer [Data Rich]	Male	0.103341	0.194053	0.083023	0.129781	0.999968	0.999217
Stomach cancer [Data Rich]	Female	0.105351	0.195219	0.085224	0.131040	0.999964	0.999066
Stomach cancer [Global]	Male	0.189562	0.358729	0.128869	0.132722	0.999613	0.981992
Stomach cancer [Global]	Female	0.178159	0.341786	0.130111	0.135141	0.999676	0.988480
Testicular cancer [Data Rich]	Male	0.128047	0.286225	0.105879	0.168685	0.999988	0.999574
Testicular cancer [Global]	Male	0.244456	0.441864	0.181654	0.176253	0.999152	0.995152
Thyroid cancer [Data Rich]	Male	0.115504	0.253887	0.088106	0.157519	0.999996	0.999843
Thyroid cancer [Data Rich]	Female	0.114199	0.238048	0.086482	0.147647	0.999996	0.999823
Thyroid cancer [Global]	Male	0.276030	0.389446	0.160503	0.167478	0.999390	0.998050
Thyroid cancer [Global]	Female	0.213996	0.355537	0.151245	0.152611	0.999503	0.998121
Tracheal, bronchus, and lung cancer [Data Rich]	Male	0.100398	0.195328	0.079817	0.132604	0.999912	0.997113
Tracheal, bronchus, and lung cancer [Data Rich]	Female	0.108383	0.215081	0.085814	0.156530	0.999972	0.998889
Tracheal, bronchus, and lung cancer [Global]	Male	0.193200	0.363302	0.124202	0.132593	0.999498	0.966618
Tracheal, bronchus, and lung cancer [Global]	Female	0.186812	0.363213	0.129272	0.136561	0.999743	0.978948
Uterine cancer [Data Rich]	Female	0.102469	0.212839	0.080423	0.135373	0.999987	0.999833
Uterine cancer [Global]	Female	0.237595	0.361737	0.139934	0.141324	0.999857	0.997966

Abbreviations: RMSE, root mean squared error. RMSE measures the deviation between the data and the model estimate. Trend refers to the percent of predictions that correctly predict the direction of the time trend from adjacent points. Coverage refers to the percent of data that are included within the ensemble uncertainty interval. For performance testing, data are split into training and out-of-sample testing sets. Columns with “in” refer to the performance within the training sample data, while columns with “out” refer to the performance of the ensemble within the withheld out-of-sample data. The model space is in brackets: “Global” models include all locations, “Data Rich” models include a subset of locations with high-quality data, and “Sub-Saharan Africa” models only include locations in Sub-Saharan Africa.

Liver cancer aetiology split models

The Level 3 cause “Liver cancer” is estimated as above, while additional methods are used to generate estimates for the underlying Level 4 causes related to liver cancer by type or aetiology. As in GBD 2021, all mortality estimates of the Level 3 Liver cancer model for those under age 10 were assigned to the Level 4 cause “Hepatoblastoma”. No aetiology was assigned to these under age 10 hepatoblastoma estimates. While hepatoblastoma is not the only type of liver cancer that children under 10 can die from, the GBD study currently only estimates Level 4 mortality from liver cancer under 10 years in the cause hepatoblastoma. Independent modelling using observed hepatoblastoma data and consideration of other liver cancer types under 10 years of age are anticipated in the future, depending on data availability.

For the rest of the age groups (age $\geq$ 10 years), the Level 3 total liver cancer deaths are proportionally split into the Level 4 causes of liver cancer representing the five aetiology groups included in the GBD: 1) Liver cancer due to hepatitis B, 2) Liver cancer due to hepatitis C, 3) Liver cancer due to alcohol use, 4) Liver cancer due to nonalcoholic steatohepatitis (NASH), and 5) Liver cancer due to other causes. To find data to inform these proportions, a systematic literature search was performed in PubMed on 10/24/2016 using the following search string:
("liver neoplasms"[All Fields] OR "HCC"[All Fields] OR "liver cancer"[All Fields] OR "Carcinoma, Hepatocellular"[Mesh]) AND (("hepatitis B"[All Fields] OR "Hepatitis B"[Mesh] OR "Hepatitis B virus"[Mesh] OR "Hepatitis B Antibodies"[Mesh] OR "Hepatitis B Antigens"[Mesh]) OR ("hepatitis C"[All Fields] OR "Hepatitis C"[Mesh] OR "hepatitis C antibodies"[MESH] OR "Hepatitis C Antigens"[Mesh] OR "Hepacivirus"[Mesh]) OR ("alcohol"[All Fields] OR "Alcohol Drinking"[Mesh] OR "Alcohol-Related Disorders"[Mesh] OR "Alcoholism"[Mesh] OR "Alcohol-Induced Disorders"[Mesh])) NOT (animals[MeSH] NOT humans[MeSH])”.

Studies were included if they provided proportion data that were representative for the location covered. Several studies not initially found through this search were added because they were included in the meta-analysis by de

Martel and colleagues.[32] We also included the study by Hong and colleagues after the authors provided us with additional data on the overlap in aetiologies.[33]

Liver cancer aetiology	Number of papers with proportion data
Liver cancer due to alcohol use	96
Liver cancer due to hepatitis B	267
Liver cancer due to hepatitis C	266
Liver cancer due to NASH	96
Liver cancer due to other causes	55

For each study, the proportions of liver cancer due to each of the five specific aetiologies were calculated. Cases were considered to be due to NASH when the manuscript explicitly listed the aetiology to be NASH or non-alcoholic fatty liver disease (NAFLD). Cases where the aetiology was listed as "cryptogenic", "idiopathic", or "unknown" were assessed as either implicitly NASH (included within the "NASH" category when alcohol, hepatitis B, and hepatitis C were reported), or of ambiguous aetiology (included within the "other causes" category when any of alcohol, hepatitis B, or hepatitis C could not be ruled out). These implicit NASH proportions were adjusted to the explicit NASH proportions using the GBD Meta-Regression– Bayesian, Regularised, Trimmed (MR-BRT) method as specified in Section X of the GBD 2023 paper "Non-fatal burden of 375 diseases and injuries, risk-attributable burden of 88 risk factors, and healthy life expectancy in 204 countries and territories, including 660 subnational locations, 1990–2023: a systematic analysis for the Global Burden of Disease Study 2023. Lancet (in review)" *(To the editors and reviewers: note that section detail will be finalized once the GBD 2023 Causes of Death Collaborators publication is final)*.[3] In manuscripts where the aetiology for a case was not known but major categories could not be ruled out (for example, if the study tested for hepatitis B and C, but did not assess alcohol use), only the explicitly defined proportions were included (in this example, including proportions for hepatitis B and C, and excluding any remainder). Any remaining named aetiologies were included under a combined "other" group (for example, hemochromatosis, autoimmune hepatitis, Wilson's disease, etc.). If multiple aetiologies were reported for any cases, these were assigned proportionally to the individual aetiologies reported in the study. Sex-combined sources were split into sex-specific proportions using the GBD MR-BRT method. Details of these models and the resulting MR-BRT crosswalk adjustment factors that were used to estimate sex-specific proportions from both-sex liver cancer aetiology proportion input data, and to adjust for implicit versus explicit reporting of NASH in these data, are provided in the table below.

Model	Crosswalk type	Reference category	Alternative category	Gamma	Beta Coefficient	Adjustment factor
Liver cancer due to alcohol use	Sex split	Male proportion	Both-sex proportion	0.00	-0.998	0.368
Liver cancer due to hepatitis B	Sex split	Male proportion	Both-sex proportion	0.07	-0.421	0.656
Liver cancer due to hepatitis C	Sex split	Male proportion	Both-sex proportion	0.42	0.299	1.349
Liver cancer due to other causes	Sex split	Male proportion	Both-sex proportion	0.37	0.260	1.297
Liver cancer due to NASH	Sex split	Male proportion	Both-sex proportion	0.00	0.090	1.095
Liver cancer due to NASH	NASH definition	Explicit NASH	Implicit NASH	0.91	-0.322	0.725

The proportion data from the liver cancer systematic literature review above were used as input for five separate DisMod-MR 2.1[3] models to determine the proportion of liver cancers due to the five aetiology subgroups for all locations, sexes, years, and all GBD age groups (#12 in Appendix 1 Figure 1). For liver cancer due to alcohol use, a prior value of 0% was set for ages 0–5 years. For liver cancer due to hepatitis B and hepatitis C, a prior value of 0% was set between age 0 and 0.01 years. The covariates used differed by model and direction. The liver cancer due to alcohol use model included positive covariates (with the beta prior minimum set at zero) for the litres of alcohol consumed *per capita* and the age-standardised proportion of alcohol drinkers. The liver cancer due to hepatitis B model included a positive covariate for age-standardised vaccine-adjusted hepatitis B seroprevalence (HBsAg) and a negative covariate for 10-year lagged hepatitis B 3-dose vaccine coverage. The liver cancer due to hepatitis C

model included a positive covariate for age-standardised chronic hepatitis C. The liver cancer due to NASH model included positive covariates for mean body mass index (BMI), the age-standardised prevalence of obesity, and the prevalence of NASH and non-alcoholic fatty liver disease.

Since the five aetiology proportion models were run independently of each other, the final proportion estimates were scaled to sum to 100% within each age, sex, year, and location, by dividing each proportion by the sum of the five (#13 in Appendix 1 Figure 1). For the liver cancer aetiology mortality estimates, we multiplied the parent cause "liver cancer" deaths (excluding deaths in ages less than 10, which are assigned to hepatoblastoma) by the corresponding scaled proportions (#14 in Appendix 1 Figure 1). Single cause estimates were later adjusted to fit into the separately modelled all-cause mortality in the CoDCorrect process. CoDCorrect also combines these five subtypes with the hepatoblastoma estimates (see below) to create a new total liver cancer estimate across all ages.

CoDCorrect

The CODEm estimates described above independently model the individual cause-level mortality without taking into account the separately modelled all-cause mortality. To ensure that all single causes add up to the all-cause mortality and that all child causes add up to the parent cause, an algorithm called "CoDCorrect" is used. Further details on the CoDCorrect algorithm can be found in Section X of the appendix to the GBD 2023 paper "Global burden of 292 causes of death in 204 countries and territories and 660 subnational locations, 1990–2023: a systematic analysis for the Global Burden of Disease Study 2023. Lancet (in review)" *(To the editors and reviewers: note that section detail will be finalized once the GBD 2023 Causes of Death Collaborators publication is final)*.[2] Final mortality estimates from a CoDCorrect run at the 250-draw level provide an estimated mean mortality with 95% uncertainty interval.

Calculating years of life lost

To calculate YLLs, the final death estimates after CoDCorrect adjustment are multiplied by the standard GBD life expectancy given the age at death. Further details on GBD life expectancy values can be found in the GBD 2023 paper, "Global age-sex-specific all-cause mortality and life expectancy estimates for 204 countries and territories and 660 subnational locations, 1950–2023: a comprehensive demographic analysis for the Global Burden of Disease Study 2023. Lancet (in drafting)".[6] Uncertainty is propagated from the CoDCorrect mortality estimates, calculating YLLs for each of the 250 CoDCorrect draws to provide estimated mean YLLs with corresponding 95% uncertainty intervals.

Incidence estimation

For all cancers except retinoblastoma, the final GBD cancer mortality estimates (after CoDCorrect adjustment) were transformed to incidence estimates by using the MIRs specific to that cancer cause (#1 in Appendix 1 Figure 2). Final mortality estimates at the individual draw-specific level were divided by the modelled MIR estimates (also at the individual draw-specific level) to generate a distribution of draws of incidence estimates (which provides an estimated mean incidence with 95% uncertainty interval). It was assumed that uncertainty in the MIRs was independent of uncertainty in the estimated mortality.

For retinoblastoma, the incidence estimation approach above was used for all locations except those in the high-income super-region. For high-income countries, death from retinoblastoma is extremely rare, which can lead to estimated MIRs close to zero and underestimation of incidence with the above approach (due to a numerator close to zero). To address this, alternative MIRs specific to locations in the high-income super-region were estimated by matching CODEm mortality estimates with cancer registry incidence data for these locations. Incidence draws for these locations were then estimated as detailed above, dividing the CoDCorrect estimates by these alternative high-income-specific MIRs (rather than the globally-informed MIRs). To avoid potential subsequent overestimation of incidence in these locations, the incidence rates were Winsorised to the 2.5th and 97.5th percentiles of incidence rates across countries in the high-income super-region.

For neuroblastoma and other eye cancer, the MIR models produced a small subset of draws with values that were implausibly low in the high-income super-region, which initially led to an overestimation of incidence in this step. To account for these influential outliers, we Winsorized the high-income MIR draws to a floor based on the 5th percentile of all the high-income MIRs for neuroblastoma, and on the 5th percentile of all high-income MIRs excluding United States of America subnationals (since this large group of locations were all very low) for other eye cancer.

Prevalence estimation

After transforming the final GBD cancer mortality estimates to incidence estimates (#1 in Appendix 1 Figure 2), incidence was combined with annual relative survival estimates from 1 to 10 years after diagnosis (#7 in Appendix 1 Figure 2). As in previous cycles, we directly used MIRs to estimate age-specific yearly cancer relative survival.[1,34]

Previous reports suggest that the value of (1 – MIR) may serve as a proxy for 5-year relative survival, with the exact correlation varying slightly by cancer type.[35] Because this correlation varies, we trained cancer-specific prediction models to estimate 5-year survival from MIRs, using data from SEER.[13] We used Surveillance Research Program, National Cancer Institute SEER*Stat software[36] to obtain mortality,[37] incidence,[38] and relative survival statistics from the nine SEER registries reporting from 1980–2014 (through 2014 so that all years had at least 5 years of follow-up time; #2 in Appendix 1 Figure 2), by cancer type, sex, 5-year time periods (e.g., 1980−84, 1985–89, etc.), and 5-year age groups (except combining 80+). For each cancer, we modelled SEER 5-year relative survival with MIRs calculated from SEER mortality and incidence, using a generalised linear model with a quasibinomial family and logit link, weighted by the number of index cases (#3 in Appendix 1 Figure 2).

To reduce variability due to small samples, we only included MIRs based on at least 25 incident cases (except for the rarer cancers mesothelioma, acute myeloid leukaemia, and acute lymphoid leukaemia, where MIRs based on at least 10 cases were included). These models were then applied to the GBD MIR estimates to predict an estimated 5-year survival for each age, sex, year, and location combination (#4 in Appendix 1 Figure 2). To prevent unrealistic values, predicted 5-year survival values were Winsorised to be between 0% and 100% survival.

To generate yearly survival estimates up to 10 years, we downloaded SEER sex- and age-specific annual 1-year through 10-year relative survival data from individuals diagnosed between 2001 and 2010 (2001 through 2010 so that all cases had at least 5 years of follow-up, with half having the full 10 years of follow-up).[38] A proportional scalar was calculated as the predicted GBD 5-year survival estimate divided by the SEER 5-year survival statistic, and was then used to generate yearly survival estimates by scaling the $1-10$ year SEER curve to the GBD survival predictions under the proportional hazards assumption (#5 in Appendix 1 Figure 2). These data and steps were the same as in GBD 2021.

To facilitate the estimation of the total person-time survived, the estimated relative survival described above (the survival of cancer cases relative to those without cancer, given the expected background mortality) was next transformed into absolute survival estimates (the overall survival of cancer cases, including all causes of mortality; #6 and #7 in Appendix 1 Figure 2). To account for background mortality in the relative survival estimates, GBD 2023 lifetables were used to calculate lambda (λ) values:

$$\lambda = \frac{\ln\left(\frac{nLx_n}{nLx_{n+1}}\right)}{5}$$

nLx = person years lived between ages x and x+n (from GBD lifetables).

GBD 2023 lifetables are described in the GBD 2023 paper, "Global age-sex-specific all-cause mortality and life expectancy estimates for 204 countries and territories and 660 subnational locations, 1950–2023: a comprehensive demographic analysis for the Global Burden of Disease Study 2023. Lancet (in drafting)".[6] Absolute survival was then calculated using an exponential survival function:

$$absolute\ survival = relative\ survival * e^{\lambda * t}$$

t = time (in years)

Absolute survival was combined with incidence to estimate the prevalence at each year 1 through 10 after diagnosis.

Disability estimation

To estimate disability for each cancer, total prevalence is split into four sequelae: (1) diagnosis and primary therapy phase; (2) controlled phase; (3) metastatic phase; and (4) terminal phase (#8 in Appendix 1 Figure 2). The diagnosis and primary therapy phase represents the time from the onset of symptoms to the end of initial treatment. The controlled phase represents the time between finishing primary treatment and the earliest of either cure (defined as recurrence- and progression-free survival after 10 years), death from another cause, or progression to the metastatic phase. The metastatic phase represents the time period of intensive treatment for metastatic disease, as determined for each cancer by evaluating data from SEER averages (Appendix 1 Table 10).[13] The terminal phase represents the one-month period prior to death. Each of these four sequelae has a separate disability weight, which are each the same across cancer types (Appendix 1 Table 11). Because of the long-term disability associated with certain treatment-related procedures, additional disability beyond these four sequelae is estimated for five cancers: breast cancer (disability due to mastectomy), larynx cancer (disability due to laryngectomy), colon and rectum cancer (disability due to stoma), bladder cancer (disability due to incontinence from cystectomy), and prostate cancer (disability due to either incontinence or impotence from prostatectomy).

For the purposes of calculating disability due to cancer, survivors beyond 10 years were considered cured. For this group, the survivor population prevalence person-time was divided into two sequelae: (1) diagnosis and primary therapy phase; and (2) controlled phase (or remission). For the population that did not survive beyond 10 years, the yearly prevalence person-time was sequentially divided into four sequelae by assigning fixed durations for each of the (4) terminal phase, (3) metastatic phase, and (1) diagnosis and primary therapy phase, and assigning any remaining prevalence person-time to the (2) controlled (or remission) phase (#8 in Appendix 1 Figure 2). The duration of these four sequelae remained the same as for GBD 2013,[39] GBD 2015,[40] GBD 2016,[41] GBD 2017,[34] GBD 2019,[1] and GBD 2021,[42] except for updates to other malignant neoplasms (which was recalculated with the addition of new cancer causes) and other non-Hodgkin lymphoma (which was set equal to non-Hodgkin lymphoma for GBD 2023, to ensure alignment of parent and child estimates). The duration of each phase, along with the sources used to determine their length,[43–48] are listed in Appendix 1 Table 10. For the diagnosis and primary therapy phase, the duration was taken from primary literature or expert opinion. For the disseminated/metastatic phase, the duration was taken from primary literature, or as the median survival time reported by SEER for the individuals described in the note column. The terminal phase duration was set at 1 month for all cancers, while the controlled (or remission) phase was calculated as any remaining time beyond the other phases.

Appendix Table 10: Duration of four prevalence phases by cancer

GBD cause	Diagnosis and primary therapy phase (months)*	Controlled phase, or remission	Disseminated/ metastatic phase (months)*	Note for disseminated/ metastatic phase	Terminal phase (months)
Lip and oral cavity cancer	5.3[43]	The remission phase duration is calculated based on the remaining time after attributing other sequelae durations.	9.3[44]	SEER Stage IVc	1.0
Nasopharynx cancer	5.3[43]		13.2[44]	SEER Stage IVc	1.0
Other pharynx cancer	5.3[43]		7.9[44]	SEER Stage IVc	1.0
Oesophageal cancer	5.0[43]		4.6[44]	SEER Summary Stage 1977 (Distant site/node involved) 1995–2000	1.0
Stomach cancer	5.2[43]		3.9[44]	SEER Summary Stage 1977 (distant site/node involved) 1995–2000	1.0
Colon and rectum cancer	4.0[45]		9.7[44]	SEER Summary Stage 1977 (distant site/node involved) 1995–2000	1.0
Liver cancer	4.0		2.5[44]	SEER Summary Stage 1977 (distant site/node involved) 1995–2000	1.0
Hepatoblastoma	6.0		23.1[44]	SEER Summary Stage 1977 (distant site/node involved) 1995–2000	1.0
Gallbladder and biliary tract cancer	4.0		3.5[44]	SEER Summary Stage 1977 (distant site/node involved) 1995–2000	1.0

Pancreatic cancer	4.1[43]		2.5[44]	SEER Summary Stage 1977 (distant site/node involved) 1995–2000	1.0
Larynx cancer	5.3[43]		8.8[44]	SEER Stage IVc	1.0
Tracheal, bronchus, and lung cancer	3.3[45]		4.5[44]	SEER Summary Stage 1977 (distant site/node involved) 1995–2000	1.0
Malignant skin melanoma	2.9[46]		7.2[44]	SEER Summary Stage 1977 (distant site/node involved) 1995–2000	1.0
Soft tissue and other extraosseous sarcomas	10.0		10.7[44]	SEER Summary Stage 1977 (distant site/node involved) 1995–2000	1.0
Malignant neoplasm of bone and articular cartilage	10.0		19.8[44]	SEER Summary Stage 1977 (distant site/node involved) 1995–2000	1.0
Breast cancer	3.0[45]		17.7[44]	SEER Summary Stage 1977 (distant site/node involved) 1995–2000	1.0
Cervical cancer	4.8[43]		9.2[44]	SEER Summary Stage 1977 (distant site/node involved) 1995–2000	1.0
Uterine cancer	4.6[43]		11.6[44]	SEER Summary Stage 1977 (distant site/node involved) 1995–2000	1.0
Ovarian cancer	3.2[45]		25.6[44]	SEER Summary Stage 1977 (distant site/node involved) 1995–2000	1.0
Prostate cancer	4.0[45]		30.4[44]	SEER Summary Stage 1977 (distant site/node involved) 1995–2000	1.0
Testicular cancer	3.7[43]		19.5[44]	SEER Stage III	1.0
Kidney cancer	5.3[43]		5.4[44]	SEER Summary Stage 1977 (distant site/node involved) 1995–2000	1.0
Bladder cancer	5.1[43]		5.8[44]	SEER Summary Stage 1977 (distant site/node involved) 1995–2000	1.0
Brain and central nervous system cancer	5.0		6.9[44]	SEER median age-standardised survival all patients, all years	1.0
Eye cancer	2.9		16.0[44]	SEER median age-standardised survival all patients, all years	1.0
Retinoblastoma	6.0		6.4[49]	Literature	1.0
Other eye cancers	2.9		16.0[44]	SEER median age-standardised survival all patients, all years	1.0
Neuroblastoma and other peripheral nervous cell tumours	10.0		47.4[44]	SEER Summary Stage 1977 (distant site/node involved) 1995–2000	1.0
Thyroid cancer	3.0		19.4[44]	SEER Stage IVc	1.0
Mesothelioma	4.0		7.8[44]	SEER Summary Stage 1977 (distant site/node involved) 1995–2000	1.0
Hodgkin lymphoma	3.7[45]		26.0[47]	Literature	1.0
Non-Hodgkin lymphoma	3.7[45]		7.7[47]	Literature	1.0
Burkitt lymphoma	6.0		8.8[44]	SEER Summary Stage 1977 (distant site/node involved) 1995–2000	1.0
Other non-Hodgkin lymphoma	3.7[45]		7.7[47]	Literature	1.0
Multiple myeloma	7.0[43]		36.8[44]	SEER median age-standardised survival all patients, all years	1.0
Leukaemia	5.0[43]		43.7[44]	SEER median age-standardised survival all patients, all years	1.0
Acute lymphoid leukaemia	12.0		7.0[44]	SEER median age-standardised survival all patients, all years	1.0
Chronic lymphoid leukaemia	6.0		48[48]	SEER median age-standardised survival all patients, all years	1.0
Acute myeloid leukaemia	6.0		4.6[44]	SEER median age-standardised survival all patients, all years	1.0
Chronic myeloid leukaemia	6.0		4.6[44]	SEER median age-standardised survival for AML (patients with CML die in blast crisis, which is treated like AML) all patients, all years	1.0
Other leukaemia	6.0		48.0[48]	SEER median age-standardised survival all patients, all years	1.0
Other malignant neoplasms	4.9**		15.8[44]	SEER median age-standardised survival all patients, all years	1.0

Abbreviations: GBD, the Global Burden of Diseases, Injuries, and Risk Factors 2023 Study; SEER, Surveillance, Epidemiology and End Results Program.*Superscripts refer to references used to inform these values. Durations without superscript are based on expert opinion. Cancers are ordered in accordance with the GBD hierarchy. Non-melanoma skin cancers and other neoplasms are not included in this table as the duration assigned for these causes is estimated using DisMod models each GBD round. **The duration for "Other malignant neoplasms" was calculated as the mean of all Level 3 causes

(Level 4 causes were excluded from this calculation).

For cancer-specific procedure sequelae, hospital data were used to estimate the number of cancer patients undergoing mastectomy, laryngectomy, stoma, prostatectomy, and cystectomy (#9 in Appendix 1 Figure 2). Input data for these proportions came from clinical informatics sources of hospital data from the United States of America,[50,51] Canada,[52] and Mexico,[53] and remained the same as in GBD 2013,[39] GBD 2015,[40] GBD 2016,[41] GBD 2017,[34] GBD 2019,[1] and GBD 2021.[42] Proportions for each procedure were generated by dividing the rate of the procedure (generated from the diagnostic codes in the hospital dataset) by the GBD age-specific and sex-specific disease incidence rates for that country. Diagnostic codes used for each procedure are listed in the table below.

**Procedure**	**Cancer**	**Procedure code (ICD-9-CM[54])**
Mastectomy	Breast cancer	854, 8541, 8542, 8543, 8544, 8545, 8546, 8547, 8548
Laryngectomy	Larynx cancer	301, 303, 304, 3029
Stoma	Colon and rectum cancer	461, 4610, 4611, 4613, 4862
Cystectomy	Bladder cancer	5771, 5779
Prostatectomy	Prostate cancer	603, 604, 605, 606, 6062

To estimate procedure-related disability for each of these five cancers, the procedure proportions (proportion of each cancer population that undergo these procedures) were used as input for a proportion model in DisMod-MR 2.1[3] to estimate the proportions for all combinations of location, age, year, and sex. Details of DisMod-MR 2.1 and clinical and claims data processing can be found in Section X of the appendix to the GBD 2023 paper "Non-fatal burden of 375 diseases and injuries, risk-attributable burden of 88 risk factors, and healthy life expectancy in 204 countries and territories, including 660 subnational locations, 1990–2023: a systematic analysis for the Global Burden of Disease Study 2023. Lancet (in review)" *(To the editors and reviewers: note that section detail will be finalized once the GBD 2023 Causes of Death Collaborators publication is final)*.[3]

Since colostomy or ileostomy procedures are also performed for reasons other than cancer, a literature review was conducted for GBD 2013 to determine the proportion of ostomies due to colon and rectum cancer. Based on the results of the literature review that an average of 58% of ostomies are done for colon and rectum cancer, the "all cause" colostomy proportions were multiplied by 0.58.[55–57]

The final procedure proportions were applied to the incident cases of the respective cancers and multiplied with the proportion of the incident population surviving for 10 years to determine the incident cases of the cancer population that underwent procedures and that survived beyond 10 years. These estimates of survivors at 10 years were then used as an input for DisMod-MR 2.1, with a remission specification of zero and an excess mortality rate prior of 0 to 0.1, as well as with increasing both the age of the population and the year by 10 years to reflect prevalence after that population has survived 10 years. The results from this model are incidence and lifetime prevalent cases of individuals with these cancer-related sequelae who have survived beyond 10 years.

Since disability associated with prostatectomy comes from impotence and incontinence, and not from the prostatectomy itself, 18% of the prostatectomy prevalence was assumed to have incontinence and 55% was assumed to have impotence, based on a literature review done for GBD 2013.[58–65] Cases were assigned disability for either impotence or incontinence, but no cases were assigned disability from both. We assumed that for the population surviving up to 10 years, only those in the remission phase experience additional disability due to procedures (e.g., women suffering from metastatic breast cancer do not experience additional disability due to a mastectomy during this phase). To estimate the prevalence of the cancer population in remission during the first 10 years after diagnosis with and without procedure-related disability, we multiplied the prevalence of the population in the remission phase with the proportion of the population undergoing a procedure. This step allowed us to estimate disability during the remission phase for both the population experiencing disability due to the remission phase alone, as well as the population experiencing disability from the remission phase and the additional procedure-related disability combined.

Years lived with disability estimation

Lastly, the procedure sequelae prevalence and general sequelae prevalence were multiplied with their respective disability weights (Appendix 1 Table 11, #10 in Appendix 1 Figure 2) to obtain the number of YLDs for each sequelae (#11 and #12 in Appendix 1 Figure 2). The methods used to generate disability weights are described in Section X, "Disability weights" in the appendix to the paper "Non-fatal burden of 375 diseases and injuries, risk-attributable burden of 88 risk factors, and healthy life expectancy in 204 countries and territories, including 660 subnational locations, 1990–2023: a systematic analysis for the Global Burden of Disease Study 2023. Lancet (in review)" *(To the editors and reviewers: note that section detail will be finalized once the GBD 2023 Causes of Death Collaborators publication is final)*.[3] In brief, disability weights are created from survey data to represent the magnitude of health loss associated with an outcome. These disability weights range from 0, implying a state equivalent to full health, to 1, a state equivalent to death. Summing these sequelae-specific YLDs then provides the total YLD estimate associated with each cancer cause.

Appendix Table 11: Disability weights

Health state	Relevant cancers	Lay description	Disability weight (95% Uncertainty Interval)
Cancer, diagnosis and primary therapy phase	All cancers except non-melanoma skin cancer	This person has pain, nausea, fatigue, weight loss and high anxiety.	0.288 (0.193 to 0.399)
Cancer, controlled phase	All cancers except non-melanoma skin cancer	This person has a chronic disease that requires medication every day and causes some worry but minimal interference with daily activities.	0.049 (0.031 to 0.072)
Cancer, metastatic phase	All cancers except non-melanoma skin cancer	This person has severe pain, extreme fatigue, weight loss and high anxiety.	0.451 (0.307 to 0.600)
Cancer, terminal phase with medication	All cancers except non-melanoma skin cancer	This person has lost a lot of weight and regularly uses strong medication to avoid constant pain. The person has no appetite, feels nauseous, and needs to spend most of the day in bed.	0.540 (0.377 to 0.687)
Mastectomy	Breast cancer	This person had one of her breast removed and sometimes has pain or swelling in the arms.	0.036 (0.020 to 0.057)
Stoma	Colon and rectum cancer	This person has a pouch attached to an opening in the belly to collect and empty stools.	0.095 (0.063 to 0.131)
Laryngectomy	Larynx cancer	This person has difficulty speaking, and others find it difficult to understand.	0.051 (0.032 to 0.078)
Urinary incontinence	Bladder cancer, Prostate cancer	This person cannot control urinating.	0.139 (0.094 to 0.198)
Impotence	Prostate cancer	This person has difficulty in obtaining or maintaining an erection.	0.017 (0.009 to 0.030)
Disfigurement, level 1	Non-melanoma skin cancer (basal cell carcinoma, cutaneous squamous cell carcinoma), mild	This person has a slight, visible physical deformity that others notice, which cause some worry and discomfort.	0.011 (0.005 to 0.021)
Disfigurement, level 2	Non-melanoma skin cancer (cutaneous squamous cell carcinoma), moderate	This person has a visible physical deformity that causes others to stare and comment. As a result, the person is worried and has trouble sleeping and concentrating.	0.067 (0.044 to 0.096)
Disfigurement, level 3, with itch or pain	Non-melanoma skin cancer (cutaneous squamous cell carcinoma), severe	This person has an obvious physical deformity that is very painful and itchy. The physical deformity makes others uncomfortable, which causes the person to avoid social contact, feel worried, sleep poorly, and think about suicide.	0.576 (0.401 to 0.731)

For persons experiencing combined disability from a surgical procedure-related disability during the remission phase, we calculated a combined disability weight for each procedure using a multiplicative function to combine the general remission phase disability weight with the procedure-related disability weight. Generally, this calculation was: 1 - (1 - 'remission phase weight') * (1 - 'procedure weight'). For example, the combined disability weight for "remission phase, with mastectomy" was calculated as: 1 - (1 – 0.049) * (1 – 0.036) = 0.083.

Reporting standards

Countries and subnational locations included in GBD 2023 can be found in the appendix to the manuscript "Global burden of 292 causes of death in 204 countries and territories and 660 subnational locations, 1990–2023: a systematic analysis for the Global Burden of Disease Study 2023. Lancet (in review)".[2] All rates are reported per 100 000 person-years. Annualised rates of change (ARC) represent the average percentage change per year over a defined time period and are calculated as:

$$ARC = \frac{\ln\left(\frac{X\_y2}{X\_y1}\right)}{y2 - y1}$$

X_yn = value of measure (e.g., incidence) at year $\_y_n$
$y1$ = starting year (e.g., 1990)
$y2$ = ending year (e.g., 2023)

The overall percent change in a measure between two years was calculated as: $[(\text{measure}_{y2} - \text{measure}_{y1}) / \text{measure}_{y1}]*100$.

The GBD world population age standard was used to calculate age-standardised rates presented in GBD 2023. The world population age standard was calculated using the non-weighted mean of the GBD cycle's age-specific population proportional distributions for all national locations with populations greater than 5 million people. The final values used for the age standard are specified in the appendix to the GBD 2023 paper, "Global age-sex-specific all-cause mortality and life expectancy estimates for 204 countries and territories and 660 subnational locations, 1950–2023: a comprehensive demographic analysis for the Global Burden of Disease Study 2023. Lancet (in drafting)".[6]

Socio-demographic Index (SDI)

Methods for the calculation of SDI are described in the appendix to the GBD 2023 paper, "Global age-sex-specific all-cause mortality and life expectancy estimates for 204 countries and territories and 660 subnational locations, 1950–2023: a comprehensive demographic analysis for the Global Burden of Disease Study 2023. Lancet (in drafting)".[6] In brief, SDI is a summary indicator to represent background levels of social and economic conditions that can influence health outcomes in a given location. This summary indicator is comprised of three indices: lag-distributed income *per capita*, mean education for those aged 15 years or older, and total fertility rate for those younger than 25 years of age. Possible values range from 0 to 1 for each of these three indices, representing the bounds with which lower or higher values of the level of development for that index would no longer worsen or improve health outcomes, respectively. The composite SDI is the geometric mean of these three indices for a given location and year combination.

Forecasting

The methods used for forecasting GBD results through 2050 are described in the GBD 2021 paper "GBD 2021 Forecasting Collaborators. Burden of disease scenarios for 204 countries and territories, 2022–2050: a forecasting analysis for the Global Burden of Disease Study 2021."[5] Because these forecast results are based on the GBD 2021 estimates, we updated the forecasts using a scalar based on GBD 2023 cancer estimates. This scalar was calculated for the mean estimate (by age, sex, and location) as the ratio of the GBD 2023 estimate to the GBD 2021 forecast result for the year 2023. Each of the GBD 2021 forecasted years 2024 through 2050 were then multiplied by this scalar to get updated mean forecast results. For the upper and lower UI, the upper and lower scalar values were calculated as the ratio of the differences between the mean and the UI boundaries (upper or lower) for the GBD 2023 estimate and the GBD 2021 forecasted values for 2023. The distance between the forecasted mean and the UI boundaries for each year 2024-2050 were then multiplied by these scalars, and the resulting distance was added or subtracted from the updated mean forecast to obtain the updated forecast UIs.

Probability of death due to cancer

The United Nations Sustainable Development Goal 3.4 has a goal target of one-third reduction in premature mortality in ages 30-70 from non-communicable diseases (including cancer) by 2030.[66] To estimate progress towards this goal we calculated the probability of death from cancer, which is a component of the non-communicable disease total, for the years 2015 and 2030. The probability of death from cancer in ages 30-70 years

was calculated as the sum of the age group-specific probabilities of death using the cancer mortality rates in the following equation:

$$P(death)_{30-70} = \sum_{i=g}^{G} \frac{n * m_i}{1 + n * (1 - a) * m_i}$$

G = the set of 5-year age groups from 30-70 (here, 30-34, 35-39, etc.)
g = a 5-year age group (e.g., 30-34)
n = the width of the age group (here, 5 for each age group, e.g., 30-34)
m_i = the age-group-specific cancer mortality rate for age group i
a = the time of death, assumed to be the midpoint (here, 0.5)

This approach assumes deaths occur on average at the midpoint of the year, assumes no other causes of death, and assumes equal probability of death for each year within an age group. The calendar year 2015 was calculated using GBD 2023 estimates for the year 2015. The calendar year 2030 was calculated using GBD 2021 forecast results scaled to GBD 2023 estimates for the year 2030 (described above). The percent change in the probability of death from 2015 to 2030 was calculated as: $[P(death)_{2030} – P(death)_{2015}] / P(death)_{2015}$.

Interpretation of results

Changes in GBD 2023 methods and results from GBD 2021

Cancer mortality estimates for GBD 2023 can differ from GBD 2021 results for multiple reasons. New and updated cancer mortality data were added from vital registration system data, verbal autopsy studies, and cancer registry incidence data. MIRs were informed by a substantial amount of new paediatric cancer registry data and modeling updates, and we introduced a new method of crosswalking CR data and dropping CR mortality input data that overlapped with VR data. For GBD 2023 there are large relative increases in the mortality estimates for 'Other neoplasms' and the underlying cause 'Other benign and in situ neoplasms' due to CodCorrect excluding deaths in the underlying cause 'Other benign and in situ neoplasms' in previous cycles, and codes for benign tumours of the brain and central nervous system were included in mortality estimates for these causes in GBD 2023, as these non-invasive tumors can still cause death through intracranial pressure.

Comparison to GLOBOCAN

The other major group producing global and country-level cancer burden estimates is the International Agency for Research on Cancer (IARC) with their GLOBOCAN estimates.[67] Differences in data sources and in methods used between the GBD study and GLOBOCAN can lead to differences in results. For GLOBOCAN, estimates are produced separately at the national level, using several different regression or imputation models differentially by country depending on the data available.[68,69] For the GBD, cancer estimation occurs globally across all locations (including national and subnational locations) and years (1990 through 2023 for the GBD 2023 study) following a consistent, well-documented ensemble modelling approach that includes relevant covariate data, which allows for cross-validation of models as well as determination of uncertainty. Another major difference is the ability in the GBD study to adjust single cause estimates to the all-cause mortality envelope, which is determined independently. This allows correction for the under-diagnosis of cancer in countries with inadequate diagnostic resources.

Redistribution of a fraction of undefined causes of death to certain cancers is another methodological advantage of the GBD study as compared with GLOBOCAN,[69] and estimates for cancer mortality can therefore differ substantially in countries with a large proportion of undefined causes of deaths in their vital registration data or a large proportion of undefined cancer cases in their cancer registry data. For instance, since GBD 2019, the GBD study has considered ICD-10 code C22.9 ("Malignant neoplasm of liver, not specified as primary or secondary") a garbage code (see section "6. Redistribution" for definition) and redistributes these deaths to liver cancer and 13 other cancer types (colon and rectum, pancreas, prostate, breast, oesophagus, bladder, kidney, stomach, ovary, uterus, cervix, gallbladder, and testes) to avoid misassigning these deaths, which may be from liver metastases rather than primary liver cancer.

There are also differences in the inclusion and categorisation of cancer types reported. For instance, squamous cell carcinomas are included in both GBD 2023 and GLOBOCAN 2022 mortality estimates, while for overall cancer incidence estimates basal cell carcinomas are included in GBD 2023 but excluded in GLOBOCAN 2022. Additionally, some cancers are individually reported in GBD 2023 which are not reported separately in GLOBOCAN 2022 (e.g., malignant neoplasm of bone and articular cartilage) or are reported in GLOBOCAN 2022 (e.g., penis cancer), which in GBD 2023 are included within the "Other malignant neoplasms" cause.[69]

Tables reporting GBD estimates globally and by various subgroups

Tables included in the main text and appendix report GBD estimates both globally and by various subgroups, such as by World Bank income groups. As not all GBD locations are included in the World Bank classifications, these estimates may not sum to the global estimate. In addition, tables reporting percentage changes over time may report global trends that differ from the subgroup trends due to Simpson's paradox.[70]

Limitations

There are certain limitations to consider when interpreting the GBD cancer burden estimates. First, even though every effort is made to include the most recently available data for each country, data-seeking resources are not limitless, and new data cannot always be accessed and processed as soon as they are made available. It is therefore possible that the GBD study does not include all available data sources for cancer incidence or cancer mortality. Second, different redistribution methods can potentially change the cancer estimates substantially if the data sources used for the estimated location contain a large proportion of undefined causes; however, neglecting to account for these undefined deaths would likely introduce an even greater bias in the disease estimates. Third, using mortality-to-incidence ratios to transform cancer registry incidence data to mortality estimates requires accurate estimation of MIRs. For GBD 2023 we made further refinements to the estimation of MIRs, but the method remains sensitive to under-diagnosis of cancer cases or under-ascertainment of cancer deaths. However, given that the majority of data used for the cancer mortality estimation come from vital registration data and not cancer registry data, this is not a major limitation. Finally, estimates are not reported for non-sovereign or low-population locations that are not modelled in the GBD 2023 framework, including Antarctica, French Guiana, Svalbard, and Western Sahara.[2] These locations are coloured grey in the global map figures presented.

Additional method summaries for non-melanoma skin cancer (squamous and basal cell carcinoma)

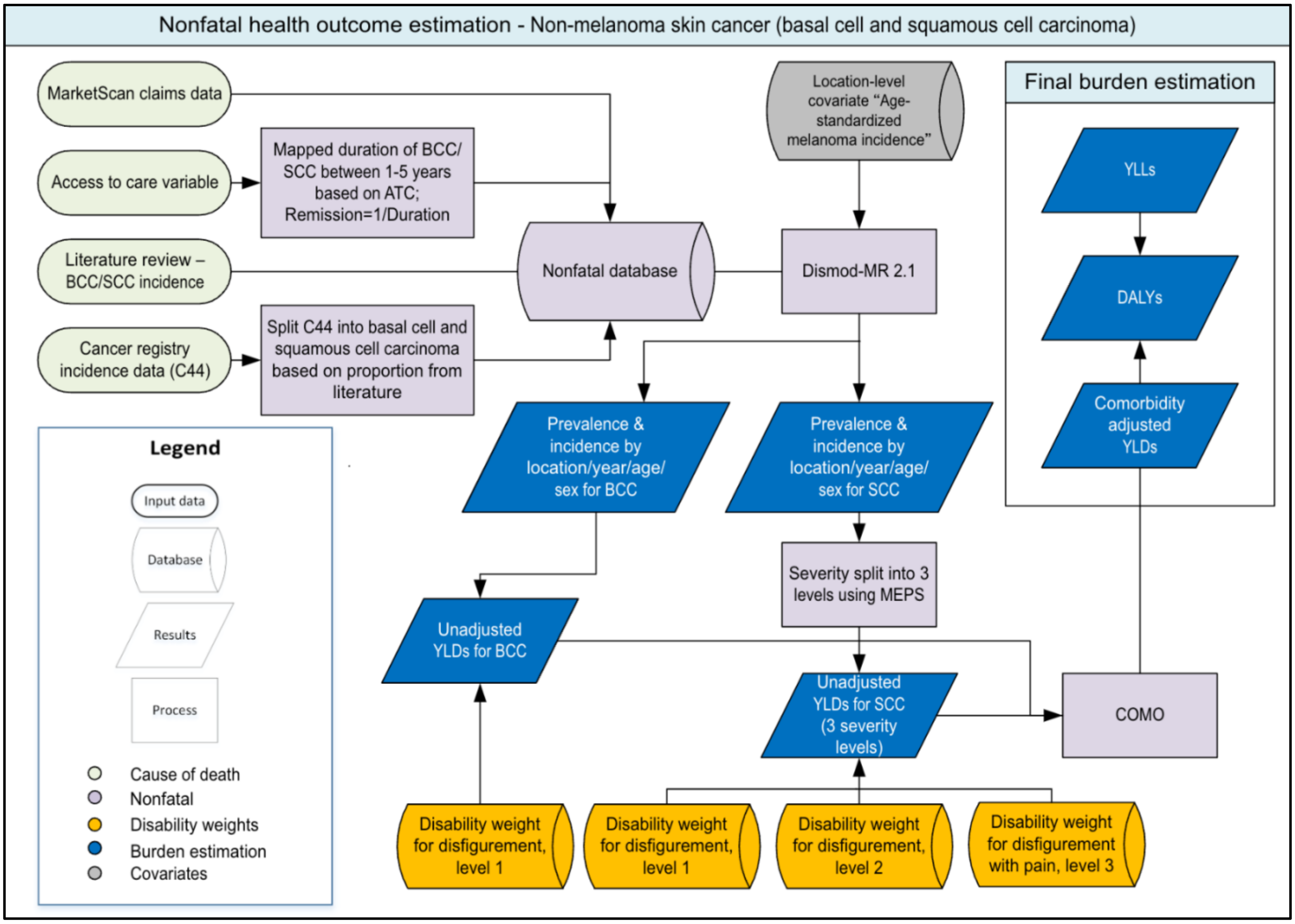

Appendix Figure 3: Flowchart of GBD non-melanoma skin cancer nonfatal estimation. Abbreviations: BCC, basal cell carcinoma; COMO, comorbidity correction microsimulation; DALYs, disability-adjusted life-years; DisMod-MR, disease model - Bayesian meta-regression; MEPS, Medical Expenditure Panel Survey; SCC, squamous cell carcinoma; YLDs, years lived with disability; YLLs, years of life lost.

Case definition

In the GBD framework, the Level 3 cause "Non-melanoma skin cancer" (NMSC) is comprised of two Level 4 causes, "Non-melanoma skin cancer (basal cell carcinoma)" and "Non-melanoma skin cancer (squamous cell carcinoma)". Mortality estimates for NMSC are only produced for squamous cell carcinoma in the GBD, under the assumption that basal cell carcinoma causes almost no deaths. Both Level 4 causes contribute to non-fatal burden estimates. NMSC in the GBD framework does not include other types of skin cancer (e.g., melanoma, Merkel cell carcinoma, etc.).

Input data

For mortality data, all deaths reported as "C44" (ICD-10) or "173" (ICD-9) were mapped to the "Non-melanoma skin cancer (squamous cell carcinoma)" GBD cause (Appendix 1 Table 12). Since squamous cell carcinomas are very infrequently recorded by cancer registries, only vital registration system data were used as input for the squamous cell carcinoma mortality modelling.

For incidence data, we used squamous cell and basal cell skin cancer incidence data from cancer registries, primary literature, and insurance claims. Only cancer registries that were listed in Cancer Incidence in Five Continents (CI5)[14–24] as registering squamous cell carcinoma or basal cell carcinoma, respectively, were included in the model input data. In total, there were 1,212 sources for basal cell carcinoma incidence data and 1,417 sources for squamous cell carcinoma incidence data. Clinical informatics sources included the MarketScan administrative claims database in the United States of America.[71] Additional details on claims data processing are available in Section X of the appendix to the GBD 2023 paper, "Non-fatal burden of 375 diseases and injuries, risk-attributable burden of 88 risk factors, and healthy life expectancy in 204 countries and territories, including 660 subnational locations, 1990–2023: a systematic analysis for the Global Burden of Disease Study 2023. Lancet (in review)" *(To the editors and reviewers: note that section detail will be finalized once the GBD 2023 Causes of Death Collaborators publication is final)*.[3]

Appendix Table 12: ICD codes mapped to non-melanoma skin cancers in clinical informatics data

GBD cause	ICD-10	ICD-9
Non-melanoma skin cancer (basal cell carcinoma)	C44.01, C44.11, C44.111, C44.112, C44.119, C44.21, C44.211, C44.212, C44.219, C44.31, C44.310, C44.311, C44.319, C44.41, C44.51, C44.510, C44.511, C44.519, C44.61, C44.611, C44.612, C44.619, C44.71, C44.711, C44.712, C44.719, C44.81, C44.91	173.01, 173.11, 173.21, 173.31, 173.41, 173.51, 173.60, 173.61, 173.71, 173.81, 173.91
Non-melanoma skin cancer (squamous cell carcinoma)	C44.02, C44.12, C44.121, C44.122, C44.129, C44.22, C44.221, C44.222, C44.229, C44.32, C44.320, C44.321, C44.329, C44.42, C44.52, C44.520, C44.521, C44.529, C44.62, C44.621, C44.622, C44.629, C44.72, C44.721, C44.722, C44.729, C44.82, C44.92	173.02, 173.12, 173.22, 173.32, 173.42, 173.52, 173.62, 173.72, 173.82, 173.92

Abbreviations: GBD, the Global Burden of Diseases, Injuries, and Risk Factors 2023 study; ICD-9, International Classification of Diseases, Ninth Revision; ICD-10, International Classification of Diseases, Tenth Revision.

Modelling strategy

NMSC deaths are modelled in CODEm using vital registration data, as detailed above. All NMSC deaths are modelled as squamous cell carcinoma deaths, as we assumed that there are no deaths from basal cell carcinoma.

For cancer registry incidence data reported at the three-digit level (i.e., C44: "Other and unspecified malignant neoplasm of skin"), fixed proportions reported in Karagas et al. were used to split C44 into squamous cell carcinoma and basal cell carcinoma.[72] For GBD 2023, we excluded data where the number of cases was less than 1. These data, along with data from clinical and literature sources, were input into cause-specific DisMod-MR 2.1[3] models to estimate incidence and prevalence for squamous cell carcinoma and basal cell carcinoma. Prevalence was calculated as a function of two extreme scenarios (duration of one versus five years). Country-, age-, sex-, and year-specific duration was estimated using a country-age-sex-year-specific relative access to care score.

The access to care score was based on the melanoma mortality-to-incidence ratio (MIR):

$$Access\ to\ care = 1 - \frac{Age\ standardized\ MIR_{cys} - Age\ standardized\ MIR_{min}}{Age\ standardized\ MIR_{max} - Age\ standardized\ MIR_{min}}$$

c = country; y = year; s = sex; Age-standardised MIR_{min} = lowest melanoma MIR for all countries and years; Age-standardised MIR_{max} = highest melanoma MIR for all countries and years.

Remission was calculated as the inverse of the duration estimates and used as additional input for DisMod-MR 2.1. Country-level covariates of age-standardized melanoma incidence and lag distributed income were included in the squamous cell carcinoma model while only age-standardized melanoma incidence was included in the basal cell carcinoma model.

To reflect differing degrees of disability due to NMSC, we used three levels of severity of disfigurement that were derived from the Medical Expenditure Panel Survey (MEPS).[73] For squamous cell carcinoma, disability severity was split into 80% mild, 15% moderate, and 5% severe disfigurement. For basal cell carcinoma, disability severity was split into 60% asymptomatic (without disability) and 40% with mild disfigurement. The prevalence person-

time was multiplied by the distinct disability weights associated with these sequelae (Appendix 1 Table 11) to generate YLDs.

Interpretation of results

Although the data availability for NMSC is a challenge, it is a common incident cancer and thus has been included in the GBD framework for both incidence and mortality since GBD 2016. Non-melanoma skin cancer incidence and mortality estimates are not widely available from other sources. GLOBOCAN, for example, reported cases and deaths due to NMSC for the first time in their 2018 release; these GLOBOCAN NMSC incident case estimates excluded basal cell carcinoma, while GLOBOCAN NMSC death estimates included all types of NMSC.[69,74]

Limitations

Cancer registry data for NMSC incidence should be interpreted with caution due to a substantial amount of underreporting, and because of common rules that only the first NMSC diagnosis is required to be registered. Many cancer registries therefore do not report NMSC at all. Information regarding whether or not cancer registries capture NMSC is not consistently available. Therefore, no cancer registry data were used to estimate deaths due to squamous cell carcinoma of the skin. For vital registration data, we assumed that there are no deaths due to basal cell carcinoma, and therefore all deaths attributed to basal cell carcinoma were included instead as squamous cell carcinoma deaths.

Additional method summary for other neoplasms

Method summary for myelodysplastic, myeloproliferative, and other haemopoietic neoplasms

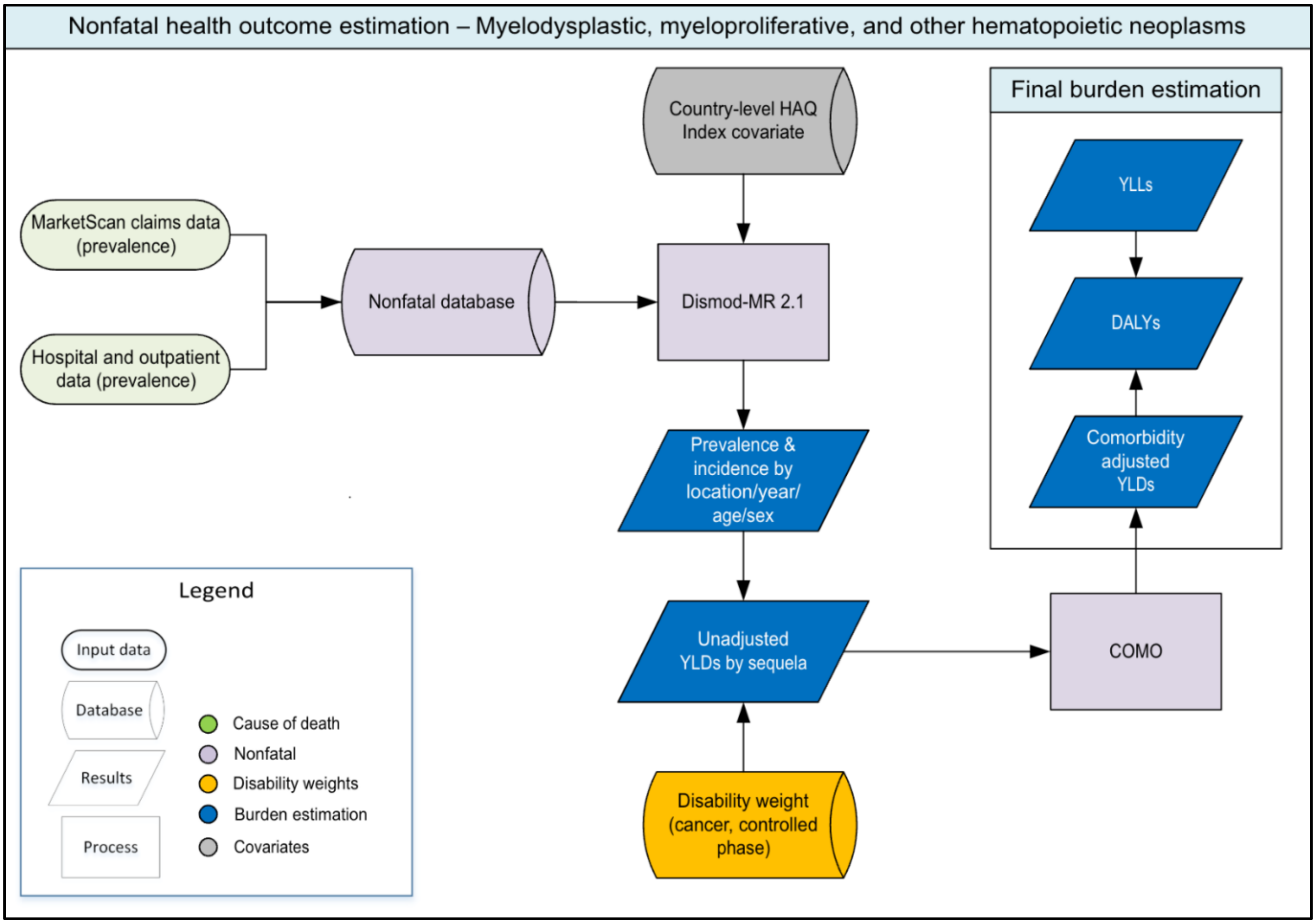

Appendix Figure 4: Flowchart of GBD myelodysplastic, myeloproliferative, and other haemopoietic neoplasms nonfatal estimation. Abbreviations: DisMod-MR, disease model - Bayesian meta-regression; COMO, comorbidity correction microsimulation; DALYs, disability-adjusted life-years; HAQ Index, Health Access and Quality Index; YLDs, years lived with disability; YLLs, years of life lost.

Case definition

Myelodysplastic, myeloproliferative, and other haemopoietic neoplasms (MDS/MPN) comprise a wide variety of diseases and outcomes, including ICD-10 codes D45 (polycythemia vera), D46 (myelodysplastic syndromes), and D47 (other neoplasms of uncertain behavior of lymphoid, hematopoietic, and related tissue). These were modelled together as a single Level 4 cause for GBD 2023 (the same as for GBD 2017, GBD 2019, and GBD 2021), under the Level 3 cause "Other neoplasms".

Input data

For mortality data, vital registration data (as outlined above) were used for MDS/MPN mortality modelling. We did not use cancer registry data for these neoplasms, as MDS/MPN has only been reported within some cancer registries since 2001 and is recognised to be generally under-reported.[75] For incidence and prevalence data, we used hospital and outpatient prevalence data from various health systems worldwide.[3] As in GBD 2021, clinical data processing and adjustment methods were based on correction factors from hospital data from Poland,[76] using age splines with frequency-based knots. This reduced the size and uncertainty of the correction factors compared with

GBD 2019, which had previously been based on claims data from MarketScan.[71] As in GBD 2019 and GBD 2021, these clinical data were adjusted for the HAQ Index of the location, and inpatient data were adjusted to account for outpatient encounters. Additional details on clinical and claims data processing are available in Section X of the paper "Non-fatal burden of 375 diseases and injuries, risk-attributable burden of 88 risk factors, and healthy life expectancy in 204 countries and territories, including 660 subnational locations, 1990–2023: a systematic analysis for the Global Burden of Disease Study 2023. Lancet (in review)" *(To the editors and reviewers: note that section detail will be finalized once the GBD 2023 Causes of Death Collaborators publication is final)*.[3]

Appendix Table 13: ICD codes mapped to myelodysplastic, myeloproliferative, and other haemopoietic neoplasms in clinical informatics data

GBD cause	ICD-10	ICD-9
Myelodysplastic, myeloproliferative, and other haemopoietic neoplasms	D45, D45.0, D45.9, D46, D46.0, D46.1, D46.2, D46.20, D46.21, D46.22, D46.3, D46.4, D46.5, D46.7, D46.9, D47, D47.0, D47.1, D47.2, D47.3, D47.4, D47.5, D47.7, D47.9	238.4, 238.5, 238.6, 238.7, 238.71, 238.72, 238.73, 238.74, 238.75, 238.76, 238.79, 238.8, 238.9

Abbreviations: GBD, the Global Burden of Diseases, Injuries, and Risk Factors 2023 study; ICD-9, International Classification of Diseases, Ninth Revision; ICD-10, International Classification of Diseases, Tenth Revision.

Modelling strategy

Deaths were estimated using CODEm, as described above and in the appendix to the manuscript "Global burden of 292 causes of death in 204 countries and territories and 660 subnational locations, 1990–2023: a systematic analysis for the Global Burden of Disease Study 2023. Lancet (in review)".[2] As MDS/MPN can be a precursor to leukaemia, our MDS/MPN model in CODEm used many of the same covariate priors as the model for acute myeloid leukaemia (Appendix 1 Table 8).

We modeled the incidence and prevalence of MDS/MPN using clinical data in a prevalence model in DisMod-MR 2.1.[3] For DisMod model specifications, cause-specific mortality rates came from the CODEm model, remission was specified to be zero, and the excess mortality rate was set to be inversely related to the HAQ Index covariate. As in GBD 2019 and GBD 2021, the "cancer, controlled phase" disability weight was assigned for all MDS/MPN cases (Appendix 1 Table 11).

Interpretation of results

MDS/MPN are a diverse grouping of malignancies which are not uniformly characterised. While this broad category of haematological neoplasms is heterogeneous in its components' severity or propensity for transformation to leukaemia, modelling the subtypes of MDS/MPN separately was not feasible for GBD 2023. This is a limitation and an area of desired future improvement as data availability improves.

Limitations

MDS/MPN are a diverse grouping of malignancies which are inconsistently (and often incompletely) captured in cancer registries. Thus, we do not have any data upon which to base MIRs. Our mortality estimates therefore rely on accurate coding of these diverse malignancies in vital registrations systems, while our incidence estimates rely upon data from clinical informatics sources. As the mortality and incidence estimates are generated independently using different data sources and methods, there is more potential for local divergence of MIRs than for most other cancer causes where MIRs are directly estimated using matched cancer registry incidence and mortality data.

Method summary for benign and in situ neoplasms (intestinal; cervical and uterine; and other benign and in situ neoplasms)

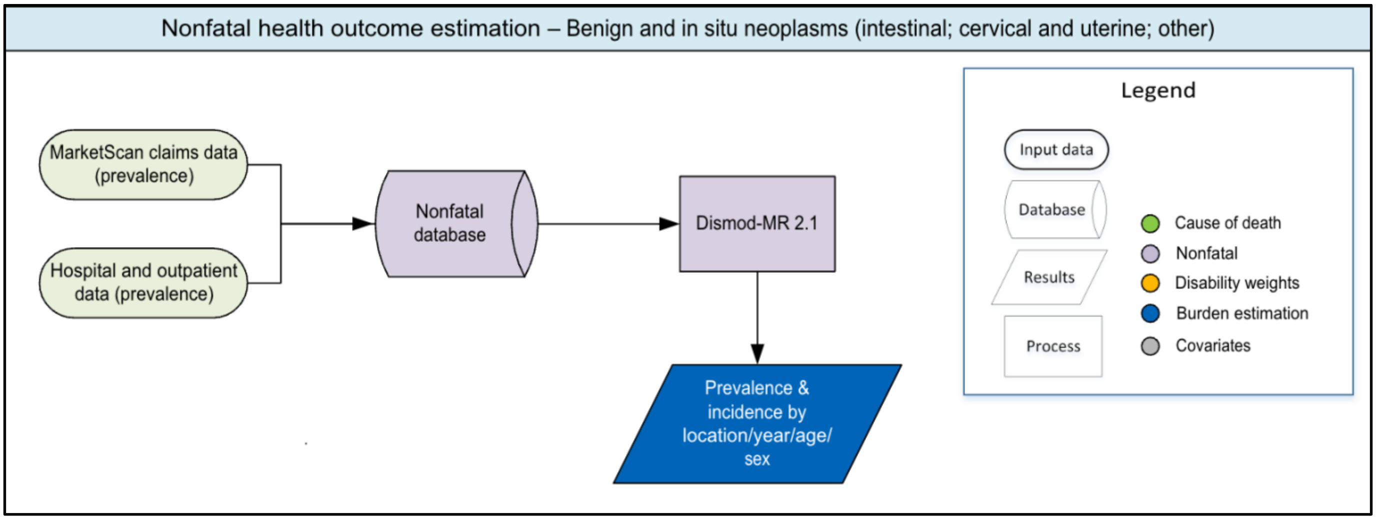

Appendix Figure 5: Flowchart of GBD benign and in situ neoplasms nonfatal estimation. Abbreviations: DisMod-MR, disease model - Bayesian meta-regression.

Case definition

In the GBD framework, the Level 3 cause "Other neoplasms" contains three Level 4 causes of benign and in situ neoplasms: (1) "Benign and in situ intestinal neoplasms"; (2) "Benign and in situ cervical and uterine neoplasms"; and (3) "Other benign and in situ neoplasms". Benign and in situ intestinal neoplasms were defined as any non-invasive intestinal growth of the digestive system beyond the stomach, from duodenum to anus. Benign and in situ cervical and uterine neoplasms were defined as any non-invasive cervical and uterine growth, except for uterine fibroids which are modelled as a separate GBD cause. Other benign and in situ neoplasms were defined as any non-invasive neoplasms not covered by other GBD causes, such as lipomas, benign breast neoplasms, and non-melanoma skin neoplasms other than basal cell carcinoma or squamous cell carcinoma. All three of these benign and in situ neoplasms are, by definition, benign and localised. As such, no deaths or disability were attributed to their occurrence in GBD 2023 for intestinal neoplasms or for cervical and uterine neoplasms. For "Other benign and in situ neoplasms", no deaths or disability were attributed for most neoplasms within this cause, with the exception of non-malignant brain tumours, which can include deaths and disability.

Input data

Of these three causes, we only estimated deaths for "Other benign and in situ neoplasms", as this cause includes neoplasms such as non-malignant central nervous system neoplasms. Though non-malignant, some of these tumours can cause death from physically impairing vital nervous system functions due to where they are located. We estimated these deaths using only vital registration data (as outlined above), as these neoplasms are not consistently captured by cancer registries.

To estimate the incidence and prevalence for each of these three causes, we used hospital and outpatient prevalence data from various health systems worldwide (Appendix 1 Table 14). As in GBD 2021, clinical data processing and adjustment methods were based on correction factors on hospital data from Poland,[76] using age splines with frequency-based knots. This reduced the size and uncertainty of the correction factors compared with GBD 2019, which had previously been based on claims data from MarketScan.[71] As in GBD 2019 and GBD 2021, these clinical data were adjusted for the HAQ Index of the location, and inpatient data were adjusted to account for outpatient encounters. Additional details on clinical and claims data processing are available in Section X of the paper "Non-fatal burden of 375 diseases and injuries, risk-attributable burden of 88 risk factors, and healthy life expectancy in 204 countries and territories, including 660 subnational locations, 1990–2023: a systematic analysis

for the Global Burden of Disease Study 2023. Lancet (in review)" *(To the editors and reviewers: note that section detail will be finalized once the GBD 2023 Causes of Death Collaborators publication is final)*.[3]

Appendix Table 14: ICD codes mapped to benign and in situ neoplasms in clinical informatics data

GBD cause	ICD-10	ICD-9
Benign and in situ intestinal neoplasms	D01, D01.0, D01.1, D01.2, D01.3, D01.4, D01.40, D01.49, D01.5, D01.7, D01.9, D12, D12.0, D12.1, D12.2, D12.3, D12.4, D12.5, D12.6, D12.7, D12.8, D12.9, D13.2, D13.3, D13.30, D13.39, D13.9, D37.2, D37.3, D37.4, D37.5, D3A.010, D3A.011, D3A.012, D3A.019, D3A.020, D3A.021, D3A.023, D3A.026, D3A.029, E34.0, K62.0, K62.1, K63.5	209.4, 209.40, 209.41, 209.42, 209.43, 209.5, 209.50, 209.51, 209.52, 209.53, 209.54, 209.55, 209.56, 209.57, 209.66, 209.67, 211.2, 211.3, 211.4, 230.3, 230.4, 230.5, 230.6, 230.7, 235.2, 259.2, 569.0
Benign and in situ cervical and uterine neoplasms	D06, D06.0, D06.1, D06.7, D06.9, D07.0, D26.0, D26.1, D26.7, D26.9, D39.0, N84.0, N84.1, N87, N87.0, N87.1, N87.2, N87.9	219, 219.0, 219.1, 219.8, 219.9, 233.1, 233.2, 236.0, 622.1, 622.10, 622.11, 622.12, 622.2, 622.7
Other benign and in situ neoplasms	D00, D00.0, D00.00, D00.01, D00.02, D00.03, D00.04, D00.05, D00.06, D00.07, D00.08, D00.1, D00.2, D02, D02.0, D02.1, D02.2, D02.20, D02.21, D02.22, D02.3, D02.4, D02.9, D03, D03.0, D03.1, D03.10, D03.11, D03.12, D03.2, D03.20, D03.21, D03.22, D03.3, D03.30, D03.39, D03.4, D03.5, D03.51, D03.52, D03.59, D03.6, D03.60, D03.61, D03.62, D03.7, D03.70, D03.71, D03.72, D03.8, D03.9, D04, D04.0, D04.1, D04.10, D04.11, D04.12, D04.2, D04.20, D04.21, D04.22, D04.3, D04.30, D04.39, D04.4, D04.5, D04.6, D04.60, D04.61, D04.62, D04.7, D04.70, D04.71, D04.72, D04.8, D04.9, D05, D05.0, D05.00, D05.01, D05.02, D05.1, D05.10, D05.11, D05.12, D05.7, D05.8, D05.80, D05.81, D05.82, D05.9, D05.90, D05.91, D05.92, D07, D07.1, D07.2, D07.3, D07.30, D07.39, D07.4, D07.5, D07.6, D07.60, D07.61, D07.69, D08, D09, D09.0, D09.1, D09.10, D09.19, D09.2, D09.20, D09.21, D09.22, D09.3, D09.7, D09.8, D09.9, D10, D10.0, D10.1, D10.2, D10.3, D10.30, D10.39, D10.4, D10.5, D10.6, D10.7, D10.9, D11, D11.0, D11.7, D11.9, D13, D13.0, D13.1, D13.4, D13.5, D13.6, D13.7, D14, D14.0, D14.1, D14.2, D14.3, D14.30, D14.31, D14.32, D14.4, D15, D15.0, D15.1, D15.2, D15.7, D15.9, D16, D16.0, D16.00, D16.01, D16.02, D16.1, D16.10, D16.11, D16.12, D16.2, D16.20, D16.21, D16.22, D16.3, D16.30, D16.31, D16.32, D16.4, D16.5, D16.6, D16.7, D16.8, D16.9, D17, D17.0, D17.1, D17.2, D17.20, D17.21, D17.22, D17.23, D17.24, D17.3, D17.30, D17.39, D17.4, D17.5, D17.6, D17.7, D17.71, D17.72, D17.79, D17.9, D18, D18.0, D18.00, D18.01, D18.03, D18.09, D18.1, D19, D19.0, D19.1, D19.7, D19.9, D20, D20.0, D20.1, D20.9, D21, D21.0, D21.1, D21.10, D21.11, D21.12, D21.2, D21.20, D21.21, D21.22, D21.3, D21.4, D21.5, D21.6, D21.9, D22, D22.0, D22.1, D22.10, D22.11, D22.12, D22.2, D22.20, D22.21, D22.22, D22.3, D22.30, D22.39, D22.4, D22.5, D22.6, D22.60, D22.61, D22.62, D22.7, D22.70, D22.71, D22.72, D22.9, D23, D23.0, D23.1, D23.10, D23.11, D23.12, D23.2, D23.20, D23.21, D23.22, D23.3, D23.30, D23.39, D23.4, D23.5, D23.6, D23.60, D23.61, D23.62, D23.7, D23.70, D23.71, D23.72, D23.9, D24, D24.0, D24.1, D24.2, D24.9, D27, D27.0, D27.1, D27.9, D28, D28.0, D28.1, D28.2, D28.7, D28.9, D29, D29.0, D29.1, D29.2, D29.20, D29.21, D29.22, D29.3, D29.30, D29.31, D29.32, D29.4, D29.7, D29.8, D29.9, D30, D30.0, D30.00, D30.01, D30.02, D30.1, D30.10, D30.11, D30.12, D30.2, D30.20, D30.21, D30.22, D30.3, D30.4, D30.7, D30.8, D30.9, D31, D31.0, D31.00, D31.01, D31.02, D31.1, D31.10, D31.11, D31.12, D31.2, D31.20, D31.21, D31.22, D31.3, D31.30, D31.31, D31.32, D31.4, D31.40, D31.41, D31.42, D31.5, D31.50, D31.51, D31.52, D31.6, D31.60, D31.61, D31.62, D31.9, D31.90, D31.91, D31.92, D34, D34.0, D34.9, D35, D35.0, D35.00, D35.01, D35.02, D35.1, D35.5, D35.6, D35.7, D35.8, D35.9, D36, D36.0, D36.1, D36.10, D36.11, D36.12, D36.13, D36.14, D36.15, D36.16, D36.17, D36.7, D36.9, D36.91, D36.92, D37, D37.0, D37.01, D37.02, D37.03, D37.030, D37.031, D37.032, D37.039, D37.04, D37.05, D37.09, D37.1, D37.6, D37.7, D37.8, D37.9, D38, D38.0, D38.1, D38.2, D38.3, D38.4, D38.5, D38.6, D39, D39.1, D39.10, D39.11, D39.12, D39.2, D39.7, D39.8, D39.9, D3A.00, D3A.090, D3A.092, D3A.094, D3A.098, D3A.8, D4, D40, D40.0, D40.1, D40.10, D40.11, D40.12, D40.7, D40.8, D40.9, D41, D41.0, D41.00, D41.01, D41.02, D41.1, D41.10, D41.11, D41.12, D41.2, D41.20, D41.21, D41.22, D41.3, D41.4, D41.7, D41.8, D41.9, D44, D44.0, D44.1, D44.10, D44.11, D44.12, D44.2, D44.6, D44.7, D44.8, D44.9, D48, D48.0, D48.1, D48.2, D48.3, D48.4, D48.5, D48.6, D48.60, D48.61, D48.62, D48.7, D48.9, D49, D49.0, D49.1, D49.2, D49.3, D49.4, D49.5, D49.6, D49.7, D49.8, D49.81, D49.89, D49.9, N60, N60.0, N60.01, N60.02, N60.09, N60.1, N60.11, N60.12, N60.19, N60.2, N60.21, N60.22, N60.29, N60.3, N60.31, N60.32, N60.39, N60.4, N60.41, N60.42, N60.49, N60.8, N60.81, N60.82, N60.89, N60.9, N60.91, N60.92, N60.99	209.61, 209.63, 209.64, 209.65, 210.0, 210.1, 210.2, 210.3, 210.4, 210.5, 210.6, 210.7, 210.8, 210.9, 211, 211.0, 211.1, 211.5, 211.6, 211.7, 211.8, 211.9, 212, 212.0, 212.1, 212.2, 212.3, 212.4, 212.5, 212.6, 212.7, 212.8, 212.9, 213, 213.0, 213.1, 213.2, 213.3, 213.4, 213.5, 213.6, 213.7, 213.8, 213.9, 214, 214.0, 214.1, 214.2, 214.3, 214.4, 214.8, 214.9, 215, 215.0, 215.2, 215.3, 215.4, 215.5, 215.6, 215.7, 215.8, 215.9, 216, 216.0, 216.1, 216.2, 216.3, 216.4, 216.5, 216.6, 216.7, 216.8, 216.9, 217, 217.0, 217.8, 220, 220.0, 220.9, 221, 221.0, 221.1, 221.2, 221.8, 221.9, 222, 222.0, 222.1, 222.2, 222.3, 222.4, 222.8, 222.9, 223, 223.0, 223.1, 223.2, 223.3, 223.8, 223.81, 223.89, 223.9, 224, 224.0, 224.1, 224.2, 224.3, 224.4, 224.5, 224.6, 224.7, 224.8, 224.9, 226, 226.0, 226.9, 227, 227.0, 227.1, 227.5, 227.6, 227.8, 227.9, 228, 228.0, 228.00, 228.01, 228.03, 228.04, 228.09, 228.1, 228.9, 229, 229.0, 229.1, 229.8, 229.9, 230, 230.0, 230.1, 230.2, 230.8, 230.9, 231, 231.0, 231.1, 231.2, 231.8, 231.9, 232, 232.0, 232.1, 232.2, 232.3, 232.4, 232.5, 232.6, 232.7, 232.8, 232.9, 233, 233.0, 233.3, 233.30, 233.31, 233.32, 233.39, 233.4, 233.5, 233.6, 233.7, 233.9, 234, 234.0, 234.5, 234.8, 234.9, 235, 235.0, 235.1, 235.3, 235.4, 235.5, 235.6, 235.7, 235.8, 235.9, 236, 236.1, 236.2, 236.3, 236.4, 236.5, 236.6, 236.7, 236.9, 236.90, 236.91, 236.99, 237, 237.2, 237.3, 237.4, 237.70, 237.71, 238, 238.0, 238.1, 238.2, 238.3, 239, 239.0, 239.1, 239.2, 239.3, 239.4, 239.5, 239.8, 239.81, 239.89, 239.9, 610, 610.1, 610.8, 610.9

Abbreviations: GBD, the Global Burden of Diseases, Injuries, and Risk Factors 2023 study; ICD-9, International Classification of Diseases, Ninth Revision; ICD-10, International Classification of Diseases, Tenth Revision.

Modelling strategy

All three of these benign and in situ neoplasms are by definition non-invasive and localised. As such, no deaths or disability were attributed to their occurrence in GBD 2023. The exception was for "other benign and in situ neoplasms", where deaths were included for a subset of this broad category that includes neoplasms such as non-malignant brain and central nervous system neoplasms. Deaths for this cause were modelled in CODEm, as described above. Because these deaths represent only a subset of the total neoplasms included in this broader cause, we did not assign any disability to these causes for GBD 2023.

For incidence and prevalence estimation, clinical informatics data were input into cause-specific DisMod-MR 2.1[3] models for each benign and in situ neoplasm cause. In the DisMod model for benign and in situ intestinal neoplasms, excess mortality rate was specified to be zero, and remission was allowed to vary from 0 to 1. In the DisMod model for benign and in situ cervical and uterine neoplasms, excess mortality rate was specified to be zero, and remission was allowed to vary from 0 to 0.75. In the DisMod model for other benign and in situ neoplasms, excess mortality rate was specified to be zero, and remission was allowed to vary from 0 to 1. As in GBD 2021, no disability weight was assigned to these prevalence estimates for these causes. This is an area of desired future improvement as data availability improves.

Interpretation of results

These causes represent benign and in situ neoplasms which have not become malignant. As such, some stakeholders will choose to exclude these causes when considering the total burden from cancer. Estimating the incidence and prevalence of these causes is important, however, given their commonality and that some of these neoplasms can progress to invasive cancer. The "Other benign and in situ neoplasms" cause is particularly broad, encompassing a large number of diagnoses which may go undiagnosed.

Limitations

These benign and in situ neoplasms causes include a diverse grouping of neoplasms which are often excluded from cancer registries. Because of this, our incidence estimates rely upon data from clinical informatics sources. As these data are sparse, incidence and prevalence estimates should be considered along with their wide uncertainty. As many benign and in situ neoplasms go undiagnosed, and our input data relies upon clinical diagnoses, our results may actually represent an underestimate of true subclinical incidence and prevalence.

Additional methodology tables

Appendix Table 15: World Bank income group used in GBD 2023 for each country

Location	World Bank Income Group
Afghanistan	Low Income
Albania	Upper Middle Income
Algeria	Upper Middle Income
American Samoa	High Income
Andorra	High Income
Angola	Lower Middle Income
Antigua and Barbuda	High Income
Argentina	Upper Middle Income
Armenia	Upper Middle Income
Australia	High Income
Austria	High Income
Azerbaijan	Upper Middle Income
Bahamas	High Income
Bahrain	High Income
Bangladesh	Lower Middle Income
Barbados	High Income
Belarus	Upper Middle Income
Belgium	High Income
Belize	Upper Middle Income
Benin	Lower Middle Income
Bermuda	High Income
Bhutan	Lower Middle Income
Bolivia (Plurinational State of)	Lower Middle Income
Bosnia and Herzegovina	Upper Middle Income
Botswana	Upper Middle Income
Brazil*	Upper Middle Income
Brunei Darussalam	High Income
Bulgaria	High Income
Burkina Faso	Low Income
Burundi	Low Income
Cabo Verde	Lower Middle Income
Cambodia	Lower Middle Income
Cameroon	Lower Middle Income
Canada	High Income
Central African Republic	Low Income
Chad	Low Income
Chile	High Income
China*	Upper Middle Income
Colombia	Upper Middle Income
Comoros	Lower Middle Income
Congo	Lower Middle Income
Cook Islands	NA
Costa Rica	Upper Middle Income
Côte d'Ivoire	Lower Middle Income
Croatia	High Income
Cuba	Upper Middle Income
Cyprus	High Income
Czechia	High Income
Democratic People's Republic of Korea	Low Income
Democratic Republic of the Congo	Low Income
Denmark	High Income
Djibouti	Lower Middle Income
Dominica	Upper Middle Income
Dominican Republic	Upper Middle Income
Ecuador	Upper Middle Income
Egypt	Lower Middle Income
El Salvador	Upper Middle Income
Equatorial Guinea	Upper Middle Income
Eritrea	Low Income
Estonia	High Income
Eswatini	Lower Middle Income
Ethiopia*	Low Income
Fiji	Upper Middle Income
Finland	High Income

France	High Income
Gabon	Upper Middle Income
Gambia	Low Income
Georgia	Upper Middle Income
Germany	High Income
Ghana	Lower Middle Income
Greece	High Income
Greenland	High Income
Grenada	Upper Middle Income
Guam	High Income
Guatemala	Upper Middle Income
Guinea	Lower Middle Income
Guinea-Bissau	Low Income
Guyana	High Income
Haiti	Lower Middle Income
Honduras	Lower Middle Income
Hungary	High Income
Iceland	High Income
India*	Lower Middle Income
Indonesia*	Upper Middle Income
Iran (Islamic Republic of)*	Upper Middle Income
Iraq	Upper Middle Income
Ireland	High Income
Israel	High Income
Italy*	High Income
Jamaica	Upper Middle Income
Japan*	High Income
Jordan	Lower Middle Income
Kazakhstan	Upper Middle Income
Kenya*	Lower Middle Income
Kiribati	Lower Middle Income
Kuwait	High Income
Kyrgyzstan	Lower Middle Income
Lao People's Democratic Republic	Lower Middle Income
Latvia	High Income
Lebanon	Lower Middle Income
Lesotho	Lower Middle Income
Liberia	Low Income
Libya	Upper Middle Income
Lithuania	High Income
Luxembourg	High Income
Madagascar	Low Income
Malawi	Low Income
Malaysia	Upper Middle Income
Maldives	Upper Middle Income
Mali	Low Income
Malta	High Income
Marshall Islands	Upper Middle Income
Mauritania	Lower Middle Income
Mauritius	Upper Middle Income
Mexico*	Upper Middle Income
Micronesia (Federated States of)	Lower Middle Income
Monaco	High Income
Mongolia	Upper Middle Income
Montenegro	Upper Middle Income
Morocco	Lower Middle Income
Mozambique	Low Income
Myanmar	Lower Middle Income
Namibia	Upper Middle Income
Nauru	High Income
Nepal	Lower Middle Income
Netherlands	High Income
New Zealand*	High Income
Nicaragua	Lower Middle Income
Niger	Low Income
Nigeria*	Lower Middle Income
Niue	NA
North Macedonia	Upper Middle Income
Northern Mariana Islands	High Income

Norway*	High Income
Oman	High Income
Pakistan*	Lower Middle Income
Palau	High Income
Palestine	Lower Middle Income
Panama	High Income
Papua New Guinea	Lower Middle Income
Paraguay	Upper Middle Income
Peru	Upper Middle Income
Philippines*	Lower Middle Income
Poland*	High Income
Portugal	High Income
Puerto Rico	High Income
Qatar	High Income
Republic of Korea	High Income
Republic of Moldova	Upper Middle Income
Romania	High Income
Russian Federation*	High Income
Rwanda	Low Income
Saint Kitts and Nevis	High Income
Saint Lucia	Upper Middle Income
Saint Vincent and the Grenadines	Upper Middle Income
Samoa	Lower Middle Income
San Marino	High Income
Sao Tome and Principe	Lower Middle Income
Saudi Arabia	High Income
Senegal	Lower Middle Income
Serbia	Upper Middle Income
Seychelles	High Income
Sierra Leone	Low Income
Singapore	High Income
Slovakia	High Income
Slovenia	High Income
Solomon Islands	Lower Middle Income
Somalia	Low Income
South Africa*	Upper Middle Income
South Sudan	Low Income
Spain	High Income
Sri Lanka	Lower Middle Income
Sudan	Low Income
Suriname	Upper Middle Income
Sweden	High Income
Switzerland	High Income
Syrian Arab Republic	Low Income
Taiwan**	High Income
Tajikistan	Lower Middle Income
Thailand	Upper Middle Income
Timor-Leste	Lower Middle Income
Togo	Low Income
Tokelau	NA
Tonga	Upper Middle Income
Trinidad and Tobago	High Income
Tunisia	Lower Middle Income
Türkiye	Upper Middle Income
Turkmenistan	Upper Middle Income
Tuvalu	Upper Middle Income
Uganda	Low Income
Ukraine*	Upper Middle Income
United Arab Emirates	High Income
United Kingdom*	High Income
United Republic of Tanzania	Lower Middle Income
United States of America*	High Income
United States Virgin Islands	High Income
Uruguay	High Income
Uzbekistan	Lower Middle Income
Vanuatu	Lower Middle Income
Venezuela (Bolivarian Republic of)	Upper Middle Income
Viet Nam	Lower Middle Income
Yemen	Low Income

Zambia	Lower Middle Income
Zimbabwe	Lower Middle Income

Abbreviations: GBD, the Global Burden of Diseases, Injuries, and Risk Factors 2023 study; NA, not applicable (no World Bank income group classification available). World Bank income group classification was based on World Bank fiscal year 2025 using data from calendar year 2023.[77] *Location is estimated subnationally in GBD 2023. **United Nations convention recognizes Taiwan as a province of China.

Appendix Table 16: GBD location hierarchy: 7 GBD super regions, 21 GBD regions, and 204 countries and territories

GBD hierarchy	Location
**GBD super region**	**Central Europe, Eastern Europe, and Central Asia**
GBD region	*Central Asia*
	Armenia
	Azerbaijan
	Georgia
	Kazakhstan
	Kyrgyzstan
	Mongolia
	Tajikistan
	Turkmenistan
	Uzbekistan
GBD region	*Central Europe*
	Albania
	Bosnia and Herzegovina
	Bulgaria
	Croatia
	Czechia
	Hungary
	Montenegro
	North Macedonia
	Poland
	Romania
	Serbia
	Slovakia
	Slovenia
GBD region	*Eastern Europe*
	Belarus
	Estonia
	Latvia
	Lithuania
	Republic of Moldova
	Russian Federation
	Ukraine
**GBD super region**	**High-income**
GBD region	*Australasia*
	Australia
	New Zealand
GBD region	*High-income Asia Pacific*
	Brunei Darussalam
	Japan
	Republic of Korea
	Singapore
GBD region	*High-income North America*
	Canada
	Greenland
	United States of America
GBD region	*Southern Latin America*
	Argentina
	Chile
	Uruguay
GBD region	*Western Europe*
	Andorra
	Austria
	Belgium
	Cyprus
	Denmark
	Finland
	France
	Germany
	Greece
	Iceland
	Ireland

	Israel
	Italy
	Luxembourg
	Malta
	Monaco
	Netherlands
	Norway
	Portugal
	San Marino
	Spain
	Sweden
	Switzerland
	United Kingdom
**GBD super region**	**Latin America and Caribbean**
GBD region	*Andean Latin America*
	Bolivia (Plurinational State of)
	Ecuador
	Peru
GBD region	*Caribbean*
	Antigua and Barbuda
	Bahamas
	Barbados
	Belize
	Bermuda
	Cuba
	Dominica
	Dominican Republic
	Grenada
	Guyana
	Haiti
	Jamaica
	Puerto Rico
	Saint Kitts and Nevis
	Saint Lucia
	Saint Vincent and the Grenadines
	Suriname
	Trinidad and Tobago
	United States Virgin Islands
GBD region	*Central Latin America*
	Colombia
	Costa Rica
	El Salvador
	Guatemala
	Honduras
	Mexico
	Nicaragua
	Panama
	Venezuela (Bolivarian Republic of)
GBD region	*Tropical Latin America*
	Brazil
	Paraguay
**GBD super region**	**North Africa and Middle East**
GBD region	*North Africa and Middle East*
	Afghanistan
	Algeria
	Bahrain
	Egypt
	Iran (Islamic Republic of)
	Iraq
	Jordan
	Kuwait
	Lebanon
	Libya
	Morocco

	Oman
	Palestine
	Qatar
	Saudi Arabia
	Sudan
	Syrian Arab Republic
	Tunisia
	Türkiye
	United Arab Emirates
	Yemen
**GBD super region**	**South Asia**
GBD region	*South Asia*
	Bangladesh
	Bhutan
	India
	Nepal
	Pakistan
**GBD super region**	**Southeast Asia, East Asia, and Oceania**
GBD region	*East Asia*
	China
	Democratic People's Republic of Korea
	Taiwan*
GBD region	*Oceania*
	American Samoa
	Cook Islands
	Fiji
	Guam
	Kiribati
	Marshall Islands
	Micronesia (Federated States of)
	Nauru
	Niue
	Northern Mariana Islands
	Palau
	Papua New Guinea
	Samoa
	Solomon Islands
	Tokelau
	Tonga
	Tuvalu
	Vanuatu
GBD region	*Southeast Asia*
	Cambodia
	Indonesia
	Lao People's Democratic Republic
	Malaysia
	Maldives
	Mauritius
	Myanmar
	Philippines
	Seychelles
	Sri Lanka
	Thailand
	Timor-Leste
	Viet Nam
**GBD super region**	**Sub-Saharan Africa**
GBD region	*Central Sub-Saharan Africa*
	Angola
	Central African Republic
	Congo
	Democratic Republic of the Congo
	Equatorial Guinea
	Gabon
GBD region	*Eastern Sub-Saharan Africa*

	Burundi
	Comoros
	Djibouti
	Eritrea
	Ethiopia
	Kenya
	Madagascar
	Malawi
	Mozambique
	Rwanda
	Somalia
	South Sudan
	Uganda
	United Republic of Tanzania
	Zambia
GBD region	*Southern Sub-Saharan Africa*
	Botswana
	Eswatini
	Lesotho
	Namibia
	South Africa
	Zimbabwe
GBD region	*Western Sub-Saharan Africa*
	Benin
	Burkina Faso
	Cabo Verde
	Cameroon
	Chad
	Côte d'Ivoire
	Gambia
	Ghana
	Guinea
	Guinea-Bissau
	Liberia
	Mali
	Mauritania
	Niger
	Nigeria
	Sao Tome and Principe
	Senegal
	Sierra Leone
	Togo

Countries listed alphabetically by GBD super region first, then by GBD region. *United Nations convention recognizes Taiwan as a province of China.

References

1 Global Burden of Disease 2019 Cancer Collaboration, Brian L. Gaw, Rixing Xu, *et al.* Cancer Incidence, Mortality, Years of Life Lost, Years Lived With Disability, and Disability-Adjusted Life Years for 29 Cancer Groups From 2010 to 2019: A Systematic Analysis for the Global Burden of Disease Study 2019. *JAMA Oncology* 2022; **8**: 420–44.

2 GBD 2023 Causes of Death Collaborators. Global burden of 292 causes of death in 204 countries and territories and 660 subnational locations, 1990–2023: a systematic analysis for the Global Burden of Disease Study 2023. Lancet (in review).

3 GBD 2023 Disease and Injury and Risk Factor Collaborators. Non-fatal burden of 375 diseases and injuries, risk-attributable burden of 88 risk factors, and healthy life expectancy in 204 countries and territories, including 660 subnational locations, 1990–2023: a systematic analysis for the Global Burden of Disease Study 2023. Lancet (in review).

4 GBD 2021 Risk Factor Collaborators. Global burden and strength of evidence for 88 risk factors in 204 countries and 811 subnational locations, 1990-2021: a systematic analysis for the Global Burden of Disease Study 2021. *The Lancet* DOI:10.1016/S0140-6736(24)00933-4.

5 GBD 2021 Forecasting Collaborators. Burden of disease scenarios for 204 countries and territories, 2022–2050: a forecasting analysis for the Global Burden of Disease Study 2021. *Lancet* 2024; **403**: 2204–56.

6 GBD 2023 Demographics Collaborators. Global age-sex-specific all-cause mortality and life expectancy estimates for 204 countries and territories and 660 subnational locations, 1950–2023: a demographic analysis for the Global Burden of Disease Study 2023. Lancet (in review).

7 Stevens G, Alkema L, Black R, et al. Guidelines for Accurate and Transparent Health Estimates Reporting: the GATHER statement. *The Lancet* 2016; **388**: 19–23.

8 World Health Organization. International Classification of Diseases (ICD). 2018. http://www.who.int/classifications/icd/en/ (accessed Feb 25, 2020).

9 Ibrahim Khalil A, Franceschi S, de Martel C, Bray F, Clifford GM. Burden of Kaposi sarcoma according to HIV status: A systematic review and global analysis. *International Journal of Cancer* 2022; **150**: 1948–57.

10 GBD 2021 Causes of Death Collaborators. Global burden of 288 causes of death and life expectancy decomposition in 204 countries and territories and 811 subnational locations, 1990-2021: a systematic analysis for the Global Burden of Disease Study 2021. *Lancet* 2024; **403**: 2100–32.

11 Steliarova-Foucher E, Stiller C, Lacour B, Kaatsch P. International Classification of Childhood Cancer, third edition. *Cancer* 2005; **103**: 1457–67.

12 Steliarova-Foucher E, Colombet M, Ries L, Rous B, Stiller C. Classification of tumours. In: International Incidence of Childhood Cancer, Volume 3. Lyon: International Agency for Research on Cancer, in press. https://iicc.iarc.fr/classification/citation/ (accessed June 27, 2023).

13 Surveillance, Epidemiology, and End Results (SEER) Program (www.Seer.Cancer.Gov) SEER*Stat Database: Incidence - SEER 18.

14 Doll R, Payne P, Waterhouse J. Cancer Incidence in Five Continents, Volume I. Geneva: Union Internationale Contre le Cancer https://publications.iarc.fr/Non-Series-Publications/Other-Non-Series-Publications/Cancer-Incidence-In-Five-Continents-Volume-I-1966 (accessed Feb 24, 2021).

15 Doll R, Muir C, Waterhouse J. Cancer Incidence in Five Continents, Volume II. Geneva: Union Internationale Contre le Cancer, 1970.

16 Waterhouse J, Muir C, Correa P, Powell J. Cancer Incidence in Five Continents III. Lyon: IARC; 1976.

17 Waterhouse J, Muir C, Shanmugaratnam K, Powell J. Cancer Incidence in Five Continents IV. Lyon: IARC; 1982.

18 Muir C, Mack T, Powell J, Whelan S. Cancer Incidence in Five Continents V. Lyon: IARC; 1987.

19 Parkin D, Muir C, Whelan S, Gao Y, Ferlay J, Powell J. Cancer Incidence in Five Continents VI. Lyon: IARC; 1992.

20 Parkin D, Whelan S, Ferlay J, Raymond L, Young J. Cancer Incidence in Five Continents VII. Lyon: IARC; 1997.

21 Parkin D, Whelan S, Ferlay J, Teppo L, Thomas D. Cancer Incidence in Five Continents VIII. Lyon: IARC; 2002.

22 Curado M, Edwards B, Shin H, et al. Cancer Incidence in Five Continents IX. Lyon: IARC; 2007. http://www.iarc.fr/en/publ.

23 Forman D, Bray F, Brewster D, et al. Cancer Incidence in Five Continents X. http://ci5.iarc.fr. Published 2013. .

24 Bray F, Colombet M, Mery L, *et al.*, editors. Cancer Incidence in Five Continents, Volumne XI. Lyon: International Agency for Research on Cancer, 2017.

25 Steliarova-Foucher E, O'Callaghan M, Ferlay J, *et al.* The European Cancer Observatory: A new data resource. *Eur J Cancer* 2015; **51**: 1131–43.

26 Engholm G, Ferlay J, Christensen N, *et al.* NORDCAN--a Nordic tool for cancer information, planning, quality control and research. *Acta Oncol* 2010; **49**: 725–36.

27 Steliarova-Foucher E, Colombet M, Ries LAG, *et al.* International incidence of childhood cancer, 2001–10: a population-based registry study. *The Lancet Oncology* 2017; **18**: 719–31.

28 Surveillance, Epidemiology, and End Results (SEER) Program (www.seer.cancer.gov) SEER*Stat Database: Incidence - SEER 18 Regs Research Data + Hurricane Katrina Impacted Louisiana Cases, Nov 2017 Sub (1973-2015 varying) - Linked To County Attributes - Total U.S., 1969-2016 Counties, National Cancer Institute, DCCPS, Surveillance Research Program, released April 2018, based on the November 2017 submission.

29 Barber RM, Fullman N, Sorensen RJD, *et al.* Healthcare Access and Quality Index based on mortality from causes amenable to personal health care in 195 countries and territories, 1990–2015: a novel analysis from the Global Burden of Disease Study 2015. *The Lancet* 2017; **390**: 231–66.

30 ihmeuw-msca/regmod. 2024; published online July 16. https://github.com/ihmeuw-msca/regmod (accessed April 11, 2025).

31 Foreman KJ, Lozano R, Lopez AD, Murray CJ. Modeling causes of death: an integrated approach using CODEm. *Popul Health Metr* 2012; **10**: 1.

32 de Martel C, Maucort-Boulch D, Plummer M, Franceschi S. World-wide relative contribution of hepatitis B and C viruses in hepatocellular carcinoma. *Hepatology* 2015; **62**: 1190–200.

33 Hong TP, Gow P, Fink M, *et al.* Novel population-based study finding higher than reported hepatocellular carcinoma incidence suggests an updated approach is needed. *Hepatology* 2016; **63**: 1205–12.

34 Global Burden of Disease Cancer Collaboration. Global, Regional, and National Cancer Incidence, Mortality, Years of Life Lost, Years Lived With Disability, and Disability-Adjusted Life-Years for 29 Cancer Groups, 1990 to 2017: A Systematic Analysis for the Global Burden of Disease Study. *JAMA Oncology* 2019; **5**: 1749–68.

35 Asadzadeh Vostakolaei F, Karim-Kos HE, Janssen-Heijnen MLG, Visser O, Verbeek ALM, Kiemeney LALM. The validity of the mortality to incidence ratio as a proxy for site-specific cancer survival. *Eur J Public Health* 2011; **21**: 573–7.

36 SEER*Stat Software.; 2014. http://seer.cancer.gov/seerstat/.

37 Surveillance, Epidemiology, and End Results (SEER) Program (www.seer.cancer.gov) SEER*Stat Database: Mortality - All COD, Aggregated With State, Total U.S. (1969-2015) <Katrina/Rita Population Adjustment>, National Cancer Institute, DCCPS, Surveillance Research Program, released December 2017. Underlying mortality data provided by NCHS (www.cdc.gov/nchs). .

38 Surveillance, Epidemiology, and End Results (SEER) Program (www.seer.cancer.gov) SEER*Stat Database: Incidence - SEER 9 Regs Research Data, Nov 2016 Sub (1973-2014) <Katrina/Rita Population Adjustment> - Linked To County Attributes - Total U.S., 1969-2015 Counties, National Cancer Institute, DCCPS, Surveillance Research Program, released April 2017, based on the November 2016 submission.

39 Global Burden of Disease Cancer Collaboration. The Global Burden of Cancer 2013. *JAMA Oncology* 2015; **1**: 505–27.

40 Global Burden of Disease Cancer Collaboration. Global, Regional, and National Cancer Incidence, Mortality, Years of Life Lost, Years Lived With Disability, and Disability-Adjusted Life-years for 32 Cancer Groups, 1990 to 2015: A Systematic Analysis for the Global Burden of Disease Study. *JAMA Oncology* 2017; **3**: 524–48.

41 Global Burden of Disease Cancer Collaboration. Global, Regional, and National Cancer Incidence, Mortality, Years of Life Lost, Years Lived With Disability, and Disability-Adjusted Life-Years for 29 Cancer Groups, 1990 to 2016: A Systematic Analysis for the Global Burden of Disease Study. *JAMA Oncology* 2018; **4**: 1553–68.

42 GBD 2021 Diseases and Injuries Collaborators. Global incidence, prevalence, years lived with disability (YLDs), disability-adjusted life-years (DALYs), and healthy life expectancy (HALE) for 371 diseases and injuries in 204 countries and territories and 811 subnational locations, 1990-2021: a systematic analysis for the Global Burden of Disease Study 2021. *Lancet* 2024; **403**: 2133–61.

43 Neal RD, Din NU, Hamilton W, *et al.* Comparison of cancer diagnostic intervals before and after implementation of NICE guidelines: analysis of data from the UK General Practice Research Database. *British Journal of Cancer* 2014; **110**: 584–92.

44 Surveillance, Epidemiology, and End Results (SEER) Program (www.seer.cancer.gov) SEER*Stat Database: Incidence - SEER 18 Regs Research Data + Hurricane Katrina Impacted Louisiana Cases, Nov 2012 Sub (1973-2010 varying) - Linked To County Attributes - Total U.S., 1969-2011 Counties, National Cancer Institute, DCCPS, Surveillance Research Program, Surveillance Systems Branch, released April 2013, based on the November 2012 submission.

45 Allgar VL, Neal RD. Delays in the diagnosis of six cancers: analysis of data from the National Survey of NHS Patients: Cancer. *Br J Cancer* 2005; **92**: 1959–70.

46 Neal RD, Cannings-John R, Hood K, *et al.* Excision of malignant melanomas in North Wales: effect of location and surgeon on time to diagnosis and quality of excision. *Family Practice* 2008; **25**: 221–7.

47 Kewalramani T, Nimer SD, Zelenetz AD, *et al.* Progressive disease following autologous transplantation in patients with chemosensitive relapsed or primary refractory Hodgkin's disease or aggressive non-Hodgkin's lymphoma. *Bone Marrow Transplant* 2003; **32**: 673–9.

48 Esteban D, Tovar N, Jiménez R, *et al.* Patients with relapsed/refractory chronic lymphocytic leukaemia may benefit from inclusion in clinical trials irrespective of the therapy received: a case-control retrospective analsysis. *Blood Cancer J* 2015; **5**: e356.

49 Kopelman JE, McLean IW, Rosenberg SH. Multivariate analysis of risk factors for metastasis in retinoblastoma treated by enucleation. *Ophthalmology* 1987; **94**: 371–7.

50 National Cancer Institute (United States). United States SEER Cancer Data 1973-2010. Bethesda, United States: National Cancer Institute (United States).

51 National Center for Health Statistics (NCHS), Centers for Disease Control and Prevention (CDC), US Census Bureau. United States National Hospital Discharge Survey. Hyattsville, United States: National Center for Health Statistics (NCHS), Centers for Disease Control and Prevention (CDC).

52 Canadian Institute for Health Information (CIHI). Canada Discharge Abstract Database 1994-2009. Ottawa, Canada: Canadian Institute for Health Information (CIHI).

53 Ministry of Health (Mexico). Mexico Ministry of Health Hospital Discharges 2000-2012. Mexico City, Mexico: Ministry of Health (Mexico).

54 ICD - ICD-9-CM - International Classification of Diseases, Ninth Revision, Clinical Modification. 2021; published online Nov 3. https://www.cdc.gov/nchs/icd/icd9cm.htm (accessed Dec 15, 2022).

55 Canova C, Giorato E, Roveron G, Turrini P, Zanotti R. Validation of a stoma-specific quality of life questionnaire in a sample of patients with colostomy or ileostomy. *Colorectal Dis* 2013; **15**: e692-698.

56 Caricato M, Ausania F, Ripetti V, Bartolozzi F, Campoli G, Coppola R. Retrospective analysis of long-term defunctioning stoma complications after colorectal surgery. *Colorectal Dis* 2007; **9**: 559–61.

57 Erwin-Toth P, Thompson SJ, Davis JS. Factors impacting the quality of life of people with an ostomy in North America: results from the Dialogue Study. *J Wound Ostomy Continence Nurs* 2012; **39**: 417–22; quiz 423–4.

58 Catalona WJ, Carvalhal GF, Mager DE, Smith DS. Potency, continence and complication rates in 1,870 consecutive radical retropubic prostatectomies. *J Urol* 1999; **162**: 433–8.

59 Donnellan SM, Duncan HJ, MacGregor RJ, Russell JM. Prospective assessment of incontinence after radical retropubic prostatectomy: objective and subjective analysis. *Urology* 1997; **49**: 225–30.

60 Eastham JA, Kattan MW, Rogers E, *et al.* Risk factors for urinary incontinence after radical prostatectomy. *J Urol* 1996; **156**: 1707–13.

61 Kundu SD, Roehl KA, Eggener SE, Antenor JAV, Han M, Catalona WJ. Potency, continence and complications in 3,477 consecutive radical retropubic prostatectomies. *J Urol* 2004; **172**: 2227–31.

62 Potosky AL, Davis WW, Hoffman RM, *et al.* Five-year outcomes after prostatectomy or radiotherapy for prostate cancer: the prostate cancer outcomes study. *J Natl Cancer Inst* 2004; **96**: 1358–67.

63 Sacco E, Prayer-Galetti T, Pinto F, *et al.* Urinary incontinence after radical prostatectomy: incidence by definition, risk factors and temporal trend in a large series with a long-term follow-up. *BJU Int* 2006; **97**: 1234–41.

64 Stanford JL, Feng Z, Hamilton AS, *et al.* Urinary and sexual function after radical prostatectomy for clinically localized prostate cancer: the Prostate Cancer Outcomes Study. *JAMA* 2000; **283**: 354–60.

65 Walsh PC, Marschke P, Ricker D, Burnett AL. Patient-reported urinary continence and sexual function after anatomic radical prostatectomy. *Urology* 2000; **55**: 58–61.

66 World Health Organization. SDG Target 3.4. Noncommunicable diseases and mental health: By 2030, reduce by one third premature mortality from non-communicable diseases through prevention and treatment and promote mental health and well-being. https://www.who.int/data/gho/data/themes/topics/indicator-groups/indicator-group-details/GHO/sdg-target-3.4-noncommunicable-diseases-and-mental-health (accessed Oct 11, 2024).

67 Ferlay J, Ervik M, Lam F, *et al.* Global Cancer Observatory: Cancer Today. Lyon, France: International Agency for Research on Cancer. 2020. https://gco.iarc.fr/today (accessed March 10, 2021).

68 Ferlay J, Colombet M, Soerjomataram I, *et al.* Cancer statistics for the year 2020: An overview. *International Journal of Cancer* 2021; **149**: 778–89.

69 Bray F, Laversanne M, Sung H, *et al.* Global cancer statistics 2022: GLOBOCAN estimates of incidence and mortality worldwide for 36 cancers in 185 countries. *CA: A Cancer Journal for Clinicians* DOI:10.3322/caac.21834.

70 Simpson EH. The Interpretation of Interaction in Contingency Tables. *Journal of the Royal Statistical Society Series B (Methodological)* 1951; **13**: 238–41.

71 Truven Health Analytics. United States MarketScan Commercial Claims and Encounters Database. Ann Arbor, United States.

72 Karagas MR, Greenberg ER, Spencer SK, Stukel TA, Mott LA. Increase in incidence rates of basal cell and squamous cell skin cancer in New Hampshire, USA. New Hampshire Skin Cancer Study Group. *Int J Cancer* 1999; **81**: 555–9.

73 Medical Expenditure Panel Survey Home. https://meps.ahrq.gov/mepsweb/ (accessed Nov 15, 2019).

74 Ferlay J, Colombet M, Soerjomataram I, *et al.* Estimating the global cancer incidence and mortality in 2018: GLOBOCAN sources and methods. *International Journal of Cancer* 2019; **144**: 1941–53.

75 Cogle CR, Craig BM, Rollison DE, List AF. Incidence of the myelodysplastic syndromes using a novel claims-based algorithm: high number of uncaptured cases by cancer registries. *Blood* 2011; **117**: 7121–5.

76 National Health Fund (Poland). Poland National Health Fund Patient Claims 2016.

77 The World Bank. World Bank Country and Lending Groups. https://datahelpdesk.worldbank.org/knowledgebase/articles/906519-world-bank-country-and-lending-groups (accessed Oct 11, 2024).
