# Supplementary material for: The global, regional, and national burden of cancer, 1990–2023, with forecasts to 2050: a systematic analysis for the Global Burden of Disease Study 2023
Source: Lancet. Author manuscript; Available in PMC 2025 Dec 9. (PMC12687902; doi:10.1016/S0140-6736(25)01635-6)
Supplement: Results appendix [file NIHMS2113905-supplement-Results_appendix.pdf]

Appendix 2: supplementary results appendix to "The global, regional, and national burden of cancer from 1990 to 2023 with forecasts to 2050: a systematic analysis for the Global Burden of Disease Study 2023"

This appendix provides additional figures and tables containing more detailed results for "The global, regional, and national burden of cancer from 1990 to 2023 with forecasts to 2050: a systematic analysis for the Global Burden of Disease Study 2023"

Figures

Appendix Figure 1. Number of cancer registry data site-years included in GBD 2023 for all cancers, all ages and sexes combined, 1980 - 2023 2
Appendix Figure 2. Number of verbal autopsy data site-years included in GBD 2023 for all cancers, all ages and sexes combined, 1980 - 2023 3
Appendix Figure 3. Number of vital registration data site-years included in GBD 2023 for all cancers, all ages and sexes combined, 1980 – 2023 4
Appendix Figure 4. Global map of most common incident cancer types in males for all ages in 2023 5
Appendix Figure 5. Global map of most common incident cancer types in females for all ages in 2023 6
Appendix Figure 6. Global map of most common cancer types causing mortality in males for all ages in 2023 7
Appendix Figure 7. Global map of most common cancer types causing mortality in females for all ages in 2023 8
Appendix Figure 8. Absolute risk-attributable deaths (top row) and proportion of deaths by cause (bottom row) per risk factor for level 3 cancer causes for all ages and all sexes combined, males, and females 9

Tables

Appendix Table 1. GBD 2023 cancer risk-outcome pairs 10
Appendix Table 2. Cancer incident case, death, and DALY counts and age-standardised rates in 2023 and percent change in counts and rates between 1990 and 2023 by World Bank income group for males of all ages 13
Appendix Table 3. Cancer incident case, death, and DALY counts and age-standardised rates in 2023 and percent change in counts and rates between 1990 and 2023 by World Bank income group for females of all ages 14
Appendix Table 4. Male-to-female ratio of global cancer incident cases and deaths for all ages in 2023 15
Appendix Table 5. Percent of cancer incident cases, deaths, and DALYs by age group for all sexes combined in 2023 17
Appendix Table 6. Proportion of cancer deaths attributable to risk factors by World Bank income group for all ages and for all sexes combined, males, and females in 2023 18
Appendix Table 7. Percent of total cancer deaths attributable to the top three leading level 2 risk factors for deaths by World Bank income group in 2023 for all ages and sexes combined 19
Appendix Table 8. Cancer incident cases and age-standardised incidence rate in 2050 and percent change in cases and rates between 2024 and 2050 by World Bank income group for all ages and sexes combined 20
Appendix Table 9. Cancer deaths and age-standardised death rate in 2050 and percent change in deaths and rates between 2024 and 2050 by World Bank income group for all ages and sexes combined 21
Appendix Table 10. Cancer deaths and age-standardised death rate 2050 and percent change in deaths and rates between 2024 and 2050 by World Bank income group for three leading cancer types for global deaths and cancer types highlighted in active WHO initiatives for all ages and sexes combined 22
Appendix Table 11. Composition of cancer DALYs in 2023 by contribution of YLDs and YLL, globally and by World Bank income groups for all sexes combined 24

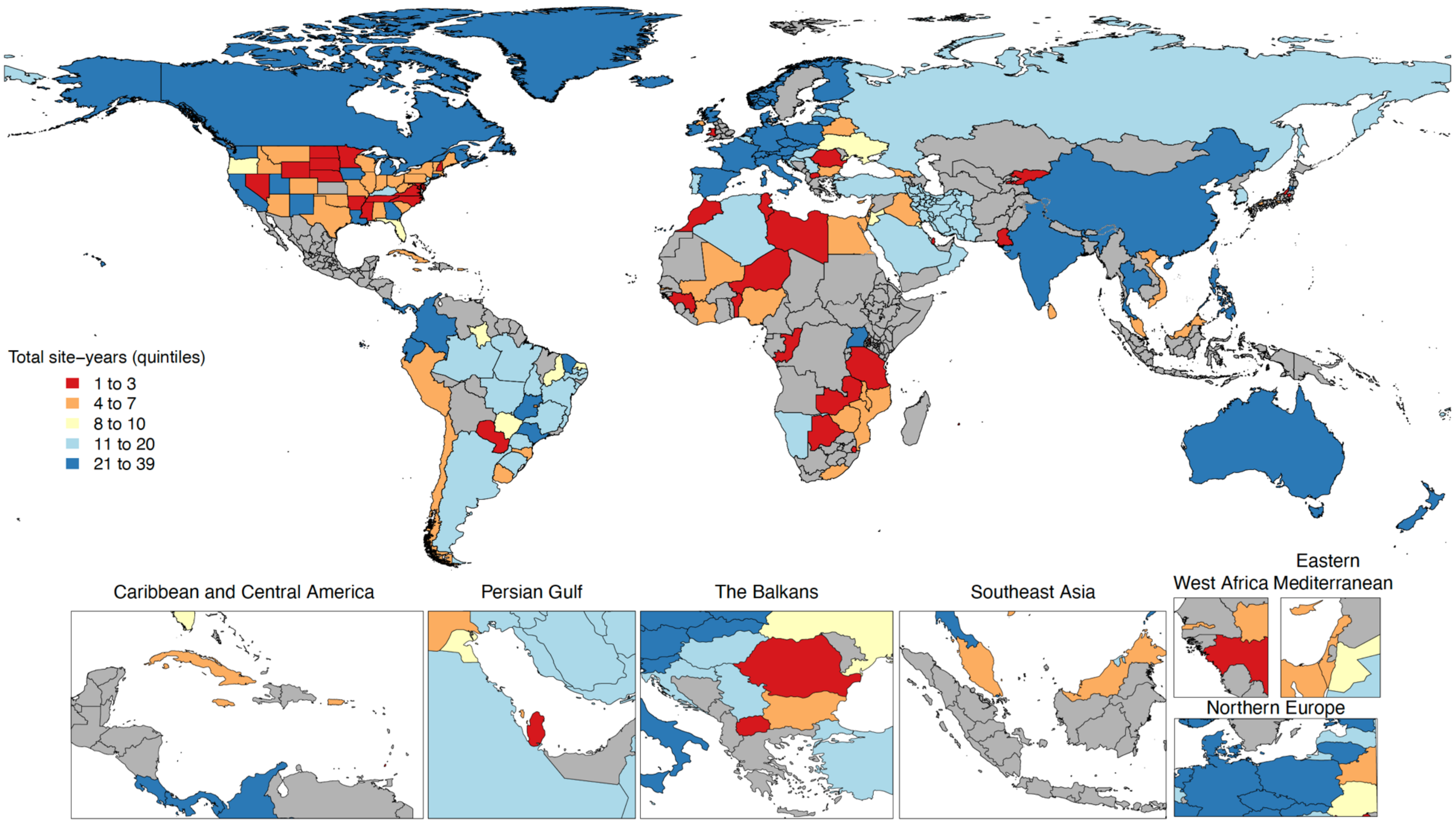

Appendix Figure 1. Number of cancer registry data site-years included in GBD 2023 for all cancers, all ages and sexes combined, 1980 - 2023

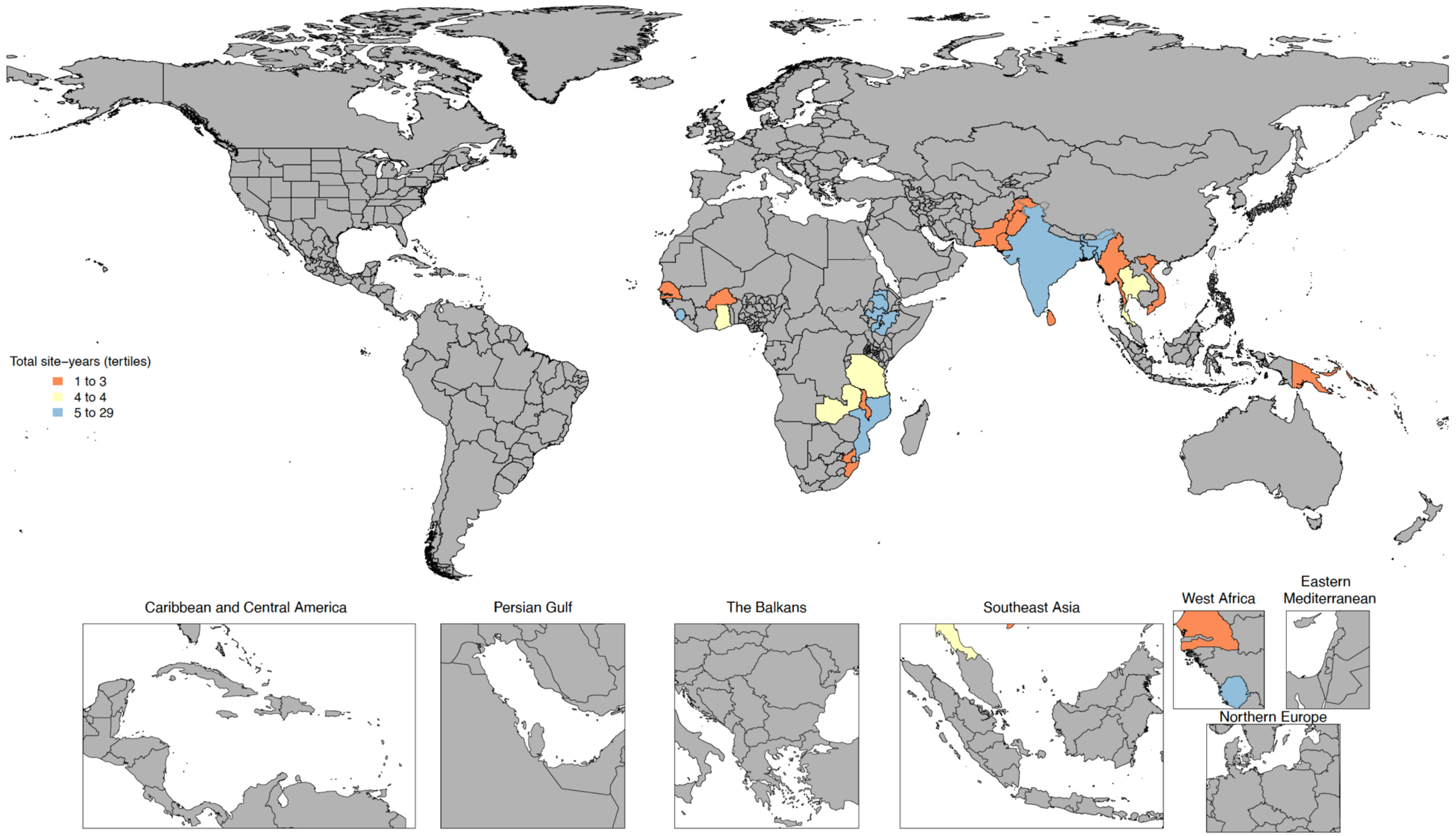

Appendix Figure 2. Number of verbal autopsy data site-years included in GBD 2023 for all cancers, all ages and sexes combined, 1980 - 2023

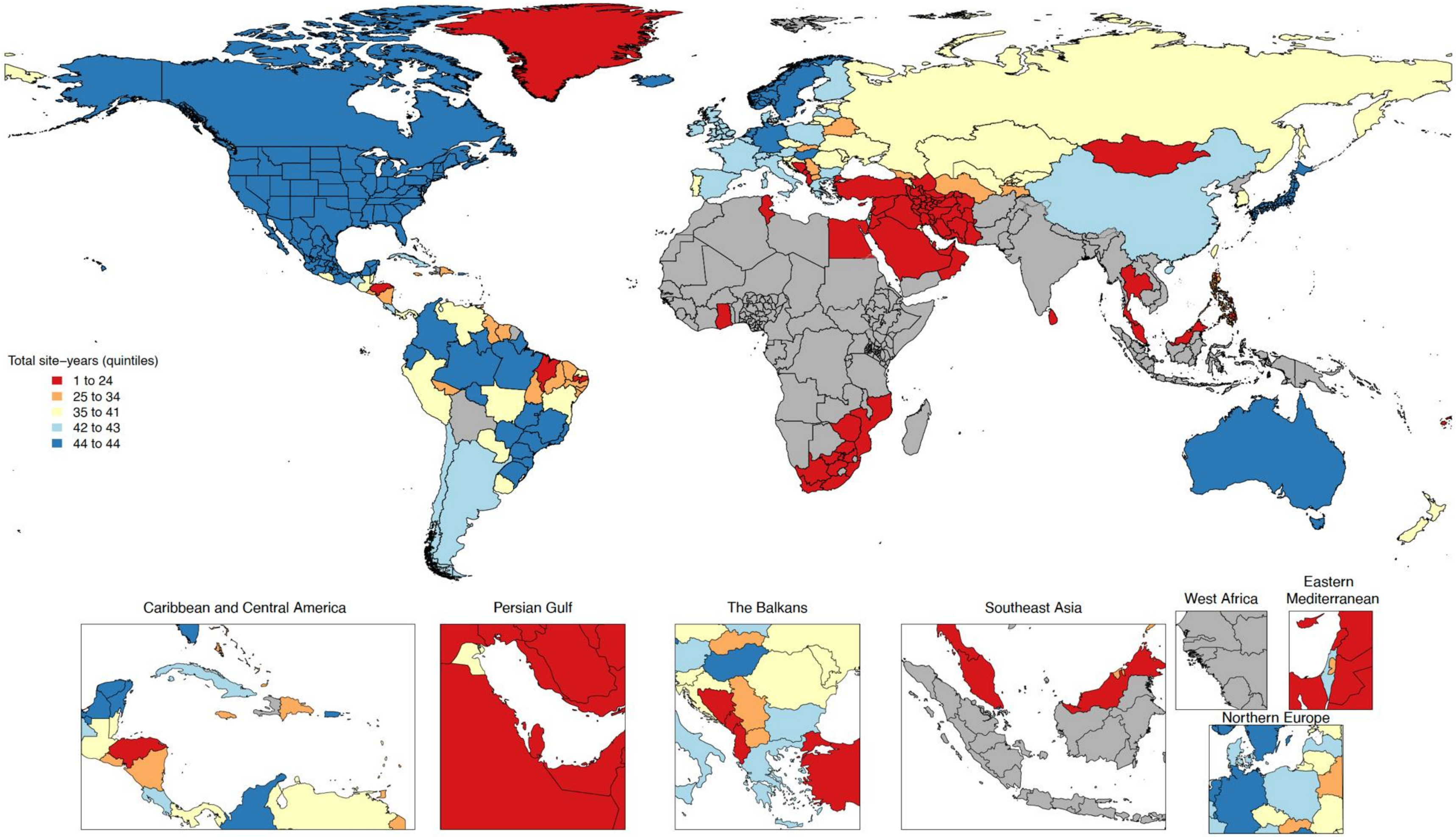

Appendix Figure 3. Number of vital registration data site-years included in GBD 2023 for all cancers, all ages and sexes combined, 1980 – 2023

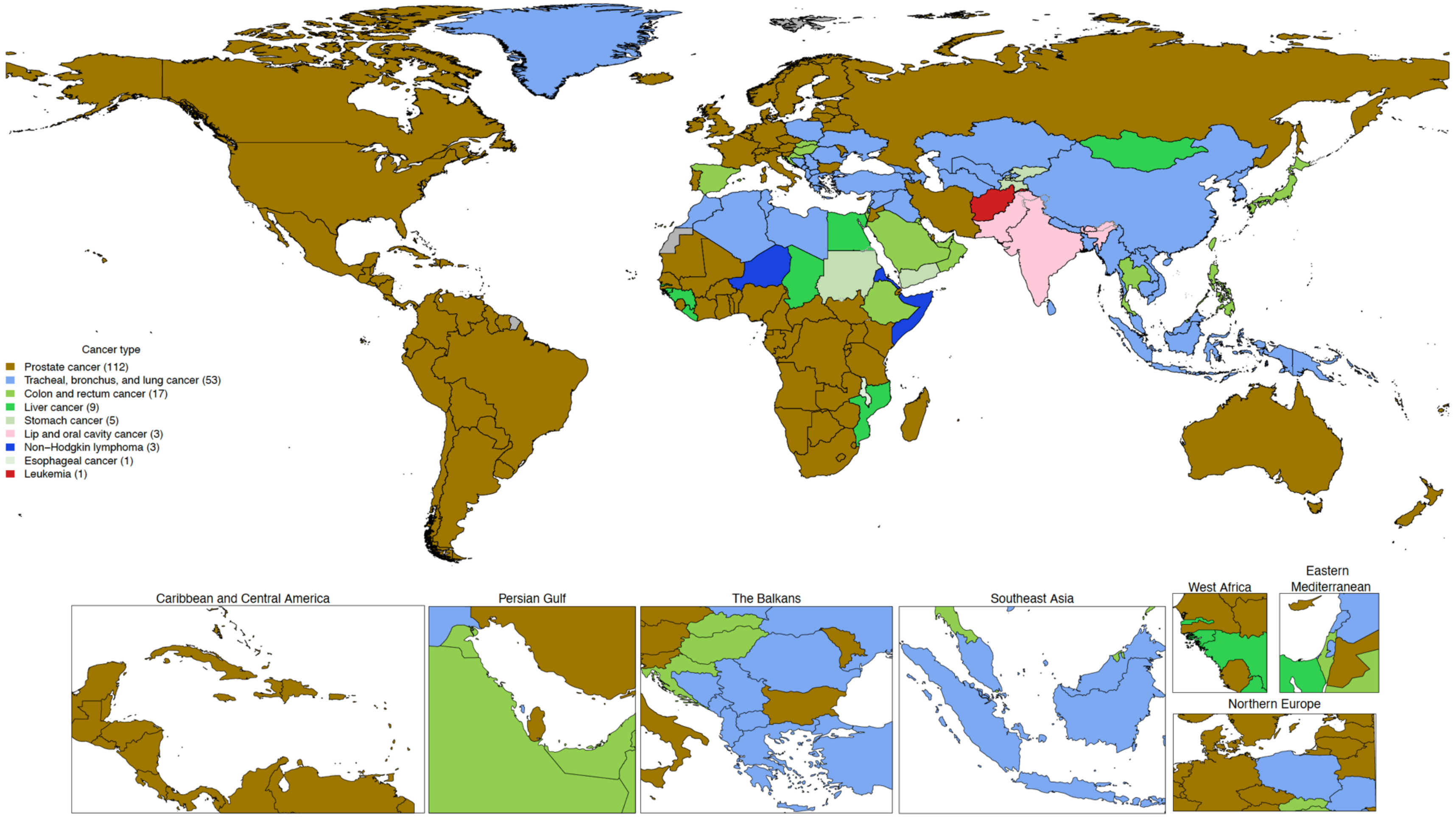

Appendix Figure 4. Global map of most common incident cancer types in males for all ages in 2023

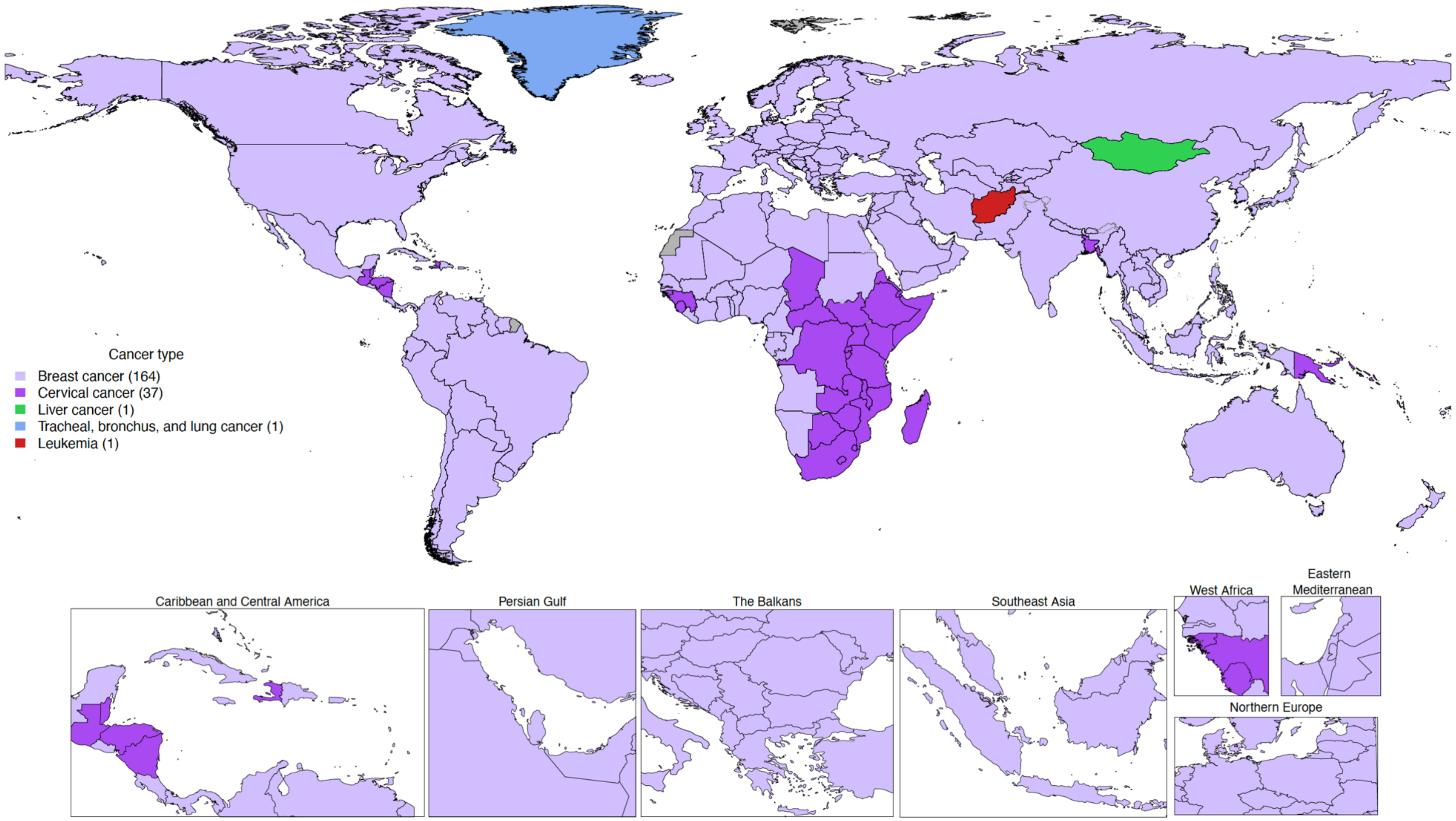

Appendix Figure 5. Global map of most common incident cancer types in females for all ages in 2023

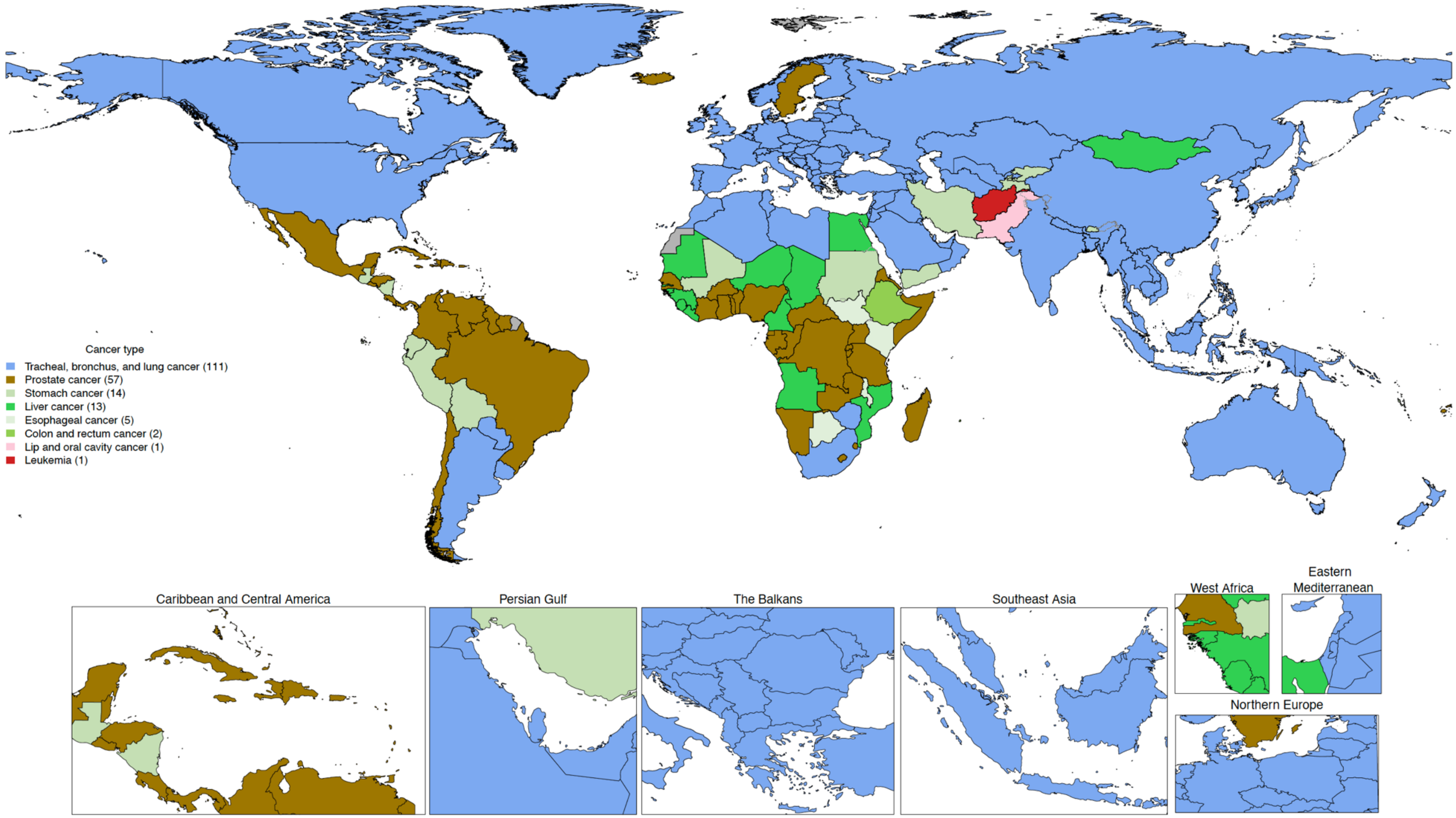

Appendix Figure 6. Global map of most common cancer types causing mortality in males for all ages in 2023

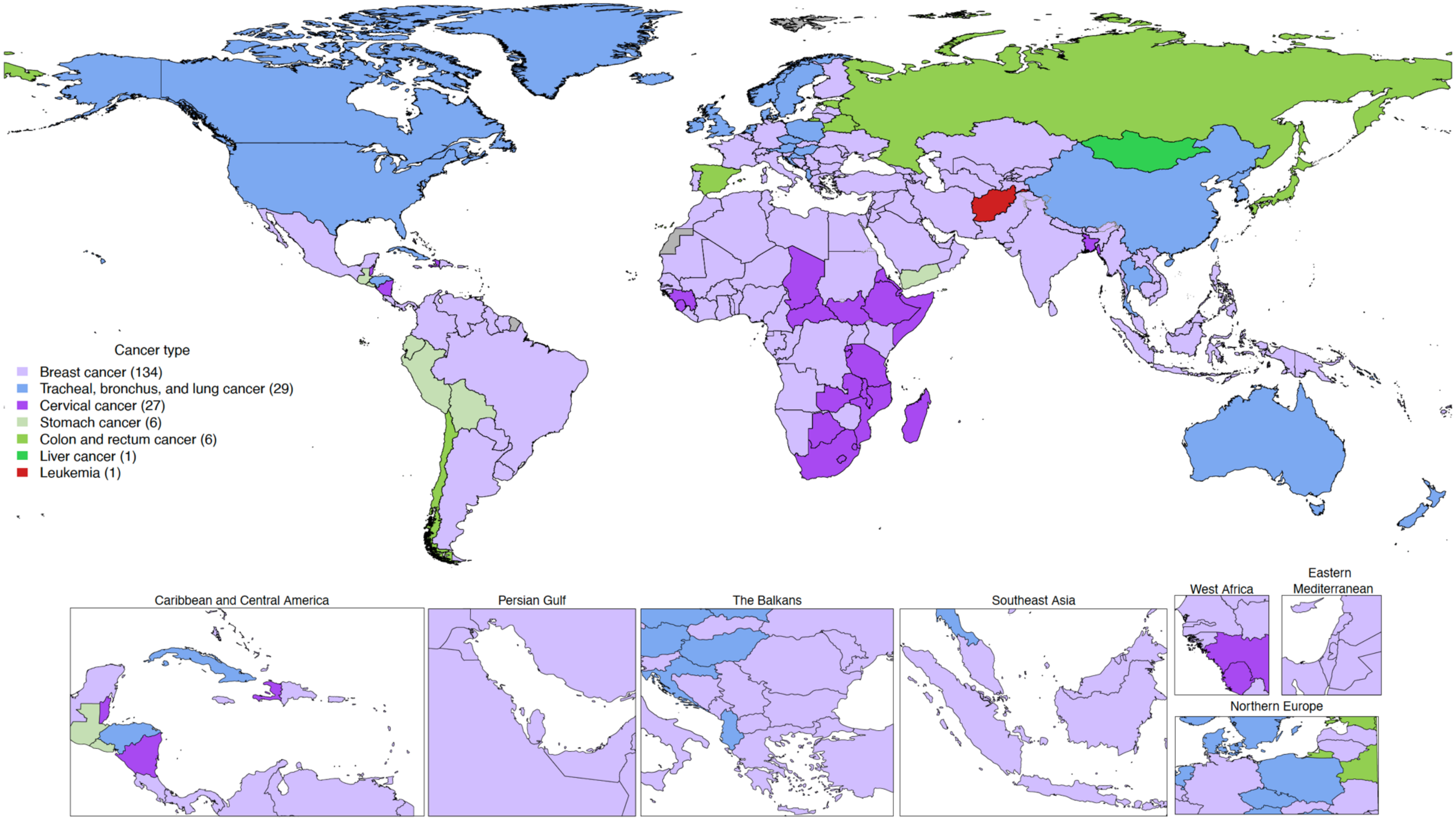

Appendix Figure 7. Global map of most common cancer types causing mortality in females for all ages in 2023

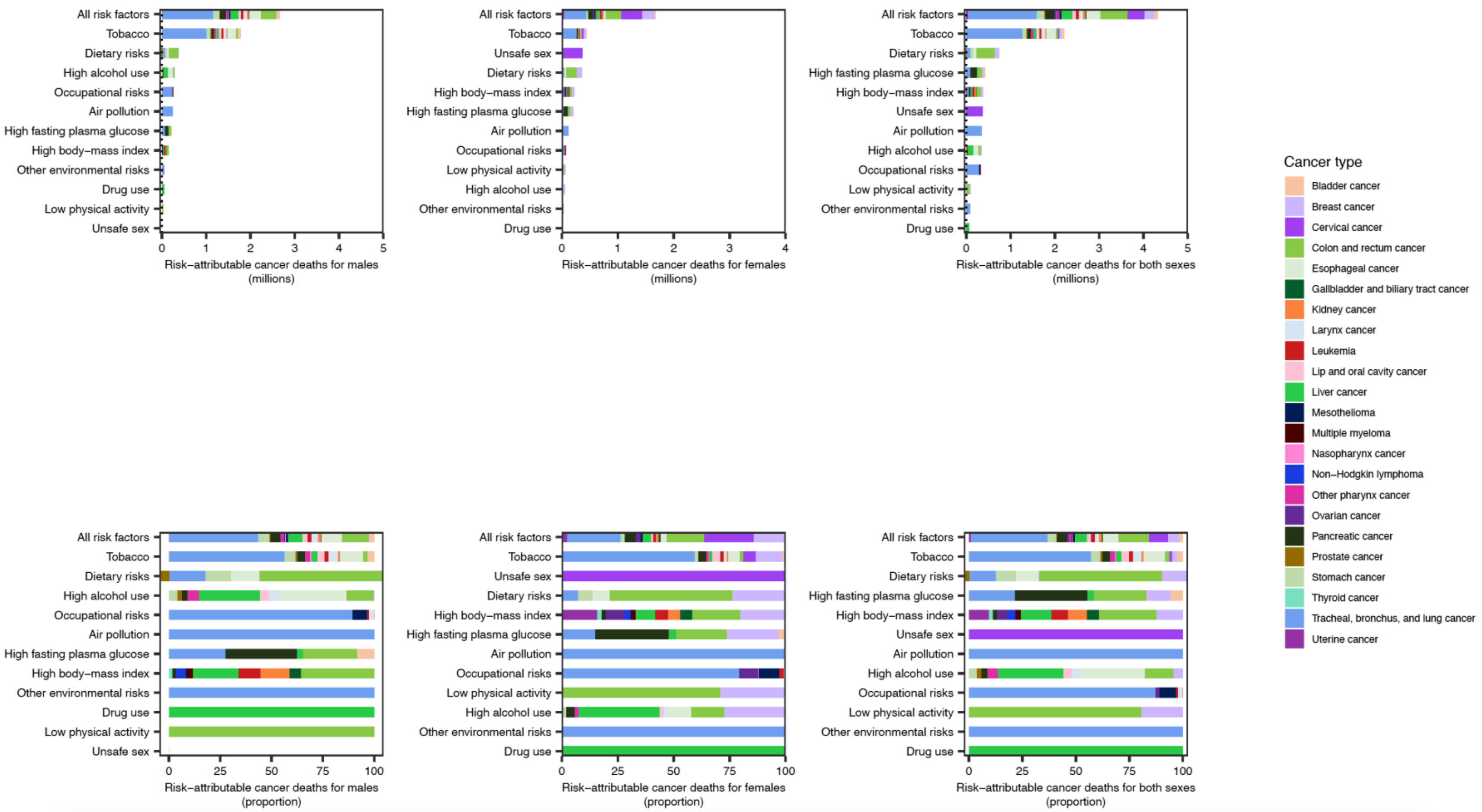

Appendix Figure 8. Absolute risk-attributable deaths (top row) and proportion of deaths by cause (bottom row) per risk factor for level 3 cancer causes for all ages and all sexes combined, males, and females

Appendix Table 1. GBD 2023 cancer risk-outcome pairs

Level 1 risk factors	Level 2 risk factors	Level 3 risk factors	Level 4 risk factors	Cancer outcomes
Environmental / Occupational	Air pollution	Particulate matter pollution	Ambient particulate matter pollution	Tracheal, bronchus, and lung cancer
			Household air pollution from solid fuels	Tracheal, bronchus, and lung cancer
	Other environmental risks	Residential radon	NA	Tracheal, bronchus, and lung cancer
	Occupational risks	Occupational carcinogens	Occupational exposure to arsenic	Tracheal, bronchus, and lung cancer
			Occupational exposure to asbestos	Larynx cancer
				Mesothelioma
				Ovarian cancer (F)
				Tracheal, bronchus, and lung cancer
			Occupational exposure to benzene	Acute lymphoid leukemia
				Acute myeloid leukemia
				Chronic lymphoid leukemia
				Chronic myeloid leukemia
				Leukemia
				Other leukemia
			Occupational exposure to beryllium	Tracheal, bronchus, and lung cancer
			Occupational exposure to cadmium	Tracheal, bronchus, and lung cancer
			Occupational exposure to chromium	Tracheal, bronchus, and lung cancer
			Occupational exposure to diesel engine exhaust	Tracheal, bronchus, and lung cancer
			Occupational exposure to formaldehyde	Acute lymphoid leukemia
				Acute myeloid leukemia
				Chronic lymphoid leukemia
				Chronic myeloid leukemia
				Leukemia
				Nasopharynx cancer
				Other leukemia
			Occupational exposure to nickel	Tracheal, bronchus, and lung cancer
			Occupational exposure to polycyclic aromatic hydrocarbons	Tracheal, bronchus, and lung cancer
			Occupational exposure to silica	Tracheal, bronchus, and lung cancer
			Occupational exposure to sulfuric acid	Larynx cancer
			Occupational exposure to trichloroethylene	Kidney cancer
Behavioural	Tobacco	Smoking	NA	Acute lymphoid leukemia
				Acute myeloid leukemia
				Bladder cancer
				Breast cancer
				Cervical cancer (F)
				Chronic lymphoid leukemia
				Chronic myeloid leukemia
				Colon and rectum cancer
				Oesophageal cancer
				Kidney cancer
				Larynx cancer
				Leukemia
				Lip and oral cavity cancer
				Liver cancer
				Liver cancer due to alcohol use
				Liver cancer due to hepatitis B

				Liver cancer due to hepatitis C
				Liver cancer due to NASH
				Liver cancer due to other causes
				Nasopharynx cancer
				Other leukemia
				Other pharynx cancer
				Pancreatic cancer
				Prostate cancer (M)
				Stomach cancer
				Tracheal, bronchus, and lung cancer
		Secondhand smoke	NA	Breast cancer
				Tracheal, bronchus, and lung cancer
		Chewing tobacco	NA	Oesophageal cancer
				Larynx cancer
				Lip and oral cavity cancer
				Nasopharynx cancer
				Other pharynx cancer
	High alcohol use	NA	NA	Breast cancer
				Colon and rectum cancer
				Oesophageal cancer
				Larynx cancer
				Lip and oral cavity cancer
				Liver cancer
				Liver cancer due to alcohol use
				Liver cancer due to hepatitis B
				Liver cancer due to hepatitis C
				Liver cancer due to NASH
				Liver cancer due to other causes
				Other pharynx cancer
				Pancreatic cancer
				Prostate cancer (M)
				Stomach cancer
	Drug use	NA	NA	Liver cancer
				Liver cancer due to hepatitis B
				Liver cancer due to hepatitis C
	Dietary risks	Diet high in processed meat	NA	Colon and rectum cancer
		Diet high in red meat	NA	Breast cancer
				Colon and rectum cancer
		Diet high in sodium	NA	Stomach cancer
		Diet low in calcium	NA	Colon and rectum cancer
				Prostate cancer (M)
		Diet low in fiber	NA	Colon and rectum cancer
		Diet low in fruits	NA	Tracheal, bronchus, and lung cancer
		Diet low in milk	NA	Colon and rectum cancer
				Prostate cancer (M)
		Diet low in vegetables	NA	Oesophageal cancer
		Diet low in whole grains	NA	Colon and rectum cancer
	Unsafe sex	NA	NA	Cervical cancer (F)
	Low physical activity	NA	NA	Breast cancer
				Colon and rectum cancer

Metabolic	High body-mass index	NA	NA	Acute lymphoid leukemia
				Acute myeloid leukemia
				Breast cancer
				Burkitt lymphoma
				Chronic lymphoid leukemia
				Chronic myeloid leukemia
				Colon and rectum cancer
				Gallbladder and biliary tract cancer
				Kidney cancer
				Leukemia
				Liver cancer
				Liver cancer due to alcohol use
				Liver cancer due to hepatitis B
				Liver cancer due to hepatitis C
				Liver cancer due to NASH
				Liver cancer due to other causes
				Multiple myeloma
				Non-Hodgkin lymphoma
				Other leukemia
				Other non-Hodgkin lymphoma
				Ovarian cancer (F)
				Pancreatic cancer
				Thyroid cancer
				Uterine cancer (F)
	High fasting plasma glucose	NA	NA	Bladder cancer
				Breast cancer
				Colon and rectum cancer
				Liver cancer
				Liver cancer due to NASH
				Liver cancer due to other causes
				Pancreatic cancer
				Tracheal, bronchus, and lung cancer

Abbreviations: (F), female-only cancer outcome; (M), male-only cancer outcome; NA, no underlying more granular risk factor

Appendix Table 2. Cancer incident case, death, and DALY counts and age-standardised rates in 2023 and percent change in counts and rates between 1990 and 2023 by World Bank income group for males of all ages

	**Global**	**World Bank income group**			
	-	High	Upper middle	Lower middle	Low
Incident cases 2023 in thousands (UI)	9560 (8510 to 10700)	4130 (3640 to 4620)	3800 (3360 to 4380)	1370 (1200 to 1530)	247 (209 to 283)
Incident cases, percent change 1990 to 2023 (UI)	104.2 (77.8 to 139.9)	70.7 (46.7 to 99.7)	125.4 (88.7 to 179.1)	193.7 (142.9 to 259.2)	139.3 (98.9 to 191.5)
Age-standardised incidence rate 2023, per 100 000 (UI)	225.6 (201.2 to 251.5)	347.0 (304.2 to 391.1)	212.0 (186.2 to 244.8)	115.9 (101.3 to 130.1)	138.1 (114.4 to 159.5)
Age-standardised incidence rate, percent change 1990 to 2023 (UI)	-11.2 (-22.4 to 4.2)	-10.3 (-23.5 to 6.0)	-8.1 (-23.2 to 13.5)	22.1 (0.6 to 48.9)	6.6 (-11.9 to 29.6)
Deaths 2023 in thousands (UI)	5770 (5370 to 6120)	1950 (1820 to 2020)	2500 (2300 to 2730)	1110 (966 to 1250)	199 (166 to 227)
Deaths, percent change 1990 to 2023 (UI)	73.2 (54.9 to 88.8)	36.1 (30.2 to 39.9)	76.7 (44.3 to 104.3)	181.6 (133.5 to 244.0)	140.8 (99.2 to 200.5)
Age-standardised mortality rate 2023, per 100 000 (UI)	138.8 (128.9 to 147.4)	157.6 (147.7 to 163.2)	141.4 (130.0 to 154.7)	99.0 (85.7 to 112.6)	120.5 (99.0 to 139.5)
Age-standardised mortality rate, percent change 1990 to 2023 (UI)	-26.6 (-34.1 to -20.6)	-33.0 (-35.4 to -31.4)	-31.0 (-43.5 to -20.0)	15.0 (-6.0 to 43.1)	5.6 (-13.5 to 33.7)
DALYs 2023 in millions (UI)	147000 (138000 to 158000)	41300 (39300 to 42500)	64700 (59700 to 70800)	33900 (29800 to 38200)	7050 (6030 to 8060)
DALYs, percent change 1990 to 2023 (UI)	49.9 (34.8 to 64.7)	12.4 (8.9 to 15.1)	46.3 (21.7 to 68.6)	146.3 (107.4 to 198.2)	114.7 (77.9 to 169.2)
Age-standardised DALY rate 2023, per 100 000 (UI)	3417.3 (3194.1 to 3657.8)	3554.5 (3399.7 to 3657.4)	3574.8 (3307.6 to 3882.4)	2680.9 (2346.2 to 3043.8)	3318.5 (2791.8 to 3783.0)
Age-standardised DALY rate, percent change 1990 to 2023 (UI)	-29.6 (-36.6 to -22.8)	-38.4 (-40.0 to -36.9)	-35.1 (-46.3 to -25.5)	10.7 (-7.6 to 34.1)	-2.0 (-18.7 to 22.2)

Abbreviations: DALY, disability-adjusted life years; UI, 95% uncertainty interval

Appendix Table 3. Cancer incident case, death, and DALY counts and age-standardised rates in 2023 and percent change in counts and rates between 1990 and 2023 by World Bank income group for females of all ages

	Global	World Bank income group			
	-	High	Upper middle	Lower middle	Low
Incident cases 2023 in thousands (UI)	8990 (7850 to 10200)	3650 (3090 to 4130)	3060 (2640 to 3520)	1820 (1,570 to 2,130)	451 (378 to 546)
Incident cases, percent change 1990 to 2023 (UI)	106.1 (74.7 to 141.2)	64.4 (40.1 to 93.3)	108.0 (65.1 to 150.3)	242.1 (168.7 to 321.3)	236.9 (157.5 to 328.7)
Age-standardised incidence rate 2023, per 100 000 (UI)	190.9 (166.2 to 217.1)	273.0 (234.5 to 310.5)	158.8 (137.3 to 184.7)	136.7 (118.8 to 158.6)	197.0 (166.0 to 235.2)
Age-standardised incidence rate, percent change 1990 to 2023 (UI)	-3.5 (-17.6 to 13.0)	1.2 (-14.3 to 20.5)	-10.2 (-28.8 to 7.4)	34.5 (6.1 to 63.6)	39.9 (7.8 to 74.3)
Deaths 2023 in thousands (UI)	4610 (4170 to 5000)	1580 (1380 to 1700)	1630 (1450 to 1830)	1130 (1010 to 1280)	256 (221 to 295)
Deaths, percent change 1990 to 2023 (UI)	75.8 (57.7 to 90.4)	39.6 (30.0 to 46.0)	57.6 (30.2 to 81.2)	210.6 (156.0 to 278.4)	200.3 (146.5 to 260.7)
Age-standardised mortality rate 2023, per 100 000 (UI)	95.5 (86.8 to 103.4)	99.5 (89.4 to 105.2)	81.9 (73.4 to 92.1)	90.8 (80.2 to 102.7)	124.4 (106.5 to 143.6)
Age-standardised mortality rate, percent change 1990 to 2023 (UI)	-21.7 (-29.9 to -15.5)	-22.9 (-26.3 to -20.4)	-37.4 (-48.4 to -28.2)	19.2 (-2.3 to 45.0)	24.6 (0.7 to 49.6)
DALYs 2023 in millions (UI)	124,000 (115000 to 134000)	32700 (29900 to 34500)	43,100 (38900 to 47600)	38000 (33900 to 43000)	10400 (9030 to 12000)
DALYs, percent change 1990 to 2023 (UI)	57.7 (41.9 to 73.4)	17.8 (11.9 to 21.9)	29.7 (10.8 to 47.2)	172.0 (126.4 to 232.5)	176.7 (128.2 to 233.1)
Age-standardised DALY rate 2023, per 100 000 (UI)	2701.2 (2505.6 to 2910.7)	2506.5 (2334.8 to 2611.3)	2276.1 (2072.3 to 2510.7)	2794.5 (2497.6 to 3164.5)	4284.4 (3711.4 to 4928.0)
Age-standardised DALY rate, percent change 1990 to 2023 (UI)	-22.1 (-30.1 to -14.8)	-27.7 (-30.2 to -25.7)	-40.4 (-49.5 to -32.2)	15.0 (-4.6 to 40.5)	21.7 (-0.5 to 46.1)

Abbreviations: DALY, disability-adjusted life years; UI, 95% uncertainty interval

Appendix Table 4. Male-to-female ratio of global cancer incident cases and deaths for all ages in 2023

Cancer type	Incidence cases, male-to-female ratio (UI)	Deaths, male-to-female ratio (UI)
**Total cancers excluding non-melanoma skin cancer**	1.07 (1.00 to 1.14)	1.25 (1.14 to 1.36)
Lip and oral cavity cancer	1.72 (1.32 to 2.22)	1.82 (1.34 to 2.43)
Nasopharynx cancer	2.17 (1.65 to 2.96)	2.28 (1.62 to 3.05)
Other pharynx cancer	4.34 (3.01 to 5.67)	4.42 (2.83 to 6.15)
Oesophageal cancer	2.46 (1.95 to 3.13)	2.38 (1.85 to 3.10)
Stomach cancer	2.05 (1.56 to 2.63)	1.88 (1.39 to 2.37)
Colon and rectum cancer	1.36 (1.21 to 1.51)	1.26 (1.08 to 1.44)
Liver cancer	2.34 (1.84 to 2.90)	2.22 (1.72 to 2.74)
Liver cancer due to hepatitis B	4.67 (3.56 to 6.03)	4.58 (3.43 to 5.83)
Liver cancer due to hepatitis C	1.23 (0.99 to 1.49)	1.15 (0.91 to 1.42)
Liver cancer due to alcohol use	4.53 (3.65 to 5.51)	4.29 (3.41 to 5.32)
Liver cancer due to NASH	1.15 (0.89 to 1.45)	1.12 (0.84 to 1.43)
Hepatoblastoma	1.82 (1.01 to 2.80)	2.08 (0.88 to 3.81)
Liver cancer due to other causes	0.87 (0.69 to 1.12)	0.86 (0.67 to 1.09)
Gallbladder and biliary tract cancer	0.90 (0.73 to 1.19)	0.82 (0.63 to 1.12)
Pancreatic cancer	1.15 (1.04 to 1.26)	1.16 (1.04 to 1.30)
Larynx cancer	6.18 (4.54 to 8.07)	6.20 (4.33 to 8.46)
Tracheal, bronchus, and lung cancer	1.96 (1.67 to 2.20)	2.02 (1.69 to 2.28)
Malignant skin melanoma	1.09 (0.95 to 1.22)	1.28 (0.98 to 1.57)
Soft tissue and other extraosseous sarcomas	1.38 (0.98 to 1.89)	1.32 (0.85 to 2.01)
Malignant neoplasm of bone and articular cartilage	1.27 (0.81 to 1.91)	1.38 (0.87 to 2.13)
Breast cancer	0.02 (0.01 to 0.03)	0.02 (0.01 to 0.03)
Cervical cancer	NA	NA
Uterine cancer	NA	NA
Ovarian cancer	NA	NA
Prostate cancer	NA	NA
Testicular cancer	NA	NA
Kidney cancer	1.81 (1.52 to 2.11)	1.89 (1.57 to 2.22)
Bladder cancer	3.15 (2.68 to 3.62)	2.85 (2.42 to 3.35)
Brain and central nervous system cancer	1.16 (0.92 to 1.48)	1.27 (1.01 to 1.65)
Eye cancer	0.98 (0.66 to 1.46)	1.01 (0.65 to 1.71)
Retinoblastoma	1.19 (0.43 to 2.86)	1.25 (0.27 to 3.99)
Other eye cancers	0.96 (0.64 to 1.44)	0.98 (0.66 to 1.43)
Neuroblastoma and other peripheral nervous cell tumors	1.19 (0.92 to 1.54)	1.28 (0.89 to 1.77)
Thyroid cancer	0.42 (0.32 to 0.54)	0.63 (0.46 to 0.85)
Mesothelioma	2.80 (2.28 to 3.49)	2.88 (2.30 to 3.57)
Hodgkin lymphoma	1.39 (0.96 to 1.94)	1.55 (0.93 to 2.37)
Non-Hodgkin lymphoma	1.30 (1.07 to 1.56)	1.38 (1.09 to 1.70)
Burkitt lymphoma	1.64 (0.91 to 2.84)	1.68 (0.85 to 2.99)
Other non-Hodgkin lymphoma	1.29 (1.07 to 1.55)	1.38 (1.09 to 1.69)
Multiple myeloma	1.19 (0.98 to 1.41)	1.13 (0.93 to 1.35)
Leukemia	1.32 (1.12 to 1.59)	1.34 (1.10 to 1.63)

Acute lymphoid leukemia	1.35 (0.98 to 1.90)	1.40 (0.99 to 2.01)
Chronic lymphoid leukemia	1.50 (1.22 to 1.90)	1.49 (1.21 to 1.86)
Acute myeloid leukemia	1.18 (0.95 to 1.48)	1.22 (0.95 to 1.57)
Chronic myeloid leukemia	1.30 (0.94 to 1.77)	1.36 (0.85 to 2.10)
Other leukemia	1.45 (0.97 to 2.32)	1.51 (1.01 to 2.46)
Other malignant neoplasms	0.91 (0.76 to 1.07)	1.00 (0.77 to 1.26)

Abbreviations: UI, 95% uncertainty interval; NASH, non-alcoholic steatohepatitis; NA, not applicable

Appendix Table 5. Percent of cancer incident cases, deaths, and DALYs by age group for all sexes combined in 2023

Age	Incident cases, % (UI)	Deaths, % (UI)	DALYs, % (UI)
0 - 14	1.4 (1.2 to 1.8)	1.0 (0.9 to 1.1)	3.2 (2.9 to 3.6)
0 - 39	9.1 (8.2 to 10.1)	5.6 (5.2 to 6.1)	13.9 (13.1 to 14.8)
15 - 39	7.7 (7.0 to 8.5)	4.6 (4.3 to 5.1)	10.6 (10.0 to 11.4)
40 - 64	38.2 (37.1 to 39.9)	32.7 (31.2 to 34.5)	45.2 (43.9 to 46.9)
65+	52.7 (50.5 to 54.3)	61.7 (59.5 to 63.4)	40.9 (39.1 to 42.5)

Abbreviations: DALY, disability-adjusted life years; UI, 95% uncertainty interval.

Appendix Table 6. Proportion of cancer deaths attributable to risk factors by World Bank income group for all ages and for all sexes combined, males, and females in 2023

	All sexes combined, % (UI)	Male, % (UI)	Female, % (UI)
**Global**	**41.7% (37.8 to 45.4)**	**46.0% (41.9 to 50.3)**	**36.3% (31.7 to 40.8)**
**World Bank income group**			
High income	42.3% (38.1 to 46.1)	46.0% (42.0 to 49.7)	37.8% (32.3 to 42.3)
Upper middle income	45.5% (41.0 to 50.1)	51.4% (46.6 to 56.8)	36.4% (31.7 to 40.9)
Lower middle income	36.1% (32.0 to 39.9)	38.1% (33.8 to 43.3)	34.2% (28.6 to 39.9)
Low income	31.3% (26.6 to 35.7)	24.5% (20.9 to 29.4)	36.6% (29.8 to 43.3)

Abbreviations: UI, 95% uncertainty interval.

Appendix Table 7. Percent of total cancer deaths attributable to the top three leading level 2 risk factors for deaths by World Bank income group in 2023 for all ages and sexes combined

	Leading risk factor	% of total cancer deaths (UI)	2nd leading risk factor	% of total cancer deaths (UI)	3rd leading risk factor	% of total cancer deaths (UI)
**Global**	**Tobacco**	**21.4 (18.8 to 24.3)**	**Dietary risks**	**7.0 (2.5 to 11.5)**	**High fasting plasma glucose**	**4.2 (3.2 to 5.4)**
**World Bank income group**						
High income	Tobacco	19.7 (16.9 to 22.8)	Dietary risks	7.8 (2.9 to 12.3)	High fasting plasma glucose	5.7 (4.5 to 7.2)
Upper middle income	Tobacco	27.3 (24.2 to 31.1)	Dietary risks	6.7 (2.3 to 11.9)	Air pollution	5.3 (3.5 to 7.3)
Lower middle income	Tobacco	16.1 (13.4 to 18.9)	Unsafe sex	7.0 (4.9 to 9.8)	Dietary risks	6.4 (1.9 to 10.6)
Low income	Unsafe sex	12.5 (8.4 to 17.0)	Tobacco	6.2 (4.6 to 8.5)	Dietary risks	6.0 (1.8 to 10.1)

Abbreviations: UI, 95% uncertainty interval

Appendix Table 8. Cancer incident cases and age-standardised incidence rate in 2050 and percent change in cases and rates between 2024 and 2050 by World Bank income group for all ages and sexes combined

	Incident cases 2050, in thousands (UI)	Incident cases, percent change 2024 to 2050 (UI)	Age-standardised incidence rate 2050, per 100 000 (UI)	Age-standardised incidence rate, percent change 2024 to 2050, per 100 000 (UI)
**Global**	**30500 (22900 to 38900)**	**60.7 (41.9 to 80.6)**	**192.9 (157.0 to 232.5)**	**-5.7 (-12.4 to 1.6)**
**World Bank income group**				
High income	10400 (8270 to 13200)	31.3 (21.6 to 40.6)	302.5 (251.1 to 393.3)	-0.2 (-5.5 to 5.3)
Upper middle income	11400 (9070 to 13800)	62.7 (38.9 to 88.3)	193.6 (159.3 to 228.6)	6.0 (-4.8 to 16.6)
Lower middle income	6740 (5220 to 8410)	104.8 (77.0 to 135.5)	132.8 (108.1 to 161.2)	5.3 (-3.8 to 16.2)
Low income	1890 (1620 to 2260)	161.4 (129.5 to 196.5)	173.1 (152.2 to 200.2)	3.7 (-3.7 to 13.6)

Abbreviations: UI, 95% uncertainty interval

Appendix Table 9. Cancer deaths and age-standardised death rate in 2050 and percent change in deaths and rates between 2024 and 2050 by World Bank income group for all ages and sexes combined

	Deaths 2050, in thousands (UI)	Deaths, percent change 2024 to 2050 (UI)	Age-standardised mortality rate 2050, per 100 000 (UI)	Age-standardised mortality rate, percent change 2024 to 2050, per 100 000 (UI)
**Global**	**18600 (15600 to 21500)**	**74.5 (50.1 to 104.2)**	**107.9 (96.7 to 119.4)**	**-5.6 (-12.8 to 4.6)**
**World Bank income group**				
High income	5160 (4420 to 5730)	42.8 (28.3 to 58.6)	119.1 (106.3 to 131.1)	-3.8 (-10.5 to 4.2)
Upper middle income	7190 (6290 to 8180)	70.0 (41.8 to 105.6)	104.9 (94.1 to 115.4)	-3.6 (-12.9 to 8.8)
Lower middle income	4950 (4030 to 6210)	114.4 (82.0 to 152.7)	95.7 (82.2 to 114.9)	1.6 (-6.5 to 13.0)
Low income	1220 (1070 to 1400)	160.2 (127.2 to 201.2)	120.8 (104.5 to 136.9)	-0.2 (-6.5 to 8.6)

Abbreviations: UI, 95% uncertainty interval

Appendix Table 10. Cancer deaths and age-standardised death rate in 2050 and percent change in deaths and rates between 2024 and 2050 by World Bank income group for three leading cancer types for global deaths and cancer types highlighted in active WHO initiatives for all ages and sexes combined

	Deaths 2050, in thousands (UI)	Deaths, percent change 2024 to 2050 (UI)	Age-standardised mortality rate 2050, per 100 000 (UI)	Age-standardised mortality rate, percent change 2024 to 2050, per 100 000 (UI)
**Leading cancer types for global deaths**				
**Stomach cancer**				
**Global**	**1160 (959 to 1300)**	**22.5 (12.3 to 33.1)**	**6.5 (5.3 to 7.2)**	**-36.0 (-39.4 to -32.6)**
High income	219 (182 to 248)	2.4 (-3.1 to 7.7)	4.6 (4.0 to 5.1)	-35.4 (-37.3 to -33.3)
Upper-middle income	619 (524 to 739)	18.0 (3.6 to 34.1)	8.6 (7.2 to 10.2)	-35.8 (-40.4 to -31.2)
Lower-middle income	247 (171 to 340)	47.8 (36.0 to 59.9)	4.7 (3.2 to 6.2)	-33.3 (-36.5 to -29.7)
Low income	67.3 (46.1 to 83.4)	75.7 (60.5 to 90.7)	7.1 (4.8 to 9.1)	-35.5 (-38.4 to -32.5)
**Colon and rectum cancer**				
**Global**	**2210 (1540 to 3070)**	**93.5 (42.2 to 154.7)**	**12.0 (9.0 to 15.9)**	**-2.0 (-21.7 to 21.5)**
High income	716 (567 to 878)	49.6 (22.9 to 79.6)	15.1 (12.4 to 18.4)	-4.9 (-19.8 to 12.5)
Upper-middle income	928 (719 to 1280)	103.3 (43.4 to 174.8)	12.6 (10.1 to 16.6)	7.5 (-17.7 to 36.6)
Lower-middle income	412 (140 to 761)	144.9 (67.4 to 233.0)	7.8 (2.8 to 14.4)	9.5 (-17.6 to 40.6)
Low income	122 (54.6 to 203)	229.3 (137.2 to 329.8)	13.2 (7.2 to 21.6)	18.6 (-5.2 to 44.8)
**Tracheal, bronchus, and lung cancer**				
**Global**	**3390 (2570 to 4290)**	**62.5 (26.2 to 104.0)**	**18.7 (14.9 to 22.8)**	**-15.2 (-28.9 to 1.1)**
High income	955 (802 to 1140)	27.5 (11.0 to 46.3)	22.3 (19.5 to 26.8)	-12.6 (-22.0 to -1.9)
Upper-middle income	1790 (1380 to 2270)	70.4 (26.7 to 120.6)	24.4 (19.1 to 29.9)	-7.5 (-25.5 to 13.2)
Lower-middle income	513 (275 to 804)	107.7 (53.0 to 167.8)	9.7 (6.0 to 15.4)	-5.0 (-24.3 to 15.8)
Low income	80.3 (54.9 to 121)	146.7 (81.1 to 218.4)	8.9 (6.4 to 13.3)	-10.4 (-28.3 to 9.2)
**Cancer types highlighted in active WHO initiatives**				
**Breast cancer**				
**Global**	**1400 (880 to 2060)**	**74.9 (37.2 to 118.0)**	**8.9 (6.2 to 12.4)**	**2.3 (-16.3 to 23.3)**
High income	333 (270 to 403)	33.7 (15.8 to 52.2)	8.2 (6.9 to 9.8)	-8.9 (-18.8 to 1.8)
Upper-middle income	348 (255 to 435)	52.9 (16.3 to 95.5)	5.8 (4.4 to 7.0)	-1.7 (-20.9 to 19.8)
Lower-middle income	577 (251 to 953)	115.7 (59.9 to 176.5)	11.4 (5.9 to 19.1)	9.2 (-13.9 to 34.6)
Low income	159 (78.7 to 255)	196.7 (132.2 to 266.9)	15.0 (7.8 to 23.3)	15.3 (-2.2 to 34.4)
**Cervical cancer**				
**Global**	**521 (319 to 817)**	**39.3 (23.6 to 57.3)**	**3.7 (2.0 to 6.4)**	**-10.0 (-22.7 to 5.8)**
High income	42.3 (36.1 to 48.1)	2.4 (-4.4 to 8.3)	1.3 (1.1 to 1.5)	-25.0 (-30.4 to -19.2)
Upper-middle income	129 (100 to 161)	14.0 (0.7 to 27.8)	2.3 (1.7 to 3.1)	-21.5 (-32.4 to -8.1)
Lower-middle income	244 (114 to 401)	51.7 (32.0 to 75.6)	4.9 (1.7 to 9.8)	-19.4 (-32.6 to -2.9)
Low income	118 (72.7 to 169)	102.5 (78.7 to 129.5)	10.2 (5.7 to 15.4)	-20.0 (-30.6 to -6.9)
**Childhood cancer**				
**Global**	**116 (97.3 to 144)**	**-18.4 (-32.1 to 0.2)**	**4.9 (4.2 to 5.9)**	**-9.5 (-19.6 to 7.0)**
High income	5.64 (5.00 to 6.25)	-27.6 (-35.6 to -18.4)	2.1 (1.9 to 2.3)	-18.0 (-24.6 to -9.8)
Upper-middle income	22.5 (20.0 to 26.2)	-42.5 (-51.1 to -31.3)	4.4 (4.0 to 5.2)	-14.3 (-25.2 to -0.9)
Lower-middle income	49.2 (40.2 to 63.2)	-21.3 (-35.9 to -1.6)	4.6 (3.9 to 5.8)	-9.5 (-20.5 to 8.0)
Low income	39.7 (32.8 to 49.6)	22.0 (-0.1 to 51.3)	7.8 (6.5 to 9.3)	-13.6 (-23.0 to 2.8)

“Childhood cancers” refers to total cancers in ages <20, and rates are crude, not age-standardised. “Cervical cancer” refers to females only. Abbreviations: UI, 95% uncertainty interval

Appendix Table 11. Composition of cancer DALYs in 2023 by contribution of YLDs and YLL, globally and by World Bank income groups for all sexes combined

	Contribution YLDs, % (UI)	Contribution YLLs, % (UI)
**Global**	**3.0 (2.2 to 4.1)**	**97.0 (95.9 to 97.8)**
**World Bank income group**		
High income	5.1 (3.7 to 6.9)	94.9 (93.1 to 96.3)
Upper-middle income	2.7 (1.9 to 3.6)	97.3 (96.4 to 98.1)
Lower-middle income	1.8 (1.4 to 2.4)	98.2 (97.6 to 98.6)
Low income	1.7 (1.2 to 2.3)	98.3 (97.7 to 98.8)

Abbreviations: DALYs, disability-adjusted life years; YLDs, years lived with disability; YLLs, years of life lost; UI, 95% uncertainty interval
