# Supplementary material for: The global, regional, and national burden of cancer, 1990–2023, with forecasts to 2050: a systematic analysis for the Global Burden of Disease Study 2023
Source: Lancet. Author manuscript; Available in PMC 2025 Dec 9. (PMC12687902; doi:10.1016/S0140-6736(25)01635-6)
Supplement: Authorship appendix [file NIHMS2113905-supplement-Authorship_appendix.pdf]

Appendix 3: Authorship appendix to "The global, regional, and national burden of cancer, 1990–2023, with forecasts to 2050: a systematic analysis for the Global Burden of Disease Study 2023"

This appendix provides further authorship detail for "The global, regional, and national burden of cancer, 1990–2023, with forecasts to 2050: a systematic analysis for the Global Burden of Disease Study 2023"

Table of Contents

Appendix 3: Authorship appendix to "The global, regional, and national burden of cancer, 1990–2023, with forecasts to 2050: a systematic analysis for the Global Burden of Disease Study 2023" 1

GBD 2023 Cancer Collaborators 2

Affiliations 8

Authors' Contributions........ 41

- Managing the overall research enterprise........ 41
- Writing the first draft of the manuscript 41
- Primary responsibility for applying analytical methods to produce estimates 41
- Primary responsibility for seeking, cataloguing, extracting, or cleaning data; designing or coding figures and tables........ 41
- Providing data or critical feedback on data sources........ 41
- Developing methods or computational machinery 44
- Providing critical feedback on methods or results 44
- Drafting the work or revising it critically for important intellectual content 49
- Managing the estimation or publications process........ 54

GBD 2023 Cancer Collaborators

Lisa M Force**, Jonathan M Kocarnik, Miranda L May, Kayleigh Bhangdia, Andrew Crist, Louise Penberthy, Natalie Pritchett, Alistair Acheson, Lee Deitesfeld, Bhoomadevi A, Hasan Aalruz, Nasir Abbas, Maryam Abbasalipour Bashash, Abdallah H A Abd Al Magied, Samar Abd ElHafeez, Ashraf Nabiel Abdalla, Mohammed Altigani Abdalla, Wael M Abdel-Rahman, Parsa Abdi, Wakgari Mosisa Abdisa, Daba Abdissa, Arash Abdollahi, Meriem Abdoun, Arman Abdous, Auwal Abdullahi, Mesfin Abebe, Armita Abedi, Syed Hani Abidi, Alemwork Abie, Olumide Abiodun, Richard Gyan Aboagye, Hassan Abolhassani, Ulric Sena Abonie, Lucas Guimarães Abreu, Aminu Kende Abubakar, Bilyaminu Abubakar, Eman Abu-Gharbieh, Hana J Abukhadijah, Salahdein Aburuz, Ahmed Abu-Zaid, Raghu Ram Achar, Juan Manuel Acuna, Lisa C Adams, Lawan Hassan Adamu, Isaac Yeboah Addo, Kamoru Ademola Adedokun, Nurudeen A Adegoke, Victor Adekanmbi, Ibukun Modupe Adesiyan, Habeeb Omoponle Adewuyi, Usha Adiga, Qorinah Estiningtyas Sakilah Adnani, Prince Owusu Adoma, Leticia Akua Adzigbli, Ahmed M Afifi, Aanuoluwapo Adeyimika Afolabi, Fatemeh Afrashteh, Arya Afrooghe, Muhammad Sohail Afzal, Suneth Buddhika Agampodi, Navidha Aggarwal, Feleke Doyore Agide, César Agostinis Sobrinho, Williams Agyemang-Duah, Bright Opoku Ahinkorah, Aqeel Ahmad, Danish Ahmad, Faisal Ahmad, Khurshid Ahmad, Muayyad M Ahmad, Sajjad Ahmad, Tauseef Ahmad, Elham Ahmadi, Amir Mahmoud Ahmadzade, Mohadese Ahmadzade, Anisuddin Ahmed, Ayman Ahmed, Gasha Salih Ahmed, Ibrar Ahmed, Luai A Ahmed, Mehrunnisha Sharif Ahmed, Meqdad Saleh Ahmed, Muktar Beshir Ahmed, Nesredin Ahmed, Syed Anees Ahmed, Dolapo Emmanuel Ajala, Marjan Ajami, Roland Eghoghosoa Akhigbe, Karolina Akinosoglou, Salah Al Awaidy, Hanadi Al Hamad, Syed Mahfuz Al Hasan, Omar Al Omari, Mohammad Al Qadire, Yazan Al Thaher, Omar Ali Mohammed Al Zaabi, Khurshid Alam, Zufishan Alam, Amani Alansari, Fahmi Y Al-Ashwal, Laila Ismael Al-Daken, Wafa A Aldhaleei, Shereen M Aleidi, Bezawit Abeje Alemayehu, Ayman Al-Eyadhy, Abdelazeem M Algammal, Adel Ali Saeed Al-Gheethi, Mohammed Khaled Al-Hanawi, Dari Alhuwail, Abid Ali, Endale Alemayehu Ali, Mohammad Daud Ali, Mohammed Usman Ali, Rafat Ali, Syed Shujait Ali, Waad Ali, Sheikh Mohammad Alif, Samah W Al-Jabi, Syed Mohamed Aljunid, Ahmad Alkhatib, Nihad A Almasri, Hesham M Al-Mekhlafi, Mohmmad Minwer Alnaeem, Hasan Yaser Alniss, Margret Beaula Alocious Sukumar, Mahmoud A Alomari, Mohammad R Alosta, Saleh A Alqahtani, Ahmad Rajeh Al-Qudimat, Rajaa M Mohammad Al-Raddadi, Ahmad Alrawashdeh, Intima Alrimawi, Sahel Majed Alrousan, Salman Khalifah Al-Sabah, Najim Z Alshahrani, Awais Altaf, Alaa B Al-Tammemi, Elysia M Alvarez, Nelson Alvis-Guzman, Yaser Mohammed Al-Worafi, Hany Aly, Mohammad Sharif Ibrahim Alyahya, Abdallah Alzoubi, Karem H Alzoubi, Md Akib Al-Zubayer, Masoud Aman Mohammadi, Tarek Tawfik Amin, Alireza Amindarolzarbi, Shiva Aminnia, Nafiu Aminu, Mohammad Hosein Amirzade-Iranaq, Hubert Amu, Ganiyu Adeniyi Amusa, Robert Ancuceanu, Deanna Anderlini, Abhishek Anil, Amir Anoushiravani, Hossein Ansariniya, Iyadunni Adesola Anuoluwa, Saeid Anvari, Saleha Anwar, Sumadi Lukman Anwar, Razique Anwer, Ekenedilichukwu Emmanuel Anyabolo, Anayochukwu Edward Anyasodor, Angela Esi Apeagyei, Juan Pablo Arab, Jalal Arabloo, Razman Arabzadeh Bahri, Elshaimaa A Arafa, Mosab Arafat, Aleksandr Y Aravkin, Martina Arcieri, Demelash Areda, Hany Ariffin, Mesay Arkew, Anton A Artamonov, Santhosh Arul, Deepavalli Arumuganainar, Mohammad Asghari-Jafarabadi, Akram Ashames, Muhammad Abdul Basit Ashraf, Tahira Ashraf, Bernard Kwadwo Yeboah Asiamah-Asare, Haftu Asmerom Asmerom, Batyrbek Assembekov, Smita Asthana, Prince Atorkey, Maha Moh'd Wahbi Atout, Julie Alaere Atta, Marcel Ausloos, Atalel Fentahun Awedew, Adedapo Wasiu Awotidebe, Beatriz Paulina Ayala Quintanilla, Haleh Ayatollahi, Seyed Mohammad Ayyoubzadeh, Ali Azargoonjahromi, Amirali Azimi, Mohd Yusmaidie Aziz, Sadat Abdulla Aziz, Shahkaar Aziz, Hosein Azizi, Ahmed Y Azzam, Muhammad Badar, Ashish D Badiye, Alaa Aboelnour Badran, Nasser Bagheri, Fereshteh Baghizadeh, Razieh Bahreini, Ruhai Bai, Atif Amin Baig, Lovenish Bains, Ram Chandra Bajpai, Shankar M Bakkannavar, Auwal Adam Bala, Wondu Feyisa

Balcha, Mohammadreza Balooch Hasankhani, Soham Bandyopadhyay, Rajon Banik, Aleksandra Barac, Shirin Barati, Mainak Bardhan, Suzanne Lyn Barker-Collo, Hiba Jawdat Barqawi, Amadou Barrow, Zarrin Basharat, Shahid Bashir, Afisu Basiru, Pritish Baskaran, Mohammad-Mahdi Bastan, Kavita Batra, Matteo Bauckneht, Mohsen Bayati, Feyisa Shasho Bayisa, Narasimha M Beeraka, Babak Behnam, Amir Hossein Behnoush, Payam Behzadi, Diana Fernanda Bejarano Ramirez, Sewunet admasu Belachew, Gokce Belge Bilgin, Luis Belo, Habib Benzian, Alemshet Yirga Yirga Berhie, Amiel Nazer C Bermudez, Ajeet Singh Bhadoria, Akshaya Srikanth Bhagavathula, Nickhill Bhakta, Neeraj Bhala, Ravi Bharadwaj, Nikha Bhardwaj, Pankaj Bhardwaj, Prarthna V Bhardwaj, Sonu Bhaskar, Ajay Nagesh Bhat, Vivek Bhat, Shuvarthi Bhattacharjee, Gurjit Kaur Bhatti, Jasvinder Singh Bhatti, Mohiuddin Ahmed Bhuiyan, Soumitra S Bhuyan, Raluca Bievel-Radulescu, Cem Bilgin, Catherine Bisignano, Mohammad Shahangir Biswas, Monirujjaman Biswas, Bruno Bizzozero-Peroni, Tone Bjørge, Trupti Bodhare, Obasanjo Afolabi Bolarinwa, Archith Boloor, Hamed Borhany, Samuel Adolf Bosoka, Alejandro Botero Carvajal, Souad Bouaoud, Marija M Bozic, Dejana Braithwaite, Michael Brauer, Hermann Brenner, Linh Phuong Bui, Katrin Burkart, Felix Busch, Maria Teresa Bustamante-Teixeira, Yasser Bustanji, Nadeem Shafique Butt, Zahid A Butt, Guangyao Cai, Luis Alberto Cámera, Ismael Campos-Nonato, Si Cao, Yubin Cao, Angelo Capodici, Giulia Carreras, Márcia Carvalho, Carlos A Castañeda-Orjuela, Ferrán Catalá-López, Maria Sofia Cattaruzza, Luca Cegolon, Francieli Cembranel, Ester Cerin, Achille Cernigliaro, Sonia Cerrai, Aditya Chakraborty, Chiranjib Chakraborty, Sandip Chakraborty, Rama Mohan Chandika, Vijay Kumar Chattu, Anis Ahmad Chaudhary, Sirshendu Chaudhuri, Akhilanand Chaurasia, Galmesa Bekana Chemeda, Hana Chen, Kai Chen, Meng Xuan Chen, Fatemeh Chichagi, Odgerel Chimed-Ochir, Patrick R Ching, Jesus Lorenzo Chirinos-Caceres, William C S Cho, Dong-Woo Choi, Sungchul Choi, Bryan Chong, Yuen Yu Chong, Hitesh Chopra, Shivani Chopra, Hou In Chou, Sonali Gajanan Choudhari, Dinh-Toi Chu, Isaac Sunday Chukwu, Eric Chung, Sunghyun Chung, Iolanda Cioffi, Alyssa Columbus, Joao Conde, Vera Marisa Costa, Natalia Cruz-Martins, Bashir Dabo, Omid Dadras, Xiaochen Dai, Lalit Dandona, Rakhi Dandona, Lucio D'Anna, Pojsakorn Danpanichkul, Reza Darvishi Cheshmeh Soltani, Aso Mohammad Darwesh, Saswati Das, Nihar Ranjan Dash, Mohsen Dashti, Cornelius Dassah, Dimash Davletov, Fernando Pio De la Hoz, Frances E Dean, Edward Christopher Dee, Sindhura Deekonda, Mohammad Delsoz, Abel Desalegn Demeke, Edgar Denova-Gutiérrez, Rupak Desai, Aragaw Tesfaw Desale, Gashaw Dessie, Devananda Devegowda, Syed Masudur Rahman Dewan, Arkadeep Dhali, Amol S Dhane, Meghnath Dhimal, Sameer Dhingra, Bibha Dhungel, Marcello Di Pumpo, Luis Antonio Diaz, Michael J Diaz, Thao Huynh Phuong Do, Saeid Doaei, Francesco Dondi, Fariba Dorostkar, Wendel Mombaque dos Santos, Ojas Prakashbhai Doshi, Leila Doshmangir, Taiwo Omotayo Dosumu, Robert Kokou Dowou, Menayit Tamrat Dresse, John Dube, Senbagam Duraisamy, Oyewole Christopher Durojaiye, Sulagna Dutta, Arkadiusz Marian Dziedzic, Abdel Rahman E'mar, Hisham Atan Edinur, Behrad Eftekhari, Ashkan Eighaei Sedeh, Michael Ekholuenetale, Temitope Cyrus Ekundayo, Rabie Adel El Arab, Abdelfatteh EL Omri, Iman El Sayed, Maysaa El Sayed Zaki, Reza Elahi, Noha Mousaad Elemam, Muhammed Elhadi, Waseem El-Huneidi, Sherif Elkannishy, Ahmed O Elmehrath, Adel B Elmoselhi, Randa Elsheikh, Yasser Mohamed El-Sherbiny, Ibrahim Elsohaby, Abdelgawad Salah Abdelgawad Eltahawy, Mohd Elmagzoub Eltahir, Syed Emdadul Haque, Theophilus I Emeto, Talha Bin Emran, Destaw Endeshaw, Babak Eshrati, Majid Eslami, Sayeh Ezzikouri, Heidar Fadavian, Adeniyi Francis Fagbamigbe, Ayesha Fahim, Saman Fahimi, Aamir Fahira, Ildar Ravisovich Fakhradiyev, Aliasghar Fakhri-Demeshghieh, Luca Falzone, Alireza Farahani, Mohammad Farahmand, Seyed Nooreddin Faraji, Mahsa Faramarzpour, Ebrahim Farashi, Zaki Farhana, MoezAlIslam Ezzat Mahmoud Faris, Pawan Sirwan Faris, Andre Faro, Umar Farooque, Hossein Farrokhpour, Abidemi Omolara Fasanmi, Kiana Fasihi, Emmanuel Toluwani Fasusi, Hamed Fattahi, Alireza Feizkhah, Ginenus Fekadu, Daniela Ferrante, Pietro Ferrara, Nuno Ferreira, Getahun Fetensa, Florian Fischer, James L

Fisher, Bobirca Teodor Florin, Olumuyiwa Shola Folayan, Arianna Fornari, Ali Forouhari, Behzad Foroutan, Maryam Fotouhi, Takeshi Fukumoto, Ami Fukunaga, Muktar A Gadanya, Márió Gajdács, Silvano Gallus, Adamu Usman Gamawa, Aravind P Gandhi, Shivaprakash Gangachannaiah, Mounika Gangireddy, Jacopo Garlasco, Rupesh K Gautam, Feven Sahle Gebre, Miglas Welay Gebregergis, Mesfin Gebrehiwot, Teferi Gebru Gebremeskel, Haftay Gebremedhin Gebreslassie, Gebremariam Wulie Geremew, Genanew K Getahun, Sadegh Ghafarian, Amir Ghaffari Jolfayi, Lobna Faiz Gharaibeh, Haniyeh Ghasrsaz, Fariba Ghassemi, Ramy Mohamed Ghazy, Maryam Gholamalizadeh, Nasim Gholizadeh, Elena Ghotbi, Konstantinos Giannakis, Artyom Urievich Gil, Syed Abdullah Gilani, Alem Abera Girmay, Alessandro Girombelli, Amit Goel, Anil Kumar Goel, Mahaveer Golechha, Davide Golinelli, MReza Goodarzian, Giuseppe Gorini, Changhao Gu, Shi-Yang Guan, Giovanni Guarducci, Mohammed Ibrahim Mohialdeen Gubari, Zheng Guo, Zhifeng Guo, Bhawna Gupta, Sapna Gupta, Reyna Alma Gutiérrez, Jose Guzman-Esquivel, Adrina Habibzadeh, Farrokh Habibzadeh, Parham Habibzadeh, Tesfahun Simon Simon Hadaro, Najah R Hadi, Dariush Haghmorad, Nguyen Hai Nam, Haimanot Ewnetu Hailu, Hassen Mosa Mosa Halil, Islam M Hamad, Randah R Hamadeh, Nadia M Hamdy, Sajid Hameed, Nasrin Hanifi, Ashanul Haque, Josep Maria Haro, Ahmed I Hasaballah, Md Kamrul Hasan, S M Mahmudul Hasan, Fatemeh Hasani, Hamidreza Hasani, Alireza Hasanzadeh, Abdiwahab Hashi, Md Saquib Hasnain, Ikrama Ibrahim Hassan, Treska S Hassan, Mahgol Sadat Hassan Zadeh Tabatabaei, Johannes Haubold, Simon I Hay, Khezar Hayat, Guohua He, Qiang He, Wen-Qiang He, Behzad Heibati, Mohammad Heidari, Claudiu Herteliu, Hamed Hesami, Majid Heydari, Mojtaba Heydari, Zahra Heydarifard, Demisu Zenbaba Heyi, Kamal Hezam, Yuta Hiraike, Mai Hoang, Ramesh Holla, Nobuyuki Horita, Mehdi Hoseinzadeh, H Dean Hosgood, Alamgir Hossain, Md Mahbub Hossain, Md Sabbir Hossain, Mohammad Bellal Hossain, Mohammad-Salar Hosseini, Ahmad Hosseinzadeh Adli, Mihaela Hostiuc, Vivian Chia-rong Hsieh, Chengxi Hu, Junjie Hu, Junjie Huang, Kiavash Hushmandi, Javid Hussain, Salman Hussain, Dursa Hussein, Nawfal R Hussein, Mohamed Ibrahim Husseiny, Hong-Han Huynh, Ivo Iavicoli, Segun Emmanuel Ibitoye, Reem Ibrahim, Anel Ibrayeva, Francisco Javier Idalsoaga, Pulwasha Maria Iftikhar, Adalia Ikiroma, Olayinka Stephen Ilesanmi, Irena M Ilic, Milena D Ilic, Muhana Fawwazy Ilyas, Salim Ilyasu, Mohammad Tarique Imam, Mustapha Immurana, Lucius Chidiebere Imoh, Javed Iqbal, Mustafa Alhaji Isa, Md Rabiul Islam, Faisal Ismail, Ramzy Issa, Masao Iwagami, Chidozie Declan Iwu, Mahalaxmi Iyer, Louis Jacob, Farhad Jadidi-Niaragh, Abdollah Jafarzadeh, Haitham Jahrami, Ayushi Jain, Ammar Abdulrahman Jairoun, Abhishek Jaiswal, Mihajlo Jakovljevic, Ali Jaliliyan, Reza Jalilzadeh Yengejeh, Mohamed L Jalloh, Qazi Mohammad Sajid Jamal, Safayet Jamil, Syed Sarmad Javaid, Talha Jawaid, Umesh Jayarajah, Shubha Jayaram, Ruwan Duminda Jayasinghe, Sun Ha Jee, Diptismita Jena, Shuai Jin, Wenyi Jin, Mohammad Jokar, Jost B Jonas, Tamas Joo, Jobin Jose, Abel Joseph, Nitin Joseph, Charity Ehimwenma Joshua, Farahnaz Joukar, Mikk Jürisson, Malik E Juweid, Anupam Jyoti, Billingsley Kaambwa, Ali Kabir, Zubair Kabir, Maryam Kabiri, Dler Hussein Kadir, Ebbie Kalan, Leila R Kalankesh, Mehnaz Kamal, Vineet Kumar Kamal, Sivesh Kathir Kamarajah, Arun Kamireddy, Ramat T. Kamorudeen, Fatemeh Kanaani Nejad, Samuel Berchi Kankam, Kehinde Kazeem Kanmodi, Neeti Kapoor, Mehrdad Karajizadeh, Jafar Karami, Reema A Karasneh, André Karch, Aliasghar Karimi, Mohammad Amin Karimi, Sahand Karimzadhagh, Tomasz M Karpiński, Faizan Zaffar Kashoo, Joonas H Kauppila, Navjot Kaur, Mohd Adnan Kausar, Foad Kazemi, Fekadu Abera Kebede, Ariz Keshwani, Emmanuelle Kesse-Guyot, Yousef Saleh Khader, Himanshu Khajuria, Asaad Khalid, Hazim O Khalifa, Anita Khalili, Pantea Khalili, Alireza Khalilian, Ghazaleh Khalili-Tanha, Mohamed khalis, Faham Khamesipour, Ajmal Khan, Faiz Ullah Khan, Gulfaraz Khan, Mohammad Jobair Khan, Moien AB Khan, Salman Ali Khan, Shaghayegh Khanmohammadi, Moawiah Mohammad Khatatbeh, Mahalaqua Nazli Khatib, Maryam Khayamzadeh, Hamid Reza Khayat Kashani, Daniel Kheradmand, Feriha Fatima Khidri, Atulya Aman Khosla, Mohammad Ali Khosravi, Jagdish

Khubchandani, Kwanghyun Kim, Yun Jin Kim, Yun Seo Kim, Ruth W Kimokoti, Adnan Kisa, Sezer Kisa, Shivakumar KM, Michail Kokkorakis, Ali-Asghar Kolahi, Farzad Kompani, Jianqiu Kong, Anastasios Georgios Konstas, Karel Kostev, Ashwin Laxmikant Kotnis, Sindhura Lakshmi Koulmane Laxminarayana, Irene Akwo Kretchy, Kewal Krishan, Raja Amir Hassan Kuchay, Mohammed Kuddus, Mukhtar Kulimbet, Dewesh Kumar, G Anil Kumar, Nithin Kumar, Vijay Kumar, Satyajit Kundu, Setor K Kunutsor, Pramod Kumar Kushawaha, Christina Yeni Yeni Kustanti, Tezer Kutluk, Ambily Kuttikkattu, Assylkhan Kuttybayev, Grace Kwakyewaa Kyei, Frank Kyei-Arthur, Ville Kytö, Carlo La Vecchia, Muhammad Awwal Ladan, Chandrakant Lahariya, Daphne Teck Ching Lai, Hanpeng Lai, Balzhan Lakanova, Dharmesh Kumar Lal, Judit Lám, Francesco Lanfranchi, Savita Lasrado, Mahrukh Latif, Paolo Lauriola, Basira Kankia Lawal, Huu-Hoai Le, Minh Huu Nhat Le, Nhi Huu Hanh Le, Thao Thi Thu Le, Caterina Ledda, Ivan Lee, Sang-woong Lee, Seung Won Lee, Shaun Wen Huey Lee, Wei-Chen Lee, Awol Yemane Legesse, James Leigh, Elvynna Leong, Ming-Chieh Li, Wang-Zhong Li, Wei Li, Xiaopan Li, Yongze Li, Zhengrui Li, Zhihui Li, Stephen S Lim, Queran Lin, Chaojie Liu, Gang Liu, Jue Liu, Liu Liu, Xiaofeng Liu, Xuefeng Liu, Erand Llanaj, Jailos Lubinda, Angelina M Lutambi, Miltiadis D Lytras, Ellina Lytvyak, Hawraz Ibrahim M Amin, Zheng Feei Ma, Raymond Saa-Eru Maalman, Farzan Madadizadeh, Seyedeh Panid Madani, Alireza Mafi, Azzam A Maghazachi, Abdulahi Abdiwali Mahamed, Nozad Hussein Mahmood, Mansour Adam Mahmoud, Rashidul Alam Mahumud, Azeem Majeed, Abdelrahman M Makram, Omar M Makram, Konstantinos Christos C Makris, Satyaveni Malasala, Hadi Maleki-Kakelar, Kashish Malhotra, Ahmad Azam Malik, Iram Malik, Lesibana Anthony Malinga, Deborah Carvalho Malta, Vahid Mansouri, Marjan Mansourian, Mohammad Ali Mansournia, Lorenzo Giovanni Mantovani, Changkun Mao, Hamid Reza Marateb, Basma Hamed Marghani, Mirko Marino, Santi Martini, Miquel Martorell, Roy Rillera Marzo, Sammer Marzouk, Yasith Mathangasinghe, Medha Mathur, Fernanda Penido Matozinhos, Indu Liz Matthew, Martin McKee, Steven M McPhail, Enkeleint A Mechili, Jitendra Kumar Meena, Elahe Meftah, Riffat Mehboob, Asim Mehmood, Tesfahun Mekene Meto, Hadush Negash Meles, Addisu Melese, Max Alberto Mendez-Lopez, Walter Mendoza, Ritesh G Menezes, Dominik Menges, Endalkachew Worku Mengesha, Tara P Menon, Alexios-Fotios A Mentis, Sultan Ayoub Ayoub Meo, Atte Meretoja, Tuomo J Meretoja, Tomislav Mestrovic, Mohamed M M Metwally, Tomasz Miazgowski, Irmina Maria Michalek, Ted R Miller, Giuseppe Minervini, Mojgan Mirghafourvand, Awoke Misganaw, Sanjeev Misra, Prasanna Mithra, Yidnek Mogessie, Ashraf Mohamadkhani, Jama Mohamed, Mona Gamal Mohamed, Nouh Saad Mohamed, Karzan Abdulmuhsin Mohammad, Taj Mohammad, Sakineh Mohammad-Alizadeh-Charandabi, Abdolreza Mohammadi, Mohammad Kazem Mohammadi, Seyed Omid Mohammadi, Abdollah Mohammadian-Hafshejani, Ibrahim Mohammadzadeh, Zeinab Mohammadzadeh, Al-Kassim Hassan Mohammed, Mustapha Mohammed, Shafiu Mohammed, Amin Mohsenzadeh, Ali H Mokdad, Hossein Molavi Vardanjani, Sabrina Molinaro, Shaher Momani, Lorenzo Monasta, Mohammad Ali Moni, Yousef Moradi, Paula Moraga, Shane Douglas Morrison, Mahmoud M Morsy, Elias Mossialos, Fatemeh Motaharinezhad, Rohith Motappa, Kimia Mozahheb Yousefi, Ahmed Msherghi, Rabia Mubarak, Sumaira Mubarik, George Duke Mukoro, Admir Mulita, Kavita Munjal, Efren Murillo-Zamora, Ali Mushtaq, Saima Mushtaq, Sherzad Ibrahim Mustafa, Saravanan Muthupandian, Woojae Myung, Amin Nabavi, Ahamarshan Jayaraman Nagarajan, Gabriele Nagel, Pirouz Naghavi, Gurudatta Naik, Mukhammad David Naimzada, Firzan Nainu, Tapas Sadasivan Nair, Hastyar Hama Rashid Najmuldeen, Hae Sung Nam, Shumaila Nargus, Yvonne Nartey, Abdulqadir J Nashwan, Mahmoud Nassar, Zuhair S Natto, Javaid Nauman, Nawsherwan , Biswa Prakash Nayak, Md Fahad Shahariar Nayon, Athare Nazri-Panjaki, Pacifique Ndishimye, Amanuel Tebabal Nega, Masoud Negahdary, Ionut Negoi, Chakib Nejjari, Omid Nekouei, Gaurav Nepal, Henok Biresaw Netsere, Jean Claude Semuto Semuto Ngabonziza, Cuong Tat Nguyen, Dang Nguyen, Kieu Viet Nhi Nguyen, Nhan Nguyen, The Phuong Nguyen, Tuan Thanh Nguyen, Van Thanh Nguyen, Robina Khan Niazi, Luciano

Nieddu, Nasrin Nikravangolsefid, Vikram Niranjan, Jean Marie Vianney Niyonsenga, Chukwudi A Nnaji, Lawrence Achilles Nnyanzi, Shuhei Nomura, Nurulamin M Noor, Syed Toukir Ahmed Noor, Mohammadamin Noorafrooz, Masoud Noroozi, Chisom Adaobi Nri-Ezedi, Fred Nugen, Dieta Nurrika, Chimezie Igwegbe Nzoputam, Ogochukwu Janet Nzoputam, Bogdan Oancea, Vincent Adeiza Obakachi, Ismail A Odetokun, Michael Safo Oduro, James Odhiambo Oguta, In-Hwan Oh, Hassan Okati-Aliabad, Sylvester Reuben Okeke, Akinkunmi Paul Okekunle, Osaretin Christabel Okonji, Andrew T Olagunju, Timothy Olusegun Olusegun Olanrewaju, Matthew Idowu Olatubi, Folorunsho Bright Omage, Hany A Omar, Goran Latif Omer, Abidemi E Omonisi, Sok King Ong, Obinna E Onwujekwe, Marcel Opitz, Michal Ordak, Verner N Orish, Atakan Orscelik, Esteban Ortiz-Prado, Augustus Osborne, Alaa AM Osman, Samuel M Ostroff, Uchechukwu Levi Osuagwu, Elham H Othman, Oche Joseph Otorkpa, Stanislav S Otstavnov, Amel Ouyahia, Mayowa O Owolabi, Oladayo Ayobami Oyebanji, Kolapo Oyebola, Ilker Ozsahin, Mahesh P A, Alicia Padron-Monedero, Jagadish Rao Padubidri, Tamás Palicz, Sujogya Kumar Panda, Songhomitra Panda-Jonas, Deepshikha Pande Katare, Seithikurippu R Pandi-Perumal, Ke Pang, Georgios D Panos, Leonidas D Panos, Ioannis Pantazopoulos, Mario Virgilio Papa, Parinaz Paranjkhoo, Peyvand Parhizkar Roudsari, Romil R Parikh, Eun-Cheol Park, Maja Pasovic, Roberto Passera, Jay Patel, Riya Jayesh Patel, Shankargouda Patil, Apurba Patra, Hamidreza Pazoki Toroudi, Jarmila Pekarcikova, Umberto Pensato, Prince Peprah, Jeevan Pereira, Mario F P Peres, Simone Perna, Richard G Pestell, Olumuyiwa James Peter, Fanny Emily Petermann-Rocha, Hoang Tran Pham, Tung Thanh Pham, Daniela Pierannunzio, Julian David Pillay, Zahra Zahid Piracha, Saeed Pirouzpanah, Ramesh Poluru, Arjun Pon Avudaiappan, Maarten J Postma, Reza Pourbabaki, Farzad Pourghazi, Disha Prabhu, Jalandhar Pradhan, Peralam Yegneswaran Prakash, Chandra P Prasad, Akila Prashant, Elton Junio Sady Prates, Dimas Ria Angga Pribadi, Jagadeesh Puvvula, Asma Saleem Qazi, Suli Qiu, Karzan Qurbani, Hadi Raeisi Shahraki, Ata Rafiee, Alireza Rafiei, Pracheth Raghuveer, Hawbash Mohammed-Amin Rahim, Fryad Majeed Rahman, Md Mijanur Rahman, Mohammad Meshbahur Rahman, Mosiur Rahman, Muhammad Aziz Rahman, Amir Masoud Rahmani, Saeed Rahmani, Masoud Rahmati, Pramila Rai, Diego Raimondo, Ivano Raimondo, Sathish Rajaa, Rayan Rajabi, Mohammad Amin Rajizadeh, Majed Ramadan, Chitra Ramasamy, Shakthi Kumaran Ramasamy, Pushkal Sinduvadi Ramesh, Zahra Ramezani, Juwel Rana, Rishabh Kumar Rana, Nemanja Rancic, Fatemeh Ranjbar Noei, Christopher Rao, Kumuda Rao, Mithun Rao, Sowmya J Rao, Vahid Rashedi, Mohammad-Mahdi Rashidi, Devarajan Rathish, Santosh Kumar Rauniyar, Ilari Rautalin, Salman Rawaf, Lal Rawal, Elrashdy Redwan, Sanika Rege, Ana Reis-Mendes, Stefano Restaino, Nazila Rezaei, Negar Rezaei, Nima Rezaei, Mohsen Rezaeian, Tércia Moreira Ribeiro da Silva, Maximiliano Ribeiro Ribeiro Guerra, Moattar Raza Rizvi, Thomas J Roberts, Hermano Alexandre Lima Rocha, João Rocha Rocha-Gomes, Thales Philipe Rodrigues da Silva, Ravi Rohilla, Peter Rohloff, Kevin T Root, Gholamreza Roshandel, Amirhossein Roshanshad, Himanshu Sekhar Rout, Guido Rovera, Nitai Roy, Sharmistha Roy, Korosh Saber, Maha Mohamed Saber-Ayad, Cameron John Sabet, Siamak Sabour, Basema Ahmad Saddik, Tarannom Sadegh, Mohammad Reza Saeb, Kanza Saeed, Mohd Saeed, Umar Saeed, Amene Saghazadeh, Fatemeh Saheb Sharif-Askari, Narjes Saheb Sharif-Askari, Amirhossein Sahebkar, Pragyan Monalisa Sahoo, Soumya Swaroop Sahoo, Mirza Rizwan Sajid, Mohamed A Saleh, Leili Salehi, Amir Salek Farrokhi, Marwa Rashad Salem, Sohrab Salimi, Pouria Samadi, Jayami Eshana Samaranayake, Yoseph Leonardo Samodra, Abdallah M Samy, Juan Sanabria, Milena M Santric-Milicevic, Made Ary Sarasmita, Aswini Saravanan, Saman Sargazi, Tanmay Sarkar, Gargi Sachin Sarode, Sachin C Sarode, Arash Sarveazad, Michele Sassano, Brijesh Sathian, Maheswar Satpathy, Reza Sattarpour, Jennifer Saulam, Monika Sawhney, Juhi Saxena, Benedikt Michael Schaarschmidt, Christophe Schinckus, Ione Jayce Ceola Schneider, Art Schuermans, Austin E Schumacher, Durairaj Sekar, Saravanan Sekaran, Mario Šekerija, Mohammad H Semreen, Ashenafi Kibret Sendekie, Pallav Sengupta, Yigit Can Senol, Subramanian Senthilkumaran, Dragos Serban, Yashendra Sethi, Seyed

Mohammad Seyed Alshohadaei, Homa Seyedmirzaei, Allen Seylani, Maryam Shabany, Mahan Shafie, Muhammad Shahab, Ataollah Shahbandi, Samiah Shahid, Syed Ahsan Ahsan Shahid, Hamid R Shahsavari, Moyad Jamal Shahwan, Masood Ali Shaikh, Muhammad Aaqib Shamim, Elahe Shams, Mehran Shams-Beyranvand, Anas Shamsi, Alfiya Shamsutdinova, Dan Shan, Mohd Shanawaz, Abhishek Shankar, Mohammed Shannawaz, Nigussie Tadesse Sharew, Amin Sharifan, Manoj Sharma, Rajesh Sharma, Ujjawal Sharma, Vishal Sharma, Shamee Shastry, Ramzi Shawahna, Maryam Shayan, Fateme Sheida, Samendra P Sherchan, Pavanchand H Shetty, Premalatha K Shetty, Ranjitha S Shetty, Suraj S Shetty, Mosa Shibani, Aminu Shittu, Velizar Shivarov, Zahra Shokati Eshkiki, Sinegugu Nosipho Shongwe, Sina Shool, Seyed Afshin Shorofi, Sunil Shrestha, Kerem Shuval, Yafei Si, Nicole R S Sibuyi, Emmanuel Edwar Siddig, Ahmed Kamal Siddiqi, Luís Manuel Lopes Rodrigues Silva, Amit Singh, Baljinder Singh, Harmanjit Singh, Jasvinder A Singh, Kalpana Singh, Mayank Singh, Paramdeep Singh, Prashant Kumar Singh, Puneetpal Singh, Satwinder Singh, Surjit Singh, Mukesh Kumar Sinha, Freddy Sitas, Dagne Feleke Siyoum, Anna Aleksandrovna Skryabina, Amanda E Smith, Farrukh Sobia, Solikhah Solikhah, Sameh S M Soliman, Aayushi Sood, Sandra Spearman, Bahadar S Srichawla, Suresh Kumar Srinivasamurthy, Devin Bailey Srivastava, Kurt Straif, Vetriselvan Subramaniyan, Auwal Garba Suleiman, Muritala Odidi Suleiman Odidi, Muhammad Suleman, Desy Sulistiyorini, Anusha Sultan Meo, Zhong Sun, Vinay Suresh, Hani Susianti, Chandan Kumar Swain, Lukasz Szarpak, Sree Sudha T Y, Payam Tabaee Damavandi, Rafael Tabarés-Seisdedos, Fatemeh Sadat Tabatabaei, Seyyed Mohammad Tabatabaei, Ramin Tabibi, Mohammad Tabish, Seyed Reza Taha, Yasaman Taheri Abkenar, Moslem Taheri Soodejani, Jabeen Taiba, Iman M Talaat, Mircea Tampa, Jacques Lukenze Tamuzi, Ker-Kan Tan, Manoj Tanwar, Saba Tariq, Seyed Mohammad Tavangar, Birhan Tsegaw Taye, Abdelghani Tbakhi, Abainash Tekola, Mohamad-Hani Temsah, Dufera Rikitu Terefa, Wegen Beyene Tesfamariam, Enoch Teye-Kwadjo, Lakshmi Thangavelu, Rekha Thapar, Rasiah Thayakaran, Mahalakshmi Thayumana Sundaram, Hadiza Theyra-Enias, Nihal Thomas, Nikhil Kenny Thomas, Jansje Henny Vera Ticoalu, Tenaw Yimer Tiruye, Krishna Tiwari, Roman Topor-Madry, Adetunji T Toriola, Mathilde Touvier, Marcos Roberto Tovani-Palone, Eugenio Traini, Bach Xuan Tran, Ngoc Ha Tran, Quynh Thuy Huong Tran, Thang Huu Tran, Domenico Trico, Aristidis Tsatsakis, Abdul Rohim Tualeka, Mike Tuffour Amirikah, Lawrence Sena Tuglo, Atta Ullah, Saeed Ullah, Srikanth Umakanthan, Lawan Umar, Shehu Salihu Umar, Umar Muhammad Umar, Brigid Unim, Bhaskaran Unnikrishnan, Dinesh Upadhya, Asokan Govindaraj Vaithinathan, Omid Vakili, Jef Van den Eynde, Javad Varasteh, Shoban Babu Varthya, Siavash Vaziri, Balachandar Vellingiri, Madhur Verma, Massimiliano Veroux, Georgios-Ioannis Verras, Simone Villa, Gabriela Ines Villanueva, Francesco S Violante, Giuseppe Vizzielli, Sergey Konstantinovitch Vladimirov, Vasily Vlassov, Stein Emil Vollset, Elpida Vounzoulaki, Yasir Waheed, Cong Wang, Ruixuan Wang, Xing Wang, Yanzhong Wang, Muhammad Waqas, Paul Ward, Toyiba Hiyaru Wassie, Kosala Gayan Weerakoon, Ronny Westerman, Anggi Lukman Wicaksana, Dakshitha Praneeth Wickramasinghe, Nuwan Darshana Darshana Wickramasinghe, Karn Wijarnpreecha, Peter Willeit, Marcin W Wojewodzic, Axel Walter Wolf, Felicia Wu, James Fan Wu, Zenghong Wu, Qing Xia, Zhijia Xia, Lishun Xiao, Wanqing Xie, Libo Xu, Suowen Xu, Mingyang Xue, Mukesh Kumar Yadav, Galal Yahya, Amir Yarahmadi, Mohamed A Yassin, Sanni Yaya, Pengpeng Ye, Subah Abderehim Yesuf, Saber Yezli, Dehui Yin, Biksegn Asrat Yirdaw, Dong Keon Yon, Naohiro Yonemoto, Mustafa Z Younis, Zabihollah Yousefi, Chuanhua Yu, Chun-Wei Yuan, Vesna Zadnik, Syed Nabeel Zafar, Manijeh Zaghampour, Fathiah Zakham, Nazar Zaki, Giulia Zamagni, Burhan Abdullah Zaman, Sojib Bin Zaman, Mohammad Javad Zare Sakhvidi, Michael Zastrozhin, Mohammed Zawiah, Mohammed G M Zeariya, Alemu Birara Zemariam, Sebastian Zensen, Jianrong Zhang, Jingya Zhang, Liqun Zhang, Xiaoyi Zhang, Zhi-Jiang Zhang, Yang Zhao, David X Zheng, Jinxin Zheng, Ming-Hua Zheng, Anthony Zhong, Claire Chenwen Zhong, Jiayan Zhou, Juexiao Zhou,

Bin Zhu, Abzal Zhumagaliuly, Magdalena Zielińska, Osama A Zitoun, Rafat Mohammad Zrieq, Mohammed Zuber, Sa'ed H Zyoud, Shaher H Zyoud, Theo Vos*, Christopher J L Murray*.

**First author
*Co-senior authors

Affiliations

Department of Health Metrics Sciences, School of Medicine (L M Force MD, A Y Aravkin PhD, K Burkart PhD, X Dai PhD, Prof R Dandona PhD, Prof S I Hay FMedSci, Prof S S Lim PhD, A Misganaw PhD, Prof A H Mokdad PhD, Prof S E Vollset DrPH, Prof T Vos PhD, Prof C J L Murray DPhil), Institute for Health Metrics and Evaluation (L M Force MD, J M Kocarnik PhD, M L May MPH, K Bhangdia MS, A Crist BSc, L Penberthy MS, N Pritchett DrPH, A Acheson BA, L Deitesfeld MA, A E Apeagyei PhD, A Y Aravkin PhD, C Bisignano MPH, Prof M Brauer DSc, K Burkart PhD, X Dai PhD, Prof L Dandona MD, Prof R Dandona PhD, F E Dean BA, Prof S I Hay FMedSci, Prof S S Lim PhD, T Mestrovic PhD, Prof A H Mokdad PhD, S M Ostroff PhD, M Pasovic MEd, A E Schumacher PhD, A E Smith MPA, S Spearman MS, Prof S E Vollset DrPH, C Yuan PhD, Prof T Vos PhD, Prof C J L Murray DPhil), Department of Applied Mathematics (A Y Aravkin PhD), School of Health Systems and Public Health (C Iwu MPH), Henry M Jackson School of International Studies (S M Ostroff PhD), University of Washington, Seattle, WA, USA; Division of Pediatric Hematology-Oncology (L M Force MD), St. Jude Children's Research Hospital, Seattle, WA, USA; Amity Institute of Public Health (Prof B A PhD), Amity University, Uttar Pradesh, India; Department of Nursing (H Aalruz PhD), Al Zaytoonah University of Jordan, Amman, Jordan; Centre for Regenerative Medicine and Health (N Abbas PhD), Chinese Academy of Sciences, Hong Kong, China; Department of Neuroscience (N Abbas PhD), Department of Infectious Diseases and Public Health (I Elsohaby PhD, G Fekadu PhD, O Nekouei PhD), Department of Biomedical Sciences (W Jin MD), City University of Hong Kong, Hong Kong, China; Molecular Medicine Department (M Abbasalipour Bashash PhD, M Khosravi PhD), Department of Immunology (A Salek Farrokhi PhD), Pasteur Institute of Iran, Tehran, Iran; College of Pharmacy (A H A Abd Al Magied MSc), Department of Pathological Sciences (A Alzoubi PhD), Department of Clinical Sciences (Prof E A Arafa PhD), College of Medicine (S Dutta PhD), Department of Education (M Eltahir PhD), Nonlinear Dynamics Research Center (NDRC) (Prof S Momani PhD), Center for Medical and Bio-Allied Health Sciences Research (Prof M J Shahwan PhD, A Shamsi PhD), Ajman University, Ajman, United Arab Emirates (A Ashames PhD); Department of Epidemiology (S Abd ElHafeez DrPH), Biomedical Informatics and Medical Statistics Department (Prof I El Sayed PhD), Tropical Health Department (R M Ghazy PhD), Department of Pathology (Prof I M Talaat PhD), Alexandria University, Alexandria, Egypt; College of Pharmacy (Prof A N Abdalla PhD), Umm Al-qura University, Makka, Saudi Arabia; Hull York Medical School (M A Abdalla PhD), University of Hull, Hull, UK; Department of Medical Laboratory Science (Prof W M Abdel-Rahman PhD), Clinical Sciences Department (Prof E Abu-Gharbieh PhD, H J Barqawi MPhil, N R Dash MD, Prof A A Maghazachi PhD, Prof M M Saber-Ayad PhD, N Saheb Sharif-Askari PhD, Prof I M Talaat PhD), College of Pharmacy (S M Aleidi PhD, H Y Alniss PhD, Prof M H Semreen PhD), Department of Pharmacy Practice and Pharmacotherapeutics (Prof K H Alzoubi PhD, Prof H A Omar PhD), Department of Basic Biomedical Sciences (Prof Y Bustanji PhD), Sharjah Institute for Medical Research (N M Elemam PhD), Department of Basic Medical Sciences (Prof W El-Huneidi PhD), Basic Medical Sciences Department (A B Elmoselhi PhD), Research Institute of Medical & Health Sciences (A B Elmoselhi PhD, Prof M H Semreen PhD), College of Medicine (Prof B A Saddik PhD, Prof M A Saleh PhD), Sharjah Institute of Medical Sciences (F Saheb Sharif-Askari PhD), Department of Medicinal Chemistry (S

S M Soliman PhD), University of Sharjah, Sharjah, United Arab Emirates; Department of Medicine (P Abdi BEng), Memorial University, St. John's, NL, Canada; Institute of Health (W M Abdisa MPH), Jimma University, Dambi Dollo, Ethiopia; Department of Public Health (D Abdissa MSc), Department of Health, Jimma, Ethiopia; Health Department (D Abdissa MSc), Diabetes Research Center, Jimma, Ethiopia; Minimally Invasive Surgery Research Center (A Abdollahi MD, A Kabir MD), School of Medicine (F Afrashteh MD, M Bastan MD), Health Management and Economics Research Center (J Arabloo PhD, H Ayatollahi PhD), Department of Health Information Management (H Ayatollahi PhD), Department of Medical Laboratory Sciences (F Dorostkar PhD), Preventive Medicine and Public Health Research Center (Prof B Eshrati PhD), Department of Medicine (A Farahani MD, M Fotouhi MD, R Rajabi MD), Department of Cardiology (A Ghaffari Jolfayi MD), Department of Ophthalmology (H Hasani MD), Mental Health Research Center (Prof M Hoseinzadeh PhD), Department of Surgery (A Jaliliyan MD), Department of Obstetrics & Gynecology (P Khalili MD), Antimicrobial Resistance Research Center (K Mozahheb Yousefi MD), Hazrat-e-Rasool General Hospital (K Mozahheb Yousefi MD), Physiology Research Center (H Pazoki Toroudi PhD), Department of Physiology (H Pazoki Toroudi PhD), Colorectal Research Center (A Sarveazad PhD), Center for Technology and Innovation in Cardiovascular Informatics (S Shool MD), Iran University of Medical Sciences, Tehran, Iran (H Fattahi PhD); Department of Medicine (Prof M Abdoun PhD), University of Setif Algeria, Sétif, Algeria; Department of Health, Sétif, Algeria (Prof M Abdoun PhD); Faculty of Veterinary Medicine (A Abdous MD), Young Researchers and Elite Club (M Jokar DVM), Islamic Azad University, Karaj, Iran; Department of Physiotherapy (A Abdullahi PhD, A W Awotidebe PhD), Department of Anatomy (L H Adamu PhD), Department of Community Medicine (Prof M A Gadanya MD), Department of Nursing Science (M Ladan PhD), Bayero University Kano, Kano, Nigeria; Department of Physiotherapy (A Abdullahi PhD), Federal University Wukari, Wukari, Nigeria; Department of Midwifery (M Abebe MSc), College of Medicine and Health Sciences (A D Demeke MSc), Dilla University, Dilla, Ethiopia; Department of Emergency Medicine (A Abedi MD), Department of Critical Care and Emergency Nursing (N Hanifi PhD), Zanjan University of Medical Sciences, Zanjan, Iran; Department of Biomedical Sciences (S Abidi PhD), Nazarbayev University School of Medicine, Astana, Kazakhstan; Department of Midwifery (A Abie MSc, B A Alemayehu MSc, W F Balcha MSc, A T Nega MSc, T H Wassie MSc), Department of Nursing (A Y Berhie MSc), Department of Adult Health Nursing (D Endeshaw MSc), Department of Medical Laboratory Science (A Melese MSc), Department of Reproductive Health and Population Studies (E W Mengesha MPH), College of Medicine and Health Sciences (H B Netsere MSc), Bahir Dar University, Bahir Dar, Ethiopia; Department of Community Medicine (Prof O Abiodun MPH), Babcock University, Ilishan-Remo, Nigeria; Department of Family and Community Health (R G Aboagye MPH), Department of Epidemiology and Biostatistics (L A Adzigbli BSc, S A Bosoka MPhil, R K Dowou MPhil), Department of Population and Behavioural Sciences (H Amu PhD), Institute of Health Research (M Immurana PhD), Basic Medical Sciences (R S Maalman PhD), Department of Microbiology and Immunology (Prof V N Orish PhD), Department of Nutrition and Dietetics (L S Tuglo MPH), University of Health and Allied Sciences, Ho, Ghana; School of Population Health (R G Aboagye MPH, Prof B A Saddik PhD), Centre for Social Research in Health (I Y Addo PhD, S R Okeke PhD), Centre for Primary Health Care and Equity (CPHCE) (F Sitas PhD), The George Institute for Global Health (P Ye PhD), University of New South Wales, Sydney, NSW, Australia; Research Center for Immunodeficiencies (H Abolhassani PhD, Prof N Rezaei PhD, A Saghazadeh MD), Experimental Medicine Research Center (A Afrooghe MD), School of Medicine (E Ahmadi MD, A Behnoush BS, H Farrokhpour MD, S Khanmohammadi MD, A Shahbandi MD), Universal Scientific Education and Research Network (USERN) (M Amirzade-Iranaq DDS), Digestive Diseases Research Institute (A Anoushiravani MD, S Fahimi MD, V

Mansouri MD, A Mohamadkhani PhD), Urology Research Center (R Arabzadeh Bahri MD, A Mohammadi MD), Department of Health Information Management (S Ayyoubzadeh PhD), Department of Medicine (A Azimi MD), Department of Epidemiology and Biostatistics (H Azizi PhD, M Mansournia PhD), Non-communicable Diseases Research Center (M Bastan MD, M Rashidi MD, N Rezaei MD, N Rezaei PhD), Department of Scientific Research (F Chichagi MD), Iranian Research Center for HIV/AIDS (IRCHA) (O Dadras PhD), Department of Radiology (R Elahi MD), Pediatric Infectious Disease Research Center (M Farahmand PhD), Department of Ophthalmology (S Ghafarian MD, Prof F Ghassemi MD), Medical School (A Hasanzadeh MD), Sina Trauma and Surgery Research Center (M Hassan Zadeh Tabatabaei MD, M Jalloh MD, Z Ramezani MD, S Shool MD), Department of Virology (A Hosseinzadeh Adli PhD), Department of Immunology (J Karami PhD), Children's Medical Center (Prof F Kompani MD), Urology Department (A Mohammadi MD), Cardiovascular Diseases Research Institute (A Mohsenzadeh MSc), Family Health Research Institute (M Noorafrooz MD), Digestive Diseases Research Center (P Parhizkar Roudsari MD), Cardiac Research Center (P Parhizkar Roudsari MD), Endocrinology and Metabolism Research Institute (N Rezaei PhD), Maternal, Fetal, and Neonatal Research Center (R Sattarpour MD), Sports Medicine Research Center (H Seyedmirzaei MD), Sina Trauma Research Center (M Shabany PhD), Department of Neurology (M Shafie MD), Department of Pathology (Prof S Tavangar MD), Tehran University of Medical Sciences, Tehran, Iran; Department of Medical Biochemistry and Biophysics (H Abolhassani PhD), Department of Neurobiology, Care Sciences and Society, Aging Research Center (B Bizzozero-Peroni PhD), Department of Medicine (T S Hassan PhD), Department of Molecular Medicine and Surgery (Prof J H Kauppila MD), Department of Medical Epidemiology and Biostatistics (MEB) (D Menges PhD), Karolinska Institute, Stockholm, Sweden; Department of Sport, Exercise and Rehabilitation (U S Abonie PhD), Northumbria University, Newcastle, UK; Department of Pediatric Dentistry (Prof L Abreu PhD), Department of Maternal-Child Nursing and Public Health (Prof D C Malta PhD, Prof F P Matozinhos PhD, E J S Prates BS), Escola de Enfermagem da UFMG (Prof T M Ribeiro da Silva PhD), Vaccination Research Observatory (T Rodrigues da Silva PhD), Federal University of Minas Gerais, Belo Horizonte, Brazil; Graduate School of Public Health (A K Abubakar MPH), St. Luke's International University, Tokyo, Japan; Division of Population Data Science (A K Abubakar MPH), National Cancer Center, Tokyo, Japan; Department of Pharmacology and Toxicology (B Abubakar PhD), Department of Pharmaceutics and Pharmaceutical Technology (N Aminu PhD), Department of Veterinary Public Health and Preventive Medicine (A Shittu MSc), Usmanu Danfodiyo University, Sokoto, Sokoto, Nigeria; Department of Biochemistry and Nutrition (K Oyebola PhD), Nigerian Institute of Medical Research, Lagos, Nigeria (B Abubakar PhD); Department of Biopharmaceutics and Clinical Pharmacy (Prof E Abu-Gharbieh PhD), College of Pharmacy (Prof S Aburuz PhD), School of Nursing (Prof M M Ahmad PhD), Department of Radiology and Nuclear Medicine (Prof M E Juweid MD), University of Jordan, Amman, Jordan; Medical Research Center (H J Abukhadijah MPH), Department of Geriatric and Long Term Care (H Al Hamad MD, B Sathian PhD), Rumailah Hospital (H Al Hamad MD), Department of Surgery (A Alansari MD), Surgical Research Section (A R Al-Qudimat MPH, A EL Omri PhD), Nursing & Midwifery Research Department (NMRD) (A J Nashwan PhD), Research Department (K Singh PhD), Hematology Section (Prof M A Yassin MD), Hamad Medical Corporation, Doha, Qatar (J Iqbal PhD); Department of Pharmacology and Therapeutics (Prof S Aburuz PhD), Institute of Public Health (Prof L A Ahmed PhD), Department of Veterinary Medicine (H O Khalifa PhD), College of Medicine and Health Sciences (Prof G Khan PhD, J Nauman PhD), Family Medicine Department (M A Khan MSc), Department of Computer Science and Software Engineering (Prof N Zaki PhD), United Arab Emirates University, Al Ain, United Arab Emirates; Department of Biochemistry and Molecular Medicine (A Abu-Zaid PhD),

Alfaisal University, Riyadh, Saudi Arabia; College of Graduate Health Sciences (A Abu-Zaid PhD), Ophthalmology Department (M Delsoz MD), Department of Ophthalmology (A Nabavi MD), University of Tennessee, Memphis, TN, USA; Department of Biochemistry (R Achar PhD), Jagadguru Sri Shivarathreeswara University, Mysuru, India; Department of Clinical Medicine (Prof J M Acuna MD), American University of Antigua, Coolidge, Antigua and Barbuda; FIU Robert Stempel College of Public Health & Social Work (Prof J M Acuna MD), Florida International University, Miami, FL, USA; Department of Diagnostic and Interventional Radiology (L C Adams PhD), School of Medicine and Health (F Busch MD), Technical University of Munich, Munich, Germany; School of Medicine (J Zhou PhD), Stanford University, Palo Alto, CA, USA (L C Adams PhD); Department of Human Anatomy (L H Adamu PhD, M O Suleiman Odidi PhD), Federal University Dutse, Dutse, Nigeria; School of Medicine (I Y Addo PhD), Faculty of Medicine and Health (W He PhD), Asbestos and Dust Diseases Research Institute (J Leigh MD), NHMRC Clinical Trials Centre (R A Mahumud PhD), Menzies Centre for Health Policy (F Sitas PhD), University of Sydney, Sydney, NSW, Australia (S R Okeke PhD); Department of Immunology, Cancer Science Program (K A Adedokun MSc), Roswell Park Comprehensive Cancer Center, Buffalo, NY, USA; Oral Pathology/Cancer Diagnostics (K A Adedokun MSc), Pediatric Intensive Care Unit (A Al-Eyadhy MD, Prof M Temsah MD), Department of Physiology (Prof S A Meo PhD), University Diabetes Center (A Sultan Meo MPH), Research Chair for Evidence-Based Health Care and Knowledge Translation (Prof M Temsah MD), King Saud University, Riyadh, Saudi Arabia; Translational Research Team (N A Adegoke PhD), Melanoma Institute Australia (N A Adegoke PhD), The University of Sydney, Sydney, NSW, Australia; Department of Obstetrics and Gynecology (V Adekanmbi PhD), Department of Family Medicine (W Lee PhD), University of Texas Medical Branch, Galveston, TX, USA; School of Public Health (I M Adesiyan PhD), Department of Microbiology (I A Anuoluwa PhD, T C Ekundayo PhD), Department of Mathematical and Computer Sciences (O Peter PhD), University of Medical Sciences, Ondo, Ondo, Nigeria; Department of Educational Counselling and Developmental Psychology (H O Adewuyi PhD), Department of Epidemiology and Medical Statistics (A F Fagbamigbe PhD), Institute of Cardiovascular Diseases (O S Folayan MPH), Department of Health Promotion and Education (S Ibitoye PhD), College of Medicine (A P Okekunle PhD), Department of Medicine (Prof M O Owolabi DrM), University of Ibadan, Ibadan, Nigeria; Department of Educational Psychology (H O Adewuyi PhD), Department of Chemical Sciences (V A Obakachi PhD), University of Johannesburg, Johannesburg, South Africa; Apollo Institute of Medical Sciences & Research Chittoor (Prof U Adiga PhD), Apollo Hospital, Chittoor, India; Department of Public Health (Q Adnani PhD), Center of Excellence in Higher Education for Pharmaceutical Care Innovation (Prof M J Postma PhD), Universitas Padjadjaran (Padjadjaran University), Bandung, Indonesia; Department of Health Administration and Education (P O Adoma PhD), University of Education Winneba, Winneba, Ghana; Department of Surgery (A M Afifi MD), University of Toledo, Toledo, OH, USA; Technical Services Directorate (A A Afolabi MPH), MSI Nigeria Reproductive Choices, Abuja, Nigeria; Department of Life Sciences (M S Afzal PhD), University of Management and Technology, Lahore, Pakistan; Department of New Initiatives (Prof S B Agampodi MD), International Vaccine Institute, Seoul, South Korea; MM College of Pharmacy (N Aggarwal PhD), Maharishi Markandeshwar (Deemed to be University), Ambala, India; Department of Health Education and Health Promotion (F D Agide PhD), College of Medicine and Health Sciences Department of Midwifery (H M Halil MSc), Wachemo University, Hossana, Ethiopia; Health Research and Innovation Sciences Center (C Agostinis Sobrinho PhD), Klaipeda University, Klaipeda, Lithuania; SPRINT Sport Physical Activity and Health Research & Innovation Center (C Agostinis Sobrinho PhD), Sport Physical Activity and Health Research & Innovation Center (SPRINT) (Prof L M L R Silva PhD), Polytechnic Institute of Guarda, Guarda, Portugal; Department

of Public Health Sciences (W Agyemang-Duah PhD), Queen's University, Kingston, ON, Canada; School of Public Health (B O Ahinkorah MPhil), School of Life Sciences (G Liu PhD), School of Biomedical Engineering (N Tran MD), University of Technology Sydney, Sydney, NSW, Australia; College of Medicine (A Ahmad PhD, M Tabish MPharm), Shaqra University, Shaqra, Saudi Arabia; School of Medicine and Psychology (D Ahmad PhD), Australian National University, Canberra, ACT, Australia; Public Health Foundation of India, Gandhinagar, India (D Ahmad PhD); Biological Production Unit National Institute of Health Islamabad Pakistan (F Ahmad PhD), National Institute of Health, Islamabad, Pakistan; World Health Organization (F Ahmad PhD), World Health Organisation, Islamabad, Pakistan; Department of Health Informatics (K Ahmad PhD), Qassim University, Buraidha, Saudi Arabia; Department of Health and Biological Sciences (S Ahmad PhD), Abasyn University, Peshawar, Pakistan; Department of Natural Sciences (S Ahmad PhD), Lebanese American University, Beirut, Lebanon; School of Public Health (T Ahmad PhD), Zhejiang University, Hangzhou, China; Department of Neuroscience (A Ahmadzade MD), Department of Medical Genetics and Molecular Medicine (G Khalili-Tanha PhD), Neurosurgery Department (D Kheradmand MD), Biotechnology Research Center (Prof A Sahebkar PhD), Department of Medical Informatics (S Tabatabaei PhD), Applied Biomedical Research Center (S Tabatabaei PhD), Department of Medicine (A Yarahmadi PhD), Mashhad University of Medical Sciences, Mashhad, Iran; Urology Department (M Ahmadzade MD), National Nutrition and Food Technology Research Institute (M Ajami PhD), Taleghani anesthesiologist (F Baghizadeh MD), Internal Medicine Department (H Borhany MD), Department of Community Nutrition (S Doaei PhD), Faculty of Medicine (A Ghaffari Jolfayi MD), Cancer Research Center (M Gholamalizadeh PhD), Obstetrics and Gynecology Department (E Ghotbi MD), Urology and Nephrology Research Center (H Hesami MD), Ophthalmic Research Center (ORC) (H Hesami MD, M Shayan MD), Department of General Medicine (M Karimi MD), Research Institute for Gastroenterology and Liver (S Karimzadhagh MD), Department of Neurosurgery (H Khayat Kashani MD), Social Determinants of Health Research Center (A Kolahi MD, M Rashidi MD), Skull Base Research Center (I Mohammadzadeh MD), Department of Epidemiology (Prof S Sabour PhD), Department of Anesthesiology (S Salimi MD, S Seyed Alshohadaei MD), Shahid Beheshti University of Medical Sciences, Tehran, Iran (M Khayamzadeh MD); Maternal and Child Health Division (A Ahmed MS, M Al-Zubayer MSc, R Banik MS, S Noor MS), International Centre for Diarrhoeal Disease Research, Bangladesh, Dhaka, Bangladesh; Department of Women's and Children's Health (A Ahmed MS), Uppsala University, Uppsala, Sweden; Institute of Endemic Diseases (A Ahmed MSc), Unit of Basic Medical Sciences (E E Siddig MD), University of Khartoum, Khartoum, Sudan; Swiss Tropical and Public Health Institute (A Ahmed MSc), University of Basel, Basel, Switzerland; Medical Laboratory Science Department (G S Ahmed MSc, H M Rahim MSc), University of Human Development, Sulaymaniyah, Iraq; Research and Development Department (I Ahmed PhD), Alpha Genomics Private Limited, Islamabad, Pakistan (Z Basharat PhD); Microbiological Analysis Team (I Ahmed PhD), Korea Research Institute of Standards and Science (KRISS), Daejeon, South Korea; College of Nursing (M S Ahmed MSc), Majmaah University, Al Majmaah, Saudi Arabia; Department of Pathology and Microbiology (M S Ahmed PhD), Basic Sciences Department (B A Zaman PhD), University of Duhok, Duhok, Iraq; College of Medicine and Public Health (M B Ahmed PhD, T G Gebremeskel PhD, B Kaambwa PhD), Health Economics Unit (B Kaambwa PhD), Department of Nursing and Health Sciences (S Shorofi PhD), Flinders University, Adelaide, SA, Australia; Faculty of Public Health (M B Ahmed PhD), Jimma University, Jimma, Ethiopia; Department of Nursing (N Ahmed MSc), Department of Medical Laboratory Sciences (M Arkew MSc), School of Medical Laboratory Sciences (H A Asmerom MSc), Epidemiology ang Biostatistics (F S Bayisa MPH), School of Public Health (A Tekola MPH), Haramaya University, Harar, Ethiopia; Brody School of Medicine (S Ahmed PhD), East

Carolina University, Greenville, NC, USA; Bowen University Hospital (D E Ajala ND), Bowen University, Iwo, Nigeria; Department of Physiology (R E Akhigbe PhD), Ladoke Akintola University, Ogbomoso, Nigeria; Department of Internal Medicine (K Akinosoglou PhD), University of Patras, Patras, Greece; Department of Internal Medicine and Infectious Diseases (K Akinosoglou PhD), University General Hospital of Patras, Patras, Greece; Department of Communicable Diseases (S Al Awaidy MSc), Ministry of Health, Muscat, Oman; Middle East, Eurasia, and Africa Influenza Stakeholders Network, Muscat, Oman (S Al Awaidy MSc); Department of Surgery (S Al Hasan PhD, Prof A T Toriola MD, C Wang PhD), Department of Pathology and Immunology (S Taha MD), Washington University in St. Louis, St. Louis, MO, USA; Fundamentals and Administration Department (Prof O Al Omari PhD), Department of Adult Health and Critical Care (O A M Al Zaabi PhD), Department of Geography (W Ali PhD), Sultan Qaboos University, Muscat, Oman; Al Al-Bayt University, Mafraq, Jordan (Prof M Al Qadire PhD); Faculty of Pharmacy (Y Al Thaher PhD), Faculty of Nursing (M M W Atout PhD), Philadelphia University, Amman, Jordan; School of Pharmacy (Y Al Thaher PhD), Cardiff University, Cardiff, UK; Murdoch Business School (K Alam PhD), Murdoch University, Perth, WA, Australia; School of Health and Environmental Studies (Z Alam PhD), Hamdan Bin Mohammed Smart University, Dubai, United Arab Emirates; Department of Clinical Pharmacy (F Y Al-Ashwal PhD), Al-Ayen Iraqi University, Thi-Qar, Iraq; Department of Clinical Pharmacy and Pharmacy Practice (F Y Al-Ashwal PhD), University of Science and Technology, Sana’a, Yemen, Sana'a, Yemen; Fundamentals and Administration Department (L I Al-Daken PhD), Isra University, Amman, Jordan; Division of Gastroenterology and Hepatology (W A Aldhaleei MD), Mayo Clinic, Jacksonville, FL, USA; School of Pharmacy (S M Aleidi PhD, Prof Y Bustanji PhD), Department of Mathematics (Prof S Momani PhD), The University of Jordan, Amman, Jordan; Department of Bacteriology, Immunology, and Mycology (Prof A M Algammal PhD), Suez Canal University, Ismailia, Egypt; Global Centre for Environmental Remediation (A A S Al-Gheethi PhD), School of Medicine and Public Health (P Atorkey PhD), University of Newcastle, Newcastle, NSW, Australia; Cooperative Research Centre for Contamination Assessment and Remediation of the Environment, Newcastle, NSW, Australia (A A S Al-Gheethi PhD); Department of Health Services and Hospital Administration (M K Al-Hanawi PhD), Health Economics Research Group (M K Al-Hanawi PhD), Department of Community Medicine (R M M Al-Raddadi MD), Department of Family and Community Medicine (Prof N S Butt PhD), Rabigh Faculty of Medicine (Prof A Malik PhD), Department of Dental Public Health (Z S Natto DrPH), King Abdulaziz University, Jeddah, Saudi Arabia; Information Science Department (D Alhuwail PhD), Kuwait University, Sabah Alsalem University City, Kuwait; Health Informatics Unit and Geohealth Lab (D Alhuwail PhD), Dasman Diabetes Institute, Dasman, Kuwait; Department of Zoology (A Ali PhD), Abdul Wali Khan University Mardan, Mardan, Pakistan; Department of Public Health and Primary Care (E Ali MSc), Faculty of Medicine (A Schuermans BSc), Department of Cardiovascular Sciences (A Schuermans BSc, J Van den Eynde BSc), Katholieke Universiteit Leuven, Leuven, Belgium; Department of Pharmacy (M Ali PhD), Mohammed Al-Mana College for Medical Sciences, Dammam, Saudi Arabia; Department of Medical Rehabilitation (Physiotherapy) (M U Ali PhD), College of Medical Sciences (A U Gamawa MSc), Department of Microbiology (M A Isa PhD), University of Maiduguri, Maiduguri, Nigeria; Department of Rehabilitation Sciences (M U Ali PhD), Hong Kong Polytechnic University, Hong Kong, China; Department of Biosciences (R Ali PhD), Centre for Interdisciplinary Research in Basic Sciences (CIRBSc) (S Anwar PhD, T Mohammad PhD, A Shamsi PhD), Jamia Millia Islamia, New Delhi, India; Center for Biotechnology and Microbiology (S S Ali PhD, M Suleman PhD), University of Swat, Swat, Pakistan; Institute of Health and Wellbeing (S M Alif PhD), Federation University Australia, Melbourne, VIC, Australia; School of Public Health and Preventive Medicine (S M Alif PhD, Prof M Asghari-Jafarabadi PhD, P Rai PhD), Department

of Public Health and Preventive Medicine (H Hailu MPH), Monash University, Melbourne, VIC, Australia; Department of Clinical and Community Pharmacy (Prof S W Al-Jabi PhD, Prof S H Zyoud PhD), Department of Physiology, Pharmacology, and Toxicology (Prof R Shawahna PhD), An-Najah National University, Nablus, Palestine; Department of Public Health and Community Medicine (Prof S M Aljunid PhD), International Medical University, Kuala Lumpur, Malaysia; International Centre for Casemix and Clinical Coding (Prof S M Aljunid PhD), National University of Malaysia, Bandar Tun Razak, Malaysia; College of Life Sciences (Prof A Alkhatib PhD), Birmingham City University, Birmingham, UK; Rehabilitation Sciences Department (Prof N A Almasri PhD), Biomedical Research Center, QU Health (M Mohammed PhD), College of Medicine (Prof M A Yassin MD), Qatar University, Doha, Qatar; Department of Parasitology (Prof H M Al-Mekhlafi PhD), Department of Paediatrics (Prof H Ariffin PhD), University of Malaya Medical Centre (Prof H Ariffin PhD), University of Malaya, Kuala Lumpur, Malaysia; Department of Parasitology (Prof H M Al-Mekhlafi PhD), Sana'a University, Sana'a, Yemen; Department of Clinical Nursing (M M Alnaeem PhD), Al-Zaytoonah University of Jordan, Amman, Jordan; School of Public Health (M Alocious Sukumar MPH), SRM Institute of Science and Technology, Chennai, India; Department of Physical Therapy and Rehabilitation Sciences (Prof M A Alomari PhD), Department of Rehabilitation Sciences and Physical Therapy (Prof M A Alomari PhD), Department of Allied Medical Sciences (A Alrawashdeh PhD), Faculty of Medicine (Prof M S I Alyahya PhD, A Alzoubi PhD), Department of Clinical Pharmacy (Prof K H Alzoubi PhD), Department of Public Health (Prof Y S Khader PhD), Jordan University of Science and Technology, Irbid, Jordan; Faculty of Nursing (M R Alosta PhD), Zarqa University, Zarqa, Jordan; Liver, Digestive, and Lifestyle Health Research Section (S A Alqahtani MD), Biostatics, Epidemiology, and Science Computing Department (S Yezli PhD), King Faisal Specialist Hospital & Research Center, Riyadh, Saudi Arabia; Division of Gastroenterology and Hepatology (S A Alqahtani MD), Weill Cornell Medicine, New York, NY, USA; Department of Nursing (I Alrimawi PhD), Department of Medicine (C J Sabet MA), Georgetown University, Washington, DC, USA; Macro-Fiscal Policy Department (S M Alrousan PhD), Ministry of Finance, Dubai, United Arab Emirates; Department of Surgery (S K Al-Sabah MD), Kuwait University, Kuwait, Kuwait; Jaber Al Ahmad Al Sabah Hospital (S K Al-Sabah MD), Ministry of Health, Kuwait, Kuwait; Department of Family and Community Medicine (N Z Alshahrani MD), University of Jeddah, Jeddah, Saudi Arabia; Institute of Molecular Biology and Biotechnology (A Altaf PhD, S Shahid PhD), University Institute of Food Science and Technology (S Bashir PhD), University Institute of Radiological Sciences and Medical Imaging Technology (M Latif PhD), University Institute of Public Health (S Nargus PhD), Research Centre for Health Sciences (RCHS) (S Shahid PhD), The University of Lahore, Lahore, Pakistan; Faculty of Health Sciences (A Altaf PhD), Equator University of Science and Technology, Uganda, Masaka, Uganda; Research, Policy, and Training Directorate (A B Al-Tammemi MPH), Jordan Center for Disease Control, Amman, Jordan; Applied Science Research Center (A B Al-Tammemi MPH), Department of Clinical Nutrition and Dietetics (Prof M E M Faris PhD), Faculty of Nursing (E H Othman PhD), Applied Science Research Centre (R M Zrieq PhD), Applied Science Private University, Amman, Jordan; Department of Pediatrics (E M Alvarez MD), University of California Davis, Sacramento, CA, USA; Research Group in Health Economics (Prof N Alvis-Guzman PhD), Universidad de Cartagena (University of Cartagena), Cartagena, Colombia; Research Group in Hospital Management and Health Policies (Prof N Alvis-Guzman PhD), Universidad de la Costa (University of the Coast), Barranquilla, Colombia; Department of Medical Sciences (Prof Y M Al-Worafi PhD), Azal University for Human Development, Sana'a, Yemen; Department of Clinical Sciences (Prof Y M Al-Worafi PhD), University of Science and Technology of Fujairah, Fujairah, United Arab Emirates; Department of Pediatrics (Prof H Aly MD, A E’mar MD), Lerner Research Institute (Prof X Liu PhD),

Department of Internal Medicine (A Mushtaq MD), Cleveland Clinic, Cleveland, OH, USA; Food and Beverages Safety Research Center (M Aman Mohammadi PhD), Solid Tumor Research Center (H Maleki-Kakelar PhD), Urmia University of Medical Sciences, Urmia, Iran; Public Health and Community Medicine Department (Prof T T Amin MD), Cairo University, Cairo, Egypt; Department of Radiology and Radiological Science (A Amindarolzarbi MD), University of Maryland, Baltimore, MD, USA; Student Research Committee (S Aminnia MD), Department of Nursing (A Azargoonjahromi BSc), Health Human Resources Research Center (M Bayati PhD), Department of Pathology (S Faraji PhD), Family medicine department (M Goodarzian MD), Poostchi Ophthalmology Research Center (M Heydari PhD), Anesthesiology and Critical Care Research Center (F Kanaani Nejad MD), Trauma Research Center (M Karajizadeh PhD), Research center for Neuromodulation and Pain (A Karimi MD), Amir Oncology Hospital (E Meftah MD), Department of Biostatistics (Prof H Molavi Vardanjani PhD), Department of Epidemiology and Biostatistics (H Raeisi Shahraki PhD), Shiraz University of Medical Sciences, Shiraz, Iran; School of Pharmacy (N Aminu PhD), University of Botswana, Gaborone, Botswana; Department of Medicine (G A Amusa MD), Department of Chemical Pathology (L C Imoh MPH), University of Jos, Jos, Nigeria; Department of Internal Medicine (G A Amusa MD), Department of Chemical Pathology (L C Imoh MPH), Jos University Teaching Hospital, Jos, Nigeria; Faculty of Pharmacy (Prof R Ancuceanu PhD), Department of General Medicine (Prof B T Florin PhD, I Negoi PhD, D Serban PhD), Department of Internal Medicine (M Hostiuc PhD), Department of Dermatology (M Tampa PhD), Carol Davila University of Medicine and Pharmacy, Bucharest, Romania; Centre for Sensorimotor Performance (D Anderlini MD), School of Public Health (S Belachew PhD), Department of Urology (Prof E Chung MD), The University of Queensland, Brisbane, QLD, Australia (M Moni PhD); Neurology Department (D Anderlini MD), Royal Brisbane and Women's Hospital, Brisbane, QLD, Australia; Department of Pharmacology (A Anil MD, A Saravanan MD, M Shamim MBBS, S Singh MD, K Tiwari MBBS, S B Varthya MD), Department of Anatomy (N Bhardwaj MD), Department of Community Medicine and Family Medicine (Prof P Bhardwaj MD), School of Public Health (Prof P Bhardwaj MD), Department of Surgical Oncology (Prof S Misra MCh), All India Institute of Medical Sciences, Jodhpur, India; All India Institute of Medical Sciences, Bhubaneswar, India (A Anil MD); Reproductive Immunology Research Center (H Ansariniya PhD), Immunology Department (H Ansariniya PhD), Department of Biostatistics and Epidemiology (M Taheri Soodejani PhD), School of Public Health (Prof M Zare Sakhvidi PhD), Shahid Sadoughi University of Medical Sciences, Yazd, Iran; Regenerative Medicine, Organ Procurement and Transplantation Multi-disciplinary Center (S Anvari MD), School of Health (S Doaei PhD), Gastrointestinal and Liver Disease Research Center (B Eftekhari MD), Department of Social Medicine and Epidemiology (A Feizkhah MD), Gastrointestinal and Liver Diseases Research Center (F Joukar PhD, F Sheida MD), Caspian Digestive Disease Research Center (F Joukar PhD), Clinical Research Development Unit of Poursina Hospital (S Karimzadhagh MD), Department of Medicine (A Khalili MD), Guilan University of Medical Sciences, Rasht, Iran; School of Chemical and Life Sciences (SCLS) (S Anwar PhD), Jamia Hamdard, New Delhi, India; Department of Surgery (S Anwar PhD), Department of Medical Surgical Nursing (A L Wicaksana MS), Gadjah Mada University, Yogyakarta, Indonesia; Department of Pathology (R Anwer PhD), Department of Pharmacology (T Jawaid PhD), Imam Mohammad Ibn Saud Islamic University, Riyadh, Saudi Arabia; Department of Medical Laboratory Sciences (E E Anyabolo BMLS), Department of Pharmacology and Therapeutics (Prof O E Onwujekwe PhD), University of Nigeria Nsukka, Enugu, Nigeria; Operations Department (E E Anyabolo BMLS), Breast Without Spot, Enugu, Nigeria; Rural Health Research Institute (A E Anyasodor PhD), Charles Sturt University, Orange, NSW, Australia; Division of Gastroenterology, Hepatology, and Nutrition (J Arab MD), Division of Infectious Diseases (P R Ching MD),

Virginia Commonwealth University, Richmond, VA, USA; Gastroenterology Department (J Arab MD), Department of Gastroenterology (L Diaz MD), Departamento de Gastroenterologia (Department of Gastroenterology) (F J Idalsoaga MD), Pontifical Catholic University of Chile, Santiago, Chile; Department of Pharmacology and Toxicology (Prof E A Arafa PhD, Prof H A Omar PhD), Beni-Suef University, Beni-Suef, Egypt; College of Pharmacy (M Arafat PhD), Al Ain University, Abu Dhabi, United Arab Emirates; Clinic of Obstetrics and Gynecology (M Arcieri PhD), Santa Maria della Misericordia University Hospital, Udine, Italy; College of Art and Science (D Areda PhD), Ottawa University, Surprise, AZ, USA; School of Life Sciences (D Areda PhD), Arizona State University, Tempe, AZ, USA; Institute for Biomedical Problems (A A Artamonov PhD), Russian Academy of Sciences, Moscow, Russia; Department of Neurological Surgery (S Arul PhD), Department of Neurosurgery (A Orscelik MD, Y Senol MD), Department of Bioengineering and Therapeutical Sciences (Prof M Zastrozhin PhD), University of California San Francisco, San Francisco, CA, USA; Department of Periodontics (D Arumuganainar PhD), Department of Biosciences (S Chopra MPH), Department of Oral Medicine and Periodontology (Prof R D Jayasinghe MS), Saveetha Dental College and Hospitals (G Minervini PhD, Prof D Sekar PhD, M Tovani-Palone PhD), Center for Global Health Research (Prof A Sahebkar PhD, Prof L Thangavelu PhD), Department of Pharmacology (S Sekaran PhD), Saveetha University, Chennai, India; Cabrini Research (Prof M Asghari-Jafarabadi PhD), Cabrini Health, Malvern, VIC, Australia; Department of Physiology (M A B Ashraf PhD), Faisalabad Medical University, Faisalabad, Pakistan; Pioneer Journal of Biostatistics and Medical Research (PJBMR), Pakistan, Pakistan (T Ashraf PhD); Deakin Health Economics/School of Health and Social Development (B K Y Asiamah-Asare PhD), Deakin University, Melbourne, VIC, Australia; Atchabar Scientific Research Institute (B Assembekov PhD), Atchabarov Scientific-Research Institute of Fundamental and Applied Medicine (D Davletov MD, A Zhumagaliuly MD), Director of the Scientific and Technological Park (I R Fakhradiyev PhD), Science and Technology Park (A Ibrayeva PhD), Research and Publication Activity Division (M Kulimbet MSc), Department of Public Health (A Kuttybayev MSc), Department of Research (B Lakanova MD), Science Department (A Shamsutdinova MD), Kazakh National Medical University, Almaty, Kazakhstan; Department of Epidemiology and Biostatistics (S Asthana MD), Indian Council of Medical Research, Noida, India; Discipline of Psychological Science (P Atorkey PhD), ACAP University College, Sydney, NSW, Australia; Management Policy and Community Health (J A Atta MPH), School of Public Health (H Theyra-Enias MD), University of Texas, Houston, TX, USA; School of Business (Prof M Ausloos PhD), Diabetes Research Centre (E Vounzoulaki PhD), University of Leicester, Leicester, UK; Department of Statistics and Econometrics (Prof M Ausloos PhD, Prof C Herteliu PhD), Bucharest University of Economic Studies, Bucharest, Romania; Department of Surgery (A F Awedew MD), Department of Public Health (A T Desale MPH), Debre Tabor University, Debre Tabor, Ethiopia; School of Nursing and Public Health (A W Awotidebe PhD), Pharmaceutical Chemistry (V A Obakachi PhD), Department of Public Health Medicine (S N Shongwe MPH), University of KwaZulu-Natal, Durban, South Africa; The Judith Lumley Centre (B Ayala Quintanilla PhD), Department of Public Health (Prof C Liu PhD), School of Nursing and Midwifery (Prof M Rahman PhD), La Trobe University, Melbourne, VIC, Australia; Universidad de San Martin de Porres, Lima, Peru (B Ayala Quintanilla PhD); Advanced Medical & Dental Institute (M Aziz PhD), Universiti Sains Malaysia, Penang, Malaysia; Department of Anesthesia (S A Aziz PhD), Cihan University -Sulaimaniya, Sulaymaniyah, Iraq; Department of Basic Sciences (S A Aziz PhD), College of Science (F M Rahman PhD), University of Sulaimani, Sulaymaniyah, Iraq (H H R Najmuldeen PhD); Institute of Biotechnology and Genetic Engineering (S Aziz MS), The University of Agriculture, Peshawar, Pakistan; Research Center of Psychiatry and Behavioral Sciences (H Azizi PhD), Immunology Research Center (M Dashti MD), Department of Health Policy and Management (Prof L

Doshmangir PhD), Division of Community Medicine (E Farashi MD), Research Center for Evidence-Based Medicine (M Hosseini MD), Department of Virology (A Hosseinzadeh Adli PhD), Department of Immunology (F Jadidi-Niaragh PhD), School of Management and Medical Informatics (L R Kalankesh PhD), Faculty of Nursing and Midwifery (Prof M Mirghafourvand PhD), Social Determinants of Health Research Center (Prof S Mohammad-Alizadeh-Charandabi PhD), Midwifery Department (Prof S Mohammad-Alizadeh-Charandabi PhD), Health Information Technology (Z Mohammadzadeh PhD), Molecular Medicine Research Center (S Pirouzpanah PhD), Women's Reproductive Health Research Center (R Sattarpour MD), Tabriz University of Medical Sciences, Tabriz, Iran; ASIDE Healthcare, Lewes, DE, USA (A Y Azzam MD); Faculty of Medicine (A Y Azzam MD), The Orthopaedic Department (A M Makram MD), Department of Cardiology (O M Makram MD), October 6 University, 6th of October City, Egypt; Directorate of Quality Assurance (M Badar PhD), Gomal University, Dera Ismail Khan, Pakistan; Department of Forensic Science (A D Badiye PhD, N Kapoor PhD), Government Institute of Forensic Science Nagpur, Nagpur, India; Rashtrasant Tukadoji Maharaj Nagpur University, Nagpur, India (A D Badiye PhD); Department of Clinical Pathology (Prof A A Badran MD, Y M El-Sherbiny PhD), Clinical Pathology Department-Faculty of Medicine (Prof M El Sayed Zaki PhD), Forensic Medicine & Clinical Toxicology Department (Prof S Elkannishy PhD), Faculty of Veterinary Medicine, Department of Physiology (Prof B H Marghani PhD), Faculty of Pharmacy (Prof M A Saleh PhD), Mansoura University, Mansoura, Egypt; Microbiology Department (Prof A A Badran MD), Horus University Egypt, Damietta, Egypt; Health Research Institute (Prof N Bagheri PhD), University of Canberra, Canberra, NSW, Australia; College of Optometry (R Bahreini MS), Pacific University, Forest Grove, OR, USA; Clinical Research Center (R Bai MD), Children's Hospital of Nanjing Medical University, Nanjing, China; International Medical School (A A Baig PhD), Management and Science University, Alam, Malaysia; Surgery, Global Surgery, Emergency Medicine Department (Prof L Bains MD), Maulana Azad Medical College, New Delhi, India; WHO Collaborating Centre for Research in Surgical Care Delivery in LMICs, Mumbai, India (Prof L Bains MD); School of Medicine (R C Bajpai PhD), Keele University, Keele, UK; Department of Forensic Medicine and Toxicology (S M Bakkannavar MD), Department of Pharmacology (S Gangachannaiah MD), Kasturba Medical College, Mangalore (R Holla MD, M Rao MD, Prof B Unnikrishnan MD), Department of Microbiology (P Y Prakash PhD), Kasturba Medical College, Manipal (Prof S Shastry MD), Department of Community Medicine (R S Shetty MD), Department of Physiotherapy (M K Sinha PhD), Kasturba Medical College (D Upadhya PhD), Manipal Academy of Higher Education, Manipal, India; Department of Human Genetics (A A Bala PhD), University of Texas, Brownsville, TX, USA; DEpartmenet of Clinical Pharmacology and Therapeutics (A A Bala PhD), Federal Univeristy Dutse, Dutse, Nigeria; Department of Epidemiology and Biostatistics (M Balooch Hasankhani PhD), Leishmaniasis Research Center (M Faramarzpour MD), Pathology and Stem Cell Research Center (M Faramarzpour MD), Department of Immunology (Prof A Jafarzadeh PhD), Department of Occupational Health and Safety Engineering (R Pourbabaki PhD), Physiology Research Center (M Rajizadeh PhD), Kerman University of Medical Sciences, Kerman, Iran; Nuffield Department of Surgical Sciences (S Bandyopadhyay MPH), University of Oxford, Oxford, UK; Department of Neurosurgery (S Bandyopadhyay MPH), Faculty of Medicine (R Thayakaran PhD), Department of Surgery (G Verras MSc), University of Southampton, Southampton, UK; Clinic for Infectious and Tropical Diseases (A Barac PhD), University Eye Hospital (Prof M M Bozic PhD), Clinical Center of Serbia, Belgrade, Serbia; Faculty of Medicine (A Barac PhD, Prof M M Bozic PhD, I M Ilic PhD, Prof M M Santric-Milicevic PhD), School of Public Health and Health Management (Prof M M Santric-Milicevic PhD), University of Belgrade, Belgrade, Serbia; Department of Anatomy (S Barati PhD), Saveh University of Medical Sciences, Saveh, Iran; Miller School of Medicine (M Bardhan MD), University

of Miami, Miami, FL, USA; School of Psychology (Prof S L Barker-Collo PhD), University of Auckland, Auckland, New Zealand; Department of Public and Environmental Health (A Barrow MPH), University of The Gambia, Banjul, The Gambia; Department of Epidemiology (A Barrow MPH, D Braithwaite PhD), College of Medicine (M J Diaz BS, K T Root BS), Department of Health Services Research, Management and Policy (R Wang MPP), University of Florida, Gainesville, FL, USA; Department of Veterinary Physiology and Biochemistry (A Basiru PhD), Department of Veterinary Public Health and Preventive Medicine (I A Odetokun PhD), Division of Nephrology (Prof T O Olanrewaju MSc), University of Ilorin, Ilorin, Nigeria; Department of Community Medicine (P Baskaran MD), Sri Manakula Vinayagar Medical College and Hospital, Puducherry, Puducherry, India; Department of Medical Education (K Batra PhD), Department of Social and Behavioral Health (Prof M Sharma PhD), University of Nevada Las Vegas, Las Vegas, NV, USA; Department of Health Sciences (DISSAL) (Prof M Bauckneht PhD), University of Genoa, Genova, Italy; Department of Human Anatomy and Histology (Prof N Beeraka PhD), I.M. Sechenov First Moscow State Medical University, Moscow, Russia; Avicenna Biotech Research, Germantown, MD, USA (B Behnam MD); Department of Regulatory Affairs (B Behnam MD), Amarex Clinical Research, Germantown, MD, USA; Endocrinology and Metabolism Research Institute (H Farrokhpour MD), Department of Epidemiology (S Khanmohammadi MD), Non-Communicable Diseases Research Center (NCDRC), Tehran, Iran (A Behnoush BS); Department of Microbiology (P Behzadi PhD), Islamic Azad University, Shahr-e-Qods, Iran; Transplant and Hepatobiliary Surgery Service (D F Bejarano Ramirez MSc), Hospital Universitario Fundación Santa Fe de Bogotá (University Hospital Santa Fe Foundation of Bogotá), Bogota, Colombia; Subdirectorate of Clinical Studies and Clinical Epidemiology (D F Bejarano Ramirez MSc), Hospital Universitorio Fundación Santa Fe de Bogotá, Bogotá, Colombia; Department of Radiology (G Belge Bilgin MD, F Nugen PhD), Department of Nephrology and Hypertension (N Nikravangolsefid MD), Department of Physiology and Biomedical Engineering (F Pourghazi MD), Mayo Clinic, Rochester, MN, USA; Department of Biological Sciences (Prof L Belo PhD), Research Unit on Applied Molecular Biosciences (UCIBIO) (Prof L Belo PhD, V M Costa PhD), Associated Laboratory for Green Chemistry (LAQV) (M Carvalho PhD), Institute for Research and Innovation in Health (i3S) (Prof N Cruz-Martins PhD), Department of Chemical Sciences (A Reis-Mendes PhD), Faculty of Medicine (J R Rocha-Gomes MD), University of Porto, Porto, Portugal; Department of Epidemiology and Health Promotion (Prof H Benzian PhD), New York University, New York, NY, USA; Department of Epidemiology and Biostatistics (A C Bermudez MD), University of the Philippines Manila, Manila, Philippines; Department of Epidemiology (A C Bermudez MD), Brown University, Providence, RI, USA; Department of Community Medicine and Family Medicine (A S Bhadoria MD), All India Institute of Medical Sciences, Rishikesh, India; Community Health Department (A S Bhadoria MD), University of South Wales, South Wales, UK; Department of Public Health (A S Bhagavathula PhD), North Dakota State University, Fargo, ND, USA; Department of Global Pediatric Medicine (N Bhakta MD), Department of Oncology (N Bhakta MD), St. Jude Children's Research Hospital, Memphis, TN, USA; Institute of Applied Health Research (N Bhala PhD), Division of Ophthalmology & Visual Sciences (Prof G D Panos MD), University of Nottingham, Nottingham, UK; Institute of Applied Health Research (N Bhala PhD, K Malhotra MBBS, R Thayakaran PhD), NIHR Global Health Research Unit on Global Surgery (S K Kamarajah MD), Department of Metabolism and Systems Science (S Tariq PhD), University of Birmingham, Birmingham, UK; Department of Medicine (R Bharadwaj PhD), Radiology Department (S Madani MD), Department of Neurology (B S Srichawla MD), University of Massachusetts Medical School, Worcester, MA, USA; Division of Hematology Oncology (P V Bhardwaj MD), University of Massachusetts Medical School, Springfield, MA, USA; Global Health Neurology Lab (S Bhaskar MD), NSW Brain Clot Bank, Sydney, NSW, Australia;

Division of Cerebrovascular Medicine and Neurology (S Bhaskar MD), National Cerebral and Cardiovascular Center, Suita, Japan; Department of General Medicine (A N Bhat MD), Department of Internal Medicine (A Boloor MD), Department of Community Medicine (N Joseph MD, N Kumar MD, P Mithra MD, R Motappa MD, R Thapar MD), Department of Forensic Medicine and Toxicology (Prof J Padubidri MD, P H Shetty MD), Manipal College of Dental Sciences, Mangalore (Prof P K Shetty MDS), Manipal Academy of Higher Education, Mangalore, India; Department of Medicine (V Bhat MBBS), SUNY Upstate Medical University, Syracuse, NY, USA; School of Sport & Health Sciences (S Bhattacharjee MPH), University of Brighton, Brighton, UK; Department of Public Health Research (S Bhattacharjee MPH), Bengal Rural Welfare Service (BRWS), Kolkata, India; Department of Medical Lab Technology (Prof G K Bhatti PhD), Chandigarh University, Mohali, India; Department of Human Genetics and Molecular Medicine (Prof J Bhatti PhD, U Sharma PhD), Department of Microbiology (P K Kushawaha PhD, A Singh PhD, M Yadav PhD), Department of Biochemistry (B Singh PhD), Department of Computer Science & Engineering (Prof S Singh PhD), Department of Zoology (B Vellingiri PhD), Central University of Punjab, Bathinda, India; Department of Pharmacy (Prof M A Bhuiyan PhD), University of Asia Pacific, Dhaka, Bangladesh; Department of Health Administration (S S Bhuyan PhD), Institute for Health, Health Care Policy and Aging Research (S Rege PhD), Rutgers University, New Brunswick, NJ, USA; Fondazione Banca Degli Occhi Del Veneto (R Bievel-Radulescu MD), Carol Davila University of Medicine and Pharmacy, Venice, Italy; Department of Radiology (C Bilgin MD), Neurovascular Research Laboratory (C Bilgin MD), Mayo Clinic College of Medicine, Rochester, MN, USA; Department of Biochemistry and Biotechnology (M Biswas PhD), University of Science and Technology Chittagong, Chittagong, Bangladesh; Centre for the Study of Regional Development (M Biswas PhD), Jawahar Lal Nehru University, New Delhi, India; Higher Institute of Physical Education, Department of Physical Education and Health (B Bizzozero-Peroni PhD), Universidad de la República, Rivera, Uruguay; Department of Global Public Health and Primary Care (Prof T Bjørge PhD), University of Bergen, Bergen, Norway; Department of Research (M W Wojewodzic PhD), Cancer Registry of Norway, Oslo, Norway (Prof T Bjørge PhD); Department of Community Medicine and Family Medicine (Prof T Bodhare MD), All India Institute of Medical Sciences, Ramanathapuram, India; Department of Demography and Population Studies (O A Bolarinwa MSc), University of Witwatersrand, Johannesburg, South Africa; Disease Surveillance Department (S A Bosoka MPhil), Ghana Health Service, Ho, Ghana; Facultad de Salud (Faculty of Health) (Prof A Botero Carvajal PhD), Universidad Santiago de Cali (Santiago de Cali University), Cali, Colombia; Department of Medicine (Prof S Bouaoud DrPH), Faculty of Medicine (Prof A Ouyahia PhD), University Ferhat Abbas of Setif, Sétif, Algeria; Department of Epidemiology and Preventive Medicine (Prof S Bouaoud DrPH), University Hospital Saadna Abdenour, Sétif, Algeria; Cancer Population Sciences Program (D Braithwaite PhD), University of Florida Health Cancer Center, Gainesville, FL, USA; School of Population and Public Health (Prof M Brauer DSc), University of British Columbia, Vancouver, BC, Canada; Division of Clinical Epidemiology and Aging Research (Prof H Brenner MD), German Cancer Research Center, Heidelberg, Germany; College of Health Sciences (L P Bui PhD, T T Pham PhD), VinUniversity, Hanoi, Viet Nam; Research Advancement Consortium in Health, Hanoi, Viet Nam (L P Bui PhD, T T Pham PhD); Department of Radiology (F Busch MD), Department of Public Health and Primary Care (Prof P Willeit PhD), University of Cambridge, Cambridge, UK; Department of Public Health (Prof M T Bustamante-Teixeira PhD, Prof M R Ribeiro Guerra PhD), Federal University of Juiz de Fora, Juiz de Fora, Brazil; School of Public Health Sciences (Z A Butt PhD, O A Zitoun MD), Department of Geography and Environmental Management (C Dassah MSc), University of Waterloo, Waterloo, ON, Canada; Al Shifa School of Public Health (Z A Butt PhD), Al Shifa Trust Eye Hospital, Rawalpindi, Pakistan; Department of Gynecologic

Oncology (G Cai MD), Guangdong Provincial Clinical Research Center for Obstetrical and Gynecological Diseases (G Cai MD), Department of Breast Surgery (Prof K Chen MD), The First Affiliated Hospital (G He PhD), Sun Yat-sen University Cancer Center (J Hu Mmed), Department of Urology (J Kong MD), The Department of Gynecology (S Qiu MD), Sun Yat-sen University, Guangzhou, China; Department of Internal and Geriatric Medicine (Prof L A Cámera MD), Hospital Italiano de Buenos Aires (Italian Hospital of Buenos Aires), Buenos Aires, Argentina; Board of Directors (Prof L A Cámera MD), Argentine Society of Medicine, Buenos Aires, Argentina; Center for Nutrition and Health Research (I Campos-Nonato PhD, E Denova-Gutiérrez DSc), National Institute of Public Health, Cuernavaca, Mexico; Department of Anesthesiology (S Cao MD), Third Xiangya Hospital of Central South University, Changsha, China; Department of Oral and Maxillofacial Surgery (Y Cao PhD), Department of Neurosurgery (Prof Q He MD), State Key Laboratory of Oral Diseases & National Center for Stomatology (L Liu DDS), Sichuan University, Chengdu, China; Unit of Hygiene and Public Health (A Capodici MD), Romagna Local Health Authority, Forlì-Cesena, Italy; Interdisciplinary Research Center for Health Science (A Capodici MD), Sant'Anna School of Advanced Studies, Pisa, Italy; Oncological Network, Prevention and Research Institute (G Gorini MD), Institute for Cancer Research, Prevention and Clinical Network, Florence, Italy (G Carreras PhD); Faculty of Health Sciences (M Carvalho PhD), University Fernando Pessoa, Porto, Portugal; Public Health Department (C A Castañeda-Orjuela PhD), Department of Public Health (Prof F P De la Hoz PhD), National University of Colombia, Bogota, Colombia; Epidemiology and Public Health Evaluation Group (C A Castañeda-Orjuela PhD), National University of Colombia, Bogotá, Colombia; Institute of Public Goods and Policies (F Catalá-López PhD), Spanish National Research Council, Madrid, Spain; Centre for Biomedical Research in Mental Health Network (CIBERSAM) (F Catalá-López PhD), National School of Public Health (A Padron-Monedero PhD), Institute of Health Carlos III, Madrid, Spain; Department of Public Health and Infectious Diseases (M S Cattaruzza PhD), La Sapienza University, Rome, Italy; Department of Medical, Surgical, and Health Sciences (Prof L Cegolon PhD), University of Trieste, Trieste, Italy; Public Health Unit (Prof L Cegolon PhD), University Health Agency Giuliano-Isontina (ASUGI), Trieste, Italy; Department of Nutrition (Prof F Cembranel DSc), Federal University of Santa Catarina, Florianópolis, Brazil; Mary MacKillop Institute for Health Research (Prof E Cerin PhD), Australian Catholic University, Melbourne, VIC, Australia; School of Public Health (Prof E Cerin PhD), Department of Medicine (H Chou MSc), University of Hong Kong, Hong Kong, China; Regional Epidemiological Observatory Department (A Cernigliaro MSc), Sicilian Regional Health Authority, Palermo, Italy; Institute of Clinical Physiology (S Cerrai MSc), Italian National Council of Research, Pisa, Italy; Joint School of Public Health (A Chakraborty PhD), Old Dominion University, Norfolk, VA, USA; Department of Biotechnology (Prof C Chakraborty PhD), Adamas University, Kolkata, India; Institute for Skeletal Aging & Orthopedic Surgery (Prof C Chakraborty PhD), Hallym University, Chuncheon, South Korea; State Disease Investigation Laboratory (S Chakraborty MVSc), Animal Resources Development Department, Agartala, India; Clinical Nutrition Department (R M Chandika PhD), Health Research Center (Prof A Khalid PhD), Department of Public Health (A Mehmood PhD, F Sobia PhD), Health Education & Promotion, College of Nursing and Health Sciences (M Shanawaz MD), Jazan University, Jazan, Saudi Arabia; Temerty Faculty of Medicine (V Chattu MD), University of Toronto, Toronto, ON, Canada; Department of Community Medicine (V Chattu MD), Datta Meghe Institute of Medical Sciences, Sawangi, India; Department of Biology (A A Chaudhary PhD), Al-Imam Mohammad Ibn Saud Islamic University, Riyadh, Saudi Arabia; Department of Public Health (S Chaudhuri MD), Indian Institute of Public Health, Hyderabad, India; Department of Oral Medicine and Radiology (Prof A Chaurasia MD), Department of Oral Pathology and Microbiology (A Jain MDS), Department of Neurology (V Suresh MBBS), King George's Medical

University, Lucknow, India; EPI (G B Chemeda MPH), Oromia Health Bureau, Addis Ababa, Ethiopia; Faculty of Humanities and Health Sciences (H Chen MSc), Curtin University, Miri, Malaysia; School of Dentistry (M Chen DDS), University of Michigan, Ann Arbor, MI, USA; Department of Public Health and Health Policy (O Chimed-Ochir PhD, A Fukunaga PhD), Hiroshima University, Hiroshima, Japan; Department of Public Health, Administration, and Social Sciences (J L Chirinos-Caceres DrPH), Cayetano Heredia University, Lima, Peru; Department of Clinical Oncology (W C S Cho PhD), Queen Elizabeth Hospital, Hong Kong, China; Cancer Big Data Center (D Choi PhD), National Cancer Center, Goyang, South Korea; College of Medicine (S Choi MD), Department of Epidemiology and Health Promotion (Prof S Jee PhD), Department of Medicine (Y Kim), Department of Preventive Medicine (Prof E Park PhD), Institute of Health Services Research (Prof E Park PhD), Yonsei University, Seoul, South Korea; Department of Medicine (B Chong MBBS), Department of Surgery (K Tan PhD), National University of Singapore, Singapore, Singapore; The Nethersole School of Nursing (Y Chong PhD), Faculty of Medicine (J Huang MD), Jockey Club School of Public Health and Primary Care (C Zhong PhD), The Chinese University of Hong Kong, Hong Kong, China; Centre for Research Impact & Outcome (H Chopra PhD), Centre for Research Impact and Outcome (A M Darwesh PhD), Chitkara University, Rajpura, India; Department of Community Medicine (Prof S G Choudhari MD), Jawaharlal Nehru Medical College, Wardha, India; The Interdisciplinary Research Group on Biomedicine and Health (D Chu PhD), Faculty of Applied Sciences (D Chu PhD), VNU International School (VNUIS), Hanoi, Viet Nam; Department of Paediatric Surgery (I S Chukwu BMedSc), Federal Medical Centre, Umuahia, Nigeria; Department of AndroUrology (Prof E Chung MD), AndroUrology Centre, Brisbane, QLD, Australia; Department of Health Behavior (S Chung MPH), Department of Biomedical Engineering (M Negahdary PhD), Center for Remote Health Technologies & Systems (M Negahdary PhD), Texas A&M University, College Station, TX, USA; Department of Food, Environmental and Nutritional Sciences (I Cioffi PhD, M Marino PhD, Prof S Perna PhD), Department of Clinical Sciences and Community Health (Prof C La Vecchia MD), Department of Biomedical Sciences for Health (S Villa MD), University of Milan, Milano, Italy; Department of Biostatistics (A Columbus MS), Russell H. Morgan Department of Radiology and Radiological Science (A Kamireddy MD), Department of Neurosurgery (F Kazemi MD), Global Surgical Initiative, Department of Surgery (Y Mogessie MD), Johns Hopkins University, Baltimore, MD, USA; Nova Medical School (Prof J Conde PhD), Nova University of Lisbon, Lisbon, Portugal; Life and Health Sciences Research Institute (ICVS) (Prof N Cruz-Martins PhD), University of Minho, Braga, Portugal; Department of Epidemiology and Biostatistics (B Dabo MSPH), Department of Medical Engineering (D Nguyen BS), University of South Florida, Tampa, FL, USA; Department of Medical Microbiology and Parasitology (B Dabo MSPH), Department of Pharmaceutics and Pharmaceutical Technology (S Ilyasu PhD), Bayero University, Kano, Nigeria; Research Center for Child Psychiatry (O Dadras PhD), Heart Center (V Kytö MD), University of Turku, Turku, Finland; Public Health Foundation of India, Gurugram, India (Prof L Dandona MD, Prof R Dandona PhD, G Kumar PhD); Department of Brain Sciences (L D'Anna PhD), WHO Collaborating Centre for Public Health Education and Training (Q Lin MPH), Department of Primary Care and Public Health (Prof A Majeed MD, Prof S Rawaf MD), School of Public Health (A M Makram MD), Department of Surgery and Cancer (Prof E Mossialos PhD, C Rao PhD), The George Institute for Global Health (Prof S Yaya PhD), Imperial College London, London, UK; Department of Internal Medicine (P Danpanichkul MD), Texas Tech University, Lubbock, TX, USA; Department of Environmental Health (R Darvishi Cheshmeh Soltani PhD), Arak University of Medical Sciences, Arak, Iran; Institute of Research and Development (A M Darwesh PhD, Prof M Hoseinzadeh PhD), Duy Tan University, Da Nang, Viet Nam; Department of Biochemistry (S Das MD), Ministry of Health and Welfare, New Delhi, India; Department

of Mathematics (F E Dean BA), School of Information (F Nugen PhD), University of California Berkeley, Berkeley, CA, USA; Memorial Sloan Kettering Cancer Center (E Dee MD), Memorial Sloan Kettering Cancer Center, New York, NY, USA; Department of Pediatrics (S Deekonda MD), Brookdale University Hospital Medical Center, Brooklyn, NY, USA; Division of Cardiology (R Desai MBBS), Atlanta Veterans Affairs Medical Center, Decatur, GA, USA; Biochemistry Department (G Dessie MSc), College of Medicine and Health Sciences (G W Geremew MSc), School of Nursing (H B Netsere MSc), Department of Clinical Pharmacy (A K Sendekie MSc), University of Gondar, Gondar, Ethiopia; JSS Medical College Department of Biochemistry (D Devegowda PhD), Jagadguru Sri Shivarathreeswara Academy of Health Education and Research, Mysuru, India; Department of Pharmacy (S Dewan PhD), United International University, Dhaka, Bangladesh; Pharmacology Division (S Dewan PhD), Center for Life Sciences Research Bangladesh, Dhaka, Bangladesh; Sheffield Teaching Hospitals NHS Foundation Trust, Sheffield, UK (A Dhali MBBS); Research and Development Cell (A S Dhane MBA), Department of Oral Pathology and Microbiology (Prof G S Sarode PhD, Prof S C Sarode PhD), Dr. D. Y. Patil Vidyapeeth, Pune (Deemed to be University), Pune, India; Research Department (M Dhimal PhD), Nepal Health Research Council, Kathmandu, Nepal; Institute of Occupational, Social and Environmental Medicine (M Dhimal PhD), Goethe University, Frankfurt am Main, Germany; Department of Pharmacy Practice (S Dhingra PhD), National Institute of Pharmaceutical Education and Research Hajipur, Hajipur, India; Population Interventions Unit (B Dhungel DrPH), School of Health Sciences (A Meretoja MD), Melbourne School of Population & Global Health (Y Si PhD), University of Melbourne, Melbourne, VIC, Australia; Department of Life Science and Public Health (M Di Pumpo DrPH), Università Cattolica del Sacro Cuore (Catholic University of the Sacred Heart), Rome, Italy; University of California San Diego, La Jolla, CA, USA (L Diaz MD); Department of Medicine (T H P Do MD), Can Tho University of Medicine and Pharmacy, Can Tho, Viet Nam; Nuclear Medicine Deparment (F Dondi MD), ASST Spedali Civili di Brescia and Università degli Studi di Brescia, Brescia, Italy; Departamento de Responsabilidade Social (Department of Social Responsibility) (W M dos Santos PhD), Oswaldo Cruz German Hospital, São Paulo, Brazil; Brazilian Centre for Evidence-based Healthcare (W M dos Santos PhD), Joanna Briggs Institute, São Paulo, Brazil; Independent Consultant, Bridgewater, NJ, USA (O P Doshi MS); Department of Maternal and Child Health (T O Dosumu MSc), Department of Nursing Science (M I Olatubi PhD), Bowen University Iwo, Iwo, Nigeria; Department of Epidemiology (M Dresse MD), University of Pittsburgh, Pittsburgh, PA, USA; Department of Psychiatry (M Dresse MD), Department of Medicine (P Habibzadeh MD), University of Pittsburgh Medical Center, Pittsburgh, PA, USA; Office of Institutional Analysis (J Dube MA), University of Windsor, Windsor, ON, Canada; Faculty of Science and Humanities (S Duraisamy PhD), SRM Institute of Science and Technology, Kattankulathur, India; Department of Infection and Tropical Medicine (O C Durojaiye MPH), Sheffield Centre for Health and Related Research (J O Oguta MSc), University of Sheffield, Sheffield, UK; Department of Conservative Dentistry with Endodontics (A M Dziedzic DSc), Medical University of Silesia, Katowice, Poland; School of Health Sciences (H A Edinur PhD), Universiti Sains Malaysia (University of Science Malaysia), Kubang Kerian, Malaysia; Isenberg School of Management (A Eighaei Sedeh MD), University of Massachusetts Amherst, Amherst, MA, USA; Department of Radiology (X Liu PhD), Department of Medicine (T J Roberts MD, D X Zheng MD), Massachusetts General Hospital, Boston, MA, USA (A Eighaei Sedeh MD); Faculty of Science and Health (M Ekholuenetale PhD), University of Portsmouth, Hampshire, UK; Almoosa College of Health Sciences, Al Ahsa, Saudi Arabia (R A El Arab PhD); College of Medicine (M Elhadi MD), Department of Medicine (I R Fakhradiyev PhD), Korea University, Seoul, South Korea; Houston Methodist Hospital, Houston, TX, USA (M Elhadi MD); Department of Radiation Oncology (A O Elmehrath MD), The University of Texas MD

Anderson Cancer Center, Houston, TX, USA; Deanery of Biomedical Sciences (R Elsheikh MD), College of Medicine and Veterinary Medicine (G Verras MSc), University of Edinburgh, Edinburgh, UK; Department of Biosciences (Y M El-Sherbiny PhD), Nottingham Trent University, Nottingham, UK; Department of Animal Medicine (I Elsohaby PhD), Department of Pathology (Prof M M M Metwally PhD), Department of Microbiology and Immunology (G Yahya PhD), Zagazig University, Zagazig, Egypt (Prof M I Husseiny PhD); Faculty of Veterinary Medicine (Prof A S A Eltahawy PhD), Damanhour University, Damanhur, Egypt; Department of Research (S Emdadul Haque PhD), UChicago Research Bangladesh, Dhaka, Bangladesh; Department of Public Health and Tropical Medicine (T I Emeto PhD), James Cook University, Townsville, QLD, Australia; Department of Pharmacology and Toxicology (T Emran DSc), University of Louisville, Louisville, KY, USA; Department of Bacteriology and Virology (M Eslami PhD), Cancer Research Center (M Eslami PhD, D Haghmorad PhD), Department of Immunology (D Haghmorad PhD), Semnan University of Medical Sciences, Semnan, Iran; Department of Virology (S Ezzikouri PhD), Pasteur Institute of Morocco, Casablanca, Morocco; Department of Electrical and Computer Engineering (H Fadavian MSc), Tarbiat Modares University, Tehran, Iran; Research Centre for Healthcare and Community (A F Fagbamigbe PhD), Coventry University, Coventry, UK; Department of Oral Biology (A Fahim PhD), Riphah International University, Islamabad, Pakistan (Z Z Piracha PhD); Key Laboratory of Computer-Aided Drug Design (A Fahira PhD, M Waqas PhD), Dongguan Key Laboratory of Computer-Aided Drug Design (M Shahab PhD), Guangdong Medical University, Dongguan, China; Department of Food Hygiene and Quality Control (A Fakhri-Demeshghieh DVM), University of Tehran, Tehran, Iran; Department of Biomedical and Biotechnological Sciences (L Falzone PhD), Department of Clinical and Experimental Medicine (Prof C Ledda PhD), Department of Medical and Surgical Sciences and Advanced Technologies "GF Ingrassia" (Prof M Veroux PhD), University of Catania, Catania, Italy; Epidemiology and Biostatistics Unit (L Falzone PhD), IRCCS Pascale, Naples, Italy; Division of Statistics (Z Farhana MS), Bangladesh Bank, Sylhet, Bangladesh; Department of Biology (Prof P S Faris PhD, K A Mohammad PhD), Research Centre (T S Hassan PhD), Department of Chemistry (H I M Amin PhD), Salahaddin University-Erbil, Erbil, Iraq; Department of Biology (Prof P S Faris PhD), Department of Business Administrations (Prof D H Kadir PhD), Department of Medical Biochemical Analysis (H I M Amin PhD), Cihan University-Erbil, Erbil, Iraq; Department of Psychology (A Faro PhD), Federal University of Sergipe, São Cristóvão, Brazil; Department of Family Medicine (U Farooque MD), Luton & Dunstable University Hospital, Luton, UK; Satcher Health Leadership Institute (A O Fasanmi PhD), Morehouse School of Medicine, Atlanta, GA, USA; School of Medicine (A O Fasanmi PhD), Department of Pediatrics (I L Matthew MD), Department of Cardiothoracic Imaging (A Siddiqi MD), Emory University, Atlanta, GA, USA; Department of Medicinal Chemistry (K Fasihi PharmD), Hamadan University of Medical Sciences, Hamadan, Iran; Department of Chemistry and Biochemistry (E T Fasusi MSc), University System of Georgia, Statesboro, GA, USA; Pharmacy Department (E T Fasusi MSc), Department of Community Medicine (O S Ilesanmi PhD), Department of Medicine (Prof M O Owolabi DrM), University College Hospital, Ibadan, Ibadan, Nigeria; Centre for Primary Health Care Network Management (H Fattahi PhD), National Agency for Strategic Research in Medical Sciences Education (M Heydari PhD), Ministry of Health and Medical Education, Tehran, Iran; Department of Pharmacy (G Fekadu PhD), Department of Nursing (G Fetensa MSc), Department of Public Health (D R Terefa MSc), Wollega University, Nekemte, Ethiopia; Department of Translational Medicine (D Ferrante PhD), University of Piemonte Orientale, Italy, Novara, Italy; Center for Public Health Research (P Ferrara PhD), School of Medicine and Surgery (Prof L G Mantovani DSc), University of Milan Bicocca, Monza, Italy; Laboratory of Public Health (P Ferrara PhD, Prof L G Mantovani DSc), IRCCS Istituto Auxologico Italiano, Milan, Italy; Department of Social Sciences (Prof N Ferreira PhD), University

of Nicosia, Nicosia, Cyprus; Institute of Public Health (F Fischer PhD), Charité Universitätsmedizin Berlin (Charité Medical University Berlin), Berlin, Germany; James Cancer Hospital at The Ohio State University (J L Fisher PhD), Ohio State University, Columbus, OH, USA; Department of Neurology, Public Health and Disability (A Fornari PhD), Fondazione IRCCS Istituto Neurologico Carlo Besta, Milano, Italy; Department of Ophthalmology (A Forouhari MD), Department of Clinical Biochemistry (A Mafi PhD, O Vakili PhD), Nutrition and Food Security Research Center (A Mafi PhD), Department of Epidemiology and Biostatistics (Prof M Mansourian PhD), Department of Medical Physics (K Saber PhD), Poursina Hakim Digestive Diseases Research Center (P Samadi PhD), Isfahan University of Medical Sciences, Isfahan, Iran; Department of Pharmacology (Prof B Foroutan PhD), Iranshahr University of Medical Sciences, Iranshahr, Iran; Department of Radiology (M Fotouhi MD), University of Southern California, Los Angeles, CA, USA; Department of Dermatology (Prof T Fukumoto PhD), Kyoto Prefectural University of Medicine, Kyoto, Japan; Department of Community Medicine (Prof M A Gadanya MD), Aminu Kano Teaching Hospital, Kano, Nigeria; Department of Oral Biology and Experimental Dental Research (M Gajdács PhD), University of Szeged, Szeged, Hungary; Department of Medical Epidemiology (S Gallus PhD), Mario Negri Institute for Pharmacological Research, Milan, Italy; Department of Community Medicine and Family Medicine (A P Gandhi MD), All India Institute of Medical Sciences, Nagpur, India; Department of Hematology/Oncology (M Gangireddy MD), MedStar Health, Baltimore, MD, USA; Infectious Diseases Unit (J Garlasco MD), University of Verona, Verona, Italy; Department of Pharmacology (Prof R K Gautam PhD), IES Institute of Pharmacy, Bhopal, India; College of health sciences (F S Gebre MD), Addis Ababa University, Addis Ababa, Ethiopia; Department of Midwifery (M W Gebregergis MSc), School of Public Health (H G Gebreslassie MPH), Department of Medical Laboratory Sciences (H N Meles MSc), Adigrat University, Adigrat, Ethiopia; Environmental Pollution Monitoring and Study Desk (M Gebrehiwot DSc), Ethiopian Environmental Protection Authority, Addis Ababa, Ethiopia; College of Medicine and Public Health (T G Gebremeskel PhD), Department of Nursing (A A Girmay MSc), Aksum University, Aksum, Ethiopia; Department of Public Health (G K Getahun MPH), Menelik II Medical and Health Science College, Addis Ababa, Ethiopia; Biopharmaceutics and Clinical Pharmacy Department (L F Gharaibeh PhD), Al-Ahliyya Amman University, Amman, Jordan; School of Medicine (H Ghasrsaz MD), Mazandaran University of Medical Sciences, Mazandaran, Iran; Family and Community Medicine Department (R M Ghazy PhD), King Khalid University, Abha, Saudi Arabia; Department of Dermatology (N Gholizadeh MD), Department of Biostatistics (Prof A Khalilian PhD), Department of Immunology (Prof A Rafiei PhD), Molecular and Cell Biology Research Center (Prof A Rafiei PhD), Department of Medical-Surgical Nursing (S Shorofi PhD), Department of Environmental Health (Prof Z Yousefi PhD), Mazandaran University of Medical Sciences, Sari, Iran; GBD Collaborating Unit (K Giannakis, Prof S E Vollset DrPH), Norwegian Institute of Public Health, Bergen, Norway; Country Office (A U Gil PhD), World Health Organization (WHO), Astana, Kazakhstan; Department of Biological Sciences and Chemistry (S A Gilani PhD, Prof J Hussain PhD), Natural and Medical Sciences Research Center (A Khan PhD, S A Shahid MPhil, A Ullah MS), University of Nizwa, Nizwa, Oman; Department of Anesthesiology and Critical Care Medicine (A Girombelli MD), Ospedale SS Annunziata Savigliano, Savigliano, Italy; Department of Hepatology (Prof A Goel DM), Sanjay Gandhi Postgraduate Institute of Medical Sciences, Lucknow, India; Department of Radiation Oncology (Prof A K Goel MD), All India Institute of Medical Sciences, Bathinda, Punjab, India; Department of Health Systems and Policy Research (Prof M Golechha PhD), Indian Institute of Public Health, Gandhinagar, India; Department of Life Sciences, Health and Healthcare Professions (Prof D Golinelli MD), Link Campus University, Rome, Italy; Health Services Research, Evaluation and Policy Unit (Prof D Golinelli MD), AUSL della Romagna, Ravenna, Italy; Department of

Gastroenterology (C Gu MD), Cangnan Hospital of Traditional Chinese Medicine, Wenzhou, China; Department of Epidemiology and Biostatistics (S Guan MD), Department of Urology (C Mao MSc), Anhui Medical University, Hefei, China; Health Direction (G Guarducci MD), Local Health Authority of Ferrara, Ferrara, Italy; Department of Clinical Science (M I M Gubari PhD), University of Sulaimani, Sulaimani, Iraq; Divison of Epidemology (Z Guo PhD), Vanderbilt University Medical Center, Nashville, TN, USA; Group Health Department (Z Guo MPH), Nanyang Central Hospital, Nanyang, China; Department of Public Health (B Gupta PhD), Torrens University Australia, Melbourne, VIC, Australia; Department of Toxicology (S Gupta PhD), Shriram Institute for Industrial Research, Delhi, India; Department of Epidemiology and Psychosocial Research (R A Gutiérrez PhD), Ramón de la Fuente Muñiz National Institute of Psychiatry, Mexico City, Mexico; Research Unit (J Guzman-Esquivel PhD), Mexican Institute of Social Security, Colima, Mexico; Neurosurgery Department (A Habibzadeh MD), Fasa University of Medical Sciences, Shiraz, Iran; Global Virus Network, Middle East Region, Shiraz, Iran (F Habibzadeh MD); Department of Midwifery (T S Hadaro MSc), Department of Public Health (T Mekene Meto MPH), Arba Minch University, Arba Minch, Ethiopia; Department of Clinical Pharmacology and Medicine (Prof N R Hadi PhD), University of Kufa, Najaf, Iraq; Department of Liver Tumor, Cancer Center (N Hai Nam PhD), Liver Transplant Unit (N Hai Nam PhD), Cho Ray Hospital, Ho Chi Minh City, Viet Nam; Department of Pharmacy (Prof I M Hamad PhD), American University of Madaba, Amman, Jordan; Department of Family and Community Medicine (Prof R R Hamadeh PhD), College of Medicine and Health Sciences (H Jahrami PhD), Arabian Gulf University, Manama, Bahrain; Biochemistry Department (Prof N M Hamdy PhD), Department of Entomology (A M Samy PhD), Medical Ain Shams Research Institute (MASRI) (A M Samy PhD), Ain Shams University, Cairo, Egypt; Department of Public Health (S Hameed PhD), Green International University, Lahore, Lahore, Pakistan; Department of Chemistry (A Haque PhD), Department of Biochemistry, College of Medicine (M A Kausar PhD), Department of Biochemistry (Prof M Kuddus PhD), Department of Biology (Prof M Saeed PhD), Department of Public Health (M G M Zeariya PhD), Department of Medical-Surgical Nursing (R M Zrieq PhD), University of Hail, Hail, Saudi Arabia; Research Unit (J M Haro MD), Parc Sanitari Sant Joan de Deu, Barcelona, Spain; Department of Mental Health (J M Haro MD), Carlos III Health Institute (Prof R Tabarés-Seisdedos PhD), Biomedical Research Networking Center for Mental Health Network (CiberSAM), Madrid, Spain; Department of Zoology and Entomology (A I Hasaballah PhD, M G M Zeariya PhD), Al-Azhar University, Cairo, Egypt; Department of Health Research Methods, Evidence, and Impact (M Hasan MPH), Department of Psychiatry and Behavioural Neurosciences (Prof A T Olagunju PhD), McMaster University, Hamilton, ON, Canada; Department of Biochemistry and Molecular Biology (M Hasan MPH), Tejgaon College, Dhaka, Bangladesh; Department of Biomedical Engineering and Public Health (S Hasan PhD), World University of Bangladesh, Dhaka, Bangladesh; Gastroenterology and Hepatology Research Center (F Hasani MD), Department of Nursing and Midwifery (F Ranjbar Noei MSc), Golestan Research Center of Gastroenterology and Hepatology (G Roshandel PhD), Golestan University of Medical Sciences, Gorgan, Iran; Department of Public Health (A Hashi PhD), Jigjiga University, Jigjiga, Ethiopia; Department of Pharmacy (Prof M S Hasnain PhD), Marwadi University, Rajkot, India; Public Health Department (I I Hassan PhD), Federal University Teaching Hospital, Lafia, Nigeria; Department of Public Health (I I Hassan PhD), Federal University of Lafia, Lafia, Nigeria; Department of Diagnostic and Interventional Radiology and Neuroradiology (Prof J Haubold MD, Prof B M Schaarschmidt MD), Institute of Artificial Intelligence in Medicine (Prof J Haubold MD), Institute of Diagnostic and Interventional Radiology and Neuroradiology (M Opitz MD), University Hospital Essen, Essen, Germany; Institute of Pharmaceutical Sciences (K Hayat MS), University of Veterinary and Animal Sciences, Lahore, Pakistan; Department of Pharmacy Administration and Clinical

Pharmacy (K Hayat MS), Xian Jiaotong University, Xian, China; Department of Medicine (B Heibati PhD, E Lytvyak MD, A Rafiee MSc), University of Alberta, Edmonton, AB, Canada; Community-Oriented Nursing Midwifery Research Center (M Heidari PhD), Modeling in Health Research Center (A Mohammadian-Hafshejani PhD), Shahrekord University of Medical Sciences, Shahrekord, Iran; Babes-Bolyai University, Cluj-Napoca, Romania (Prof C Herteliu PhD); Department of Virology (Z Heydarifard PhD), Lorestan University of Medical Sciences, khoramabad, Iran; Department of Public Health (D Z Heyi MPH), Madda Walabu University, Robe, Ethiopia; Department of Microbiology (K Hezam PhD), Taiz University, Taiz, Yemen; School of Medicine (K Hezam PhD), Nankai University, Tianjin, China; Graduate School of Medicine (Y Hiraike PhD), Department of Global Health Policy (S K Rauniyar PhD), University of Tokyo, Tokyo, Japan; School of Medicine (M Hoang MD), Tan Tao University, Long An, Viet Nam; Department of Pulmonology (N Horita PhD), Yokohama City University, Yokohama, Japan; National Human Genome Research Institute (NHGRI) (N Horita PhD), National Institutes of Health, Bethesda, MD, USA; Department of Epidemiology and Population Health (H Hosgood PhD), Department of Internal Medicine (X Zhang MD), Albert Einstein College of Medicine, Bronx, NY, USA; Department of Physics (A Hossain PhD), Department of Population Science and Human Resource Development (Prof M Rahman DrPH), University of Rajshahi, Rajshahi, Bangladesh; Department of Decision and Information Sciences (M Hossain DrPH), Department of Biology and Biochemistry (S Ullah MSc), University of Houston, Houston, TX, USA; Public Health Research Group (M Hossain DrPH), Nature Study Society of Bangladesh, Khulna, Bangladesh; Department of Statistics (M Hossain BSc, S Noor MS), Shahjalal University of Science and Technology, Sylhet, Bangladesh; Department of Population Sciences (Prof M B Hossain PhD), University of Dhaka, Dhaka, Bangladesh; Department of Health Services Administration (V Hsieh PhD), China Medical University, Taichung, Taiwan; Department of Psychological and Cognitive Sciences (C Hu PhD), Tsinghua Vanke School of Public Health (Z Li PhD), Tsinghua University, Beijing, China; Nephrology and Urology Research Center (K Hushmandi PhD), Baqiyatallah University of Medical Sciences, Tehran, Iran; Czech National Centre for Evidence-Based Healthcare and Knowledge Translation (S Hussain PhD), Institute of Biostatistics and Analyses (S Hussain PhD), Masaryk University, Brno, Czech Republic; Clinical Governance and Quality Improvement Head (D Hussein MPH), Salale University, Gerba Guracha, Ethiopia; Department of Biomolecular Sciences (Prof N R Hussein PhD), University of Zakho, Zakho, Iraq; Artur Riggs Diabetes & Metabolism Research Institute (Prof M I Husseiny PhD), Cancer Prevention and Research Institute, Duarte, CA, USA; International Master Program for Translational Science (H Huynh BS), International Ph.D. Program in Medicine (M H N Le MD), Research Center for Artificial Intelligence in Medicine (M H N Le MD), College of Medicine (K Nguyen PhD), Department of Clinical Pharmacy (M A Sarasmita PharmD), School of Nursing (A L Wicaksana MS), Taipei Medical University, Taipei, Taiwan; Department of Safety and Bioethics (Prof I Iavicoli PhD), Università Cattolica del Sacro Cuore, Rome, Italy; Genetics and Molecular Biology Department (R Ibrahim PhD), Abu Dhabi University, Abu Dhabi, United Arab Emirates; Department of Medicine (F J Idalsoaga MD), Western University, London, ON, Canada; Health Policy and Management Department (P M Iftikhar MD), City University of New York, New York, NY, USA; Collaborative Alliance Research and Education (CARE) Programme (A Ikiroma PhD), Episcope Research Service, Aberdeen, Scotland; West Africa RCC (O S Ilesanmi PhD), Africa Centre for Disease Control and Prevention, Abuja, Nigeria; Faculty of Medical Sciences (Prof M D Ilic PhD), University of Kragujevac, Kragujevac, Serbia; Department of Neurosurgery (M F Ilyas MD), Universitas Sebelas Maret (March Eleventh University), Jakarta, Indonesia; Department of Clinical Pharmacy (M Imam PhD), Department of Electrical Engineering (I Malik PhD), Prince Sattam bin Abdulaziz University, Al Kharj, Saudi Arabia; Department of Biotechnology (M A Isa PhD), Sharda University, Greater Noida,

India; School of Pharmacy (M Islam PhD), BRAC University, Dhaka, Bangladesh; Clinical Laboratory Department (F Ismail PhD), Tobruk University, Tobruk, Libya; Department of Blood Transmitted Diseases (F Ismail PhD), National Centre for Disease Control (NCDC), Tobruk, Libya; Feinberg School of Medicine (R Issa BA, A Keshwani MPH, D B Srivastava BA), Medical Scientist Training Program (S Marzouk MA), Northwestern University, Chicago, IL, USA; Department of Health Services Research (M Iwagami PhD), University of Tsukuba, Tsukuba, Japan; Department of Non-Communicable Disease Epidemiology (M Iwagami PhD), Department of Health Services Research and Policy (Prof M McKee DSc), Faculty of Epidemiology and Population Health (B A Yirdaw PhD), London School of Hygiene & Tropical Medicine, London, UK; Centre for Neuroscience, Department of Biotechnology (M Iyer PhD), Karpagam Academy of Higher Education, Coimbatore, Coimbatore, India; Department of Physical Medicine and Rehabilitation (L Jacob MD), Université Paris Cité, Paris, France; Parc Sanitari Sant Joan de Déu, Research and Development Unit (L Jacob MD), Biomedical Research Networking Center for Mental Health Network (CiberSAM), Barcelona, Spain; Department of Immunology (Prof A Jafarzadeh PhD), Department of Epidemiology and Biostatistics (Prof M Rezaeian PhD), Rafsanjan University of Medical Sciences, Rafsanjan, Iran; Government Hospitals, Manama, Bahrain (H Jahrami PhD); Department of Health and Safety (A A Jairoun PhD), Dubai Municipality, Dubai, United Arab Emirates; Centre for Community Medicine (A Jaiswal MD), Department of Preventive Oncology (J K Meena MD), Department of Biophysics (T Mohammad PhD), Medical Oncology Lab (C P Prasad PhD, M Singh PhD), Department of Radiation Oncology (A Shankar MD), Department of Laboratory Medicine (A Singh PhD), All India Institute of Medical Sciences, New Delhi, India; UNESCO-TWAS Section of Economic & Social Sciences, Humanities & Arts (Prof M Jakovljevic PhD), The World Academy of Sciences UNESCO-TWAS, Trieste, Italy; Shaanxi University of Technology, Hanzhong, China (Prof M Jakovljevic PhD); Department of Environmental Engineering (Prof R Jalilzadeh Yengejeh PhD), Department of Medicinal Chemistry (M K Mohammadi PhD), Islamic Azad University, Ahvaz, Iran; Department of Neurosurgery (M Jalloh MD), Division of Hematology and Oncology (J F Wu MD), Medical College of Wisconsin, Milwaukee, WI, USA; Department of Health Informatics, College of Applied Medical Sciences (Q Jamal PhD), Qassim University, Buraydah, Saudi Arabia; Department of Public Health (S Jamil MPH), Daffodil International University, Dhaka, Bangladesh; Department of Public and Community Health (S Jamil MPH), Frontier University Garowe, Puntland, Somalia; Department of Medicine (S Javaid MD), University of Mississippi Medical Center, Jackson, MS, USA; Department of Medicine (S Javaid MD), Jinnah Sindh Medical University, Karachi, Pakistan; Postgraduate Institute of Medicine (U Jayarajah MD, J Samaranayake MBBS), Department of Anatomy, Genetics and Biomedical Informatics (Y Mathangasinghe PhD), Department of Surgery (D P Wickramasinghe MD), University of Colombo, Colombo, Sri Lanka; Department of Surgery (U Jayarajah MD), National Hospital, Colombo, Sri Lanka; Department of Biochemistry (Prof S Jayaram MD), Government Medical College, Mysuru, India; Department of Oral Medicine and Periodontology (Prof R D Jayasinghe MS), University of Peradeniya, Peradeniya, Sri Lanka; Graphic Era Deemed to be University (D Jena PhD), Graphic Era Deemed to be University, Dehradun, India; School of Biology and Engineering (School of Health Medicine Modern Industry) (S Jin MPH), Guizhou Medical University, Guiyang, China; Department of Orthopedics (W Jin MD), Department of Epidemiology and Biostatistics (Prof S Mubarik PhD, Prof C Yu PhD), School of Public Health (Prof Z Zhang PhD), Wuhan University, Wuhan, China; Faculty of Veterinary Medicine (M Jokar DVM), Cumming School of Medicine (M I Olatubi PhD), University of Calgary, Calgary, AB, Canada; Rothschild Foundation Hospital (Prof J B Jonas MD), Institut Français de Myopie, Paris, France; Singapore Eye Research Institute (Prof J B Jonas MD), Singapore Eye Research Institute, Singapore, Singapore; Health Services

Management Training Centre (T Joo PhD, J Lám PhD, T Palicz MD), Semmelweis University, Budapest, Hungary; Hungarian Health Management Association, Budapest, Hungary (T Joo PhD, T Palicz MD); NGSM Institute of Pharmaceutical Sciences (J Jose PhD), KS Hegde Medical Academy (S S Shetty MD), Nitte University, Mangalore, India; Department of Gastroenterology and Hepatology (A Joseph MD), Department of Radiology (S Ramasamy MD), Stanford University, Stanford, CA, USA; Department of Economics (C E Joshua BSc), National Open University, Benin City, Nigeria; Institute of Family Medicine and Public Health (M Jürisson PhD), University of Tartu, Tartu, Estonia; Department of Life Science (A Jyoti PhD), Department of Biotechnology, Parul Institute of Technology (J Saxena PhD), Parul University, Vadodara, India; Research Department (Z Kabir PhD), TobaccoFree Research Institute Ireland, Dublin, Ireland; School of Public Health (Z Kabir PhD), University College Cork, Cork, Ireland; SUNY Upstate Medical University (M Kabiri PharmD), Microbiology and Immunology Department (M Kabiri PharmD), State University of New York, Syracuse, NY, USA; Department of Statistics (Prof D H Kadir PhD), Salahaddin University, Erbil, Iraq; Department of Behavioral and Community Health (E Kalan PhD), University of Maryland, College Park, MD, USA; College of Pharmacy (M Kamal PhD), Jamia Hamdard, Al Kharj, Saudi Arabia; Department of Biostatistics (V K Kamal PhD), All India Institute of Medical Sciences, Kalyani, India; Department of Biostatistics (V K Kamal PhD), Indian Council of Medical Research, New Delhi, India (D K Lal MD); Department of Public Health (R T Kamorudeen MPH), South Wales University, Treforest, UK; Osun State Hospital Management Board, (R T Kamorudeen MPH); T.H. Chan School of Public Health (S B Kankam MD, M Tuffour Amirikah MD), Department of Medicine (M Kokkorakis BSc, F Tabatabaei MD), Department of Global Health and Population (Z Li PhD), Department of Health Policy and Oral Epidemiology (Z S Natto DrPH), Harvard Medical School (T J Roberts MD, A Zhong MA), Division of Global Health Equity (P Rohloff MD), Department of Ophthalmology (M Shayan MD), Harvard University, Boston, MA, USA; Office of the Executive Director (Prof K K Kanmodi MPH), Cephas Health Research Initiative Inc, Ibadan, Nigeria; Laboratory Science Department (J Karami PhD), Khomein University of Medical Sciences, Khomein, Iran; Department of Basic Medical Sciences (R A Karasneh PhD, Prof M M Khatatbeh PhD), Yarmouk University, Irbid, Jordan; Institute for Epidemiology and Social Medicine (A Karch MD), University of Münster, Münster, Germany; Chair and Department of Medical Microbiology (Prof T M Karpiński DDS), Poznan University of Medical Sciences, Poznan, Poland; Department of Physical Therapy and Health Rehabilitation (Prof F Z Kashoo PhD), Majmaah University, Majmaah, Saudi Arabia; Surgery Research Unit (Prof J H Kauppila MD), University of Oulu, Oulu, Finland; Department of ENT (N Kaur MS), Dr. B. R. Ambedkar State Institute of Medical Sciences (AIMS), Mohali, India; Adult Health Nursing Department (F A Kebede MSc), Department of Adult Health Nursing (F A Kebede MSc), Ethiopian Public Health Institute, West Hararghe, Ethiopia; Department of Human Nutrition (E Kesse-Guyot PhD), National Research Institute for Agriculture, Food and Environment, Jouy-en-Josas, France; Department of Health, Medicine and Human Biology (M Touvier PhD), Sorbonne Paris Nord University, Bobigny, France (E Kesse-Guyot PhD); Amity Institute of Forensic Sciences (H Khajuria PhD, B P Nayak PhD), Amity Institute of Pharmacy (K Munjal PhD), Amity Institute of Public Health (M Shannawaz PhD), Amity University, Noida, India; Faculty of Veterinary Medicine (H O Khalifa PhD), Kafrelsheikh University, Kafrelsheikh, Egypt; Department of Public Health (Prof M khalis PhD), Mohammed VI Center for Research and Innovation, Rabat, Morocco; Higher Institute of Nursing Professions and Health Techniques, Rabat, Morocco (Prof M khalis PhD); Halal Research Center of the Islamic Republic of Iran (IRI) (F Khamesipour PhD), Iran Food and Drug Administration, Tehran, Iran; Department of Pharmacy Administration and Clinical Pharmacy (F U Khan PhD), Department of Epidemiology and Biostatistics (Prof J Liu PhD), Peking University, Beijing, China; Department of

Pharmacy Administration and Clinical Pharmacy (F U Khan PhD), Department of Pharmacy (S Mushtaq PhD), The First Affiliated Hospital of Xi'an Jiaotong University, Xi'an, China; BDStatistics Center for Research, Dhaka, Bangladesh (M Khan MPH); Primary Care Department (M A Khan MSc), NHS North West London, London, UK; Joint Doctoral School (S A Khan MSc), Silesian University of Technology, Gliwice, Poland; Dr. Panjwani Center for Molecular Medicine & Drug Research (S A Khan MSc), International Center for Chemical and Biological Sciences (S Ullah MSc), University of Karachi, Karachi, Pakistan; Global Consortium for Public Health Research (Prof M Khatib PhD), Datta Meghe Institute of Higher Education and Research, Wardha, India; Academy of Medical Science, Tehran, Iran (M Khayamzadeh MD); Department of Biochemistry (F Khidri PhD), Liaquat University of Medical and Health Sciences, Jamshoro, Pakistan; Department of Internal Medicine (A A Khosla MD), Corewell Health East William Beaumont University Hospital, Royal Oak, MI, USA; Department of Medical Oncology (A A Khosla MD), Department of Medical and Surgical Oncology (A Pon Avudaiappan MD), Miami Cancer Institute, Miami, FL, USA; Department of Public Health (J Khubchandani PhD, S Roy MD), New Mexico State University, Las Cruces, NM, USA; School of Medicine (Prof K Kim PhD), Creighton University, Omaha, NE, USA; School of Traditional Chinese Medicine (Y Kim PhD), Xiamen University Malaysia, Sepang, Malaysia; Health and Healing Research, Education, and Service, Inc., Boston, MA, USA (R W Kimokoti MD); Millennium Prevention, Inc., Westwood, MA, USA (R W Kimokoti MD); School of Health Sciences (Prof A Kisa PhD), Kristiania University College, Oslo, Norway; Department of International Health and Sustainable Development (Prof A Kisa PhD), Department of Environmental Health Sciences (S P Sherchan PhD), Tulane University, New Orleans, LA, USA (E Lytvyak MD); Department of Nursing and Health Promotion (S Kisa PhD), Faculty of Health Sciences (Prof A W Wolf PhD), Oslo Metropolitan University, Oslo, Norway; Department of Public Health Dentistry (Prof S KM PhD), Krishna Vishwa Vidyapeeth (Deemed to be University), Karad, India; Department of Ophthalmology (Prof A G Konstas PhD), First Department of Ophthalmology (Prof G D Panos MD), Aristotle University of Thessaloniki, Thessaloniki, Greece; Department of Epidemiology (Prof K Kostev PhD), IQVIA, Frankfurt am Main, Germany; University Hospital Marburg, Marburg, Germany (Prof K Kostev PhD); Department of Biochemistry (A L Kotnis PhD), All India Institute of Medical Sciences, Bhopal, India; Kasturba Medical College, Manipal (S Koulmane Laxminarayana MD), Manipal Academy of Higher Education, Udupi, India; School of Pharmacy (Prof I A Kretchy PhD), Department of Psychology (E Teye-Kwadjo PhD), University of Ghana, Legon, Ghana; Department of Anthropology (Prof K Krishan PhD), Institute of Forensic Science & Criminology (V Sharma PhD), Panjab University, Chandigarh, India; Department of Biotechnology (R H Kuchay PhD), Baba Ghulam Shah Badshah University, Jammu and Kashmir, India; Center of Medicine and Public Health (M Kulimbet MSc), Asfendiyarov Kazakh National Medical University, Almaty, Kazakhstan; Department of Community Medicine (D Kumar MD), Rajendra Institute of Medical Sciences, Ranchi, India; Centre for Digital Transformation (V Kumar PhD), Indian Institute of Management, Ahmedabad, Ahmedabad, India; Department of Public Health (S Kundu MPH), Griffith University, Gold Coast, QLD, Australia; Section of Cardiology (Prof S K Kunutsor PhD), University of Manitoba, Winnipeg, MB, Canada; Translational Health Sciences (Prof S K Kunutsor PhD), Bristol Medical School, Population Health Sciences (Y Nartey PhD), University of Bristol, Bristol, UK; Institute for Health Sciences (C Y Kustanti PhD), STIKES Bethesda Yakkum Yogyakarta Indonesia, Yogyakarta, Indonesia; Department of Pediatric Oncology (Prof T Kutluk MD), Medicana Health International, Istanbul, Turkiye; Department of Pediatric Oncology (Prof T Kutluk MD), Hacettepe University, Ankara, Turkiye; Department of Nephrology (A Kuttikkattu MD), Pushpagiri Institute of Medical Sciences and Research Centre, Thiruvalla, India; Department of Nursing (G K Kyei BSc), University of Massachusetts Boston, Boston, MA, USA; Department of Environment and Public

Health (F Kyei-Arthur PhD), University of Environment and Sustainable Development, Somanya, Ghana; Clinical Research Center (V Kytö MD), Turku University Hospital, Turku, Finland; Division of Evidence Synthesis (C Lahariya MD), Foundation for People-centric Health Systems, New Delhi, India; Division of Lifestyle Medicine (C Lahariya MD), Centre for Health: The Specialty Practice, New Delhi, India; School of Digital Science (D Lai PhD), Institute of Applied Data Analytics (D Lai PhD), Faculty of Science (E Leong PhD), Universiti Brunei Darussalam (University of Brunei Darussalam), Bandar Seri Begawan, Brunei; Department of Occupational and Environmental Health (H Lai PhD), Division of Gastroenterology (Prof Z Wu PhD), Huazhong University of Science and Technology, Wuhan, China; Department of Respiratory and Critical Care Medicine (H Lai PhD), Northern Jiangsu People's Hospital, Yangzhou, China; NEVES Society for Patient Safety, Budapest, Hungary (J Lám PhD); Department of Experimental Medicine (DIMES) (F Lanfranchi MD), University of Genoa, Genoa, Italy; Department of Otorhinolaryngology (S Lasrado MS), Father Muller Medical College, Mangalore, India; International Society of Doctors for the Environment, Arezzo, Italy (P Lauriola MD); Department of Clinical Pharmacy and Pharmacy Management (B K Lawal PhD), Department of Radiology (H Theyra-Enias MD), Kaduna State University, Kaduna, Nigeria; Faculty of Medicine (H Le MD, N Le MD), School of Medicine (T Nguyen MD), Department of General Medicine (V T Nguyen MD), Department of Internal Medicine (T H Tran MD), University of Medicine and Pharmacy at Ho Chi Minh City, Ho Chi Minh City, Viet Nam (T T T Le MD); Department of Cardiovascular Research (H Le MD, N Le MD), Methodist Hospital, Merrillville, IN, USA; STEM (Prof I Lee PhD), Department of Allied Health and Human Performance (T Y Tiruye PhD), University of South Australia, Adelaide, SA, Australia; Pattern Recognition and Machine Learning Lab (Prof S Lee PhD), Gachon University, Seongnam, South Korea; Department of Precision Medicine (Prof S Lee MD), Sungkyunkwan University, Suwon-si, South Korea; School of Pharmacy (Prof S W H Lee PhD), Jeffrey Cheah School of Medicine and Health Sciences (Prof R R Marzo MD), Monash University, Subang Jaya, Malaysia; School of Pharmacy (Prof S W H Lee PhD), Taylor's University Lakeside Campus, Subang Jaya, Malaysia; Obstetrics and Gynecology Department (A Y Legesse MD), Department of Medical Biochemistry and Molecular Biology (W B Tesfamariam MSc), Mekelle University, Mekelle, Ethiopia; Department of Health Promotion and Health Education (M Li PhD), National Taiwan Normal University, Taipei, Taiwan; The First Affiliated Hospital of Guangzhou Medical University (W Li MD), Guangzhou Medical University, Guangzhou, China; Department of Psychiatry (W Li PhD), Department of Radiology and Biomedical Imaging (X Liu PhD, S Rahmani MD), Yale University, New Haven, CT, USA; Department of Health Management Center (X Li PhD), Fudan University, Shanghai, China; Department of Endocrinology and Metabolism (Prof Y Li PhD), The First Hospital of China Medical University, Shenyang, China; School of Medicine (Z Li PhD), School of Global health (J Zheng PhD), Shanghai Jiao Tong University, Shanghai, China; Department of Food Science and Human Nutrition (Q Lin MPH), Iowa State University, Ames, IA, USA; Department of Quantitative Health Science (Prof X Liu PhD), Department of Neurology (G Nepal MD), University Hospitals (O A Oyebanji MD), Department of Nutrition and Preventive Medicine (Prof J Sanabria MD), Department of Endocrinology (A Sood MD), Case Western Reserve University, Cleveland, OH, USA; Department of Molecular Epidemiology (E Llanaj PhD), German Institute of Human Nutrition Potsdam-Rehbrücke, Potsdam, Germany; German Center for Diabetes Research (DZD), München-Neuherberg, Germany (E Llanaj PhD); Geospatial Health and Development Team-Child Health Analytics (J Lubinda PhD), Telethon Kids Institute, Perth, WA, Australia; Scientific Research and Surveillance Systems (J Lubinda PhD), Macha Research Trust, Choma, Zambia; Dodoma Medical Research Centre (A M Lutambi PhD), National Institute for Medical Research in Tanzania, Dodoma, Tanzania; College of Engineering (Prof M D Lytras PhD), Effat University, Jeddah, Saudi Arabia;

Management of Information Systems Department (Prof M D Lytras PhD), The American College of Greece, Aghia Paraskevi, Greece; Centre for Public Health and Wellbeing (Z Ma PhD), University of the West of England, Bristol, UK; Department of Biostatistics and Epidemiology (F Madadizadeh PhD), Yazd University of Medical Sciences, Yazd, Iran; Department of Research (A A Mahamed MPH), Kayd Consultancy, Garowe, Somalia; Research Center (N H Mahmood PhD), Cihan University-Sulaimaniya, Sulaymaniyah, Iraq; Department of Clinical and Hospital Pharmacy (M A Mahmoud PhD), Ashok and Rita Patel Institute of Physiotherapy, Al-Madinah Al-Munawarrah, Saudi Arabia; Department of Medicine (O M Makram MD), Medical College of Georgia at Augusta University, Augusta, GA, USA; Cyprus International Institute for Environmental and Public Health (Prof K C C Makris PhD), Cyprus University of Technology, Limassol, Cyprus; Department of Chemistry and Biochemistry (S Malasala PhD), University of South Carolina, Columbia, SC, USA; Rama Medical College Hospital and Research Centre, Uttar Pradesh, India (K Malhotra MBBS); Department of Medical Microbiology (L A Malinga PhD), University of Pretoria, Pretoria, South Africa; Department of Health Research (L A Malinga PhD), Ministry of Health, Pretoria, South Africa; Biomedical Engineering Research Center (CREB) (Prof M Mansourian PhD), Automatic Control Department (H Marateb PhD), Universitat Politècnica de Catalunya (Barcelona Tech - UPC), Barcelona, Spain; Department of Biomedical Engineering (H Marateb PhD, M Noroozi BSc), University of Isfahan, Isfahan, Iran; Faculty of Veterinary Medicine (Prof B H Marghani PhD), King Salman International University, Ras Sudr, Egypt; Faculty of Public Health (Prof S Martini PhD), Universitas Airlangga (University of Airlangga), Surabaya, Indonesia; Indonesian Public Health Association, Surabaya, Indonesia (Prof S Martini PhD); Department of Nutrition and Dietetics (M Martorell PhD), Centre for Healthy Living (M Martorell PhD), University of Concepción, Concepción, Chile; Faculty of Humanities and Health Sciences (Prof R R Marzo MD), Curtin University, Sarawak, Malaysia; Department of Anatomy and Developmental Biology (Y Mathangasinghe PhD), Monash University, Clayton, VIC, Australia; Department of Community Medicine (M Mathur MD), Geetanjali Medical College and Hospital, Udaipur, India; Australian Centre for Health Services Innovation (Prof S M McPhail PhD), Queensland University of Technology, Kelvin Grove, QLD, Australia; Digital Health and Informatics Directorate (Prof S M McPhail PhD), Queensland Health, Brisbane, QLD, Australia; Department of Healthcare (Prof E A Mechili PhD), University of Vlora, Vlora City, Albania; Clinic of Social and Family Medicine (Prof E A Mechili PhD), Department of Medicine (Prof A Tsatsakis DSc), University of Crete, Heraklion, Greece; National Heart, Lung and Blood Institute (Prof R Mehboob PhD), National Heart, Lung, and Blood Institute, Bethesda, MD, USA; Research and Development Department (Prof R Mehboob PhD), Lahore Medical Research Center, Lahore, Pakistan; Department of Medical Oncology and Hematology (M A Mendez-Lopez PhD), Hoch St. Gallen, St. Gallen, Switzerland; Dirección General de Investigación, Desarrollo e Innovación (DGIDI) (W Mendoza MD), Universidad Científica del Sur (University of the South), Lima, Peru; Division of Forensic Medicine (Prof R G Menezes MD), Imam Abdulrahman Bin Faisal University, Dammam, Saudi Arabia; Epidemiology, Biostatistics and Prevention Institute (D Menges PhD), University of Zürich, Zurich, Switzerland; Virginia Tech Carilion School of Medicine (T P Menon BS), Virginia Tech, Roanoke, VA, USA; International Dx Department (A A Mentis MD), BGI Genomics, Copenhagen, Denmark; General Administration Department (A Meretoja MD), Comprehensive Cancer Center (T J Meretoja MD), Department of Neurosurgery (I Rautalin PhD), Helsinki University Hospital, Helsinki, Finland; University of Helsinki, Helsinki, Finland (T J Meretoja MD); University Centre Varazdin (T Mestrovic PhD), University North, Varazdin, Croatia; Faculty of Veterinary Medicine (Prof M M M Metwally PhD), King Salman International University, Ras Sedr, Egypt; Department of Propedeutics of Internal Diseases & Arterial Hypertension (Prof T Miazgowski MD), Pomeranian Medical University, Szczecin, Poland; Department of

Pathology (I Michalek PhD), Maria Sklodowska-Curie National Research Institute of Oncology, Warsaw, Poland; College of Human Medicine (T R Miller PhD), Michigan State University, Flint, MI, USA; Multidisciplinary Department of Medical-Surgical and Dental Specialties (G Minervini PhD), University of Campania Luigi Vanvitelli, Naples, Italy; National Data Management Center for Health (A Misganaw PhD), Ethiopian Public Health Institute, Addis Ababa, Ethiopia; Department of Family Medicine (S A Yesuf MSc), St. Paul's Hospital Millennium Medical College, Addis Ababa, Ethiopia (Y Mogessie MD); College of Applied and Natural Science (J Mohamed MSc), University of Hargeisa, Hargeisa, Somalia; RAK College of Nursing (M Mohamed PhD), Department of Pharmacology (S Srinivasamurthy MD), RAK Medical and Health Sciences University, Ras Al Khaimah, United Arab Emirates; Nursing College (M Mohamed PhD), Sohag University, Sohag, Egypt; Molecular Biology Unit (N S Mohamed MSc), Bio-Statistical and Molecular Biology Department (N S Mohamed MSc), Sirius Training and Research Centre, Khartoum, Sudan; Department of Medical Microbiology (K A Mohammad PhD), Knowledge University, Erbil, Iraq; Department of Medicine (S Mohammadi BS), Anne Burnett Marion School of Medicine at Texas Christian University, Fort Worth, TX, USA; Graduate School of Health Sciences (A Mohammed PhD), Ankara University, Ankara, Turkiye; Faculty of Pharmaceutical Sciences (A Mohammed PhD), Bayero University, Kano, Kano, Nigeria; Health Systems and Policy Research Unit (Prof S Mohammed PhD), Department of Community Medicine (S S Umar FWACS), Department of Community medicine (U M Umar MPH), Ahmadu Bello University, Zaria, Nigeria; Heidelberg Institute of Global Health (HIGH) (Prof S Mohammed PhD), Department of Ophthalmology (S Panda-Jonas MD), Heidelberg University, Heidelberg, Germany; Institute of Clinical Physiology (S Molinaro PhD), National Research Council, Pisa, Italy; Clinical Epidemiology and Public Health Research Unit (L Monasta DSc, E Traini PhD, G Zamagni MSc), Burlo Garofolo Institute for Maternal and Child Health, Trieste, Italy; AI & Cyber Futures Institute (M Moni PhD), Charles Sturt University, Bathurst, NSW, Australia; Department of Epidemiology and Biostatistics (Y Moradi PhD), Kurdistan University of Medical Sciences, Sanandaj, Iran; Computer, Electrical, and Mathematical Sciences and Engineering Division (P Moraga PhD), King Abdullah University of Science and Technology, Thuwal, Saudi Arabia; Division of Plastic and Reconstructive Surgery (S D Morrison MD), University of Washington Medical Center, Seattle, WA, USA; Faculty of Medicine (M Morsy MD), October 6 University, Giza, Egypt; Department of Health Policy (Prof E Mossialos PhD), London School of Economics and Political Science, London, UK; Department of Occupational Therapy (F Motaharinezhad PhD), Iran University of Medical Sciences, Semnan, Iran; Department of Radiology (A Msherghi MD), University of Tripoli, Tripoli, Libya; Arid Agriculture University Rawalpindi (R Mubarak MSc), PMAS Arid Agriculture University Rawalpindi, Rawalpindi, Pakistan; Unit of Pharmacotherapy, Epidemiology and Economics (Prof S Mubarik PhD), University of Groningen (Rijksuniversiteit Groningen), Groningen, Netherlands; Department of Surgery (G D Mukoro MD), Ahmadu Bello University Teaching Hospital, Zaria, Nigeria; Department of Medicine (A Mulita PhD), Democritus University of Thrace, Alexandroupolis, Greece; Clinical Epidemiology Research Unit (E Murillo-Zamora PhD), Mexican Institute of Social Security, Villa de Alvarez, Mexico; Postgraduate in Medical Sciences (E Murillo-Zamora PhD), Universidad de Colima, Colima, Mexico; Department of Pathology and Microbiology (S I Mustafa PhD), Duhok University, Duhok, Iraq; Prince Fahad bin Sultan Chair for Biomedical Research (S Muthupandian PhD), University of Tabuk, Tabuk, Saudi Arabia (S Muthupandian PhD); Department of Psychiatry (W Myung PhD), Department of Food and Nutrition (A P Okekunle PhD), Seoul National University, Seoul, South Korea; Department of Neuropsychiatry (W Myung PhD), Seoul National University Bundang Hospital, Seongnam, South Korea; Research and Analytics Department (A J Nagarajan MTech), Initiative for Financing Health and Human Development, Chennai, India; Department of Research and Analytics (A

J Nagarajan MTech), Bioinsilico Technologies, Chennai, India; Institute of Epidemiology and Medical Biometry (Prof G Nagel PhD), Ulm University, Ulm, Germany; Department of Computer Science (P Naghavi MS), University of Illinois, Urbana, IL, USA; Department Health Services Research (G Naik MPH), Department of Radiology (M Tanwar MD), University of Alabama at Birmingham, Birmingham, AL, USA; Laboratory of Public Health Indicators Analysis and Health Digitalization (M Naimzada MD, S S Otstavnov PhD), Moscow Institute of Physics and Technology, Dolgoprudny, Russia; Experimental Surgery and Oncology Laboratory (M Naimzada MD), Kursk State Medical University, Kursk, Russia; Faculty of Pharmacy (Prof F Nainu PhD), Hasanuddin University, Makassar, Indonesia; Department of Community Medicine (T S Nair MD), MOSC Medical College, Kolenchery, India; College of Health Sciences (H H R Najmuldeen PhD), Cihan University Sulaimaniya, Sulaymaniyah, Iraq; Department of Preventive Medicine and Public Health (Prof H Nam PhD), Chungnam National University School of Medicine, Daejeon, South Korea; Daejeon Regional Cancer Center (Prof H Nam PhD), Chungnam National University Hospital, Daejeon, South Korea; Division of Endocrinology and Diabetes (M Nassar PhD), University of Vermont, South Burlington, VT, USA; Department of Circulation and Medical Imaging (J Nauman PhD), Norwegian University of Science and Technology, Trondheim, Norway; Xiamen Cardiovascular Hospital of Xiamen University (N PhD), Fujian Branch of National Clinical Research Center for Cardiovascular Diseases, Xiamen, China; Zilber College of Public Health (M Nayon MPH), University of Wisconsin Milwaukee, Milwaukee, WI, USA; Department of Health Promotion (A Nazri-Panjaki MSc), Health Promotion Research Center (H Okati-Aliabad PhD), Department of Biochemistry (S Sargazi PhD), Zahedan University of Medical Sciences, Zahedan, Iran; Research and Innovation Center (P Ndishimye PhD), Dalhousie University, Kigali, Rwanda; African Institute for Mathematical Sciences, Kigali, Rwanda (P Ndishimye PhD); Department of General Surgery (I Negoi PhD), Emergency University Hospital of Bucharest, Bucharest, Romania; Euromed Research center (Prof C Nejjari MD), Euromed University of Fes, Fez, Morocco; Faculty of Medicine, Pharmacy, and Dentistry (Prof C Nejjari MD), University Sidi Mohammed Ben Abdellah, Fez, Morocco; Research, Innovation, and Data Science Division (J S Ngabonziza PhD), Rwanda Biomedical Centre, Kigali, Rwanda; Institute for Global Health Innovations (C T Nguyen MPH), Duy Tan University, Hanoi, Viet Nam; T.H. Chan School of Public Health (D Nguyen BS), Harvard University, Cambridge, MA, USA; Radiology Department (N Nguyen BSc), University of California Los Angeles, Los Angeles, CA, USA; Hitotsubashi Institute for Advanced Study (HIAS) (T Nguyen DrPH), Hitotsubashi University, Tokyo, Japan; Institute for Cancer Control (T Nguyen DrPH), National Cancer Center, Chuo-ku, Japan; Department of Urology (T Nguyen MD), University of California Irvine, Irvine, CA, USA; Tuberculosis Group (V T Nguyen MD), Oxford University Clinical Research Unit, Vietnam, Ho Chi Minh City, Viet Nam; International Islamic University Islamabad, Islamabad, Pakistan (R K Niazi PhD); Department of Humanities and Social Science (L Nieddu PhD), University for International Studies in Rome, Rome, Italy; School of Medicine (V Niranjan PhD), University of Limerick, Limerick, Ireland; Department of Public Health (V Niranjan PhD), UNICAF, Larnaca, Cyprus; Research Department (J Niyonsenga MPH), JFI Consults Ltd, Kigali, Rwanda; Technical Department (C A Nnaji PhD), School of Public Health and Family Medicine (C A Nnaji PhD), University of Cape Town, Cape Town, South Africa; Center for Public Health (L A Nnyanzi PhD), Teesside University, Middlesbrough, UK; Global Research Institute (Prof S Nomura PhD), Keio University, Tokyo, Japan; Medical Research Council Clinical Trials Unit (N M Noor MRCP), University College London, London, UK; Department of Gastroenterology (N M Noor MRCP), Cambridge University Hospitals, Cambridge, UK; School of Biomedical Engineering, Science and Health Systems (M Noroozi BSc), Drexel University, Philadelphia, PA, USA; Department of Paediatrics (C A Nri-Ezedi PhD), Nnamdi Azikiwe University, Awka, Nigeria; Department of Public Health

(D Nurrika PhD), Banten School of Health Science, South Tangerang, Indonesia; Ministry of Research, Technology and Higher Education (D Nurrika PhD), Higher Education Service Institutions (LL-DIKTI) Region IV, Bandung, Indonesia; Center of Excellence in Reproductive Health Innovation (CERHI) (C I Nzoputam MPH), University of Benin, Benin City, Nigeria; Department of Physiology (O J Nzoputam PhD), University of Benin, Edo, Nigeria; Department of Physiology (O J Nzoputam PhD), Benson Idahosa University, Benin City, Nigeria; Department of Applied Economics and Quantitative Analysis (Prof B Oancea PhD), University of Bucharest, Bucharest, Romania; Bioinformatics Department (Prof B Oancea PhD), National Institute of Research and Development for Biological Sciences, Bucharest, Romania; PSSM Data Sciences, Pfizer Research & Development (M Oduro PhD), Pfizer Inc., Groton, CT, USA; Department of Preventive Medicine (Prof I Oh MD), University of Ulsan, Ulsan, South Korea; School of Pharmacy (O C Okonji MSc), University of the Western Cape, Cape Town, South Africa; Department of Psychiatry (Prof A T Olagunju PhD), University of Lagos, Lagos, Nigeria; Julius Global Health (Prof T O Olanrewaju MSc), Utrecht University, Utrecht, Netherlands; Institute of Chemistry (F B Omage PhD), Universidade Estadual de Campinas (State University of Campinas), Campinas, Brazil; Department of Computational Biology (F B Omage PhD), Brazilian Agricultural Research Institute (EMBRAPA), Campinas, Brazil; Surgery Department (G L Omer MD), Sulaimani University, Sulaimani, Iraq; ENT Department (G L Omer MD), Tor Vergata University of Rome, Rome, Italy; Department of Anatomic Pathology (A E Omonisi FWACP), Ekiti State University, Ado- Ekiti, Nigeria; Department of Anatomic Pathology (A E Omonisi FWACP), Ekiti State University Teaching Hospital, Ado Ekiti, Nigeria; Department of Public Health (S Ong FAMS), Ministry of Health, Bandar Seri Begawan, Brunei; Institute of Health Sciences (S Ong FAMS), Universiti Brunei Darussalam, Bandar Seri Begawan, Brunei; Department of Pharmacotherapy and Pharmaceutical Care (M Ordak PhD), Department of Biochemistry and Pharmacogenomics (M Zielińska MPharm), Medical University of Warsaw, Warsaw, Poland; Sickle Cell Unit (Prof V N Orish PhD), Ho Teaching Hospital, Ho, Ghana; One Health Global Research Group (Prof E Ortiz-Prado PhD), Universidad de las Americas (University of the Americas), Quito, Ecuador; Department of Biological Sciences (A Osborne MSc), Njala University, Freetown, Sierra Leone; Department of Clinical Pharmacy and Pharmacy Practice (A A Osman MSc), University of Gezira, Wad Madani, Sudan; School of Medicine (U L Osuagwu PhD), Western Sydney University, Bathurst, NSW, Australia; Department of Optometry and Vision Science (U L Osuagwu PhD), University of KwaZulu-Natal, KwaZulu-Natal, South Africa; School of Public Health (O J Otorkpa PhD), Texila American University, Georgetown, Guyana; Department of Project Management (S S Otstavnov PhD), Department of Health Care Administration and Economics (Prof V Vlassov MD), National Research University Higher School of Economics, Moscow, Russia; Division of Infectious Diseases (Prof A Ouyahia PhD), University Hospital of Setif, Sétif, Algeria; Operational Research Center in Healthcare (I Ozsahin PhD, Prof U Saeed PhD), Near East University, Nicosia, Turkiye; Department of Respiratory Medicine (Prof M P A DNB), Jagadguru Sri Shivarathreeswara University, Mysore, India; Centre for Biotechnology (S K Panda PhD), Siksha ‘O’ Anusandhan (Deemed to be University), Bhubaneswar, India; Amity Institute of Biotechnology (Prof D Pande Katare PhD), Amity University Uttar Pradesh, Noida, India; Centre for Research and Development (Prof S R Pandi-Perumal MSc), Chandigarh University, Punjab, India; Division of Research and Development (Prof S R Pandi-Perumal MSc), Lovely Professional University, Phagwara, India; Department of Neurological Sciences (K Pang MS), Department of Environmental, Agricultural and Occupational Health (J Taiba PhD), University of Nebraska Medical Center, Omaha, NE, USA; Anesthesiology Department of The Third Xiangya Hospital (K Pang MS), Central South University, Changsha, China; Department of Neurology (L D Panos MD), University of Bern, Biel/Biene, Switzerland;

Department of Neurology (L D Panos MD), University of Cyprus, Nicosia, Cyprus; Department of Emergency Medicine (Prof I Pantazopoulos PhD), University of Thessaly, Larissa, Greece; Department of Emergency Medicine (Prof I Pantazopoulos PhD), University of Bern, Bern, Switzerland; University of Padua, Padua, Italy (M Papa MD); Ottawa Hospital Research Institute, Ottawa, ON, Canada (P Paranjkhoo MD); Division of Health Policy and Management (R R Parikh MD), University of Minnesota, Minneapolis, MN, USA; Department of Medical Sciences (R Passera PhD), University of Torino, Torino, Italy; Department of Imaging (R Passera PhD), AOU Città della Salute e della Scienza di Torino (AOU City of Health and Science of Turin), Torino, Italy; Faculty of Medicine and Health (J Patel MChD), University of Leeds, Leeds, UK; Roswell Park Comprehensive Cancer Center (R J Patel MD), The State University of New York at Buffalo, Buffalo, NY, USA; College of Dental Medicine (Prof S Patil PhD), Roseman University of Health Sciences, South Jordan, UT, USA; Department of Human Anatomy (A Patra MD), Department of Community Medicine and Family Medicine (S S Sahoo MD, M Verma MD), Department of Radiodiagnosis (P Singh MD), All India Institute of Medical Sciences, Bathinda, India; Department of Public Health (J Pekarcikova PhD), Trnava University, Trnava, Slovakia; Department of Biomedical Sciences (U Pensato MD), Humanitas University, Pieve Emanuele (MI), Italy; Australian Institute of Health Innovation (P Peprah MSc), Macquarie University, Sydney, NSW, Australia; Department of Orthopedics (J Pereira MS), Yenepoya Medical College, Mangalore, India; Department of Psychiatry (Prof M F P Peres MD), University of Sao Paulo, São Paulo, Brazil; International Institute for Educational Planning (IIEP) (Prof M F P Peres MD), Albert Einstein Hospital, São Paulo, Brazil; Pennsylvania Cancer and Regenerative Medicine Center (R G Pestell MD), Baruch S Blumberg Institute, Doylestown, PA, USA; Department of Medicine (R G Pestell MD), Xavier University School of Medicine, Woodbury, NY, USA; Facultad de Medicina (Faculty of Medicine) (F E Petermann-Rocha PhD), Universidad Diego Portales (Diego Portales University), Santiago, Chile; School of Cardiovascular and Metabolic Health (F E Petermann-Rocha PhD), University of Glasgow, Glasgow, UK; Department of Internal Medicine (H Pham MD), Weiss Memorial Hospital, Chicago, IL, USA; National Centre for Disease Prevention and Health Promotion (D Pierannunzio PhD), Department of Cardiovascular, Endocrine-metabolic Diseases and Aging (B Unim PhD), National Institute of Health, Rome, Italy; Basic Medical Sciences Department (J D Pillay PhD), Durban University of Technology, Durban, South Africa; International Center of Medical Sciences Research (Z Z Piracha PhD), International Center of Medical Sciences Research, Islamabad, Pakistan (Prof U Saeed PhD); Department of Data Management and Analysis (R Poluru PhD), The INCLEN Trust International, New Delhi, India; University Medical Center Groningen (Prof M J Postma PhD), Interdisciplinary Center Psychopathology and Emotion Regulation (ICPE) (N T Sharew MSc), University of Groningen, Groningen, Netherlands; Independent Consultant, San Diego, CA, USA (D Prabhu PhD); Department of Humanities and Social Sciences (Prof J Pradhan PhD), National Institute of Technology Rourkela, Rourkela, India; Department of Biochemistry (Prof A Prashant PhD), JSS Academy of Higher Education and Research, Mysuru, India; Health Sciences Department (D R A Pribadi MSc), Muhammadiyah University of Surakarta, Sukoharjo, Indonesia; Department of Biostatistics, Epidemiology, and Informatics (J Puvvula PhD), University of Pennsylvania, Philadelphia, PA, USA; Department of Biological Sciences (A S Qazi PhD), National University of Medical Sciences (NUMS), Rawalpindi, Pakistan; Department of Biology (K Qurbani MSc), University of Raparin, Rania, Iraq; Department of Epidemiology (P Raghuveer MD), National Institute of Mental Health and Neurosciences, Bengaluru, India; Faculty of Health Sciences (F M Rahman PhD), Qaiwan International University, Sulaymaniyah, Iraq; Collaboration for Cancer Outcomes Research and Evaluation (CCORE) (M Rahman PhD), University of New South Wales, Liverpool, NSW, Australia; School of Medicine and Public Health

(M Rahman PhD), University of Sydney, Wollongong, NSW, Australia; Department of Biostatistics (Prof M Rahman MS), National Institute of Preventive and Social Medicine, Dhaka, Bangladesh; Institute of Health and Wellbeing (Prof M Rahman PhD), Federation University Australia, Berwick, VIC, Australia; Future Technology Research Center (A Rahmani PhD), National Yunlin University of Science and Technology, Yunlin, Taiwan; Health Service Research and Quality of Life Center (CEReSS) (Prof M Rahmati PhD), Aix-Marseille University, Marseille, France; Division of Gynecology and Human Reproduction Physiopathology (D Raimondo PhD), IRCCS Azienda Ospedaliero-Universitaria di Bologna, Bologna, Italy; Department of Medical, Surgical and Experimental Sciences (I Raimondo MD), University of Sassari, Sassari, Italy; Gynecology and Breast Care Center (I Raimondo MD), Mater Olbia Hospital, Olbia, Italy; Department of Community Medicine (S Rajaa MD), Employees' State Insurance Model Hospital, Chennai, India; Department of Population Health (M Ramadan DrPH), King Saud bin Abdulaziz University for Health Sciences, Jeddah, Saudi Arabia; Department of Anatomy (C Ramasamy MD), Govt. Siddhartha Medical College, Vijayawada, India; Developmental, Molecular & Chemical Biology (P S Ramesh PhD), Tufts Medical Center, Boston, MA, USA; South Asian Institute for Social Transformation (SAIST), Dhaka, Bangladesh (J Rana MPH); Department of Epidemiology, Biostatistics and Occupational Health (J Rana MPH), McGill University, Montreal, QC, Canada; Department of Community Medicine (R K Rana MD), Shaheed Nirmal Mahto Medical College and Hospital, Dhanbad, India; Centre for Clinical Pharmacology (N Rancic PhD), University of Defence in Belgrade, Belgrade, Serbia; Centre for Clinical Pharmacology (N Rancic PhD), Medical College of Georgia at Augusta University, Belgrade, Serbia; Department of Colorectal Surgery (C Rao PhD), North Cumbria Integrated Care NHS Foundation Trust, Carlisle, UK; Department of Oral Medicine and Radiology (K Rao PhD), Nitte (deemed to be) University, Mangalore, India; Department of Oral Pathology, Microbiology and Forensic Odontology (S J Rao MDS), Sharavathi Dental College and Hospital, Shimogga, India; Iranian Research Center on Aging (V Rashedi PhD), University of Social Welfare and Rehabilitation Sciences, Tehran, Iran; Department of Family Medicine (Prof D Rathish PhD), Department of Parasitology (Prof K G Weerakoon PhD), Department of Community Medicine (N D Wickramasinghe MD), Rajarata University of Sri Lanka, Anuradhapura, Sri Lanka; The National Institute for Stroke and Applied Neurosciences (I Rautalin PhD), Auckland University of Technology, Auckland, New Zealand; Academic Public Health England (Prof S Rawaf MD), Public Health England, London, UK; School of Health, Medical and Applied Sciences (L Rawal PhD), CQ University, Sydney, NSW, Australia; Department of Biological Sciences (Prof E Redwan PhD), King Abdulaziz University, Jeddah, Egypt; Department of Protein Research (Prof E Redwan PhD), Research and Academic Institution, Alexandria, Egypt; Department of Obstetrics and Gynecology (S Restaino MD), Department of Mother Child Health (G Vizzielli PhD), Azienda Sanitaria Universitaria Friuli Centrale, Udine, Italy; Network of Immunity in Infection, Malignancy and Autoimmunity (NIIMA) (Prof N Rezaei PhD), Universal Scientific Education and Research Network (USERN), Tehran, Iran; Department of Physiology and Physiotherapy (Prof M R Rizvi PhD), DIT University, Delhi, India; Community Health Department (Prof H A L Rocha PhD), Federal University of Ceará, Fortaleza, Brazil; Department of Nursing in Women's Health (T Rodrigues da Silva PhD), Federal University of São Paulo, São Paulo, Brazil; Department of Community Medicine (R Rohilla MD), Government Medical College, Chandigarh, India; Center for Indigenous Health Research (P Rohloff MD), Wuqu' Kawoq Maya Health Alliance, Tecpan, Guatemala; Department of Ophthalmology and Visual Sciences (A Roshanshad MD), Surgical Oncology Department (S Zafar MD), University of Wisconsin-Madison, Madison, WI, USA; Department of Analytical and Applied Economics (Prof H Rout PhD, P Sahoo MA, C Swain MPhil), RUSA Centre of Excellence in Public Policy and Governance (Prof H Rout PhD), UGC Centre of Advanced Study in

Psychology (Prof M Satpathy PhD), Utkal University, Bhubaneswar, India; Department of Medical Sciences (G Rovera PhD), University of Turin, Turin, Italy; Department of Biochemistry and Food Analysis (N Roy PhD), Patuakhali Science and Technology University, Patuakhali, Bangladesh; Department of Medical Pharmacology (Prof M M Saber-Ayad PhD), Public Health and Community Medicine Department (M R Salem MD), Cairo University, Giza, Egypt; Department of Computer (T Sadegh MSc), University of Science and Culture, Tehran, Iran; Department of Pharmaceutical Chemistry (Prof M Saeb PhD), International Medical University, Gdańsk, Poland; Institute of Food Technology & Nutrition Sciences (K Saeed PhD), Lahore University of Biological and Applied Sciences, Lahore, Pakistan; Department of Statistics (M R Sajid PhD), University of Gujrat, Gujrat, Pakistan; Department of Health Education & Promotion (Prof L Salehi PhD), A.C.S. Medical College and Hospital, Karaj, Iran; Research Center for Health, Safety and Environment (Prof L Salehi PhD), School of Medicine (M Shams-Beyranvand MSc), Alborz University of Medical Sciences, Karaj, Iran; Surgical Department (J Samaranayake MBBS), North Colombo Teaching Hospital, Ragama, Sri Lanka; Institute of Epidemiology and Preventive Medicine (Y L Samodra PhD), National Taiwan University, Taipei, Taiwan; Benang Merah Research Center (Y L Samodra PhD), Benang Merah Research Center (BMRC), Minahasa Utara, Indonesia; Department of Surgery (Prof J Sanabria MD), Marshall University, Huntington, WV, USA; Pharmacy Study Program (M A Sarasmita PharmD), Udayana University, Badung, Indonesia; Indira Gandhi Medical College and Research Institute, Puducherry, India (A Saravanan MD); Department of Food Processing Technology (T Sarkar PhD), West Bengal State Council of Technical Education, Malda, India; Department of Medical and Surgical Sciences (M Sassano MD, Prof F S Violante MD), University of Bologna, Bologna, Italy; Faculty of Health & Social Sciences (B Sathian PhD), Bournemouth University, Bournemouth, UK; Udyam-Global Association for Sustainable Development, Bhubaneswar, India (Prof M Satpathy PhD); Department of Medical Informatics (J Saulam MSc), Kagawa University, Miki-cho, Japan; Food Processing and Nutrition (J Saulam MSc), Karnataka State Akkamahadevi Women's University, Vijayapura, India; Department of Public Health Sciences (M Sawhney PhD), University of North Carolina at Charlotte, Charlotte, NC, USA; Faculty of Business and Computing (Prof C Schinckus PhD), University of the Fraser Valley, Abbotsford, BC, Canada; Department of Finance (Prof C Schinckus PhD), International School of Management, Paris, France; Department of Physiotherapy (I J C Schneider PhD), Federal University of Santa Catarina, Araranguá, Brazil; Department of Medical Statistics (M Šekerija PhD), University of Zagreb, Zagreb, Croatia; Department of Epidemiology and Prevention of Chronic Noncommunicable Diseases (M Šekerija PhD), Croatian Institute of Public Health, Zagreb, Croatia; School of Pharmacy (A K Sendekie MSc), Curtin University, Perth, WA, Australia; Department of Biomedical Sciences (P Sengupta PhD), Gulf Medical University, Ajman, United Arab Emirates; Emergency Department (S Senthilkumaran PhD), Manian Medical Centre, Erode, India; Fourth Department of General Surgery (D Serban PhD), Emergency University Hospital Bucharest, Bucharest, Romania; Department of Medicine (Y Sethi MD), Swami Vivekanand Subharti University, Meerut, India; National Heart, Lung, and Blood Institute (A Seylani MD), National Institutes of Health, Rockville, MD, USA; Department of Community Health (M Shabany PhD), Aja University of Medical Sciences, Tehran, Iran; State Key Laboratories of Chemical Resources Engineering (M Shahab PhD), Beijing University Of Chemical Technology, Beijing, China; Department of Biotechnology (S A Shahid MPhil), Quaid-i-Azam University Islamabad, Islamabad, Pakistan; Department of Chemistry (H R Shahsavari PhD), Institute for Advanced Studies in Basic Sciences (IASBS), Zanjan, Iran; Independent Consultant, Karachi, Pakistan (M A Shaikh MD); Regenerative Medicine Department (E Shams MSc), Royan Institution, Tehran, Iran; National University of Ireland - Galway, Galway, Ireland (D Shan MD); Columbia University, New York, NY, USA (D Shan MD); Department of Nursing (N T Sharew

MSc), School of Nursing and Midwifery (B Taye MSc), Debre Berhan University, Debre Berhan, Ethiopia; Department for Evidence-based Medicine and Evaluation (A Sharifan PharmD), University for Continuing Education Krems, Krems, Austria; Department of Humanities and Social Sciences (R Sharma PhD), National Institute of Technology Kurukshetra, Haryana, India; Department of Biology (S P Sherchan PhD), Morgan State University, Baltimore, MD, USA; General Surgery Department (M Shibani MD), Glasgow Royal Infirmary, Glasgow, UK; Department of Experimental Research (V Shivarov PhD), Medical University Pleven, Pleven, Bulgaria; Department of Genetics (V Shivarov PhD), Sofia University "St. Kliment Ohridiski", Sofia, Bulgaria; Alimentary Tract Research Center (Z Shokati Eshkiki PhD), Ahvaz Jundishapur University of Medical Sciences, Ahvaz, Iran; Department of Research and Academics (S Shrestha PhD), Kathmandu Cancer Center, Bhaktapur, Nepal; Person-Centered Research (S Shrestha PhD), Monash University, Box Hill, VIC, Australia; Kenneth H. Cooper Institute (Prof K Shuval PhD), Texas Tech University Health Sciences Center, Dallas, TX, USA; Advanced Materials Division (N R S Sibuyi PhD), Mintek, Randburg, South Africa; Department of Biotechnology (N R S Sibuyi PhD), University of the Western Cape, Bellville, South Africa; Department of Medical Microbiology and Infectious Diseases (E E Siddig MD), Erasmus University, Rotterdam, Netherlands; RISE Health, University of Beira Interior (Prof L M L R Silva PhD), University of Beira Interior, Covilhã, Portugal; Department of Pharmacology (H Singh DM), Government Medical College and Hospital, Chandigarh, India; School of Medicine (Prof J A Singh MD), Baylor College of Medicine, Houston, TX, USA; Department of Medicine Service (Prof J A Singh MD), US Department of Veterans Affairs (VA), Houston, TX, USA; National Institute of Cancer Prevention and Research, Noida, India (P Singh PhD); Department of Human Genetics (P Singh PhD), Punjabi University Patiala, Patiala, India; Department of Anesthesiology (D Siyoum MD), New York Medical College, Passaic, NJ, USA; Department of Infectious Diseases and Epidemiology (A A Skryabina MD), Pirogov Russian National Research Medical University, Moscow, Russia; Faculty of Public Health (Prof S Solikhah DrPH), Universitas Ahmad Dahlan, Yogyakarta, Indonesia; Global Observatory on Pollution and Health (Prof K Straif PhD), Boston College, Chestnut Hill, MA, USA; ISGlobal Instituto de Salud Global de Barcelona, Barcelona, Spain (Prof K Straif PhD); Department of Medical Sciences (Prof V Subramaniyan PhD), Sunway University, Subang Jaya, Malaysia; Department of Community Medicine (A G Suleiman MPH), Ahmadu Bello University, Kaduna, Nigeria; School of Life Sciences (M Suleman PhD), Xiamen University, Xiamen, China; Faculty of Health Science (D Sulistiyorini MSc), Universitas Indonesia Maju, Jakarta, Indonesia; School of Medicine, Medical Sciences and Nutrition (A Sultan Meo MPH), University of Aberdeen, Aberdeen, UK; Department of Biomedical Sciences (Z Sun PhD), Universiti Putra Malaysia, Selangor, Malaysia; Department of Clinical Pathology (H Susianti PhD), Brawijaya University, Malang, Indonesia; Hospital Central Laboratory (H Susianti PhD), Dr Saiful Anwar General Hospital, Malang, Indonesia; Department of Clinical Research and Development (Prof L Szarpak PhD), LUXMED Group, Warsaw, Poland; Collegium Medicum (Prof L Szarpak PhD), John Paul II Catholic University of Lublin, Lublin, Poland; Department of Pharmacology (S T Y MD), All India Institute of Medical Sciences, Deoghar, India; Department of Neurology (P Tabaee Damavandi MD), Neurocenter of Southern Switzerland (NSI), Lugano, Switzerland; Department of Medicine (Prof R Tabarés-Seisdedos PhD), University of Valencia, Valencia, Spain; Department of Health, Safety, and Environmental Management (R Tabibi PhD), Abadan School of Medical Sciences, Abadan, Iran; Saveetha Medical College and Hospital (M Tabish MPharm), Saveetha Institute of Medical and Technical Sciences, Chennai, India; Living Systems Institute (Y Taheri Abkenar PharmD), University of Exeter, Exeter, UK; Department of Dermato-Venereology (M Tampa PhD), Dr. Victor Babes Clinical Hospital of Infectious Diseases and Tropical Diseases, Bucharest, Romania; Department of Epidemiology (J L Tamuzi MSc), Department of Industrial Psychology (E Teye-Kwadjo

PhD), Stellenbosch University, Cape Town, South Africa; Department of Medicine (J L Tamuzi MSc), Northlands Medical Group, Omuthiya, Namibia; Department of Pharmacology and Therapeutics (S Tariq PhD), The University of Faisalabad, Faisalabad, Pakistan; Department of Cell Therapy and Applied Genomics (A Tbakhi MD), King Hussein Cancer Center, Amman, Jordan; Outpatient Department (D R Terefa MSc), Wollega University, Bedele Town, Ethiopia; Department of Obstetrics and Gynecology (M Thayumana Sundaram MS), St. John's National Academy of Health Sciences, Miami, FL, USA; Department of Endocrinology, Diabetes and Metabolism (Prof N Thomas PhD), Christian Medical College and Hospital (CMC), Vellore, India; Department of Gastroenterology (N K Thomas MD), St. Luke's Hospital, Patanamthitta, India; Faculty of Public Health (J H V Ticoalu MPH), Universitas Sam Ratulangi (Sam Ratulangi University), Manado, Indonesia; Public Health Department (T Y Tiruye PhD), Debre Markos University, Debre Markos, Ethiopia; Interdisciplinary Health Data Center (R Topor-Madry PhD), Jagiellonian University Medical College, Kraków, Poland; Nutritional Epidemiology Research Team (EREN) (M Touvier PhD), National Institute for Health and Medical Research (INSERM), Paris, France; Netherlands Organisation for Applied Scientific Research (TNO) (E Traini PhD), Netherlands Organisation for Applied Scientific Research (TNO), Utrecht, Netherlands; Faculty of Public Health (Prof B X Tran PhD), Vietnam National University, Hanoi, Viet Nam; Bloomberg School of Public Health (Prof B X Tran PhD), Johns Hopkins University, Hanoi, Viet Nam; Second Department of Internal Medicine (Q T H Tran MD), Kansai Medical University, Hirakata, Japan; Department of Business Analytics (T H Tran MD), University of Massachusetts Dartmouth, Dartmouth, MA, USA; Department of Clinical and Experimental Medicine (D Trico MD), University of Pisa, Pisa, Italy; Department of Occupational Health and Safety (A R Tualeka PhD), University of Development, Surabaya, Indonesia; Department of Paraclinical Sciences (S Umakanthan MD), University of the West Indies, St. Augustine, Trinidad and Tobago; Federal University of Health Sciences Azare (L Umar PhD), Federal Teaching Hospital, Azare, Nigeria; Federal Teaching Hospital Azare (L Umar PhD), Federal Medical Centre, Azare, Nigeria; Department of Oncology (S S Umar FWACS), Federal Medical Centre, Gusau, Nigeria; College of Health and Sport Sciences (A G Vaithinathan MSc), University of Bahrain, Zallaq, Bahrain; Faculty of Sciences (J Varasteh MSc), University of Guilan, Rasht, Iran; Department of Infectious Disease (Prof S Vaziri MD), Kermanshah University of Medical Sciences, Kermanshah, Iran; Department of Human Genetics & Molecular Biology (B Vellingiri PhD), Bharathiar University, Coimbatore, India; Department Pediatric Hematology and Oncology (G I Villanueva MD), Hospital de Clinicas Jose de San Martin (Jose de San Martín Clinical Hospital), Buenos Aires, Argentina; Occupational Medicine Unit (Prof F S Violante MD), Sant'Orsola Malpighi Hospital, Bologna, Italy; Department of Medicine (G Vizzielli PhD), University of Udine, Udine, Italy; Research Center for Genetics and Life Sciences (S K Vladimirov PhD), Sirius University of Science and Technology, Krasnodar Region, Russia; Scientific Laboratory of Neurolinguistic and Cognitive Research (S K Vladimirov PhD), Peoples Friendship University of Russia (RUDN University), Moscow, Russia; NUST School of Health Sciences (Prof Y Waheed PhD), National University of Sciences and Technology (NUST), Islamabad, Pakistan; Széchenyi István University, Gyor, Hungary (Prof Y Waheed PhD); Brigham and Women's Hospital, Boston, MA, USA (C Wang PhD); College of Agriculture (X Wang PhD), Northwest A&F University, Xianyang City, China; School of Life Course and Population Sciences (Prof Y Wang PhD), King's College London, London, UK; Department of Biotechnology and Genetic Engineering (M Waqas PhD), Hazara University Mansehra, Mansehra, Pakistan; Centre for Health Policy Research (Prof P Ward PhD), Torrens University Australia, Adelaide, SA, Australia; Competence Center of Mortality-Follow-Up of the German National Cohort (R Westerman DSc), Federal Institute for Population Research, Wiesbaden, Germany; Internal Medicine Department (K Wijarnpreecha MD), University of Arizona, Phoenix, AZ, USA;

Institute of Clinical Epidemiology (Prof P Willeit PhD), Medical University Innsbruck, Innsbruck, Austria; Department of Chemical Toxicology (M W Wojewodzic PhD), Norwegian Institute of Public Health, Oslo, Norway; Institute of Health and Care Sciences (Prof A W Wolf PhD), University of Gothenburg, Gothenburg, Sweden; Department of Food Science and Human Nutrition (Prof F Wu PhD), Michigan State University, East Lansing, MI, USA; Australian Centre for Health Services Innovation (Q Xia PhD), Queensland University of Technology, Brisbane, QLD, Australia; Western Institute of Digital-Intelligent Medicine (Z Xia MD), Chongqing Medical University, Chongqing, China; School of Public Health (L Xiao PhD), Department of Epidemiology (D Yin DrPH), Xuzhou Medical University, Xuzhou, China; Department of Intelligent Medical Engineering (Prof W Xie DrPH), Anhui Medical University, Anhui, China; Department of Surgery (Prof W Xie DrPH), The First Affiliated Hospital of Anhui Medical University, Hefei, Anhui, China; The Second Affiliated Hospital of Harbin Medical University (L Xu Mmed), Harbin Medical University, Harbin, China; Department of Endocrinology (Prof S Xu PhD), University of Science and Technology of China, Hefei, China; School of Medicine (Prof S Xu PhD), University of Rochester, Rochester, NY, USA; School of Medicine (M Xue MSc), Kunming University of Science and Technology, Kunming, China; Department of Cells and Tissues (G Yahya PhD), Molecular Biology Institute of Barcelona, Barcelona, Spain; Department of Medicine (A Yarahmadi PhD), Thomas Jefferson University, Philadelphia, PA, USA; National Center for Chronic and Noncommunicable Disease Control and Prevention (P Ye PhD), Chinese Center for Disease Control and Prevention, Beijing, China; Family Medicine Department (S A Yesuf MSc), St. Peter's Specialized Hospital, Addis Ababa, Ethiopia; Department of Pediatrics (Prof D Yon MD), Kyung Hee University, Seoul, South Korea; Department of Biostatistics (Prof N Yonemoto PhD), University of Toyama, Toyama, Japan; Department of Public Health (Prof N Yonemoto PhD), Juntendo University, Tokyo, Japan; Department of Health Policy and Management (Prof M Z Younis PhD), Jackson State University, Jackson, MS, USA; School of Business & Economics (Prof M Z Younis PhD), Universiti Putra Malaysia (University of Putra Malaysia), Kuala Lumpur, Malaysia; Epidemiology and Cancer Registry Sector (Prof V Zadnik PhD), Institute of Oncology Ljubljana, Ljubljana, Slovenia; Department of Surgery (S Zafar MD), Aga Khan University, Karachi, Pakistan; Islamic Azad University, Tehran, Iran (M Zaghampour MD); Faculty of Medicine and Health Sciences (F Zakham PhD), Hodeidah University, Hodeidah, Yemen; Department of Health Sciences (S Zaman PhD), James Madison University, Harrisonburg, VA, USA; Department of Administration (Prof M Zastrozhin PhD), PGxAI, San Francisco, CA, USA; Department of Clinical Practice, Faculty of Pharmacy (M Zawiah PhD), Northern Border University, Rafha, Saudi Arabia; Department of Pediatrics and Child Health Nursing (A Zemariam MSc), Woldia University, Woldia, Ethiopia; Institute of Diagnostic and Interventional Radiology and Neuroradiology (S Zensen MD), University of Duisburg-Essen, Essen, Germany; Victorian Cancer Registry (J Zhang MD), Cancer Council Victoria, Melbourne, VIC, Australia; College of Medical, Veterinary and Life Sciences (J Zhang MD), University of Glasgow, Glasgow, Scotland; School of Public Policy and Administration (J Zhang BA), Xi 'an Jiaotong University, Xi'an, China; Medical Oncology Department of Gastrointestinal Cancer (L Zhang MS), Cancer Hospital of Dalian University of Technology, Shenyang, China; School of Biomedical Engineering (L Zhang MS), Dalian University of Technology, Dalian, China; Department of Internal Medicine (X Zhang MD), Jacobi Medical Center, Bronx, NY, USA; The First Affiliated Hospital of Jinan University (Y Zhao MD), Jinan University, Guang Zhou, China; National Institute of Parasitic Diseases (J Zheng PhD), Chinese Center for Disease Control and Prevention, Shanghai, China; Department of Hepatology (Prof M Zheng PhD), Wenzhou Medical University, Wenzhou, China; School of Data Science (J Zhou PhD), The Chinese University of Hong Kong, Shenzhen, Shenzhen, China; School of Public Health and Emergency Management (B Zhu PhD), Southern

University of Science and Technology, Shenzhen, China; College of Medicine (O A Zitoun MD), Sulaiman Alrajhi University, Al Bukairiyah, Saudi Arabia; Clinical and Administrative Pharmacy (M Zuber PharmD), University of Georgia, Athens, GA, USA; Clinical Research Centre (Prof S H Zyoud PhD), An-Najah National University Hospital, Nablus, Palestine; Department of Building Engineering and Environment (S H Zyoud PhD), Civil Engineering and Sustainable Structures (S H Zyoud PhD), Palestine Technical University (Kadoorie), Tulkarem, Palestine.

Authors' Contributions

Managing the overall research enterprise

Lisa M Force, Miranda May, Theo Vos, Christopher J L Murray

Writing the first draft of the manuscript

Lisa M Force

Primary responsibility for applying analytical methods to produce estimates

Lisa M Force, Jonathan Kocarnik, Kayleigh Bhangdia, Natalie Pritchett, Andrew Crist, Louise Penberthy

Primary responsibility for seeking, cataloguing, extracting, or cleaning data; designing or coding figures and tables

Lisa Force (data seeking and design of tables and figures), Alistair Acheson, Andrew Crist, Louise Penberthy (extracting and cleaning of data, coding of tables and figures), Lee Deitesfeld (data seeking and data cataloguing), Jonathan Kocarnik, Kayleigh Bhangdia, Natalie Pritchett, and Miranda May (coding, entering values, and/or fact checking of tables and figures)

Providing data or critical feedback on data sources

Bhoomadevi A, Abdallah H A Abd Al Magied, Samar Abd ElHafeez, Parsa Abdi, Wakgari Mosisa Abdisa, Auwal Abdullahi, Armita Abedi, Richard Gyan Aboagye, Hassan Abolhassani, Lucas Guimarães Abreu, Bilyaminu Abubakar, Hana J Abukhadijah, Salahdein Aburuz, Ahmed Abu-Zaid, Raghu Ram Achar, Alistair Acheson, Juan Manuel Acuna, Lisa C Adams, Kamoru Ademola Adedokun, Victor Adekanmbi, Habeeb Omoponle Adewuyi, Qorinah Estiningtyas Sakilah Adnani, Leticia Akua Adzigbli, Feleke Doyore Agide, César Agostinis Sobrinho, Bright Opoku Ahinkorah, Sajjad Ahmad, Tauseef Ahmad, Ayman Ahmed, Gasha Salih Ahmed, Muktar Beshir Ahmed, Roland Eghoghosoa Akhigbe, Salah Al Awaidy, Hanadi Al Hamad, Omar Al Omari, Mohammad Al Qadire, Omar Ali Mohammed Al Zaabi, Abdelazeem M Algammal, Dari Alhuwail, Abid Ali, Syed Shujait Ali, Sheikh Mohammad Alif, Syed Mohamed Aljunid, Hesham M Al-Mekhlafi, Hasan Yaser Alniss, Saleh A Alqahtani, Ahmad Rajeh Al-Qudimat, Intima Alrimawi, Najim Z. Alshahrani, Awais Altaf, Alaa B Al-Tammemi, Nelson Alvis-Guzman, Hany Aly, Abdallah Alzoubi, Alireza Amindarolzarbi, Nafiu Aminu, Hubert Amu, Deanna Anderlini, Hossein Ansariniya, Saeid Anvari, Saleha Anwar, Sumadi Lukman Anwar, Razique Anwer, Ekenedilichukwu Emmanuel Anyabolo, Angela Esi Apeagyei, Jalal Arabloo, Elshaimaa A Arafa, Martina Arcieri, Santhosh Arul, Deepavalli Arumuganainar, Muhammad Abdul Basit Ashraf, Tahira Ashraf, Batyrbek Assembekov, Smita Asthana, Prince Atorkey, Marcel Ausloos, Beatriz Paulina Ayala Quintanilla, Amirali Azimi, Mohd Yusmaidie Aziz, Shahkaar Aziz, Ahmed Y Azzam, Ashish D Badiye, Alaa Aboelnour Badran, Atif Amin Baig, Lovenish Bains, Soham Bandyopadhyay, Aleksandra Barac, Shirin Barati, Mainak Bardhan, Amadou Barrow, Shahid Bashir, Mohammad-Mahdi Bastan, Matteo Bauckneht, Feyisa Shasho Bayisa, Narasimha M Beeraka, Babak Behnam, Diana Fernanda Bejarano Ramirez, Akshaya

Srikanth Bhagavathula, Neeraj Bhala, Kayleigh Bhangdia, Ravi Bharadwaj, Sonu Bhaskar, Ajay Nagesh Bhat, Shuvarthi Bhattacharjee, Gurjit Kaur Bhatti, Jasvinder Singh Bhatti, Raluca Bievel-Radulescu, Cem Bilgin, Monirujjaman Biswas, Obasanjo Afolabi Bolarinwa, Archith Boloor, Hamed Borhany, Souad Bouaoud, Marija M Bozic, Dejana Braithwaite, Felix Busch, Maria Teresa Bustamante-Teixeira, Nadeem Shafique Butt, Carlos A Castañeda-Orjuela, Ferrán Catalá-López, Francieli Cembranel, Aditya Chakraborty, Chiranjib Chakraborty, Vijay Kumar Chattu, Akhilanand Chaurasia, Kai Chen, William C S Cho, Dong-Woo Choi, Sungchul Choi, Bryan Chong, Hitesh Chopra, Shivani Chopra, Dinh-Toi Chu, Sunghyun Chung, Alyssa Columbus, Joao Conde, Andrew Crist, Natalia Cruz-Martins, Xiaochen Dai, Lalit Dandona, Rakhi Dandona, Lucio D'Anna, Pojsakorn Danpanichkul, Aso Mohammad Darwesh, Saswati Das, Fernando Pio De la Hoz, Sindhura Deekonda, Lee Deitesfeld, Abel Desalegn Demeke, Rupak Desai, Devananda Devegowda, Meghnath Dhimal, Sameer Dhingra, Marcello Di Pumpo, Michael J Diaz, Thao Huynh Phuong Do, Wendel Mombaque dos Santos, Ojas Prakashbhai Doshi, Leila Doshmangir, Taiwo Omotayo Dosumu, Robert Kokou Dowou, Senbagam Duraisamy, Ashkan Eighaei Sedeh, Michael Ekholuenetale, Temitope Cyrus Ekundayo, Rabie Adel El Arab, Iman El Sayed, Maysaa El Sayed Zaki, Sherif Elkannishy, Ahmed O Elmehrath, Randa Elsheikh, Yasser Mohamed El-Sherbiny, Abdelgawad Salah Abdelgawad Eltahawy, Talha Bin Emran, Babak Eshrati, Majid Eslami, Sayeh Ezzikouri, Heidar Fadavian, Adeniyi Francis Fagbamigbe, Ayesha Fahim, Saman Fahimi, Aamir Fahira, Ildar Ravisovich Fakhradiyev, Aliasghar Fakhri-Demeshghieh, Luca Falzone, Alireza Farahani, Ebrahim Farashi, Pawan Sirwan Faris, Hossein Farrokhpour, Abidemi Omolara Fasanmi, Alireza Feizkhah, Ginenus Fekadu, Lisa M Force, Arianna Fornari, Maryam Fotouhi, Takeshi Fukumoto, Muktar A Gadanya, Silvano Gallus, Mounika Gangireddy, Teferi Gebru Gebremeskel, Gebremariam Wulie Geremew, Fariba Ghassemi, Elena Ghotbi, Syed Abdullah Gilani, Alem Abera Girmay, Mahaveer Golechha, Davide Golinelli, Shi-Yang Guan, Zheng Guo, Bhawna Gupta, Sapna Gupta, Adrina Habibzadeh, Najah R Hadi, Dariush Haghmorad, Nguyen Hai Nam, Hassen Mosa Mosa Halil, Islam M Hamad, Nadia M Hamdy, Nasrin Hanifi, Ashanul Haque, Josep Maria Haro, Fatemeh Hasani, Alireza Hasanzadeh, Mahgol Sadat Hassan Zadeh Tabatabaei, Simon I Hay, Qiang He, Wen-Qiang He, Mohammad Heidari, Claudiu Herteliu, Demisu Zenbaba Heyi, Mai Hoang, Nobuyuki Horita, Mehdi Hoseinzadeh, H Dean Hosgood, Md Mahbub Hossain, Mohammad Bellal Hossain, Chengxi Hu, Salman Hussain, Dursa Hussein, Nawfal R Hussein, Mohamed Ibrahim Husseiny, Hong-Han Huynh, Segun Emmanuel Ibitoye, Pulwasha Maria Iftikhar, Olayinka Stephen Ilesanmi, Muhana Fawwazy Ilyas, Javed Iqbal, Md Rabiul Islam, Ramzy Issa, Mahalaxmi Iyer, Haitham Jahrami, Abhishek Jaiswal, Mihajlo Jakovljevic, Ali Jaliliyan, Mohamed L Jalloh, Syed Sarmad Javaid, Talha Jawaid, Shubha Jayaram, Diptismita Jena, Wenyi Jin, Jost B Jonas, Tamas Joo, Jobin Jose, Abel Joseph, Charity Ehimwenma Joshua, Billingsley Kaambwa, Zubair Kabir, Dler Hussein Kadir, Leila R Kalankesh, Sivesh Kathir Kamarajah, Ramat T. Kamorudeen, André Karch, Mohammad Amin Karimi, Tomasz M Karpiński, Faizan Zaffar Kashoo, Mohd Adnan Kausar, Foad Kazemi, Ariz Keshwani, Emmanuelle Kesse-Guyot, Yousef Saleh Khader, Himanshu Khajuria, Hazim O. Khalifa, Pantea Khalili, Faham Khamesipour, Ajmal Khan, Moien AB Khan, Salman Ali Khan, Mahalaqua Nazli Khatib, Feriha Fatima Khidri, Atulya Aman Khosla, Jagdish Khubchandani, Yun Jin Kim, Adnan Kisa, Sezer Kisa, Shivakumar KM, Jonathan M Kocarnik, Michail Kokkorakis, Sindhura Lakshmi Koulmane Laxminarayana, Irene Akwo Kretchy, Kewal Krishan, Raja Amir Hassan Kuchay, Dewesh Kumar, G Anil Kumar, Vijay Kumar, Satyajit Kundu, Ambily Kuttikkattu, Ville Kytö, Muhammad Awwal Ladan, Chandrakant Lahariya, Dharmesh Kumar Lal, Savita Lasrado, Mahrukh Latif, Huu-Hoai Le, Minh Huu Nhat Le, Nhi Huu Hanh Le, Thao Thi Thu Le, Caterina Ledda, Sang-woong Lee, Seung Won Lee, Shaun Wen Huey Lee, Wei-Chen Lee, Awol Yemane Legesse, James Leigh, Wang-Zhong Li, Zhengrui Li, Stephen S Lim, Queran Lin, Gang Liu, Jue Liu, Xuefeng Liu, Erand Llanaj, Miltiadis D Lytras, Ellina Lytvyak, Zheng Feei Ma, Nozad Hussein Mahmood, Rashidul Alam Mahumud, Hadi Maleki-Kakelar,

Kashish Malhotra, Ahmad Azam Malik, Lesibana Anthony Malinga, Deborah Carvalho Malta, Mohammad Ali Mansournia, Roy Rillera Marzo, Sammer Marzouk, Medha Mathur, Indu Liz Matthew, Steven M McPhail, Enkeleint A Mechili, Riffat Mehboob, Asim Mehmood, Tesfahun Mekene Meto, Walter Mendoza, Ritesh G Menezes, Atte Meretoja, Awoke Misganaw, Prasanna Mithra, Yidnek Mogessie, Mona Gamal Mohamed, Nouh Saad Mohamed, Abdolreza Mohammadi, Mohammad Kazem Mohammadi, Seyed Omid Mohammadi, Abdollah Mohammadian-Hafshejani, Mustapha Mohammed, Shafiu Mohammed, Ali H Mokdad, Hossein Molavi Vardanjani, Sabrina Molinaro, Shaher Momani, Lorenzo Monasta, Yousef Moradi, Mahmoud M Morsy, Rohith Motappa, Rabia Mubarak, Sumaira Mubarik, George Duke Mukoro, Admir Mulita, Efren Murillo-Zamora, Christopher J L Murray, Saima Mushtaq, Sherzad Ibrahim Mustafa, Saravanan Muthupandian, Ahamarshan Jayaraman Nagarajan, Gabriele Nagel, Hae Sung Nam, Shumaila Nargus, Yvonne Nartey, Zuhair S Natto, Biswa Prakash Nayak, Masoud Negahdary, Ionut Negoi, Henok Biresaw Netsere, Cuong Tat Nguyen, Dang Nguyen, Kieu Viet Nhi Nguyen, Nhan Nguyen, Van Thanh Nguyen, Robina Khan Niazi, Luciano Nieddu, Chukwudi A Nnaji, Lawrence Achilles Nnyanzi, Shuhei Nomura, Syed Toukir Ahmed Noor, Masoud Noroozi, Fred Nugen, Chimezie Igwegbe Nzoputam, Ogochukwu Janet Nzoputam, Bogdan Oancea, Vincent Adeiza Obakachi, Ismail A Odetokun, James Odhiambo Oguta, Andrew T Olagunju, Matthew Idowu Olatubi, Folorunsho Bright Omage, Hany A Omar, Abidemi E Omonisi, Sok King Ong, Obinna E Onwujekwe, Marcel Opitz, Atakan Orscelik, Uchechukwu Levi Osuagwu, Oche Joseph Otorkpa, Stanislav S Otstavnov, Amel Ouyahia, Mayowa O Owolabi, Oladayo Ayobami Oyebanji, Mahesh P A, Jagadish Rao Padubidri, Sujogya Kumar Panda, Songhomitra Panda-Jonas, Seithikurippu R Pandi-Perumal, Leonidas D Panos, Mario Virgilio Papa, Parinaz Paranjkhoo, Romil R Parikh, Eun-Cheol Park, Maja Pasovic, Riya Jayesh Patel, Shankargouda Patil, Apurba Patra, Prince Peprah, Jeevan Pereira, Simone Perna, Hoang Tran Pham, Daniela Pierannunzio, Zahra Zahid Piracha, Ramesh Poluru, Arjun Pon Avudaiappan, Maarten J Postma, Reza Pourbabaki, Jalandhar Pradhan, Elton Junio Sady Prates, Dimas Ria Angga Pribadi, Natalie Pritchett, Jagadeesh Puvvula, Asma Saleem Qazi, Hadi Raeisi Shahraki, Pracheth Raghuveer, Hawbash Mohammed-Amin Rahim, Amir Masoud Rahmani, Masoud Rahmati, Diego Raimondo, Sathish Rajaa, Mohammad Amin Rajizadeh, Chitra Ramasamy, Shakthi Kumaran Ramasamy, Pushkal Sinduvadi Ramesh, Juwel Rana, Nemanja Rancic, Kumuda Rao, Sowmya J Rao, Santosh Kumar Rauniyar, Ilari Rautalin, Salman Rawaf, Lal Rawal, Elrashdy Redwan, Ana Reis-Mendes, Stefano Restaino, Nima Rezaei, Moattar Raza Rizvi, Hermano Alexandre Lima Rocha, Peter Rohloff, Amirhossein Roshanshad, Sharmistha Roy, Korosh Saber, Cameron John Sabet, Siamak Sabour, Basema Ahmad Saddik, Tarannom Sadegh, Umar Saeed, Narjes Saheb Sharif-Askari, Pragyan Monalisa Sahoo, Mirza Rizwan Sajid, Amir Salek Farrokhi, Marwa Rashad Salem, Abdallah M Samy, Juan Sanabria, Milena M Santric-Milicevic, Saman Sargazi, Tanmay Sarkar, Brijesh Sathian, Maheswar Satpathy, Reza Sattarpour, Monika Sawhney, Christophe Schinckus, Ione Jayce Ceola Schneider, Saravanan Sekaran, Mario Šekerija, Yigit Can Senol, Subramanian Senthilkumaran, Yashendra Sethi, Homa Seyedmirzaei, Allen Seylani, Muhammad Shahab, Samiah Shahid, Masood Ali Shaikh, Muhammad Aaqib Shamim, Mehran Shams-Beyranvand, Anas Shamsi, Alfiya Shamsutdinova, Dan Shan, Mohammed Shannawaz, Amin Sharifan, Vishal Sharma, Shamee Shastry, Ramzi Shawahna, Maryam Shayan, Samendra P Sherchan, Premalatha K Shetty, Aminu Shittu, Zahra Shokati Eshkiki, Sunil Shrestha, Ahmed Kamal Siddiqi, Luís Manuel Lopes Rodrigues Silva, Amit Singh, Baljinder Singh, Harmanjit Singh, Jasvinder A Singh, Kalpana Singh, Paramdeep Singh, Prashant Kumar Singh, Anna Aleksandrovna Skryabina, Solikhah Solikhah, Aayushi Sood, Sandra Spearman, Bahadar S Srichawla, Devin Bailey Srivastava, Vetriselvan Subramaniyan, Muhammad Suleman, Anusha Sultan Meo, Chandan Kumar Swain, Lukasz Szarpak, Sree Sudha T Y, Rafael Tabarés-Seisdedos, Seyyed Mohammad Tabatabaei, Ramin Tabibi, Yasaman Taheri Abkenar, Jabeen Taiba, Mircea Tampa, Ker-Kan Tan, Manoj Tanwar, Birhan Tsegaw Taye, Abdelghani Tbakhi,

Abainash Tekola, Mohamad-Hani Temsah, Dufera Rikitu Terefa, Mahalakshmi Thayumana Sundaram, Nihal Thomas, Nikhil Kenny Thomas, Krishna Tiwari, Adetunji T Toriola, Mathilde Touvier, Marcos Roberto Tovani-Palone, Bach Xuan Tran, Ngoc Ha Tran, Quynh Thuy Huong Tran, Domenico Trico, Abdul Rohim Tualeka, Srikanth Umakanthan, Bhaskaran Unnikrishnan, Dinesh Upadhya, Jef Van den Eynde, Shoban Babu Varthya, Balachandar Vellingiri, Georgios-Ioannis Verras, Sergey Konstantinovitch Vladimirov, Vasily Vlassov, Theo Vos, Yasir Waheed, Kosala Gayan Weerakoon, Dakshitha Praneeth Wickramasinghe, Peter Willeit, Marcin W Wojewodzic, Axel Walter Wolf, Felicia Wu, Zenghong Wu, Suowen Xu, Mingyang Xue, Amir Yarahmadi, Sanni Yaya, Pengpeng Ye, Subah Abderehim Yesuf, Naohiro Yonemoto, Mustafa Z Younis, Chuanhua Yu, Vesna Zadnik, Syed Nabeel Zafar, Michael Zastrozhin, Mohammed G M Zeariya, Sebastian Zensen, Jingya Zhang, Xiaoyi Zhang, David X Zheng, Jinxin Zheng, Anthony Zhong, Abzal Zhumagaliuly, Magdalena Zielińska, Rafat Mohammad Zrieq, Sa'ed H Zyoud.

Developing methods or computational machinery

Aleksandr Y Aravkin, Kayleigh Bhangdia, Michael Brauer, Katrin Burkart, Andrew Crist, Xiaochen Dai, Lisa M Force, Konstantinos Giannakis, Simon I Hay, Jonathan M Kocarnik, Ali H Mokdad, Christopher J L Murray, Maja Pasovic, Louise Penberthy, Natalie Pritchett, Austin E Schumacher, Sandra Spearman, Stein Emil Vollset, Theo Vos, Chun-Wei Yuan

Providing critical feedback on methods or results

Bhoomadevi A, Nasir Abbas, Maryam Abbasalipour Bashash, Abdallah H A Abd Al Magied, Samar Abd ElHafeez, Mohammed Altigani Abdalla, Parsa Abdi, Wakgari Mosisa Abdisa, Daba Abdissa, Arash Abdollahi, Meriem Abdoun, Arman Abdous, Auwal Abdullahi, Mesfin Abebe, Armita Abedi, Syed Hani Abidi, Alemwork Abie, Richard Gyan Aboagye, Hassan Abolhassani, Ulric Sena Abonie, Lucas Guimarães Abreu, Bilyaminu Abubakar, Eman Abu-Gharbieh, Hana J Abukhadijah, Salahdein Aburuz, Ahmed Abu-Zaid, Raghu Ram Achar, Juan Manuel Acuna, Lisa C Adams, Lawan Hassan Adamu, Isaac Yeboah Addo, Kamoru Ademola Adedokun, Nurudeen A Adegoke, Victor Adekanmbi, Habeeb Omoponle Adewuyi, Usha Adiga, Qorinah Estiningtyas Sakilah Adnani, Leticia Akua Adzigbli, Ahmed M Afifi, Aanuoluwapo Adeyimika Afolabi, Fatemeh Afrashteh, Muhammad Sohail Afzal, Navidha Aggarwal, Feleke Doyore Agide, César Agostinis Sobrinho, Williams Agyemang-Duah, Bright Opoku Ahinkorah, Aqeel Ahmad, Danish Ahmad, Faisal Ahmad, Khurshid Ahmad, Muayyad M Ahmad, Sajjad Ahmad, Tauseef Ahmad, Amir Mahmoud Ahmadzade, Mohadese Ahmadzade, Anisuddin Ahmed, Ayman Ahmed, Gasha Salih Ahmed, Luai A Ahmed, Mehrunnisha Sharif Ahmed, Muktar Beshir Ahmed, Nesredin Ahmed, Syed Anees Ahmed, Dolapo Emmanuel Ajala, Roland Eghoghosoa Akhigbe, Karolina Akinosoglou, Salah Al Awaidy, Hanadi Al Hamad, Syed Mahfuz Al Hasan, Omar Al Omari, Mohammad Al Qadire, Yazan Al Thaher, Omar Ali Mohammed Al Zaabi, Khurshid Alam, Zufishan Alam, Amani Alansari, Fahmi Y Al-Ashwal, Laila Ismael Al-Daken, Shereen M Aleidi, Bezawit Abeje Alemayehu, Abdelazeem M Algammal, Adel Ali Saeed Al-Gheethi, Mohammed Khaled Al-Hanawi, Abid Ali, Endale Alemayehu Ali, Mohammad Daud Ali, Mohammed Usman Ali, Rafat Ali, Syed Shujait Ali, Waad Ali, Sheikh Mohammad Alif, Samah W Al-Jabi, Syed Mohamed Aljunid, Ahmad Alkhatib, Hesham M Al-Mekhlafi, Hasan Yaser Alniss, Margret Beaula Alocious Sukumar, Mahmoud A Alomari, Mohammad R Alosta, Saleh A Alqahtani, Ahmad Rajeh Al-Qudimat, Rajaa M Mohammad Al-Raddadi, Intima Alrimawi, Salman Khalifah Al-Sabah, Najim Z. Alshahrani, Awais Altaf, Alaa B Al-Tammemi, Elysia M Alvarez, Nelson Alvis-Guzman, Yaser Mohammed Al-Worafi, Hany Aly, Mohammad Sharif Ibrahim Alyahya, Abdallah Alzoubi, Karem H Alzoubi, Md. Akib

Al-Zubayer, Masoud Aman Mohammadi, Tarek Tawfik Amin, Alireza Amindarolzarbi, Nafiu Aminu, Hubert Amu, Ganiyu Adeniyi Amusa, Robert Ancuceanu, Deanna Anderlini, Amir Anoushiravani, Hossein Ansariniya, Iyadunni Adesola Anuoluwa, Saeid Anvari, Saleha Anwar, Sumadi Lukman Anwar, Razique Anwer, Ekenedilichukwu Emmanuel Anyabolo, Anayochukwu Edward Anyasodor, Angela Esi Apeagyei, Jalal Arabloo, Elshaimaa A Arafa, Mosab Arafat, Demelash Areda, Hany Ariffin, Mesay Arkew, Anton A Artamonov, Santhosh Arul, Deepavalli Arumuganainar, Mohammad Asghari-Jafarabadi, Akram Ashames, Muhammad Abdul Basit Ashraf, Tahira Ashraf, Bernard Kwadwo Yeboah Asiamah-Asare, Haftu Asmerom Asmerom, Batyrbek Assembekov, Prince Atorkey, Maha Moh'd Wahbi Atout, Julie Alaere Atta, Marcel Ausloos, Atalel Fentahun Awedew, Adedapo Wasiu Awotidebe, Beatriz Paulina Ayala Quintanilla, Haleh Ayatollahi, Ali Azargoonjahromi, Amirali Azimi, Mohd Yusmaidie Aziz, Sadat Abdulla Aziz, Shahkaar Aziz, Hosein Azizi, Ahmed Y Azzam, Muhammad Badar, Ashish D Badiye, Alaa Aboelnour Badran, Nasser Bagheri, Fereshteh Baghizadeh, Razieh Bahreini, Ruhai Bai, Atif Amin Baig, Lovenish Bains, Ram Chandra Bajpai, Shankar M Bakkannavar, Auwal Adam Bala, Wondu Feyisa Balcha, Mohammadreza Balooch Hasankhani, Soham Bandyopadhyay, Rajon Banik, Aleksandra Barac, Mainak Bardhan, Hiba Jawdat Barqawi, Amadou Barrow, Zarrin Basharat, Shahid Bashir, Pritish Baskaran, Mohammad-Mahdi Bastan, Kavita Batra, Matteo Bauckneht, Mohsen Bayati, Feyisa Shasho Bayisa, Narasimha M Beeraka, Amir Hossein Behnoush, Diana Fernanda Bejarano Ramirez, Sewunet admasu Belachew, Gokce Belge Bilgin, Habib Benzian, Alemshet Yirga Yirga Berhie, Amiel Nazer C Bermudez, Ajeet Singh Bhadoria, Akshaya Srikanth Bhagavathula, Nickhill Bhakta, Neeraj Bhala, Kayleigh Bhangdia, Ravi Bharadwaj, Nikha Bhardwaj, Pankaj Bhardwaj, Sonu Bhaskar, Ajay Nagesh Bhat, Vivek Bhat, Shuvarthi Bhattacharjee, Gurjit Kaur Bhatti, Jasvinder Singh Bhatti, Mohiuddin Ahmed Bhuiyan, Soumitra S Bhuyan, Raluca Bievel-Radulescu, Cem Bilgin, Mohammad Shahangir Biswas, Monirujjaman Biswas, Bruno Bizzozero-Peroni, Trupti Bodhare, Obasanjo Afolabi Bolarinwa, Archith Boloor, Hamed Borhany, Alejandro Botero Carvajal, Souad Bouaoud, Marija M Bozic, Dejana Braithwaite, Hermann Brenner, Linh Phuong Bui, Katrin Burkart, Felix Busch, Maria Teresa Bustamante-Teixeira, Yasser Bustanji, Nadeem Shafique Butt, Zahid A Butt, Guangyao Cai, Luis Alberto Cámera, Ismael Campos-Nonato, Si Cao, Yubin Cao, Angelo Capodici, Márcia Carvalho, Ferrán Catalá-López, Luca Cegolon, Francieli Cembranel, Ester Cerin, Sonia Cerrai, Aditya Chakraborty, Chiranjib Chakraborty, Vijay Kumar Chattu, Sirshendu Chaudhuri, Akhilanand Chaurasia, Galmesa Bekana Chemeda, Hana Chen, Kai Chen, Meng Xuan Chen, Fatemeh Chichagi, Odgerel Chimed-Ochir, Jesus Lorenzo Chirinos-Caceres, William C S Cho, Dong-Woo Choi, Sungchul Choi, Bryan Chong, Yuen Yu Chong, Hitesh Chopra, Shivani Chopra, Hou In Chou, Sonali Gajanan Choudhari, Dinh-Toi Chu, Isaac Sunday Chukwu, Eric Chung, Sunghyun Chung, Iolanda Cioffi, Alyssa Columbus, Joao Conde, Andrew Crist, Natalia Cruz-Martins, Bashir Dabo, Omid Dadras, Xiaochen Dai, Lalit Dandona, Rakhi Dandona, Lucio D'Anna, Pojsakorn Danpanichkul, Reza Darvishi Cheshmeh Soltani, Aso Mohammad Darwesh, Saswati Das, Nihar Ranjan Dash, Mohsen Dashti, Cornelius Dassah, Dimash Davletov, Fernando Pio De la Hoz, Frances E Dean, Edward Christopher Dee, Sindhura Deekonda, Mohammad Delsoz, Abel Desalegn Demeke, Rupak Desai, Aragaw Tesfaw Desale, Gashaw Dessie, Devananda Devegowda, Arkadeep Dhali, Amol S Dhane, Meghnath Dhimal, Bibha Dhungel, Marcello Di Pumpo, Michael J Diaz, Thao Huynh Phuong Do, Saeid Doaei, Fariba Dorostkar, Wendel Mombaque dos Santos, Ojas Prakashbhai Doshi, Leila Doshmangir, Taiwo Omotayo Dosumu, Robert Kokou Dowou, Senbagam Duraisamy, Oyewole Christopher Durojaiye, Sulagna Dutta, Arkadiusz Marian Dziedzic, Abdel Rahman E'mar, Hisham Atan Edinur, Ashkan Eighaei Sedeh, Michael Ekholuenetale, Temitope Cyrus Ekundayo, Rabie Adel El Arab, Abdelfatteh EL Omri, Iman El Sayed, Maysaa El Sayed Zaki, Reza Elahi, Noha Mousaad Elemam, Muhammed Elhadi, Waseem El-Huneidi, Sherif Elkannishy, Ahmed O Elmehrath, Adel B

Elmoselhi, Randa Elsheikh, Yasser Mohamed El-Sherbiny, Ibrahim Elsohaby, Abdelgawad Salah Abdelgawad Eltahawy, Mohd Elmagzoub Eltahir, Syed Emdadul Haque, Theophilus I Emeto, Talha Bin Emran, Babak Eshrati, Majid Eslami, Heidar Fadavian, Adeniyi Francis Fagbamigbe, Ayesha Fahim, Saman Fahimi, Aamir Fahira, Ildar Ravisovich Fakhradiyev, Aliasghar Fakhri-Demeshghieh, Luca Falzone, Alireza Farahani, Mohammad Farahmand, Seyed Nooreddin Faraji, Mahsa Faramarzpour, Ebrahim Farashi, Zaki Farhana, Pawan Sirwan Faris, Andre Faro, Umar Farooque, Hossein Farrokhpour, Abidemi Omolara Fasanmi, Kiana Fasihi, Alireza Feizkhah, Ginenus Fekadu, Daniela Ferrante, Getahun Fetensa, Florian Fischer, James L Fisher, Bobirca Teodor Florin, Lisa M Force, Arianna Fornari, Behzad Foroutan, Maryam Fotouhi, Takeshi Fukumoto, Ami Fukunaga, Muktar A Gadanya, Márió Gajdács, Adamu Usman Gamawa, Aravind P Gandhi, Mounika Gangireddy, Jacopo Garlasco, Rupesh K Gautam, Miglas Welay Gebregergis, Mesfin Gebrehiwot, Teferi Gebru Gebremeskel, Haftay Gebremedhin Gebreslassie, Gebremariam Wulie Geremew, Genanew K Getahun, Amir Ghaffari Jolfayi, Lobna Faiz Gharaibeh, Fariba Ghassemi, Ramy Mohamed Ghazy, Maryam Gholamalizadeh, Elena Ghotbi, Konstantinos Giannakis, Syed Abdullah Gilani, Alem Abera Girmay, Alessandro Girombelli, Amit Goel, Anil Kumar Goel, Mahaveer Golechha, MReza Goodarzian, Changhao Gu, Shi-Yang Guan, Mohammed Ibrahim Mohialdeen Gubari, Zheng Guo, Zhifeng Guo, Bhawna Gupta, Sapna Gupta, Reyna Alma Gutiérrez, Jose Guzman-Esquivel, Adrina Habibzadeh, Farrokh Habibzadeh, Parham Habibzadeh, Tesfahun Simon Simon Hadaro, Najah R Hadi, Dariush Haghmorad, Nguyen Hai Nam, Haimanot Ewnetu Hailu, Hassen Mosa Mosa Halil, Islam M Hamad, Randah R Hamadeh, Nadia M Hamdy, Nasrin Hanifi, Ashanul Haque, Ahmed I Hasaballah, Md Kamrul Hasan, S. M. Mahmudul Hasan, Fatemeh Hasani, Hamidreza Hasani, Alireza Hasanzadeh, Abdiwahab Hashi, Md Saquib Hasnain, Ikrama Ibrahim Hassan, Treska S Hassan, Mahgol Sadat Hassan Zadeh Tabatabaei, Johannes Haubold, Simon I Hay, Khezar Hayat, Qiang He, Wen-Qiang He, Behzad Heibati, Mohammad Heidari, Claudiu Herteliu, Hamed Hesami, Majid Heydari, Mojtaba Heydari, Zahra Heydarifard, Demisu Zenbaba Heyi, Kamal Hezam, Yuta Hiraike, Mai Hoang, Ramesh Holla, Nobuyuki Horita, Mehdi Hoseinzadeh, H Dean Hosgood, Alamgir Hossain, Md Mahbub Hossain, Md Sabbir Hossain, Mohammad Bellal Hossain, Mohammad-Salar Hosseini, Mihaela Hostiuc, Chengxi Hu, Junjie Hu, Junjie Huang, Kiavash Hushmandi, Javid Hussain, Salman Hussain, Dursa Hussein, Nawfal R Hussein, Mohamed Ibrahim Husseiny, Hong-Han Huynh, Segun Emmanuel Ibitoye, Reem Ibrahim, Francisco Javier Idalsoaga, Olayinka Stephen Ilesanmi, Irena M Ilic, Milena D Ilic, Muhana Fawwazy Ilyas, Salim Ilyasu, Mohammad Tarique Imam, Mustapha Immurana, Lucius Chidiebere Imoh, Javed Iqbal, Mustafa Alhaji Isa, Ramzy Issa, Masao Iwagami, Chidozie Declan Iwu, Mahalaxmi Iyer, Louis Jacob, Farhad Jadidi-Niaragh, Haitham Jahrami, Ayushi Jain, Ammar Abdulrahman Jairoun, Abhishek Jaiswal, Mihajlo Jakovljevic, Ali Jaliliyan, Reza Jalilzadeh Yengejeh, Mohamed L Jalloh, Safayet Jamil, Syed Sarmad Javaid, Umesh Jayarajah, Shubha Jayaram, Ruwan Duminda Jayasinghe, Sun Ha Jee, Diptismita Jena, Shuai Jin, Wenyi Jin, Mohammad Jokar, Jost B Jonas, Tamas Joo, Jobin Jose, Abel Joseph, Nitin Joseph, Charity Ehimwenma Joshua, Farahnaz Joukar, Mikk Jürisson, Malik E Juweid, Anupam Jyoti, Billingsley Kaambwa, Ali Kabir, Zubair Kabir, Maryam Kabiri, Dler Hussein Kadir, Ebbie Kalan, Leila R Kalankesh, Mehnaz Kamal, Vineet Kumar Kamal, Sivesh Kathir Kamarajah, Ramat T. Kamorudeen, Fatemeh Kanaani Nejad, Samuel Berchi Kankam, Kehinde Kazeem Kanmodi, Neeti Kapoor, Mehrdad Karajizadeh, André Karch, Mohammad Amin Karimi, Sahand Karimzadhagh, Tomasz M Karpiński, Faizan Zaffar Kashoo, Joonas H Kauppila, Navjot Kaur, Mohd Adnan Kausar, Foad Kazemi, Fekadu Abera Kebede, Ariz Keshwani, Emmanuelle Kesse-Guyot, Yousef Saleh Khader, Himanshu Khajuria, Asaad Khalid, Hazim O. Khalifa, Pantea Khalili, Alireza Khalilian, Ghazaleh Khalili-Tanha, Mohamed khalis, Faham Khamesipour, Ajmal Khan, Faiz Ullah Khan, Mohammad Jobair Khan, Moien AB Khan, Salman Ali Khan, Shaghayegh

Khanmohammadi, Moawiah Mohammad Khatatbeh, Mahalaqua Nazli Khatib, Maryam Khayamzadeh, Daniel Kheradmand, Feriha Fatima Khidri, Atulya Aman Khosla, Mohammad Ali Khosravi, Jagdish Khubchandani, Kwanghyun Kim, Yun Jin Kim, Ruth W Kimokoti, Adnan Kisa, Sezer Kisa, Shivakumar KM, Jonathan M Kocarnik, Michail Kokkorakis, Ali-Asghar Kolahi, Farzad Kompani, Jianqiu Kong, Anastasios Georgios Panagiotis Konstas, Ashwin Laxmikant Kotnis, Sindhura Lakshmi Koulmane Laxminarayana, Irene Akwo Kretchy, Kewal Krishan, Mohammed Kuddus, Dewesh Kumar, G Anil Kumar, Nithin Kumar, Vijay Kumar, Satyajit Kundu, Setor K Kunutsor, Pramod Kumar Kushawaha, Christina Yeni Yeni Kustanti, Tezer Kutluk, Ambily Kuttikkattu, Assylkhan Kuttybayev, Grace Kwakyewaa Kyei, Frank Kyei-Arthur, Ville Kytö, Carlo La Vecchia, Muhammad Awwal Ladan, Chandrakant Lahariya, Daphne Teck Ching Lai, Hanpeng Lai, Dharmesh Kumar Lal, Judit Lám, Francesco Lanfranchi, Savita Lasrado, Mahrukh Latif, Paolo Lauriola, Basira Kankia Lawal, Huu-Hoai Le, Minh Huu Nhat Le, Nhi Huu Hanh Le, Thao Thi Thu Le, Caterina Ledda, Ivan Lee, Sang-woong Lee, Seung Won Lee, Wei-Chen Lee, Awol Yemane Legesse, James Leigh, Elvynna Leong, Ming-Chieh Li, Wang-Zhong Li, Wei Li, Xiaopan Li, Yongze Li, Zhengrui Li, Zhihui Li, Stephen S Lim, Queran Lin, Chaojie Liu, Gang Liu, Jue Liu, Liu Liu, Xiaofeng Liu, Xuefeng Liu, Erand Llanaj, Jailos Lubinda, Angelina M Lutambi, Miltiadis D Lytras, Ellina Lytvyak, Hawraz Ibrahim M. Amin, Zheng Feei Ma, Farzan Madadizadeh, Alireza Mafi, Azzam A Maghazachi, Abdulahi Abdiwali Mahamed, Nozad Hussein Mahmood, Rashidul Alam Mahumud, Azeem Majeed, Abdelrahman M Makram, Omar M Makram, Konstantinos Christos C. Makris, Hadi Maleki-Kakelar, Kashish Malhotra, Ahmad Azam Malik, Iram Malik, Lesibana Anthony Malinga, Deborah Carvalho Malta, Vahid Mansouri, Marjan Mansourian, Mohammad Ali Mansournia, Lorenzo Giovanni Mantovani, Hamid Reza Marateb, Basma Hamed Marghani, Mirko Marino, Santi Martini, Miquel Martorell, Roy Rillera Marzo, Sammer Marzouk, Yasith Mathangasinghe, Medha Mathur, Fernanda Penido Matozinhos, Indu Liz Matthew, Martin McKee, Steven M McPhail, Enkeleint A Mechili, Jitendra Kumar Meena, Elahe Meftah, Riffat Mehboob, Asim Mehmood, Tesfahun Mekene Meto, Hadush Negash Meles, Addisu Melese, Max Alberto Mendez-Lopez, Walter Mendoza, Ritesh G Menezes, Dominik Menges, Tara P Menon, Sultan Ayoub Ayoub Meo, Atte Meretoja, Tuomo J Meretoja, Tomislav Mestrovic, Tomasz Miazgowski, Irmina Maria Michalek, Ted R Miller, Giuseppe Minervini, Awoke Misganaw, Sanjeev Misra, Prasanna Mithra, Yidnek Mogessie, Jama Mohamed, Mona Gamal Mohamed, Nouh Saad Mohamed, Taj Mohammad, Sakineh Mohammad-Alizadeh-Charandabi, Abdolreza Mohammadi, Mohammad Kazem Mohammadi, Seyed Omid Mohammadi, Abdollah Mohammadian-Hafshejani, Ibrahim Mohammadzadeh, Zeinab Mohammadzadeh, Al-Kassim Hassan Mohammed, Mustapha Mohammed, Shafiu Mohammed, Amin Mohsenzadeh, Ali H Mokdad, Hossein Molavi Vardanjani, Sabrina Molinaro, Shaher Momani, Lorenzo Monasta, Mohammad Ali Moni, Yousef Moradi, Paula Moraga, Shane Douglas Morrison, Mahmoud M Morsy, Elias Mossialos, Fatemeh Motaharinezhad, Rohith Motappa, Kimia Mozahheb Yousefi, Ahmed Msherghi, Rabia Mubarak, Sumaira Mubarik, George Duke Mukoro, Admir Mulita, Kavita Munjal, Efren Murillo-Zamora, Christopher J L Murray, Ali Mushtaq, Saima Mushtaq, Saravanan Muthupandian, Woojae Myung, Amin Nabavi, Ahamarshan Jayaraman Nagarajan, Gabriele Nagel, Pirouz Naghavi, Gurudatta Naik, Mukhammad David Naimzada, Firzan Nainu, Tapas Sadasivan Nair, Hastyar Hama Rashid Najmuldeen, Shumaila Nargus, Yvonne Nartey, Abdulqadir J Nashwan, Mahmoud Nassar, Zuhair S Natto, Javaid Nauman, Biswa Prakash Nayak, Md Fahad Shahariar Nayon, Athare Nazri-Panjaki, Pacifique Ndishimye, Amanuel Tebabal Nega, Masoud Negahdary, Ionut Negoi, Chakib Nejjari, Omid Nekouei, Gaurav Nepal, Henok Biresaw Netsere, Jean Claude Semuto Semuto Ngabonziza, Cuong Tat Nguyen, Dang Nguyen, Kieu Viet Nhi Nguyen, Nhan Nguyen, The Phuong Nguyen, Tuan Thanh Nguyen, Van Thanh Nguyen, Robina Khan Niazi, Luciano Nieddu, Nasrin Nikravangolsefid, Vikram Niranjan, Jean

Marie Vianney Niyonsenga, Chukwudi A Nnaji, Lawrence Achilles Nnyanzi, Shuhei Nomura, Nurulamin M Noor, Syed Toukir Ahmed Noor, Masoud Noroozi, Nawsherwan NOT APPLICABLE, Chisom Adaobi Nri-Ezedi, Fred Nugen, Dieta Nurrika, Chimezie Igwegbe Nzoputam, Ogochukwu Janet Nzoputam, Bogdan Oancea, Vincent Adeiza Obakachi, Ismail A Odetokun, Michael Safo Oduro, James Odhiambo Oguta, In-Hwan Oh, Hassan Okati-Aliabad, Akinkunmi Paul Okekunle, Osaretin Christabel Okonji, Andrew T Olagunju, Matthew Idowu Olatubi, Folorunsho Bright Omage, Hany A Omar, Goran Latif Omer, Abidemi E Omonisi, Obinna E Onwujekwe, Marcel Opitz, Michal Ordak, Atakan Orscelik, Samuel M Ostroff, Uchechukwu Levi Osuagwu, Elham H Othman, Oche Joseph Otorkpa, Stanislav S Otstavnov, Amel Ouyahia, Mayowa O Owolabi, Oladayo Ayobami Oyebanji, Kolapo Oyebola, Ilker Ozsahin, Mahesh P A, Jagadish Rao Padubidri, Tamás Palicz, Sujogya Kumar Panda, Songhomitra Panda-Jonas, Deepshikha Pande Katare, Seithikurippu R Pandi-Perumal, Ke Pang, Georgios D Panos, Leonidas D Panos, Ioannis Pantazopoulos, Mario Virgilio Papa, Parinaz Paranjkhoo, Peyvand Parhizkar Roudsari, Romil R Parikh, Eun-Cheol Park, Roberto Passera, Jay Patel, Riya Jayesh Patel, Shankargouda Patil, Apurba Patra, Jarmila Pekarcikova, Prince Peprah, Jeevan Pereira, Mario F P Peres, Richard G Pestell, Olumuyiwa James Peter, Fanny Emily Petermann-Rocha, Hoang Tran Pham, Tung Thanh Pham, Daniela Pierannunzio, Julian David Pillay, Zahra Zahid Piracha, Saeed Pirouzpanah, Ramesh Poluru, Arjun Pon Avudaiappan, Maarten J Postma, Reza Pourbabaki, Farzad Pourghazi, Disha Prabhu, Jalandhar Pradhan, Peralam Yegneswaran Prakash, Chandra P Prasad, Akila Prashant, Elton Junio Sady Prates, Natalie Pritchett, Jagadeesh Puvvula, Asma Saleem Qazi, Suli Qiu, Karzan Qurbani, Hadi Raeisi Shahraki, Ata Rafiee, Alireza Rafiei, Pracheth Raghuveer, Hawbash Mohammed-Amin Rahim, Fryad Majeed Rahman, Md Mijanur Rahman, Mohammad Meshbahur Rahman, Mosiur Rahman, Muhammad Aziz Rahman, Amir Masoud Rahmani, Saeed Rahmani, Masoud Rahmati, Pramila Rai, Diego Raimondo, Sathish Rajaa, Rayan Rajabi, Mohammad Amin Rajizadeh, Majed Ramadan, Chitra Ramasamy, Shakthi Kumaran Ramasamy, Pushkal Sinduvadi Ramesh, Zahra Ramezani, Juwel Rana, Rishabh Kumar Rana, Nemanja Rancic, Christopher Rao, Kumuda Rao, Mithun Rao, Sowmya J Rao, Mohammad-Mahdi Rashidi, Devarajan Rathish, Santosh Kumar Rauniyar, Ilari Rautalin, Salman Rawaf, Lal Rawal, Elrashdy Redwan, Sanika Rege, Ana Reis-Mendes, Stefano Restaino, Nazila Rezaei, Negar Rezaei, Nima Rezaei, Mohsen Rezaeian, Tércia Moreira Ribeiro da Silva, Maximiliano Ribeiro Ribeiro Guerra, Moattar Raza Rizvi, Thomas J Roberts, Hermano Alexandre Lima Rocha, João Rocha Rocha-Gomes, Thales Philipe Rodrigues da Silva, Ravi Rohilla, Peter Rohloff, Kevin T Root, Gholamreza Roshandel, Amirhossein Roshanshad, Himanshu Sekhar Rout, Sharmistha Roy, Korosh Saber, Maha Mohamed Saber-Ayad, Cameron John Sabet, Siamak Sabour, Basema Ahmad Saddik, Tarannom Sadegh, Mohammad Reza Saeb, Kanza Saeed, Mohd Saeed, Umar Saeed, Narjes Saheb Sharif-Askari, Pragyan Monalisa Sahoo, Soumya Swaroop Sahoo, Mirza Rizwan Sajid, Mohamed A Saleh, Amir Salek Farrokhi, Marwa Rashad Salem, Sohrab Salimi, Pouria Samadi, Jayami Eshana Samaranayake, Yoseph Leonardo Samodra, Abdallah M Samy, Juan Sanabria, Milena M Santric-Milicevic, Made Ary Sarasmita, Aswini Saravanan, Tanmay Sarkar, Gargi Sachin Sarode, Sachin C Sarode, Arash Sarveazad, Michele Sassano, Brijesh Sathian, Maheswar Satpathy, Reza Sattarpour, Jennifer Saulam, Monika Sawhney, Juhi Saxena, Christophe Schinckus, Ione Jayce Ceola Schneider, Art Schuermans, Saravanan Sekaran, Mario Šekerija, Mohammad H Semreen, Ashenafi Kibret Sendekie, Pallav Sengupta, Yigit Can Senol, Subramanian Senthilkumaran, Dragos Serban, Yashendra Sethi, Seyed Mohammad Seyed Alshohadaei, Homa Seyedmirzaei, Maryam Shabany, Mahan Shafie, Muhammad Shahab, Ataollah Shahbandi, Samiah Shahid, Syed Ahsan Ahsan Shahid, Hamid R Shahsavari, Masood Ali Shaikh, Muhammad Aaqib Shamim, Elahe Shams, Mehran Shams-Beyranvand, Anas Shamsi, Dan Shan, Mohd Shanawaz, Abhishek Shankar, Mohammed Shannawaz, Nigussie Tadesse Sharew, Amin Sharifan,

Rajesh Sharma, Ujjawal Sharma, Vishal Sharma, Shamee Shastry, Ramzi Shawahna, Maryam Shayan, Fateme Sheida, Samendra P Sherchan, Ranjitha S Shetty, Mosa Shibani, Aminu Shittu, Sina Shool, Seyed Afshin Shorofi, Sunil Shrestha, Kerem Shuval, Yafei Si, Nicole R S Sibuyi, Emmanuel Edwar Siddig, Ahmed Kamal Siddiqi, Luís Manuel Lopes Rodrigues Silva, Amit Singh, Baljinder Singh, Harmanjit Singh, Jasvinder A Singh, Kalpana Singh, Mayank Singh, Paramdeep Singh, Prashant Kumar Singh, Satwinder Singh, Mukesh Kumar Sinha, Freddy Sitas, Anna Aleksandrovna Skryabina, Amanda E Smith, Solikhah Solikhah, Sameh S M Soliman, Aayushi Sood, Bahadar S Srichawla, Devin Bailey Srivastava, Kurt Straif, Vetriselvan Subramaniyan, Muritala Odidi Suleiman Odidi, Muhammad Suleman, Desy Sulistiyorini, Anusha Sultan Meo, Zhong Sun, Vinay Suresh, Chandan Kumar Swain, Lukasz Szarpak, Sree Sudha T Y, Payam Tabaee Damavandi, Rafael Tabarés-Seisdedos, Fatemeh Sadat Tabatabaei, Seyyed Mohammad Tabatabaei, Ramin Tabibi, Mohammad Tabish, Seyed Reza Taha, Yasaman Taheri Abkenar, Moslem Taheri Soodejani, Jabeen Taiba, Iman M Talaat, Mircea Tampa, Jacques Lukenze Tamuzi, Ker-Kan Tan, Manoj Tanwar, Saba Tariq, Seyed Mohammad Tavangar, Birhan Tsegaw Taye, Abdelghani Tbakhi, Abainash Tekola, Mohamad-Hani Temsah, Dufera Rikitu Terefa, Enoch Teye-Kwadjo, Lakshmi Thangavelu, Rekha Thapar, Rasiah Thayakaran, Mahalakshmi Thayumana Sundaram, Hadiza Theyra-Enias, Nihal Thomas, Nikhil Kenny Thomas, Jansje Henny Vera Ticoalu, Tenaw Yimer Tiruye, Roman Topor-Madry, Adetunji T Toriola, Mathilde Touvier, Marcos Roberto Tovani-Palone, Eugenio Traini, Bach Xuan Tran, Ngoc Ha Tran, Quynh Thuy Huong Tran, Domenico Trico, Mike Tuffour Amirikah, Lawrence Sena Tuglo, Atta Ullah, Saeed Ullah, Srikanth Umakanthan, Shehu Salihu Umar, Umar Muhammad Umar, Bhaskaran Unnikrishnan, Dinesh Upadhya, Omid Vakili, Jef Van den Eynde, Shoban Babu Varthya, Siavash Vaziri, Balachandar Vellingiri, Madhur Verma, Massimiliano Veroux, Georgios-Ioannis Verras, Simone Villa, Gabriela Ines Villanueva, Francesco S Violante, Giuseppe Vizzielli, Stein Emil Vollset, Theo Vos, Elpida Vounzoulaki, Yasir Waheed, Cong Wang, Ruixuan Wang, Xing Wang, Yanzhong Wang, Muhammad Waqas, Paul Ward, Toyiba Hiyaru Wassie, Kosala Gayan Weerakoon, Ronny Westerman, Anggi Lukman Wicaksana, Dakshitha Praneeth Wickramasinghe, Nuwan Darshana Darshana Wickramasinghe, Karn Wijarnpreecha, Peter Willeit, Marcin W Wojewodzic, Axel Walter Wolf, Felicia Wu, James Fan Wu, Qing Xia, Zhijia Xia, Lishun Xiao, Wanqing Xie, Suowen Xu, Mingyang Xue, Mukesh Kumar Yadav, Galal Yahya, Amir Yarahmadi, Mohamed A Yassin, Sanni Yaya, Pengpeng Ye, Subah Abderehim Yesuf, Saber Yezli, Dehui Yin, Biksegn Asrat Yirdaw, Dong Keon Yon, Naohiro Yonemoto, Mustafa Z Younis, Chuanhua Yu, Vesna Zadnik, Syed Nabeel Zafar, Manijeh Zaghampour, Fathiah Zakham, Nazar Zaki, Giulia Zamagni, Burhan Abdullah Zaman, Sojib Bin Zaman, Mohammad Javad Zare Sakhvidi, Michael Zastrozhin, Mohammed Zawiah, Mohammed G M Zeariya, Sebastian Zensen, Jianrong Zhang, Jingya Zhang, Liqun Zhang, Xiaoyi Zhang, Yang Zhao, David X Zheng, Jinxin Zheng, Ming-Hua Zheng, Anthony Zhong, Claire Chenwen Zhong, Jiayan Zhou, Juexiao Zhou, Bin Zhu, Magdalena Zielińska, Osama A Zitoun, Rafat Mohammad Zrieq, Sa'ed H Zyoud, Shaher H Zyoud

Drafting the work or revising it critically for important intellectual content

Bhoomadevi A, Hasan Aalruz, Maryam Abbasalipour Bashash, Abdallah H A Abd Al Magied, Samar Abd ElHafeez, Ashraf Nabiel Abdalla, Wael M Abdel-Rahman, Parsa Abdi, Wakgari Mosisa Abdisa, Daba Abdissa, Arman Abdous, Auwal Abdullahi, Armita Abedi, Olumide Abiodun, Hassan Abolhassani, Ulric Sena Abonie, Lucas Guimarães Abreu, Aminu Kende Abubakar, Bilyaminu Abubakar, Eman Abu-Gharbieh, Hana J Abukhadijah, Salahdein Aburuz, Ahmed Abu-Zaid, Raghu Ram Achar, Juan Manuel Acuna, Lawan Hassan Adamu, Isaac Yeboah Addo, Kamoru Ademola Adedokun, Nurudeen A Adegoke,

Victor Adekanmbi, Ibukun Modupe Adesiyan, Habeeb Omoponle Adewuyi, Usha Adiga, Qorinah Estiningtyas Sakilah Adnani, Prince Owusu Adoma, Ahmed M Afifi, Aanuoluwapo Adeyimika Afolabi, Arya Afrooghe, Muhammad Sohail Afzal, Suneth Buddhika Agampodi, Feleke Doyore Agide, César Agostinis Sobrinho, Bright Opoku Ahinkorah, Danish Ahmad, Muayyad M Ahmad, Tauseef Ahmad, Elham Ahmadi, Mohadese Ahmadzade, Anisuddin Ahmed, Ayman Ahmed, Gasha Salih Ahmed, Ibrar Ahmed, Luai A Ahmed, Mehrunnisha Sharif Ahmed, Meqdad Saleh Ahmed, Muktar Beshir Ahmed, Nesredin Ahmed, Syed Anees Ahmed, Dolapo Emmanuel Ajala, Marjan Ajami, Roland Eghoghosoa Akhigbe, Omar Al Omari, Yazan Al Thaher, Omar Ali Mohammed Al Zaabi, Khurshid Alam, Zufishan Alam, Amani Alansari, Fahmi Y Al-Ashwal, Laila Ismael Al-Daken, Wafa A Aldhaleei, Shereen M Aleidi, Ayman Al-Eyadhy, Abdelazeem M Algammal, Mohammed Khaled Al-Hanawi, Dari Alhuwail, Abid Ali, Mohammed Usman Ali, Syed Shujait Ali, Waad Ali, Samah W Al-Jabi, Ahmad Alkhatib, Nihad A Almasri, Hesham M Al-Mekhlafi, Mohmmad Minwer Alnaeem, Hasan Yaser Alniss, Mahmoud A Alomari, Mohammad R Alosta, Saleh A Alqahtani, Ahmad Rajeh Al-Qudimat, Ahmad Alrawashdeh, Intima Alrimawi, Sahel Majed Alrousan, Salman Khalifah Al-Sabah, Awais Altaf, Alaa B Al-Tammemi, Elysia M Alvarez, Nelson Alvis-Guzman, Yaser Mohammed Al-Worafi, Hany Aly, Mohammad Sharif Ibrahim Alyahya, Abdallah Alzoubi, Karem H Alzoubi, Tarek Tawfik Amin, Alireza Amindarolzarbi, Shiva Aminnia, Nafiu Aminu, Mohammad Hosein Amirzade-Iranaq, Hubert Amu, Ganiyu Adeniyi Amusa, Robert Ancuceanu, Deanna Anderlini, Abhishek Anil, Hossein Ansariniya, Iyadunni Adesola Anuoluwa, Saeid Anvari, Saleha Anwar, Anayochukwu Edward Anyasodor, Juan Pablo Arab, Jalal Arabloo, Razman Arabzadeh Bahri, Elshaimaa A Arafa, Martina Arcieri, Hany Ariffin, Mesay Arkew, Santhosh Arul, Muhammad Abdul Basit Ashraf, Bernard Kwadwo Yeboah Asiamah-Asare, Haftu Asmerom Asmerom, Prince Atorkey, Maha Moh'd Wahbi Atout, Marcel Ausloos, Atalel Fentahun Awedew, Adedapo Wasiu Awotidebe, Beatriz Paulina Ayala Quintanilla, Seyed Mohammad Ayyoubzadeh, Amirali Azimi, Sadat Abdulla Aziz, Ahmed Y Azzam, Muhammad Badar, Ashish D Badiye, Alaa Aboelnour Badran, Fereshteh Baghizadeh, Ruhai Bai, Atif Amin Baig, Auwal Adam Bala, Mohammadreza Balooch Hasankhani, Soham Bandyopadhyay, Aleksandra Barac, Mainak Bardhan, Suzanne Lyn Barker-Collo, Hiba Jawdat Barqawi, Amadou Barrow, Shahid Bashir, Afisu Basiru, Pritish Baskaran, Mohammad-Mahdi Bastan, Matteo Bauckneht, Feyisa Shasho Bayisa, Babak Behnam, Amir Hossein Behnoush, Payam Behzadi, Luis Belo, Habib Benzian, Ajeet Singh Bhadoria, Akshaya Srikanth Bhagavathula, Nickhill Bhakta, Neeraj Bhala, Kayleigh Bhangdia, Ravi Bharadwaj, Prarthna V Bhardwaj, Sonu Bhaskar, Ajay Nagesh Bhat, Vivek Bhat, Shuvarthi Bhattacharjee, Gurjit Kaur Bhatti, Jasvinder Singh Bhatti, Mohiuddin Ahmed Bhuiyan, Soumitra S Bhuyan, Raluca Bievel-Radulescu, Catherine Bisignano, Mohammad Shahangir Biswas, Monirujjaman Biswas, Bruno Bizzozero-Peroni, Tone Bjørge, Hamed Borhany, Samuel Adolf Bosoka, Alejandro Botero Carvajal, Souad Bouaoud, Marija M Bozic, Dejana Braithwaite, Hermann Brenner, Felix Busch, Maria Teresa Bustamante-Teixeira, Yasser Bustanji, Ismael Campos-Nonato, Yubin Cao, Angelo Capodici, Giulia Carreras, Márcia Carvalho, Carlos A Castañeda-Orjuela, Ferrán Catalá-López, Maria Sofia Cattaruzza, Luca Cegolon, Francieli Cembranel, Ester Cerin, Achille Cernigliaro, Aditya Chakraborty, Sandip Chakraborty, Rama Mohan Chandika, Vijay Kumar Chattu, Anis Ahmad Chaudhary, Sirshendu Chaudhuri, Akhilanand Chaurasia, Hana Chen, Kai Chen, Meng Xuan Chen, Patrick R Ching, Jesus Lorenzo Chirinos-Caceres, William C S Cho, Bryan Chong, Yuen Yu Chong, Hitesh Chopra, Shivani Chopra, Hou In Chou, Eric Chung, Sunghyun Chung, Alyssa Columbus, Joao Conde, Vera Marisa Costa, Natalia Cruz-Martins, Bashir Dabo, Lucio D'Anna, Pojsakorn Danpanichkul, Aso Mohammad Darwesh, Mohsen Dashti, Dimash Davletov, Frances E Dean, Edward Christopher Dee, Sindhura Deekonda, Abel Desalegn Demeke, Edgar Denova-Gutiérrez, Rupak Desai, Devananda Devegowda, Syed Masudur Rahman Dewan, Arkadeep Dhali, Amol S Dhane, Meghnath

Dhimal, Sameer Dhingra, Marcello Di Pumpo, Luis Antonio Diaz, Michael J Diaz, Francesco Dondi, Wendel Mombaque dos Santos, Ojas Prakashbhai Doshi, Leila Doshmangir, Taiwo Omotayo Dosumu, Menayit Tamrat Dresse, John Dube, Senbagam Duraisamy, Oyewole Christopher Durojaiye, Arkadiusz Marian Dziedzic, Abdel Rahman E'mar, Behrad Eftekhari, Ashkan Eighaei Sedeh, Michael Ekholuenetale, Rabie Adel El Arab, Abdelfatteh EL Omri, Iman El Sayed, Maysaa El Sayed Zaki, Reza Elahi, Noha Mousaad Elemam, Muhammed Elhadi, Sherif Elkannishy, Ahmed O Elmehrath, Adel B Elmoselhi, Yasser Mohamed El-Sherbiny, Abdelgawad Salah Abdelgawad Eltahawy, Mohd Elmagzoub Eltahir, Syed Emdadul Haque, Theophilus I Emeto, Talha Bin Emran, Destaw Endeshaw, Majid Eslami, Sayeh Ezzikouri, Heidar Fadavian, Adeniyi Francis Fagbamigbe, Ayesha Fahim, Saman Fahimi, Aamir Fahira, Luca Falzone, Alireza Farahani, Seyed Nooreddin Faraji, Ebrahim Farashi, Zaki Farhana, MoezAlIslam Ezzat Mahmoud Faris, Andre Faro, Umar Farooque, Hossein Farrokhpour, Kiana Fasihi, Emmanuel Toluwani Fasusi, Hamed Fattahi, Pietro Ferrara, Nuno Ferreira, Getahun Fetensa, Florian Fischer, James L Fisher, Olumuyiwa Shola Folayan, Lisa M Force, Ali Forouhari, Maryam Fotouhi, Takeshi Fukumoto, Ami Fukunaga, Muktar A Gadanya, Márió Gajdács, Silvano Gallus, Adamu Usman Gamawa, Shivaprakash Gangachannaiah, Mounika Gangireddy, Jacopo Garlasco, Rupesh K Gautam, Feven Sahle Gebre, Miglas Welay Gebregergis, Haftay Gebremedhin Gebreslassie, Gebremariam Wulie Geremew, Sadegh Ghafarian, Lobna Faiz Gharaibeh, Haniyeh Ghasrsaz, Fariba Ghassemi, Ramy Mohamed Ghazy, Nasim Gholizadeh, Elena Ghotbi, Artyom Urievich Gil, Syed Abdullah Gilani, Alem Abera Girmay, Alessandro Girombelli, Anil Kumar Goel, Davide Golinelli, Giuseppe Gorini, Shi-Yang Guan, Giovanni Guarducci, Zheng Guo, Zhifeng Guo, Bhawna Gupta, Sapna Gupta, Reyna Alma Gutiérrez, Adrina Habibzadeh, Farrokh Habibzadeh, Parham Habibzadeh, Tesfahun Simon Simon Hadaro, Najah R Hadi, Dariush Haghmorad, Nguyen Hai Nam, Haimanot Ewnetu Hailu, Hassen Mosa Mosa Halil, Islam M Hamad, Randah R Hamadeh, Nadia M Hamdy, Sajid Hameed, Nasrin Hanifi, Josep Maria Haro, Ahmed I Hasaballah, Md Kamrul Hasan, Fatemeh Hasani, Alireza Hasanzadeh, Abdiwahab Hashi, Md Saquib Hasnain, Treska S Hassan, Mahgol Sadat Hassan Zadeh Tabatabaei, Johannes Haubold, Simon I Hay, Guohua He, Qiang He, Wen-Qiang He, Behzad Heibati, Claudiu Herteliu, Hamed Hesami, Mojtaba Heydari, Kamal Hezam, Yuta Hiraike, Mai Hoang, Ramesh Holla, Mehdi Hoseinzadeh, H Dean Hosgood, Alamgir Hossain, Md Mahbub Hossain, Md Sabbir Hossain, Mohammad Bellal Hossain, Mohammad-Salar Hosseini, Ahmad Hosseinzadeh Adli, Vivian Chia-rong Hsieh, Chengxi Hu, Salman Hussain, Dursa Hussein, Nawfal R Hussein, Mohamed Ibrahim Husseiny, Hong-Han Huynh, Ivo Iavicoli, Segun Emmanuel Ibitoye, Reem Ibrahim, Anel Ibrayeva, Francisco Javier Idalsoaga, Pulwasha Maria Iftikhar, Adalia Ikiroma, Olayinka Stephen Ilesanmi, Irena M Ilic, Milena D Ilic, Muhana Fawwazy Ilyas, Salim Ilyasu, Mustapha Immurana, Lucius Chidiebere Imoh, Javed Iqbal, Mustafa Alhaji Isa, Md Rabiul Islam, Faisal Ismail, Chidozie Declan Iwu, Mahalaxmi Iyer, Louis Jacob, Abdollah Jafarzadeh, Haitham Jahrami, Abhishek Jaiswal, Mihajlo Jakovljevic, Ali Jaliliyan, Mohamed L Jalloh, Qazi Mohammad Sajid Jamal, Syed Sarmad Javaid, Shubha Jayaram, Ruwan Duminda Jayasinghe, Shuai Jin, Wenyi Jin, Jost B Jonas, Tamas Joo, Jobin Jose, Abel Joseph, Nitin Joseph, Charity Ehimwenma Joshua, Mikk Jürisson, Malik E Juweid, Ali Kabir, Leila R Kalankesh, Sivesh Kathir Kamarajah, Arun Kamireddy, Ramat T. Kamorudeen, Fatemeh Kanaani Nejad, Samuel Berchi Kankam, Kehinde Kazeem Kanmodi, Neeti Kapoor, Jafar Karami, Reema A Karasneh, André Karch, Aliasghar Karimi, Mohammad Amin Karimi, Sahand Karimzadhagh, Tomasz M Karpiński, Faizan Zaffar Kashoo, Joonas H Kauppila, Navjot Kaur, Mohd Adnan Kausar, Foad Kazemi, Fekadu Abera Kebede, Ariz Keshwani, Emmanuelle Kesse-Guyot, Yousef Saleh Khader, Himanshu Khajuria, Asaad Khalid, Hazim O. Khalifa, Anita Khalili, Pantea Khalili, Ghazaleh Khalili-Tanha, Ajmal Khan, Gulfaraz Khan, Moien AB Khan, Shaghayegh Khanmohammadi, Moawiah Mohammad Khatatbeh, Mahalaqua Nazli Khatib, Hamid Reza Khayat Kashani, Daniel Kheradmand,

Feriha Fatima Khidri, Atulya Aman Khosla, Mohammad Ali Khosravi, Jagdish Khubchandani, Kwanghyun Kim, Yun Jin Kim, Yun Seo Kim, Adnan Kisa, Sezer Kisa, Shivakumar KM, Jonathan M Kocarnik, Michail Kokkorakis, Jianqiu Kong, Karel Kostev, Ashwin Laxmikant Kotnis, Sindhura Lakshmi Koulmane Laxminarayana, Irene Akwo Kretchy, Kewal Krishan, Raja Amir Hassan Kuchay, Mohammed Kuddus, Mukhtar Kulimbet, Dewesh Kumar, Satyajit Kundu, Setor K Kunutsor, Christina Yeni Yeni Kustanti, Tezer Kutluk, Frank Kyei-Arthur, Carlo La Vecchia, Muhammad Awwal Ladan, Chandrakant Lahariya, Daphne Teck Ching Lai, Hanpeng Lai, Balzhan Lakanova, Judit Lám, Francesco Lanfranchi, Savita Lasrado, Mahrukh Latif, Paolo Lauriola, Basira Kankia Lawal, Huu-Hoai Le, Minh Huu Nhat Le, Nhi Huu Hanh Le, Thao Thi Thu Le, Caterina Ledda, Sang-woong Lee, Shaun Wen Huey Lee, Awol Yemane Legesse, James Leigh, Elvynna Leong, Wang-Zhong Li, Zhengrui Li, Zhihui Li, Queran Lin, Chaojie Liu, Jue Liu, Liu Liu, Erand Llanaj, Jailos Lubinda, Angelina M Lutambi, Miltiadis D Lytras, Ellina Lytvyak, Zheng Feei Ma, Raymond Saa-Eru Maalman, Farzan Madadizadeh, Seyedeh Panid Madani, Alireza Mafi, Abdulahi Abdiwali Mahamed, Mansour Adam Mahmoud, Rashidul Alam Mahumud, Abdelrahman M Makram, Omar M Makram, Satyaveni Malasala, Hadi Maleki-Kakelar, Kashish Malhotra, Ahmad Azam Malik, Deborah Carvalho Malta, Vahid Mansouri, Marjan Mansourian, Lorenzo Giovanni Mantovani, changkun mao, Hamid Reza Marateb, Basma Hamed Marghani, Mirko Marino, Miquel Martorell, Roy Rillera Marzo, Sammer Marzouk, Yasith Mathangasinghe, Medha Mathur, Fernanda Penido Matozinhos, Indu Liz Matthew, Miranda L May, Steven M McPhail, Enkeleint A Mechili, Jitendra Kumar Meena, Elahe Meftah, Riffat Mehboob, Asim Mehmood, Tesfahun Mekene Meto, Hadush Negash Meles, Max Alberto Mendez-Lopez, Walter Mendoza, Ritesh G Menezes, Dominik Menges, Endalkachew Worku Mengesha, Alexios-Fotios A Mentis, Sultan Ayoub Ayoub Meo, Atte Meretoja, Tuomo J Meretoja, Tomislav Mestrovic, Mohamed M. M. Metwally, Tomasz Miazgowski, Irmina Maria Michalek, Ted R Miller, Giuseppe Minervini, Mojgan Mirghafourvand, Awoke Misganaw, Prasanna Mithra, Ashraf Mohamadkhani, Mona Gamal Mohamed, Nouh Saad Mohamed, Karzan Abdulmuhsin Mohammad, Taj Mohammad, Sakineh Mohammad-Alizadeh-Charandabi, Abdolreza Mohammadi, Mohammad Kazem Mohammadi, Abdollah Mohammadian-Hafshejani, Mustapha Mohammed, Shafiu Mohammed, Amin Mohsenzadeh, Ali H Mokdad, Lorenzo Monasta, Mohammad Ali Moni, Paula Moraga, Shane Douglas Morrison, Mahmoud M Morsy, Ahmed Msherghi, George Duke Mukoro, Admir Mulita, Efren Murillo-Zamora, Christopher J L Murray, Ali Mushtaq, Saima Mushtaq, Woojae Myung, Amin Nabavi, Ahamarshan Jayaraman Nagarajan, Mukhammad David Naimzada, Hae Sung Nam, Shumaila Nargus, Abdulqadir J Nashwan, Mahmoud Nassar, Zuhair S Natto, Javaid Nauman, Biswa Prakash Nayak, Md Fahad Shahariar Nayon, Pacifique Ndishimye, Masoud Negahdary, Ionut Negoi, Gaurav Nepal, Jean Claude Semuto Semuto Ngabonziza, Cuong Tat Nguyen, Dang Nguyen, Kieu Viet Nhi Nguyen, Nhan Nguyen, Tuan Thanh Nguyen, Van Thanh Nguyen, Robina Khan Niazi, Luciano Nieddu, Nasrin Nikravangolsefid, Vikram Niranjan, Jean Marie Vianney Niyonsenga, Mohammadamin Noorafrooz, Masoud Noroozi, Nawsherwan NOT APPLICABLE, Chisom Adaobi Nri-Ezedi, Fred Nugen, Dieta Nurrika, Chimezie Igwegbe Nzoputam, Ogochukwu Janet Nzoputam, Bogdan Oancea, Ismail A Odetokun, In-Hwan Oh, Sylvester Reuben Okeke, Osaretin Christabel Okonji, Andrew T Olagunju, Timothy Olusegun Olusegun Olanrewaju, Matthew Idowu Olatubi, Hany A Omar, Abidemi E Omonisi, Obinna E Onwujekwe, Marcel Opitz, Michal Ordak, Verner N Orish, Atakan Orscelik, Esteban Ortiz-Prado, Augustus Osborne, Alaa AM Osman, Samuel M Ostroff, Uchechukwu Levi Osuagwu, Elham H Othman, Oche Joseph Otorkpa, Amel Ouyahia, Mayowa O Owolabi, Oladayo Ayobami Oyebanji, Kolapo Oyebola, Ilker Ozsahin, Mahesh P A, Alicia Padron-Monedero, Jagadish Rao Padubidri, Tamás Palicz, Sujogya Kumar Panda, Songhomitra Panda-Jonas, Deepshikha Pande Katare, Seithikurippu R Pandi-Perumal, Leonidas D Panos, Ioannis

Pantazopoulos, Mario Virgilio Papa, Parinaz Paranjkhoo, Romil R Parikh, Roberto Passera, Riya Jayesh Patel, Shankargouda Patil, Apurba Patra, Hamidreza Pazoki Toroudi, Umberto Pensato, Mario F P Peres, Simone Perna, Richard G Pestell, Fanny Emily Petermann-Rocha, Hoang Tran Pham, Daniela Pierannunzio, Julian David Pillay, Zahra Zahid Piracha, Saeed Pirouzpanah, Maarten J Postma, Jalandhar Pradhan, Chandra P Prasad, Akila Prashant, Elton Junio Sady Prates, Natalie Pritchett, Jagadeesh Puvvula, Asma Saleem Qazi, Suli Qiu, Karzan Qurbani, Hadi Raeisi Shahraki, Ata Rafiee, Alireza Rafiei, Pracheth Raghuveer, Hawbash Mohammed-Amin Rahim, Md Mijanur Rahman, Mohammad Meshbahur Rahman, Amir Masoud Rahmani, Masoud Rahmati, Diego Raimondo, Ivano Raimondo, Sathish Rajaa, Mohammad Amin Rajizadeh, Chitra Ramasamy, Shakthi Kumaran Ramasamy, Pushkal Sinduvadi Ramesh, Zahra Ramezani, Rishabh Kumar Rana, Nemanja Rancic, Fatemeh - Ranjbar Noei, Christopher Rao, Kumuda Rao, Mithun Rao, Sowmya J Rao, Vahid Rashedi, Mohammad-Mahdi Rashidi, Devarajan Rathish, Ilari Rautalin, Salman Rawaf, Lal Rawal, Elrashdy Redwan, Sanika Rege, Ana Reis-Mendes, Stefano Restaino, Nima Rezaei, Tércia Moreira Ribeiro da Silva, Maximiliano Ribeiro Ribeiro Guerra, Moattar Raza Rizvi, Thomas J Roberts, Hermano Alexandre Lima Rocha, João Rocha Rocha-Gomes, Thales Philipe Rodrigues da Silva, Ravi Rohilla, Peter Rohloff, Kevin T Root, Gholamreza Roshandel, Amirhossein Roshanshad, Guido Rovera, Nitai Roy, Sharmistha Roy, Maha Mohamed Saber-Ayad, Cameron John Sabet, Basema Ahmad Saddik, Tarannom Sadegh, Umar Saeed, Amene Saghazadeh, Fatemeh Saheb Sharif-Askari, Amirhossein Sahebkar, Soumya Swaroop Sahoo, Mirza Rizwan Sajid, Leili Salehi, Amir Salek Farrokhi, Marwa Rashad Salem, Pouria Samadi, Jayami Eshana Samaranayake, Abdallah M Samy, Juan Sanabria, Milena M Santric-Milicevic, Aswini Saravanan, Tanmay Sarkar, Gargi Sachin Sarode, Sachin C Sarode, Arash Sarveazad, Michele Sassano, Maheswar Satpathy, Reza Sattarpour, Benedikt Michael Schaarschmidt, Ione Jayce Ceola Schneider, Art Schuermans, Durairaj Sekar, Mario Šekerija, Mohammad H Semreen, Ashenafi Kibret Sendekie, Yigit Can Senol, Dragos Serban, Yashendra Sethi, Seyed Mohammad Seyed Alshohadaei, Homa Seyedmirzaei, Allen Seylani, Maryam Shabany, Mahan Shafie, Muhammad Shahab, Samiah Shahid, Moyad Jamal Shahwan, Muhammad Aaqib Shamim, Elahe Shams, Mehran Shams-Beyranvand, Anas Shamsi, Alfiya Shamsutdinova, Dan Shan, Mohd Shanawaz, Abhishek Shankar, Mohammed Shannawaz, Amin Sharifan, Manoj Sharma, Rajesh Sharma, Ujjawal Sharma, Vishal Sharma, Shamee Shastry, Ramzi Shawahna, Samendra P Sherchan, Pavanchand H Shetty, Premalatha K Shetty, Suraj S Shetty, Mosa Shibani, Aminu Shittu, Velizar Shivarov, Sinegugu Nosipho Shongwe, Sina Shool, Seyed Afshin Shorofi, Sunil Shrestha, Kerem Shuval, Emmanuel Edwar Siddig, Ahmed Kamal Siddiqi, Luís Manuel Lopes Rodrigues Silva, Harmanjit Singh, Jasvinder A Singh, Paramdeep Singh, Prashant Kumar Singh, Puneetpal Singh, Satwinder Singh, Surjit Singh, Freddy Sitas, Dagne Feleke Siyoum, Anna Aleksandrovna Skryabina, Amanda E Smith, Farrukh Sobia, Solikhah Solikhah, Aayushi Sood, Suresh Kumar Srinivasamurthy, Devin Bailey Srivastava, Kurt Straif, Auwal Garba Suleiman, Muritala Odidi Suleiman Odidi, Muhammad Suleman, Desy Sulistiyorini, Anusha Sultan Meo, Hani Susianti, Lukasz Szarpak, Sree Sudha T Y, Payam Tabaee Damavandi, Mohammad Tabish, Seyed Reza Taha, Yasaman Taheri Abkenar, Iman M Talaat, Mircea Tampa, Jacques Lukenze Tamuzi, Ker-Kan Tan, Manoj Tanwar, Saba Tariq, Seyed Mohammad Tavangar, Birhan Tsegaw Taye, Abainash Tekola, Mohamad-Hani Temsah, Dufera Rikitu Terefa, Wegen Beyene Tesfamariam, Tenaw Yimer Tiruye, Roman Topor-Madry, Adetunji T Toriola, Mathilde Touvier, Marcos Roberto Tovani-Palone, Bach Xuan Tran, Thang Huu Tran, Domenico Trico, Aristidis Tsatsakis, Mike Tuffour Amirikah, Lawrence Sena Tuglo, Srikanth Umakanthan, Lawan Umar, Shehu Salihu Umar, Umar Muhammad Umar, Brigid Unim, Bhaskaran Unnikrishnan, Dinesh Upadhya, Asokan Govindaraj Vaithinathan, Jef Van den Eynde, Javad Varasteh, Shoban Babu Varthya, Balachandar Vellingiri, Madhur Verma, Massimiliano Veroux, Georgios-

Ioannis Verras, Simone Villa, Gabriela Ines Villanueva, Giuseppe Vizzielli, Vasily Vlassov, Stein Emil Vollset, Theo Vos, Yasir Waheed, Cong Wang, Ruixuan Wang, Xing Wang, Yanzhong Wang, Paul Ward, Kosala Gayan Weerakoon, Ronny Westerman, Anggi Lukman Wicaksana, Dakshitha Praneeth Wickramasinghe, Nuwan Darshana Darshana Wickramasinghe, Karn Wijarnpreecha, Peter Willeit, Marcin W Wojewodzic, Axel Walter Wolf, James Fan Wu, Libo Xu, Suowen Xu, Mingyang Xue, Mukesh Kumar Yadav, Galal Yahya, Amir Yarahmadi, Sanni Yaya, Saber Yezli, Biksegn Asrat Yirdaw, Dong Keon Yon, Naohiro Yonemoto, Zabihollah Yousefi, Vesna Zadnik, Syed Nabeel Zafar, Manijeh Zaghampour, Fathiah Zakham, Burhan Abdullah Zaman, Sojib Bin Zaman, Michael Zastrozhin, Mohammed Zawiah, Mohammed G M Zeariya, Alemu Birara Zemariam, Sebastian Zensen, Jianrong Zhang, Xiaoyi Zhang, Zhi-Jiang Zhang, David X Zheng, Anthony Zhong, Claire Chenwen Zhong, Jiayan Zhou, Bin Zhu, Magdalena Zielińska, Osama A Zitoun, Rafat Mohammad Zrieq, Mohammed Zuber, Sa'ed H Zyoud, Shaher H Zyoud

Managing the estimation or publications process

Kayleigh Bhangdia, Lisa M Force, Simon I Hay, Jonathan M Kocarnik, Miranda L May, Ali H Mokdad, Christopher J L Murray, Samuel M Ostroff, Maja Pasovic, Amanda E Smith, Stein Emil Vollset, Theo Vos
